# Comparative transcriptome analysis of equine alveolar macrophages

**DOI:** 10.1111/evj.12584

**Published:** 2016-07-09

**Authors:** A.E. Karagianni, R. Kapetanovic, K.M. Summers, B.C. McGorum, D.A. Hume, R.S. Pirie

**Affiliations:** ^1^ Roslin Institute and Royal (Dick) School of Veterinary StudiesUniversity of EdinburghUK; ^2^ Present address: Moredun Research InstitutePentlands Science Park, Bush Loan, PenicuikMidlothianEH26 0PZUK; ^3^ Present address: Institute for Molecular BioscienceUniversity of QueenslandSt LuciaQueensland4072Australia

**Keywords:** horse, lungs, peritoneal cavity, lipopolysaccharide, microarray

## Abstract

**Reasons for performing study:**

Alveolar macrophages (AMs) are the first line of defence against pathogens in the lungs of all mammalian species and thus may constitute appropriate therapeutic target cells in the treatment and prevention of opportunistic airway infections. Therefore, acquiring a better understanding of equine macrophage biology is of paramount importance in addressing this issue in relation to the horse.

**Objectives:**

To compare the transcriptome of equine AMs with that of equine peritoneal macrophages (PMs) and to investigate the effect of lipopolysaccharide (LPS) on equine AM.

**Study design:**

Gene expression study of equine AMs.

**Methods:**

Cells from both bronchoalveolar and peritoneal lavage fluid were isolated from systemically healthy horses that had been submitted to euthanasia. Cells were cryopreserved. RNA was extracted and comparative microarray analyses were performed in AMs and PMs, and in AMs treated and untreated with LPS. Comparisons with published data derived from human AM studies were made, with particular focus on LPS‐induced inflammatory status.

**Results:**

The comparison between AMs and PMs revealed the differential basal expression of 451 genes. Gene expression analysis revealed an alternative (M2) macrophage polarisation profile in AMs and a hybrid macrophage activation profile in PMs, a phenomenon potentially attributable to a degree of induced endotoxin tolerance. The gene expression profile of equine AMs following LPS stimulation revealed significant changes in the expression of 240 genes, including well‐known upregulated inflammatory genes. This LPS‐induced gene expression profile of equine AMs more closely resembles that of human rather than murine macrophages.

**Conclusions:**

This study improves current understanding of equine macrophage biology. These data suggest that the horse may represent a suitable animal model for the study of human macrophage‐associated lung inflammation and data derived from human macrophage studies may have significant relevance to the horse.

## Introduction

The mucosal surface of the lung is continuously exposed to potential pathogens, proinflammatory particulates and inhaled toxins. In addition to the barriers provided by surfactants and mucus, alveolar macrophages (AMs) provide a first line of immune defence and an efficient mechanism of particulate and pathogen clearance. They also play a key role in the initiation and propagation of both acute and chronic lung injury‐induced pathology 1. The function and gene expression of AMs in other species have proved to be distinct from those of macrophages derived from other anatomic locations, reflecting both a unique environment and a distinct pathway of differentiation 2, 3. Although we have previously identified and reported various functional and phenotypic differences between equine AMs and peritoneal macrophages (PMs) 4, to date no published data exist on such a comparison in the horse at the level of cellular global gene expression. We consider this to be a natural progression from the previously identified differences between these cell populations. Furthermore, recognition of the potential importance of endotoxin in various equine airway diseases is evidenced by the publication of numerous studies reporting the transcriptomic and protein translational response of the equine AM to a lipopolysaccharide (LPS) stimulus 4, 5. However, such reports have focused on the transcription and translation of specific genes and inflammatory proteins. We therefore considered the generation of LPS‐induced global gene expression data in the equine AM to be justified as an exercise which permits a more detailed comparison with the LPS response of AMs derived from other species. Indeed, despite the likely critical importance of AMs in equine airway defence, much of our presumed knowledge of equine AM biology is extrapolated from data derived from other species. Such translation of data derived from one species to another assumes a degree of conservation of key biological processes; however, this assumption is often unsubstantiated. For example, despite the common use of murine model‐derived data to improve our understanding of human innate immunity, there is an ever‐increasing appreciation that significant differences exist between man and mice with respect to the gene expression profiles associated with certain pathologies 6, 7. Therefore, an appreciation of both similarities and dissimilarities between the equine AM and AMs derived from other species is a necessary prerequisite to the translation of data derived from other species to the horse, and vice versa.

To further characterise the specific biology of the equine AM, we analysed the basal gene expression of equine AMs and made comparisons with, firstly, the basal gene expression of an alternative macrophage population (PMs) and, secondly, the LPS‐induced gene expression of equine AMs. Additionally, to further investigate the appropriateness of the horse as an abundant source of cells for translational studies of human AM biology, we compared data on the LPS‐induced gene expression of equine AMs with previously published data on LPS‐induced AM gene expression in both man and mice.

## Materials and methods

### Cell isolation

Bronchoalveolar and peritoneal lavage samples were obtained post‐mortem from 5 to 3 systemically healthy horses, respectively. The horses (7 female and one male; median age: 17 years [range: 4–20 years]) were submitted to euthanasia at the Equine Hospital at the Royal (Dick) School of Veterinary Studies, Edinburgh University, Edinburgh, UK) by the i.v. administration of secobarbitone and cinchocaine (Somulose^™^ 1). The reasons for euthanasia were predominantly related to orthopaedic or behavioural issues. None of the horses were submitted to euthanasia as a result of underlying intestinal or respiratory disease.

Cells from both bronchoalveolar and peritoneal lavage fluid were isolated and cryopreserved as previously described 4. Prior to cryopreservation, an aliquot of lavage fluid was retained for cytological analysis and differential cell count calculation, achieved by counting 500 cells under light microscopy 4, 8. Horses were considered free from inflammatory airway disorders based on the respective differential cell ratios not exceeding the following cut‐off values: neutrophils, 10%; mast cells, 5%, and eosinophils, 2% 9, 10.

### Cell culture

Thawed cells were seeded in duplicate (1 × 10^6^ cells/mL) in Petri dishes in complete medium: RPMI‐1640 medium supplemented with GlutaMAX^™^‐I Supplement^2^, penicillin/streptomycin^2^ and 10% heat‐inactivated horse serum (HS) (cat. no. H1138^3^) and incubated at 37°C and 5% carbon dioxide overnight. The next day, nonadherent cells were removed and fresh complete medium was added before adherent cells were stimulated with LPS (100 ng/mL) from *Salmonella enterica* serotype Minnesota Re 595 (L9764)^3^. Supernatant from the plates was collected prior to and 6 h following LPS stimulation.

### RNA extraction

RNA was extracted from both AMs and PMs using 1 mL RNA‐Bee^4^. RNA concentration and purity were measured using ND‐1000 Nanodrop^5^. RNA integrity was also confirmed with the RNA 6000 Pico Assay^6^; an RNA integrity value of >7 was considered appropriate for microarray.

### Microarray assay

Microarrays (https://www.ncbi.nlm.nih.gov/geo/query/acc.cgi?acc=GSe69871) were processed by Edinburgh Genomics (http://genomics.ed.ac.uk). The libraries prepared from RNA samples (50 ng) were hybridised to Equine Gene 1.1 or to 1.0 ST Array Strips from Affymetrix^7^, according to the manufacturer's instructions. The same group of 30,559 probesets was used in both array types. The Affymetrix analysis procedure is a single‐dye protocol, which thereby avoids problems of dye bias. Image preparation and the CEL files required for analysis were produced using Affymetrix Gene Chip Command Console Version 3.0.1^7^. Expression values were normalised according to the RMA algorithm 11. The Equine Gene 1.1 ST Array Strip was used for basal and LPS‐induced AM gene expression analyses in 3 horses. Thereafter, all subsequent analyses (AMs and PMs) were conducted using 1.0 ST Array Strips. CEL files were imported to Partek Genomic Suite 6.6^8^ for the microarray data analysis, using default parameters. The human microarray datasets used for comparison were derived from a human study 12 (GDS4419; platform: GPL570 [HG‐U133_Plus_2]; Affymetrix Human Genome U133 Plus 2.0 Array^7^) and were submitted to the same analysis. Consistent with cut‐off values widely used in microarray data analysis, changes of at least 2‐fold in magnitude and a P value of 0.05 were applied in all experiments with the exception of comparisons between AMs and PMs, in which the extensive gene list generated was considered to justify the use of more rigorous cut‐off points (a 9‐fold change and a P value of 0.01). In the first analysis, 5 untreated AMs were compared with 3 untreated PMs, whereas in the second analysis, 5 untreated AMs were compared with 5 LPS‐treated AMs.

A network analysis of expression data was performed in BioLayout Express^3D^ 13, whereby pairwise Pearson correlation coefficients (r) were calculated and a threshold of r ≥ 0.93 was chosen for the transcript‐to‐transcript comparison across array samples. The resulting network graph consists of nodes (representing transcripts) and edges, representing correlations above the threshold between the expression patterns of the transcripts. The Markov cluster algorithm (MCL) was used with an inflation value of 2.2 13 to identify groups of tightly coexpressed genes. Clusters are numbered according to the number of transcripts they contain: Cluster 1 has the greatest number of transcripts. Transcripts with a dynamic range of <1.5 were removed from the analysis. Only clusters that showed consistent up‐ or downregulation of genes across all samples in the group were analysed; any clusters resulting from aberrant expression in a single horse were excluded.

### Functional annotation

Initial annotation used the most recent Affymetrix annotation file imported into the Partek software^8^ or identified in the Affymetrix Netaffx site^7^ (http://www.affymetrix.com). Partek Genomic Suite Version 6.6^8^ was used to determine the biological processes of the genes included in the gene lists.

## Results

### Cell recovery and populations

Approximately 3 × 10^8^ AMs and 2 × 10^8^ PMs were isolated from each of the 8 horses. Differential cell counts of bronchoalveolar lavage fluid showed a mean ± s.d. of 69.7 ± 7.2% macrophages, 22.0 ± 9.0% lymphocytes, 3.5 ± 5.0% neutrophils and 4.8 ± 3.0% mast cells. In the peritoneal lavage fluid, a mixed population of 43.7 ± 13.1% macrophages, 10.3 ± 7.6% lymphocytes and 46.0 ± 19.1% neutrophils was identified. Following overnight culture, nonadherent cells were removed. More than 85% of adherent cells, from both alveolar and peritoneal lavages, were identified morphologically as macrophages after the culture plates had been submitted to Leishman staining. Cell viability on the day of cell harvesting exceeded 90% as assessed by Trypan blue staining.

### Gene expression in equine AMs and PMs

**Figure 1:**
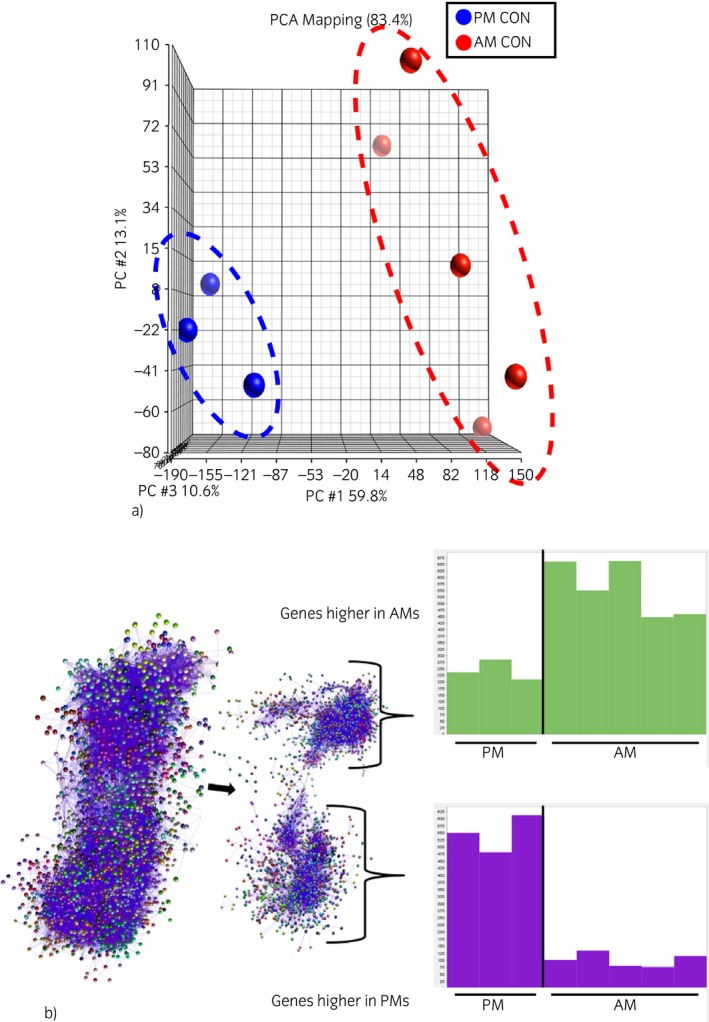
Alveolar macrophages (AMs) and peritoneal macrophages (PMs) display different gene expression profiles at homeostasis. (a) Principal component analysis was conducted in 5 samples of AMs and 3 samples of PMs. (b) Network analysis of genes differentially expressed in AMs and PMs at homeostasis. The graph was generated by BioLayout Express^3D^ software (MCL 2.2, r ≥ 0.93). Nodes with the same colour belong to the same cluster of coexpressed genes. The graphs at the right show expression profiles of genes expressed more highly in AMs and PMs, respectively. The *x*‐axis shows each horse and the *y*‐axis shows the normalised relative intensity of expression in that horse.

Figure 1(a) shows a principal component analysis (PCA) of the combined AM and PM gene expression results. A statistically significant difference in the expression of 451 (P<0.01, >9‐fold change) transcripts was identified between AMs and PMs; however, few had informative annotation (Supplementary Item 1). A more extensive gene list with less stringent cut‐off levels (P<0.05 and >2‐fold change) was also created (Supplementary Item 1). As an alternative approach, the same normalised data were analysed using BioLayout Express^3D^ software, with an MCL inflation value of 2.2, a Pearson correlation coefficient threshold of 0.93 and the smallest cluster set at 3 nodes. The graph created consisted of a total of 12,341 nodes, connected with 1,947,031 edges. This provided a more in‐depth understanding of the gene expression profiles of the 2 cell types. Consistent with the results of the PCA, network analysis (Fig 1b) clearly distinguished 2 groups of clusters, of which one consisted of genes with higher expression in AMs and one consisted of genes with higher expression in PMs. The top clusters of transcripts with comparatively greater expression in AMs were numbers 2, 3, 4 and 5; those in PMs were numbers 1, 8, 15, 17 and 25. Total gene lists of these clusters of AMs and PMs are presented in Supplementary Item 1.

Analysis of AM gene expression suggested an important role of cellular metabolic processes, myeloid cell differentiation and immune response. In particular, the genes more highly expressed in AMs included genes specifically expressed by macrophages or encoding proteins highly involved in macrophage differentiation, such as *CSF1* and its receptor. Of transcripts showing relatively greater expression in AMs, many were clearly related to immune response. These included genes such as Mannose receptor C type 1 (*MRC1*), genes that play a role in the nuclear factor κB (NFκB) signalling pathway, the transforming growth factor‐β1 gene (*TGFB1*), several pattern recognition receptors (*TLR6*,* TLR7*,* TLR8*), members of the tumour necrosis factor (TNF) and TNF receptor superfamily, and interleukin *IL18* and interleukin receptors (*IL6R*,* IL17R*). In PMs, the genes most highly expressed (in particular the Cluster 1 expression profile) were associated with inflammatory defence and immune response. Examples of these included several chemotactic chemokines (*CXCL1*,* CXCL3*,* CXCL6*), and both pro‐ and anti‐inflammatory cytokines (*IL1A*,* IL1B*,* IL6*,* IL8*,* IL10*). The full list is presented in Supplementary Item 1.

### LPS‐induced gene expression in equine AMs

**Figure 2:**
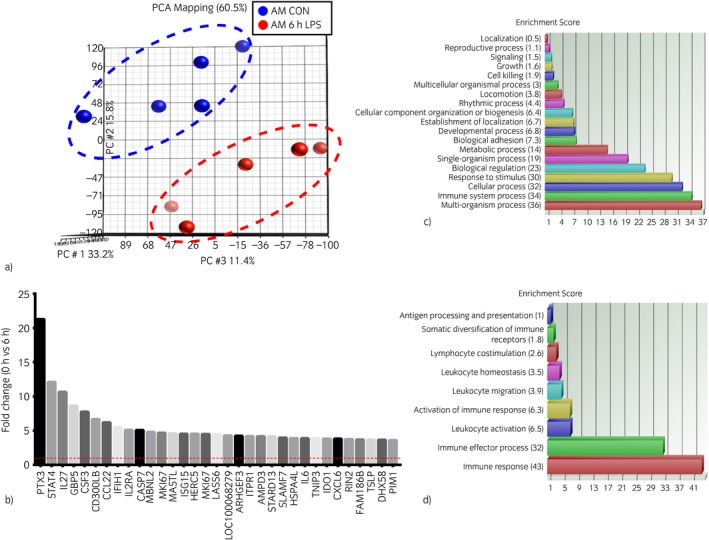
Lipopolysaccharide (LPS)‐induced gene expression in equine alveolar macrophages (AMs). (a) Principal component analysis indicates differences in gene expression profiles between LPS‐treated and untreated cells. Analysis was performed using Partek Genomic Suite Version 6.6.^8^ This is a 3D presentation of the microarray expression data. The relative difference in colour intensity for each group reflects the transcription of a 3D image onto a 2D format. (b) Top upregulated genes in AMs after 6 h of LPS stimulation. Genes are sorted in order of their fold change increase. (c) Main gene functional groups induced by LPS treatment in horse AMs. The gene ontology analysis tool from the Partek Genomic Suite^8^ was used. The histogram shows the main gene functional groups affected by LPS stimulation. (d) The immune‐related biological processes in which the upregulated genes are involved.

All of the LPS‐stimulated AMs showed an increase in TNFα secretion, as reported previously 4. LPS treatment of the AMs produced a radical change in gene expression, and the PCA of the resulting data clearly separated the baseline and LPS‐treated datasets (Fig 2a). The expression of 240 genes was significantly altered by LPS stimulation (P<0.05, >2‐fold change); 220 of these were upregulated and 20 were downregulated. By contrast with the set of genes differentially expressed between AMs and PMs, the LPS‐regulated AM‐derived genes were predominantly well annotated and previously identified in other species (Supplementary Item 2). The main gene functional groups induced by LPS are presented in Fig 2(c, d). In order to characterise these genes, the enrichment score of biological processes in the genes upregulated by LPS stimulation was calculated (Fig 2c). The second most important gene functional group induced by LPS includes all immune system processes. A deeper analysis (Fig 2d) revealed biological processes involved in this immune response such as leucocyte migration or activation.

The top 30 upregulated genes include well‐known inflammatory genes such as *TNF*,* PTX3*, signal transducer and activator of transcription protein 4 (*STAT4*), *IL6* and indoleamine 2,3‐dioxygenase 1 (*IDO1*), guanylate binding protein 5 (*GBP5*) (Fig 2b). The LPS‐induced expression of many genes related to apoptosis and programmed cell death detected in the current study was consistent with the well‐established phenomenon of constant interaction between inflammation and apoptosis, such as apoptotic peptidase activating factor 1 and caspase 7 14. Numerous transcription factors, such as *STAT4*,* ETV7* and basic leucine zipper transcriptional factor ATF‐like 3 (*BATF3*) were also found. A large number of genes responsible for the initiation and maintenance of inflammation were detected; examples included members of the TNF and TNF receptor superfamily (*TNF*,* CD40*,* TNFSF13B*), interleukins (*IL1α*,* IL1β*,* IL2Rα*,* IL6*,* IL27*) and chemoattractant chemokines (*CCL2*,* CXCL3*,* CXCL6*). The inducible gene list also included several secreted growth factors, including Schlafen family member 5 and *CSF3* (Supplementary Item 2). Network analysis using BioLayout Express^3D^ confirmed the results shown here (data not shown).

### LPS response in horse macrophages is similar to that in human macrophages

**Figure 3:**
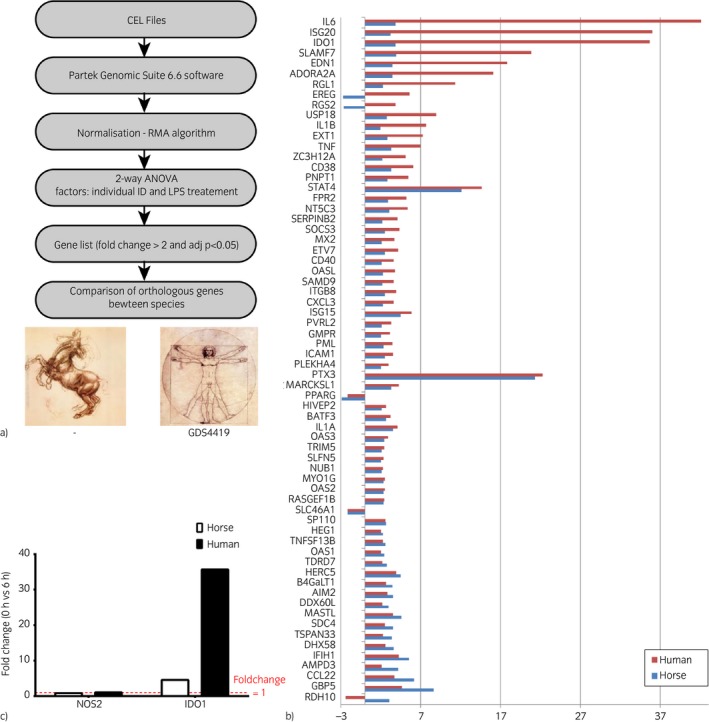
Lipopolysaccharide (LPS) response in horse macrophages is similar to that in human macrophages. (a) Strategy of identifying potential similarities in the gene expression of LPS‐treated alveolar macrophages (AMs) between species showed a list of orthologous genes to be similarly expressed between man and horses (drawings by Leonardo da Vinci http://www.leonardoda-vinci.org). (b) Fold change in gene expression between horse (current study) and human (GDS4419) microarray data derived from LPS‐treated and control AMs. Genes with a >2‐fold change, a P value of 0.05 and which showed statistically significant differences between the LPS‐treated AMs and controls, were included in the comparison. (c) *IDO1* and *NOS2* gene expression profiles were extracted from the 2 microarrays and plotted as fold change. The red dashed line represents no change in gene expression (fold change: 1).

Data for the equine LPS‐induced genes were compared with published data for human AMs (GDS4419) 12. Analysis revealed 66 orthologous genes induced in both species (Fig 3a). Many of these were shared with equine AMs, including *STAT4*,* IDO1* and *BATF3* (Fig 3b). *IDO1* and *NOS2* (nitric oxide synthase 2) are 2 genes that perfectly depict the differences between mice and man 15. Equine AMs and PMs, like human and porcine macrophages, did not metabolise arginine to produce nitric oxide (NO) as do mouse macrophages. Rather, human and porcine macrophages metabolise tryptophan through the induction of indoleamine dioxygenase (encoded by the *IDO* gene) 16, 17. Figure 3(c) shows the expression patterns of these genes in horse and human AMs. *IDO1* was upregulated after LPS stimulation in the horse, although to a lesser extent, whereas basal *NOS2* expression did not differ from that detected in LPS‐stimulated cells.

## Discussion

These data confirm that AMs in the horse, like those in other species, have a unique gene expression profile. The greater AM expression of genes related to macrophage differentiation and development, such as *CD14*,* CD68*,* CD71* and *CD163* 12, 18 complemented our previous results derived from flow cytometric analysis, which confirmed the cell surface expression of most of these markers 4. Some of the genes more highly expressed in AMs have previously been shown to have lung‐specific roles; these include *CS2RB*, which is shown in man to regulate surfactant homeostasis 19. Furthermore, the greater expression in AMs of several members of the scavenger receptor family, C‐type lectins and others (*CD14*,* CD40*,* CD68*,* CD163*,* MRS1*) essential for functions such as endocytosis, phagocytosis, adhesion and antigen recognition 20, was also consistent with our previous finding that AMs showed a greater capacity for phagocytosis compared with PMs 4. Moreover, analysis of pig macrophages 21, 22 revealed that AMs specifically over‐express many C‐type lectins compared with other tissue macrophage populations, an adaptation that may support the recognition of inhaled particles. Equine AMs also showed relatively greater expression of certain important pattern recognition receptors, findings which are likely to reflect the airway's status as a common site of airborne viral and bacterial challenge.

The greater expression of genes encoding *TGFB1*, which plays a key role in alveolar homeostasis 23, and the C‐type lectin *MRC1*, which is also highly expressed in porcine AMs 21, 22, is supportive of a predominantly M2 (alternatively activated) phenotype in equine AMs, which may play a role in suppressing spontaneous and/or over‐exuberant inflammation in the lung 24. By contrast, the highly expressed transcripts identified in PMs may reflect either a classical [M1] (*CXCL1*,* CXCL3*,* IL1A*,* IL1B*,* IL8*,* NFKB2*) or an alternative [M2] (*CCL22*,* IL4R*,* IL10*) phenotype. Although a more activated state in PMs might be supported by the greater expression of the acute phase protein PTX3 and several interferons (*IFNA1*,* IFNA2*), certain PM transcripts (*IFNA2*,* IL6*) are more supportive of a novel state of hybrid polarisation recently reported in murine LPS‐tolerant macrophages 25. Both of these features may partly explain the previously reported nonresponsiveness of horse PMs to an LPS stimulus 4. Additionally, the PM expression of several neutrophil chemoattractants is consistent with neutrophil presence within the peritoneal fluid of healthy horses. It is possible that both the presence of neutrophils within, and the LPS tolerance of macrophages derived from, the peritoneal cavity of horses may reflect low‐grade endotoxin translocation across the gastrointestinal tract. Furthermore, the PM expression of certain hypoxia‐induced genes (*VEGFA*,* FLT1*,* CXCR7*,* HIF3A*) may reflect the hypoxic environment of the peritoneal cavity 26, 27.

The sizes of samples of AMs and PMs were relatively small and samples came from different horses, which is a limitation of this study. However, the use of BioLayout Express^3D^ allowed us to identify and exclude any genes that were up‐ or downregulated in a single sample (single animal) and hence the genes in our analysis are those that were consistently differentially regulated. The fact that the changes seen are largely corroborated by studies in other species indicates that this analysis is robust despite the small sample size.

Although the LPS response of equine AMs has been subject to previous study by others, largely justified by the hypothesised role of inhaled endotoxin in certain equine environmental airway diseases 5, 28, the data presented here are the most comprehensive to date. LPS‐induced upregulation of many of the genes identified has previously been reported in a variety of species, including the horse 28, 29. In comparison with equine monocytes, in which LPS seems to activate mainly targets of the MyD88 pathway 30, our data revealed LPS‐induced activation of both MyD88 (*TNF*,* IL1*,* IL6*,* IL10*) and TRIF (*IFNB*,* CCL5*) pathways in equine AMs, a finding which may reflect a degree of adaptation to the unique airway microenvironment. Similarly, the detection of anti‐inflammatory genes and genes related to both apoptosis and cytoprotection (*IL1RN*,* SOCS3*) 31 may reflect a role for AMs in minimising collateral tissue damage in a delicate tissue environment adapted for efficient gas exchange yet variably exposed to proinflammatory agents on a breath‐by‐breath basis. The greater LPS responsiveness and phagocytic capacity of AMs compared with PMs and the negligible neutrophil presence within this compartment probably reflect their adaptation to this key role. Furthermore, the transcriptomic data generated in this study provide a valuable reference dataset against which the biological effects of various novel therapeutic and/or prophylactic interventions aimed at up‐ or downregulating the innate immune response might be measured.

Comparison of our LPS‐induced gene expression data with those derived from studies in human AMs revealed many similarities 6, 12. A total of 66 orthologue genes amongst the differentially expressed genes were identified, 63 of which followed similar patterns of expression across species. As well as including genes involved with the stereotypical immune response to bacteria, this list included genes that were induced in man but not in mice. It would therefore appear that a dissimilarity in LPS‐induced macrophage gene expression also exists between the horse and the mouse, similar to that previously reported following man and mouse, and pig and mouse macrophage comparisons 6, 15. The failure to detect the LPS‐induced expression of genes involved in the NO pathway is consistent both with our previous inability to detect LPS‐induced NO metabolism in equine AMs 4 and with gene expression data derived from both human and porcine studies 15, 32. By contrast with murine macrophages, man‐ and pig‐derived macrophages in vitro metabolise tryptophan via IDO (indoleamine‐pyrrole 2,3‐dioxygenase) rather than producing NO via arginine metabolism in response to LPS 6, 15. Similarly, and in agreement with previous reports derived from equine AMs 4, 33, our data also provide evidence of tryptophan metabolism with the subsequent upregulation of IDO in the horse, thus further supporting a common pathway in this species and in both the pig and man. In light of the previously reported fundamental differences in human and murine cellular biology and the associated requirement for the development of novel animal models 15, 34, the similarities between man and horse macrophage gene expression revealed by our study justify further evaluation of the horse as an appropriate model for human subject inflammatory research and offer more assurance of the appropriateness of extrapolating from human subject‐derived data in this field of study.

## Conclusions

In combination, the findings described in this study have significantly increased our knowledge of equine macrophage biology, particularly that of AMs. Tissue‐dependent heterogeneity of macrophage function and phenotype was demonstrated and represents an appropriate platform of knowledge on which future studies can be based. The interspecies comparative data provide support for the potential role of the horse as a model for studies on human macrophage biology and completion of the equine genome annotation would provide a major tool for future transcriptomic horse studies, the findings of which may also be applicable in man.

## Authors’ declaration of interests

No competing interests have been declared.

## Ethical animal research

The Veterinary Ethical Review Committee of the University of Edinburgh approved the study protocols. The study was performed in material collected during post‐mortem examinations with the informed consent of the horses’ owners.

## Sources of funding

This work and the contributions of the authors were supported by the Royal (Dick) School of Veterinary Studies, and Zoetis pharmaceutical company (grant code G31244/33600). R. Kapetanovic is supported by an Australian Research Council Discovery Early Career Researcher Award Fellowship (DE1310470). The Roslin Institute (K. M. Summers and D. A. Hume) is supported by Strategic Programme Grants from the Biotechnology and Biological Sciences Research Council of the UK (grant code BB/J004235/1 and BB/J004227/1).

## Authorship

A. E. Karagianni contributed to the design of the experiments, performed all research experiments and analyses, and wrote the manuscript. R. Kapetanovic contributed to the experimental design, supervised the experiments and edited the manuscript. K. M. Summers supervised the microarray analysis and edited the manuscript. B. C. McGorum sourced research funding and proofread the final manuscript. R. S. Pirie and D. A. Hume supervised all research, contributed to the design of the experiments, sourced research funding and edited the manuscript.

## Supporting information


**Supplementary Item 1:** (a) Genes differentially expressed (DE) (alveolar macrophages [AMs] vs. peritoneal macrophages [PMs]); (b) genes more expressed in AMs; (c) genes more expressed in PMs, and (d) genes DE (AM vs. PM extensive).

**Supplementary Item 2:** Genelist of lipopolysaccharide‐treated alveolar macrophages.

## Manufacturers' addresses

1 Dechra Veterinary Products, West Pavilion/Sansaw Business Park, Hadnall, Shrewsbury UK.

2 Invitrogen Ltd, Paisley, Renfrewshire, UK.

3 Sigma‐Aldrich Co. Ltd, Gillingham, Dorset, UK.

4 AMS Biotechnology Europe Ltd (AMSBIO), Abingdon, Oxfordshire, UK.

5 Thermo Fihser Scientific, Wilmington, North Carolina, USA.

6 Agilent Technologies Deutschland GmbH & Co. KG, Waldbronn, Germany.

7 Affymetrix, Inc., Santa Clara, California, USA.

8 North America Partek, Inc., St Louis, Missouri, USA.
