# Supplementary material for: Comparative transcriptome analysis of equine alveolar macrophages
Source: Equine Vet J. 2016 Jul 9;49(3):375–82. doi: 10.1111/evj.12584 (PMC5412682; doi:10.1111/evj.12584)
Supplement: Supplementary file 1 — Supplementary Item 1: (a) Genes differentially expressed (DE) (alveolar macrophages [AMs] vs. peritoneal macrophages [PMs]); (b) genes more expressed in AMs; (c) genes more expressed in PMs, and (d) genes DE (AM vs. PM extensive). [file EVJ-49-375-s001.pdf]

Equine Veterinary Journal

Supplementary Item 1a: Genelist of 451 transcripts differentially expressed between AMs and PMs - with p adjusted value <0.01 and fold cha**nge >9**

Transcript ID	Gene_assignment	Gene name	Gene symbol	p Value	Fold change	Description
15121321	ENSECAT00000013628 // LOC100052888 // cholesterol 7-alpha-monooxygenase-like // --- //	cholesterol 7-alpha-monooxygenase-like	LOC100052888	<0,01	49.5489	AM up vs PM
15080122	XM_001487907 // LOC100049847 // platelet glycoprotein 4-like // --- // 100049847 /// EN	platelet glycoprotein 4-like	LOC100049847	<0,01	38.3266	AM up vs PM
15066795	XM_001916447 // LOC100057831 // n-acylethanolamine-hydrolyzing acid amidase-like // ---	n-acylethanolamine-hydrolyzing acid amidase-lik	LOC100057831	<0,01	25.7024	AM up vs PM
15088442	ENSECAT00000011655 // MCOLN2 // mucolipin 2 // --- // 100052719 /// XM_001496833 // MCO	mucolipin 2	MCOLN2	<0,01	21.8081	AM up vs PM
15126979	NM_001111301 // TLR8 // toll-like receptor 8 // --- // 100054367 /// ENSECAT00000009327	toll-like receptor 8	TLR8	<0,01	20.9228	AM up vs PM
14966871	---			<0,01	17.1072	AM up vs PM
15134514	---			<0,01	16.2329	AM up vs PM
15051976	ENSECAT00000005057 // PTGR1 // prostaglandin reductase 1 // --- // ---	prostaglandin reductase 1	PTGR1	<0,01	14.2633	AM up vs PM
15023455	XM_001501495 // LOC100071667 // complement C1q subcomponent subunit B-like // --- // 10	complement C1q subcomponent subunit B-like	LOC100071667	<0,01	14.0384	AM up vs PM
15100625	ENSECAT00000015675 // PLBD1 // phospholipase B domain containing 1 // --- // 100066968	phospholipase B domain containing 1	PLBD1	<0,01	11.7085	AM up vs PM
14943095	ENSECAT00000011152 // EGR2 // early growth response 2 // --- // 100062890 /// XM_001503	early growth response 2	EGR2	<0,01	10.7066	AM up vs PM
15023462	XM_001504261 // LOC100058097 // complement C1q subcomponent subunit A-like // --- // 10	complement C1q subcomponent subunit A-like	LOC100058097	<0,01	10.5162	AM up vs PM
14969419	XM_001914718 // LOC100060647 // catalase-like // --- // 100060647 /// ENSECAT0000002124	catalase-like	LOC100060647	<0,01	9.51748	AM up vs PM
15023458	XM_001504258 // LOC100058056 // complement C1q subcomponent subunit C-like // --- // 10	complement C1q subcomponent subunit C-like	LOC100058056	<0,01	9.32528	AM up vs PM
14939930	---			<0,01	-9.0112	AM down vs PM
14938040	---			<0,01	-9.09147	AM down vs PM
15131203	---			<0,01	-9.10315	AM down vs PM
14950082	---			<0,01	-9.12671	AM down vs PM
15087535	---			<0,01	-9.15312	AM down vs PM
15033411	---			<0,01	-9.18649	AM down vs PM
15060455	---			<0,01	-9.19773	AM down vs PM
15136936	---			<0,01	-9.25399	AM down vs PM
14934920	---			<0,01	-9.27641	AM down vs PM
14938232	---			<0,01	-9.31958	AM down vs PM
15126812	---			<0,01	-9.3368	AM down vs PM
15082470	---			<0,01	-9.33748	AM down vs PM
14989338	---			<0,01	-9.33968	AM down vs PM
15106374	---			<0,01	-9.42303	AM down vs PM
15001110	---			<0,01	-9.44207	AM down vs PM
14942828	---			<0,01	-9.44707	AM down vs PM
15006694	---			<0,01	-9.4937	AM down vs PM
15080985	---			<0,01	-9.52693	AM down vs PM
15071325	---			<0,01	-9.52777	AM down vs PM
15103641	NR_032873 // MIR7-2 // microRNA mir-7-2 // --- // 100315008	microRNA mir-7-2	MIR7-2	<0,01	-9.67189	AM down vs PM
15100530	---			<0,01	-9.69646	AM down vs PM
15123607	---			<0,01	-9.69954	AM down vs PM
15018734	---			<0,01	-9.70939	AM down vs PM
15120364	ENSECAT00000018691 // TCF7L2 // transcription factor 7-like 2 (T-cell specific, HMG-box	transcription factor 7-like 2	TCF7L2	<0,01	-9.74285	AM down vs PM
14928021	---			<0,01	-9.77627	AM down vs PM
15034555	---			<0,01	-9.82738	AM down vs PM
15082266	---			<0,01	-9.83919	AM down vs PM
15009515	---			<0,01	-9.85658	AM down vs PM
15116897	---			<0,01	-9.85674	AM down vs PM
14972822	---			<0,01	-9.86893	AM down vs PM
15136724	---			<0,01	-9.87303	AM down vs PM
15137306	---			<0,01	-9.89187	AM down vs PM
15123696	---			<0,01	-9.94105	AM down vs PM
15056884	---			<0,01	-9.94743	AM down vs PM
15076475	---			<0,01	-9.96444	AM down vs PM
15131878	---			<0,01	-9.97001	AM down vs PM
14929483	---			<0,01	-9.97779	AM down vs PM
14990746	---			<0,01	-9.98512	AM down vs PM
15035443	---			<0,01	-10.0111	AM down vs PM
15091422	---			<0,01	-10.0137	AM down vs PM

15136932	---			<0,01	-10.0562	AM down vs PM
15021574	---			<0,01	-10.125	AM down vs PM
15119937	---			<0,01	-10.1436	AM down vs PM
15131829	---			<0,01	-10.1701	AM down vs PM
15136330	---			<0,01	-10.1879	AM down vs PM
15136396	---			<0,01	-10.1879	AM down vs PM
14925107	---			<0,01	-10.2665	AM down vs PM
15133248	---			<0,01	-10.2836	AM down vs PM
15133576	---			<0,01	-10.3176	AM down vs PM
15094905	NM_001257078 // CXCR7 // chemokine (C-X-C motif) receptor 7 // --- // 100057501 /// ENS	chemokine (C-X-C motif) receptor 7	CXCR7	<0,01	-10.324	AM down vs PM
14986670	---			<0,01	-10.3378	AM down vs PM
15013214	---			<0,01	-10.3447	AM down vs PM
14938015	---			<0,01	-10.365	AM down vs PM
15016292	---			<0,01	-10.3722	AM down vs PM
14969935	---			<0,01	-10.43	AM down vs PM
15136988	---			<0,01	-10.4448	AM down vs PM
14927731	---			<0,01	-10.4651	AM down vs PM
14942998	---			<0,01	-10.4891	AM down vs PM
15045391	---			<0,01	-10.5134	AM down vs PM
14937067	---			<0,01	-10.5778	AM down vs PM
15137284	---			<0,01	-10.614	AM down vs PM
15006532	---			<0,01	-10.9163	AM down vs PM
15071344	---			<0,01	-10.9631	AM down vs PM
15020143	---			<0,01	-10.9861	AM down vs PM
15003586	---			<0,01	-11.0115	AM down vs PM
15030212	XM_001505026 // LOC100052624 // histone H2A type 1-like // --- // 100052624 /// ENSECAT	histone H2A type 1-like	LOC100052624	<0,01	-11.0312	AM down vs PM
15029956	---			<0,01	-11.0774	AM down vs PM
14934714	---			<0,01	-11.1017	AM down vs PM
15011590	---			<0,01	-11.1183	AM down vs PM
15136368	---			<0,01	-11.1494	AM down vs PM
14981433	---			<0,01	-11.2217	AM down vs PM
15057108	---			<0,01	-11.2319	AM down vs PM
14982053	---			<0,01	-11.2916	AM down vs PM
15015661	---			<0,01	-11.3752	AM down vs PM
15030285	XM_003363755 // LOC100629501 // olfactory receptor 2W1-like // --- // 100629501 /// ENS	olfactory receptor 2W1-like	LOC100629501	<0,01	-11.4432	AM down vs PM
15076479	XM_001489227 // SEMA3C // sema domain, immunoglobulin domain (Ig), short basic domain,	sema domain, immunoglobulin domain (Ig)	SEMA3C	<0,01	-11.5196	AM down vs PM
15128302	---			<0,01	-11.5748	AM down vs PM
14986175	---			<0,01	-11.63	AM down vs PM
15120682	---			<0,01	-11.7021	AM down vs PM
14934934	---			<0,01	-11.7342	AM down vs PM
14945248	---			<0,01	-11.7834	AM down vs PM
15120725	---			<0,01	-11.8124	AM down vs PM
15077680	---			<0,01	-11.8639	AM down vs PM
15106867	---			<0,01	-11.883	AM down vs PM
15136798	---			<0,01	-11.923	AM down vs PM
15129744	---			<0,01	-11.9658	AM down vs PM
15081996	---			<0,01	-12.0043	AM down vs PM
14987283	---			<0,01	-12.0545	AM down vs PM
15136978	---			<0,01	-12.1102	AM down vs PM
14943919	---			<0,01	-12.2044	AM down vs PM
14962196	---			<0,01	-12.3211	AM down vs PM
15136494	---			<0,01	-12.3273	AM down vs PM
15081440	---			<0,01	-12.3633	AM down vs PM
15137740	---			<0,01	-12.4339	AM down vs PM
14979733	---			<0,01	-12.5955	AM down vs PM
15107853	---			<0,01	-12.5985	AM down vs PM
15070812	---			<0,01	-12.634	AM down vs PM

15031964	---			<0,01	-12.6654	AM down vs PM
15029693	---			<0,01	-12.67	AM down vs PM
15136558	---			<0,01	-12.67	AM down vs PM
15101493	---			<0,01	-12.7499	AM down vs PM
15136774	---			<0,01	-12.7733	AM down vs PM
15137338	---			<0,01	-12.7785	AM down vs PM
14928003	---			<0,01	-12.8005	AM down vs PM
14934917	---			<0,01	-12.8454	AM down vs PM
15006335	---			<0,01	-12.8522	AM down vs PM
15136364	---			<0,01	-12.8522	AM down vs PM
15063181	---			<0,01	-12.865	AM down vs PM
15053644	XR_036256 // LOC100068798 // calponin-3-like // --- // 100068798 /// XM_001490827 // LO	calponin-3-like	LOC100068798	<0,01	-12.8677	AM down vs PM
15006938	---			<0,01	-12.8778	AM down vs PM
15033037	---			<0,01	-12.9188	AM down vs PM
14949316	---			<0,01	-12.9467	AM down vs PM
15003625	---			<0,01	-13.0727	AM down vs PM
15045573	---			<0,01	-13.1091	AM down vs PM
15057372	---			<0,01	-13.1106	AM down vs PM
15129919	---			<0,01	-13.1292	AM down vs PM
15060545	---			<0,01	-13.2365	AM down vs PM
14937073	---			<0,01	-13.2641	AM down vs PM
15019934	---			<0,01	-13.3434	AM down vs PM
15008985	---			<0,01	-13.4618	AM down vs PM
15128327	---			<0,01	-13.4813	AM down vs PM
15030305	---			<0,01	-13.5114	AM down vs PM
15128934	---			<0,01	-13.5188	AM down vs PM
14929431	---			<0,01	-13.5294	AM down vs PM
15001469	---			<0,01	-13.6178	AM down vs PM
14948030	---			<0,01	-13.6582	AM down vs PM
15137280	---			<0,01	-13.6852	AM down vs PM
15054737	---			<0,01	-13.7121	AM down vs PM
15044653	---			<0,01	-13.7904	AM down vs PM
15079137	---			<0,01	-13.915	AM down vs PM
15005672	---			<0,01	-13.9337	AM down vs PM
15060218	NM_001081780 // LUM // lumican // --- // 100009681 /// ENSECAT00000019349 // LUM // lum	lumican	LUM	<0,01	-13.935	AM down vs PM
15110227	---			<0,01	-14.0682	AM down vs PM
15121666	---			<0,01	-14.2476	AM down vs PM
15137750	---			<0,01	-14.4959	AM down vs PM
14939195	---			<0,01	-14.5698	AM down vs PM
14945347	---			<0,01	-14.7454	AM down vs PM
15121695	---			<0,01	-14.8225	AM down vs PM
15128857	---			<0,01	-14.966	AM down vs PM
15136466	---			<0,01	-14.9687	AM down vs PM
15132461	---			<0,01	-15.1515	AM down vs PM
15077411	---			<0,01	-15.1611	AM down vs PM
15062898	---			<0,01	-15.2624	AM down vs PM
15137382	---			<0,01	-15.2918	AM down vs PM
15137596	---			<0,01	-15.2918	AM down vs PM
15128427	---			<0,01	-15.3044	AM down vs PM
15062932	---			<0,01	-15.4685	AM down vs PM
15006034	---			<0,01	-15.6644	AM down vs PM
15054877	---			<0,01	-15.7366	AM down vs PM
14953056	---			<0,01	-15.7403	AM down vs PM
15029587	---			<0,01	-15.771	AM down vs PM
14956069	---			<0,01	-15.8166	AM down vs PM
14998899	---			<0,01	-15.8931	AM down vs PM
15133022	---			<0,01	-15.9749	AM down vs PM

15068686	---			<0,01	-16.0166 AM down vs PM
15003583	---			<0,01	-16.0171 AM down vs PM
15132692	---			<0,01	-16.0431 AM down vs PM
14957420	---			<0,01	-16.0549 AM down vs PM
15088030	---			<0,01	-16.318 AM down vs PM
14981953	ENSECAT00000019198 // ADAM19 // ADAM metallopeptidase domain 19 // --- // ---	ADAM metallopeptidase domain 19	ADAM19	<0,01	-16.3272 AM down vs PM
15078648	---			<0,01	-16.6413 AM down vs PM
15021779	---			<0,01	-16.771 AM down vs PM
15132014	---			<0,01	-16.9077 AM down vs PM
14987063	---			<0,01	-17.0099 AM down vs PM
15132754	---			<0,01	-17.0311 AM down vs PM
15136740	---			<0,01	-17.0521 AM down vs PM
15133394	---			<0,01	-17.0556 AM down vs PM
14972192	---			<0,01	-17.1074 AM down vs PM
15131131	---			<0,01	-17.1132 AM down vs PM
15137618	---			<0,01	-17.16 AM down vs PM
14957006	---			<0,01	-17.1707 AM down vs PM
15063592	---			<0,01	-17.1957 AM down vs PM
15020762	---			<0,01	-17.2581 AM down vs PM
14970532	---			<0,01	-17.2951 AM down vs PM
15136360	---			<0,01	-17.3386 AM down vs PM
15137394	---			<0,01	-17.4174 AM down vs PM
15136446	---			<0,01	-17.4933 AM down vs PM
15136374	---			<0,01	-17.5034 AM down vs PM
15136754	---			<0,01	-17.5074 AM down vs PM
15127535	---			<0,01	-17.5681 AM down vs PM
15082473	---			<0,01	-17.6968 AM down vs PM
15128311	---			<0,01	-17.7146 AM down vs PM
15117833	---			<0,01	-17.727 AM down vs PM
15018759	---			<0,01	-17.8395 AM down vs PM
14956620	---			<0,01	-17.8451 AM down vs PM
14972820	XM_001497247 // LOC100067132 // olfactory receptor 476-like // --- // 100067132 /// ENS	olfactory receptor 476-like	LOC100067132	<0,01	-17.8474 AM down vs PM
15032277	---			<0,01	-17.8782 AM down vs PM
14925807	---			<0,01	-17.9532 AM down vs PM
14925837	---			<0,01	-17.9532 AM down vs PM
14925879	---			<0,01	-17.9532 AM down vs PM
14925951	---			<0,01	-17.9532 AM down vs PM
14978864	---			<0,01	-18.0059 AM down vs PM
15137146	---			<0,01	-18.007 AM down vs PM
15082809	---			<0,01	-18.1827 AM down vs PM
15132482	---			<0,01	-18.2294 AM down vs PM
15020350	---			<0,01	-18.2685 AM down vs PM
15117290	---			<0,01	-18.4039 AM down vs PM
15136286	---			<0,01	-18.4182 AM down vs PM
15129022	---			<0,01	-18.5343 AM down vs PM
14947605	---			<0,01	-18.5416 AM down vs PM
15136472	---			<0,01	-18.5454 AM down vs PM
14995193	---			<0,01	-18.5635 AM down vs PM
15078277	---			<0,01	-18.5838 AM down vs PM
15029101	---			<0,01	-18.6205 AM down vs PM
14936458	---			<0,01	-18.6637 AM down vs PM
15045035	---			<0,01	-18.677 AM down vs PM
15124574	---			<0,01	-18.7169 AM down vs PM
14986730	---			<0,01	-18.8457 AM down vs PM
15042777	---			<0,01	-18.9303 AM down vs PM
15130707	---			<0,01	-19.2377 AM down vs PM
15136302	---			<0,01	-19.2564 AM down vs PM

15067799	---			<0,01	-19.4071	AM down vs PM
15136210	---			<0,01	-19.4734	AM down vs PM
15051455	---			<0,01	-19.4757	AM down vs PM
14943132	---			<0,01	-19.567	AM down vs PM
15053642	---			<0,01	-19.9222	AM down vs PM
15130705	---			<0,01	-19.9706	AM down vs PM
14992455	---			<0,01	-19.9856	AM down vs PM
15136206	---			<0,01	-19.9856	AM down vs PM
15088797	---			<0,01	-20.068	AM down vs PM
15137694	---			<0,01	-20.078	AM down vs PM
14945595	---			<0,01	-20.1176	AM down vs PM
15081326	---			<0,01	-20.253	AM down vs PM
15070665	---			<0,01	-20.3069	AM down vs PM
15077744	---			<0,01	-20.5505	AM down vs PM
14981259	---			<0,01	-20.5628	AM down vs PM
15136398	---			<0,01	-20.5731	AM down vs PM
15137488	---			<0,01	-20.6121	AM down vs PM
15136990	---			<0,01	-20.6359	AM down vs PM
15137038	---			<0,01	-20.7475	AM down vs PM
15013715	---			<0,01	-20.9782	AM down vs PM
15095850	---			<0,01	-21.0675	AM down vs PM
15130280	---			<0,01	-21.4392	AM down vs PM
15137376	---			<0,01	-21.4701	AM down vs PM
15009301	---			<0,01	-21.5584	AM down vs PM
15132186	---			<0,01	-21.6506	AM down vs PM
15081319	---			<0,01	-21.6663	AM down vs PM
15073950	---			<0,01	-21.7265	AM down vs PM
15006991	---			<0,01	-21.8189	AM down vs PM
15081224	---			<0,01	-21.873	AM down vs PM
14994624	---			<0,01	-21.8924	AM down vs PM
15136260	---			<0,01	-22.0647	AM down vs PM
15136808	---			<0,01	-22.0968	AM down vs PM
14997932	---			<0,01	-22.1161	AM down vs PM
14951445	---			<0,01	-22.1351	AM down vs PM
14956174	---			<0,01	-22.2277	AM down vs PM
15121381	---			<0,01	-22.2542	AM down vs PM
15131450	---			<0,01	-22.2625	AM down vs PM
15137370	---			<0,01	-22.3584	AM down vs PM
15104019	---			<0,01	-22.4938	AM down vs PM
15133141	---			<0,01	-22.5699	AM down vs PM
15137422	---			<0,01	-22.5763	AM down vs PM
15067039	---			<0,01	-22.5821	AM down vs PM
14952507	---			<0,01	-22.6912	AM down vs PM
15047659	---			<0,01	-22.7562	AM down vs PM
15104015	---			<0,01	-22.7969	AM down vs PM
15136580	---			<0,01	-23.1692	AM down vs PM
15013552	---			<0,01	-23.2454	AM down vs PM
14978556	---			<0,01	-23.3349	AM down vs PM
15136930	---			<0,01	-23.3383	AM down vs PM
15091433	---			<0,01	-23.5413	AM down vs PM
14975354	AF508034 // PAI-1 // plasminogen activator inhibitor-1 // --- // 100033931	plasminogen activator inhibitor-1	PAI-1	<0,01	-23.8273	AM down vs PM
15128853	---			<0,01	-24.1031	AM down vs PM
15052447	---			<0,01	-24.1896	AM down vs PM
15035006	---			<0,01	-24.1938	AM down vs PM
15002933	---			<0,01	-24.2487	AM down vs PM
15003532	---			<0,01	-24.3102	AM down vs PM
15025136	---			<0,01	-24.7365	AM down vs PM

15137522	---			<0,01	-24.8593	AM down vs PM
15066331	---			<0,01	-25.1991	AM down vs PM
14927037	---			<0,01	-25.2551	AM down vs PM
15000483	---			<0,01	-25.2714	AM down vs PM
15011434	ENSECAT00000004554 // PTX3 // pentraxin 3, long // --- // ---	pentraxin 3	PTX3	<0,01	-25.3722	AM down vs PM
15067000	---			<0,01	-25.8976	AM down vs PM
15097038	---			<0,01	-25.9746	AM down vs PM
15137450	---			<0,01	-26.0121	AM down vs PM
15075331	---			<0,01	-26.0917	AM down vs PM
15024544	---			<0,01	-26.1531	AM down vs PM
14943439	---			<0,01	-26.813	AM down vs PM
15136662	---			<0,01	-26.9099	AM down vs PM
15003542	---			<0,01	-26.9591	AM down vs PM
15137700	---			<0,01	-26.9642	AM down vs PM
15047220	---			<0,01	-27.28	AM down vs PM
15136458	---			<0,01	-27.4124	AM down vs PM
15115746	---			<0,01	-27.4148	AM down vs PM
15022133	---			<0,01	-27.6343	AM down vs PM
15019959	---			<0,01	-27.7861	AM down vs PM
15136532	---			<0,01	-27.9585	AM down vs PM
15001446	---			<0,01	-28.1817	AM down vs PM
15136292	---			<0,01	-28.1817	AM down vs PM
15136620	---			<0,01	-28.2968	AM down vs PM
15136790	---			<0,01	-28.3046	AM down vs PM
15131847	---			<0,01	-28.5569	AM down vs PM
14949750	---			<0,01	-28.8852	AM down vs PM
15085237	---			<0,01	-28.9337	AM down vs PM
15137490	---			<0,01	-29.0469	AM down vs PM
15136244	---			<0,01	-29.1084	AM down vs PM
14935042				<0,01	-29.2099	AM down vs PM
15136584	---			<0,01	-29.3036	AM down vs PM
14939913	XM_003363631 // LOC100629686 // ig kappa chain V-III region PC 2485/PC 4039-like // ---	ig kappa chain V-III region PC 2485/PC 4039-like	LOC100629686	<0,01	-29.312	AM down vs PM
15025367	---			<0,01	-29.3716	AM down vs PM
15026893	---			<0,01	-29.4833	AM down vs PM
15081328	---			<0,01	-29.8041	AM down vs PM
15060263	---			<0,01	-29.8346	AM down vs PM
15137214	---			<0,01	-29.9268	AM down vs PM
15096136	---			<0,01	-29.931	AM down vs PM
15004469	---			<0,01	-29.9682	AM down vs PM
14938460	---			<0,01	-29.9793	AM down vs PM
15013653	---			<0,01	-30.0631	AM down vs PM
15137684	---			<0,01	-30.1908	AM down vs PM
14970624	---			<0,01	-30.2479	AM down vs PM
14943758	---			<0,01	-30.3293	AM down vs PM
15077222	---			<0,01	-30.5649	AM down vs PM
15136770	---			<0,01	-30.8029	AM down vs PM
15067793	---			<0,01	-31.2409	AM down vs PM
14949314	---			<0,01	-31.3397	AM down vs PM
15136442	---			<0,01	-31.7406	AM down vs PM
14943621	---			<0,01	-32.1469	AM down vs PM
15128313	---			<0,01	-32.2533	AM down vs PM
14927251	---			<0,01	-32.2825	AM down vs PM
15008001	---			<0,01	-32.2965	AM down vs PM
14942831	---			<0,01	-32.4793	AM down vs PM
15136884	---			<0,01	-32.5006	AM down vs PM
14939826	---			<0,01	-32.5426	AM down vs PM
15136786	---			<0,01	-32.7815	AM down vs PM

15136478	---	<0,01	-33.1294	AM down vs PM
15137138	---	<0,01	-33.5849	AM down vs PM
15026633	---	<0,01	-33.6268	AM down vs PM
15033547	---	<0,01	-33.6459	AM down vs PM
15115930	---	<0,01	-33.8559	AM down vs PM
15047176	---	<0,01	-34.2521	AM down vs PM
14986542	---	<0,01	-34.5596	AM down vs PM
15123572	---	<0,01	-34.7115	AM down vs PM
15031962	---	<0,01	-34.8324	AM down vs PM
15013612	---	<0,01	-34.8776	AM down vs PM
15128937	---	<0,01	-35.153	AM down vs PM
15137318	---	<0,01	-35.153	AM down vs PM
15110451	---	<0,01	-35.2867	AM down vs PM
15080685	---	<0,01	-35.702	AM down vs PM
14952251	---	<0,01	-35.9024	AM down vs PM
15137586	---	<0,01	-35.9024	AM down vs PM
15137582	---	<0,01	-36.0792	AM down vs PM
14996189	---	<0,01	-36.1282	AM down vs PM
15137460	---	<0,01	-36.4128	AM down vs PM
15132102	---	<0,01	-36.6078	AM down vs PM
15136926	---	<0,01	-37.4779	AM down vs PM
15128840	---	<0,01	-37.5234	AM down vs PM
15136666	---	<0,01	-37.6173	AM down vs PM
14935334	---	<0,01	-37.6561	AM down vs PM
15136698	---	<0,01	-37.8108	AM down vs PM
15137182	---	<0,01	-37.991	AM down vs PM
15097439	---	<0,01	-38.1454	AM down vs PM
15128145	---	<0,01	-38.3314	AM down vs PM
14969512	---	<0,01	-38.3468	AM down vs PM
15136230	---	<0,01	-38.4083	AM down vs PM
14937086	---	<0,01	-38.7106	AM down vs PM
15021111	---	<0,01	-38.751	AM down vs PM
15053588	---	<0,01	-38.7772	AM down vs PM
15007548	---	<0,01	-38.8115	AM down vs PM
14944218	---	<0,01	-39.0484	AM down vs PM
14936804	---	<0,01	-39.2676	AM down vs PM
15136530	---	<0,01	-40.011	AM down vs PM
15002538	---	<0,01	-40.1598	AM down vs PM
15026631	---	<0,01	-40.6787	AM down vs PM
14978691	---	<0,01	-40.6799	AM down vs PM
15136238	---	<0,01	-41.0053	AM down vs PM
14995011	---	<0,01	-41.0053	AM down vs PM
15137326	---	<0,01	-42.4186	AM down vs PM
15126570		<0,01	-42.6344	AM down vs PM
15137436	---	<0,01	-43.1264	AM down vs PM
15137530	---	<0,01	-43.429	AM down vs PM
15129820	---	<0,01	-43.4952	AM down vs PM
15136308	---	<0,01	-44.3743	AM down vs PM
15081696	---	<0,01	-44.4924	AM down vs PM
15137562	---	<0,01	-45.255	AM down vs PM
15128737	---	<0,01	-45.3077	AM down vs PM
15137556	---	<0,01	-45.6471	AM down vs PM
14948800	---	<0,01	-46.3879	AM down vs PM
15136922	---	<0,01	-47.074	AM down vs PM
15137244	---	<0,01	-47.511	AM down vs PM
14997839	---	<0,01	-47.6731	AM down vs PM
14996894	---	<0,01	-48.2224	AM down vs PM

15123626	---	<0,01	-48.8002	AM down vs PM
15136502	---	<0,01	-49.0912	AM down vs PM
15042340	---	<0,01	-49.9077	AM down vs PM
15128949	---	<0,01	-50.085	AM down vs PM
15132227	---	<0,01	-50.2184	AM down vs PM
15137396	---	<0,01	-50.2184	AM down vs PM
15088688	---	<0,01	-50.5678	AM down vs PM
14943127	---	<0,01	-51.5054	AM down vs PM
15004330	---	<0,01	-51.7294	AM down vs PM
15122064	---	<0,01	-51.8703	AM down vs PM
15137288	---	<0,01	-52.1903	AM down vs PM
15137524	---	<0,01	-52.5658	AM down vs PM
15137386	---	<0,01	-53.6274	AM down vs PM
15025326	---	<0,01	-56.308	AM down vs PM
15042941	---	<0,01	-56.308	AM down vs PM
14992314	---	<0,01	-57.2961	AM down vs PM
15056875	---	<0,01	-57.3263	AM down vs PM
15128806	---	<0,01	-57.683	AM down vs PM
15137304	---	<0,01	-57.683	AM down vs PM
15137420	---	<0,01	-57.893	AM down vs PM
15065033	---	<0,01	-58.9359	AM down vs PM
15137316	---	<0,01	-59.0041	AM down vs PM
15054769	---	<0,01	-59.3197	AM down vs PM
15040330	---	<0,01	-59.991	AM down vs PM
15136282	---	<0,01	-60.5216	AM down vs PM
14992104	---	<0,01	-60.7582	AM down vs PM
14926511	---	<0,01	-61.6782	AM down vs PM
15077655	---	<0,01	-62.1591	AM down vs PM
15136898	---	<0,01	-62.1591	AM down vs PM
15111236	---	<0,01	-62.2758	AM down vs PM
15136202	---	<0,01	-62.3235	AM down vs PM
15137282	---	<0,01	-62.6104	AM down vs PM
14956238	---	<0,01	-62.9583	AM down vs PM
15065994	---	<0,01	-64.2191	AM down vs PM
15137094	---	<0,01	-67.7203	AM down vs PM
14998362	---	<0,01	-68.0454	AM down vs PM
15136264	---	<0,01	-68.0454	AM down vs PM
15137612	---	<0,01	-68.869	AM down vs PM
15009517	---	<0,01	-70.1267	AM down vs PM
15136414	---	<0,01	-70.1267	AM down vs PM
14952023	---	<0,01	-70.8349	AM down vs PM
15137574	---	<0,01	-70.8349	AM down vs PM
15137466	---	<0,01	-72.524	AM down vs PM
15137516	---	<0,01	-77.4462	AM down vs PM
15132417	---	<0,01	-82.3122	AM down vs PM
15136682	---	<0,01	-89.1531	AM down vs PM
14941506	---	<0,01	-89.2852	AM down vs PM
15137340	---	<0,01	-91.5812	AM down vs PM
15025365	---	<0,01	-93.9848	AM down vs PM
15136288	---	<0,01	-98.4802	AM down vs PM
14941804	---	<0,01	-130.059	AM down vs PM
15136486	---	<0,01	-178.292	AM down vs PM
15136730	---	<0,01	-184.898	AM down vs PM
15136318	---	<0,01	-200.831	AM down vs PM
15136676	---	<0,01	-242.107	AM down vs PM
