# Supplementary material for: Comparative transcriptome analysis of equine alveolar macrophages
Source: Equine Vet J. 2016 Jul 9;49(3):375–82. doi: 10.1111/evj.12584 (PMC5412682; doi:10.1111/evj.12584)
Supplement: Supplementary file 2 [file EVJ-49-375-s002.pdf]

Equine Veterinary Journal

Supplementary Item 1b: Using BIOLAYOUT : Top clusters of genes more highly expressed in AMs

Transcript ID	Gene_assignment	Gene symbol	MCL_2.2
15030673	XM_001490934 // ABHD16A // abhydrolase domain containing 16A // --- // 100058736 /// EN	ABHD16A	Cluster0002
14994711	XM_001503658 // ACP1 // acid phosphatase 1, soluble // --- // 100057408 /// ENSECAT0000	ACP1	Cluster0002
15011734	XM_001496284 // ACTL6A // actin-like 6A // --- // 100065792 /// ENSECAT00000014242 // A	ACTL6A	Cluster0002
14988137	XM_001491463 // ACTR1B // ARP1 actin-related protein 1 homolog B, centractin beta (yeas	ACTR1B	Cluster0002
15009338	XM_001491549 // ACVR1 // activin A receptor, type I // --- // 100050899 /// ENSECAT0000	ACVR1	Cluster0002
14947073	ENSECAT00000015275 // ADAL // adenosine deaminase-like // --- // 100070813	ADAL	Cluster0002
15072643	ENSECAT00000014034 // ADD1 // adducin 1 (alpha) // --- // 100051451	ADD1	Cluster0002
15074824	XM_001496039 // ADIPOR1 // adiponectin receptor 1 // --- // 100052436 /// ENSECAT000000	ADIPOR1	Cluster0002
15099658	NM_001163830 // ADIPOR2 // adiponectin receptor 2 // --- // 100050051 /// ENSECAT000000	ADIPOR2	Cluster0002
15094456	ENSECAT00000015868 // AGFG1 // ArfGAP with FG repeats 1 // --- // ---	AGFG1	Cluster0002
14975160	ENSECAT00000017199 // AGFG2 // ArfGAP with FG repeats 2 // --- // 100068998	AGFG2	Cluster0002
15077776	XM_001496547 // AHR // aryl hydrocarbon receptor // --- // 100066186 /// ENSECAT0000001	AHR	Cluster0002
14971483	NM_001081937 // AIP // aryl hydrocarbon receptor interacting protein // --- // 10003414	AIP	Cluster0002
15018296	XM_001917282 // ALDH4A1 // aldehyde dehydrogenase 4 family, member A1 // --- // 1001472	ALDH4A1	Cluster0002
15051570	XM_001504059 // ALG2 // asparagine-linked glycosylation 2, alpha-1,3-mannosyltransferas	ALG2	Cluster0002
15014171	XM_001497526 // ALG3 // asparagine-linked glycosylation 3, alpha-1,3- mannosyltransfera	ALG3	Cluster0002
15106791	ENSECAT00000024510 // ALG8 // asparagine-linked glycosylation 8, alpha-1,3-glucosyltran	ALG8	Cluster0002
15002728	NM_001163965 // ALOX5AP // arachidonate 5-lipoxygenase-activating protein // --- // 100	ALOX5AP	Cluster0002
14972264	ENSECAT00000025776 // AMBRA1 // autophagy/beclin-1 regulator 1 // --- // 100050652 ///	AMBRA1	Cluster0002
14964870	ENSECAT00000011973 // AMZ2 // archaelysin family metallopeptidase 2 // --- // 100062918	AMZ2	Cluster0002
14934969	XM_001504023 // ANXA11 // annexin A11 // --- // 100064425 /// ENSECAT00000021182 // ANX	ANXA11	Cluster0002
14937776	NM_001123380 // ANXA2 // annexin A2 // --- // 100054320 /// ENSECAT00000013926 // ANXA2	ANXA2	Cluster0002
15043873	ENSECAT00000013423 // APTX // aprataxin // --- // 100068091 /// XM_001917719 // APTX //	APTX	Cluster0002
14972376	ENSECAT00000026736 // ARFGAP2 // ADP-ribosylation factor GTPase activating protein 2 //	ARFGAP2	Cluster0002
14972287	XM_001489971 // ARHGAP1 // Rho GTPase activating protein 1 // --- // 100050810 /// ENSE	ARHGAP1	Cluster0002
14959599	ENSECAT00000006684 // ARHGAP27 // Rho GTPase activating protein 27 // --- // --- /// EN	ARHGAP27	Cluster0002
15107874	NM_001081921 // ARNTL // aryl hydrocarbon receptor nuclear translocator-like // --- //	ARNTL	Cluster0002
15032771	ENSECAT00000017452 // ARRDC2 // arrestin domain containing 2 // --- // ---	ARRDC2	Cluster0002
14989694	NM_001205056 // ASB3 // ankyrin repeat and SOCS box containing 3 // --- // 100052747 //	ASB3	Cluster0002
15030874	ENSECAT00000023281 // ATF6B // activating transcription factor 6 beta // --- // 1000593	ATF6B	Cluster0002
14969721	NM_001242529 // ATG13 // autophagy related 13 // --- // 100050734	ATG13	Cluster0002
15129390	XM_001490668 // ATG4A // autophagy related 4A, cysteine peptidase // --- // 100060165 /	ATG4A	Cluster0002
15065478	ENSECAT00000012349 // ATMIN // ATM interactor // --- // 100069801 /// XM_001501932 // A	ATMIN	Cluster0002
15102827	NM_001195525 // ATP5B // ATP synthase, H+ transporting, mitochondrial F1 complex, beta	ATP5B	Cluster0002
14999509	ENSECAT00000026737 // ATXN7 // ataxin 7 // --- // 100055963 /// XM_001489903 // ATXN7 /	ATXN7	Cluster0002
15032628	ENSECAT00000018090 // BABAM1 // BRISC and BRCA1 A complex member 1 // --- // ---	BABAM1	Cluster0002
14941374	ENSECAT00000005416 // BAG3 // BCL2-associated athanogene 3 // --- // 100065784 /// XM_0	BAG3	Cluster0002
15030643	ENSECAT00000014870 // BAG6 // BCL2-associated athanogene 6 // --- // 100050296	BAG6	Cluster0002
14948644	ENSECAT00000008241 // BAZ1A // bromodomain adjacent to zinc finger domain, 1A // --- //	BAZ1A	Cluster0002
14950309	ENSECAT00000013003 // BCKDHA // branched chain keto acid dehydrogenase E1, alpha polype	BCKDHA	Cluster0002
14950528	ENSECAT00000013680 // BCL3 // B-cell CLL/lymphoma 3 // --- // ---	BCL3	Cluster0002
15014349	XM_001499782 // BCL6 // B-cell CLL/lymphoma 6 // --- // 100059642 /// ENSECAT0000000568	BCL6	Cluster0002
14980135	ENSECAT00000017875 // BFAR // bifunctional apoptosis regulator // --- // 100050577 ///	BFAR	Cluster0002
15125188	XM_001495762 // BOP1 // block of proliferation 1 // --- // 100065050 /// ENSECAT0000001	BOP1	Cluster0002
15017241	XM_001503784 // BSDC1 // BSD domain containing 1 // --- // 100055839 /// ENSECAT0000000	BSDC1	Cluster0002
15125853	NM_001098795 // BSG // basigin (Ok blood group) // --- // 100049616 /// ENSECAT00000012	BSG	Cluster0002
15126726	NM_001098795 // BSG // basigin (Ok blood group) // --- // 100049616 /// EF564280 // BSG	BSG	Cluster0002
14942141	ENSECAT00000024676 // BTRC // beta-transducin repeat containing E3 ubiquitin protein li	BTRC	Cluster0002
14941182	XM_001490046 // BUB3 // budding uninhibited by benzimidazoles 3 homolog (yeast) // ---	BUB3	Cluster0002
15126709	ENSECAT00000008528 // C19orf6 // chromosome 19 open reading frame 6 // --- // ---	C19orf6	Cluster0002
15033824	ENSECAT00000023863 // C1QTNF3 // C1q and tumor necrosis factor related protein 3 // ---	C1QTNF3	Cluster0002

15072073	ENSECAT00000025811 // C4orf52 // chromosome 4 open reading frame 52 // --- // ---	C4orf52	Cluster0002
14958248	XM_001490865 // CANT1 // calcium activated nucleotidase 1 // --- // 100057405 /// ENSEC	CANT1	Cluster0002
14988744	XM_001498985 // CAPG // capping protein (actin filament), gelsolin-like // --- // 10005	CAPG	Cluster0002
15004101	ENSECAT00000010445 // CARKD // carbohydrate kinase domain containing // --- // ---	CARKD	Cluster0002
15008128	ENSECAT00000005749 // CASP8 // caspase 8, apoptosis-related cysteine peptidase // --- /	CASP8	Cluster0002
15018507	ENSECAT00000018509 // CASP9 // caspase 9, apoptosis-related cysteine peptidase // --- /	CASP9	Cluster0002
15104949	ENSECAT00000005112 // CBL // Cbl proto-oncogene, E3 ubiquitin protein ligase // --- //	CBL	Cluster0002
15076754	XM_001491503 // CBLL1 // Cbl proto-oncogene, E3 ubiquitin protein ligase-like 1 // ---	CBLL1	Cluster0002
15043302	XM_001490027 // CBWD1 // COBW domain containing 1 // --- // 100050598 /// ENSECAT000000	CBWD1	Cluster0002
15120493	ENSECAT00000008399 // CCBE1 // collagen and calcium binding EGF domains 1 // --- // ---	CCBE1	Cluster0002
14934716	ENSECAT00000006365 // CCDC109A // mitochondrial calcium uniporter // --- // 100072824 /	CCDC109A	Cluster0002
14943017	XM_001503415 // CCDC6 // coiled-coil domain containing 6 // --- // 100062742 /// ENSECA	CCDC6	Cluster0002
15006497	XM_001915413 // CCNT2 // cyclin T2 // --- // 100050510 /// ENSECAT00000024357 // CCNT2	CCNT2	Cluster0002
15038286	NM_001081902 // CD40 // CD40 molecule, TNF receptor superfamily member 5 // --- // 1000	CD40	Cluster0002
15063552	XM_001499137 // CDC123 // cell division cycle 123 homolog (S. cerevisiae) // --- // 100	CDC123	Cluster0002
15065896	ENSECAT00000026987 // CDK10 // cyclin-dependent kinase 10 // --- // 100052321 /// XM_00	CDK10	Cluster0002
15127821	XM_001491879 // CDK16 // cyclin-dependent kinase 16 // --- // 100060831 /// XM_00336578	CDK16	Cluster0002
14957074	ENSECAT00000010295 // CDK19 // cyclin-dependent kinase 19 // --- // ---	CDK19	Cluster0002
15065801	XM_001488043 // CDT1 // chromatin licensing and DNA replication factor 1 // --- // 1000	CDT1	Cluster0002
15069822	XM_001499438 // CDYL2 // chromodomain protein, Y-like 2 // --- // 100069707 /// ENSECAT	CDYL2	Cluster0002
15104660	ENSECAT00000025639 // CEP164 // centrosomal protein 164kDa // --- // 100062791 /// XM_0	CEP164	Cluster0002
15072923	ENSECAT00000012839 // CEP170 // centrosomal protein 170kDa // --- // ---	CEP170	Cluster0002
15115639	XM_001489254 // CEP76 // centrosomal protein 76kDa // --- // 100050324 /// XM_003365561	CEP76	Cluster0002
15131559	XM_001492656 // CFP // complement factor properdin // --- // 100051317 /// ENSECAT00000	CFP	Cluster0002
15059308	NM_001242479 // CHADL // chondroadherin-like // --- // 100034030	CHADL	Cluster0002
15034605	ENSECAT00000013582 // CHERP // calcium homeostasis endoplasmic reticulum protein // ---	CHERP	Cluster0002
15064948	XM_001497119 // CIRH1A // cirrhosis, autosomal recessive 1A (cirhin) // --- // 10006696	CIRH1A	Cluster0002
14991986	XM_001495749 // CKAP2L // cytoskeleton associated protein 2-like // --- // 100052298 //	CKAP2L	Cluster0002
15077383	NM_001257104 // CLDN12 // claudin 12 // --- // 100060361 /// ENSECAT00000005680 // CLDN	CLDN12	Cluster0002
14981933	ENSECAT00000009622 // CLINT1 // clathrin interactor 1 // --- // ---	CLINT1	Cluster0002
14945848	ENSECAT00000024012 // CLK3 // CDC-like kinase 3 // --- // 100062320 /// XM_001917649 //	CLK3	Cluster0002
14950588	XM_001916976 // CLPTM1 // cleft lip and palate associated transmembrane protein 1 // --	CLPTM1	Cluster0002
15034294	ENSECAT00000014377 // CLPTM1L // CLPTM1-like // --- // 100071903	CLPTM1L	Cluster0002
14981640	ENSECAT00000017599 // CLTB // clathrin, light chain B // --- // ---	CLTB	Cluster0002
14991168	ENSECAT00000013227 // CMPK2 // cytidine monophosphate (UMP-CMP) kinase 2, mitochondrial	CMPK2	Cluster0002
14991728	ENSECAT00000010954 // CNNM4 // cyclin M4 // --- // 100062145	CNNM4	Cluster0002
15126218	XM_001914723 // CNOT2 // CCR4-NOT transcription complex, subunit 2 // --- // 100062938	CNOT2	Cluster0002
15082948	ENSECAT00000021792 // CNOT4 // CCR4-NOT transcription complex, subunit 4 // --- // 1000	CNOT4	Cluster0002
14964801	XM_001916362 // COG1 // component of oligomeric golgi complex 1 // --- // 100061294 ///	COG1	Cluster0002
15034835	XM_001503394 // COPE // coatomer protein complex, subunit epsilon // --- // 100069610 /	COPE	Cluster0002
14998713	XM_001488529 // COPG // coatomer protein complex, subunit gamma 1 // --- // 100050080 /	COPG	Cluster0002
14963755	ENSECAT00000022283 // COPS3 // COP9 constitutive photomorphogenic homolog subunit 3 (Ar	COPS3	Cluster0002
15045218	XM_001490168 // COQ6 // coenzyme Q6 homolog, monooxygenase (S. cerevisiae) // --- // 10	COQ6	Cluster0002
14976027	XM_001502009 // CORO1A // coronin, actin binding protein, 1A // --- // 100064170 /// EN	CORO1A	Cluster0002
15064272	ENSECAT00000020690 // CPNE2 // copine II // --- // ---	CPNE2	Cluster0002
15101248	XM_001503265 // CPNE8 // copine VIII // --- // 100064982 /// ENSECAT00000016311 // CPNE	CPNE8	Cluster0002
15019217	XM_001496373 // CPSF3L // cleavage and polyadenylation specific factor 3-like // --- //	CPSF3L	Cluster0002
15098284	ENSECAT00000016964 // CPSF6 // cleavage and polyadenylation specific factor 6, 68kDa //	CPSF6	Cluster0002
15052893	XM_001917228 // CRAT // carnitine O-acetyltransferase // --- // 100070062 /// ENSECAT00	CRAT	Cluster0002
15086539	XM_001495407 // CRTC2 // CREB regulated transcription coactivator 2 // --- // 100056571	CRTC2	Cluster0002
15058935	ENSECAT00000021519 // CSF2RB // colony stimulating factor 2 receptor, beta, low-affinit	CSF2RB	Cluster0002
14945826	XM_001492384 // CSK // c-src tyrosine kinase // --- // 100051462 /// ENSECAT00000014515	CSK	Cluster0002
15129047	ENSECAT00000009937 // CSTF2 // cleavage stimulation factor, 3 pre-RNA, subunit 2, 64kD	CSTF2	Cluster0002
14946971	XM_001502842 // CTDSPL2 // CTD (carboxy-terminal domain, RNA polymerase II, polypeptide	CTDSPL2	Cluster0002

15092213	XM_001498786 // CTTNBP2NL // CTTNBP2 N-terminal like // --- // 100059014 /// ENSECAT000	CTTNBP2NL	Cluster0002
15120713	NM_001159732 // CYB5A // cytochrome b5 type A (microsomal) // --- // 100052210 /// ENSE	CYB5A	Cluster0002
15081965	ENSECAT00000015009 // CYCS // cytochrome c, somatic // --- // 100053958 /// NM_00116401	CYCS	Cluster0002
15058946	ENSECAT00000026719 // CYTH4 // cytohesin 4 // --- // 100069735 /// XM_001499462 // CYTH	CYTH4	Cluster0002
15000425	XM_001497613 // DAG1 // dystroglycan 1 (dystrophin-associated glycoprotein 1) // --- //	DAG1	Cluster0002
15108641	XM_001503352 // DAPK3 // death-associated protein kinase 3 // --- // 100061244 /// ENSE	DAPK3	Cluster0002
15112387	ENSECAT00000009578 // DCHS1 // dachsous 1 (Drosophila) // --- // ---	DCHS1	Cluster0002
14995879	XM_001491404 // DCP1A // DCP1 decapping enzyme homolog A (S. cerevisiae) // --- // 1000	DCP1A	Cluster0002
15056483	XM_001492330 // DCTD // dCMP deaminase // --- // 100051279 /// ENSECAT00000018587 // LO	DCTD	Cluster0002
15103014	XM_001489083 // DCTN2 // dynactin 2 (p50) // --- // 100050464 /// XM_001489130 // DCTN2	DCTN2	Cluster0002
14982187	XM_001917648 // DCTN4 // dynactin 4 (p62) // --- // 100071625 /// XM_003362785 // DCTN4	DCTN4	Cluster0002
14981501	ENSECAT00000010318 // DDX41 // DEAD (Asp-Glu-Ala-Asp) box polypeptide 41 // --- // 1000	DDX41	Cluster0002
14966824	ENSECAT00000013254 // DDX52 // DEAD (Asp-Glu-Ala-Asp) box polypeptide 52 // --- // 1000	DDX52	Cluster0002
15080814	XM_001495821 // DDX56 // DEAD (Asp-Glu-Ala-Asp) box helicase 56 // --- // 100065131 ///	DDX56	Cluster0002
15090516	XM_001503843 // DEDD // death effector domain containing // --- // 100066256 /// XM_001	DEDD	Cluster0002
14936213	ENSECAT00000022645 // DET1 // de-etiolated homolog 1 (Arabidopsis) // --- // 100053984	DET1	Cluster0002
14969698	ENSECAT00000020744 // DGKZ // diacylglycerol kinase, zeta // --- // --- /// ENSECAT0000	DGKZ	Cluster0002
15000510	XM_001499602 // DHX30 // DEAH (Asp-Glu-Ala-His) box polypeptide 30 // --- // 100054034	DHX30	Cluster0002
15037905	ENSECAT00000015014 // DHX35 // DEAH (Asp-Glu-Ala-His) box polypeptide 35 // --- // 1000	DHX35	Cluster0002
15077233	ENSECAT00000022460 // DMTF1 // cyclin D binding myb-like transcription factor 1 // ---	DMTF1	Cluster0002
14980404	ENSECAT00000005377 // DNAJA3 // DnaJ (Hsp40) homolog, subfamily A, member 3 // --- // 1	DNAJA3	Cluster0002
15024760	XM_001490395 // DOK2 // docking protein 2, 56kDa // --- // 100053679 /// ENSECAT0000002	DOK2	Cluster0002
14970946	XM_001492616 // DPF2 // D4, zinc and double PHD fingers family 2 // --- // 100051328 //	DPF2	Cluster0002
15108715	ENSECAT00000027007 // DPP9 // dipeptidyl-peptidase 9 // --- // 100062517 /// XM_0033653	DPP9	Cluster0002
14978672	XM_003362699 // DTX2 // deltex homolog 2 (Drosophila) // --- // 100060906 /// ENSECAT00	DTX2	Cluster0002
15011927	ENSECAT00000009739 // DVL3 // dishevelled, dsh homolog 3 (Drosophila) // --- // ---	DVL3	Cluster0002
15064787	XM_001498275 // EDC4 // enhancer of mRNA decapping 4 // --- // 100053605 /// ENSECAT000	EDC4	Cluster0002
14973214	NM_001081785 // EEF1G // eukaryotic translation elongation factor 1 gamma // --- // 100	EEF1G	Cluster0002
14943095	ENSECAT00000011152 // EGR2 // early growth response 2 // --- // 100062890 /// XM_001503	EGR2	Cluster0002
15051121	XM_001918079 // EHMT1 // euchromatic histone-lysine N-methyltransferase 1 // --- // 100	EHMT1	Cluster0002
15089194	ENSECAT00000000249 // EIF2D // eukaryotic translation initiation factor 2D // --- // --	EIF2D	Cluster0002
15022756	XM_001503817 // EIF3I // eukaryotic translation initiation factor 3, subunit I // --- /	EIF3I	Cluster0002
15059038	XM_001501206 // EIF3L // eukaryotic translation initiation factor 3, subunit L // --- /	EIF3L	Cluster0002
15108335	XM_001502745 // EIF3M // eukaryotic translation initiation factor 3, subunit M // --- /	EIF3M	Cluster0002
15052696	XM_001500078 // ENG // endoglin // --- // 100070421 /// XM_003364144 // ENG // endoglin	ENG	Cluster0002
15034572	ENSECAT00000021166 // EPS15L1 // epidermal growth factor receptor pathway substrate 15-	EPS15L1	Cluster0002
15100712	ENSECAT00000016023 // EPS8 // epidermal growth factor receptor pathway substrate 8 // -	EPS8	Cluster0002
14980166	ENSECAT00000010995 // ERCC4 // excision repair cross-complementing rodent repair defici	ERCC4	Cluster0002
15033132	XM_001916106 // ERCC8 // excision repair cross-complementing rodent repair deficiency,	ERCC8	Cluster0002
15037603	ENSECAT00000013208 // ERGIC3 // ERGIC and golgi 3 // --- // ---	ERGIC3	Cluster0002
15126407	ENSECAT00000007956 // ESYT2 // extended synaptotagmin-like protein 2 // --- // 10005794	ESYT2	Cluster0002
15081700	ENSECAT00000021856 // ETV1 // ets variant 1 // --- // 100065470 /// XM_001495325 // ETV	ETV1	Cluster0002
15117773	XM_001495303 // EWSR1 // Ewing sarcoma breakpoint region 1 // --- // 100034010 /// ENSE	EWSR1	Cluster0002
14958526	XM_003362474 // EXOC7 // exocyst complex component 7 // --- // 100059108 /// ENSECAT000	EXOC7	Cluster0002
15017478	XM_001500462 // EYA3 // eyes absent homolog 3 (Drosophila) // --- // 100070791 /// ENSE	EYA3	Cluster0002
14994217	ENSECAT00000024757 // FAM59B // family with sequence similarity 59, member B // --- //	FAM59B	Cluster0002
14935414	ENSECAT00000017306 // FAM89A // family with sequence similarity 89, member A // --- //	FAM89A	Cluster0002
15069171	NM_001242528 // FAM96B // family with sequence similarity 96, member B // --- // 100052	FAM96B	Cluster0002
15105822	XM_001504881 // FARSA // phenylalanyl-tRNA synthetase, alpha subunit // --- // 10006327	FARSA	Cluster0002
15098880	XM_001495406 // FARSB // phenylalanyl-tRNA synthetase, beta subunit // --- // 100056565	FARSB	Cluster0002
14942833	ENSECAT00000014464 // FAS // Fas (TNF receptor superfamily, member 6) // --- // 1000716	FAS	Cluster0002
15095342	ENSECAT00000026686 // FBXL14 // F-box and leucine-rich repeat protein 14 // --- // ---	FBXL14	Cluster0002
15085566	XM_001503798 // FCER1G // Fc fragment of IgE, high affinity I, receptor for; gamma poly	FCER1G	Cluster0002
14987515	ENSECAT00000025525 // FCHO2 // FCH domain only 2 // --- // 100065702 /// XM_001504693 /	FCHO2	Cluster0002

14970688	XM_001488884 // FERMT3 // fermitin family member 3 // --- // 100050011 /// XM_001488969	FERMT3	Cluster0002
15091218	ENSECAT00000024367 // FLAD1 // FAD1 flavin adenine dinucleotide synthetase homolog (S.	FLAD1	Cluster0002
15105493	ENSECAT00000022701 // FLI1 // Friend leukemia virus integration 1 // --- // 100064591 /	FLI1	Cluster0002
14965306	ENSECAT00000024666 // FMNL1 // formin-like 1 // --- // 100064045	FMNL1	Cluster0002
14978127	ENSECAT00000015842 // FOXK1 // forkhead box K1 // --- // 100060644 /// XM_001492851 //	FOXK1	Cluster0002
14963856	ENSECAT00000022376 // FOXK2 // forkhead box K2 // --- // 100056711 /// XM_001490345 //	FOXK2	Cluster0002
14995322	ENSECAT00000009612 // FOXP1 // forkhead box P1 // --- // 100053199 /// XM_003363048 //	FOXP1	Cluster0002
15049853	ENSECAT00000013956 // FPGS // folylpolyglutamate synthase // --- // ---	FPGS	Cluster0002
14970959	ENSECAT00000007240 // FRMD8 // FERM domain containing 8 // --- // --- /// ENSECAT000000	FRMD8	Cluster0002
15126951	ENSECAT00000000154 // FRMPD4 // FERM and PDZ domain containing 4 // --- // 100050197 //	FRMPD4	Cluster0002
15098306	ENSECAT00000021508 // FRS2 // fibroblast growth factor receptor substrate 2 // --- // 1	FRS2	Cluster0002
15028370	ENSECAT00000016043 // FTSJD2 // FtsJ methyltransferase domain containing 2 // --- // --	FTSJD2	Cluster0002
15088549	XM_001496671 // FUBP1 // far upstream element (FUSE) binding protein 1 // --- // 100066	FUBP1	Cluster0002
15130596	ENSECAT00000029147 // FUNDC2 // FUN14 domain containing 2 // --- // ---	FUNDC2	Cluster0002
14934793	XM_001503903 // FUT11 // fucosyltransferase 11 (alpha (1,3) fucosyltransferase) // ---	FUT11	Cluster0002
15032975	XM_001500843 // GATAD2A // GATA zinc finger domain containing 2A // --- // 100071147 //	GATAD2A	Cluster0002
15086578	XM_001495330 // GATAD2B // GATA zinc finger domain containing 2B // --- // 100062097 //	GATAD2B	Cluster0002
15062688	ENSECAT00000009433 // GDI2 // GDP dissociation inhibitor 2 // --- // ---	GDI2	Cluster0002
15043368	XM_001491923 // GLIS3 // GLIS family zinc finger 3 // --- // 100051202 /// ENSECAT00000	GLIS3	Cluster0002
14985174	NM_001081912 // GM2A // GM2 ganglioside activator // --- // 100034082 /// ENSECAT000000	GM2A	Cluster0002
15092410	XM_001494890 // GNAI3 // guanine nucleotide binding protein (G protein), alpha inhibiti	GNAI3	Cluster0002
15045671	XM_001497455 // GOLGA5 // golgin A5 // --- // 100053313 /// ENSECAT00000010933 // GOLGA	GOLGA5	Cluster0002
15017600	XM_001504051 // GPATCH3 // G patch domain containing 3 // --- // 100057040 /// ENSECAT0	GPATCH3	Cluster0002
15044855	ENSECAT00000006887 // GPHN // gephyrin // --- // 100063078 /// ENSECAT00000006965 // GP	GPHN	Cluster0002
15131677	XM_001495227 // GPKOW // G patch domain and KOW motifs // --- // 100062702 /// XM_00336	GPKOW	Cluster0002
15108952	ENSECAT00000015263 // GPR108 // G protein-coupled receptor 108 // --- // --- /// ENSECA	GPR108	Cluster0002
15003788	XM_001916057 // GPR180 // G protein-coupled receptor 180 // --- // 100059006 /// ENSECA	GPR180	Cluster0002
14996446	NM_001166479 // GPX1 // glutathione peroxidase 1 // --- // 100053396 /// ENSECAT0000001	GPX1	Cluster0002
14950384	ENSECAT00000021007 // GSK3A // glycogen synthase kinase 3 alpha // --- // ---	GSK3A	Cluster0002
15063916	XM_001915211 // HEATR3 // HEAT repeat containing 3 // --- // 100058410 /// ENSECAT00000	HEATR3	Cluster0002
15026812	ENSECAT00000011146 // HIVEP1 // human immunodeficiency virus type I enhancer binding pr	HIVEP1	Cluster0002
14936835	XM_001490810 // HMG20A // high mobility group 20A // --- // 100061369 /// ENSECAT000000	HMG20A	Cluster0002
15018062	ENSECAT00000008232 // HP1BP3 // heterochromatin protein 1, binding protein 3 // --- //	HP1BP3	Cluster0002
15027712	NM_001256923 // HSPA1A // heat shock 70kDa protein 1A // --- // 100050827 /// AF397192	HSPA1A	Cluster0002
15111156	ENSECAT00000014969 // ICAM1 // intercellular adhesion molecule 1 // --- // ---	ICAM1	Cluster0002
15032093	ENSECAT00000023604 // ICK // intestinal cell (MAK-like) kinase // --- // 100056645 ///	ICK	Cluster0002
14942821	ENSECAT00000010390 // IFIT3 // interferon-induced protein with tetratricopeptide repeat	IFIT3	Cluster0002
14943690	XM_001498209 // IFT74 // intraflagellar transport 74 homolog (Chlamydomonas) // --- //	IFT74	Cluster0002
15042300	XM_001498209 // IFT74 // intraflagellar transport 74 homolog (Chlamydomonas) // --- //	IFT74	Cluster0002
14999113	ENSECAT00000009909 // IL17RC // interleukin 17 receptor C // --- // 100058271 /// XM_00	IL17RC	Cluster0002
15091244	ENSECAT00000025853 // IL6R // interleukin 6 receptor // --- // ---	IL6R	Cluster0002
14967664	ENSECAT00000017717 // INPP5K // inositol polyphosphate-5-phosphatase K // --- // ---	INPP5K	Cluster0002
15025032	XM_001493351 // INTS9 // integrator complex subunit 9 // --- // 100061398 /// XM_003364	INTS9	Cluster0002
14996394	ENSECAT00000017793 // IP6K1 // inositol hexakisphosphate kinase 1 // --- // 100052910 /	IP6K1	Cluster0002
15021917	ENSECAT00000016749 // IPO13 // importin 13 // --- // 100066178 /// XM_001496543 // IPO1	IPO13	Cluster0002
14955202	ENSECAT00000019481 // IRF3 // interferon regulatory factor 3 // --- // ---	IRF3	Cluster0002
15078776	ENSECAT00000010372 // IRF5 // interferon regulatory factor 5 // --- // 100071786 /// XM	IRF5	Cluster0002
15065723	ENSECAT00000016408 // IRF8 // interferon regulatory factor 8 // --- // 100056218 /// XM	IRF8	Cluster0002
15045772	NM_001114146 // ISG12(A) // IFN stimulated gene 12(A) // --- // 100064838 /// ENSECAT00	ISG12(A)	Cluster0002
15055602	XM_001490002 // ITGB2 // integrin, beta 2 (complement component 3 receptor 3 and 4 subu	ITGB2	Cluster0002
15047952	ENSECAT00000025731 // ITPK1 // inositol-tetrakisphosphate 1-kinase // --- // ---	ITPK1	Cluster0002
15069779	XM_003364638 // KARS // lysyl-tRNA synthetase // --- // 100055335 /// XM_001501578 // K	KARS	Cluster0002
14985828	XM_001918163 // KDM3B // lysine (K)-specific demethylase 3B // --- // 100062288 /// ENS	KDM3B	Cluster0002
15024699	ENSECAT00000024971 // KLHL17 // kelch-like 17 (Drosophila) // --- // 100146295 /// XM_0	KLHL17	Cluster0002

15097158	ENSECAT00000021817 // KRT7 // keratin 7 // --- // 100062790 /// XM_001504375 // KRT7 //	KRT7	Cluster0002
14985087	ENSECAT00000018194 // LARP1 // La ribonucleoprotein domain family, member 1 // --- // 1	LARP1	Cluster0002
14981746	ENSECAT00000023396 // LCP2 // lymphocyte cytosolic protein 2 (SH2 domain containing leu	LCP2	Cluster0002
14958252	XM_001490950 // LGALS3BP // lectin, galactoside-binding, soluble, 3 binding protein //	LGALS3BP	Cluster0002
14991332	ENSECAT00000018770 // LIMS1 // LIM and senescent cell antigen-like domains 1 // --- //	LIMS1	Cluster0002
14951037	ENSECAT00000021415 // LIN7B // lin-7 homolog B (C. elegans) // --- // ---	LIN7B	Cluster0002
14992482	ENSECAT00000004426 // LOC100049814 // STAM-binding protein-like // --- // 100049814	LOC100049814	Cluster0002
15055715	ENSECAT00000002632 // LOC100049837 // arylamine N-acetyltransferase 1-like // --- // 10	LOC100049837	Cluster0002
15075266	XM_001499329 // LOC100049846 // TATA-box-binding protein-like // --- // 100049846 /// E	LOC100049846	Cluster0002
15111346	ENSECAT00000027025 // LOC100049857 // e3 ubiquitin-protein ligase MARCH2-like // --- //	LOC100049857	Cluster0002
15113304	XM_003365441 // LOC100049858 // mediator of RNA polymerase II transcription subunit 15-	LOC100049858	Cluster0002
14959534	XM_001487945 // LOC100049943 // Golgi SNAP receptor complex member 2-like // --- // 100	LOC100049943	Cluster0002
15098340	ENSECAT00000000020 // LOC100049987 // uncharacterized protein C2orf67-like // --- // 10	LOC100049987	Cluster0002
15118199	ENSECAT00000023785 // LOC100050056 // malectin-like // --- // 100050056 /// XM_00148852	LOC100050056	Cluster0002
15115610	ENSECAT00000013895 // LOC100050057 // gamma-soluble NSF attachment protein-like // ---	LOC100050057	Cluster0002
14970700	XM_001489010 // LOC100050076 // nucleoside diphosphate-linked moiety X motif 22-like //	LOC100050076	Cluster0002
15113421	XM_001489060 // LOC100050121 // small nuclear ribonucleoprotein Sm D3-like // --- // 10	LOC100050121	Cluster0002
14940439	XM_001489582 // LOC100050137 // AP-4 complex subunit sigma-1-like // --- // 100050137 /	LOC100050137	Cluster0002
15013610	ENSECAT00000014453 // LOC100050155 // PRELI domain-containing protein 1, mitochondrial-	LOC100050155	Cluster0002
15065861	XM_001488825 // LOC100050173 // 60S ribosomal protein L13-like // --- // 100050173 ///	LOC100050173	Cluster0002
14973517	ENSECAT00000017936 // LOC100050210 // tRNA methyltransferase 112 homolog // --- // 1000	LOC100050210	Cluster0002
15038511	XM_001489360 // LOC100050229 // cleavage stimulation factor subunit 1-like // --- // 10	LOC100050229	Cluster0002
14964064	XM_001489490 // LOC100050276 // charged multivesicular body protein 6-like // --- // 10	LOC100050276	Cluster0002
14994805	ENSECAT00000018570 // LOC100050283 // ras-related protein Rab-43-like // --- // 1000502	LOC100050283	Cluster0002
15041749	XM_001916945 // LOC100050300 // Krueppel-like factor 9-like // --- // 100050300 /// ENS	LOC100050300	Cluster0002
14972235	XM_001489345 // LOC100050344 // peroxisomal membrane protein PEX16-like // --- // 10005	LOC100050344	Cluster0002
15044308	XM_001495733 // LOC100050367 // galectin-3-like // --- // 100050367 /// ENSECAT00000006	LOC100050367	Cluster0002
15049214	ENSECAT00000020834 // LOC100050369 // high affinity copper uptake protein 1-like // ---	LOC100050369	Cluster0002
15106634	ENSECAT00000001822 // LOC100050389 // ras-related protein Rab-38-like // --- // 1000503	LOC100050389	Cluster0002
15089119	XM_001490736 // LOC100050458 // ras-related protein Rab-7L1-like // --- // 100050458 //	LOC100050458	Cluster0002
14971988	XM_001488475 // LOC100050482 // ribonuclease inhibitor-like // --- // 100050482 /// ENS	LOC100050482	Cluster0002
15127004	XM_003365792 // LOC100050487 // ras-related protein Rab-9A-like // --- // 100050487 ///	LOC100050487	Cluster0002
15006813	ENSECAT00000007599 // LOC100050511 // ADP-ribosylation factor-like protein 6-interactin	LOC100050511	Cluster0002
15056840	XM_001490279 // LOC100050524 // golgin subfamily A member 7-like // --- // 100050524 //	LOC100050524	Cluster0002
15030545	XM_001917482 // LOC100050550 // MHC class I antigen 3.7 // --- // 100050550 /// ENSECAT	LOC100050550	Cluster0002
15130771	ENSECAT00000014823 // LOC100050563 // trafficking protein particle complex subunit 2-li	LOC100050563	Cluster0002
15079530	ENSECAT00000004777 // LOC100050611 // caspase-2-like // --- // 100050611 /// XM_0014956	LOC100050611	Cluster0002
15005720	ENSECAT00000007725 // LOC100050664 // serine/threonine-protein kinase 24-like // --- //	LOC100050664	Cluster0002
14950926	XM_001488495 // LOC100050794 // epithelial membrane protein 3-like // --- // 100050794	LOC100050794	Cluster0002
14972034	XM_001488795 // LOC100050797 // interferon-induced transmembrane protein 3-like // ---	LOC100050797	Cluster0002
14970848	XM_001491691 // LOC100050811 // sorting nexin-15-like // --- // 100050811 /// ENSECAT00	LOC100050811	Cluster0002
15029644	XM_001489822 // LOC100050826 // neuritin-like // --- // 100050826 /// ENSECAT0000000711	LOC100050826	Cluster0002
15054351	ENSECAT00000014793 // LOC100050835 // cystatin-B-like // --- // 100050835 /// XM_001491	LOC100050835	Cluster0002
15073525	XM_001915707 // LOC100050843 // regulator of G-protein signaling 18-like // --- // 1000	LOC100050843	Cluster0002
15117372	ENSECAT00000018670 // LOC100050855 // crk-like protein-like // --- // 100050855 /// XM_	LOC100050855	Cluster0002
14944092	XM_001492754 // LOC100050879 // tubulin-specific chaperone E-like // --- // 100050879 /	LOC100050879	Cluster0002
15101638	XM_001490907 // LOC100050927 // ankyrin repeat and SOCS box protein 8-like // --- // 10	LOC100050927	Cluster0002
15123700	XM_001914853 // LOC100050939 // nibrin-like // --- // 100050939 /// ENSECAT00000014953	LOC100050939	Cluster0002
14954951	XM_001488542 // LOC100050944 // ER lumen protein retaining receptor 1-like // --- // 10	LOC100050944	Cluster0002
14963748	ENSECAT00000021845 // LOC100050945 // phosphatidylethanolamine N-methyltransferase-like	LOC100050945	Cluster0002
15103052	XM_001489894 // LOC100051005 // cyclin-dependent kinase 4-like // --- // 100051005 ///	LOC100051005	Cluster0002
15126056	ENSECAT00000014659 // LOC100051023 // integral membrane protein DGCR2/IDD-like // --- /	LOC100051023	Cluster0002
15024111	ENSECAT00000023185 // LOC100051052 // type-1 angiotensin II receptor-associated protein	LOC100051052	Cluster0002
14972045	XM_001489209 // LOC100051094 // BET1-like protein-like // --- // 100051094 /// ENSECAT0	LOC100051094	Cluster0002

14949479	XM_001492237 // LOC100051102 // RNA-binding protein 42-like // --- // 100051102 /// XM_	LOC100051102	Cluster0002
15018553	XM_001492165 // LOC100051122 // mitotic spindle assembly checkpoint protein MAD2B-like	LOC100051122	Cluster0002
15038755	XM_001915306 // LOC100051201 // proteasomal ubiquitin receptor ADRM1-like // --- // 100	LOC100051201	Cluster0002
15027480	ENSECAT00000024112 // LOC100051230 // 28S ribosomal protein S18b, mitochondrial-like //	LOC100051230	Cluster0002
15099853	ENSECAT00000023705 // LOC100051294 // uncharacterized protein C12orf4 homolog // --- //	LOC100051294	Cluster0002
14958544	XM_001915669 // LOC100051325 // signal recognition particle 68 kDa protein-like // ---	LOC100051325	Cluster0002
14959863	XM_001490840 // LOC100051326 // protein LSM12 homolog // --- // 100051326 /// ENSECAT00	LOC100051326	Cluster0002
14999822	XM_001915127 // LOC100051333 // protein kinase C delta type-like // --- // 100051333 //	LOC100051333	Cluster0002
15003426	ENSECAT00000009881 // LOC100051337 // epithelial-stromal interaction protein 1-like //	LOC100051337	Cluster0002
15084114	XM_001493038 // LOC100051363 // POU domain, class 2, transcription factor 1-like // ---	LOC100051363	Cluster0002
14974452	ENSECAT00000020571 // LOC100051383 // toll-interacting protein-like // --- // 100051383	LOC100051383	Cluster0002
15102567	XM_001504778 // LOC100051450 // CD63 antigen-like // --- // 100051450 /// ENSECAT000000	LOC100051450	Cluster0002
15131576	XM_001492729 // LOC100051459 // protein UXT-like // --- // 100051459 /// ENSECAT0000001	LOC100051459	Cluster0002
14995050	ENSECAT00000021499 // LOC100051473 // transcription cofactor vestigial-like protein 4-l	LOC100051473	Cluster0002
15027840	XM_001493316 // LOC100051483 // lysosomal thioesterase PPT2-like // --- // 100051483 //	LOC100051483	Cluster0002
15045880	ENSECAT00000020074 // LOC100051490 // cyclin-K-like // --- // 100051490	LOC100051490	Cluster0002
15097530	XM_001504781 // LOC100051518 // ORM1-like protein 2-like // --- // 100051518 /// ENSECA	LOC100051518	Cluster0002
14963647	XM_001488126 // LOC100051522 // serine hydroxymethyltransferase, cytosolic-like // ---	LOC100051522	Cluster0002
14948874	XM_001494060 // LOC100051535 // 40S ribosomal protein S29-like // --- // 100051535 ///	LOC100051535	Cluster0002
14949543	ENSECAT00000007805 // LOC100051536 // gamma-secretase subunit PEN-2-like // --- // 1000	LOC100051536	Cluster0002
14971062	XM_001494184 // LOC100051541 // histone acetyltransferase KAT5-like // --- // 100051541	LOC100051541	Cluster0002
15081477	XM_001492902 // LOC100051572 // BET1 homolog // --- // 100051572 /// ENSECAT00000025977	LOC100051572	Cluster0002
14940251	ENSECAT00000026310 // LOC100051589 // interferon regulatory factor 9-like // --- // 100	LOC100051589	Cluster0002
14949548	XM_001492756 // LOC100051603 // protein lin-37 homolog // --- // 100051603 /// ENSECAT0	LOC100051603	Cluster0002
14995989	ENSECAT00000000752 // LOC100051614 // glycosyltransferase 8 domain-containing protein 1	LOC100051614	Cluster0002
15027858	XM_001493452 // LOC100051626 // e3 ubiquitin-protein ligase RNF5-like // --- // 1000516	LOC100051626	Cluster0002
15038914	XM_001495050 // LOC100051629 // dnaJ homolog subfamily C member 5-like // --- // 100051	LOC100051629	Cluster0002
15111775	XM_001494053 // LOC100051651 // protein EMSY-like // --- // 100051651 /// ENSECAT000000	LOC100051651	Cluster0002
15024309	ENSECAT00000019902 // LOC100051697 // SPRY domain-containing SOCS box protein 1-like //	LOC100051697	Cluster0002
15041299	XM_001495344 // LOC100051702 // regulator of G-protein signaling 19-like // --- // 1000	LOC100051702	Cluster0002
15119714	ENSECAT00000028913 // LOC100051723 // oxysterol-binding protein-related protein 1-like	LOC100051723	Cluster0002
14953710	ENSECAT00000019582 // LOC100051740 // TYRO protein tyrosine kinase-binding protein-like	LOC100051740	Cluster0002
15111832	XM_001494844 // LOC100051780 // 40S ribosomal protein S3-like // --- // 100051780 /// E	LOC100051780	Cluster0002
14963678	XM_001918207 // LOC100051793 // protein flightless-1 homolog // --- // 100051793 /// EN	LOC100051793	Cluster0002
15131614	ENSECAT00000010426 // LOC100051797 // mitochondrial import inner membrane translocase s	LOC100051797	Cluster0002
15070839	XM_001492667 // LOC100051833 // 60S ribosomal protein L21-like // --- // 100051833 ///	LOC100051833	Cluster0002
15119752	XM_001494100 // LOC100051844 // protein SSXT-like // --- // 100051844 /// ENSECAT000000	LOC100051844	Cluster0002
14995095	XM_001494063 // LOC100051933 // transmembrane protein 111-like // --- // 100051933 ///	LOC100051933	Cluster0002
15066585	ENSECAT00000019091 // LOC100051954 // transmembrane protein 150C-like // --- // 1000519	LOC100051954	Cluster0002
14951141	XM_001491984 // LOC100051978 // 40S ribosomal protein S11-like // --- // 100051978 ///	LOC100051978	Cluster0002
15131653	ENSECAT00000022104 // LOC100051983 // transcription factor E3-like // --- // 100051983	LOC100051983	Cluster0002
15016270	ENSECAT00000023840 // LOC100052003 // protein O-linked-mannose beta-1,2-N-acetylglucosa	LOC100052003	Cluster0002
15097672	XM_003365264 // LOC100052035 // myosin light polypeptide 6-like // --- // 100052035 ///	LOC100052035	Cluster0002
14965544	XM_001492387 // LOC100052050 // interferon-induced 35 kDa protein-like // --- // 100052	LOC100052050	Cluster0002
14971156	ENSECAT00000026135 // LOC100052051 // barrier-to-autointegration factor-like // --- //	LOC100052051	Cluster0002
15073844	XM_001495138 // LOC100052076 // mitochondrial import inner membrane translocase subunit	LOC100052076	Cluster0002
14963704	ENSECAT00000020441 // LOC100052098 // ATP synthase mitochondrial F1 complex assembly fa	LOC100052098	Cluster0002
15128012	XM_001495293 // LOC100052104 // proteolipid protein 2-like // --- // 100052104 /// ENSE	LOC100052104	Cluster0002
14992966	XM_001917544 // LOC100052114 // poly(A) polymerase gamma-like // --- // 100052114 /// E	LOC100052114	Cluster0002
14999142	XM_001494365 // LOC100052115 // protein jagunal homolog 1-like // --- // 100052115 ///	LOC100052115	Cluster0002
14996124	XM_001494476 // LOC100052116 // abhydrolase domain-containing protein 14B-like // --- /	LOC100052116	Cluster0002
14972570	XM_001488291 // LOC100052157 // olfactory receptor 5M9-like // --- // 100052157 /// ENS	LOC100052157	Cluster0002
14972175	XM_001488298 // LOC100052172 // COMM domain-containing protein 9-like // --- // 1000521	LOC100052172	Cluster0002
15055220	ENSECAT00000011402 // LOC100052191 // quinone oxidoreductase-like protein 1-like // ---	LOC100052191	Cluster0002

15064330	XM_001494211 // LOC100052195 // DNA-directed RNA polymerase II subunit RPB3-like // ---	LOC100052195	Cluster0002
15074750	ENSECAT00000022656 // LOC100052198 // ADP-ribosylation factor-like protein 8A-like // -	LOC100052198	Cluster0002
15043689	ENSECAT00000026587 // LOC100052253 // perilipin-2-like // --- // 100052253 /// XM_00191	LOC100052253	Cluster0002
14946015	XM_001494920 // LOC100052291 // sentrin-specific protease 8-like // --- // 100052291 //	LOC100052291	Cluster0002
14959983	ENSECAT00000019623 // LOC100052295 // beclin-1-like // --- // 100052295 /// XM_00149322	LOC100052295	Cluster0002
15045469	XM_001493951 // LOC100052429 // SRA stem-loop-interacting RNA-binding protein, mitochon	LOC100052429	Cluster0002
15100078	XM_003365166 // LOC100052443 // myeloid leukemia factor 2-like // --- // 100052443 ///	LOC100052443	Cluster0002
14996262	ENSECAT00000016313 // LOC100052473 // tumor suppressor candidate 2-like // --- // 10005	LOC100052473	Cluster0002
15036859	ENSECAT00000022931 // LOC100052483 // UPF0687 protein C20orf27-like // --- // 100052483	LOC100052483	Cluster0002
14965646	ENSECAT00000013111 // LOC100052528 // tubulin gamma-2 chain-like // --- // 100052528 //	LOC100052528	Cluster0002
15039563	XM_001496329 // LOC100052544 // inosine triphosphate pyrophosphatase-like // --- // 100	LOC100052544	Cluster0002
14955587	ENSECAT00000013117 // LOC100052583 // leukocyte receptor cluster member 1-like // --- /	LOC100052583	Cluster0002
14965653	XM_001493847 // LOC100052586 // tubulin gamma-1 chain-like // --- // 100052586 /// ENSE	LOC100052586	Cluster0002
15069031	ENSECAT00000017874 // LOC100052607 // aspartate aminotransferase, mitochondrial-like //	LOC100052607	Cluster0002
15095725	XM_001497472 // LOC100052671 // triosephosphate isomerase-like // --- // 100052671 ///	LOC100052671	Cluster0002
15114154	ENSECAT00000007660 // LOC100052676 // signal peptide peptidase-like 3-like // --- // 10	LOC100052676	Cluster0002
15009906	XM_001497963 // LOC100052812 // methyltransferase-like protein 5-like // --- // 1000528	LOC100052812	Cluster0002
15047046	XM_001499883 // LOC100052868 // phosphatidylinositol N-acetylglucosaminyltransferase su	LOC100052868	Cluster0002
15100091	ENSECAT00000018013 // LOC100052934 // prohibitin-2-like // --- // 100052934 /// XM_0014	LOC100052934	Cluster0002
15037005	XM_001497067 // LOC100052969 // isocitrate dehydrogenase [NAD] subunit beta, mitochondr	LOC100052969	Cluster0002
14960117	XM_001494956 // LOC100053004 // ras-related protein Rab-5C-like // --- // 100053004 ///	LOC100053004	Cluster0002
14971463	ENSECAT00000013162 // LOC100053005 // ribosomal protein S6 kinase beta-2-like // --- //	LOC100053005	Cluster0002
15037017	XM_001497470 // LOC100053021 // small nuclear ribonucleoprotein-associated proteins B a	LOC100053021	Cluster0002
15068371	ENSECAT00000002811 // LOC100053044 // uncharacterized LOC100053044 // --- // 100053044	LOC100053044	Cluster0002
14965720	ENSECAT00000018960 // LOC100053094 // NF-kappa-B inhibitor-interacting Ras-like protein	LOC100053094	Cluster0002
15114171	ENSECAT00000006233 // LOC100053127 // 2-methoxy-6-polyprenyl-1,4-benzoquinol methylase,	LOC100053127	Cluster0002
14992180	XM_001498803 // LOC100053147 // e3 ubiquitin-protein ligase RNF181-like // --- // 10005	LOC100053147	Cluster0002
14996429	XM_001497632 // LOC100053200 // nicolin-1-like // --- // 100053200 /// ENSECAT000000116	LOC100053200	Cluster0002
14971495	XM_001498106 // LOC100053249 // glutathione S-transferase P-like // --- // 100053249 //	LOC100053249	Cluster0002
15023737	XM_001914692 // LOC100053258 // adaptin ear-binding coat-associated protein 2-like // -	LOC100053258	Cluster0002
15039736	XM_001498911 // LOC100053261 // proteasome inhibitor PI31 subunit-like // --- // 100053	LOC100053261	Cluster0002
15030167	XM_001505039 // LOC100053279 // histone H2B type 1-K-like // --- // 100053279 /// ENSEC	LOC100053279	Cluster0002
15072675	ENSECAT00000026437 // LOC100053283 // protein FAM193A-like // --- // 100053283 /// ENSE	LOC100053283	Cluster0002
14963787	ENSECAT00000023675 // LOC100053286 // dehydrogenase/reductase SDR family member 7B-like	LOC100053286	Cluster0002
15016514	XM_001498548 // LOC100053305 // elongation of very long chain fatty acids protein 1-lik	LOC100053305	Cluster0002
15126067	ENSECAT00000015346 // LOC100053332 // protein DGCR14-like // --- // 100053332 /// XM_00	LOC100053332	Cluster0002
14971519	ENSECAT00000010424 // LOC100053392 // NADH dehydrogenase [ubiquinone] iron-sulfur prote	LOC100053392	Cluster0002
15118238	XM_001488666 // LOC100053420 // cytochrome c oxidase subunit 6A1, mitochondrial-like //	LOC100053420	Cluster0002
15028217	XM_001918090 // LOC100053449 // 60S ribosomal protein L10a-like // --- // 100053449 ///	LOC100053449	Cluster0002
15030165	XM_001505043 // LOC100053472 // histone H2B type 1-J-like // --- // 100053472 /// ENSEC	LOC100053472	Cluster0002
15030131	XM_001497402 // LOC100053499 // histone H2B type 1-like // --- // 100053499 /// ENSECAT	LOC100053499	Cluster0002
15074323	XM_001488034 // LOC100053608 // 3 (2),5 -bisphosphate nucleotidase 1-like // --- // 10	LOC100053608	Cluster0002
15117264	ENSECAT00000013321 // LOC100053618 // macrophage migration inhibitory factor-like // --	LOC100053618	Cluster0002
14953461	XM_001488752 // LOC100053634 // cytochrome b-c1 complex subunit Rieske, mitochondrial-l	LOC100053634	Cluster0002
14996539	XM_001498296 // LOC100053641 // glutaminyl-tRNA synthetase-like // --- // 100053641 ///	LOC100053641	Cluster0002
15022164	XM_001503121 // LOC100053643 // peptidyl-prolyl cis-trans isomerase H-like // --- // 10	LOC100053643	Cluster0002
14992400	XM_001500046 // LOC100053688 // serine protease HTRA2, mitochondrial-like // --- // 100	LOC100053688	Cluster0002
15056734	XM_001488787 // LOC100053704 // protein farnesyltransferase/geranylgeranyltransferase t	LOC100053704	Cluster0002
14998666	XM_001488819 // LOC100053785 // monoglyceride lipase-like // --- // 100053785 /// ENSEC	LOC100053785	Cluster0002
15085655	XM_001504441 // LOC100053868 // CD48 antigen-like // --- // 100053868 /// ENSECAT000000	LOC100053868	Cluster0002
15095973	ENSECAT00000015525 // LOC100053911 // gamma-aminobutyric acid receptor-associated prote	LOC100053911	Cluster0002
14959379	XM_001500833 // LOC100053934 // SWI/SNF-related matrix-associated actin-dependent regul	LOC100053934	Cluster0002
15001139	ENSECAT00000002299 // LOC100053940 // myeloid differentiation primary response protein	LOC100053940	Cluster0002
15069423	ENSECAT00000009199 // LOC100054003 // chromosome transmission fidelity protein 8 homolo	LOC100054003	Cluster0002

14978256	ENSECAT00000012348 // LOC100054023 // Golgi to ER traffic protein 4 homolog // --- // 1	LOC100054023	Cluster0002
14951689	ENSECAT00000010799 // LOC100054029 // leukocyte immunoglobulin-like receptor subfamily	LOC100054029	Cluster0002
15101335	ENSECAT00000012553 // LOC100054055 // YY1-associated factor 2-like // --- // 100054055	LOC100054055	Cluster0002
15068311	XM_001917737 // LOC100054064 // alpha-2-macroglobulin receptor-associated protein-like	LOC100054064	Cluster0002
15035960	XM_001500700 // LOC100054195 // UPF0489 protein C5orf22-like // --- // 100054195 /// EN	LOC100054195	Cluster0002
14969914	XM_001489004 // LOC100054215 // olfactory receptor 10A7-like // --- // 100054215 /// EN	LOC100054215	Cluster0002
14965055	ENSECAT00000015308 // LOC100054228 // translational activator of cytochrome c oxidase 1	LOC100054228	Cluster0002
15117284	XM_001489018 // LOC100054251 // cationic amino acid transporter 4-like // --- // 100054	LOC100054251	Cluster0002
15027586	XM_001489019 // LOC100054254 // MHC class I polypeptide-related sequence B-like // ---	LOC100054254	Cluster0002
14972593	XM_001489022 // LOC100054258 // olfactory receptor 1019-like // --- // 100054258 /// EN	LOC100054258	Cluster0002
14973472	ENSECAT00000021404 // LOC100054275 // atlastin-3-like // --- // 100054275 /// XM_001488	LOC100054275	Cluster0002
15030145	XM_001498193 // LOC100054284 // histone H3.1-like // --- // 100054284 /// ENSECAT000000	LOC100054284	Cluster0002
14969916	XM_001489042 // LOC100054307 // olfactory receptor 5AP2-like // --- // 100054307 /// EN	LOC100054307	Cluster0002
15086997	XM_001488599 // LOC100054350 // splicing factor 3B subunit 4-like // --- // 100054350 /	LOC100054350	Cluster0002
15045850	XM_001489078 // LOC100054383 // poly(A) polymerase alpha-like // --- // 100054383 /// E	LOC100054383	Cluster0002
15053552	ENSECAT00000005235 // LOC100054431 // CGG triplet repeat-binding protein 1-like // ---	LOC100054431	Cluster0002
15022437	XM_001503545 // LOC100054471 // probable U3 small nucleolar RNA-associated protein 11-l	LOC100054471	Cluster0002
15016778	ENSECAT00000012199 // LOC100054519 // four and a half LIM domains protein 3-like // ---	LOC100054519	Cluster0002
15118294	ENSECAT00000025211 // LOC100054578 // sin3 histone deacetylase corepressor complex comp	LOC100054578	Cluster0002
15040027	XM_001501536 // LOC100054650 // eukaryotic translation initiation factor 6-like // ---	LOC100054650	Cluster0002
14960581	XM_001501076 // LOC100054686 // protein C17orf37 homolog // --- // 100054686 /// ENSECA	LOC100054686	Cluster0002
14993745	ENSECAT00000015187 // LOC100054688 // xanthine dehydrogenase/oxidase-like // --- // 100	LOC100054688	Cluster0002
15051573	ENSECAT00000018079 // LOC100054749 // endoplasmic reticulum resident protein 44-like //	LOC100054749	Cluster0002
15059001	ENSECAT00000011392 // LOC100054799 // nucleolar protein 12-like // --- // 100054799 ///	LOC100054799	Cluster0002
14970661	XM_001489294 // LOC100054829 // ubiquitin thioesterase OTUB1-like // --- // 100054829 /	LOC100054829	Cluster0002
14993900	ENSECAT00000019494 // LOC100054872 // BRCA1-A complex subunit BRE-like // --- // 100054	LOC100054872	Cluster0002
15016938	XM_001503613 // LOC100054878 // 28S ribosomal protein S15, mitochondrial-like // --- //	LOC100054878	Cluster0002
15044120	XM_001917854 // LOC100054905 // spindlin-1-like // --- // 100054905 /// ENSECAT00000026	LOC100054905	Cluster0002
15132747	ENSECAT00000005033 // LOC100054910 // importin subunit alpha-2-like // --- // 100054910	LOC100054910	Cluster0002
15025108	XM_001496538 // LOC100054913 // PIN2/TERF1-interacting telomerase inhibitor 1-like // -	LOC100054913	Cluster0002
15031643	XM_001501628 // LOC100054928 // male-enhanced antigen 1-like // --- // 100054928 /// EN	LOC100054928	Cluster0002
15059067	ENSECAT00000025369 // LOC100054935 // DNA-directed RNA polymerases I, II, and III subun	LOC100054935	Cluster0002
15121402	XM_001914675 // LOC100054947 // v-type proton ATPase subunit H-like // --- // 100054947	LOC100054947	Cluster0002
15086918	XM_001490481 // LOC100054948 // cathepsin S-like // --- // 100054948 /// ENSECAT0000002	LOC100054948	Cluster0002
14974360	XM_001494157 // LOC100055087 // interferon regulatory factor 7-like // --- // 100055087	LOC100055087	Cluster0002
15044235	ENSECAT00000012109 // LOC100055223 // hippocampus abundant transcript-like protein 1-li	LOC100055223	Cluster0002
14958079	XM_001488427 // LOC100055281 // protein FAM195B-like // --- // 100055281 /// ENSECAT000	LOC100055281	Cluster0002
14940123	ENSECAT00000006600 // LOC100055307 // neuroguidin-like // --- // 100055307	LOC100055307	Cluster0002
14966049	ENSECAT00000000672 // LOC100055317 // 39S ribosomal protein L45, mitochondrial-like //	LOC100055317	Cluster0002
15031711	ENSECAT00000018028 // LOC100055327 // protein YIPF3-like // --- // 100055327 /// XM_001	LOC100055327	Cluster0002
15037743	XM_001502264 // LOC100055328 // protein MANBAL-like // --- // 100055328 /// ENSECAT0000	LOC100055328	Cluster0002
15091547	XM_001491678 // LOC100055347 // tumor necrosis factor alpha-induced protein 8-like prot	LOC100055347	Cluster0002
15040286	XM_003363924 // LOC100055373 // bladder cancer-associated protein-like // --- // 100055	LOC100055373	Cluster0002
15091539	ENSECAT00000025732 // LOC100055389 // sodium channel modifier 1-like // --- // 10005538	LOC100055389	Cluster0002
15111213	XM_001493654 // LOC100055392 // peptidyl-prolyl cis-trans isomerase NIMA-interacting 1-	LOC100055392	Cluster0002
14966080	XM_001501874 // LOC100055406 // 39S ribosomal protein L10, mitochondrial-like // --- //	LOC100055406	Cluster0002
15123877	ENSECAT00000022575 // LOC100055428 // uncharacterized protein C8orf37 homolog // --- //	LOC100055428	Cluster0002
15111217	XM_001493676 // LOC100055431 // ubiquitin-like protein 5-like // --- // 100055431 /// E	LOC100055431	Cluster0002
15030434	ENSECAT00000009940 // LOC100055517 // nurim-like // --- // 100055517 /// XM_001491340 /	LOC100055517	Cluster0002
14951122	XM_001489661 // LOC100055522 // fms-related tyrosine kinase 3 ligand-like // --- // 100	LOC100055522	Cluster0002
14994113	ENSECAT00000011520 // LOC100055570 // transmembrane protein 214-like // --- // 10005557	LOC100055570	Cluster0002
15078357	ENSECAT00000013011 // LOC100055586 // dnaJ homolog subfamily B member 9-like // --- //	LOC100055586	Cluster0002
14963988	ENSECAT00000011767 // LOC100055613 // hepatocyte growth factor-regulated tyrosine kinas	LOC100055613	Cluster0002
14940101	XM_001493694 // LOC100055644 // polyadenylate-binding protein 2-like // --- // 10005564	LOC100055644	Cluster0002

15003205	XM_001488678 // LOC100055705 // importin subunit alpha-3-like // --- // 100055705 /// E	LOC100055705	Cluster0002
15051240	ENSECAT00000019337 // LOC100055711 // uncharacterized protein KIAA1539-like // --- // 1	LOC100055711	Cluster0002
14994221	XM_001502813 // LOC100055747 // ras-related protein Rab-10-like // --- // 100055747 ///	LOC100055747	Cluster0002
15063264	XM_003364353 // LOC100055759 // COMM domain-containing protein 3-like // --- // 1000557	LOC100055759	Cluster0002
14955199	XM_001917334 // LOC100055781 // ras-related protein R-Ras-like // --- // 100055781 ///	LOC100055781	Cluster0002
15082409	ENSECAT00000014657 // LOC100055933 // transcription factor EC-like // --- // 100055933	LOC100055933	Cluster0002
15017254	XM_001503810 // LOC100055965 // myotubularin-related protein 9-like // --- // 100055965	LOC100055965	Cluster0002
14971836	XM_001489918 // LOC100055991 // transaldolase-like // --- // 100055991 /// ENSECAT00000	LOC100055991	Cluster0002
15127605	ENSECAT00000002908 // LOC100055994 // mid1-interacting protein 1-like // --- // 1000559	LOC100055994	Cluster0002
15075817	XM_001489928 // LOC100056012 // brachyury protein-like // --- // 100056012 /// ENSECAT0	LOC100056012	Cluster0002
15018529	ENSECAT00000014686 // LOC100056045 // uncharacterized protein KIAA2013 homolog // --- /	LOC100056045	Cluster0002
15059461	XM_001502727 // LOC100056050 // UPF0466 protein C22orf32, mitochondrial-like // --- //	LOC100056050	Cluster0002
15094190	XM_001492847 // LOC100056095 // AN1-type zinc finger protein 2B-like // --- // 10005609	LOC100056095	Cluster0002
15017354	ENSECAT00000022702 // LOC100056122 // peflin-like // --- // 100056122 /// XM_001503887	LOC100056122	Cluster0002
15038136	ENSECAT00000014936 // LOC100056125 // elafin-like // --- // 100056125 /// XM_001503186	LOC100056125	Cluster0002
15123048	ENSECAT00000014351 // LOC100056139 // transcription factor E2F5-like // --- // 10005613	LOC100056139	Cluster0002
15055586	XM_001490058 // LOC100056215 // ubiquitin-conjugating enzyme E2 G2-like // --- // 10005	LOC100056215	Cluster0002
14963903	ENSECAT00000010567 // LOC100056238 // COP9 signalosome complex subunit 1-like // --- //	LOC100056238	Cluster0002
14990739	XM_001503183 // LOC100056247 // pre-mRNA branch site protein p14-like // --- // 1000562	LOC100056247	Cluster0002
15038597	ENSECAT00000013948 // LOC100056253 // vesicle-associated membrane protein-associated pr	LOC100056253	Cluster0002
14955261	XM_001493330 // LOC100056315 // nuclear pore glycoprotein p62-like // --- // 100056315	LOC100056315	Cluster0002
15085326	ENSECAT00000003128 // LOC100056345 // UPF0739 protein C1orf74 homolog // --- // 1000563	LOC100056345	Cluster0002
14940052	ENSECAT00000016572 // LOC100056354 // mitochondrial inner membrane protein OXA1L-like /	LOC100056354	Cluster0002
15091398	ENSECAT00000022278 // LOC100056390 // Friend of PRMT1 protein-like // --- // 100056390	LOC100056390	Cluster0002
14990846	XM_001503353 // LOC100056409 // lysosomal-associated transmembrane protein 4A-like // -	LOC100056409	Cluster0002
15091393	ENSECAT00000021710 // LOC100056434 // SNARE-associated protein Snapin-like // --- // 10	LOC100056434	Cluster0002
15132026	XM_001490186 // LOC100056440 // PDZ domain-containing protein 11-like // --- // 1000564	LOC100056440	Cluster0002
14972220	ENSECAT00000025635 // LOC100056452 // synaptotagmin-13-like // --- // 100056452 /// XM_	LOC100056452	Cluster0002
15068197	ENSECAT00000025766 // LOC100056472 // protein S100-P-like // --- // 100056472 /// XM_00	LOC100056472	Cluster0002
15044204	XM_001490235 // LOC100056530 // uncharacterized LOC100056530 // --- // 100056530 /// EN	LOC100056530	Cluster0002
14966386	ENSECAT00000016323 // LOC100056543 // cytochrome c oxidase assembly protein COX11, mito	LOC100056543	Cluster0002
15024766	XM_003364489 // LOC100056631 // nucleoside diphosphate-linked moiety X motif 18-like //	LOC100056631	Cluster0002
14980249	XM_001916473 // LOC100056639 // cytosolic Fe-S cluster assembly factor NUBP1-like // --	LOC100056639	Cluster0002
15027391	ENSECAT00000016672 // LOC100056703 // DNA-directed RNA polymerase I subunit RPA12-like	LOC100056703	Cluster0002
15106013	XM_001490341 // LOC100056704 // transcription elongation factor 1 homolog // --- // 100	LOC100056704	Cluster0002
14970888	XM_001492016 // LOC100056723 // 39S ribosomal protein L49, mitochondrial-like // --- //	LOC100056723	Cluster0002
15041011	ENSECAT00000000477 // LOC100056734 // protein slowmo homolog 2-like // --- // 100056734	LOC100056734	Cluster0002
14947149	ENSECAT00000012882 // LOC100056798 // calpain-3-like // --- // 100056798 /// ENSECAT000	LOC100056798	Cluster0002
14940028	ENSECAT00000003088 // LOC100056825 // abhydrolase domain-containing protein 4-like // -	LOC100056825	Cluster0002
15052048	ENSECAT00000019674 // LOC100056842 // thymic stromal cotransporter homolog // --- // 10	LOC100056842	Cluster0002
15086464	ENSECAT00000018792 // LOC100056861 // uncharacterized protein C1orf43 homolog // --- //	LOC100056861	Cluster0002
15017528	XM_001504025 // LOC100056884 // wiskott-Aldrich syndrome protein family member 2-like /	LOC100056884	Cluster0002
14966515	ENSECAT00000017502 // LOC100056960 // transcription elongation factor SPT4-like // ---	LOC100056960	Cluster0002
15090762	XM_001490503 // LOC100056988 // myeloid cell nuclear differentiation antigen-like // --	LOC100056988	Cluster0002
15045260	XM_001490976 // LOC100057010 // rRNA-processing protein FCF1 homolog // --- // 10005701	LOC100057010	Cluster0002
15018695	ENSECAT00000018718 // LOC100057041 // DNA fragmentation factor subunit alpha-like // --	LOC100057041	Cluster0002
14991091	XM_001503580 // LOC100057123 // 14-3-3 protein theta-like // --- // 100057123 /// ENSEC	LOC100057123	Cluster0002
15001394	ENSECAT00000019622 // LOC100057124 // glycerol-3-phosphate dehydrogenase 1-like protein	LOC100057124	Cluster0002
14957907	ENSECAT00000010590 // LOC100057193 // casein kinase I isoform delta-like // --- // 1000	LOC100057193	Cluster0002
15091154	ENSECAT00000020867 // LOC100057221 // sugar transporter SWEET1-like // --- // 100057221	LOC100057221	Cluster0002
14972644	XM_001490643 // LOC100057228 // olfactory receptor 8J3-like // --- // 100057228 /// ENS	LOC100057228	Cluster0002
15045306	XM_001490655 // LOC100057247 // translation initiation factor eIF-2B subunit beta-like	LOC100057247	Cluster0002
15013580	XM_001915284 // LOC100057287 // translocon-associated protein subunit gamma-like // ---	LOC100057287	Cluster0002
15124441	XM_001916266 // LOC100057344 // derlin-1-like // --- // 100057344 /// ENSECAT0000002549	LOC100057344	Cluster0002

15086309	XM_001498730 // LOC100057345 // secretory carrier-associated membrane protein 3-like //	LOC100057345	Cluster0002
15111015	ENSECAT00000004374 // LOC100057430 // uncharacterized protein C19orf52-like // --- // 1	LOC100057430	Cluster0002
15062782	ENSECAT00000025128 // LOC100057457 // Krueppel-like factor 6-like // --- // 100057457 /	LOC100057457	Cluster0002
14973802	XM_001490817 // LOC100057483 // transcription factor p65-like // --- // 100057483 /// E	LOC100057483	Cluster0002
15013831	XM_001915861 // LOC100057566 // polyhomeotic-like protein 3-like // --- // 100057566 //	LOC100057566	Cluster0002
14947591	ENSECAT00000011324 // LOC100057596 // uncharacterized protein C15orf41-like // --- // 1	LOC100057596	Cluster0002
14973815	XM_001494534 // LOC100057603 // cofilin-1-like // --- // 100057603 /// ENSECAT000000211	LOC100057603	Cluster0002
14992467	ENSECAT00000014783 // LOC100057605 // deoxyguanosine kinase, mitochondrial-like // ---	LOC100057605	Cluster0002
15111040	ENSECAT00000024627 // LOC100057627 // dynamin-2-like // --- // 100057627	LOC100057627	Cluster0002
15024815	ENSECAT00000023912 // LOC100057635 // bridging integrator 3-like // --- // 100057635 //	LOC100057635	Cluster0002
15017790	XM_001504204 // LOC100057686 // hydroxymethylglutaryl-CoA lyase, mitochondrial-like //	LOC100057686	Cluster0002
15054285	ENSECAT00000003259 // LOC100057691 // NADH dehydrogenase [ubiquinone] flavoprotein 3, m	LOC100057691	Cluster0002
15133776	ENSECAT00000020048 // LOC100057896 // isocitrate dehydrogenase [NAD] subunit gamma, mit	LOC100057896	Cluster0002
14973847	ENSECAT00000026110 // LOC100057911 // probable RNA-binding protein EIF1AD-like // --- /	LOC100057911	Cluster0002
15090985	ENSECAT00000014727 // LOC100057939 // apolipoprotein A-I-binding protein-like // --- //	LOC100057939	Cluster0002
14939458	XM_001503674 // LOC100057999 // transmembrane protein 85-like // --- // 100057999 /// E	LOC100057999	Cluster0002
14969830	XM_001491132 // LOC100058004 // zinc transporter ZIP13-like // --- // 100058004 /// ENS	LOC100058004	Cluster0002
15119386	XM_001489204 // LOC100058028 // proteasome assembly chaperone 2-like // --- // 10005802	LOC100058028	Cluster0002
14989013	ENSECAT00000009766 // LOC100058052 // bolA-like protein 3-like // --- // 100058052 ///	LOC100058052	Cluster0002
14981473	XM_001502076 // LOC100058092 // H/ACA ribonucleoprotein complex subunit 2-like // --- /	LOC100058092	Cluster0002
15013983	ENSECAT00000013311 // LOC100058096 // zinc finger matrin-type protein 3-like // --- //	LOC100058096	Cluster0002
15023462	XM_001504261 // LOC100058097 // complement C1q subcomponent subunit A-like // --- // 10	LOC100058097	Cluster0002
15030600	ENSECAT00000024180 // LOC100058098 // patr class I histocompatibility antigen, A-126 al	LOC100058098	Cluster0002
14999059	XM_001493888 // LOC100058184 // probable protein BRICK1-like // --- // 100058184 /// EN	LOC100058184	Cluster0002
15114725	XM_001491288 // LOC100058244 // protein phosphatase PTC7 homolog // --- // 100058244 //	LOC100058244	Cluster0002
15106245	XM_001491320 // LOC100058299 // sphingosine 1-phosphate receptor 2-like // --- // 10005	LOC100058299	Cluster0002
15094009	XM_001915148 // LOC100058331 // actin-related protein 2/3 complex subunit 2-like // ---	LOC100058331	Cluster0002
15057185	XM_003364229 // LOC100058367 // RNA-binding protein with multiple splicing-like // ---	LOC100058367	Cluster0002
15057193	XM_001495715 // LOC100058409 // dynactin subunit 6-like // --- // 100058409 /// ENSECAT	LOC100058409	Cluster0002
15080237	XM_001488867 // LOC100058412 // dnaJ homolog subfamily C member 2-like // --- // 100058	LOC100058412	Cluster0002
15119399	XM_001491395 // LOC100058421 // tubulin beta-5 chain-like // --- // 100058421 /// ENSEC	LOC100058421	Cluster0002
14981567	XM_001502371 // LOC100058443 // vesicular integral-membrane protein VIP36-like // --- /	LOC100058443	Cluster0002
15117609	XM_001497319 // LOC100058463 // RING finger protein 185-like // --- // 100058463 /// EN	LOC100058463	Cluster0002
15120901	XM_001915132 // LOC100058465 // 28S ribosomal protein S28, mitochondrial-like // --- //	LOC100058465	Cluster0002
15111188	XM_001491424 // LOC100058468 // UPF0515 protein C19orf66 homolog // --- // 100058468 //	LOC100058468	Cluster0002
15085717	XM_001491425 // LOC100058470 // peroxisomal biogenesis factor 19-like // --- // 1000584	LOC100058470	Cluster0002
14999206	ENSECAT00000022269 // LOC100058531 // myotubularin-related protein 14-like // --- // 10	LOC100058531	Cluster0002
15111419	XM_001916685 // LOC100058546 // protein KIAA1731-like // --- // 100058546 /// ENSECAT00	LOC100058546	Cluster0002
14939579	ENSECAT00000001230 // LOC100058565 // enhancer of rudimentary homolog // --- // 1000585	LOC100058565	Cluster0002
15094086	XM_001491531 // LOC100058624 // cell differentiation protein RCD1 homolog // --- // 100	LOC100058624	Cluster0002
14947888	XM_001505132 // LOC100058648 // transmembrane protein 55B-like // --- // 100058648 ///	LOC100058648	Cluster0002
15027656	XM_001917690 // LOC100058658 // casein kinase II subunit beta-like // --- // 100058658	LOC100058658	Cluster0002
15114833	XM_001496472 // LOC100058668 // 26S proteasome non-ATPase regulatory subunit 9-like //	LOC100058668	Cluster0002
15118724	ENSECAT00000021600 // LOC100058709 // diablo homolog, mitochondrial-like // --- // 1000	LOC100058709	Cluster0002
14932781	ENSECAT00000020131 // LOC100058723 // uncharacterized protein C10orf84-like // --- // 1	LOC100058723	Cluster0002
14973945	ENSECAT00000024543 // LOC100058729 // RNA-binding protein 4B-like // --- // 100058729 /	LOC100058729	Cluster0002
15091713	ENSECAT00000012027 // LOC100058752 // pleckstrin homology domain-containing family O me	LOC100058752	Cluster0002
14961924	XM_001504075 // LOC100058771 // galectin-9C-like // --- // 100058771 /// XM_001504079 /	LOC100058771	Cluster0002
14998450	XM_001491640 // LOC100058811 // glycogenin-1-like // --- // 100058811 /// ENSECAT000000	LOC100058811	Cluster0002
15091689	XM_001489373 // LOC100058935 // 28S ribosomal protein S21, mitochondrial-like // --- //	LOC100058935	Cluster0002
15097534	ENSECAT00000011075 // LOC100058940 // transmembrane protein 198-B-like // --- // 100058	LOC100058940	Cluster0002
15119428	XM_001490037 // LOC100058979 // proteasome assembly chaperone 2-like // --- // 10005897	LOC100058979	Cluster0002
15051115	XM_001917558 // LOC100059025 // arrestin domain-containing protein 1-like // --- // 100	LOC100059025	Cluster0002
14984667	ENSECAT00000000976 // LOC100059037 // 60S ribosomal protein L26-like 1-like // --- // 1	LOC100059037	Cluster0002

14970296	XM_001501678 // LOC100059110 // transmembrane protein 109-like // --- // 100059110 ///	LOC100059110	Cluster0002
15063976	XM_001915288 // LOC100059123 // nucleotide-binding oligomerization domain-containing pr	LOC100059123	Cluster0002
15118895	ENSECAT00000022871 // LOC100059129 // translation initiation factor eIF-2B subunit alph	LOC100059129	Cluster0002
15120912	ENSECAT00000022041 // LOC100059131 // interleukin-7-like // --- // 100059131 /// XM_001	LOC100059131	Cluster0002
15070936	ENSECAT00000026405 // LOC100059167 // 39S ribosomal protein L1, mitochondrial-like // -	LOC100059167	Cluster0002
14991577	ENSECAT00000025843 // LOC100059235 // 60S ribosomal protein L31-like // --- // 10005923	LOC100059235	Cluster0002
15043389	XM_001917215 // LOC100059240 // GTP:AMP phosphotransferase, mitochondrial-like // --- /	LOC100059240	Cluster0002
14967386	XM_001504154 // LOC100059307 // stromal cell-derived factor 2-like // --- // 100059307	LOC100059307	Cluster0002
15098813	XM_001491978 // LOC100059355 // aspartyl aminopeptidase-like // --- // 100059355 /// EN	LOC100059355	Cluster0002
14977843	ENSECAT00000007813 // LOC100059364 // cleavage and polyadenylation specificity factor s	LOC100059364	Cluster0002
15130484	ENSECAT00000014632 // LOC100059369 // emerin-like // --- // 100059369	LOC100059369	Cluster0002
14947996	XM_001505161 // LOC100059376 // ras-related protein Rab-2B-like // --- // 100059376 ///	LOC100059376	Cluster0002
14974563	ENSECAT00000002287 // LOC100059401 // ATP synthase subunit f, mitochondrial-like // ---	LOC100059401	Cluster0002
14974040	ENSECAT00000019733 // LOC100059453 // membrane-associated phosphatidylinositol transfer	LOC100059453	Cluster0002
15087392	XM_001500064 // LOC100059469 // GTPase NRas-like // --- // 100059469 /// ENSECAT0000001	LOC100059469	Cluster0002
15094270	XM_001492055 // LOC100059471 // mannose-1-phosphate guanyltransferase alpha-like // ---	LOC100059471	Cluster0002
14967411	ENSECAT00000012433 // LOC100059494 // uncharacterized protein C17orf63-like // --- // 1	LOC100059494	Cluster0002
15030901	ENSECAT00000015111 // LOC100059501 // 1-acyl-sn-glycerol-3-phosphate acyltransferase al	LOC100059501	Cluster0002
15075675	ENSECAT00000009872 // LOC100059508 // dynein light chain Tctex-type 1-like // --- // 10	LOC100059508	Cluster0002
14964498	XM_001495435 // LOC100059530 // SAP30-binding protein-like // --- // 100059530 /// ENSE	LOC100059530	Cluster0002
14962064	ENSECAT00000014162 // LOC100059531 // GTPase Era, mitochondrial-like // --- // 10005953	LOC100059531	Cluster0002
14971490	XM_001492090 // LOC100059533 // glutathione S-transferase P-like // --- // 100059533 //	LOC100059533	Cluster0002
14971500	ENSECAT00000022916 // LOC100059569 // NADH dehydrogenase [ubiquinone] flavoprotein 1, m	LOC100059569	Cluster0002
15018880	ENSECAT00000025347 // LOC100059575 // cytosolic acyl coenzyme A thioester hydrolase-lik	LOC100059575	Cluster0002
14974861	ENSECAT00000012678 // LOC100059590 // putative ribosomal RNA methyltransferase 2-like /	LOC100059590	Cluster0002
14978598	XM_001504462 // LOC100059592 // mitochondrial fission 1 protein-like // --- // 10005959	LOC100059592	Cluster0002
14949449	XM_001915953 // LOC100059603 // transmembrane protein 147-like // --- // 100059603 ///	LOC100059603	Cluster0002
14978230	ENSECAT00000009517 // LOC100059629 // 7,8-dihydro-8-oxoguanine triphosphatase-like // -	LOC100059629	Cluster0002
14978630	XM_001504472 // LOC100059659 // probable alpha-ketoglutarate-dependent dioxygenase ABH4	LOC100059659	Cluster0002
14978634	XM_001504471 // LOC100059695 // DNA-directed RNA polymerase II subunit RPB11-like // --	LOC100059695	Cluster0002
15126614	ENSECAT00000010657 // LOC100059698 // small glutamine-rich tetratricopeptide repeat-con	LOC100059698	Cluster0002
15114791	ENSECAT00000013459 // LOC100059726 // calcium release-activated calcium channel protein	LOC100059726	Cluster0002
14975442	XM_001504476 // LOC100059731 // 14-3-3 protein gamma-like // --- // 100059731 /// ENSEC	LOC100059731	Cluster0002
14978696	XM_001504478 // LOC100059763 // heat shock protein beta-1-like // --- // 100059763 ///	LOC100059763	Cluster0002
14962186	XM_001504253 // LOC100059771 // Golgi SNAP receptor complex member 1-like // --- // 100	LOC100059771	Cluster0002
14941987	ENSECAT00000025914 // LOC100059833 // outcome predictor in acute leukemia 1-like // ---	LOC100059833	Cluster0002
14936651	XM_001492287 // LOC100059835 // pro-cathepsin H-like // --- // 100059835 /// ENSECAT000	LOC100059835	Cluster0002
15102842	XM_001492303 // LOC100059858 // prostaglandin E synthase 3-like // --- // 100059858 ///	LOC100059858	Cluster0002
15111234	XM_001492338 // LOC100059920 // zinc finger protein 426-like // --- // 100059920 /// EN	LOC100059920	Cluster0002
15095644	XM_001496881 // LOC100059941 // COP9 signalosome complex subunit 7a-like // --- // 1000	LOC100059941	Cluster0002
15091467	XM_003365031 // LOC100059946 // tuftelin-like // --- // 100059946 /// ENSECAT0000001160	LOC100059946	Cluster0002
14944137	ENSECAT00000025059 // LOC100059958 // cancer-related nucleoside-triphosphatase-like //	LOC100059958	Cluster0002
14933244	XM_001498878 // LOC100059986 // CUE domain-containing protein 2-like // --- // 10005998	LOC100059986	Cluster0002
15087182	XM_001501227 // LOC100059997 // vesicle-trafficking protein SEC22b-like // --- // 10005	LOC100059997	Cluster0002
14974815	ENSECAT00000021390 // LOC100060005 // guanine nucleotide-binding protein subunit alpha-	LOC100060005	Cluster0002
15046870	ENSECAT00000011872 // LOC100060062 // dehydrogenase/reductase SDR family member 7-like	LOC100060062	Cluster0002
14989117	XM_001492476 // LOC100060120 // protein TEX261-like // --- // 100060120 /// ENSECAT0000	LOC100060120	Cluster0002
14962339	ENSECAT00000005331 // LOC100060145 // ovarian cancer-associated gene 2 protein homolog	LOC100060145	Cluster0002
15008689	ENSECAT00000015334 // LOC100060199 // ras-related protein Ral-B-like // --- // 10006019	LOC100060199	Cluster0002
14973302	XM_001502760 // LOC100060210 // UBX domain-containing protein 1-like // --- // 10006021	LOC100060210	Cluster0002
15089479	ENSECAT00000026316 // LOC100060224 // phosphatidylinositol N-acetylglucosaminyltransfer	LOC100060224	Cluster0002
15076135	XR_036390 // LOC100060323 // proteasome subunit beta type-2-like // --- // 100060323 //	LOC100060323	Cluster0002
15024938	XM_001915185 // LOC100060341 // tripartite motif-containing protein 35-like // --- // 1	LOC100060341	Cluster0002
15127342	ENSECAT00000018712 // LOC100060382 // eukaryotic translation initiation factor 2 subuni	LOC100060382	Cluster0002

15021368	XM_001915662 // LOC100060394 // tetratricopeptide repeat protein 4-like // --- // 10006	LOC100060394	Cluster0002
15084370	XM_001492668 // LOC100060398 // protein osteopotentia homolog // --- // 100060398 /// E	LOC100060398	Cluster0002
15024477	XM_001492683 // LOC100060427 // DNA fragmentation factor subunit beta-like // --- // 10	LOC100060427	Cluster0002
15108549	XM_001494016 // LOC100060448 // mitochondrial import inner membrane translocase subunit	LOC100060448	Cluster0002
14933390	XM_001500281 // LOC100060451 // NADH dehydrogenase [ubiquinone] 1 beta subcomplex subun	LOC100060451	Cluster0002
14962444	XM_001504725 // LOC100060628 // transmembrane protein 93-like // --- // 100060628 /// E	LOC100060628	Cluster0002
14975753	XM_001499574 // LOC100060727 // coiled-coil-helix-coiled-coil-helix domain-containing p	LOC100060727	Cluster0002
15100216	XM_001498661 // LOC100060780 // adaptin ear-binding coat-associated protein 1-like // -	LOC100060780	Cluster0002
15103940	XM_001498213 // LOC100060851 // 40S ribosomal protein S28-like // --- // 100060851 ///	LOC100060851	Cluster0002
14978914	XM_001499915 // LOC100060879 // UPF0458 protein C7orf42-like // --- // 100060879 /// EN	LOC100060879	Cluster0002
15027919	XM_001493006 // LOC100060885 // proteasome subunit beta type-9-like // --- // 100060885	LOC100060885	Cluster0002
15120939	XM_001493018 // LOC100060902 // transcription elongation factor B polypeptide 1-like //	LOC100060902	Cluster0002
14987821	ENSECAT00000020846 // LOC100060911 // ADP-ribosylation factor 1-like // --- // 10006091	LOC100060911	Cluster0002
14933622	XM_001501347 // LOC100060949 // arginine vasopressin-induced protein 1-like // --- // 1	LOC100060949	Cluster0002
14944212	XM_001494310 // LOC100060950 // protein ARV1-like // --- // 100060950 /// ENSECAT000000	LOC100060950	Cluster0002
14992679	XM_001490697 // LOC100060958 // u4/U6.U5 small nuclear ribonucleoprotein 27 kDa protein	LOC100060958	Cluster0002
14972141	ENSECAT00000006602 // LOC100060978 // probable methylthioribulose-1-phosphate dehydrata	LOC100060978	Cluster0002
14984243	ENSECAT00000004417 // LOC100060980 // histone H2B type 1-L-like // --- // 100060980	LOC100060980	Cluster0002
14964777	ENSECAT00000024658 // LOC100060986 // protein tweety homolog 2-like // --- // 100060986	LOC100060986	Cluster0002
15109189	ENSECAT00000014032 // LOC100061007 // protein CWC15 homolog // --- // 100061007 /// XM_	LOC100061007	Cluster0002
14962585	XM_001504739 // LOC100061027 // derlin-2-like // --- // 100061027 /// ENSECAT0000001050	LOC100061027	Cluster0002
14968189	ENSECAT00000012405 // LOC100061089 // RPA-interacting protein-like // --- // 100061089	LOC100061089	Cluster0002
14975453	XM_001493164 // LOC100061105 // transmembrane protein 120A-like // --- // 100061105 ///	LOC100061105	Cluster0002
14945741	XM_001493197 // LOC100061150 // tetraspanin-3-like // --- // 100061150 /// ENSECAT00000	LOC100061150	Cluster0002
15131860	ENSECAT00000005799 // LOC100061180 // testis-specific Y-encoded-like protein 2-like //	LOC100061180	Cluster0002
14978980	ENSECAT00000026699 // LOC100061188 // protein NipSnap homolog 2-like // --- // 10006118	LOC100061188	Cluster0002
15127861	ENSECAT00000025939 // LOC100061212 // serine/threonine-protein kinase A-Raf-like // ---	LOC100061212	Cluster0002
14968243	XM_001504749 // LOC100061251 // e3 ubiquitin-protein ligase RNF167-like // --- // 10006	LOC100061251	Cluster0002
14984254	XM_001494754 // LOC100061285 // 39S ribosomal protein L55, mitochondrial-like // --- //	LOC100061285	Cluster0002
15039160	XM_001489907 // LOC100061304 // n-alpha-acetyltransferase 20, NatB catalytic subunit-li	LOC100061304	Cluster0002
15071656	ENSECAT00000025177 // LOC100061309 // SLAIN motif-containing protein 2-like // --- // 1	LOC100061309	Cluster0002
15111622	XM_001493368 // LOC100061424 // transmembrane protein 126B-like // --- // 100061424 ///	LOC100061424	Cluster0002
14962705	XM_001504756 // LOC100061442 // c-X-C motif chemokine 16-like // --- // 100061442 /// E	LOC100061442	Cluster0002
15027958	XM_001493382 // LOC100061452 // e3 ubiquitin-protein ligase RING1-like // --- // 100061	LOC100061452	Cluster0002
15075298	XM_001493402 // LOC100061484 // mitochondrial dimethyladenosine transferase 1-like // -	LOC100061484	Cluster0002
14933903	XM_001502179 // LOC100061542 // cytochrome P450 2C19-like // --- // 100061542 /// ENSEC	LOC100061542	Cluster0002
15092420	XM_003365077 // LOC100061557 // cytochrome b561 domain-containing protein 1-like // ---	LOC100061557	Cluster0002
15100256	ENSECAT00000023553 // LOC100061559 // cation-dependent mannose-6-phosphate receptor-lik	LOC100061559	Cluster0002
14945777	ENSECAT00000015570 // LOC100061677 // COMM domain-containing protein 4-like // --- // 1	LOC100061677	Cluster0002
14978946	ENSECAT00000011475 // LOC100061681 // DNA-directed RNA polymerase III subunit RPC9-like	LOC100061681	Cluster0002
14978770	ENSECAT00000016225 // LOC100061703 // b-cell CLL/lymphoma 7 protein family member B-lik	LOC100061703	Cluster0002
14968442	ENSECAT00000011216 // LOC100061712 // G protein pathway suppressor 2-like // --- // 100	LOC100061712	Cluster0002
14996235	XM_001493584 // LOC100061750 // nitrogen permease regulator 2-like protein-like // ---	LOC100061750	Cluster0002
15031178	ENSECAT00000025691 // LOC100061755 // putative Bcl-2 homologous antagonist/killer 2-lik	LOC100061755	Cluster0002
15102165	ENSECAT00000019712 // LOC100061764 // keratin, type II cytoskeletal 1-like // --- // 10	LOC100061764	Cluster0002
15109637	XM_001501779 // LOC100061921 // alpha-crystallin B chain-like // --- // 100061921 /// E	LOC100061921	Cluster0002
15092334	XM_001493712 // LOC100061953 // protein FAM40A-like // --- // 100061953 /// ENSECAT0000	LOC100061953	Cluster0002
14991723	XM_001493723 // LOC100061973 // cytochrome c oxidase subunit 5B, mitochondrial-like //	LOC100061973	Cluster0002
14996282	XM_001496249 // LOC100061975 // interferon-related developmental regulator 2-like // --	LOC100061975	Cluster0002
14936941	XM_001493806 // LOC100062111 // secretory carrier-associated membrane protein 2-like //	LOC100062111	Cluster0002
15097292	XM_001504512 // LOC100062123 // UPF0160 protein MYG1, mitochondrial-like // --- // 1000	LOC100062123	Cluster0002
15130624	ENSECAT00000002112 // LOC100062203 // prefoldin subunit 3-like // --- // 100062203 ///	LOC100062203	Cluster0002
15042499	ENSECAT00000010602 // LOC100062234 // semaphorin-4D-like // --- // 100062234	LOC100062234	Cluster0002
14968547	ENSECAT00000011785 // LOC100062246 // trafficking protein particle complex subunit 1-li	LOC100062246	Cluster0002

15115331	XM_001493904 // LOC100062278 // tRNA pseudouridine synthase A, mitochondrial-like // --	LOC100062278	Cluster0002
14978005	ENSECAT00000007741 // LOC100062309 // eukaryotic translation initiation factor 2-alpha	LOC100062309	Cluster0002
14955952	XM_003362313 // LOC100062322 // zinc finger protein 256-like // --- // 100062322 /// EN	LOC100062322	Cluster0002
15031223	ENSECAT00000005973 // LOC100062360 // 40S ribosomal protein S10-like // --- // 10006236	LOC100062360	Cluster0002
15089686	ENSECAT00000011389 // LOC100062369 // uncharacterized LOC100062369 // --- // 100062369	LOC100062369	Cluster0002
15100438	XM_001493960 // LOC100062370 // oxidized low-density lipoprotein receptor 1-like // ---	LOC100062370	Cluster0002
14970396	XM_001502117 // LOC100062392 // succinate dehydrogenase assembly factor 2, mitochondria	LOC100062392	Cluster0002
15064321	ENSECAT00000008347 // LOC100062499 // ubiquinone biosynthesis protein COQ9, mitochondri	LOC100062499	Cluster0002
15004807	XM_001495632 // LOC100062531 // protein FAM48A-like // --- // 100062531 /// ENSECAT0000	LOC100062531	Cluster0002
15047013	XM_003364029 // LOC100062536 // protein max-like // --- // 100062536 /// XM_001494066 /	LOC100062536	Cluster0002
14963225	XM_001504818 // LOC100062561 // ran guanine nucleotide release factor-like // --- // 10	LOC100062561	Cluster0002
15003002	XM_001495546 // LOC100062567 // exosome complex component RRP43-like // --- // 10006256	LOC100062567	Cluster0002
14973119	XM_001494112 // LOC100062603 // UPF0197 transmembrane protein C11orf10 homolog // --- /	LOC100062603	Cluster0002
14942763	ENSECAT00000017042 // LOC100062658 // polycomb group RING finger protein 5-like // ---	LOC100062658	Cluster0002
15042928	XM_001494169 // LOC100062680 // transducin-like enhancer protein 4-like // --- // 10006	LOC100062680	Cluster0002
15020509	ENSECAT00000029039 // LOC100062717 // sodium/bile acid cotransporter 7-like // --- // 1	LOC100062717	Cluster0002
15045606	ENSECAT00000009414 // LOC100062752 // calmodulin-like // --- // 100062752	LOC100062752	Cluster0002
14973150	ENSECAT00000006983 // LOC100062780 // guanine nucleotide exchange factor for Rab-3A-lik	LOC100062780	Cluster0002
14985972	XM_001504379 // LOC100062781 // UPF0461 protein C5orf24-like // --- // 100062781 /// EN	LOC100062781	Cluster0002
14951475	XM_001917999 // LOC100062799 // electron transfer flavoprotein subunit beta-like // ---	LOC100062799	Cluster0002
15020562	ENSECAT00000026243 // LOC100062837 // anaphase-promoting complex subunit 10-like // ---	LOC100062837	Cluster0002
15117517	XM_001494300 // LOC100062878 // 14-3-3 protein eta-like // --- // 100062878 /// ENSECAT	LOC100062878	Cluster0002
15030204	XM_001494338 // LOC100062934 // histone H2B type 2-F-like // --- // 100062934 /// ENSEC	LOC100062934	Cluster0002
15033337	ENSECAT00000015662 // LOC100062980 // ADP-ribosylation factor-like protein 15-like // -	LOC100062980	Cluster0002
15042967	ENSECAT00000016395 // LOC100063011 // uncharacterized LOC100063011 // --- // 100063011	LOC100063011	Cluster0002
15109929	ENSECAT00000015047 // LOC100063051 // intraflagellar transport protein 46 homolog // --	LOC100063051	Cluster0002
15084585	ENSECAT00000019110 // LOC100063083 // torsin-3A-like // --- // 100063083 /// XM_0014944	LOC100063083	Cluster0002
15035449	XM_001494494 // LOC100063168 // NADH dehydrogenase [ubiquinone] iron-sulfur protein 4,	LOC100063168	Cluster0002
15111700	ENSECAT00000018577 // LOC100063181 // aquaporin-11-like // --- // 100063181 /// XM_0014	LOC100063181	Cluster0002
15105800	XM_001504875 // LOC100063222 // syntaxin-10-like // --- // 100063222 /// ENSECAT0000001	LOC100063222	Cluster0002
15125274	ENSECAT00000006916 // LOC100063246 // vacuolar protein sorting-associated protein 28 ho	LOC100063246	Cluster0002
14975790	XM_001500509 // LOC100063252 // apoptosis-associated speck-like protein containing a CA	LOC100063252	Cluster0002
15110015	XM_001503132 // LOC100063303 // UDP-N-acetylglucosamine--dolichyl-phosphate N-acetylglu	LOC100063303	Cluster0002
15086375	ENSECAT00000007364 // LOC100063346 // dolichol-phosphate mannosyltransferase subunit 3-	LOC100063346	Cluster0002
14975814	XM_001500789 // LOC100063360 // vitamin K epoxide reductase complex subunit 1-like // -	LOC100063360	Cluster0002
15086369	ENSECAT00000020676 // LOC100063378 // keratinocyte-associated protein 2-like // --- //	LOC100063378	Cluster0002
15091132	XM_001494688 // LOC100063479 // metaxin-1-like // --- // 100063479 /// ENSECAT000000077	LOC100063479	Cluster0002
14986255	ENSECAT00000026576 // LOC100063560 // protein PRRC1-like // --- // 100063560 /// XM_001	LOC100063560	Cluster0002
15042173	ENSECAT00000005636 // LOC100063565 // ras-related GTP-binding protein A-like // --- //	LOC100063565	Cluster0002
15111763	ENSECAT00000029021 // LOC100063572 // alkaline ceramidase 3-like // --- // 100063572 //	LOC100063572	Cluster0002
15096034	XM_001501469 // LOC100063606 // retinoic acid-induced protein 3-like // --- // 10006360	LOC100063606	Cluster0002
15133741	ENSECAT00000000695 // LOC100063613 // centrin-2-like // --- // 100063613 /// XM_0014947	LOC100063613	Cluster0002
15041410	XM_001494977 // LOC100063910 // fructose-1,6-bisphosphatase 1-like // --- // 100063910	LOC100063910	Cluster0002
14970574	XM_001495003 // LOC100063952 // transmembrane protein 179B-like // --- // 100063952 ///	LOC100063952	Cluster0002
14973343	XM_001495026 // LOC100063983 // transmembrane protein 223-like // --- // 100063983 ///	LOC100063983	Cluster0002
15001610	ENSECAT00000026211 // LOC100063989 // ELL-associated factor 1-like // --- // 100063989	LOC100063989	Cluster0002
15019080	ENSECAT00000010036 // LOC100064074 // peroxisome biogenesis factor 10-like // --- // 10	LOC100064074	Cluster0002
15051507	XM_001495095 // LOC100064095 // tripartite motif-containing protein 14-like // --- // 1	LOC100064095	Cluster0002
15083565	ENSECAT00000022109 // LOC100064100 // transmembrane and ubiquitin-like domain-containin	LOC100064100	Cluster0002
15086128	XM_001495102 // LOC100064105 // lysosomal protein NCU-G1-like // --- // 100064105 /// E	LOC100064105	Cluster0002
15028300	ENSECAT00000025946 // LOC100064123 // uncharacterized protein C6orf89 homolog // --- //	LOC100064123	Cluster0002
15102337	ENSECAT00000024651 // LOC100064131 // ATP synthase lipid-binding protein, mitochondrial	LOC100064131	Cluster0002
14959667	XM_001495148 // LOC100064169 // dephospho-CoA kinase domain-containing protein-like //	LOC100064169	Cluster0002
15051495	ENSECAT00000011518 // LOC100064200 // uncharacterized LOC100064200 // --- // 100064200	LOC100064200	Cluster0002

14949911	XM_001497212 // LOC100064307 // eukaryotic translation initiation factor 3 subunit K-li	LOC100064307	Cluster0002
15105373	XM_001505079 // LOC100064327 // etoposide-induced protein 2.4 homolog // --- // 1000643	LOC100064327	Cluster0002
15105916	ENSECAT00000023387 // LOC100064330 // UPF0139 membrane protein C19orf56 homolog // ---	LOC100064330	Cluster0002
15122935	ENSECAT00000001401 // LOC100064332 // COMM domain-containing protein 5-like // --- // 1	LOC100064332	Cluster0002
14949941	ENSECAT00000015087 // LOC100064368 // NF-kappa-B inhibitor beta-like // --- // 10006436	LOC100064368	Cluster0002
14949948	XM_001497611 // LOC100064399 // 28S ribosomal protein S12, mitochondrial-like // --- //	LOC100064399	Cluster0002
14954018	XM_001497889 // LOC100064427 // glia maturation factor gamma-like // --- // 100064427 /	LOC100064427	Cluster0002
15083640	XM_001495351 // LOC100064440 // GTP-binding protein Rheb-like // --- // 100064440 /// E	LOC100064440	Cluster0002
15021233	ENSECAT00000011363 // LOC100064467 // integrator complex subunit 12-like // --- // 1000	LOC100064467	Cluster0002
14959736	ENSECAT00000019216 // LOC100064544 // G patch domain-containing protein 8-like // --- /	LOC100064544	Cluster0002
15097379	XM_001504569 // LOC100064590 // coatomer subunit zeta-1-like // --- // 100064590 /// EN	LOC100064590	Cluster0002
14975774	ENSECAT00000011574 // LOC100064608 // UPF0420 protein C16orf58 homolog // --- // 100064	LOC100064608	Cluster0002
15096349	ENSECAT00000017757 // LOC100064619 // FGFR1 oncogene partner 2 homolog // --- // 100064	LOC100064619	Cluster0002
15125524	XM_001495522 // LOC100064692 // rab-like protein 2A-like // --- // 100064692 /// ENSECA	LOC100064692	Cluster0002
14965438	XM_001495576 // LOC100064778 // transmembrane and ubiquitin-like domain-containing prot	LOC100064778	Cluster0002
15088485	ENSECAT00000020720 // LOC100064813 // guanine nucleotide-binding protein G(I)/G(S)/G(O)	LOC100064813	Cluster0002
15110598	XM_001505113 // LOC100064816 // thymocyte nuclear protein 1-like // --- // 100064816 //	LOC100064816	Cluster0002
15002913	ENSECAT00000008327 // LOC100064836 // klotho-like // --- // 100064836 /// XM_001495612	LOC100064836	Cluster0002
15009997	ENSECAT00000007189 // LOC100064837 // corepressor interacting with RBPJ 1-like // --- /	LOC100064837	Cluster0002
15074810	XM_001495619 // LOC100064841 // guanine nucleotide exchange factor MSS4-like // --- //	LOC100064841	Cluster0002
14950227	ENSECAT00000001571 // LOC100064859 // egl nine homolog 2-like // --- // 100064859 /// X	LOC100064859	Cluster0002
14956026	XM_001495629 // LOC100064860 // charged multivesicular body protein 2a-like // --- // 1	LOC100064860	Cluster0002
15028393	ENSECAT00000018261 // LOC100064913 // AN1-type zinc finger protein 3-like // --- // 100	LOC100064913	Cluster0002
15076905	ENSECAT00000021345 // LOC100064918 // ubiquitin-conjugating enzyme E2 D4-like // --- //	LOC100064918	Cluster0002
14954236	ENSECAT00000026031 // LOC100064997 // exosome complex component RRP46-like // --- // 10	LOC100064997	Cluster0002
14954245	XM_001500372 // LOC100065058 // ATP synthase subunit s-like protein-like // --- // 1000	LOC100065058	Cluster0002
15079026	ENSECAT00000003310 // LOC100065089 // transmembrane protein 140-like // --- // 10006508	LOC100065089	Cluster0002
15091776	XM_001499277 // LOC100065113 // DNA-directed RNA polymerase III subunit RPC3-like // --	LOC100065113	Cluster0002
14998078	ENSECAT00000013925 // LOC100065124 // phakinin-like // --- // 100065124 /// XM_00149581	LOC100065124	Cluster0002
15122776	XM_001495843 // LOC100065171 // repressor of RNA polymerase III transcription MAF1 homo	LOC100065171	Cluster0002
15031437	ENSECAT00000020970 // LOC100065189 // lactoylglutathione lyase-like // --- // 100065189	LOC100065189	Cluster0002
15080829	XM_001915794 // LOC100065195 // transmembrane emp24 domain-containing protein 4-like //	LOC100065195	Cluster0002
15087064	ENSECAT00000002893 // LOC100065233 // peroxisomal membrane protein 11B-like // --- // 1	LOC100065233	Cluster0002
15131927	ENSECAT00000019208 // LOC100065318 // zinc finger C4H2 domain-containing protein-like /	LOC100065318	Cluster0002
14937278	ENSECAT00000018015 // LOC100065359 // ceroid-lipofuscinosis neuronal protein 6 homolog	LOC100065359	Cluster0002
15042284	ENSECAT00000015135 // LOC100065392 // s-methyl-5 -thioadenosine phosphorylase-like // -	LOC100065392	Cluster0002
15123461	XM_001496022 // LOC100065448 // YTH domain family protein 3-like // --- // 100065448 //	LOC100065448	Cluster0002
14975875	XM_001496047 // LOC100065483 // zinc finger protein 764-like // --- // 100065483 /// EN	LOC100065483	Cluster0002
15125086	XM_003365638 // LOC100065550 // poly(U)-binding-splicing factor PUF60-like // --- // 10	LOC100065550	Cluster0002
14977498	XM_001497974 // LOC100065572 // 40S ribosomal protein S2-like // --- // 100065572 /// E	LOC100065572	Cluster0002
14954530	XM_001502481 // LOC100065619 // trafficking protein particle complex subunit 6A-like //	LOC100065619	Cluster0002
14965512	XM_001491967 // LOC100065621 // transmembrane protein 106A-like // --- // 100065621 ///	LOC100065621	Cluster0002
14954569	XM_001502594 // LOC100065660 // DNA excision repair protein ERCC-1-like // --- // 10006	LOC100065660	Cluster0002
14987501	XM_001504690 // LOC100065663 // transcription factor BTF3-like // --- // 100065663 ///	LOC100065663	Cluster0002
14977385	XM_001498749 // LOC100065738 // RNA-binding protein with serine-rich domain 1-like // -	LOC100065738	Cluster0002
14956518	XM_001503761 // LOC100065742 // LYR motif-containing protein 2-like // --- // 100065742	LOC100065742	Cluster0002
14954698	XM_001502832 // LOC100065786 // 40S ribosomal protein S17-like // --- // 100065786 ///	LOC100065786	Cluster0002
15045822	XM_001917419 // LOC100065822 // glutaredoxin-related protein 5, mitochondrial-like // -	LOC100065822	Cluster0002
15021812	XM_001496761 // LOC100065845 // 40S ribosomal protein S8-like // --- // 100065845 /// E	LOC100065845	Cluster0002
14979335	ENSECAT00000012705 // LOC100065899 // CDP-diacylglycerol--inositol 3-phosphatidyltransf	LOC100065899	Cluster0002
15016465	ENSECAT00000026685 // LOC100065903 // transmembrane protein 53-like // --- // 100065903	LOC100065903	Cluster0002
14980449	ENSECAT00000021946 // LOC100065966 // n-acetyltransferase 15-like // --- // 100065966 /	LOC100065966	Cluster0002
14979358	XM_001496402 // LOC100065972 // BTB/POZ domain-containing adapter for CUL3-mediated Rho	LOC100065972	Cluster0002
15069257	ENSECAT00000007216 // LOC100066008 // tubulin polymerization-promoting protein family m	LOC100066008	Cluster0002

15093242	XM_001496470 // LOC100066063 // interferon-induced protein 44-like // --- // 100066063	LOC100066063	Cluster0002
14954850	XM_001503216 // LOC100066095 // alpha-soluble NSF attachment protein-like // --- // 100	LOC100066095	Cluster0002
15107170	ENSECAT00000022428 // LOC100066106 // protein HSPC020 homolog // --- // 100066106 /// X	LOC100066106	Cluster0002
14980398	XM_001502330 // LOC100066114 // heme oxygenase 2-like // --- // 100066114 /// ENSECAT00	LOC100066114	Cluster0002
14956757	ENSECAT00000026298 // LOC100066119 // cyclin-C-like // --- // 100066119 /// XM_00150389	LOC100066119	Cluster0002
15069286	XM_001915863 // LOC100066184 // adrenocortical dysplasia protein homolog // --- // 1000	LOC100066184	Cluster0002
15122687	XM_001496550 // LOC100066193 // lymphocyte antigen 6E-like // --- // 100066193 /// ENSE	LOC100066193	Cluster0002
15048663	XM_001504273 // LOC100066293 // RNA (guanine-9-)-methyltransferase domain-containing pr	LOC100066293	Cluster0002
15112101	XM_001496655 // LOC100066358 // rho-related GTP-binding protein RhoG-like // --- // 100	LOC100066358	Cluster0002
15024680	XM_001496658 // LOC100066364 // ubiquitin-like protein ISG15-like // --- // 100066364 /	LOC100066364	Cluster0002
15090478	XM_001503876 // LOC100066365 // upstream stimulatory factor 1-like // --- // 100066365	LOC100066365	Cluster0002
15004871	ENSECAT00000018650 // LOC100066403 // uncharacterized protein C13orf23-like // --- // 1	LOC100066403	Cluster0002
15124419	ENSECAT00000022595 // LOC100066471 // 39S ribosomal protein L13, mitochondrial-like //	LOC100066471	Cluster0002
14960216	XM_001495869 // LOC100066485 // cytosolic 5 -nucleotidase III-like protein-like // ---	LOC100066485	Cluster0002
14976176	XM_001915877 // LOC100066486 // protein spinster homolog 1-like // --- // 100066486 ///	LOC100066486	Cluster0002
15108997	ENSECAT00000008551 // LOC100066498 // uncharacterized LOC100066498 // --- // 100066498	LOC100066498	Cluster0002
14970059	XM_001915002 // LOC100066506 // probable palmitoyltransferase ZDHHC5-like // --- // 100	LOC100066506	Cluster0002
15113996	ENSECAT00000006341 // LOC100066526 // glycolipid transfer protein-like // --- // 100066	LOC100066526	Cluster0002
14937490	XM_003363589 // LOC100066582 // methionyl-tRNA formyltransferase, mitochondrial-like //	LOC100066582	Cluster0002
14970164	XM_001504696 // LOC100066623 // protein FAM111A-like // --- // 100066623 /// ENSECAT000	LOC100066623	Cluster0002
15085509	ENSECAT00000000479 // LOC100066646 // nitrilase homolog 1-like // --- // 100066646 ///	LOC100066646	Cluster0002
14952767	XM_001504002 // LOC100066652 // sphingomyelin phosphodiesterase 2-like // --- // 100066	LOC100066652	Cluster0002
14932368	ENSECAT00000018606 // LOC100066719 // uncharacterized LOC100066719 // --- // 100066719	LOC100066719	Cluster0002
15118108	XM_001496945 // LOC100066762 // ubiquitin carboxyl-terminal hydrolase 30-like // --- //	LOC100066762	Cluster0002
14988625	XM_001496953 // LOC100066774 // t-cell surface glycoprotein CD8 alpha chain-like // ---	LOC100066774	Cluster0002
15076386	ENSECAT00000000196 // LOC100066798 // syntaxin-11-like // --- // 100066798 /// XM_00150	LOC100066798	Cluster0002
14972800	XM_001498309 // LOC100066866 // ubiquitin/ISG15-conjugating enzyme E2 L6-like // --- //	LOC100066866	Cluster0002
15052833	XM_001500399 // LOC100066906 // probable palmitoyltransferase ZDHHC12-like // --- // 10	LOC100066906	Cluster0002
15036985	XM_001915913 // LOC100066943 // protein FAM113A-like // --- // 100066943 /// ENSECAT000	LOC100066943	Cluster0002
15049937	ENSECAT00000014413 // LOC100066985 // ubiquitin-related modifier 1 homolog // --- // 10	LOC100066985	Cluster0002
14974977	ENSECAT00000012980 // LOC100066997 // zinc finger protein with KRAB and SCAN domains 5-	LOC100066997	Cluster0002
14972810	XM_001498524 // LOC100066998 // mediator of RNA polymerase II transcription subunit 19-	LOC100066998	Cluster0002
14952904	XM_001504125 // LOC100067001 // RWD domain-containing protein 1-like // --- // 10006700	LOC100067001	Cluster0002
15129214	ENSECAT00000012441 // LOC100067023 // transcription elongation factor A protein-like 4-	LOC100067023	Cluster0002
15049888	XM_001501110 // LOC100067056 // UPF0184 protein C9orf16-like // --- // 100067056 /// EN	LOC100067056	Cluster0002
15028695	ENSECAT00000011282 // LOC100067096 // protein canopy homolog 3-like // --- // 100067096	LOC100067096	Cluster0002
15052724	ENSECAT00000019047 // LOC100067121 // alpha-N-acetyl-neuraminyl-2,3-beta-galactosyl-1,3	LOC100067121	Cluster0002
15064988	ENSECAT00000000227 // LOC100067168 // cytochrome b5 type B-like // --- // 100067168 ///	LOC100067168	Cluster0002
14992166	ENSECAT00000004110 // LOC100067210 // u4/U6.U5 tri-snRNP-associated protein 2-like // -	LOC100067210	Cluster0002
14935818	XM_001497324 // LOC100067226 // stAR-related lipid transfer protein 5-like // --- // 10	LOC100067226	Cluster0002
15052542	XM_001502120 // LOC100067317 // proteasome subunit beta type-7-like // --- // 100067317	LOC100067317	Cluster0002
14992193	ENSECAT00000007620 // LOC100067333 // s-adenosylmethionine synthase isoform type-2-like	LOC100067333	Cluster0002
15007540	XM_001917545 // LOC100067359 // pleckstrin homology domain-containing family A member 3	LOC100067359	Cluster0002
15037030	XM_001497436 // LOC100067361 // signal-regulatory protein beta-1 isoform 3-like // ---	LOC100067361	Cluster0002
15008507	XM_001504925 // LOC100067394 // protein FAM168B-like // --- // 100067394 /// ENSECAT000	LOC100067394	Cluster0002
14992226	ENSECAT00000029001 // LOC100067403 // trans-Golgi network integral membrane protein 1-l	LOC100067403	Cluster0002
15022195	ENSECAT00000019067 // LOC100067429 // phosphopantothenate--cysteine ligase-like // ---	LOC100067429	Cluster0002
14992239	XM_001497509 // LOC100067474 // e3 ubiquitin-protein ligase KCMF1-like // --- // 100067	LOC100067474	Cluster0002
15082942	ENSECAT00000019270 // LOC100067491 // solute carrier family 23 member 2-like // --- //	LOC100067491	Cluster0002
14975214	XM_001505065 // LOC100067533 // ribonuclease P protein subunit p20-like // --- // 10006	LOC100067533	Cluster0002
15119990	ENSECAT00000024782 // LOC100067571 // tubulin polyglutamylase complex subunit 2-like //	LOC100067571	Cluster0002
15052407	ENSECAT00000024562 // LOC100067589 // n-acetyllactosaminide alpha-1,3-galactosyltransfe	LOC100067589	Cluster0002
15052383	ENSECAT00000012059 // LOC100067611 // ras-related protein Rab-14-like // --- // 1000676	LOC100067611	Cluster0002
15037048	ENSECAT00000007007 // LOC100067632 // NSFL1 cofactor p47-like // --- // 100067632 /// X	LOC100067632	Cluster0002

15051364	XM_001497667 // LOC100067699 // uncharacterized LOC100067699 // --- // 100067699 /// EN	LOC100067699	Cluster0002
15099520	XM_001497705 // LOC100067736 // serine/threonine-protein kinase 25-like // --- // 10006	LOC100067736	Cluster0002
15008051	ENSECAT00000026929 // LOC100067782 // aldehyde oxidase-like // --- // 100067782 /// XM_	LOC100067782	Cluster0002
15048497	XM_001914806 // LOC100067794 // cyclic AMP-responsive element-binding protein 3-like //	LOC100067794	Cluster0002
15011105	ENSECAT00000021236 // LOC100067857 // peptidyl-prolyl cis-trans isomerase-like 3-like /	LOC100067857	Cluster0002
15012190	XM_001497843 // LOC100067916 // nmrA-like family domain-containing protein 1-like // --	LOC100067916	Cluster0002
15053355	XM_001495072 // LOC100068036 // endothelial differentiation-related factor 1-like // --	LOC100068036	Cluster0002
14960586	ENSECAT00000009491 // LOC100068111 // post-GPI attachment to proteins factor 3-like //	LOC100068111	Cluster0002
14954133	XM_001498028 // LOC100068145 // flavin reductase-like // --- // 100068145 /// ENSECAT00	LOC100068145	Cluster0002
14977399	XM_001498121 // LOC100068257 // phosphoglycolate phosphatase-like // --- // 100068257 /	LOC100068257	Cluster0002
14950206	XM_001499336 // LOC100068274 // u1 small nuclear ribonucleoprotein A-like // --- // 100	LOC100068274	Cluster0002
14977393	XM_001498154 // LOC100068292 // enoyl-CoA delta isomerase 1, mitochondrial-like // ---	LOC100068292	Cluster0002
14965963	XM_001917560 // LOC100068316 // cyclin-dependent kinase 12-like // --- // 100068316 ///	LOC100068316	Cluster0002
14976380	ENSECAT00000025755 // LOC100068356 // conserved oligomeric Golgi complex subunit 7-like	LOC100068356	Cluster0002
14979627	XM_001501020 // LOC100068373 // dynactin subunit 5-like // --- // 100068373 /// ENSECAT	LOC100068373	Cluster0002
15088736	ENSECAT00000020999 // LOC100068435 // leucine-rich repeat-containing protein 40-like //	LOC100068435	Cluster0002
15108508	XM_001504389 // LOC100068453 // mps one binder kinase activator-like 2A-like // --- //	LOC100068453	Cluster0002
15008377	XM_001498308 // LOC100068470 // elongation factor 1-beta-like // --- // 100068470 /// E	LOC100068470	Cluster0002
14993264	ENSECAT00000007015 // LOC100068479 // rho-related GTP-binding protein RhoQ-like // ---	LOC100068479	Cluster0002
14980561	ENSECAT00000025143 // LOC100068494 // BTB/POZ domain-containing protein KCTD5-like // -	LOC100068494	Cluster0002
15039797	ENSECAT00000009698 // LOC100068515 // p53 and DNA damage-regulated protein 1-like // --	LOC100068515	Cluster0002
15044031	XM_001503698 // LOC100068546 // dynactin subunit 3-like // --- // 100068546 /// ENSECAT	LOC100068546	Cluster0002
15126632	XM_001498387 // LOC100068567 // guanine nucleotide-binding protein subunit alpha-15-lik	LOC100068567	Cluster0002
15078932	ENSECAT00000026029 // LOC100068568 // muskelin-like // --- // 100068568 /// XM_00150099	LOC100068568	Cluster0002
15037237	XM_001498396 // LOC100068573 // transmembrane 9 superfamily member 4-like // --- // 100	LOC100068573	Cluster0002
15033905	ENSECAT00000006417 // LOC100068637 // Golgi phosphoprotein 3-like // --- // 100068637 /	LOC100068637	Cluster0002
14978360	XM_001505047 // LOC100068673 // DNA replication licensing factor MCM7-like // --- // 10	LOC100068673	Cluster0002
15039822	ENSECAT00000007932 // LOC100068709 // COMM domain-containing protein 7-like // --- // 1	LOC100068709	Cluster0002
15065360	XM_001498629 // LOC100068818 // e3 ubiquitin-protein ligase ZNRF1-like // --- // 100068	LOC100068818	Cluster0002
15058853	XM_001498659 // LOC100068847 // f-box only protein 7-like // --- // 100068847 /// ENSEC	LOC100068847	Cluster0002
15103333	ENSECAT00000000406 // LOC100068855 // NADH dehydrogenase [ubiquinone] iron-sulfur prote	LOC100068855	Cluster0002
14984579	ENSECAT00000022244 // LOC100068903 // HIG1 domain family member 2A-like // --- // 10006	LOC100068903	Cluster0002
15016763	ENSECAT00000016449 // LOC100068904 // c-Myc-binding protein-like // --- // 100068904 //	LOC100068904	Cluster0002
14980512	XM_001499647 // LOC100068915 // THO complex subunit 6 homolog // --- // 100068915 /// E	LOC100068915	Cluster0002
15053235	ENSECAT00000000831 // LOC100069029 // ficolin-1-like // --- // 100069029 /// XM_0014988	LOC100069029	Cluster0002
15058897	ENSECAT00000001574 // LOC100069058 // heme oxygenase 1-like // --- // 100069058 /// XM_	LOC100069058	Cluster0002
14988918	XM_001498907 // LOC100069090 // 39S ribosomal protein L53, mitochondrial-like // --- //	LOC100069090	Cluster0002
15016893	ENSECAT00000024079 // LOC100069157 // chromatin modification-related protein MEAF6-like	LOC100069157	Cluster0002
14969905	ENSECAT00000000764 // LOC100069180 // olfactory receptor 5D18-like // --- // 100069180	LOC100069180	Cluster0002
14946599	ENSECAT00000001594 // LOC100069185 // GPI mannosyltransferase 3-like // --- // 10006918	LOC100069185	Cluster0002
15037460	XM_001499007 // LOC100069227 // charged multivesicular body protein 4b-like // --- // 1	LOC100069227	Cluster0002
15006078	ENSECAT00000016185 // LOC100069263 // ras GTPase-activating protein 3-like // --- // 10	LOC100069263	Cluster0002
14960864	ENSECAT00000007817 // LOC100069329 // nuclear factor erythroid 2-related factor 1-like	LOC100069329	Cluster0002
15037497	XM_001499130 // LOC100069352 // dynein light chain roadblock-type 1-like // --- // 1000	LOC100069352	Cluster0002
15060838	ENSECAT00000014581 // LOC100069490 // thioredoxin, mitochondrial-like // --- // 1000694	LOC100069490	Cluster0002
14960892	XM_001499269 // LOC100069523 // ATP synthase lipid-binding protein, mitochondrial-like	LOC100069523	Cluster0002
14960897	ENSECAT00000022608 // LOC100069535 // ubiquitin-conjugating enzyme E2 Z-like // --- //	LOC100069535	Cluster0002
15108410	XM_001499364 // LOC100069627 // methyl-CpG-binding domain protein 3-like // --- // 1000	LOC100069627	Cluster0002
14941856	XM_003363424 // LOC100069640 // glutathione S-transferase omega-1-like // --- // 100069	LOC100069640	Cluster0002
15037687	ENSECAT00000009144 // LOC100069675 // uncharacterized protein C20orf4 homolog // --- //	LOC100069675	Cluster0002
15014592	ENSECAT00000000384 // LOC100069731 // leucine-rich repeat-containing protein 33-like //	LOC100069731	Cluster0002
14966218	XM_001502482 // LOC100069752 // solute carrier family 35 member B1-like // --- // 10006	LOC100069752	Cluster0002
14984662	XM_001502922 // LOC100069785 // v-type proton ATPase subunit e 1-like // --- // 1000697	LOC100069785	Cluster0002
14980378	ENSECAT00000011648 // LOC100069818 // e3 ubiquitin-protein ligase MGRN1-like // --- //	LOC100069818	Cluster0002

14984671	ENSECAT00000007756 // LOC100069822 // endoplasmic reticulum-Golgi intermediate compartm	LOC100069822	Cluster0002
15112364	XM_001918081 // LOC100070052 // ribosomal RNA-processing protein 8-like // --- // 10007	LOC100070052	Cluster0002
15050148	XM_001500126 // LOC100070077 // dolichyldiphosphatase 1-like // --- // 100070077 /// EN	LOC100070077	Cluster0002
15112372	XM_001499761 // LOC100070082 // transcription initiation factor TFIID subunit 10-like /	LOC100070082	Cluster0002
15017200	ENSECAT00000015186 // LOC100070089 // transmembrane protein 54-like // --- // 100070089	LOC100070089	Cluster0002
15052879	XM_001500252 // LOC100070129 // dolichol kinase-like // --- // 100070129 /// ENSECAT000	LOC100070129	Cluster0002
15032662	ENSECAT00000010523 // LOC100070171 // multivesicular body subunit 12A-like // --- // 10	LOC100070171	Cluster0002
15069958	ENSECAT00000008862 // LOC100070186 // TLD domain-containing protein KIAA1609-like // --	LOC100070186	Cluster0002
14961180	ENSECAT00000008650 // LOC100070268 // u3 small nucleolar RNA-associated protein 18 homo	LOC100070268	Cluster0002
15052731	XM_001500031 // LOC100070379 // uncharacterized LOC100070379 // --- // 100070379 /// EN	LOC100070379	Cluster0002
15052677	ENSECAT00000015249 // LOC100070462 // prosalusin-like // --- // 100070462 /// XM_001501	LOC100070462	Cluster0002
15012747	XM_001500147 // LOC100070489 // 3-hydroxyacyl-CoA dehydratase 2-like // --- // 10007048	LOC100070489	Cluster0002
15049781	XM_001500206 // LOC100070546 // zinc finger protein 79-like // --- // 100070546 /// ENS	LOC100070546	Cluster0002
14966391	XM_001500221 // LOC100070559 // monocyte to macrophage differentiation protein-like //	LOC100070559	Cluster0002
14993709	ENSECAT00000023065 // LOC100070614 // zinc transporter 6-like // --- // 100070614 /// X	LOC100070614	Cluster0002
14946985	ENSECAT00000017166 // LOC100070663 // protein CASC4-like // --- // 100070663 /// XM_001	LOC100070663	Cluster0002
14950603	XM_001916978 // LOC100070664 // transcription factor RelB-like // --- // 100070664 ///	LOC100070664	Cluster0002
15061257	ENSECAT00000012509 // LOC100070677 // PPPDE peptidase domain-containing protein 2-like	LOC100070677	Cluster0002
15017453	XM_001500352 // LOC100070682 // transcription initiation factor TFIID subunit 12-like /	LOC100070682	Cluster0002
14942385	ENSECAT00000009383 // LOC100070708 // ubiquitin domain-containing protein 1-like // ---	LOC100070708	Cluster0002
14966428	XM_001500440 // LOC100070771 // 28S ribosomal protein S23, mitochondrial-like // --- //	LOC100070771	Cluster0002
14966443	XM_001500508 // LOC100070832 // vascular endothelial zinc finger 1-like // --- // 10007	LOC100070832	Cluster0002
15017504	XM_001500542 // LOC100070860 // interferon alpha-inducible protein 6-like // --- // 100	LOC100070860	Cluster0002
15032815	XM_001500556 // LOC100070878 // gamma-interferon-inducible-lysosomal thiol reductase-li	LOC100070878	Cluster0002
15032822	XM_003363830 // LOC100070886 // mpv17-like protein 2-like // --- // 100070886 /// ENSEC	LOC100070886	Cluster0002
15049659	ENSECAT00000022973 // LOC100070894 // rab9 effector protein with kelch motifs-like // -	LOC100070894	Cluster0002
15038156	ENSECAT00000022597 // LOC100070943 // protein SYS1 homolog // --- // 100070943 /// XM_0	LOC100070943	Cluster0002
15052572	XM_001502048 // LOC100070944 // 60S ribosomal protein L35-like // --- // 100070944 ///	LOC100070944	Cluster0002
15017608	XM_001504054 // LOC100070998 // GPN-loop GTPase 2-like // --- // 100070998 /// ENSECAT0	LOC100070998	Cluster0002
15061338	XM_001503005 // LOC100071003 // NFAT activation molecule 1-like // --- // 100071003 ///	LOC100071003	Cluster0002
15107646	XM_001504903 // LOC100071004 // 60S ribosomal protein L27a-like // --- // 100071004 ///	LOC100071004	Cluster0002
15061344	XM_001503030 // LOC100071022 // ribosomal RNA-processing protein 7 homolog A-like // --	LOC100071022	Cluster0002
14993835	ENSECAT00000016734 // LOC100071067 // serine/threonine-protein phosphatase PP1-beta cat	LOC100071067	Cluster0002
15059496	XM_001503143 // LOC100071110 // translocator protein-like // --- // 100071110 /// ENSEC	LOC100071110	Cluster0002
15034884	XM_001503445 // LOC100071132 // protein MEF2BNB-like // --- // 100071132 /// ENSECAT000	LOC100071132	Cluster0002
14950799	ENSECAT00000023978 // LOC100071150 // calmodulin-like // --- // 100071150 /// ENSECAT00	LOC100071150	Cluster0002
14954752	XM_001917113 // LOC100071175 // striatin-4-like // --- // 100071175 /// ENSECAT00000003	LOC100071175	Cluster0002
15052467	ENSECAT00000022631 // LOC100071180 // phosducin-like protein-like // --- // 100071180 /	LOC100071180	Cluster0002
15049529	XM_001500963 // LOC100071258 // olfactory receptor 1L6-like // --- // 100071258 /// ENS	LOC100071258	Cluster0002
15023253	XM_001504118 // LOC100071263 // protein FAM54B-like // --- // 100071263 /// ENSECAT0000	LOC100071263	Cluster0002
15104851	XM_001503069 // LOC100071306 // trafficking protein particle complex subunit 4-like //	LOC100071306	Cluster0002
15104874	ENSECAT00000019023 // LOC100071339 // porphobilinogen deaminase-like // --- // 10007133	LOC100071339	Cluster0002
14939190	ENSECAT00000026484 // LOC100071368 // uncharacterized protein C15orf57-like // --- // 1	LOC100071368	Cluster0002
14961618	XM_001501168 // LOC100071416 // dehydrogenase/reductase SDR family member 11-like // --	LOC100071416	Cluster0002
15038451	XM_001501211 // LOC100071447 // RING finger protein 114-like // --- // 100071447 /// EN	LOC100071447	Cluster0002
15023321	ENSECAT00000017238 // LOC100071457 // chloride intracellular channel protein 4-like //	LOC100071457	Cluster0002
14982073	ENSECAT00000013782 // LOC100071499 // copper transport protein ATOX1-like // --- // 100	LOC100071499	Cluster0002
15052443	XM_001501351 // LOC100071560 // olfactory receptor 1J1-like // --- // 100071560 /// ENS	LOC100071560	Cluster0002
15049460	ENSECAT00000027081 // LOC100071623 // ribosome-recycling factor, mitochondrial-like //	LOC100071623	Cluster0002
14982212	XM_001503714 // LOC100071636 // 40S ribosomal protein S14-like // --- // 100071636 ///	LOC100071636	Cluster0002
15128873	ENSECAT00000002701 // LOC100071709 // peroxiredoxin-1-like // --- // 100071709 /// XM_0	LOC100071709	Cluster0002
14967022	XM_001918320 // LOC100071770 // 26S proteasome non-ATPase regulatory subunit 11-like //	LOC100071770	Cluster0002
15052312	XM_001501657 // LOC100071800 // 26S proteasome non-ATPase regulatory subunit 5-like //	LOC100071800	Cluster0002
14990841	ENSECAT00000015437 // LOC100071892 // syndecan-1-like // --- // 100071892 /// XM_001501	LOC100071892	Cluster0002

14939484	XM_001501844 // LOC100071996 // olfactory receptor 4F21-like // --- // 100071996 /// EN	LOC100071996	Cluster0002
14967266	ENSECAT00000016620 // LOC100072041 // homeobox protein SEBOX-like // --- // 100072041 /	LOC100072041	Cluster0002
15067826	ENSECAT00000002179 // LOC100072076 // 60S ribosomal protein L23a-like // --- // 1000720	LOC100072076	Cluster0002
14934214	XM_001503361 // LOC100072162 // CDGSH iron-sulfur domain-containing protein 1-like // -	LOC100072162	Cluster0002
15015343	ENSECAT00000021973 // LOC100072249 // protein TFG-like // --- // 100072249 /// XM_00150	LOC100072249	Cluster0002
15128880	XM_001502247 // LOC100072334 // apolipoprotein O-like // --- // 100072334 /// ENSECAT00	LOC100072334	Cluster0002
14994472	XM_001502263 // LOC100072348 // PQ-loop repeat-containing protein 3-like // --- // 1000	LOC100072348	Cluster0002
14982649	XM_001502340 // LOC100072416 // steroid receptor RNA activator 1-like // --- // 1000724	LOC100072416	Cluster0002
14939620	XM_001505131 // LOC100072588 // DNA-(apurinic or apyrimidinic site) lyase-like // --- /	LOC100072588	Cluster0002
15132294	ENSECAT00000010652 // LOC100072650 // transcription initiation factor TFIID subunit 9B-	LOC100072650	Cluster0002
14939663	ENSECAT00000014270 // LOC100072699 // methyltransferase-like protein 17, mitochondrial-	LOC100072699	Cluster0002
14968159	ENSECAT00000026936 // LOC100072746 // XIAP-associated factor 1-like // --- // 100072746	LOC100072746	Cluster0002
14943332	XM_001502798 // LOC100072758 // uncharacterized LOC100072758 // --- // 100072758 /// EN	LOC100072758	Cluster0002
14934642	XM_001502808 // LOC100072768 // equilibrative nucleoside transporter 3-like // --- // 1	LOC100072768	Cluster0002
14983003	ENSECAT00000018154 // LOC100072795 // keratinocyte-associated transmembrane protein 2-l	LOC100072795	Cluster0002
14943366	ENSECAT00000018296 // LOC100072800 // activating signal cointegrator 1 complex subunit	LOC100072800	Cluster0002
15026392	XM_001502923 // LOC100072864 // coiled-coil domain-containing protein 109B-like // ---	LOC100072864	Cluster0002
14962748	XM_001503003 // LOC100072920 // chromatin complexes subunit BAP18-like // --- // 100072	LOC100072920	Cluster0002
14934962	ENSECAT00000009285 // LOC100073016 // peptidyl-prolyl cis-trans isomerase F, mitochondr	LOC100073016	Cluster0002
14968611	XM_001503188 // LOC100073047 // vesicle-associated membrane protein 2-like // --- // 10	LOC100073047	Cluster0002
15125887	XM_001914994 // LOC100073052 // glioma tumor suppressor candidate region gene 2 protein	LOC100073052	Cluster0002
14986447	ENSECAT00000025231 // LOC100073132 // tumor necrosis factor alpha-induced protein 8-lik	LOC100073132	Cluster0002
14957654	XM_003362372 // LOC100073151 // serine/threonine-protein kinase Sgk1-like // --- // 100	LOC100073151	Cluster0002
14986603	XM_001503550 // LOC100073184 // signal recognition particle 19 kDa protein-like // ---	LOC100073184	Cluster0002
14957828	ENSECAT00000017544 // LOC100073201 // ralBP1-associated Eps domain-containing protein 1	LOC100073201	Cluster0002
15111351	XM_001917013 // LOC100146180 // ras-related protein Rab-11B-like // --- // 100146180 //	LOC100146180	Cluster0002
14954417	ENSECAT00000011285 // LOC100146195 // protein ETHE1, mitochondrial-like // --- // 10014	LOC100146195	Cluster0002
15108701	ENSECAT00000023579 // LOC100146236 // UBX domain-containing protein 6-like // --- // 10	LOC100146236	Cluster0002
15087565	ENSECAT00000010529 // LOC100146263 // rho-related GTP-binding protein RhoC-like // ---	LOC100146263	Cluster0002
15050999	ENSECAT00000009587 // LOC100146303 // uncharacterized protein C9orf142-like // --- // 1	LOC100146303	Cluster0002
15061672	ENSECAT00000013531 // LOC100146361 // histone deacetylase 10-like // --- // 100146361	LOC100146361	Cluster0002
15106505	XM_001916994 // LOC100146400 // zinc finger protein 414-like // --- // 100146400 /// EN	LOC100146400	Cluster0002
15069524	XM_001916072 // LOC100146422 // IST1 homolog // --- // 100146422 /// ENSECAT00000024550	LOC100146422	Cluster0002
15044313	ENSECAT00000004127 // LOC100146457 // f-box only protein 34-like // --- // 100146457	LOC100146457	Cluster0002
14974056	XM_001917559 // LOC100146465 // cyclin-dependent kinase 2-associated protein 2-like //	LOC100146465	Cluster0002
15038789	ENSECAT00000028942 // LOC100146483 // opioid growth factor receptor-like // --- // 1001	LOC100146483	Cluster0002
15091026	ENSECAT00000006190 // LOC100146589 // polyamine-modulated factor 1-like // --- // 10014	LOC100146589	Cluster0002
15110764	ENSECAT00000026537 // LOC100146594 // UV excision repair protein RAD23 homolog A-like /	LOC100146594	Cluster0002
14953972	XM_001916350 // LOC100146611 // NAD-dependent deacetylase sirtuin-2-like // --- // 1001	LOC100146611	Cluster0002
15061614	ENSECAT00000025871 // LOC100146652 // dolichyl-P-Man:Man(7)GlcNAc(2)-PP-dolichyl-alpha-	LOC100146652	Cluster0002
15032778	ENSECAT00000008180 // LOC100146664 // microtubule-associated serine/threonine-protein k	LOC100146664	Cluster0002
15122768	ENSECAT00000021923 // LOC100146668 // cytochrome c1, heme protein, mitochondrial-like /	LOC100146668	Cluster0002
15038569	XM_001914850 // LOC100146683 // RNA-binding protein 38-like // --- // 100146683 /// ENS	LOC100146683	Cluster0002
14962809	XM_001918138 // LOC100146743 // eukaryotic translation initiation factor 5A-1-like // -	LOC100146743	Cluster0002
14984536	ENSECAT00000023790 // LOC100146751 // tetraspanin-17-like // --- // 100146751 /// XM_00	LOC100146751	Cluster0002
14971846	XM_001917602 // LOC100146758 // patatin-like phospholipase domain-containing protein 2-	LOC100146758	Cluster0002
14970550	XM_001916375 // LOC100146759 // tetratricopeptide repeat protein 9C-like // --- // 1001	LOC100146759	Cluster0002
14998654	ENSECAT00000017231 // LOC100146781 // transmembrane protein adipocyte-associated 1-like	LOC100146781	Cluster0002
15110820	XM_001915055 // LOC100146801 // transcription factor jun-B-like // --- // 100146801 ///	LOC100146801	Cluster0002
15125865	XM_001914701 // LOC100146886 // RING finger protein 126-like // --- // 100146886 /// EN	LOC100146886	Cluster0002
15026845	XM_001915240 // LOC100146898 // nucleolar protein 7-like // --- // 100146898 /// ENSECA	LOC100146898	Cluster0002
14954767	XM_001917363 // LOC100146913 // neutral amino acid transporter B(0)-like // --- // 1001	LOC100146913	Cluster0002
14967510	ENSECAT00000009742 // LOC100146940 // ARF GTPase-activating protein GIT1-like // --- //	LOC100146940	Cluster0002
14977731	XM_001915361 // LOC100147047 // jmjC domain-containing protein 8-like // --- // 1001470	LOC100147047	Cluster0002

15058963	XM_001916763 // LOC100147078 // ADP-ribosylation factor-binding protein GGA1-like // --	LOC100147078	Cluster0002
15032736	XM_001915146 // LOC100147159 // 60S ribosomal protein L18a-like // --- // 100147159 ///	LOC100147159	Cluster0002
14972597	XM_001915957 // LOC100147187 // olfactory receptor 8U8-like // --- // 100147187 /// ENS	LOC100147187	Cluster0002
15051055	XM_001917520 // LOC100147202 // Sjoegren syndrome nuclear autoantigen 1 homolog // ---	LOC100147202	Cluster0002
15000138	ENSECAT00000014540 // LOC100147234 // testis-expressed sequence 264 protein-like // ---	LOC100147234	Cluster0002
15032479	ENSECAT00000006719 // LOC100147248 // ras-related protein Rab-8A-like // --- // 1001472	LOC100147248	Cluster0002
15125203	ENSECAT00000016258 // LOC100147359 // diacylglycerol O-acyltransferase 1-like // --- //	LOC100147359	Cluster0002
14962744	XM_001918130 // LOC100147426 // ribonuclease kappa-like // --- // 100147426 /// ENSECAT	LOC100147426	Cluster0002
15119976	XM_001916074 // LOC100147463 // zinc transporter ZIP6-like // --- // 100147463 /// ENSE	LOC100147463	Cluster0002
15053532	XM_001917548 // LOC100147491 // WD repeat-containing protein 85-like // --- // 10014749	LOC100147491	Cluster0002
14960985	XM_001917680 // LOC100147514 // pyruvate dehydrogenase [lipoamide] kinase isozyme 2-lik	LOC100147514	Cluster0002
15094181	ENSECAT00000014590 // LOC100147528 // protein FAM134A-like // --- // 100147528 /// XM_0	LOC100147528	Cluster0002
15126505	XM_001915070 // LOC100147535 // lymphocyte-specific protein 1-like // --- // 100147535	LOC100147535	Cluster0002
15065419	XM_001916328 // LOC100147597 // vacuolar fusion protein MON1 homolog B-like // --- // 1	LOC100147597	Cluster0002
14965865	XM_001918070 // LOC100147612 // 26S proteasome non-ATPase regulatory subunit 3-like //	LOC100147612	Cluster0002
14958026	ENSECAT00000018452 // LOC100147615 // transcription factor MafG-like // --- // 10014761	LOC100147615	Cluster0002
15126748	XM_001916534 // LOC100147619 // midnolin-like // --- // 100147619 /// ENSECAT0000000825	LOC100147619	Cluster0002
15125800	XM_001916676 // LOC100147630 // DDB1- and CUL4-associated factor 15-like // --- // 1001	LOC100147630	Cluster0002
15034418	ENSECAT00000018663 // LOC100147631 // acetolactate synthase-like protein-like // --- //	LOC100147631	Cluster0002
15126700	ENSECAT00000022388 // LOC100147642 // mediator of RNA polymerase II transcription subun	LOC100147642	Cluster0002
14990246	XM_001917878 // LOC100147678 // protein MEMO1-like // --- // 100147678 /// ENSECAT00000	LOC100147678	Cluster0002
14989780	XM_003362994 // LOC100629145 // calmodulin-like // --- // 100629145 /// ENSECAT00000008	LOC100629145	Cluster0002
15089804	XM_003364942 // LOC100629233 // uncharacterized LOC100629233 // --- // 100629233 /// EN	LOC100629233	Cluster0002
15028080	XM_003363770 // LOC100629238 // high mobility group protein HMG-I/HMG-Y-like // --- //	LOC100629238	Cluster0002
15007979	ENSECAT00000028818 // LOC100629260 // 20 kDa chaperonin, chloroplastic-like // --- // 1	LOC100629260	Cluster0002
15104419	ENSECAT00000012267 // LOC100629277 // UPF0686 protein C11orf1 homolog // --- // 1006292	LOC100629277	Cluster0002
15111033	ENSECAT00000021186 // LOC100629291 // protein HIDE1-like // --- // 100629291 /// XM_003	LOC100629291	Cluster0002
15124038	XM_003365653 // LOC100629307 // zinc finger protein 706-like // --- // 100629307 /// EN	LOC100629307	Cluster0002
15086861	XM_003365033 // LOC100629318 // CDC42 small effector protein 1-like // --- // 100629318	LOC100629318	Cluster0002
15079262	XM_003364867 // LOC100629380 // single-stranded DNA-binding protein, mitochondrial-like	LOC100629380	Cluster0002
15019185	XM_003364468 // LOC100629488 // uncharacterized LOC100629488 // --- // 100629488 /// EN	LOC100629488	Cluster0002
15066373	ENSECAT00000012525 // LOC100629544 // uncharacterized protein C4orf36-like // --- // 10	LOC100629544	Cluster0002
15025697	XM_003364506 // LOC100629585 // u6 snRNA-associated Sm-like protein LSm6-like // --- //	LOC100629585	Cluster0002
15075834	ENSECAT00000021493 // LOC100629586 // brain protein 44-like protein-like // --- // 1006	LOC100629586	Cluster0002
15046503	ENSECAT00000008215 // LOC100629687 // inverted formin-2-like // --- // 100629687	LOC100629687	Cluster0002
15031219	ENSECAT00000005038 // LOC100629770 // uncharacterized protein C3orf18-like // --- // 10	LOC100629770	Cluster0002
15096334	ENSECAT00000019669 // LOC100629790 // LYR motif-containing protein 5-like // --- // 100	LOC100629790	Cluster0002
15027038	ENSECAT00000021472 // LOC100629805 // acyl-coenzyme A thioesterase 13-like // --- // 10	LOC100629805	Cluster0002
15064514	XM_003364593 // LOC100629814 // CKLF-like MARVEL transmembrane domain-containing protei	LOC100629814	Cluster0002
14939658	ENSECAT00000012091 // LOC100629897 // ribonuclease K6-like // --- // 100629897 /// XM_0	LOC100629897	Cluster0002
15100413	XM_003365170 // LOC100629907 // c-type lectin domain family 2 member B-like // --- // 1	LOC100629907	Cluster0002
15052920	XM_003364107 // LOC100629960 // torsin-1A-like // --- // 100629960 /// ENSECAT000000147	LOC100629960	Cluster0002
15027612	ENSECAT00000011113 // LOC100629969 // leukocyte-specific transcript 1 protein-like // -	LOC100629969	Cluster0002
15113545	XM_003365481 // LOC100630004 // ubiquitin-conjugating enzyme E2 L3-like // --- // 10063	LOC100630004	Cluster0002
15055592	XM_003364189 // LOC100630015 // uncharacterized LOC100630015 // --- // 100630015 /// EN	LOC100630015	Cluster0002
14952468	XM_003362348 // LOC100630070 // proline-rich nuclear receptor coactivator 1-like // ---	LOC100630070	Cluster0002
15120823	ENSECAT00000014301 // LOC100630092 // uncharacterized protein C8orf59 homolog // --- //	LOC100630092	Cluster0002
14956129	ENSECAT00000012977 // LOC100630209 // cytochrome c oxidase subunit 7A2, mitochondrial-l	LOC100630209	Cluster0002
14992425	XM_003362965 // LOC100630257 // WW domain-binding protein 1-like // --- // 100630257 //	LOC100630257	Cluster0002
14999157	XM_003363036 // LOC100630258 // actin-related protein 2/3 complex subunit 4-like // ---	LOC100630258	Cluster0002
14947684	XM_003363530 // LOC100630310 // H/ACA ribonucleoprotein complex subunit 3-like // --- /	LOC100630310	Cluster0002
15045955	XM_003364025 // LOC100630389 // transcriptional repressor protein YY1-like // --- // 10	LOC100630389	Cluster0002
15066529	XM_003364673 // LOC100630403 // placenta-specific gene 8 protein-like // --- // 1006304	LOC100630403	Cluster0002
14951938	XM_003362241 // LOC100630476 // uncharacterized LOC100630476 // --- // 100630476 /// EN	LOC100630476	Cluster0002

15066070	XM_003364657 // LOC100630481 // ubiquitin-conjugating enzyme E2 D3-like // --- // 10063	LOC100630481	Cluster0002
15023382	ENSECAT00000026511 // LOC100630531 // proline-rich nuclear receptor coactivator 2-like	LOC100630531	Cluster0002
14998629	ENSECAT00000001739 // LOC100630536 // small ubiquitin-related modifier 2-like // --- //	LOC100630536	Cluster0002
15025826	XM_003364513 // LOC100630562 // microsomal glutathione S-transferase 2-like // --- // 1	LOC100630562	Cluster0002
15088800	ENSECAT00000014687 // LOC100630574 // guanine nucleotide-binding protein G(I)/G(S)/G(O)	LOC100630574	Cluster0002
15091698	ENSECAT00000013394 // LOC100630631 // uncharacterized protein C1orf54-like // --- // 10	LOC100630631	Cluster0002
14965618	XM_003362480 // LOC100630685 // receptor activity-modifying protein 2-like // --- // 10	LOC100630685	Cluster0002
15042435	XM_003363965 // LOC100630740 // galactose-1-phosphate uridylyltransferase-like // --- /	LOC100630740	Cluster0002
15062811	ENSECAT00000006430 // LOC100630762 // signal peptidase complex subunit 1-like // --- //	LOC100630762	Cluster0002
14949221	XM_003362325 // LOC100630820 // CCAAT/enhancer-binding protein gamma-like // --- // 100	LOC100630820	Cluster0002
15077975	ENSECAT00000020901 // LOC100630867 // coiled-coil domain-containing protein 126-like //	LOC100630867	Cluster0002
15100100	XM_001497952 // LPCAT3 // lysophosphatidylcholine acyltransferase 3 // --- // 100053035	LPCAT3	Cluster0002
15012575	ENSECAT00000025615 // LRCH3 // leucine-rich repeats and calponin homology (CH) domain c	LRCH3	Cluster0002
15050083	XM_001500261 // LRRC8A // leucine rich repeat containing 8 family, member A // --- // 1	LRRC8A	Cluster0002
15123056	ENSECAT00000015817 // LRRCC1 // leucine rich repeat and coiled-coil centrosomal protein	LRRCC1	Cluster0002
15060388	XM_001494765 // LTA4H // leukotriene A4 hydrolase // --- // 100052257 /// ENSECAT000000	LTA4H	Cluster0002
15123195	NM_001081898 // LY96 // lymphocyte antigen 96 // --- // 100034040 /// ENSECAT0000001850	LY96	Cluster0002
15108665	ENSECAT00000010103 // MAP2K2 // mitogen-activated protein kinase kinase 2 // --- // ---	MAP2K2	Cluster0002
14965039	ENSECAT00000009384 // MAP3K3 // mitogen-activated protein kinase kinase kinase 3 // ---	MAP3K3	Cluster0002
15062032	XM_001494766 // MAP3K8 // mitogen-activated protein kinase kinase kinase 8 // --- // 10	MAP3K8	Cluster0002
15032956	XM_001503477 // MAU2 // MAU2 chromatid cohesion factor homolog (C. elegans) // --- // 1	MAU2	Cluster0002
15101847	XM_001504207 // MCRS1 // microspherule protein 1 // --- // 100051964 /// XM_001504209 /	MCRS1	Cluster0002
14978700	NM_001195526 // MDH2 // malate dehydrogenase 2, NAD (mitochondrial) // --- // 100061046	MDH2	Cluster0002
14960598	ENSECAT00000009851 // MED1 // mediator complex subunit 1 // --- // 100054967	MED1	Cluster0002
14936372	XM_001489154 // MEF2A // myocyte enhancer factor 2A // --- // 100050068 /// ENSECAT0000	MEF2A	Cluster0002
14975148	XM_001498760 // MEPCE // methylphosphate capping enzyme // --- // 100068939 /// ENSECAT	MEPCE	Cluster0002
14968255	XM_001918365 // MINK1 // misshapen-like kinase 1 // --- // 100061370 /// ENSECAT0000000	MINK1	Cluster0002
15095058	NR_032854 // MIR149 // microRNA mir-149 // --- // 100314817	MIR149	Cluster0002
15089475	NR_032843 // MIR214 // microRNA mir-214 // --- // 100314811	MIR214	Cluster0002
15131506	NR_033079 // MIR221 // microRNA mir-221 // --- // 100314997	MIR221	Cluster0002
15079960	NR_032831 // MIR671 // microRNA mir-671 // --- // 100315004	MIR671	Cluster0002
15016244	ENSECAT00000002623 // MKNK1 // MAP kinase interacting serine/threonine kinase 1 // ---	MKNK1	Cluster0002
15016240	ENSECAT00000025642 // MOB3C // MOB kinase activator 3C // --- // ---	MOB3C	Cluster0002
14996293	XM_001915918 // MON1A // MON1 homolog A (yeast) // --- // 100062456 /// ENSECAT00000025	MON1A	Cluster0002
15054013	XM_001493319 // MORC3 // MORC family CW-type zinc finger 3 // --- // 100061343 /// ENSE	MORC3	Cluster0002
14975199	XM_001505059 // MOSPD3 // motile sperm domain containing 3 // --- // 100067442 /// ENSE	MOSPD3	Cluster0002
15092177	XM_001499121 // MOV10 // Mov10, Moloney leukemia virus 10, homolog (mouse) // --- // 10	MOV10	Cluster0002
14962969	XM_001504782 // MPDU1 // mannose-P-dolichol utilization defect 1 // --- // 100062007 //	MPDU1	Cluster0002
14936472	ENSECAT00000006174 // MPHOSPH10 // M-phase phosphoprotein 10 (U3 small nucleolar ribonu	MPHOSPH10	Cluster0002
14973918	ENSECAT00000014431 // MRPL11 // mitochondrial ribosomal protein L11 // --- // 100052531	MRPL11	Cluster0002
15126359	XM_001915037 // MTA1 // metastasis associated 1 // --- // 100146742 /// ENSECAT00000009	MTA1	Cluster0002
15123896	XM_001490437 // MTERFD1 // MTERF domain containing 1 // --- // 100055471 /// ENSECAT000	MTERFD1	Cluster0002
15063012	ENSECAT00000000008 // MTPAP // mitochondrial poly(A) polymerase // --- // 100055113 ///	MTPAP	Cluster0002
15020172	XM_001498445 // NAF1 // nuclear assembly factor 1 homolog (S. cerevisiae) // --- // 100	NAF1	Cluster0002
15061278	ENSECAT00000012021 // NAGA // N-acetylgalactosaminidase, alpha- // --- // 100070793 ///	NAGA	Cluster0002
14965682	XM_001917361 // NAGLU // N-acetylglucosaminidase, alpha // --- // 100066052 /// ENSECAT	NAGLU	Cluster0002
15038323	ENSECAT00000022749 // NCOA3 // nuclear receptor coactivator 3 // --- // 100071296 /// X	NCOA3	Cluster0002
14976166	ENSECAT00000010865 // NFATC2IP // nuclear factor of activated T-cells, cytoplasmic, cal	NFATC2IP	Cluster0002
15040133	XM_001501792 // NFS1 // NFS1 nitrogen fixation 1 homolog (S. cerevisiae) // --- // 1000	NFS1	Cluster0002
15022238	ENSECAT00000002155 // NFYC // nuclear transcription factor Y, gamma // --- // 100053895	NFYC	Cluster0002
15023353	ENSECAT00000024594 // NIPAL3 // NIPA-like domain containing 3 // --- // 100071495 /// X	NIPAL3	Cluster0002
15043924	XM_001499370 // NOL6 // nucleolar protein family 6 (RNA-associated) // --- // 100053901	NOL6	Cluster0002
15039632	XM_001497181 // NOP56 // NOP56 ribonucleoprotein homolog (yeast) // --- // 100067054 //	NOP56	Cluster0002
14977797	ENSECAT00000018658 // NPRL3 // nitrogen permease regulator-like 3 (S. cerevisiae) // --	NPRL3	Cluster0002

14993989	ENSECAT00000008586 // NRBP1 // nuclear receptor binding protein 1 // --- // 100055145 /	NRBP1	Cluster0002
15130299	ENSECAT00000002465 // NSDHL // NAD(P) dependent steroid dehydrogenase-like // --- // 10	NSDHL	Cluster0002
15050413	XM_001917273 // NUP214 // nucleoporin 214kDa // --- // 100069634 /// ENSECAT00000024446	NUP214	Cluster0002
14962611	ENSECAT00000013793 // NUP88 // nucleoporin 88kDa // --- // 100061117 /// XM_001504741 /	NUP88	Cluster0002
15114137	NM_001081797 // OASL // 2 -5 -oligoadenylate synthetase-like // --- // 100009702 /// EN	OASL	Cluster0002
15021553	XM_001491520 // OSBPL9 // oxysterol binding protein-like 9 // --- // 100051050 /// ENSE	OSBPL9	Cluster0002
15065586	ENSECAT00000024631 // OSGIN1 // oxidative stress induced growth inhibitor 1 // --- // -	OSGIN1	Cluster0002
15020540	ENSECAT00000025178 // OTUD4 // OTU domain containing 4 // --- // 100070999 /// XM_00150	OTUD4	Cluster0002
15111981	NM_001257152 // P2RY2 // purinergic receptor P2Y, G-protein coupled, 2 // --- // 100065	P2RY2	Cluster0002
15061389	ENSECAT00000012057 // PACSIN2 // protein kinase C and casein kinase substrate in neuron	PACSIN2	Cluster0002
14977149	ENSECAT00000020560 // PAM16 // presequence translocase-associated motor 16 homolog (S.	PAM16	Cluster0002
14987230	ENSECAT00000013349 // PAPD4 // PAP associated domain containing 4 // --- // 100065390 /	PAPD4	Cluster0002
14971013	ENSECAT00000012180 // PCNXL3 // pecanex-like 3 (Drosophila) // --- // 100057407	PCNXL3	Cluster0002
15109812	XM_001502605 // PCSK7 // proprotein convertase subtilisin/kexin type 7 // --- // 100062	PCSK7	Cluster0002
14999682	ENSECAT00000005601 // PDE12 // phosphodiesterase 12 // --- // 100058532 /// XM_00148997	PDE12	Cluster0002
15097419	XM_001504577 // PDE1B // phosphodiesterase 1B, calmodulin-dependent // --- // 100064711	PDE1B	Cluster0002
14980570	ENSECAT00000002053 // PDPK1 // 3-phosphoinositide dependent protein kinase-1 // --- //	PDPK1	Cluster0002
14962710	ENSECAT00000022417 // PELP1 // proline, glutamate and leucine rich protein 1 // --- //	PELP1	Cluster0002
15107234	ENSECAT00000022985 // PGAP2 // post-GPI attachment to proteins 2 // --- // ---	PGAP2	Cluster0002
15022978	ENSECAT00000024227 // PHACTR4 // phosphatase and actin regulator 4 // --- // 100070745	PHACTR4	Cluster0002
15004990	ENSECAT00000015762 // PHF11 // PHD finger protein 11 // --- // ---	PHF11	Cluster0002
15086809	XM_001492226 // PI4KB // phosphatidylinositol 4-kinase, catalytic, beta // --- // 10005	PI4KB	Cluster0002
14946132	XM_001496440 // PIAS1 // protein inhibitor of activated STAT, 1 // --- // 100052741 ///	PIAS1	Cluster0002
15103566	ENSECAT00000020105 // PIAS4 // protein inhibitor of activated STAT, 4 // --- // 1000613	PIAS4	Cluster0002
15085745	ENSECAT00000004685 // PIGM // phosphatidylinositol glycan anchor biosynthesis, class M	PIGM	Cluster0002
14967293	XM_001504143 // PIGS // phosphatidylinositol glycan anchor biosynthesis, class S // ---	PIGS	Cluster0002
15038166	XM_001503260 // PIGT // phosphatidylinositol glycan anchor biosynthesis, class T // ---	PIGT	Cluster0002
15039937	XM_001501271 // PIGU // phosphatidylinositol glycan anchor biosynthesis, class U // ---	PIGU	Cluster0002
15064847	XM_001498913 // PLA2G15 // phospholipase A2, group XV // --- // 100053908 /// ENSECAT00	PLA2G15	Cluster0002
15118353	ENSECAT00000009632 // PLBD2 // phospholipase B domain containing 2 // --- // 100056432	PLBD2	Cluster0002
14946322	EF397514 // PLEKHO2 // pleckstrin homology domain containing, family O member 2 // ---	PLEKHO2	Cluster0002
14945899	ENSECAT00000006078 // PML // promyelocytic leukemia // --- // 100062744 /// XM_00149324	PML	Cluster0002
14955234	XM_001917356 // PNKP // polynucleotide kinase 3 -phosphatase // --- // 100052218 /// EN	PNKP	Cluster0002
14984074	XM_001504673 // POC5 // POC5 centriolar protein homolog (Chlamydomonas) // --- // 10007	POC5	Cluster0002
14967254	ENSECAT00000014913 // POLDIP2 // polymerase (DNA-directed), delta interacting protein 2	POLDIP2	Cluster0002
15061351	ENSECAT00000019601 // POLDIP3 // polymerase (DNA-directed), delta interacting protein 3	POLDIP3	Cluster0002
14974010	XM_001491904 // PPP1CA // protein phosphatase 1, catalytic subunit, alpha isozyme // --	PPP1CA	Cluster0002
15046204	ENSECAT00000020879 // PPP2R5C // protein phosphatase 2, regulatory subunit B , gamma //	PPP2R5C	Cluster0002
14976018	XM_001501848 // PPP4C // protein phosphatase 4, catalytic subunit // --- // 100064118 /	PPP4C	Cluster0002
14950783	XM_001500787 // PPP5C // protein phosphatase 5, catalytic subunit // --- // 100071104 /	PPP5C	Cluster0002
15049219	XM_001488863 // PRPF4 // PRP4 pre-mRNA processing factor 4 homolog (yeast) // --- // 10	PRPF4	Cluster0002
15045145	ENSECAT00000004545 // PSEN1 // presenilin 1 // --- // ---	PSEN1	Cluster0002
14950066	XM_001498409 // PSMC4 // proteasome (prosome, macropain) 26S subunit, ATPase, 4 // ---	PSMC4	Cluster0002
14940223	ENSECAT00000017709 // PSME1 // proteasome (prosome, macropain) activator subunit 1 (PA2	PSME1	Cluster0002
15080173	ENSECAT00000009183 // PTPN12 // protein tyrosine phosphatase, non-receptor type 12 // -	PTPN12	Cluster0002
15006122	XM_001504923 // PTPN18 // protein tyrosine phosphatase, non-receptor type 18 (brain-der	PTPN18	Cluster0002
14941039	ENSECAT00000010165 // PTPRE // protein tyrosine phosphatase, receptor type, E // --- //	PTPRE	Cluster0002
15058834	ENSECAT00000024435 // PWP1 // PWP1 homolog (S. cerevisiae) // --- // ---	PWP1	Cluster0002
15054399	ENSECAT00000007027 // PWP2 // PWP2 periodic tryptophan protein homolog (yeast) // --- /	PWP2	Cluster0002
15108354	ENSECAT00000007668 // QSER1 // glutamine and serine rich 1 // --- // 100057700 /// XM_0	QSER1	Cluster0002
15102967	ENSECAT00000005429 // R3HDM2 // R3H domain containing 2 // --- // 100053125	R3HDM2	Cluster0002
15006508	XM_001489470 // RAB3GAP1 // RAB3 GTPase activating protein subunit 1 (catalytic) // ---	RAB3GAP1	Cluster0002
14987684	ENSECAT00000011814 // RAD17 // RAD17 homolog (S. pombe) // --- // 100065769 /// XM_0015	RAD17	Cluster0002
15049099	XM_001915992 // RAD23B // RAD23 homolog B (S. cerevisiae) // --- // 100059968 /// ENSEC	RAD23B	Cluster0002

15070391	ENSECAT00000022636 // RAP1GDS1 // RAP1, GTP-GDP dissociation stimulator 1 // --- // ---	RAP1GDS1	Cluster0002
15052970	ENSECAT00000020778 // RAPGEF1 // Rap guanine nucleotide exchange factor (GEF) 1 // ---	RAPGEF1	Cluster0002
14948052	XM_001494818 // RBM23 // RNA binding motif protein 23 // --- // 100052786 /// ENSECAT00	RBM23	Cluster0002
14955698	ENSECAT00000024790 // RDH13 // retinol dehydrogenase 13 (all-trans/9-cis) // --- // 100	RDH13	Cluster0002
15048507	ENSECAT00000010431 // RGP1 // RGP1 retrograde golgi transport homolog (S. cerevisiae) /	RGP1	Cluster0002
15039165	ENSECAT00000016226 // RIN2 // Ras and Rab interactor 2 // --- // ---	RIN2	Cluster0002
14998480	ENSECAT00000025072 // RNF13 // ring finger protein 13 // --- // 100058094 /// XM_001491	RNF13	Cluster0002
15021860	ENSECAT00000005223 // RNF220 // ring finger protein 220 // --- // 100052861 /// XM_0019	RNF220	Cluster0002
15098634	XM_001492149 // RNF25 // ring finger protein 25 // --- // 100058790 /// ENSECAT00000009	RNF25	Cluster0002
14940229	XM_001490663 // RNF31 // ring finger protein 31 // --- // 100051661 /// ENSECAT00000015	RNF31	Cluster0002
14966668	ENSECAT00000018593 // RNFT1 // ring finger protein, transmembrane 1 // --- // 100072218	RNFT1	Cluster0002
14977083	ENSECAT00000019560 // ROGDI // rogdi homolog (Drosophila) // --- // ---	ROGDI	Cluster0002
15120210	AY246726 // RPL17 // ribosomal protein L17 // --- // 100034005 /// XM_003365546 // RPL1	RPL17	Cluster0002
14954972	NM_001195513 // RPL18 // ribosomal protein L18 // --- // 100051091 /// ENSECAT000000234	RPL18	Cluster0002
15050562	AF508309 // RPL7A // ribosomal protein L7a // --- // 100033932	RPL7A	Cluster0002
15122937	ENSECAT00000008654 // RPL8 // ribosomal protein L8 // --- // ---	RPL8	Cluster0002
14946059	XM_001495725 // RPLP1 // ribosomal protein, large, P1 // --- // 100052525 /// ENSECAT00	RPLP1	Cluster0002
14971844	NM_001085436 // RPLP2 // ribosomal protein, large, P2 // --- // 100034001	RPLP2	Cluster0002
15097590	NM_001163890 // RPS26 // ribosomal protein S26 // --- // 100034009 /// ENSECAT000000260	RPS26	Cluster0002
14996139	XM_001494739 // RRP9 // ribosomal RNA processing 9, small subunit (SSU) processome comp	RRP9	Cluster0002
15087508	ENSECAT00000008155 // RSBN1 // round spermatid basic protein 1 // --- // ---	RSBN1	Cluster0002
14976858	ENSECAT00000027038 // RSL1D1 // ribosomal L1 domain containing 1 // --- // ---	RSL1D1	Cluster0002
15118784	ENSECAT00000014736 // RSRC2 // arginine/serine-rich coiled-coil 2 // --- // 100058861	RSRC2	Cluster0002
14943204	ENSECAT00000011962 // RUFY2 // RUN and FYVE domain containing 2 // --- // 100063098 ///	RUFY2	Cluster0002
14965557	ENSECAT00000007839 // RUNDC1 // RUN domain containing 1 // --- // 100065701 /// XM_0014	RUNDC1	Cluster0002
15086701	NM_001163867 // S100A10 // S100 calcium binding protein A10 // --- // 100034012 /// ENS	S100A10	Cluster0002
15006176	XM_003363264 // SAP130 // Sin3A-associated protein, 130kDa // --- // 100067466 /// ENSE	SAP130	Cluster0002
14970973	XM_001490530 // SCYL1 // SCY1-like 1 (S. cerevisiae) // --- // 100057036 /// ENSECAT000	SCYL1	Cluster0002
15089419	ENSECAT00000023819 // SCYL3 // SCY1-like 3 (S. cerevisiae) // --- // ---	SCYL3	Cluster0002
15123517	ENSECAT00000016077 // SDCBP // syndecan binding protein (syntenin) // --- // 100052836	SDCBP	Cluster0002
15117681	ENSECAT00000021479 // SEC14L2 // SEC14-like 2 (S. cerevisiae) // --- // 100058707 /// X	SEC14L2	Cluster0002
14944855	XM_001498879 // SEMA4B // sema domain, immunoglobulin domain (Ig), transmembrane domain	SEMA4B	Cluster0002
15101621	ENSECAT00000011103 // SENP1 // SUMO1/sentrin specific peptidase 1 // --- // 100056855 /	SENP1	Cluster0002
14950873	NM_001170422 // SEPW1 // selenoprotein W, 1 // --- // 100050195 /// ENSECAT00000006991	SEPW1	Cluster0002
14977517	NM_001170424 // SEPX1 // methionine sulfoxide reductase B1 // --- // 100068001 /// ENSE	SEPX1	Cluster0002
15029572	XM_001488303 // SERPINB9 // serpin peptidase inhibitor, clade B (ovalbumin), member 9 /	SERPINB9	Cluster0002
14979164	ENSECAT00000015927 // SETD1A // SET domain containing 1A // --- // 100063523 /// XM_001	SETD1A	Cluster0002
14973574	XM_001490608 // SF1 // splicing factor 1 // --- // 100050420 /// ENSECAT00000026475 //	SF1	Cluster0002
15113729	XM_001498224 // SF3A1 // splicing factor 3a, subunit 1, 120kDa // --- // 100058749 ///	SF3A1	Cluster0002
15103356	ENSECAT00000029107 // SF3A2 // splicing factor 3a, subunit 2, 66kDa // --- // ---	SF3A2	Cluster0002
15052683	ENSECAT00000019146 // SH2D3C // SH2 domain containing 3C // --- // 100070448 /// XM_001	SH2D3C	Cluster0002
15023177	NM_001242455 // SH3BGRL3 // SH3 domain binding glutamic acid-rich protein like 3 // ---	SH3BGRL3	Cluster0002
15072660	ENSECAT00000023523 // SH3BP2 // SH3-domain binding protein 2 // --- // 100051380 /// XM	SH3BP2	Cluster0002
15093066	XM_003365100 // SH3GLB1 // SH3-domain GRB2-like endophilin B1 // --- // 100052441 /// X	SH3GLB1	Cluster0002
15086393	XM_001497785 // SHC1 // SHC (Src homology 2 domain containing) transforming protein 1 /	SHC1	Cluster0002
15104618	XM_001502574 // SIDT2 // SID1 transmembrane family, member 2 // --- // 100062652 /// EN	SIDT2	Cluster0002
15032560	ENSECAT00000008817 // SIN3B // SIN3 transcription regulator homolog B (yeast) // --- //	SIN3B	Cluster0002
14971044	ENSECAT00000013717 // SIPA1 // signal-induced proliferation-associated 1 // --- // ---	SIPA1	Cluster0002
15133955	ENSECAT00000014359 // SLC10A3 // solute carrier family 10 (sodium/bile acid cotransport	SLC10A3	Cluster0002
15055625	XM_001489728 // SLC19A1 // solute carrier family 19 (folate transporter), member 1 // -	SLC19A1	Cluster0002
15035773	XM_001499617 // SLC1A3 // solute carrier family 1 (glial high affinity glutamate transp	SLC1A3	Cluster0002
15049772	XM_001500215 // SLC2A8 // solute carrier family 2 (facilitated glucose transporter), me	SLC2A8	Cluster0002
15031770	XM_001497724 // SLC35B2 // solute carrier family 35, member B2 // --- // 100067750 ///	SLC35B2	Cluster0002
15044573	ENSECAT00000005976 // SLC38A6 // solute carrier family 38, member 6 // --- // 100060809	SLC38A6	Cluster0002

14958923	ENSECAT00000023379 // SLC39A11 // solute carrier family 39 (metal ion transporter), mem	SLC39A11	Cluster0002
14970579	XM_001495061 // SLC3A2 // solute carrier family 3 (activators of dibasic and neutral am	SLC3A2	Cluster0002
14967281	ENSECAT00000006927 // SLC46A1 // solute carrier family 46 (folate transporter), member	SLC46A1	Cluster0002
15113466	XM_001489987 // SLC7A4 // solute carrier family 7 (orphan transporter), member 4 // ---	SLC7A4	Cluster0002
15116515	AB076030 // SMAD4 // SMAD family member 4 // --- // 100033846	SMAD4	Cluster0002
14989567	ENSECAT00000014967 // SMEK2 // SMEK homolog 2, suppressor of mek1 (Dictyostelium) // --	SMEK2	Cluster0002
15084979	XM_003364947 // SMG7 // smg-7 homolog, nonsense mediated mRNA decay factor (C. elegans)	SMG7	Cluster0002
14994004	XM_001502317 // SNX17 // sorting nexin 17 // --- // 100055239 /// XM_003363022 // SNX17	SNX17	Cluster0002
14974851	ENSECAT00000025400 // SNX8 // sorting nexin 8 // --- // 100059657 /// XM_001492178 // S	SNX8	Cluster0002
15009979	XM_001495258 // SP3 // Sp3 transcription factor // --- // 100064317 /// ENSECAT00000014	SP3	Cluster0002
15040760	ENSECAT00000007222 // SPATA2 // spermatogenesis associated 2 // --- // 100071437 /// XM	SPATA2	Cluster0002
15008014	ENSECAT00000019755 // SPATS2L // spermatogenesis associated, serine-rich 2-like // ---	SPATS2L	Cluster0002
15111862	ENSECAT00000007304 // SPCS2 // signal peptidase complex subunit 2 homolog (S. cerevisia	SPCS2	Cluster0002
15013088	XM_001502881 // SPICE1 // spindle and centriole associated protein 1 // --- // 10006133	SPICE1	Cluster0002
14989284	XM_001494344 // SPRED2 // sprouty-related, EVH1 domain containing 2 // --- // 100062947	SPRED2	Cluster0002
15023327	ENSECAT00000028857 // SRRM1 // serine/arginine repetitive matrix 1 // --- // ---	SRRM1	Cluster0002
14975297	ENSECAT00000013256 // SRRT // serrate RNA effector molecule homolog (Arabidopsis) // --	SRRT	Cluster0002
15063416	XM_001498179 // STAM // signal transducing adaptor molecule (SH3 domain and ITAM motif)	STAM	Cluster0002
15009255	ENSECAT00000022963 // STAM2 // signal transducing adaptor molecule (SH3 domain and ITAM	STAM2	Cluster0002
14965942	ENSECAT00000013079 // STARD3 // StAR-related lipid transfer (START) domain containing 3	STARD3	Cluster0002
15128400	XM_001496620 // STARD8 // StAR-related lipid transfer (START) domain containing 8 // --	STARD8	Cluster0002
14947114	ENSECAT00000024489 // STARD9 // StAR-related lipid transfer (START) domain containing 9	STARD9	Cluster0002
14960057	XM_003362556 // STAT-3 // STAT-3 protein // --- // 100034011 /// ENSECAT00000018255 //	STAT-3	Cluster0002
15102927	ENSECAT00000009359 // STAT6 // signal transducer and activator of transcription 6, inte	STAT6	Cluster0002
14957974	ENSECAT00000018859 // STRA13 // stimulated by retinoic acid 13 homolog (mouse) // --- /	STRA13	Cluster0002
15048883	XM_001504042 // STX17 // syntaxin 17 // --- // 100054797 /// ENSECAT00000015963 // STX1	STX17	Cluster0002
14950034	XM_001915937 // SUPT5H // suppressor of Ty 5 homolog (S. cerevisiae) // --- // 10006451	SUPT5H	Cluster0002
14964298	ENSECAT00000023421 // SYNGR2 // synaptogyrin 2 // --- // ---	SYNGR2	Cluster0002
15047158	NM_001242552 // SYNJ2BP // synaptojanin 2 binding protein // --- // 100049833 /// ENSEC	SYNJ2BP	Cluster0002
15131220	ENSECAT00000018502 // TAB3 // TGF-beta activated kinase 1/MAP3K7 binding protein 3 // -	TAB3	Cluster0002
14978382	ENSECAT00000009086 // TAF6 // TAF6 RNA polymerase II, TATA box binding protein (TBP)-as	TAF6	Cluster0002
14962091	ENSECAT00000006735 // TAOK1 // TAO kinase 1 // --- // 100059705 /// XM_001504208 // TAO	TAOK1	Cluster0002
15067996	XM_001498618 // TAPT1 // transmembrane anterior posterior transformation 1 // --- // 10	TAPT1	Cluster0002
15050067	XM_001500358 // TBC1D13 // TBC1 domain family, member 13 // --- // 100070184 /// ENSECA	TBC1D13	Cluster0002
15037108	XM_003363867 // TBC1D20 // TBC1 domain family, member 20 // --- // 100067965 /// ENSECA	TBC1D20	Cluster0002
15127924	ENSECAT00000025175 // TBC1D25 // TBC1 domain family, member 25 // --- // 100061931 ///	TBC1D25	Cluster0002
14978775	ENSECAT00000016510 // TBL2 // transducin (beta)-like 2 // --- // 100059856 /// XM_00150	TBL2	Cluster0002
15023403	NM_001252407 // TCEB3 // transcription elongation factor B (SIII), polypeptide 3 (110kD	TCEB3	Cluster0002
15061332	ENSECAT00000007527 // TCF20 // transcription factor 20 (AR1) // --- // 100056129 /// XM	TCF20	Cluster0002
14941662	ENSECAT00000025956 // TCF7L2 // transcription factor 7-like 2 (T-cell specific, HMG-box	TCF7L2	Cluster0002
14971525	ENSECAT00000011204 // TCIRG1 // T-cell, immune regulator 1, ATPase, H+ transporting, ly	TCIRG1	Cluster0002
15110653	ENSECAT00000020391 // TECR // trans-2,3-enoyl-CoA reductase // --- // 100065024 /// XM_	TECR	Cluster0002
15048470	XM_001497920 // TESK1 // testis-specific kinase 1 // --- // 100068022 /// ENSECAT000000	TESK1	Cluster0002
15016347	XM_001496148 // TESK2 // testis-specific kinase 2 // --- // 100052478 /// ENSECAT000000	TESK2	Cluster0002
15026516	XM_001503217 // TET2 // tet methylcytosine dioxygenase 2 // --- // 100073065 /// ENSECA	TET2	Cluster0002
14992457	XM_001917114 // TET3 // tet methylcytosine dioxygenase 3 // --- // 100057820 /// ENSECA	TET3	Cluster0002
15051587	XM_001504032 // TEX10 // testis expressed 10 // --- // 100063401 /// ENSECAT00000019581	TEX10	Cluster0002
15031547	ENSECAT00000021087 // TFEB // transcription factor EB // --- // 100066433 /// XM_001496	TFEB	Cluster0002
14954224	ENSECAT00000012048 // TGFB1 // transforming growth factor, beta 1 // --- // 100033900 /	TGFB1	Cluster0002
15126975	NM_001081771 // TLR7 // toll-like receptor 7 // --- // 791248 /// ENSECAT00000007908 //	TLR7	Cluster0002
15126979	NM_001111301 // TLR8 // toll-like receptor 8 // --- // 100054367 /// ENSECAT00000009327	TLR8	Cluster0002
14948312	XM_001490013 // TM9SF1 // transmembrane 9 superfamily member 1 // --- // 100051449 ///	TM9SF1	Cluster0002
14985681	XM_001504162 // TMCO6 // transmembrane and coiled-coil domains 6 // --- // 100061843 //	TMCO6	Cluster0002
14964697	ENSECAT00000006437 // TMEM104 // transmembrane protein 104 // --- // ---	TMEM104	Cluster0002

14983853	ENSECAT00000027090 // TMEM161B // transmembrane protein 161B // --- // 100073241 /// XM	TMEM161B	Cluster0002
15072812	XM_001488221 // TMEM175 // transmembrane protein 175 // --- // 100051913 /// ENSECAT000	TMEM175	Cluster0002
14942025	ENSECAT00000015514 // TMEM180 // transmembrane protein 180 // --- // 100059957 /// XM_0	TMEM180	Cluster0002
15073923	ENSECAT00000008973 // TMEM183A // transmembrane protein 183A // --- // 100065104 /// XM	TMEM183A	Cluster0002
15023303	ENSECAT00000023814 // TMEM50A // transmembrane protein 50A // --- // ---	TMEM50A	Cluster0002
15021734	ENSECAT00000025112 // TMEM69 // transmembrane protein 69 // --- // 100065125 /// XM_001	TMEM69	Cluster0002
14961946	ENSECAT00000013503 // TNFAIP1 // tumor necrosis factor, alpha-induced protein 1 (endoth	TNFAIP1	Cluster0002
15099945	ENSECAT00000017432 // TNFRSF1A // tumor necrosis factor receptor superfamily, member 1A	TNFRSF1A	Cluster0002
15105879	ENSECAT00000014998 // TNPO2 // transportin 2 // --- // 100063482 /// XM_001504888 // TN	TNPO2	Cluster0002
15059199	ENSECAT00000010386 // TNRC6B // trinucleotide repeat containing 6B // --- // ---	TNRC6B	Cluster0002
15021768	ENSECAT00000024892 // TOE1 // target of EGR1, member 1 (nuclear) // --- // 100052538 //	TOE1	Cluster0002
15058884	XM_003364290 // TOM1 // target of myb1 (chicken) // --- // 100054292 /// ENSECAT0000001	TOM1	Cluster0002
14968520	ENSECAT00000008309 // TP53 // tumor protein p53 // --- // 100062044 /// NM_001202405 //	TP53	Cluster0002
15046331	XM_001490000 // TRAF3 // TNF receptor-associated factor 3 // --- // 100055110 /// ENSEC	TRAF3	Cluster0002
15112116	ENSECAT00000006616 // TRIM21 // tripartite motif containing 21 // --- // 100066782 ///	TRIM21	Cluster0002
15079112	XM_001496985 // TRIM24 // tripartite motif containing 24 // --- // 100066810 /// ENSECA	TRIM24	Cluster0002
15030361	ENSECAT00000017994 // TRIM26 // tripartite motif containing 26 // --- // 100051726 ///	TRIM26	Cluster0002
15030268	XM_001492536 // TRIM27 // tripartite motif containing 27 // --- // 100060192 /// ENSECA	TRIM27	Cluster0002
14975351	NM_001257087 // TRIM56 // tripartite motif containing 56 // --- // 100060136 /// ENSECA	TRIM56	Cluster0002
14998490	ENSECAT00000026741 // TSC22D2 // TSC22 domain family, member 2 // --- // ---	TSC22D2	Cluster0002
15047787	ENSECAT00000013808 // TTC7B // tetratricopeptide repeat domain 7B // --- // 100062817 /	TTC7B	Cluster0002
15040288	XM_001502393 // TTI1 // TELO2 interacting protein 1 // --- // 100055499 /// ENSECAT0000	TTI1	Cluster0002
15094145	XM_001492221 // TTLL4 // tubulin tyrosine ligase-like family, member 4 // --- // 100055	TTLL4	Cluster0002
15038091	ENSECAT00000016197 // TTPAL // tocopherol (alpha) transfer protein-like // --- // 10005	TTPAL	Cluster0002
15096839	XM_001504181 // TUBA1C // tubulin, alpha 1c // --- // 100051901 /// ENSECAT00000009703	TUBA1C	Cluster0002
15113972	XM_001488340 // TUBA3D // tubulin, alpha 3d // --- // 100052388 /// ENSECAT00000015106	TUBA3D	Cluster0002
15098777	XM_001491910 // TUBA4A // tubulin, alpha 4a // --- // 100059249 /// ENSECAT00000015274	TUBA4A	Cluster0002
14947054	XM_001503141 // TUBGCP4 // tubulin, gamma complex associated protein 4 // --- // 100056	TUBGCP4	Cluster0002
14951839	XM_001496109 // U2AF2 // U2 small nuclear RNA auxiliary factor 2 // --- // 100050729 //	U2AF2	Cluster0002
14995366	XM_001498639 // UBA3 // ubiquitin-like modifier activating enzyme 3 // --- // 100053395	UBA3	Cluster0002
15067117	XM_001497368 // UBA6 // ubiquitin-like modifier activating enzyme 6 // --- // 100067272	UBA6	Cluster0002
14996357	XM_001497081 // UBA7 // ubiquitin-like modifier activating enzyme 7 // --- // 100052809	UBA7	Cluster0002
15042406	XM_001499590 // UBAP1 // ubiquitin associated protein 1 // --- // 100053997 /// ENSECAT	UBAP1	Cluster0002
15091286	ENSECAT00000006859 // UBAP2L // ubiquitin associated protein 2-like // --- // 100056822	UBAP2L	Cluster0002
15129707	ENSECAT00000020216 // UBE2A // ubiquitin-conjugating enzyme E2A // --- // 100058679 ///	UBE2A	Cluster0002
15040763	ENSECAT00000012363 // UBE2V1 // ubiquitin-conjugating enzyme E2 variant 1 // --- // ---	UBE2V1	Cluster0002
14981592	ENSECAT00000012064 // UIMC1 // ubiquitin interaction motif containing 1 // --- // 10005	UIMC1	Cluster0002
14974095	XM_001916968 // UNC93B1 // unc-93 homolog B1 (C. elegans) // --- // 100059773 /// ENSEC	UNC93B1	Cluster0002
14958804	XM_001496857 // USH1G // Usher syndrome 1G (autosomal recessive) // --- // 100052352 //	USH1G	Cluster0002
15085523	XM_001503830 // USP21 // ubiquitin specific peptidase 21 // --- // 100066197 /// XM_001	USP21	Cluster0002
14969315	XM_001488603 // USP22 // ubiquitin specific peptidase 22 // --- // 100053238 /// ENSECA	USP22	Cluster0002
14958266	XM_001491130 // USP36 // ubiquitin specific peptidase 36 // --- // 100057557 /// ENSECA	USP36	Cluster0002
15002716	XM_001493357 // USPL1 // ubiquitin specific peptidase like 1 // --- // 100062086 /// EN	USPL1	Cluster0002
15058197	ENSECAT00000004005 // VEZT // vezatin, adherens junctions transmembrane protein // ---	VEZT	Cluster0002
14961454	XM_001503742 // VMP1 // vacuole membrane protein 1 // --- // 100057406 /// ENSECAT00000	VMP1	Cluster0002
15126737	ENSECAT00000029068 // VPS25 // vacuolar protein sorting 25 homolog (S. cerevisiae) // -	VPS25	Cluster0002
14938922	ENSECAT00000006741 // VPS39 // vacuolar protein sorting 39 homolog (S. cerevisiae) // -	VPS39	Cluster0002
15063031	XM_001495136 // WAC // WW domain containing adaptor with coiled-coil // --- // 10006415	WAC	Cluster0002
15100665	ENSECAT00000008643 // WBP11 // WW domain binding protein 11 // --- // 100063867	WBP11	Cluster0002
15107707	XM_001504915 // WEE1 // WEE1 homolog (S. pombe) // --- // 100071182 /// ENSECAT00000022	WEE1	Cluster0002
14944698	XM_001498201 // WHAMM // WAS protein homolog associated with actin, golgi membranes and	WHAMM	Cluster0002
15068409	ENSECAT00000010781 // WHSC2 // Wolf-Hirschhorn syndrome candidate 2 // --- // ---	WHSC2	Cluster0002
14978100	ENSECAT00000023722 // WIPI2 // WD repeat domain, phosphoinositide interacting 2 // ---	WIPI2	Cluster0002
14973869	NM_001110210 // YIF1A // Yip1 interacting factor homolog A (S. cerevisiae) // --- // 10	YIF1A	Cluster0002

15120723	ENSECAT00000002752 // ZADH2 // zinc binding alcohol dehydrogenase domain containing 2 /	ZADH2	Cluster0002
15075498	XM_001501770 // ZBTB2 // zinc finger and BTB domain containing 2 // --- // 100060294 //	ZBTB2	Cluster0002
15129726	ENSECAT00000007209 // ZBTB33 // zinc finger and BTB domain containing 33 // --- // 1000	ZBTB33	Cluster0002
15104363	ENSECAT00000000381 // ZC3H12C // zinc finger CCCH-type containing 12C // --- // 1000700	ZC3H12C	Cluster0002
15045541	ENSECAT00000014389 // ZC3H14 // zinc finger CCCH-type containing 14 // --- // ---	ZC3H14	Cluster0002
15082783	ENSECAT00000019165 // ZC3HC1 // zinc finger, C3HC-type containing 1 // --- // 100057013	ZC3HC1	Cluster0002
14942391	ENSECAT00000010893 // ZDHHC16 // zinc finger, DHHC-type containing 16 // --- // 1000610	ZDHHC16	Cluster0002
14998879	ENSECAT00000022971 // ZFYVE20 // zinc finger, FYVE domain containing 20 // --- // 10005	ZFYVE20	Cluster0002
15076970	ENSECAT00000026848 // ZMIZ2 // zinc finger, MIZ-type containing 2 // --- // 100051958 /	ZMIZ2	Cluster0002
15098625	ENSECAT00000018824 // ZNF142 // zinc finger protein 142 // --- // 100055767 /// XM_0014	ZNF142	Cluster0002
15107691	XM_001504913 // ZNF143 // zinc finger protein 143 // --- // 100055723 /// ENSECAT000000	ZNF143	Cluster0002
14951962	ENSECAT00000022753 // ZNF264 // zinc finger protein 264 // --- // ---	ZNF264	Cluster0002
15133309	ENSECAT00000018945 // ZNF280C // zinc finger protein 280C // --- // 100058803	ZNF280C	Cluster0002
14937879	ENSECAT00000008641 // ZNF280D // zinc finger protein 280D // --- // 100054593	ZNF280D	Cluster0002
15055462	ENSECAT00000013536 // ZNF295 // zinc finger protein 295 // --- // 100058234 /// XM_0014	ZNF295	Cluster0002
15100060	XM_003365164 // ZNF384 // zinc finger protein 384 // --- // 100059879 /// ENSECAT000000	ZNF384	Cluster0002
15045207	XM_001490028 // ZNF410 // zinc finger protein 410 // --- // 100050679 /// ENSECAT000000	ZNF410	Cluster0002
14952089	ENSECAT00000017488 // ZNF446 // zinc finger protein 446 // --- // ---	ZNF446	Cluster0002
14951297	ENSECAT00000025016 // ZNF473 // zinc finger protein 473 // --- // 100056907 /// XM_0014	ZNF473	Cluster0002
14979141	XM_001500835 // ZNF646 // zinc finger protein 646 // --- // 100063391 /// ENSECAT000000	ZNF646	Cluster0002
14975864	ENSECAT00000004478 // ZNF689 // zinc finger protein 689 // --- // ---	ZNF689	Cluster0002
15130030	XM_001494693 // ZNF75D // zinc finger protein 75D // --- // 100063485 /// ENSECAT000000	ZNF75D	Cluster0002
15006103	XM_001504106 // ZNF828 // chromosome alignment maintaining phosphoprotein 1 // --- // 1	ZNF828	Cluster0002
14949709	XM_001495128 // ZNF829 // zinc finger protein 829 // --- // 100061841 /// ENSECAT000000	ZNF829	Cluster0002
15109678	XM_001502053 // ZW10 // ZW10, kinetochore associated, homolog (Drosophila) // --- // 10	ZW10	Cluster0002
14961638	ENSECAT00000008813 // AATF // apoptosis antagonizing transcription factor // --- // 100	AATF	Cluster0003
14958998	ENSECAT00000007910 // ABCA6 // ATP-binding cassette, sub-family A (ABC1), member 6 // -	ABCA6	Cluster0003
14959027	ENSECAT00000025043 // ABCA9 // ATP-binding cassette, sub-family A (ABC1), member 9 // -	ABCA9	Cluster0003
14935005	ENSECAT00000018939 // ABCB10 // ATP-binding cassette, sub-family B (MDR/TAP), member 10	ABCB10	Cluster0003
15014145	ENSECAT00000008988 // ABCC5 // ATP-binding cassette, sub-family C (CFTR/MRP), member 5	ABCC5	Cluster0003
15047303	XM_001490380 // ABCD4 // ATP-binding cassette, sub-family D (ALD), member 4 // --- // 1	ABCD4	Cluster0003
15066315	DQ825759 // ABCG2 // ATP-binding cassette, sub-family G (WHITE), member 2 // --- // 100	ABCG2	Cluster0003
14997353	XM_001488559 // ACAA1 // acetyl-CoA acyltransferase 1 // --- // 100050082 /// ENSECAT00	ACAA1	Cluster0003
14966758	ENSECAT00000013599 // ACACA // acetyl-CoA carboxylase alpha // --- // 100071472 /// XM_	ACACA	Cluster0003
14998753	XM_001489245 // ACAD9 // acyl-CoA dehydrogenase family, member 9 // --- // 100054922 //	ACAD9	Cluster0003
15072515	ENSECAT00000009087 // ACOX3 // acyl-CoA oxidase 3, pristanoyl // --- // 100056690 /// X	ACOX3	Cluster0003
14997992	ENSECAT00000022354 // ACPP // acid phosphatase, prostate // --- // 100064835 /// XM_001	ACPP	Cluster0003
15132878	ENSECAT00000018304 // ACSL4 // acyl-CoA synthetase long-chain family member 4 // --- //	ACSL4	Cluster0003
15097128	XM_003365238 // ACVR1B // activin A receptor, type IB // --- // 100062619 /// ENSECAT00	ACVR1B	Cluster0003
15019599	ENSECAT00000015968 // ADAM28 // ADAM metallopeptidase domain 28 // --- // ---	ADAM28	Cluster0003
14967087	ENSECAT00000016351 // ADAP2 // ArfGAP with dual PH domains 2 // --- // 100058484 /// XM	ADAP2	Cluster0003
15092694	NM_001110308 // AGL // amylo-alpha-1, 6-glucosidase, 4-alpha-glucanotransferase // ---	AGL	Cluster0003
15054367	ENSECAT00000024640 // AGPAT3 // 1-acylglycerol-3-phosphate O-acyltransferase 3 // --- /	AGPAT3	Cluster0003
14973199	ENSECAT00000014851 // AHNAK // AHNAK nucleoprotein // --- // ---	AHNAK	Cluster0003
14952626	XM_001915854 // AIM1 // absent in melanoma 1 // --- // 100066371 /// ENSECAT00000015934	AIM1	Cluster0003
14945047	ENSECAT00000009990 // AKAP13 // A kinase (PRKA) anchor protein 13 // --- // 100070242 /	AKAP13	Cluster0003
15077413	ENSECAT00000022231 // AKAP9 // A kinase (PRKA) anchor protein (yotiao) 9 // --- // 1000	AKAP9	Cluster0003
15048857	ENSECAT00000005562 // ALK5 // TGF beta receptor type I // --- // 100034117 /// XM_00149	ALK5	Cluster0003
14985700	NM_001204105 // ANKHD1 // ankyrin repeat and KH domain containing 1 // --- // 100072419	ANKHD1	Cluster0003
15077461	XM_001915085 // ANKIB1 // ankyrin repeat and IBR domain containing 1 // --- // 10006126	ANKIB1	Cluster0003
15119118	ENSECAT00000026398 // ANKLE2 // ankyrin repeat and LEM domain containing 2 // --- // 10	ANKLE2	Cluster0003
15010940	ENSECAT00000009858 // ANKRD44 // ankyrin repeat domain 44 // --- // 100070473 /// ENSEC	ANKRD44	Cluster0003
15011051	ENSECAT00000021847 // AOX1 // aldehyde oxidase 1 // --- // 100147243 /// ENSECAT0000002	AOX1	Cluster0003
14948235	XM_001491874 // AP1G2 // adaptor-related protein complex 1, gamma 2 subunit // --- // 1	AP1G2	Cluster0003

14984011	ENSECAT00000024967 // AP3B1 // adaptor-related protein complex 3, beta 1 subunit // ---	AP3B1	Cluster0003
15063102	ENSECAT00000024444 // APBB1IP // amyloid beta (A4) precursor protein-binding, family B,	APBB1IP	Cluster0003
14986608	XM_001504579 // APC // adenomatous polyposis coli // --- // 100064431 /// XM_003362839	APC	Cluster0003
15060677	ENSECAT00000022563 // APLP2 // amyloid beta (A4) precursor-like protein 2 // --- // 100	APLP2	Cluster0003
14939315	XM_001503643 // AQR // aquarius homolog (mouse) // --- // 100057637 /// ENSECAT00000017	AQR	Cluster0003
15107095	ENSECAT00000017568 // ARAP1 // ArfGAP with RhoGAP domain, ankyrin repeat and PH domain	ARAP1	Cluster0003
15038345	ENSECAT00000025931 // ARFGEF2 // ADP-ribosylation factor guanine nucleotide-exchange fa	ARFGEF2	Cluster0003
15062004	XM_001493210 // ARHGAP12 // Rho GTPase activating protein 12 // --- // 100055065 /// EN	ARHGAP12	Cluster0003
14976292	ENSECAT00000018524 // ARHGAP17 // Rho GTPase activating protein 17 // --- // 100069347	ARHGAP17	Cluster0003
14957489	XM_001503227 // ARHGAP18 // Rho GTPase activating protein 18 // --- // 100073070 /// EN	ARHGAP18	Cluster0003
15070742	XM_001495116 // ARHGAP24 // Rho GTPase activating protein 24 // --- // 100052555 /// EN	ARHGAP24	Cluster0003
15102991	XM_001488847 // ARHGAP9 // Rho GTPase activating protein 9 // --- // 100050388 /// ENSE	ARHGAP9	Cluster0003
14998207	ENSECAT00000015368 // ARMC8 // armadillo repeat containing 8 // --- // ---	ARMC8	Cluster0003
15086259	XM_001499084 // ASH1L // ash1 (absent, small, or homeotic)-like (Drosophila) // --- //	ASH1L	Cluster0003
15057009	ENSECAT00000014641 // ASH2L // ash2 (absent, small, or homeotic)-like (Drosophila) // -	ASH2L	Cluster0003
15085626	XM_001488045 // ATF6 // activating transcription factor 6 // --- // 100051169 /// ENSEC	ATF6	Cluster0003
14998988	ENSECAT00000024336 // ATG7 // autophagy related 7 // --- // 100051544 /// XM_001493002	ATG7	Cluster0003
15093930	XM_001489038 // ATIC // 5-aminoimidazole-4-carboxamide ribonucleotide formyltransferase	ATIC	Cluster0003
14945363	XM_001917920 // ATP10A // ATPase, class V, type 10A // --- // 100062211 /// ENSECAT0000	ATP10A	Cluster0003
15004140	ENSECAT00000015729 // ATP11A // ATPase, class VI, type 11A // --- // 100066940	ATP11A	Cluster0003
15133614	ENSECAT00000006990 // ATP11C // ATPase, class VI, type 11C // --- // 100055432 /// XM_0	ATP11C	Cluster0003
15083820	ENSECAT00000006583 // ATP2B4 // ATPase, Ca++ transporting, plasma membrane 4 // --- //	ATP2B4	Cluster0003
15067593	ENSECAT00000025132 // ATP8A1 // ATPase, aminophospholipid transporter (APLT), class I,	ATP8A1	Cluster0003
14938303	XM_001499692 // ATP8B4 // ATPase, class I, type 8B, member 4 // --- // 100069998 /// EN	ATP8B4	Cluster0003
14939304	ENSECAT00000014987 // ATPBD4 // ATP binding domain 4 // --- // ---	ATPBD4	Cluster0003
15002056	ENSECAT00000012259 // ATR // ataxia telangiectasia and Rad3 related // --- // 100062529	ATR	Cluster0003
14950270	ENSECAT00000012479 // AXL // AXL receptor tyrosine kinase // --- // 100064907 /// ENSEC	AXL	Cluster0003
15009379	ENSECAT00000013365 // BAZ2B // bromodomain adjacent to zinc finger domain, 2B // --- //	BAZ2B	Cluster0003
15015251	ENSECAT00000025291 // BBX // bobby sox homolog (Drosophila) // --- // 100061782	BBX	Cluster0003
15088133	ENSECAT00000024424 // BCAR3 // breast cancer anti-estrogen resistance 3 // --- // 10005	BCAR3	Cluster0003
14966692	ENSECAT00000009604 // BCAS3 // breast carcinoma amplified sequence 3 // --- // --- ///	BCAS3	Cluster0003
14993636	ENSECAT00000021569 // BIRC6 // baculoviral IAP repeat containing 6 // --- // 100070568	BIRC6	Cluster0003
15070843	ENSECAT00000017038 // BMP2K // BMP2 inducible kinase // --- // ---	BMP2K	Cluster0003
14943866	XM_001489945 // BMS1 // BMS1 homolog, ribosome assembly protein (yeast) // --- // 10005	BMS1	Cluster0003
15031426	ENSECAT00000018372 // BTBD9 // BTB (POZ) domain containing 9 // --- // 100065100 /// EN	BTBD9	Cluster0003
14934927	ENSECAT00000010910 // C10orf11 // chromosome 10 open reading frame 11 // --- // ---	C10orf11	Cluster0003
15048088	ENSECAT00000016037 // C14orf49 // chromosome 14 open reading frame 49 // --- // ---	C14orf49	Cluster0003
14979961	ENSECAT00000008938 // C16orf62 // chromosome 16 open reading frame 62 // --- // ---	C16orf62	Cluster0003
15131423	ENSECAT00000015283 // CASK // calcium/calmodulin-dependent serine protein kinase (MAGUK	CASK	Cluster0003
15057493	XM_001491168 // CCDC111 // coiled-coil domain containing 111 // --- // 100058061 /// EN	CCDC111	Cluster0003
15105323	ENSECAT00000009747 // CCDC15 // coiled-coil domain containing 15 // --- // ---	CCDC15	Cluster0003
15012340	ENSECAT00000015618 // CCDC50 // coiled-coil domain containing 50 // --- // ---	CCDC50	Cluster0003
15085643	NM_001081927 // CD14 // CD14 molecule-like // --- // 100034125 /// NM_001081927 // CD14	CD14	Cluster0003
15100171	ENSECAT00000020379 // CD163 // CD163 molecule // --- // ---	CD163	Cluster0003
15085645	ENSECAT00000024021 // CD244 // CD244 molecule, natural killer cell receptor 2B4 // ---	CD244	Cluster0003
15092628	ENSECAT00000017996 // CDC14A // CDC14 cell division cycle 14 homolog A (S. cerevisiae)	CDC14A	Cluster0003
15026981	XM_001494637 // CDKAL1 // CDK5 regulatory subunit associated protein 1-like 1 // --- //	CDKAL1	Cluster0003
15070754	XM_001915383 // CDS1 // CDP-diacylglycerol synthase (phosphatidate cytidylyltransferase	CDS1	Cluster0003
15061584	XM_001488742 // CERK // ceramide kinase // --- // 100053604 /// ENSECAT00000005822 // C	CERK	Cluster0003
15040346	XM_001502770 // CHD6 // chromodomain helicase DNA binding protein 6 // --- // 100055799	CHD6	Cluster0003
15064009	ENSECAT00000012261 // CHD9 // chromodomain helicase DNA binding protein 9 // --- // ---	CHD9	Cluster0003
14974104	XM_001492289 // CHKA // choline kinase alpha // --- // 100059839 /// ENSECAT00000012200	CHKA	Cluster0003
14997439	ENSECAT00000017801 // CLASP2 // cytoplasmic linker associated protein 2 // --- // 10005	CLASP2	Cluster0003
15128045	XM_001495945 // CLCN5 // chloride channel, voltage-sensitive 5 // --- // 100052289 ///	CLCN5	Cluster0003

15024088	ENSECAT00000016665 // CLCN6 // chloride channel, voltage-sensitive 6 // --- // 10005620	CLCN6	Cluster0003
15107148	XM_001917473 // CLPB // ClpB caseinolytic peptidase B homolog (E. coli) // --- // 10006	CLPB	Cluster0003
15068976	XM_001495241 // CNOT1 // CCR4-NOT transcription complex, subunit 1 // --- // 100052554	CNOT1	Cluster0003
14965666	XM_001494164 // COASY // CoA synthase // --- // 100066023 /// ENSECAT00000008734 // COA	COASY	Cluster0003
15051087	ENSECAT00000008185 // COBRA1 // cofactor of BRCA1 // --- // ---	COBRA1	Cluster0003
15080426	XM_001492486 // COG5 // component of oligomeric golgi complex 5 // --- // 100060129 ///	COG5	Cluster0003
14984090	ENSECAT00000018857 // COL4A3BP // collagen, type IV, alpha 3 (Goodpasture antigen) bind	COL4A3BP	Cluster0003
15082825	XM_001498558 // COPG2 // coatomer protein complex, subunit gamma 2 // --- // 100068740	COPG2	Cluster0003
14974139	ENSECAT00000018528 // CPT1A // carnitine palmitoyltransferase 1A (liver) // --- // 1000	CPT1A	Cluster0003
15082071	XM_001500152 // CPVL // carboxypeptidase, vitellogenic-like // --- // 100069591 /// ENS	CPVL	Cluster0003
15077251	ENSECAT00000009328 // CROT // carnitine O-octanoyltransferase // --- // 100050111 /// X	CROT	Cluster0003
14982268	ENSECAT00000003682 // CSF1R // colony stimulating factor 1 receptor // --- // 100060422	CSF1R	Cluster0003
15024564	NM_001081818 // C-SKI // SKI // --- // 100033833 /// ENSECAT00000013268 // C-SKI // SKI	C-SKI	Cluster0003
14937537	EF397512 // CSNK1G1 // casein kinase 1, gamma 1 // --- // 100053829 /// EF397513 // CSN	CSNK1G1	Cluster0003
15117068	XM_001496009 // CTDP1 // CTD (carboxy-terminal domain, RNA polymerase II, polypeptide A	CTDP1	Cluster0003
14970086	ENSECAT00000019101 // CTNND1 // catenin (cadherin-associated protein), delta 1 // --- /	CTNND1	Cluster0003
15061879	XM_001491887 // CUL2 // cullin 2 // --- // 100054982 /// ENSECAT00000016792 // CUL2 //	CUL2	Cluster0003
15044474	XM_001497278 // DAAM1 // dishevelled associated activator of morphogenesis 1 // --- //	DAAM1	Cluster0003
15007258	ENSECAT00000025338 // DCAF17 // DDB1 and CUL4 associated factor 17 // --- // 100063621	DCAF17	Cluster0003
14988953	XM_001916726 // DCTN1 // dynactin 1 // --- // 100053938 /// ENSECAT00000007498 // DCTN1	DCTN1	Cluster0003
15046670	XM_001489495 // DDHD1 // DDHD domain containing 1 // --- // 100055200 /// ENSECAT000000	DDHD1	Cluster0003
15104341	XM_001499568 // DDX10 // DEAD (Asp-Glu-Ala-Asp) box polypeptide 10 // --- // 100069845	DDX10	Cluster0003
15069606	XM_001498400 // DDX19B // DEAD (Asp-Glu-Ala-Asp) box polypeptide 19B // --- // 10006857	DDX19B	Cluster0003
15028186	XM_003363703 // DEF6 // differentially expressed in FDCP 6 homolog (mouse) // --- // 10	DEF6	Cluster0003
15052520	ENSECAT00000023899 // DENND1A // DENN/MADD domain containing 1A // --- // ---	DENND1A	Cluster0003
15087625	XM_001498238 // DENND2D // DENN/MADD domain containing 2D // --- // 100058822 /// ENSEC	DENND2D	Cluster0003
15117519	XM_003365446 // DEPDC5 // DEP domain containing 5 // --- // 100058243 /// XM_001496321	DEPDC5	Cluster0003
14961270	XM_001503319 // DGKE // diacylglycerol kinase, epsilon 64kDa // --- // 100056676 /// EN	DGKE	Cluster0003
15084903	XM_001489480 // DHX9 // DEAH (Asp-Glu-Ala-His) box polypeptide 9 // --- // 100055171 //	DHX9	Cluster0003
15128987	ENSECAT00000012332 // DIAPH2 // diaphanous homolog 2 (Drosophila) // --- // 100057058	DIAPH2	Cluster0003
15048050	ENSECAT00000007356 // DICER1 // dicer 1, ribonuclease type III // --- // 100065648 ///	DICER1	Cluster0003
14946253	XM_001918098 // DIS3L // DIS3 mitotic control homolog (S. cerevisiae)-like // --- // 10	DIS3L	Cluster0003
15012405	ENSECAT00000013029 // DLG1 // discs, large homolog 1 (Drosophila) // --- // 100060094 /	DLG1	Cluster0003
14938164	XM_001918182 // DMXL2 // Dmx-like 2 // --- // 100055186 /// ENSECAT00000001362 // DMXL2	DMXL2	Cluster0003
15018809	ENSECAT00000007404 // DNAJC11 // DnaJ (Hsp40) homolog, subfamily C, member 11 // --- //	DNAJC11	Cluster0003
14982693	XM_001504229 // DNAJC18 // DnaJ (Hsp40) homolog, subfamily C, member 18 // --- // 10006	DNAJC18	Cluster0003
15129618	ENSECAT00000022507 // DOCK11 // dedicator of cytokinesis 11 // --- // 100055441 /// ENS	DOCK11	Cluster0003
14984758	ENSECAT00000010317 // DOCK2 // dedicator of cytokinesis 2 // --- // 100070446 /// ENSEC	DOCK2	Cluster0003
14952314	ENSECAT00000006627 // DOPEY1 // dopey family member 1 // --- // 100065361 /// XM_001915	DOPEY1	Cluster0003
15088003	XM_001490618 // DPYD // dihydropyrimidine dehydrogenase // --- // 100057177 /// ENSECAT	DPYD	Cluster0003
15033910	XM_001500714 // DROSHA // drosha, ribonuclease type III // --- // 100054238 /// ENSECAT	DROSHA	Cluster0003
15032182	ENSECAT00000019218 // DST // dystonin // --- // 100056885 /// ENSECAT00000019286 // DST	DST	Cluster0003
15120193	ENSECAT00000003572 // DYM // dymeclin // --- // 100053667	DYM	Cluster0003
15015220	XM_001503246 // DZIP3 // DAZ interacting protein 3, zinc finger // --- // 100061685 ///	DZIP3	Cluster0003
14979763	ENSECAT00000024745 // EEF2K // eukaryotic elongation factor-2 kinase // --- // 10005838	EEF2K	Cluster0003
15122538	ENSECAT00000016194 // EFR3A // EFR3 homolog A (S. cerevisiae) // --- // 100057838 /// X	EFR3A	Cluster0003
15077164	ENSECAT00000011366 // EGFR // epidermal growth factor receptor // --- // 100067755	EGFR	Cluster0003
14970995	ENSECAT00000010852 // EHBP1L1 // EH domain binding protein 1-like 1 // --- // ---	EHBP1L1	Cluster0003
15016415	XM_001496198 // EIF2B3 // eukaryotic translation initiation factor 2B, subunit 3 gamma,	EIF2B3	Cluster0003
15022551	XM_001503663 // EIF2C1 // eukaryotic translation initiation factor 2C, 1 // --- // 1000	EIF2C1	Cluster0003
15019835	XM_001495587 // ELP3 // elongation protein 3 homolog (S. cerevisiae) // --- // 10005468	ELP3	Cluster0003
15066132	ENSECAT00000024297 // EMCN // endomucin // --- // ---	EMCN	Cluster0003
15115914	ENSECAT00000026568 // EMILIN2 // elastin microfibril interfacer 2 // --- // 100062096 /	EMILIN2	Cluster0003
14993407	XM_003363001 // EML4 // echinoderm microtubule associated protein like 4 // --- // 1000	EML4	Cluster0003

15029016	ENSECAT00000009130 // ENPP4 // ectonucleotide pyrophosphatase/phosphodiesterase 4 (puta	ENPP4	Cluster0003
15031833	ENSECAT00000012639 // ENPP5 // ectonucleotide pyrophosphatase/phosphodiesterase 5 (puta	ENPP5	Cluster0003
14942463	ENSECAT00000017357 // ENTPD1 // ectonucleoside triphosphate diphosphohydrolase 1 // ---	ENTPD1	Cluster0003
15073388	XM_001488930 // EPRS // glutamyl-prolyl-tRNA synthetase // --- // 100050245 /// ENSECAT	EPRS	Cluster0003
14983672	XM_001503689 // ERAP1 // endoplasmic reticulum aminopeptidase 1 // --- // 100073224 ///	ERAP1	Cluster0003
15099674	ENSECAT00000026910 // ERC1 // ELKS/RAB6-interacting/CAST family member 1 // --- // 1000	ERC1	Cluster0003
14935525	ENSECAT00000014986 // ERCC6 // excision repair cross-complementing rodent repair defici	ERCC6	Cluster0003
15036635	ENSECAT00000020543 // ESF1 // ESF1, nucleolar pre-rRNA processing protein, homolog (S.	ESF1	Cluster0003
15097636	ENSECAT00000014392 // ESYT1 // extended synaptotagmin-like protein 1 // --- // 10005197	ESYT1	Cluster0003
15088159	XM_003365094 // EVI5 // ecotropic viral integration site 5 // --- // 100059782 /// ENSE	EVI5	Cluster0003
15071487	ENSECAT00000025747 // EXOC1 // exocyst complex component 1 // --- // 100059087	EXOC1	Cluster0003
15029502	ENSECAT00000000068 // EXOC2 // exocyst complex component 2 // --- // 100049898 /// ENSE	EXOC2	Cluster0003
14935402	NM_001256924 // EXOC8 // exocyst complex component 8 // --- // 100051172 /// ENSECAT000	EXOC8	Cluster0003
15001061	ENSECAT00000016498 // EXOG // endo/exonuclease (5 -3), endonuclease G-like // --- // 1	EXOG	Cluster0003
15075680	XM_001492052 // EZR // ezrin // --- // 100059467 /// ENSECAT00000019839 // EZR // ezrin	EZR	Cluster0003
15016093	XM_001494368 // FAF1 // Fas (TNFRSF6) associated factor 1 // --- // 100062977 /// ENSEC	FAF1	Cluster0003
15075256	ENSECAT00000020080 // FAM120B // family with sequence similarity 120B // --- // ---	FAM120B	Cluster0003
14983808	ENSECAT00000019460 // FAM172A // family with sequence similarity 172, member A // --- /	FAM172A	Cluster0003
14942200	ENSECAT00000011855 // FAM178A // family with sequence similarity 178, member A // --- /	FAM178A	Cluster0003
14995794	ENSECAT00000010119 // FAM208A // family with sequence similarity 208, member A // --- /	FAM208A	Cluster0003
15026646	ENSECAT00000000235 // FARS2 // phenylalanyl-tRNA synthetase 2, mitochondrial // --- //	FARS2	Cluster0003
15063689	XM_001916971 // FBXO18 // F-box protein, helicase, 18 // --- // 100070322 /// ENSECAT00	FBXO18	Cluster0003
14952778	ENSECAT00000016794 // FIG4 // FIG4 homolog, SAC1 lipid phosphatase domain containing (S	FIG4	Cluster0003
15031285	ENSECAT00000011657 // FKBP5 // FK506 binding protein 5 // --- // 100053546 /// XM_00149	FKBP5	Cluster0003
15062493	ENSECAT00000014180 // FRMD4A // FERM domain containing 4A // --- // 100056511	FRMD4A	Cluster0003
14995340	ENSECAT00000017447 // FRMD4B // FERM domain containing 4B // --- // 100063494 /// XM_00	FRMD4B	Cluster0003
15067385	ENSECAT00000027142 // FRYL // FRY-like // --- // 100054294	FRYL	Cluster0003
15064067	ENSECAT00000024522 // FTO // fat mass and obesity associated // --- // 100060565 /// XM	FTO	Cluster0003
15050338	ENSECAT00000012324 // FUBP3 // far upstream element (FUSE) binding protein 3 // --- //	FUBP3	Cluster0003
15044831	ENSECAT00000015238 // FUT8 // fucosyltransferase 8 (alpha (1,6) fucosyltransferase) //	FUT8	Cluster0003
14997072	ENSECAT00000014425 // FYCO1 // FYVE and coiled-coil domain containing 1 // --- // 10005	FYCO1	Cluster0003
15103167	AF510336 // G6S // N-acetylglucosamine-6-sulfatase // --- // 100034131	G6S	Cluster0003
15106776	XM_001494299 // GAB2 // GRB2-associated binding protein 2 // --- // 100062877 /// ENSEC	GAB2	Cluster0003
15047666	ENSECAT00000010104 // GALC // galactosylceramidase // --- // 100034130	GALC	Cluster0003
15102031	ENSECAT00000017151 // GALNT6 // UDP-N-acetyl-alpha-D-galactosamine:polypeptide N-acetyl	GALNT6	Cluster0003
14973278	ENSECAT00000015836 // GANAB // glucosidase, alpha; neutral AB // --- // 100060146 /// X	GANAB	Cluster0003
14947172	ENSECAT00000026930 // GANC // glucosidase, alpha; neutral C // --- // 100070973 /// XM_	GANC	Cluster0003
15055189	XM_001497921 // GART // phosphoribosylglycinamide formyltransferase, phosphoribosylglyc	GART	Cluster0003
14981996	ENSECAT00000022126 // GEMIN5 // gem (nuclear organelle) associated protein 5 // --- //	GEMIN5	Cluster0003
15094691	ENSECAT00000026267 // GIGYF2 // GRB10 interacting GYF protein 2 // --- // 100064814 ///	GIGYF2	Cluster0003
15079849	XM_001494502 // GIMAP8 // GTPase, IMAP family member 8 // --- // 100063176 /// ENSECAT0	GIMAP8	Cluster0003
14946686	ENSECAT00000021118 // GLDN // gliomedin // --- // ---	GLDN	Cluster0003
15049976	XM_001500680 // GLE1 // GLE1 RNA export mediator homolog (yeast) // --- // 100066964 //	GLE1	Cluster0003
15029536	ENSECAT00000001963 // GMDS // GDP-mannose 4,6-dehydratase // --- // 100057325 /// XM_00	GMDS	Cluster0003
15001238	XM_001489013 // GOLGA4 // golgin A4 // --- // 100050216 /// ENSECAT00000014443 // GOLGA	GOLGA4	Cluster0003
15036779	XM_001915486 // GPCPD1 // glycerophosphocholine phosphodiesterase GDE1 homolog (S. cere	GPCPD1	Cluster0003
15006839	ENSECAT00000019811 // GPD2 // glycerol-3-phosphate dehydrogenase 2 (mitochondrial) // -	GPD2	Cluster0003
15081027	XM_001498401 // GRB10 // growth factor receptor-bound protein 10 // --- // 100052610 //	GRB10	Cluster0003
15131648	ENSECAT00000021176 // GRIPAP1 // GRIP1 associated protein 1 // --- // ---	GRIPAP1	Cluster0003
14984435	ENSECAT00000021646 // GRK6 // G protein-coupled receptor kinase 6 // --- // 100068570 /	GRK6	Cluster0003
14975641	ENSECAT00000022482 // GTF2I // general transcription factor IIi // --- // --- /// ENSEC	GTF2I	Cluster0003
14976233	ENSECAT00000011161 // GTF3C1 // general transcription factor IIIC, polypeptide 1, alpha	GTF3C1	Cluster0003
15010893	XM_001502507 // GTF3C3 // general transcription factor IIIC, polypeptide 3, 102kDa // -	GTF3C3	Cluster0003
14982930	XM_001504362 // H2AFY // H2A histone family, member Y // --- // 100062747 /// XM_003362	H2AFY	Cluster0003

14935112	ENSECAT00000023505 // HEATR1 // HEAT repeat containing 1 // --- // 100050411 /// XM_001	HEATR1	Cluster0003
14948567	ENSECAT00000017428 // HEATR5A // HEAT repeat containing 5A // --- // ---	HEATR5A	Cluster0003
14945429	ENSECAT00000019649 // HERC2 // HECT and RLD domain containing E3 ubiquitin protein liga	HERC2	Cluster0003
14934513	XM_001917996 // HK1 // hexokinase 1 // --- // 100072687 /// ENSECAT00000012040 // HK1 /	HK1	Cluster0003
15055274	ENSECAT00000012521 // HLCS // holocarboxylase synthetase (biotin-(proprionyl-CoA-carbox	HLCS	Cluster0003
15058873	XM_001499685 // HMGXB4 // HMG box domain containing 4 // --- // 100054243 /// ENSECAT00	HMGXB4	Cluster0003
14998456	ENSECAT00000008736 // HPS3 // Hermansky-Pudlak syndrome 3 // --- // 100058614	HPS3	Cluster0003
15066504	ENSECAT00000025455 // HPSE // heparanase // --- // 100061267 /// XM_001493282 // HPSE /	HPSE	Cluster0003
14986424	NM_001081901 // HSD17B4 // hydroxysteroid (17-beta) dehydrogenase 4 // --- // 100034046	HSD17B4	Cluster0003
15128169	ENSECAT00000024109 // HUWE1 // HECT, UBA and WWE domain containing 1, E3 ubiquitin prot	HUWE1	Cluster0003
15042613	XM_001491121 // IARS // isoleucyl-tRNA synthetase // --- // 100054721 /// ENSECAT000000	IARS	Cluster0003
15075040	XM_001491469 // IGF2R // insulin-like growth factor 2 receptor // --- // 100058539 ///	IGF2R	Cluster0003
15051731	XM_001492506 // IKBKAP // inhibitor of kappa light polypeptide gene enhancer in B-cells	IKBKAP	Cluster0003
15084003	ENSECAT00000000739 // IKBKE // inhibitor of kappa light polypeptide gene enhancer in B-	IKBKE	Cluster0003
15077128	ENSECAT00000019692 // IKZF1 // IKAROS family zinc finger 1 (Ikaros) // --- // 100052437	IKZF1	Cluster0003
15116025	ENSECAT00000021625 // IMPACT // Impact homolog (mouse) // --- // ---	IMPACT	Cluster0003
14991652	XM_003363011 // INPP4A // inositol polyphosphate-4-phosphatase, type I, 107kDa // --- /	INPP4A	Cluster0003
14941354	XM_001496265 // INPP5F // inositol polyphosphate-5-phosphatase F // --- // 100065762 //	INPP5F	Cluster0003
14961511	ENSECAT00000022612 // INTS2 // integrator complex subunit 2 // --- // 100071151 /// XM_	INTS2	Cluster0003
15035226	XM_001493727 // IPO11 // importin 11 // --- // 100050828 /// ENSECAT00000015335 // IPO1	IPO11	Cluster0003
15101117	XM_001503000 // IPO8 // importin 8 // --- // 100064845 /// ENSECAT00000014160 // IPO8 /	IPO8	Cluster0003
14987340	XM_001503963 // IQGAP2 // IQ motif containing GTPase activating protein 2 // --- // 100	IQGAP2	Cluster0003
15068603	ENSECAT00000014564 // ITFG1 // integrin alpha FG-GAP repeat containing 1 // --- // 1000	ITFG1	Cluster0003
15007561	ENSECAT00000011502 // ITGA4 // integrin, alpha 4 (antigen CD49D, alpha 4 subunit of VLA	ITGA4	Cluster0003
14979289	EU881921 // ITGAL // integrin, alpha L (antigen CD11A (p180), lymphocyte function-assoc	ITGAL	Cluster0003
15100980	ENSECAT00000009891 // ITPR2 // inositol 1,4,5-trisphosphate receptor, type 2 // --- //	ITPR2	Cluster0003
15083149	XM_001496508 // JHDM1D // jumonji C domain containing histone demethylase 1 homolog D (	JHDM1D	Cluster0003
15024455	ENSECAT00000012981 // KCNAB2 // potassium voltage-gated channel, shaker-related subfami	KCNAB2	Cluster0003
15026924	XM_001496578 // KDM1B // lysine (K)-specific demethylase 1B // --- // 100066240 /// ENS	KDM1B	Cluster0003
15042028	XM_001492896 // KDM4C // lysine (K)-specific demethylase 4C // --- // 100060699 /// ENS	KDM4C	Cluster0003
15095372	ENSECAT00000023294 // KDM5A // lysine (K)-specific demethylase 5A // --- // 100056935 /	KDM5A	Cluster0003
15120541	ENSECAT00000021754 // KDSR // 3-ketodihydrosphingosine reductase // --- // ---	KDSR	Cluster0003
14952852	ENSECAT00000023234 // KIAA1919 // KIAA1919 // --- // ---	KIAA1919	Cluster0003
15071905	ENSECAT00000009334 // KLHL5 // kelch-like 5 (Drosophila) // --- // 100064472 /// XM_001	KLHL5	Cluster0003
15006628	ENSECAT00000019639 // KYNU // kynureninase // --- // 100057449 /// XM_001490785 // KYNU	KYNU	Cluster0003
14952686	ENSECAT00000025234 // LACE1 // lactation elevated 1 // --- // 100066538 /// XM_00150398	LACE1	Cluster0003
14982416	XM_001503913 // LARS // leucyl-tRNA synthetase // --- // 100061092 /// ENSECAT000000101	LARS	Cluster0003
15003307	XM_001490588 // LCP1 // lymphocyte cytosolic protein 1 (L-plastin) // --- // 100050663	LCP1	Cluster0003
15016572	XM_001497401 // LEPRE1 // leucine proline-enriched proteoglycan (leprecan) 1 // --- //	LEPRE1	Cluster0003
15068422	XM_001488446 // LETM1 // leucine zipper-EF-hand containing transmembrane protein 1 // -	LETM1	Cluster0003
14966954	ENSECAT00000017866 // LIG3 // ligase III, DNA, ATP-dependent // --- // 100071671 /// XM	LIG3	Cluster0003
15101941	ENSECAT00000012390 // LIMA1 // LIM domain and actin binding 1 // --- // 100060033	LIMA1	Cluster0003
15083766	ENSECAT00000026308 // LMBR1 // limb region 1 homolog (mouse) // --- // 100066736 /// XM	LMBR1	Cluster0003
14941034	XM_001488425 // LOC100049801 // methylated-DNA--protein-cysteine methyltransferase-like	LOC100049801	Cluster0003
15015651	XM_001488028 // LOC100049896 // lipid phosphate phosphohydrolase 3-like // --- // 10004	LOC100049896	Cluster0003
15065811	ENSECAT00000020893 // LOC100050042 // trafficking protein particle complex subunit 2-li	LOC100050042	Cluster0003
15002536	XM_003363190 // LOC100050287 // transcription elongation factor A protein-like 8-like /	LOC100050287	Cluster0003
14980087	ENSECAT00000009379 // LOC100050421 // nuclear distribution protein nudE homolog 1-like	LOC100050421	Cluster0003
15084669	XM_001487852 // LOC100050551 // sterol O-acyltransferase 1-like // --- // 100050551 ///	LOC100050551	Cluster0003
14976819	ENSECAT00000019299 // LOC100050655 // poly(A)-specific ribonuclease PARN-like // --- //	LOC100050655	Cluster0003
15006280	XM_001488507 // LOC100050708 // TFIIH basal transcription factor complex helicase XPB s	LOC100050708	Cluster0003
15055539	XM_001491140 // LOC100050760 // cystatin-B-like // --- // 100050760 /// ENSECAT00000014	LOC100050760	Cluster0003
15059541	ENSECAT00000021915 // LOC100050914 // gamma-parvin-like // --- // 100050914 /// XM_0014	LOC100050914	Cluster0003
15085249	XM_001915520 // LOC100050924 // transmembrane protein 206-like // --- // 100050924 ///	LOC100050924	Cluster0003

15002158	XM_001492309 // LOC100051043 // phospholipid scramblase 2-like // --- // 100051043 ///	LOC100051043	Cluster0003
15030409	XM_001491453 // LOC100051158 // putative pre-mRNA-splicing factor ATP-dependent RNA hel	LOC100051158	Cluster0003
15070135	XM_001488069 // LOC100051284 // n-acetylgalactosamine-6-sulfatase-like // --- // 100051	LOC100051284	Cluster0003
14944257	ENSECAT00000007871 // LOC100051390 // AN1-type zinc finger and ubiquitin domain-contain	LOC100051390	Cluster0003
15090249	XM_001915744 // LOC100051433 // digestive organ expansion factor homolog // --- // 1000	LOC100051433	Cluster0003
15044562	XM_001497949 // LOC100051488 // CDK-activating kinase assembly factor MAT1-like // ---	LOC100051488	Cluster0003
14992859	XM_003362988 // LOC100051543 // UTP--glucose-1-phosphate uridylyltransferase-like // --	LOC100051543	Cluster0003
15097539	XM_003365259 // LOC100051662 // diacylglycerol kinase alpha-like // --- // 100051662 //	LOC100051662	Cluster0003
15030919	ENSECAT00000005280 // LOC100051758 // pre-B-cell leukemia transcription factor 2-like /	LOC100051758	Cluster0003
15053979	ENSECAT00000024598 // LOC100051829 // protein dopey-2-like // --- // 100051829	LOC100051829	Cluster0003
15127262	ENSECAT00000018780 // LOC100051857 // spermine synthase-like // --- // 100051857 /// XM	LOC100051857	Cluster0003
15064179	ENSECAT00000005664 // LOC100051892 // nuclear pore complex protein Nup93-like // --- //	LOC100051892	Cluster0003
14940205	XM_001491019 // LOC100051911 // DDB1- and CUL4-associated factor 11-like // --- // 1000	LOC100051911	Cluster0003
15103243	ENSECAT00000000800 // LOC100051965 // nuclear protein MDM1-like // --- // 100051965	LOC100051965	Cluster0003
15033271	XM_001916390 // LOC100052007 // ATP-dependent RNA helicase DHX29-like // --- // 1000520	LOC100052007	Cluster0003
14988441	ENSECAT00000007417 // LOC100052052 // tubulin--tyrosine ligase-like // --- // 100052052	LOC100052052	Cluster0003
15098273	ENSECAT00000014580 // LOC100052084 // e3 ubiquitin-protein ligase Mdm2-like // --- // 1	LOC100052084	Cluster0003
15107038	XM_001498480 // LOC100052144 // mitochondrial uncoupling protein 2-like // --- // 10005	LOC100052144	Cluster0003
15132128	ENSECAT00000019797 // LOC100052401 // histone deacetylase 8-like // --- // 100052401 //	LOC100052401	Cluster0003
15102879	XM_001488356 // LOC100052445 // retinol dehydrogenase 16-like // --- // 100052445 /// E	LOC100052445	Cluster0003
15095662	XM_001497051 // LOC100052502 // t-cell surface glycoprotein CD4-like // --- // 10005250	LOC100052502	Cluster0003
14944475	ENSECAT00000014974 // LOC100052580 // protein FAM190B-like // --- // 100052580 /// XM_0	LOC100052580	Cluster0003
15035571	XM_001498159 // LOC100052705 // NAD(P) transhydrogenase, mitochondrial-like // --- // 1	LOC100052705	Cluster0003
15052116	XM_001488664 // LOC100052763 // delta-aminolevulinic acid dehydratase-like // --- // 10	LOC100052763	Cluster0003
15005451	ENSECAT00000013234 // LOC100053014 // probable E3 ubiquitin-protein ligase MYCBP2-like	LOC100053014	Cluster0003
15113390	ENSECAT00000015042 // LOC100053038 // cytospin-A-like // --- // 100053038 /// XM_001488	LOC100053038	Cluster0003
14937447	ENSECAT00000011730 // LOC100053293 // poly [ADP-ribose] polymerase 16-like // --- // 10	LOC100053293	Cluster0003
15004337	XM_001488643 // LOC100053345 // paraspeckle component 1-like // --- // 100053345 /// EN	LOC100053345	Cluster0003
15027124	ENSECAT00000026969 // LOC100053350 // hereditary hemochromatosis protein homolog // ---	LOC100053350	Cluster0003
14937499	XM_001498388 // LOC100053578 // maspardin-like // --- // 100053578 /// ENSECAT000000135	LOC100053578	Cluster0003
14959310	XM_001917124 // LOC100053831 // testis-expressed sequence 2 protein-like // --- // 1000	LOC100053831	Cluster0003
14993476	ENSECAT00000015307 // LOC100054079 // aldose 1-epimerase-like // --- // 100054079 /// X	LOC100054079	Cluster0003
15073336	ENSECAT00000019881 // LOC100054203 // MOSC domain-containing protein 1, mitochondrial-l	LOC100054203	Cluster0003
15019752	XM_003364471 // LOC100054410 // dihydropyrimidinase-related protein 2-like // --- // 10	LOC100054410	Cluster0003
15099561	ENSECAT00000015117 // LOC100054664 // uncharacterized LOC100054664 // --- // 100054664	LOC100054664	Cluster0003
14990261	ENSECAT00000015187 // LOC100054688 // xanthine dehydrogenase/oxidase-like // --- // 100	LOC100054688	Cluster0003
15048891	XM_001915559 // LOC100054700 // inversin-like // --- // 100054700 /// ENSECAT0000001006	LOC100054700	Cluster0003
15026521	ENSECAT00000015223 // LOC100054792 // dual specificity protein phosphatase 22-like // -	LOC100054792	Cluster0003
15028672	ENSECAT00000021875 // LOC100054835 // uncharacterized protein KIAA0240-like // --- // 1	LOC100054835	Cluster0003
14951027	XM_001917290 // LOC100054907 // u1 small nuclear ribonucleoprotein 70 kDa-like // --- /	LOC100054907	Cluster0003
15041680	ENSECAT00000021328 // LOC100054931 // retinal dehydrogenase 1-like // --- // 100054931	LOC100054931	Cluster0003
15086910	XM_001490599 // LOC100054991 // cathepsin K-like // --- // 100054991 /// ENSECAT0000002	LOC100054991	Cluster0003
14940154	XM_001489375 // LOC100054994 // dehydrogenase/reductase SDR family member 4-like // ---	LOC100054994	Cluster0003
15028781	XM_001501854 // LOC100055106 // cullin-9-like // --- // 100055106 /// ENSECAT0000001283	LOC100055106	Cluster0003
15017014	ENSECAT00000014327 // LOC100055411 // dyslexia-associated protein KIAA0319-like protein	LOC100055411	Cluster0003
15086704	XM_001916034 // LOC100055681 // thioesterase superfamily member 4-like // --- // 100055	LOC100055681	Cluster0003
14980179	ENSECAT00000020766 // LOC100055746 // sorting nexin-29-like // --- // 100055746	LOC100055746	Cluster0003
14980193	ENSECAT00000021317 // LOC100055746 // sorting nexin-29-like // --- // 100055746 /// XM_	LOC100055746	Cluster0003
14994882	ENSECAT00000014207 // LOC100055795 // sodium- and chloride-dependent taurine transporte	LOC100055795	Cluster0003
15092686	XM_001489930 // LOC100056015 // UDP-N-acetylglucosamine transporter-like // --- // 1000	LOC100056015	Cluster0003
14958128	ENSECAT00000014077 // LOC100056075 // putative sodium-coupled neutral amino acid transp	LOC100056075	Cluster0003
15045187	XM_001489871 // LOC100056126 // acyl-coenzyme A thioesterase 6-like // --- // 100056126	LOC100056126	Cluster0003
14940444	XM_001490020 // LOC100056157 // iron-sulfur protein NUBPL-like // --- // 100056157 ///	LOC100056157	Cluster0003
14964072	XM_001489603 // LOC100056244 // regulatory-associated protein of mTOR-like // --- // 10	LOC100056244	Cluster0003

14971876	ENSECAT00000016112 // LOC100056318 // AP-2 complex subunit alpha-2-like // --- // 10005	LOC100056318	Cluster0003
15090278	ENSECAT00000020548 // LOC100056429 // corticosteroid 11-beta-dehydrogenase isozyme 1-li	LOC100056429	Cluster0003
14963811	ENSECAT00000011318 // LOC100056490 // tubulin-specific chaperone D-like // --- // 10005	LOC100056490	Cluster0003
14958192	XM_001916620 // LOC100056499 // n-sulphoglucosamine sulphohydrolase-like // --- // 1000	LOC100056499	Cluster0003
15044288	XM_001490223 // LOC100056508 // protein Smaug homolog 1-like // --- // 100056508 /// EN	LOC100056508	Cluster0003
15114527	ENSECAT00000014088 // LOC100056569 // ATP-dependent RNA helicase DDX54-like // --- // 1	LOC100056569	Cluster0003
15057415	ENSECAT00000018058 // LOC100056687 // cytochrome P450 4V2-like // --- // 100056687 ///	LOC100056687	Cluster0003
15078669	XM_001502591 // LOC100056692 // staphylococcal nuclease domain-containing protein 1-lik	LOC100056692	Cluster0003
15057426	ENSECAT00000022544 // LOC100056739 // protein FAM149A-like // --- // 100056739 /// XM_0	LOC100056739	Cluster0003
15054439	XM_001490477 // LOC100056927 // 6-phosphofructokinase, liver type-like // --- // 100056	LOC100056927	Cluster0003
15114570	ENSECAT00000025656 // LOC100056981 // probable E3 ubiquitin-protein ligase C12orf51-lik	LOC100056981	Cluster0003
15030316	XM_001490546 // LOC100057057 // ubiquitin D-like // --- // 100057057 /// ENSECAT0000000	LOC100057057	Cluster0003
15054376	ENSECAT00000015988 // LOC100057131 // trafficking protein particle complex subunit 10-l	LOC100057131	Cluster0003
14932561	ENSECAT00000007942 // LOC100057314 // protein FAM53B-like // --- // 100057314	LOC100057314	Cluster0003
15095425	ENSECAT00000022092 // LOC100057420 // peptidyl-prolyl cis-trans isomerase FKBP4-like //	LOC100057420	Cluster0003
15108234	ENSECAT00000007548 // LOC100057503 // growth arrest-specific protein 2-like // --- // 1	LOC100057503	Cluster0003
15123100	XM_001489456 // LOC100057538 // fatty acid-binding protein, epidermal-like // --- // 10	LOC100057538	Cluster0003
15044163	ENSECAT00000011672 // LOC100057755 // constitutive coactivator of PPAR-gamma-like prote	LOC100057755	Cluster0003
15044359	XM_001491002 // LOC100057780 // UPF0679 protein C14orf101-like // --- // 100057780 ///	LOC100057780	Cluster0003
15026566	ENSECAT00000008719 // LOC100057868 // ribosyldihydronicotinamide dehydrogenase [quinone	LOC100057868	Cluster0003
15092551	XM_001493212 // LOC100057881 // protein FAM102B-like // --- // 100057881 /// ENSECAT000	LOC100057881	Cluster0003
15057059	XM_001493791 // LOC100057973 // erlin-2-like // --- // 100057973 /// ENSECAT00000010647	LOC100057973	Cluster0003
15086056	XM_001500345 // LOC100057984 // G patch domain-containing protein 4-like // --- // 1000	LOC100057984	Cluster0003
15045332	ENSECAT00000010806 // LOC100058017 // feline leukemia virus subgroup C receptor-related	LOC100058017	Cluster0003
15046218	ENSECAT00000017777 // LOC100058101 // cytoplasmic dynein 1 heavy chain 1-like // --- //	LOC100058101	Cluster0003
15071400	XM_001916811 // LOC100058152 // RE1-silencing transcription factor-like // --- // 10005	LOC100058152	Cluster0003
15133906	ENSECAT00000011997 // LOC100058256 // filamin-A-like // --- // 100058256	LOC100058256	Cluster0003
15118640	XM_001915599 // LOC100058510 // calcium/calmodulin-dependent protein kinase kinase 2-li	LOC100058510	Cluster0003
15085680	ENSECAT00000022399 // LOC100058672 // SLAM family member 6-like // --- // 100058672 ///	LOC100058672	Cluster0003
14958470	ENSECAT00000014219 // LOC100058914 // phosphoribosyl pyrophosphate synthase-associated	LOC100058914	Cluster0003
15087488	XM_001917840 // LOC100059247 // tyrosine-protein phosphatase non-receptor type 22-like	LOC100059247	Cluster0003
15092846	ENSECAT00000010453 // LOC100059248 // protein Dr1-like // --- // 100059248 /// XM_00149	LOC100059248	Cluster0003
15071516	XM_001916943 // LOC100059582 // probable polyprenol reductase-like // --- // 100059582	LOC100059582	Cluster0003
14973090	ENSECAT00000019275 // LOC100059672 // cytochrome b ascorbate-dependent protein 3-like /	LOC100059672	Cluster0003
15114116	ENSECAT00000000808 // LOC100059916 // chemokine-like receptor 1-like // --- // 10005991	LOC100059916	Cluster0003
15100129	ENSECAT00000014889 // LOC100060326 // complement C1r subcomponent-like protein-like //	LOC100060326	Cluster0003
14967823	ENSECAT00000017441 // LOC100060346 // protein KIAA0664-like // --- // 100060346	LOC100060346	Cluster0003
14935396	XM_001916677 // LOC100060551 // egl nine homolog 1-like // --- // 100060551 /// ENSECAT	LOC100060551	Cluster0003
14933472	ENSECAT00000019497 // LOC100060589 // erlin-1-like // --- // 100060589 /// XM_001500565	LOC100060589	Cluster0003
15056490	XM_001492844 // LOC100060636 // N(4)-(beta-N-acetylglucosaminyl)-L-asparaginase-like //	LOC100060636	Cluster0003
15119586	XM_001492917 // LOC100060744 // collectin-12-like // --- // 100060744 /// ENSECAT000000	LOC100060744	Cluster0003
15115032	XM_001915436 // LOC100061102 // ATP-dependent RNA helicase DDX55-like // --- // 1000611	LOC100061102	Cluster0003
15066517	ENSECAT00000013095 // LOC100061199 // 4-hydroxybenzoate polyprenyltransferase, mitochon	LOC100061199	Cluster0003
15021462	XM_001493315 // LOC100061338 // non-specific lipid-transfer protein-like // --- // 1000	LOC100061338	Cluster0003
15019145	ENSECAT00000024692 // LOC100061432 // NAD kinase-like // --- // 100061432	LOC100061432	Cluster0003
15000218	XM_001493395 // LOC100061475 // MAP kinase-activated protein kinase 3-like // --- // 10	LOC100061475	Cluster0003
14992741	ENSECAT00000009486 // LOC100061509 // rho GTPase-activating protein 25-like // --- // 1	LOC100061509	Cluster0003
15109487	ENSECAT00000020755 // LOC100061658 // alkylated DNA repair protein alkB homolog 8-like	LOC100061658	Cluster0003
15104243	ENSECAT00000025920 // LOC100061694 // ras-related protein Rab-39A-like // --- // 100061	LOC100061694	Cluster0003
14982598	ENSECAT00000023878 // LOC100061748 // histidyl-tRNA synthetase, cytoplasmic-like // ---	LOC100061748	Cluster0003
15045526	XM_001495194 // LOC100061785 // psychosine receptor-like // --- // 100061785 /// ENSECA	LOC100061785	Cluster0003
15057139	ENSECAT00000000424 // LOC100062021 // Werner syndrome ATP-dependent helicase-like // --	LOC100062021	Cluster0003
14982677	ENSECAT00000018338 // LOC100062046 // transmembrane protein 173-like // --- // 10006204	LOC100062046	Cluster0003
15075308	XM_001493771 // LOC100062058 // connector enhancer of kinase suppressor of ras 3-like /	LOC100062058	Cluster0003

15072895	XM_001493822 // LOC100062135 // adenylosuccinate synthetase isozyme 2-like // --- // 10	LOC100062135	Cluster0003
15097325	XM_001504534 // LOC100062193 // RISC-loading complex subunit TARBP2-like // --- // 1000	LOC100062193	Cluster0003
15003918	ENSECAT00000020134 // LOC100062251 // propionyl-CoA carboxylase alpha chain, mitochondr	LOC100062251	Cluster0003
15109872	XM_001502701 // LOC100062824 // FXYD domain-containing ion transport regulator 6-like /	LOC100062824	Cluster0003
15073695	ENSECAT00000013381 // LOC100062957 // calmodulin-regulated spectrin-associated protein	LOC100062957	Cluster0003
14945520	ENSECAT00000013562 // LOC100063100 // cytoplasmic FMR1-interacting protein 1-like // --	LOC100063100	Cluster0003
15020688	XM_001502431 // LOC100063257 // histone-lysine N-methyltransferase SETD7-like // --- //	LOC100063257	Cluster0003
15056160	XM_001494597 // LOC100063334 // malignant fibrous histiocytoma-amplified sequence 1-lik	LOC100063334	Cluster0003
15016135	XM_001494616 // LOC100063364 // spermatogenesis-associated protein 6-like // --- // 100	LOC100063364	Cluster0003
15047903	ENSECAT00000007937 // LOC100063370 // thyroid receptor-interacting protein 11-like // -	LOC100063370	Cluster0003
15117667	ENSECAT00000022284 // LOC100063639 // transcobalamin-2-like // --- // 100063639 /// XM_	LOC100063639	Cluster0003
14943387	ENSECAT00000018865 // LOC100063651 // calcium uptake protein 1, mitochondrial-like // -	LOC100063651	Cluster0003
15026054	ENSECAT00000020736 // LOC100063889 // uncharacterized protein KIAA1109-like // --- // 1	LOC100063889	Cluster0003
15088397	ENSECAT00000026954 // LOC100063932 // box C/D snoRNA protein 1-like // --- // 100063932	LOC100063932	Cluster0003
15066279	ENSECAT00000020707 // LOC100063998 // protein FAM13A-like // --- // 100063998	LOC100063998	Cluster0003
15091003	XM_001500143 // LOC100064158 // ammonium transporter Rh type B-like // --- // 100064158	LOC100064158	Cluster0003
14986709	ENSECAT00000010439 // LOC100064643 // tyrosine-protein kinase Fer-like // --- // 100064	LOC100064643	Cluster0003
15083233	XM_001498241 // LOC100064670 // 28S ribosomal protein S33, mitochondrial-like // --- //	LOC100064670	Cluster0003
15085965	ENSECAT00000024151 // LOC100064689 // rho guanine nucleotide exchange factor 11-like //	LOC100064689	Cluster0003
14988383	XM_001916541 // LOC100064727 // tyrosine-protein kinase Mer-like // --- // 100064727 //	LOC100064727	Cluster0003
14946085	ENSECAT00000008438 // LOC100064749 // membrane progestin receptor gamma-like // --- //	LOC100064749	Cluster0003
15083683	ENSECAT00000024323 // LOC100064812 // histone-lysine N-methyltransferase MLL3-like // -	LOC100064812	Cluster0003
14992917	ENSECAT00000006018 // LOC100064970 // activator of 90 kDa heat shock protein ATPase hom	LOC100064970	Cluster0003
15091832	XM_001497170 // LOC100064990 // chromodomain-helicase-DNA-binding protein 1-like // ---	LOC100064990	Cluster0003
15079049	ENSECAT00000010428 // LOC100064995 // nuclear pore complex protein Nup205-like // --- /	LOC100064995	Cluster0003
14952125	ENSECAT00000021015 // LOC100065059 // protein MTO1 homolog, mitochondrial-like // --- /	LOC100065059	Cluster0003
15029823	XM_001495773 // LOC100065066 // coiled-coil domain-containing protein 90A, mitochondria	LOC100065066	Cluster0003
15000661	ENSECAT00000010050 // LOC100065601 // phosphatidylinositide phosphatase SAC1-like // --	LOC100065601	Cluster0003
15029832	ENSECAT00000013221 // LOC100065604 // dysbindin-like // --- // 100065604 /// XM_0014961	LOC100065604	Cluster0003
14987488	XM_001918348 // LOC100065643 // u3 small nucleolar RNA-associated protein 15 homolog //	LOC100065643	Cluster0003
14940762	XM_001916065 // LOC100065920 // ADP-ribosylation factor 6-like // --- // 100065920 ///	LOC100065920	Cluster0003
15094888	XM_001915910 // LOC100065931 // arf-GAP with GTPase, ANK repeat and PH domain-containin	LOC100065931	Cluster0003
14932731	XM_001493741 // LOC100065967 // sideroflexin-4-like // --- // 100065967 /// ENSECAT0000	LOC100065967	Cluster0003
14944547	XM_001496540 // LOC100066171 // UPF0765 protein C10orf58-like // --- // 100066171 /// E	LOC100066171	Cluster0003
15005988	ENSECAT00000003176 // LOC100066322 // ras-related protein Rab-20-like // --- // 1000663	LOC100066322	Cluster0003
15036910	ENSECAT00000019406 // LOC100066493 // uncharacterized protein C20orf194-like // --- //	LOC100066493	Cluster0003
15025173	ENSECAT00000010768 // LOC100066545 // n-acetylgalactosaminyltransferase 7-like // --- /	LOC100066545	Cluster0003
15066160	ENSECAT00000012933 // LOC100066636 // RNA (guanine-9-)-methyltransferase domain-contain	LOC100066636	Cluster0003
14979469	ENSECAT00000021542 // LOC100066772 // uncharacterized protein KIAA0556-like // --- // 1	LOC100066772	Cluster0003
15072037	ENSECAT00000012364 // LOC100066908 // TBC1 domain family member 19-like // --- // 10006	LOC100066908	Cluster0003
14937572	XM_001918045 // LOC100067089 // probable E3 ubiquitin-protein ligase HERC1-like // ---	LOC100067089	Cluster0003
15052631	XM_001917241 // LOC100067215 // target of rapamycin complex 2 subunit MAPKAP1-like // -	LOC100067215	Cluster0003
15076431	ENSECAT00000008422 // LOC100067266 // androgen-induced gene 1 protein-like // --- // 10	LOC100067266	Cluster0003
14988729	XM_001498891 // LOC100067312 // vitamin K-dependent gamma-carboxylase-like // --- // 10	LOC100067312	Cluster0003
14988752	XM_001497450 // LOC100067379 // all-trans-retinol 13,14-reductase-like // --- // 100067	LOC100067379	Cluster0003
15049552	ENSECAT00000019641 // LOC100067385 // rab GTPase-activating protein 1-like // --- // 10	LOC100067385	Cluster0003
14957520	ENSECAT00000014308 // LOC100067494 // band 4.1-like protein 2-like // --- // 100067494	LOC100067494	Cluster0003
14997217	XM_001917025 // LOC100067713 // serine/threonine-protein kinase ULK4-like // --- // 100	LOC100067713	Cluster0003
15104013	XM_001497953 // LOC100068054 // jerky protein homolog-like // --- // 100068054 /// ENSE	LOC100068054	Cluster0003
15031816	ENSECAT00000018747 // LOC100068070 // transcription initiation protein SPT3 homolog //	LOC100068070	Cluster0003
15063411	XM_001498147 // LOC100068285 // transmembrane protein 236-like // --- // 100068285 ///	LOC100068285	Cluster0003
14946518	ENSECAT00000020857 // LOC100068294 // protein FAM63B-like // --- // 100068294	LOC100068294	Cluster0003
14977362	XM_001498167 // LOC100068313 // ATP-binding cassette sub-family A member 3-like // ---	LOC100068313	Cluster0003
15100889	ENSECAT00000022411 // LOC100068403 // uncharacterized protein KIAA0528-like // --- // 1	LOC100068403	Cluster0003

14980587	XM_001498282 // LOC100068441 // putative N-acetylglucosamine-6-phosphate deacetylase-li	LOC100068441	Cluster0003
15108512	ENSECAT00000016245 // LOC100068522 // AP-3 complex subunit delta-1-like // --- // 10006	LOC100068522	Cluster0003
15037225	XM_001916263 // LOC100068557 // tyrosine-protein kinase HCK-like // --- // 100068557 //	LOC100068557	Cluster0003
15053657	XM_001498520 // LOC100068696 // multidrug resistance-associated protein 1-like // --- /	LOC100068696	Cluster0003
14979701	XM_001498635 // LOC100068821 // methyltransferase-like protein 9-like // --- // 1000688	LOC100068821	Cluster0003
14936106	ENSECAT00000007574 // LOC100069086 // isocitrate dehydrogenase [NADP], mitochondrial-li	LOC100069086	Cluster0003
15096425	ENSECAT00000011309 // LOC100069159 // 28S ribosomal protein S35, mitochondrial-like //	LOC100069159	Cluster0003
14989883	ENSECAT00000022521 // LOC100069170 // thyroid adenoma-associated protein homolog // ---	LOC100069170	Cluster0003
14989918	ENSECAT00000018766 // LOC100069189 // zinc finger protein 36, C3H1 type-like 2-like //	LOC100069189	Cluster0003
14960851	XM_001499043 // LOC100069268 // CDK5 regulatory subunit-associated protein 3-like // --	LOC100069268	Cluster0003
15109422	ENSECAT00000009016 // LOC100069290 // caspase-12-like // --- // 100069290 /// XM_001499	LOC100069290	Cluster0003
14977210	XM_001499300 // LOC100069561 // heat shock protein 75 kDa, mitochondrial-like // --- //	LOC100069561	Cluster0003
15130244	XM_001499390 // LOC100069653 // myotubularin-related protein 1-like // --- // 100069653	LOC100069653	Cluster0003
15050175	XM_001499639 // LOC100069929 // torsin-1B-like // --- // 100069929 /// ENSECAT000000089	LOC100069929	Cluster0003
15096496	ENSECAT00000023757 // LOC100070034 // dynamin-1-like protein-like // --- // 100070034 /	LOC100070034	Cluster0003
15061140	ENSECAT00000019625 // LOC100070283 // platelet-derived growth factor subunit B-like //	LOC100070283	Cluster0003
14954466	ENSECAT00000007577 // LOC100070296 // protein SMG9-like // --- // 100070296 /// XM_0014	LOC100070296	Cluster0003
14946927	XM_001918205 // LOC100070575 // sorbitol dehydrogenase-like // --- // 100070575 /// ENS	LOC100070575	Cluster0003
15023095	ENSECAT00000022834 // LOC100070978 // nuclear migration protein nudC-like // --- // 100	LOC100070978	Cluster0003
15049599	ENSECAT00000010586 // LOC100071002 // serine/threonine-protein kinase Nek6-like // ---	LOC100071002	Cluster0003
15078414	XM_001500729 // LOC100071043 // myoD family inhibitor domain-containing protein-like //	LOC100071043	Cluster0003
14956522	XM_001915421 // LOC100071075 // midasin-like // --- // 100071075 /// ENSECAT00000009278	LOC100071075	Cluster0003
14950827	XM_001500918 // LOC100071215 // SUMO-activating enzyme subunit 1-like // --- // 1000712	LOC100071215	Cluster0003
15013154	XM_001501232 // LOC100071466 // uncharacterized LOC100071466 // --- // 100071466 /// EN	LOC100071466	Cluster0003
15023348	ENSECAT00000021482 // LOC100071487 // calcipressin-3-like // --- // 100071487 /// XM_00	LOC100071487	Cluster0003
14961814	ENSECAT00000010739 // LOC100071750 // myosin-Id-like // --- // 100071750 /// XM_0015016	LOC100071750	Cluster0003
15049378	XM_001501609 // LOC100071754 // centriolin-like // --- // 100071754 /// ENSECAT00000024	LOC100071754	Cluster0003
14990953	XM_001918247 // LOC100072177 // neuroblastoma-amplified sequence-like // --- // 1000721	LOC100072177	Cluster0003
14956968	ENSECAT00000015226 // LOC100072238 // peptidyl-prolyl cis-trans isomerase-like 6-like /	LOC100072238	Cluster0003
15105346	ENSECAT00000011015 // LOC100072304 // sugar phosphate exchanger 2-like // --- // 100072	LOC100072304	Cluster0003
14957087	ENSECAT00000028978 // LOC100072399 // DNA polymerase zeta catalytic subunit-like // ---	LOC100072399	Cluster0003
14994537	XM_001502409 // LOC100072474 // TATA box-binding protein-associated factor RNA polymera	LOC100072474	Cluster0003
14982794	XM_001504289 // LOC100072599 // cell division cycle protein 23 homolog // --- // 100072	LOC100072599	Cluster0003
14985920	ENSECAT00000021976 // LOC100072674 // transforming growth factor-beta-induced protein i	LOC100072674	Cluster0003
14968040	ENSECAT00000019214 // LOC100072696 // zinc finger ZZ-type and EF-hand domain-containing	LOC100072696	Cluster0003
14953059	ENSECAT00000010921 // LOC100073006 // probable E3 ubiquitin-protein ligase RNF217-like	LOC100073006	Cluster0003
15026455	ENSECAT00000006504 // LOC100073027 // aminoacyl tRNA synthase complex-interacting multi	LOC100073027	Cluster0003
14986301	XM_001503206 // LOC100073058 // phosphorylated adapter RNA export protein-like // --- /	LOC100073058	Cluster0003
14968881	ENSECAT00000027158 // LOC100073084 // protein SCO1 homolog, mitochondrial-like // --- /	LOC100073084	Cluster0003
14968906	ENSECAT00000027179 // LOC100073102 // zinc phosphodiesterase ELAC protein 2-like // ---	LOC100073102	Cluster0003
14983996	XM_003362887 // LOC100073268 // arylsulfatase B-like // --- // 100073268 /// ENSECAT000	LOC100073268	Cluster0003
14987582	ENSECAT00000010512 // LOC100073301 // methylcrotonoyl-CoA carboxylase beta chain, mitoc	LOC100073301	Cluster0003
15082921	XM_001914846 // LOC100146184 // WD repeat-containing protein 91-like // --- // 10014618	LOC100146184	Cluster0003
15079860	XM_003364904 // LOC100146250 // GTPase IMAP family member 5-like // --- // 100146250 //	LOC100146250	Cluster0003
15097736	ENSECAT00000018874 // LOC100146471 // retinol dehydrogenase 7-like // --- // 100146471	LOC100146471	Cluster0003
15091755	ENSECAT00000016795 // LOC100146677 // DNA-directed RNA polymerase III subunit RPC7-like	LOC100146677	Cluster0003
14955254	ENSECAT00000019105 // LOC100147304 // l-amino-acid oxidase-like // --- // 100147304 ///	LOC100147304	Cluster0003
15040413	XM_001916766 // LOC100147308 // fat storage-inducing transmembrane protein 2-like // --	LOC100147308	Cluster0003
15110725	ENSECAT00000010876 // LOC100147574 // leydig cell tumor 10 kDa protein homolog // --- /	LOC100147574	Cluster0003
14995313	XM_003363046 // LOC100629236 // eukaryotic translation initiation factor 4E type 3-like	LOC100629236	Cluster0003
15090318	ENSECAT00000023700 // LOC100629269 // c4b-binding protein alpha chain-like // --- // 10	LOC100629269	Cluster0003
15004651	ENSECAT00000001214 // LOC100629621 // ubiquitin-like protein 3-like // --- // 100629621	LOC100629621	Cluster0003
15017070	XM_003364413 // LOC100629945 // uncharacterized LOC100629945 // --- // 100629945 /// EN	LOC100629945	Cluster0003
15002296	ENSECAT00000024736 // LOC100630046 // p2Y purinoceptor 12-like // --- // 100630046 ///	LOC100630046	Cluster0003

15129681	ENSECAT00000025985 // LONRF3 // LON peptidase N-terminal domain and ring finger 3 // --	LONRF3	Cluster0003
15105697	ENSECAT00000023701 // LPHN1 // latrophilin 1 // --- // --- /// ENSECAT00000023703 // LP	LPHN1	Cluster0003
15119641	ENSECAT00000007256 // LPIN2 // lipin 2 // --- // 100051300	LPIN2	Cluster0003
15097754	ENSECAT00000013743 // LRP1 // low density lipoprotein receptor-related protein 1 // ---	LRP1	Cluster0003
14989846	ENSECAT00000025535 // LRPPRC // leucine-rich pentatricopeptide repeat containing // ---	LRPPRC	Cluster0003
14936403	XM_001489861 // LRRK1 // leucine-rich repeat kinase 1 // --- // 100050271 /// ENSECAT00	LRRK1	Cluster0003
15022334	ENSECAT00000017611 // MACF1 // microtubule-actin crosslinking factor 1 // --- // 100068	MACF1	Cluster0003
14969794	XM_003362595 // MADD // MAP-kinase activating death domain // --- // 100051327 /// ENSE	MADD	Cluster0003
14957335	XM_001502930 // MAN1A1 // mannosidase, alpha, class 1A, member 1 // --- // 100072869 //	MAN1A1	Cluster0003
15035333	ENSECAT00000007112 // MAP3K1 // mitogen-activated protein kinase kinase kinase 1, E3 ub	MAP3K1	Cluster0003
14989961	XM_001499556 // MAP4K3 // mitogen-activated protein kinase kinase kinase kinase 3 // --	MAP4K3	Cluster0003
15028238	ENSECAT00000015735 // MAPK14 // mitogen-activated protein kinase 14 // --- // 100063532	MAPK14	Cluster0003
14979415	ENSECAT00000015512 // MAPK3 // mitogen-activated protein kinase 3 // --- // 100066173 /	MAPK3	Cluster0003
14998572	XM_001488737 // MBNL1 // muscleblind-like splicing regulator 1 // --- // 100050018 ///	MBNL1	Cluster0003
15004168	ENSECAT00000018395 // MCF2L // MCF.2 cell line derived transforming sequence-like // --	MCF2L	Cluster0003
15055672	ENSECAT00000020592 // MCM3AP // minichromosome maintenance complex component 3 associat	MCM3AP	Cluster0003
14936314	ENSECAT00000024451 // MCTP2 // multiple C2 domains, transmembrane 2 // --- // 100049873	MCTP2	Cluster0003
15017433	XM_001503934 // MECR // mitochondrial trans-2-enoyl-CoA reductase // --- // 100070598 /	MECR	Cluster0003
15094444	ENSECAT00000005458 // MFF // mitochondrial fission factor // --- // 100056853	MFF	Cluster0003
15068519	XM_001488157 // MFSD7 // major facilitator superfamily domain containing 7 // --- // 10	MFSD7	Cluster0003
15006466	XM_001489097 // MGAT5 // mannosyl (alpha-1,6-)-glycoprotein beta-1,6-N-acetyl-glucosami	MGAT5	Cluster0003
15076622	XM_001915160 // MLL5 // myeloid/lymphoid or mixed-lineage leukemia 5 (trithorax homolog	MLL5	Cluster0003
15063383	XM_001497471 // MRC1 // mannose receptor, C type 1 // --- // 100068247 /// ENSECAT00000	MRC1	Cluster0003
15044756	XM_001499131 // MTHFD1 // methylenetetrahydrofolate dehydrogenase (NADP+ dependent) 1,	MTHFD1	Cluster0003
15076110	ENSECAT00000001146 // MTHFD1L // methylenetetrahydrofolate dehydrogenase (NADP+ depende	MTHFD1L	Cluster0003
15130232	ENSECAT00000016970 // MTM1 // myotubularin 1 // --- // 100069680 /// XM_001505085 // MT	MTM1	Cluster0003
15018568	ENSECAT00000017925 // MTOR // mechanistic target of rapamycin (serine/threonine kinase)	MTOR	Cluster0003
14975955	XM_001501685 // MVP // major vault protein // --- // 100063984 /// ENSECAT00000027087 /	MVP	Cluster0003
14967469	XM_003362429 // MYO18A // myosin XVIIIA // --- // 100059638 /// ENSECAT00000022672 // M	MYO18A	Cluster0003
15106484	ENSECAT00000018321 // MYO1F // myosin IF // --- // --- /// ENSECAT00000018418 // MYO1F	MYO1F	Cluster0003
14937157	ENSECAT00000017832 // MYO9A // myosin IXA // --- // 100052350	MYO9A	Cluster0003
14987636	ENSECAT00000003247 // NAIP // NLR family, apoptosis inhibitory protein // --- // 100073	NAIP	Cluster0003
14969390	ENSECAT00000012721 // NAT10 // N-acetyltransferase 10 (GCN5-related) // --- // ---	NAT10	Cluster0003
15073786	ENSECAT00000016536 // NAV1 // neuron navigator 1 // --- // ---	NAV1	Cluster0003
14994258	XM_001503106 // NCOA1 // nuclear receptor coactivator 1 // --- // 100056042 /// XM_0033	NCOA1	Cluster0003
15121053	XM_001493237 // NCOA2 // nuclear receptor coactivator 2 // --- // 100051656 /// ENSECAT	NCOA2	Cluster0003
14935474	XM_001494039 // NCOA4 // nuclear receptor coactivator 4 // --- // 100062485 /// ENSECAT	NCOA4	Cluster0003
14937919	ENSECAT00000010394 // NEDD4 // neural precursor cell expressed, developmentally down-re	NEDD4	Cluster0003
14967157	ENSECAT00000009831 // NF1 // neurofibromin 1 // --- // 100071896	NF1	Cluster0003
15042382	XM_001499262 // NFX1 // nuclear transcription factor, X-box binding 1 // --- // 1000537	NFX1	Cluster0003
15064236	ENSECAT00000015396 // NLRC5 // NLR family, CARD domain containing 5 // --- // ---	NLRC5	Cluster0003
15082100	ENSECAT00000014494 // NOD1 // nucleotide-binding oligomerization domain containing 1 //	NOD1	Cluster0003
14991061	XM_001918262 // NOL10 // nucleolar protein 10 // --- // 100057037 /// ENSECAT0000001649	NOL10	Cluster0003
15018831	ENSECAT00000013774 // NOL9 // nucleolar protein 9 // --- // ---	NOL9	Cluster0003
15080078	ENSECAT00000007782 // NOM1 // nucleolar protein with MIF4G domain 1 // --- // ---	NOM1	Cluster0003
15068321	XM_001489809 // NOP14 // NOP14 nucleolar protein homolog (yeast) // --- // 100051519 //	NOP14	Cluster0003
15008186	XM_001497246 // NOP58 // NOP58 ribonucleoprotein homolog (yeast) // --- // 100067129 //	NOP58	Cluster0003
15087188	ENSECAT00000016738 // NOTCH2 // notch 2 // --- // 100067078 /// XM_001497204 // NOTCH2	NOTCH2	Cluster0003
15115708	XM_001490178 // NPC1 // Niemann-Pick disease, type C1 // --- // 100059175 /// ENSECAT00	NPC1	Cluster0003
15054820	NM_001256955 // NRIP1 // nuclear receptor interacting protein 1 // --- // 100054242 ///	NRIP1	Cluster0003
14984469	XM_001502429 // NSD1 // nuclear receptor binding SET domain protein 1 // --- // 1000585	NSD1	Cluster0003
14952873	XM_001502648 // NT5DC1 // 5 -nucleotidase domain containing 1 // --- // 100072637 /// E	NT5DC1	Cluster0003
15070651	ENSECAT00000020843 // NUDT9 // nudix (nucleoside diphosphate linked moiety X)-type moti	NUDT9	Cluster0003
14972534	XM_001491461 // NUP160 // nucleoporin 160kDa // --- // 100058528 /// ENSECAT00000023778	NUP160	Cluster0003

15050095	XM_001499796 // NUP188 // nucleoporin 188kDa // --- // 100070114 /// ENSECAT00000007360	NUP188	Cluster0003
15127006	ENSECAT00000015763 // OFD1 // oral-facial-digital syndrome 1 // --- // ---	OFD1	Cluster0003
15029439	ENSECAT00000025473 // OGFRL1 // opioid growth factor receptor-like 1 // --- // 10007108	OGFRL1	Cluster0003
15012355	ENSECAT00000026313 // OPA1 // optic atrophy 1 (autosomal dominant) // --- // 100059993	OPA1	Cluster0003
15059926	XM_001491827 // OSBPL8 // oxysterol binding protein-like 8 // --- // 100059086 /// ENSE	OSBPL8	Cluster0003
15114775	XM_001492153 // P2RX4 // purinergic receptor P2X, ligand-gated ion channel, 4 // --- //	P2RX4	Cluster0003
15114762	XM_001495572 // P2RX7 // purinergic receptor P2X, ligand-gated ion channel, 7 // --- //	P2RX7	Cluster0003
15016731	XM_001503450 // PABPC4 // poly(A) binding protein, cytoplasmic 4 (inducible form) // --	PABPC4	Cluster0003
15018328	ENSECAT00000023886 // PADI2 // peptidyl arginine deiminase, type II // --- // 100050025	PADI2	Cluster0003
15026760	XM_001491437 // PAK1IP1 // PAK1 interacting protein 1 // --- // 100051197 /// ENSECAT00	PAK1IP1	Cluster0003
15025216	ENSECAT00000024847 // PALLD // palladin, cytoskeletal associated protein // --- // 1000	PALLD	Cluster0003
15102698	XM_001504835 // PAN2 // PAN2 poly(A) specific ribonuclease subunit homolog (S. cerevisi	PAN2	Cluster0003
15036175	XM_001501188 // PAPD7 // PAP associated domain containing 7 // --- // 100071429 /// ENS	PAPD7	Cluster0003
15021189	XM_001503578 // PAPSS1 // 3 -phosphoadenosine 5 -phosphosulfate synthase 1 // --- // 10	PAPSS1	Cluster0003
14995999	ENSECAT00000004449 // PBRM1 // polybromo 1 // --- // 100051687	PBRM1	Cluster0003
14998166	XM_001496380 // PCCB // propionyl CoA carboxylase, beta polypeptide // --- // 100065950	PCCB	Cluster0003
14992663	ENSECAT00000016266 // PCYOX1 // prenylcysteine oxidase 1 // --- // ---	PCYOX1	Cluster0003
14941901	ENSECAT00000022542 // PDCD11 // programmed cell death 11 // --- // 100034019	PDCD11	Cluster0003
14953555	XM_001490241 // PEPD // peptidase D // --- // 100056540 /// ENSECAT00000025611 // PEPD	PEPD	Cluster0003
15010915	ENSECAT00000021411 // PGAP1 // post-GPI attachment to proteins 1 // --- // 100070459 //	PGAP1	Cluster0003
14967435	XM_001504186 // PHF12 // PHD finger protein 12 // --- // 100059567 /// ENSECAT000000176	PHF12	Cluster0003
15126240	XM_001914744 // PHF8 // PHD finger protein 8 // --- // 100060410 /// ENSECAT00000029154	PHF8	Cluster0003
15063854	NM_001163825 // PHKB // phosphorylase kinase, beta // --- // 100050040 /// ENSECAT00000	PHKB	Cluster0003
15116792	XM_001915101 // PHLPP1 // PH domain and leucine rich repeat protein phosphatase 1 // --	PHLPP1	Cluster0003
15080148	ENSECAT00000017074 // PHTF2 // putative homeodomain transcription factor 2 // --- // 10	PHTF2	Cluster0003
15072859	ENSECAT00000008955 // PIGG // phosphatidylinositol glycan anchor biosynthesis, class G	PIGG	Cluster0003
15001641	XM_001496778 // PIK3R4 // phosphoinositide-3-kinase, regulatory subunit 4 // --- // 100	PIK3R4	Cluster0003
14960712	XM_001501544 // PIP4K2B // phosphatidylinositol-5-phosphate 4-kinase, type II, beta //	PIP4K2B	Cluster0003
14959184	NM_001163878 // PITPNC1 // phosphatidylinositol transfer protein, cytoplasmic 1 // ---	PITPNC1	Cluster0003
14939209	XM_001918300 // PLCB2 // phospholipase C, beta 2 // --- // 100057315 /// ENSECAT0000002	PLCB2	Cluster0003
15065525	XM_001501998 // PLCG2 // phospholipase C, gamma 2 (phosphatidylinositol-specific) // --	PLCG2	Cluster0003
15001570	ENSECAT00000010359 // PLCL2 // phospholipase C-like 2 // --- // 100063395 /// XM_001917	PLCL2	Cluster0003
14955063	XM_001489167 // PLEKHA4 // pleckstrin homology domain containing, family A (phosphoinos	PLEKHA4	Cluster0003
15002129	XM_001493103 // PLOD2 // procollagen-lysine, 2-oxoglutarate 5-dioxygenase 2 // --- // 1	PLOD2	Cluster0003
15063306	XM_001496595 // PLXDC2 // plexin domain containing 2 // --- // 100055892 /// ENSECAT000	PLXDC2	Cluster0003
14988666	ENSECAT00000011191 // POLR1A // polymerase (RNA) I polypeptide A, 194kDa // --- // 1000	POLR1A	Cluster0003
14988448	XM_001495461 // POLR1B // polymerase (RNA) I polypeptide B, 128kDa // --- // 100052113	POLR1B	Cluster0003
14943692	XM_001503996 // POLR3A // polymerase (RNA) III (DNA directed) polypeptide A, 155kDa //	POLR3A	Cluster0003
15082609	ENSECAT00000009237 // POT1 // protection of telomeres 1 homolog (S. pombe) // --- // 10	POT1	Cluster0003
14993330	XM_003362999 // PPM1B // protein phosphatase, Mg2+/Mn2+ dependent, 1B // --- // 1000535	PPM1B	Cluster0003
15103134	ENSECAT00000009257 // PPM1H // protein phosphatase, Mg2+/Mn2+ dependent, 1H // --- // 1	PPM1H	Cluster0003
15066119	XM_001497487 // PPP3CA // protein phosphatase 3, catalytic subunit, alpha isozyme // --	PPP3CA	Cluster0003
14956862	XM_001501777 // PREP // prolyl endopeptidase // --- // 100071926 /// ENSECAT00000025507	PREP	Cluster0003
15083648	ENSECAT00000013580 // PRKAG2 // protein kinase, AMP-activated, gamma 2 non-catalytic su	PRKAG2	Cluster0003
14959145	XM_001494589 // PRKCA // protein kinase C, alpha // --- // 100063318 /// ENSECAT0000000	PRKCA	Cluster0003
15044590	XM_001498126 // PRKCH // protein kinase C, eta // --- // 100051560 /// ENSECAT000000147	PRKCH	Cluster0003
15013909	XM_001493497 // PRKCSH // protein kinase C substrate 80K-H // --- // 100057774 /// XM_0	PRKCSH	Cluster0003
15054677	XM_001489273 // PRMT2 // protein arginine methyltransferase 2 // --- // 100054798 /// E	PRMT2	Cluster0003
14943342	XM_001503764 // PSAP // prosaposin // --- // 100063520 /// ENSECAT00000023432 // PSAP /	PSAP	Cluster0003
15043637	NM_001082513 // PSIP1 // PC4 and SFRS1 interacting protein 1 // --- // 100034217 /// EN	PSIP1	Cluster0003
15124874	ENSECAT00000016200 // PTK2 // PTK2 protein tyrosine kinase 2 // --- // ---	PTK2	Cluster0003
15022885	ENSECAT00000012816 // PTPRU // protein tyrosine phosphatase, receptor type, U // --- //	PTPRU	Cluster0003
14989474	ENSECAT00000014833 // PUS10 // pseudouridylate synthase 10 // --- // 100052053	PUS10	Cluster0003
15036350	XM_001490567 // PYGB // phosphorylase, glycogen; brain // --- // 100057085 /// ENSECAT0	PYGB	Cluster0003

15075141	ENSECAT00000018574 // QKI // QKI, KH domain containing, RNA binding // --- // 100033866	QKI	Cluster0003
15000143	XM_001495092 // RAD54L2 // RAD54-like 2 (S. cerevisiae) // --- // 100052240 /// ENSECAT	RAD54L2	Cluster0003
14995024	XM_001492240 // RAF1 // v-raf-1 murine leukemia viral oncogene homolog 1 // --- // 1000	RAF1	Cluster0003
14948698	ENSECAT00000026965 // RALGAPA1 // Ral GTPase activating protein, alpha subunit 1 (catal	RALGAPA1	Cluster0003
15036443	XM_001489543 // RALGAPA2 // Ral GTPase activating protein, alpha subunit 2 (catalytic)	RALGAPA2	Cluster0003
14983173	ENSECAT00000022661 // RAPGEF6 // Rap guanine nucleotide exchange factor (GEF) 6 // ---	RAPGEF6	Cluster0003
14984827	XM_001503271 // RARS // arginyl-tRNA synthetase // --- // 100059383 /// ENSECAT00000026	RARS	Cluster0003
15084530	NM_001163972 // RASAL2 // RAS protein activator like 2 // --- // 100033986 /// ENSECAT0	RASAL2	Cluster0003
15040236	XM_001499515 // RBL1 // retinoblastoma-like 1 (p107) // --- // 100069786 /// ENSECAT000	RBL1	Cluster0003
15064045	XM_001492689 // RBL2 // retinoblastoma-like 2 (p130) // --- // 100060431 /// ENSECAT000	RBL2	Cluster0003
15114473	XM_001491957 // RBM19 // RNA binding motif protein 19 // --- // 100056226 /// ENSECAT00	RBM19	Cluster0003
15082669	ENSECAT00000008258 // RBM28 // RNA binding motif protein 28 // --- // 100071725 /// XM_	RBM28	Cluster0003
15000337	XM_001496699 // RBM6 // RNA binding motif protein 6 // --- // 100052591 /// ENSECAT0000	RBM6	Cluster0003
15003226	ENSECAT00000023524 // RCBTB1 // regulator of chromosome condensation (RCC1) and BTB (PO	RCBTB1	Cluster0003
15046321	ENSECAT00000012690 // RCOR1 // REST corepressor 1 // --- // 100058618 /// XM_001491522	RCOR1	Cluster0003
15018772	XM_001915530 // RERE // arginine-glutamic acid dipeptide (RE) repeats // --- // 1000519	RERE	Cluster0003
14988016	XM_001490099 // REV1 // REV1, polymerase (DNA directed) // --- // 100050347 /// ENSECAT	REV1	Cluster0003
14961776	ENSECAT00000016306 // RFFL // ring finger and FYVE-like domain containing E3 ubiquitin	RFFL	Cluster0003
15086797	ENSECAT00000026716 // RFX5 // regulatory factor X, 5 (influences HLA class II expressio	RFX5	Cluster0003
14937909	ENSECAT00000002772 // RFX7 // regulatory factor X, 7 // --- // 100054682 /// XM_0015007	RFX7	Cluster0003
15076646	ENSECAT00000019909 // RINT1 // RAD50 interactor 1 // --- // 100050455 /// XM_001914893	RINT1	Cluster0003
15026677	XM_001490360 // RIOK1 // RIO kinase 1 (yeast) // --- // 100050980 /// ENSECAT0000000957	RIOK1	Cluster0003
15089840	NM_001089586 // RNASEL // ribonuclease L (2 ,5 -oligoisoadenylate synthetase-dependent)	RNASEL	Cluster0003
15000360	ENSECAT00000024935 // RNF123 // ring finger protein 123 // --- // 100053010	RNF123	Cluster0003
15026945	ENSECAT00000002824 // RNF144B // ring finger protein 144B // --- // 100052124 /// XM_00	RNF144B	Cluster0003
14979202	ENSECAT00000011634 // RNF40 // ring finger protein 40, E3 ubiquitin protein ligase // -	RNF40	Cluster0003
15115781	NM_001163985 // ROCK1 // Rho-associated, coiled-coil containing protein kinase 1 // ---	ROCK1	Cluster0003
14956511	XM_001500697 // RRAGD // Ras-related GTP binding D // --- // 100071018 /// ENSECAT00000	RRAGD	Cluster0003
15091114	ENSECAT00000025221 // RUSC1 // RUN and SH3 domain containing 1 // --- // ---	RUSC1	Cluster0003
15114096	ENSECAT00000000594 // SART3 // squamous cell carcinoma antigen recognized by T cells 3	SART3	Cluster0003
15061763	ENSECAT00000024091 // SBF1 // SET binding factor 1 // --- // 100056491	SBF1	Cluster0003
15112648	ENSECAT00000025304 // SBF2 // SET binding factor 2 // --- // 100055769 /// ENSECAT00000	SBF2	Cluster0003
14945704	ENSECAT00000018533 // SCAPER // S-phase cyclin A-associated protein in the ER // --- //	SCAPER	Cluster0003
15067342	ENSECAT00000003387 // SCFD2 // sec1 family domain containing 2 // --- // 100060187 ///	SCFD2	Cluster0003
15029563	ENSECAT00000010506 // SERPINB1 // serpin peptidase inhibitor, clade B (ovalbumin), memb	SERPINB1	Cluster0003
15010479	ENSECAT00000012633 // SESTD1 // SEC14 and spectrin domains 1 // --- // 100067450 /// XM	SESTD1	Cluster0003
15064466	ENSECAT00000008835 // SETD6 // SET domain containing 6 // --- // --- /// ENSECAT0000000	SETD6	Cluster0003
14934146	XM_001503226 // SGMS1 // sphingomyelin synthase 1 // --- // 100062484 /// ENSECAT000000	SGMS1	Cluster0003
14962359	XM_001918353 // SGSM2 // small G protein signaling modulator 2 // --- // 100072485 ///	SGSM2	Cluster0003
15110265	ENSECAT00000014843 // SIAE // sialic acid acetylesterase // --- // 100072250	SIAE	Cluster0003
15037044	ENSECAT00000002328 // SIRPB2 // signal-regulatory protein beta 2 // --- // ---	SIRPB2	Cluster0003
15035389	ENSECAT00000025806 // SKIV2L2 // superkiller viralicidic activity 2-like 2 (S. cerevisi	SKIV2L2	Cluster0003
14973012	XM_001916178 // SLC15A3 // solute carrier family 15, member 3 // --- // 100062045 /// E	SLC15A3	Cluster0003
15099120	ENSECAT00000019696 // SLC16A14 // solute carrier family 16, member 14 (monocarboxylic a	SLC16A14	Cluster0003
14986121	ENSECAT00000023412 // SLC22A5 // solute carrier family 22 (organic cation/carnitine tra	SLC22A5	Cluster0003
15036814	ENSECAT00000015857 // SLC23A2 // solute carrier family 23 (nucleobase transporters), me	SLC23A2	Cluster0003
15081547	XM_001494425 // SLC25A13 // solute carrier family 25 (aspartate/glutamate carrier), mem	SLC25A13	Cluster0003
15101311	XM_001915404 // SLC2A13 // solute carrier family 2 (facilitated glucose transporter), m	SLC2A13	Cluster0003
15019160	ENSECAT00000001557 // SLC35E2 // solute carrier family 35, member E2 // --- // ---	SLC35E2	Cluster0003
15004629	ENSECAT00000011800 // SLC46A3 // solute carrier family 46, member 3 // --- // 100062949	SLC46A3	Cluster0003
15072570	XM_001488793 // SLC6A4 // solute carrier family 6 (neurotransmitter transporter, seroto	SLC6A4	Cluster0003
14989940	DQ178640 // SLC8A1 // solute carrier family 8 (sodium/calcium exchanger), member 1 // -	SLC8A1	Cluster0003
15130065	ENSECAT00000020591 // SLC9A6 // solute carrier family 9, subfamily A (NHE6, cation prot	SLC9A6	Cluster0003
15111842	NM_001081789 // SLCO2B1 // solute carrier organic anion transporter family, member 2B1	SLCO2B1	Cluster0003

15041841	ENSECAT00000026435 // SMARCA2 // SWI/SNF related, matrix associated, actin dependent re	SMARCA2	Cluster0003
15105089	ENSECAT00000021013 // SORL1 // sortilin-related receptor, L(DLR class) A repeats contai	SORL1	Cluster0003
15087730	XM_001917352 // SORT1 // sortilin 1 // --- // 100061385 /// ENSECAT00000016625 // SORT1	SORT1	Cluster0003
15102236	ENSECAT00000017267 // SPRYD3 // SPRY domain containing 3 // --- // 100056536 /// XM_001	SPRYD3	Cluster0003
15039319	XM_001492503 // SPTLC3 // serine palmitoyltransferase, long chain base subunit 3 // ---	SPTLC3	Cluster0003
14946851	XM_001500135 // SQRDL // sulfide quinone reductase-like (yeast) // --- // 100070480 ///	SQRDL	Cluster0003
14989805	ENSECAT00000014562 // SRBD1 // S1 RNA binding domain 1 // --- // 100068692 /// XM_00149	SRBD1	Cluster0003
15031300	XM_001499381 // SRPK1 // SRSF protein kinase 1 // --- // 100063400 /// ENSECAT000000152	SRPK1	Cluster0003
15080357	ENSECAT00000011176 // SRPK2 // SRSF protein kinase 2 // --- // 100050379 /// XM_0014896	SRPK2	Cluster0003
14966449	NM_001195548 // SRSF1 // serine/arginine-rich splicing factor 1 // --- // 100056763 ///	SRSF1	Cluster0003
14967542	XM_001502056 // SSH2 // slingshot homolog 2 (Drosophila) // --- // 100072180 /// ENSECA	SSH2	Cluster0003
15124747	XM_001498838 // ST3GAL1 // ST3 beta-galactoside alpha-2,3-sialyltransferase 1 // --- //	ST3GAL1	Cluster0003
15100883	XM_001502320 // ST8SIA1 // ST8 alpha-N-acetyl-neuraminide alpha-2,8-sialyltransferase 1	ST8SIA1	Cluster0003
15001844	ENSECAT00000025069 // STAG1 // stromal antigen 1 // --- // 100066027 /// XM_001496443 /	STAG1	Cluster0003
15107241	XM_001499852 // STIM1 // stromal interaction molecule 1 // --- // 100053079 /// ENSECAT	STIM1	Cluster0003
15094301	ENSECAT00000018591 // STK11IP // serine/threonine kinase 11 interacting protein // ---	STK11IP	Cluster0003
15123923	XM_001492746 // STK3 // serine/threonine kinase 3 // --- // 100060508 /// ENSECAT000000	STK3	Cluster0003
14961216	XM_001503277 // STXBP4 // syntaxin binding protein 4 // --- // 100056587 /// ENSECAT000	STXBP4	Cluster0003
14995422	XM_001495004 // SUCLG2 // succinate-CoA ligase, GDP-forming, beta subunit // --- // 100	SUCLG2	Cluster0003
14942012	XM_001498704 // SUFU // suppressor of fused homolog (Drosophila) // --- // 100059929 //	SUFU	Cluster0003
15034854	XM_001503418 // SUGP2 // SURP and G patch domain containing 2 // --- // 100071102 /// E	SUGP2	Cluster0003
14995199	ENSECAT00000021716 // SUMF1 // sulfatase modifying factor 1 // --- // 100052857 /// XM_	SUMF1	Cluster0003
14978265	ENSECAT00000018761 // SUN1 // Sad1 and UNC84 domain containing 1 // --- // --- /// ENSE	SUN1	Cluster0003
14947941	XM_001505160 // SUPT16H // suppressor of Ty 16 homolog (S. cerevisiae) // --- // 100059	SUPT16H	Cluster0003
15062973	XM_001494423 // SVIL // supervillin // --- // 100063045 /// ENSECAT00000022469 // SVIL	SVIL	Cluster0003
14954663	ENSECAT00000008705 // SYMPK // symplekin // --- // --- /// ENSECAT00000008770 // SYMPK	SYMPK	Cluster0003
15055130	ENSECAT00000006588 // SYNJ1 // synaptojanin 1 // --- // 100052605 /// ENSECAT0000000659	SYNJ1	Cluster0003
15056961	ENSECAT00000015644 // TACC1 // transforming, acidic coiled-coil containing protein 1 //	TACC1	Cluster0003
14961661	XM_001503840 // TADA2A // transcriptional adaptor 2A // --- // 100057641 /// ENSECAT000	TADA2A	Cluster0003
15075701	ENSECAT00000022234 // TAGAP // T-cell activation RhoGTPase activating protein // --- //	TAGAP	Cluster0003
15006903	ENSECAT00000020014 // TANC1 // tetratricopeptide repeat, ankyrin repeat and coiled-coil	TANC1	Cluster0003
15050974	ENSECAT00000013996 // TANK // TRAF family member-associated NFKB activator // --- // 10	TANK	Cluster0003
14935304	XM_001916573 // TARBP1 // TAR (HIV-1) RNA binding protein 1 // --- // 100059375 /// ENS	TARBP1	Cluster0003
15071937	ENSECAT00000024010 // TBC1D1 // TBC1 (tre-2/USP6, BUB2, cdc16) domain family, member 1	TBC1D1	Cluster0003
15068209	ENSECAT00000005488 // TBC1D14 // TBC1 domain family, member 14 // --- // 100070312 ///	TBC1D14	Cluster0003
14945627	ENSECAT00000026577 // TBC1D2B // TBC1 domain family, member 2B // --- // 100060015 ///	TBC1D2B	Cluster0003
14981341	ENSECAT00000018936 // TBC1D9B // TBC1 domain family, member 9B (with GRAM domain) // --	TBC1D9B	Cluster0003
15014304	XM_001499222 // TBCCD1 // TBCC domain containing 1 // --- // 100059422 /// ENSECAT00000	TBCCD1	Cluster0003
15013963	ENSECAT00000008912 // TBL1XR1 // transducin (beta)-like 1 X-linked receptor 1 // --- //	TBL1XR1	Cluster0003
15046300	ENSECAT00000022829 // TECPR2 // tectonin beta-propeller repeat containing 2 // --- // 1	TECPR2	Cluster0003
14947820	XM_001505130 // TEP1 // telomerase-associated protein 1 // --- // 100072576 /// ENSECAT	TEP1	Cluster0003
15012507	NM_001081913 // TFRC // transferrin receptor (p90, CD71) // --- // 100034089 /// ENSECA	TFRC	Cluster0003
15001420	ENSECAT00000016147 // TGFBR2 // transforming growth factor, beta receptor II (70/80kDa)	TGFBR2	Cluster0003
15055033	XM_001498833 // TIAM1 // T-cell lymphoma invasion and metastasis 1 // --- // 100052764	TIAM1	Cluster0003
14937228	ENSECAT00000007482 // TLE3 // transducin-like enhancer of split 3 (E(sp1) homolog, Dros	TLE3	Cluster0003
15009919	XM_001498377 // TLK1 // tousled-like kinase 1 // --- // 100052963 /// ENSECAT0000002473	TLK1	Cluster0003
15051296	ENSECAT00000019115 // TLN1 // talin 1 // --- // --- /// ENSECAT00000019221 // TLN1 // t	TLN1	Cluster0003
15067746	NM_001257142 // TLR6 // toll-like receptor 6 // --- // 100064554 /// ENSECAT00000001932	TLR6	Cluster0003
15101040	XM_001502786 // TM7SF3 // transmembrane 7 superfamily member 3 // --- // 100069046 ///	TM7SF3	Cluster0003
15064919	ENSECAT00000016960 // TMCO7 // transmembrane and coiled-coil domains 7 // --- // 100066	TMCO7	Cluster0003
15096682	XM_001488535 // TMEM117 // transmembrane protein 117 // --- // 100050185 /// ENSECAT000	TMEM117	Cluster0003
14988115	ENSECAT00000008072 // TMEM131 // transmembrane protein 131 // --- // 100061749 /// XM_0	TMEM131	Cluster0003
15111557	ENSECAT00000007695 // TMEM135 // transmembrane protein 135 // --- // 100060782	TMEM135	Cluster0003
15028907	ENSECAT00000014451 // TMEM63B // transmembrane protein 63B // --- // 100067695	TMEM63B	Cluster0003

14988402	ENSECAT00000015896 // TMEM87B // transmembrane protein 87B // --- // 100064755 /// XM_0	TMEM87B	Cluster0003
15058310	ENSECAT00000025320 // TMPO // thymopoietin // --- // ---	TMPO	Cluster0003
15101091	ENSECAT00000008820 // TMTC1 // transmembrane and tetratricopeptide repeat containing 1	TMTC1	Cluster0003
15019073	ENSECAT00000008962 // TNFRSF14 // tumor necrosis factor receptor superfamily, member 14	TNFRSF14	Cluster0003
15013883	XM_001492782 // TNIK // TRAF2 and NCK interacting kinase // --- // 100057722 /// XM_003	TNIK	Cluster0003
15082744	ENSECAT00000013691 // TNPO3 // transportin 3 // --- // ---	TNPO3	Cluster0003
14978943	XM_001493663 // TPST1 // tyrosylprotein sulfotransferase 1 // --- // 100061873 /// ENSE	TPST1	Cluster0003
15059737	XM_001914876 // TRABD // TraB domain containing // --- // 100147257 /// ENSECAT00000023	TRABD	Cluster0003
15000886	ENSECAT00000015724 // TRAK1 // trafficking protein, kinesin binding 1 // --- // 1000676	TRAK1	Cluster0003
15016697	XM_001498287 // TRIT1 // tRNA isopentenyltransferase 1 // --- // 100068446 /// ENSECAT0	TRIT1	Cluster0003
14953099	XM_001503142 // TRMT11 // tRNA methyltransferase 11 homolog (S. cerevisiae) // --- // 1	TRMT11	Cluster0003
15054457	ENSECAT00000025586 // TRPM2 // transient receptor potential cation channel, subfamily M	TRPM2	Cluster0003
14977875	ENSECAT00000011311 // TRRAP // transformation/transcription domain-associated protein /	TRRAP	Cluster0003
14980725	ENSECAT00000023586 // TSC2 // tuberous sclerosis 2 // --- // 100065679	TSC2	Cluster0003
14998956	XM_001492367 // TSEN2 // tRNA splicing endonuclease 2 homolog (S. cerevisiae) // --- //	TSEN2	Cluster0003
14938833	ENSECAT00000025875 // TTBK2 // tau tubulin kinase 2 // --- // 100056539 /// XM_00150321	TTBK2	Cluster0003
14969531	XM_001489510 // TTC17 // tetratricopeptide repeat domain 17 // --- // 100055237 /// ENS	TTC17	Cluster0003
14983727	XM_001918332 // TTC37 // tetratricopeptide repeat domain 37 // --- // 100065001 /// ENS	TTC37	Cluster0003
14945546	XM_001917950 // TUBGCP5 // tubulin, gamma complex associated protein 5 // --- // 100147	TUBGCP5	Cluster0003
15051007	XM_001497726 // UAP1L1 // UDP-N-acteylglucosamine pyrophosphorylase 1-like 1 // --- //	UAP1L1	Cluster0003
14958453	ENSECAT00000007493 // UBE2O // ubiquitin-conjugating enzyme E2O // --- // ---	UBE2O	Cluster0003
15045695	ENSECAT00000005327 // UBR7 // ubiquitin protein ligase E3 component n-recognin 7 (putat	UBR7	Cluster0003
15113041	XM_001504967 // UEVLD // UEV and lactate/malate dehyrogenase domains // --- // 10005714	UEVLD	Cluster0003
15008523	XM_001504931 // UGGT1 // UDP-glucose glycoprotein glucosyltransferase 1 // --- // 10006	UGGT1	Cluster0003
15048396	ENSECAT00000021730 // UNC13B // unc-13 homolog B (C. elegans) // --- // 100055666 /// E	UNC13B	Cluster0003
14943775	XM_001496353 // URB2 // URB2 ribosome biogenesis 2 homolog (S. cerevisiae) // --- // 10	URB2	Cluster0003
14961566	XM_001503781 // USP32 // ubiquitin specific peptidase 32 // --- // 100071369 /// ENSECA	USP32	Cluster0003
14989403	ENSECAT00000024397 // USP34 // ubiquitin specific peptidase 34 // --- // 100064939 ///	USP34	Cluster0003
15099226	XM_001495779 // USP40 // ubiquitin specific peptidase 40 // --- // 100065074 /// ENSECA	USP40	Cluster0003
15058444	ENSECAT00000007600 // UTP20 // UTP20, small subunit (SSU) processome component, homolog	UTP20	Cluster0003
15101585	NM_001163959 // VDR // vitamin D (1,25- dihydroxyvitamin D3) receptor // --- // 1000340	VDR	Cluster0003
15121915	ENSECAT00000021375 // VPS13B // vacuolar protein sorting 13 homolog B (yeast) // --- //	VPS13B	Cluster0003
15023955	XM_001490932 // VPS13D // vacuolar protein sorting 13 homolog D (S. cerevisiae) // ---	VPS13D	Cluster0003
14947346	XM_001503497 // VPS18 // vacuolar protein sorting 18 homolog (S. cerevisiae) // --- //	VPS18	Cluster0003
14944812	ENSECAT00000027032 // VPS33B // vacuolar protein sorting 33 homolog B (yeast) // --- //	VPS33B	Cluster0003
15080649	ENSECAT00000007739 // VPS41 // vacuolar protein sorting 41 homolog (S. cerevisiae) // -	VPS41	Cluster0003
14962240	ENSECAT00000007354 // VPS53 // vacuolar protein sorting 53 homolog (S. cerevisiae) // -	VPS53	Cluster0003
15012113	XM_001498641 // VPS8 // vacuolar protein sorting 8 homolog (S. cerevisiae) // --- // 10	VPS8	Cluster0003
14941297	ENSECAT00000021574 // WDR11 // WD repeat domain 11 // --- // 100065386	WDR11	Cluster0003
15091919	XM_001500852 // WDR3 // WD repeat domain 3 // --- // 100059816 /// ENSECAT00000007455 /	WDR3	Cluster0003
15116587	ENSECAT00000007500 // WDR7 // WD repeat domain 7 // --- // 100049995 /// ENSECAT0000001	WDR7	Cluster0003
15023079	ENSECAT00000026317 // WDTC1 // WD and tetratricopeptide repeats 1 // --- // 100056964 /	WDTC1	Cluster0003
15099694	ENSECAT00000025398 // WNK1 // WNK lysine deficient protein kinase 1 // --- // --- /// E	WNK1	Cluster0003
15122997	XM_001488288 // WWP1 // WW domain containing E3 ubiquitin protein ligase 1 // --- // 10	WWP1	Cluster0003
15065008	JN712754 // WWP2 // WW domain containing E3 ubiquitin protein ligase 2 // --- // 100067	WWP2	Cluster0003
15004386	ENSECAT00000020649 // XPO4 // exportin 4 // --- // 100054789 /// XM_001488990 // XPO4 /	XPO4	Cluster0003
15031721	XM_001918236 // XPO5 // exportin 5 // --- // 100067497 /// ENSECAT00000003174 // XPO5 /	XPO5	Cluster0003
15019360	XM_001490507 // XPO7 // exportin 7 // --- // 100056362 /// ENSECAT00000025425 // XPO7 /	XPO7	Cluster0003
15017212	XM_001499806 // YARS // tyrosyl-tRNA synthetase // --- // 100070128 /// ENSECAT00000010	YARS	Cluster0003
15101203	ENSECAT00000011549 // YARS2 // tyrosyl-tRNA synthetase 2, mitochondrial // --- // 10006	YARS2	Cluster0003
14986556	XM_001918301 // YTHDC2 // YTH domain containing 2 // --- // 100064372 /// ENSECAT000000	YTHDC2	Cluster0003
15084437	ENSECAT00000014156 // ZBTB37 // zinc finger and BTB domain containing 37 // --- // 1000	ZBTB37	Cluster0003
15065763	ENSECAT00000012512 // ZC3H18 // zinc finger CCCH-type containing 18 // --- // --- /// E	ZC3H18	Cluster0003
15015924	ENSECAT00000023091 // ZCCHC11 // zinc finger, CCHC domain containing 11 // --- // 10005	ZCCHC11	Cluster0003

15124756	ENSECAT00000026725 // ZFAT // zinc finger and AT hook domain containing // --- // 10006	ZFAT	Cluster0003
15047065	ENSECAT00000019613 // ZFYVE26 // zinc finger, FYVE domain containing 26 // --- // 10006	ZFYVE26	Cluster0003
15002478	ENSECAT00000004370 // ZMYM2 // zinc finger, MYM-type 2 // --- // --- /// ENSECAT0000000	ZMYM2	Cluster0003
15022603	ENSECAT00000009693 // ZMYM4 // zinc finger, MYM-type 4 // --- // 100069550	ZMYM4	Cluster0003
15116990	ENSECAT00000006376 // ZNF236 // zinc finger protein 236 // --- // 100063239 /// XM_0014	ZNF236	Cluster0003
15130042	ENSECAT00000003600 // ZNF449 // zinc finger protein 449 // --- // 100057349 /// XM_0014	ZNF449	Cluster0003
15029272	ENSECAT00000010065 // ZNF451 // zinc finger protein 451 // --- // 100070011 /// XM_0014	ZNF451	Cluster0003
14942429	NM_001257122 // ZNF518A // zinc finger protein 518A // --- // 100061148	ZNF518A	Cluster0003
14992602	ENSECAT00000028898 // ZNF638 // zinc finger protein 638 // --- // --- /// ENSECAT000000	ZNF638	Cluster0003
15025716	XM_001501949 // ABCE1 // ATP-binding cassette, sub-family E (OABP), member 1 // --- //	ABCE1	Cluster0004
15096954	ENSECAT00000023621 // ACCN2 // acid-sensing (proton-gated) ion channel 1 // --- // 1000	ACCN2	Cluster0004
14948119	XM_001494104 // ACIN1 // apoptotic chromatin condensation inducer 1 // --- // 100055865	ACIN1	Cluster0004
14960190	XM_001495646 // ACLY // ATP citrate lyase // --- // 100053195 /// ENSECAT00000010787 //	ACLY	Cluster0004
15042343	XM_001497806 // ACO1 // aconitase 1, soluble // --- // 100067866 /// ENSECAT00000016500	ACO1	Cluster0004
15006670	NM_001242549 // ACVR2A // activin A receptor, type IIA // --- // 100049823	ACVR2A	Cluster0004
15097118	ENSECAT00000009473 // ACVRL1 // activin A receptor type II-like 1 // --- // 100061313 /	ACVRL1	Cluster0004
14941778	XM_001497076 // ADD3 // adducin 3 (gamma) // --- // 100059447 /// ENSECAT00000015130 //	ADD3	Cluster0004
14937077	ENSECAT00000021871 // ADPGK // ADP-dependent glucokinase // --- // 100052166 /// XM_001	ADPGK	Cluster0004
15079234	XM_001498120 // AGK // acylglycerol kinase // --- // 100064603 /// ENSECAT00000015203 /	AGK	Cluster0004
15034494	ENSECAT00000016071 // AKAP8 // A kinase (PRKA) anchor protein 8 // --- // 100063189	AKAP8	Cluster0004
15072910	XM_001491965 // AKT3 // v-akt murine thymoma viral oncogene homolog 3 (protein kinase B	AKT3	Cluster0004
15096516	ENSECAT00000014031 // ALG10 // asparagine-linked glycosylation 10, alpha-1,2-glucosyltr	ALG10	Cluster0004
15072090	XM_001497457 // ANAPC4 // anaphase promoting complex subunit 4 // --- // 100067388 ///	ANAPC4	Cluster0004
15088722	XM_001499345 // ANKRD13C // ankyrin repeat domain 13C // --- // 100053662 /// ENSECAT00	ANKRD13C	Cluster0004
15102655	XM_001492032 // ANKRD52 // ankyrin repeat domain 52 // --- // 100059440 /// ENSECAT0000	ANKRD52	Cluster0004
14997153	ENSECAT00000000574 // ANO10 // anoctamin 10 // --- // 100054874 /// XM_001501370 // ANO	ANO10	Cluster0004
14951237	ENSECAT00000007814 // AP2A1 // adaptor-related protein complex 2, alpha 1 subunit // --	AP2A1	Cluster0004
15011940	XM_001497146 // AP2M1 // adaptor-related protein complex 2, mu 1 subunit // --- // 1000	AP2M1	Cluster0004
15025584	XM_003364540 // ARFIP1 // ADP-ribosylation factor interacting protein 1 // --- // 10062	ARFIP1	Cluster0004
15057715	ENSECAT00000017030 // ARHGEF10 // Rho guanine nucleotide exchange factor (GEF) 10 // --	ARHGEF10	Cluster0004
15044413	ENSECAT00000020848 // ARID4A // AT rich interactive domain 4A (RBP1-like) // --- // 100	ARID4A	Cluster0004
14977869	XM_001494582 // ARPC1A // actin related protein 2/3 complex, subunit 1A, 41kDa // --- /	ARPC1A	Cluster0004
14968333	XM_001502964 // ARRB2 // arrestin, beta 2 // --- // 100072892 /// ENSECAT00000020354 //	ARRB2	Cluster0004
15124610	ENSECAT00000017553 // ASAP1 // ArfGAP with SH3 domain, ankyrin repeat and PH domain 1 /	ASAP1	Cluster0004
15113773	ENSECAT00000025242 // ASCC2 // activating signal cointegrator 1 complex subunit 2 // --	ASCC2	Cluster0004
15050324	XM_001499340 // ASS1 // argininosuccinate synthase 1 // --- // 100069776 /// ENSECAT000	ASS1	Cluster0004
15111110	XM_001491040 // ATG4D // autophagy related 4D, cysteine peptidase // --- // 100057841 /	ATG4D	Cluster0004
15034955	XM_001500904 // ATP13A1 // ATPase type 13A1 // --- // 100071205 /// ENSECAT00000022204	ATP13A1	Cluster0004
15130509	XM_003365908 // ATP6AP1 // ATPase, H+ transporting, lysosomal accessory protein 1 // --	ATP6AP1	Cluster0004
15078229	ENSECAT00000020747 // AVL9 // AVL9 homolog (S. cerevisiase) // --- // 100070105 /// XM_	AVL9	Cluster0004
15002764	XM_001495151 // B3GALTL // beta 1,3-galactosyltransferase-like // --- // 100064175 ///	B3GALTL	Cluster0004
15020858	XM_001503097 // BBS7 // Bardet-Biedl syndrome 7 // --- // 100063923 /// ENSECAT00000025	BBS7	Cluster0004
14937788	ENSECAT00000024286 // BNIP2 // BCL2/adenovirus E1B 19kDa interacting protein 2 // --- /	BNIP2	Cluster0004
15130603	ENSECAT00000023027 // BRCC3 // BRCA1/BRCA2-containing complex, subunit 3 // --- // 1000	BRCC3	Cluster0004
15035852	XM_001500161 // BRIX1 // BRX1, biogenesis of ribosomes, homolog (S. cerevisiae) // ---	BRIX1	Cluster0004
15047965	ENSECAT00000013587 // BTBD7 // BTB (POZ) domain containing 7 // --- // 100053454	BTBD7	Cluster0004
15132639	XM_001493218 // BTK // Bruton agammaglobulinemia tyrosine kinase // --- // 100057626 //	BTK	Cluster0004
14943564	XM_001503918 // CAMK2G // calcium/calmodulin-dependent protein kinase II gamma // --- /	CAMK2G	Cluster0004
15033015	ENSECAT00000011466 // CCDC125 // coiled-coil domain containing 125 // --- // 100055798	CCDC125	Cluster0004
15096438	ENSECAT00000015713 // CCDC91 // coiled-coil domain containing 91 // --- // 100064764 //	CCDC91	Cluster0004
15098313	XM_003365200 // CCT2 // chaperonin containing TCP1, subunit 2 (beta) // --- // 10005991	CCT2	Cluster0004
14992523	XM_003362972 // CCT7 // chaperonin containing TCP1, subunit 7 (eta) // --- // 100050014	CCT7	Cluster0004
15048250	XM_001491699 // CDC42BPB // CDC42 binding protein kinase beta (DMPK-like) // --- // 100	CDC42BPB	Cluster0004
15035018	XM_001491054 // CDK7 // cyclin-dependent kinase 7 // --- // 100049964 /// ENSECAT000000	CDK7	Cluster0004

14972465	XM_003362602 // CELF1 // CUGBP, Elav-like family member 1 // --- // 100051608 /// ENSEC	CELF1	Cluster0004
14936281	XM_001488013 // CHD2 // chromodomain helicase DNA binding protein 2 // --- // 100049803	CHD2	Cluster0004
15119156	XM_001493348 // CHFR // checkpoint with forkhead and ring finger domains, E3 ubiquitin	CHFR	Cluster0004
15001371	XM_001491223 // CNOT10 // CCR4-NOT transcription complex, subunit 10 // --- // 10005081	CNOT10	Cluster0004
15065293	XM_001500840 // COG4 // component of oligomeric golgi complex 4 // --- // 100054801 ///	COG4	Cluster0004
15003062	ENSECAT00000011756 // COG6 // component of oligomeric golgi complex 6 // --- // 1000508	COG6	Cluster0004
14974019	XM_001917529 // CORO1B // coronin, actin binding protein, 1B // --- // 100053095 /// EN	CORO1B	Cluster0004
14974947	ENSECAT00000016488 // COX19 // COX19 cytochrome c oxidase assembly homolog (S. cerevisi	COX19	Cluster0004
14984636	XM_001502804 // CPEB4 // cytoplasmic polyadenylation element binding protein 4 // --- /	CPEB4	Cluster0004
15008423	JQ044378 // CREB1 // cAMP responsive element binding protein 1 // --- // 100066388	CREB1	Cluster0004
15068447	ENSECAT00000016987 // CTBP1 // C-terminal binding protein 1 // --- // 100052216 /// XM_	CTBP1	Cluster0004
15004218	ENSECAT00000020697 // CUL4A // cullin 4A // --- // 100033930 /// ENSECAT00000020701 //	CUL4A	Cluster0004
15133114	ENSECAT00000025736 // CUL4B // cullin 4B // --- // 100033938	CUL4B	Cluster0004
15010507	ENSECAT00000011599 // CWC22 // CWC22 spliceosome-associated protein homolog (S. cerevis	CWC22	Cluster0004
15042730	XM_003363984 // DAPK1 // death-associated protein kinase 1 // --- // 100061567 /// ENSE	DAPK1	Cluster0004
15077284	XM_001489324 // DBF4 // DBF4 homolog (S. cerevisiae) // --- // 100050313 /// ENSECAT000	DBF4	Cluster0004
15038161	ENSECAT00000000414 // DBNDD2 // dysbindin (dystrobrevin binding protein 1) domain conta	DBNDD2	Cluster0004
15043978	XM_001499560 // DCAF12 // DDB1 and CUL4 associated factor 12 // --- // 100068462 /// EN	DCAF12	Cluster0004
15069612	XM_001500964 // DDX19B // DEAD (Asp-Glu-Ala-Asp) box polypeptide 19B // --- // 10005493	DDX19B	Cluster0004
15101716	XM_001504119 // DDX23 // DEAD (Asp-Glu-Ala-Asp) box polypeptide 23 // --- // 100051439	DDX23	Cluster0004
15038404	XM_001501119 // DDX27 // DEAD (Asp-Glu-Ala-Asp) box polypeptide 27 // --- // 100071375	DDX27	Cluster0004
15053039	XM_001499227 // DDX31 // DEAD (Asp-Glu-Ala-Asp) box polypeptide 31 // --- // 100069463	DDX31	Cluster0004
15074578	ENSECAT00000021935 // DENND1B // DENN/MADD domain containing 1B // --- // 100061134	DENND1B	Cluster0004
15042177	ENSECAT00000000697 // DENND4C // DENN/MADD domain containing 4C // --- // 100052312	DENND4C	Cluster0004
15023161	ENSECAT00000017264 // DHDDS // dehydrodolichyl diphosphate synthase // --- // 100057242	DHDDS	Cluster0004
14965485	ENSECAT00000021248 // DHX8 // DEAH (Asp-Glu-Ala-His) box polypeptide 8 // --- // 100051	DHX8	Cluster0004
15130576	ENSECAT00000025423 // DKC1 // dyskeratosis congenita 1, dyskerin // --- // 100062345 //	DKC1	Cluster0004
15104450	ENSECAT00000023060 // DLAT // dihydrolipoamide S-acetyltransferase // --- // 100062027	DLAT	Cluster0004
15007619	ENSECAT00000010535 // DNAJC10 // DnaJ (Hsp40) homolog, subfamily C, member 10 // --- //	DNAJC10	Cluster0004
15121516	NM_001163858 // DNAPK // DNA-dependent protein kinase catalytic subunit // --- // 79123	DNAPK	Cluster0004
15105837	ENSECAT00000020211 // DNASE2 // deoxyribonuclease II, lysosomal // --- // ---	DNASE2	Cluster0004
15088902	ENSECAT00000009395 // DOCK7 // dedicator of cytokinesis 7 // --- // 100070382 /// XM_00	DOCK7	Cluster0004
15111593	ENSECAT00000017377 // EED // embryonic ectoderm development // --- // ---	EED	Cluster0004
15004441	XM_001489625 // EFHA1 // EF-hand domain family, member A1 // --- // 100055452 /// ENSEC	EFHA1	Cluster0004
14959697	XM_003362540 // EFTUD2 // elongation factor Tu GTP binding domain containing 2 // --- /	EFTUD2	Cluster0004
15012029	NM_001163871 // EIF4G1 // eukaryotic translation initiation factor 4 gamma, 1 // --- //	EIF4G1	Cluster0004
14991615	ENSECAT00000024261 // EIF5B // eukaryotic translation initiation factor 5B // --- // 10	EIF5B	Cluster0004
15020704	ENSECAT00000023598 // ELF2 // E74-like factor 2 (ets domain transcription factor) // --	ELF2	Cluster0004
15116254	XM_001916080 // ELP2 // elongation protein 2 homolog (S. cerevisiae) // --- // 10005293	ELP2	Cluster0004
15024860	XM_001492944 // ENTPD4 // ectonucleoside triphosphate diphosphohydrolase 4 // --- // 10	ENTPD4	Cluster0004
15006699	ENSECAT00000011273 // EPC2 // enhancer of polycomb homolog 2 (Drosophila) // --- // 100	EPC2	Cluster0004
14959323	XM_001495274 // ERN1 // endoplasmic reticulum to nucleus signaling 1 // --- // 10006433	ERN1	Cluster0004
15046637	ENSECAT00000014461 // ERO1L // ERO1-like (S. cerevisiae) // --- // ---	ERO1L	Cluster0004
15115769	XM_001491258 // ESCO1 // establishment of cohesion 1 homolog 1 (S. cerevisiae) // --- /	ESCO1	Cluster0004
14942619	XM_001502528 // EXOC6 // exocyst complex component 6 // --- // 100061840 /// ENSECAT000	EXOC6	Cluster0004
14989084	ENSECAT00000020799 // EXOC6B // exocyst complex component 6B // --- // 100059535 /// XM	EXOC6B	Cluster0004
14959999	ENSECAT00000009661 // EZH1 // enhancer of zeste homolog 1 (Drosophila) // --- // 100052	EZH1	Cluster0004
14983519	XM_001918313 // FBXL17 // F-box and leucine-rich repeat protein 17 // --- // 100146140	FBXL17	Cluster0004
15068036	ENSECAT00000026208 // FBXL5 // F-box and leucine-rich repeat protein 5 // --- // 100055	FBXL5	Cluster0004
15018401	XM_001488662 // FBXO42 // F-box protein 42 // --- // 100053402 /// ENSECAT00000019734 /	FBXO42	Cluster0004
15017509	XM_001504019 // FGR // Gardner-Rasheed feline sarcoma viral (v-fgr) oncogene homolog //	FGR	Cluster0004
14967415	ENSECAT00000012832 // FLOT2 // flotillin 2 // --- // 100072090 /// XM_001501952 // FLOT	FLOT2	Cluster0004
14965457	ENSECAT00000026227 // G6PC3 // glucose 6 phosphatase, catalytic, 3 // --- // 100064862	G6PC3	Cluster0004
15133967	XM_001492232 // G6PD // glucose-6-phosphate dehydrogenase // --- // 100059734 /// ENSEC	G6PD	Cluster0004

15007881	ENSECAT00000011641 // GLS // glutaminase // --- // 100069617 /// XM_001499354 // GLS //	GLS	Cluster0004
15032705	XM_001499899 // GLT25D1 // glycosyltransferase 25 domain containing 1 // --- // 1000702	GLT25D1	Cluster0004
14977069	XM_001499634 // GLYR1 // glyoxylate reductase 1 homolog (Arabidopsis) // --- // 1000699	GLYR1	Cluster0004
15016871	XM_001503591 // GNL2 // guanine nucleotide binding protein-like 2 (nucleolar) // --- //	GNL2	Cluster0004
15060512	ENSECAT00000011449 // GNPTAB // N-acetylglucosamine-1-phosphate transferase, alpha and	GNPTAB	Cluster0004
15012858	ENSECAT00000009916 // GOLGB1 // golgin B1 // --- // 100070765 /// XM_001916854 // GOLGB	GOLGB1	Cluster0004
14933535	XM_001501044 // GOT1 // glutamic-oxaloacetic transaminase 1, soluble (aspartate aminotr	GOT1	Cluster0004
14944084	XM_001916496 // GPR137B // G protein-coupled receptor 137B // --- // 100050643 /// ENSE	GPR137B	Cluster0004
14976850	XM_001489900 // GSPT1 // G1 to S phase transition 1 // --- // 100055960 /// XM_00336273	GSPT1	Cluster0004
14987651	XM_001504043 // GTF2H2 // general transcription factor IIH, polypeptide 2, 44kDa // ---	GTF2H2	Cluster0004
15059103	ENSECAT00000019388 // GTPBP1 // GTP binding protein 1 // --- // 100147081 /// XM_001916	GTPBP1	Cluster0004
14956830	XM_001501736 // HACE1 // HECT domain and ankyrin repeat containing E3 ubiquitin protein	HACE1	Cluster0004
14990571	XM_001502783 // HADHA // hydroxyacyl-CoA dehydrogenase/3-ketoacyl-CoA thiolase/enoyl-Co	HADHA	Cluster0004
14966849	ENSECAT00000020639 // HEATR6 // HEAT repeat containing 6 // --- // 100071508 /// XM_001	HEATR6	Cluster0004
14972074	XM_003362582 // HIPK3 // homeodomain interacting protein kinase 3 // --- // 100059137 /	HIPK3	Cluster0004
14984691	NM_001081835 // HMGB1 // high mobility group box 1 // --- // 100033873 /// AB275457 //	HMGB1	Cluster0004
15129125	XM_001493778 // HNRNPH2 // heterogeneous nuclear ribonucleoprotein H2 (H) // --- // 10	HNRNPH2	Cluster0004
14953947	ENSECAT00000013350 // HNRNPL // heterogeneous nuclear ribonucleoprotein L // --- // 100	HNRNPL	Cluster0004
15093058	ENSECAT00000001370 // HS2ST1 // heparan sulfate 2-O-sulfotransferase 1 // --- // 100063	HS2ST1	Cluster0004
15004676	XM_001493517 // HSPH1 // heat shock 105kDa/110kDa protein 1 // --- // 100062150 /// ENS	HSPH1	Cluster0004
15056786	ENSECAT00000005219 // IKBKB // inhibitor of kappa light polypeptide gene enhancer in B-	IKBKB	Cluster0004
15033233	ENSECAT00000021352 // IL6ST // interleukin 6 signal transducer (gp130, oncostatin M rec	IL6ST	Cluster0004
14988650	ENSECAT00000026571 // IMMT // inner membrane protein, mitochondrial // --- // 100067027	IMMT	Cluster0004
15003160	ENSECAT00000014628 // INTS6 // integrator complex subunit 6 // --- // 100053346 /// XM_	INTS6	Cluster0004
15085258	XM_003364961 // INTS7 // integrator complex subunit 7 // --- // 100051001 /// XM_001489	INTS7	Cluster0004
15121776	ENSECAT00000022027 // INTS8 // integrator complex subunit 8 // --- // 100055266	INTS8	Cluster0004
15096670	XM_001488439 // IRAK4 // interleukin-1 receptor-associated kinase 4 // --- // 100054848	IRAK4	Cluster0004
14943098	ENSECAT00000013768 // JMJD1C // jumonji domain containing 1C // --- // ---	JMJD1C	Cluster0004
15023419	ENSECAT00000021147 // KDM1A // lysine (K)-specific demethylase 1A // --- // 100071646	KDM1A	Cluster0004
15022822	ENSECAT00000020673 // KHDRBS1 // KH domain containing, RNA binding, signal transduction	KHDRBS1	Cluster0004
14967348	ENSECAT00000019247 // KIAA0100 // KIAA0100 // --- // --- /// ENSECAT00000019280 // KIAA	KIAA0100	Cluster0004
15035258	XM_001493976 // KIF2A // kinesin heavy chain member 2A // --- // 100050982 /// ENSECAT0	KIF2A	Cluster0004
15062601	XM_001916725 // KIN // KIN, antigenic determinant of recA protein homolog (mouse) // --	KIN	Cluster0004
15046411	ENSECAT00000016217 // KLC1 // kinesin light chain 1 // --- // 100055290 /// XM_00149135	KLC1	Cluster0004
15003616	XM_001495367 // KLF5 // Kruppel-like factor 5 (intestinal) // --- // 100052058 /// ENSE	KLF5	Cluster0004
15078860	XM_001502956 // KLHDC10 // kelch domain containing 10 // --- // 100057051 /// ENSECAT00	KLHDC10	Cluster0004
15025290	ENSECAT00000011464 // KLHL2 // kelch-like 2, Mayven (Drosophila) // --- // 100068150 //	KLHL2	Cluster0004
15043751	NM_001257143 // KLHL9 // kelch-like 9 (Drosophila) // --- // 100064784 /// ENSECAT00000	KLHL9	Cluster0004
15081362	ENSECAT00000018262 // KRIT1 // KRIT1, ankyrin repeat containing // --- // 100050998 ///	KRIT1	Cluster0004
15000681	ENSECAT00000025995 // LIMD1 // LIM domains containing 1 // --- // ---	LIMD1	Cluster0004
15091054	XM_001499888 // LMNA // lamin A/C // --- // 100057663 /// ENSECAT00000011877 // LMNA //	LMNA	Cluster0004
15070075	XM_001488006 // LOC100049843 // cytochrome b-245 light chain-like // --- // 100049843 /	LOC100049843	Cluster0004
15063833	XM_001487932 // LOC100049912 // ras-related protein Rap-1A-like // --- // 100049912 ///	LOC100049912	Cluster0004
15049327	XM_001487977 // LOC100049971 // transmembrane protein C9orf91-like // --- // 100049971	LOC100049971	Cluster0004
15101338	XM_001914700 // LOC100049989 // zinc finger CCHC-type and RNA-binding motif-containing	LOC100049989	Cluster0004
14976601	ENSECAT00000010646 // LOC100050012 // glycerophosphodiester phosphodiesterase 1-like //	LOC100050012	Cluster0004
15045865	XM_001488249 // LOC100050033 // serine/threonine-protein kinase VRK1-like // --- // 100	LOC100050033	Cluster0004
15120406	XM_001488219 // LOC100050058 // asparaginyl-tRNA synthetase, cytoplasmic-like // --- //	LOC100050058	Cluster0004
15106580	XM_001488653 // LOC100050120 // cysteine and histidine-rich domain-containing protein 1	LOC100050120	Cluster0004
14994788	XM_003363052 // LOC100050146 // cellular nucleic acid-binding protein-like // --- // 10	LOC100050146	Cluster0004
15085619	XM_001487994 // LOC100050332 // dual specificity protein phosphatase 12-like // --- //	LOC100050332	Cluster0004
15125464	ENSECAT00000008830 // LOC100050718 // uncharacterized protein C22orf25 homolog // --- /	LOC100050718	Cluster0004
15089172	XM_001490878 // LOC100050772 // ras-related protein Rab-7b-like // --- // 100050772 ///	LOC100050772	Cluster0004
14944107	XM_001492832 // LOC100050954 // geranylgeranyl pyrophosphate synthase-like // --- // 10	LOC100050954	Cluster0004

15008961	XM_001490165 // LOC100050974 // c-X-C chemokine receptor type 4-like // --- // 10005097	LOC100050974	Cluster0004
14948784	ENSECAT00000012263 // LOC100051031 // trafficking protein particle complex subunit 6B-l	LOC100051031	Cluster0004
15127079	XM_001490349 // LOC100051095 // carbonic anhydrase 5B, mitochondrial-like // --- // 100	LOC100051095	Cluster0004
14970893	ENSECAT00000024544 // LOC100051106 // calpain-1 catalytic subunit-like // --- // 100051	LOC100051106	Cluster0004
15084804	XM_001488444 // LOC100051159 // xenotropic and polytropic retrovirus receptor 1-like //	LOC100051159	Cluster0004
15072698	XM_001489181 // LOC100051309 // e3 ubiquitin ligase RNF4-like // --- // 100051309 /// E	LOC100051309	Cluster0004
14988191	XM_001492041 // LOC100051330 // VIP36-like protein-like // --- // 100051330 /// ENSECAT	LOC100051330	Cluster0004
15068851	XM_001915110 // LOC100051355 // cleavage and polyadenylation specificity factor subunit	LOC100051355	Cluster0004
15121733	ENSECAT00000017156 // LOC100051376 // Pyruvate dehydrogenase [acetyl-transferring]-phos	LOC100051376	Cluster0004
14936929	ENSECAT00000000149 // LOC100051391 // cytochrome c oxidase subunit 5A, mitochondrial-li	LOC100051391	Cluster0004
15041910	XM_001917198 // LOC100051417 // hsp90 co-chaperone Cdc37-like 1-like // --- // 10005141	LOC100051417	Cluster0004
15095534	XM_001494601 // LOC100051504 // NADH dehydrogenase [ubiquinone] 1 alpha subcomplex subu	LOC100051504	Cluster0004
14958623	XM_001494673 // LOC100051538 // 39S ribosomal protein L38, mitochondrial-like // --- //	LOC100051538	Cluster0004
15003503	ENSECAT00000013728 // LOC100051549 // suppressor of G2 allele of SKP1 homolog // --- //	LOC100051549	Cluster0004
15018702	XM_001493871 // LOC100051553 // protein LZIC-like // --- // 100051553 /// ENSECAT000000	LOC100051553	Cluster0004
15076912	XM_001495620 // LOC100051642 // drebrin-like protein-like // --- // 100051642 /// XM_00	LOC100051642	Cluster0004
14945930	ENSECAT00000004496 // LOC100052046 // CD276 antigen-like // --- // 100052046 /// XM_001	LOC100052046	Cluster0004
15024339	XM_001495398 // LOC100052123 // protein DJ-1-like // --- // 100052123 /// ENSECAT000000	LOC100052123	Cluster0004
15064289	ENSECAT00000014565 // LOC100052133 // RING finger and SPRY domain-containing protein 1-	LOC100052133	Cluster0004
15089466	XM_001496519 // LOC100052201 // vesicle-associated membrane protein 4-like // --- // 10	LOC100052201	Cluster0004
15116149	XM_001495353 // LOC100052335 // e3 ubiquitin-protein ligase RNF125-like // --- // 10005	LOC100052335	Cluster0004
15102692	XM_001504833 // LOC100052340 // protein canopy homolog 2-like // --- // 100052340 /// E	LOC100052340	Cluster0004
14959996	XM_001493268 // LOC100052353 // coiled-coil domain-containing protein 56-like // --- //	LOC100052353	Cluster0004
15055179	XM_001497986 // LOC100052430 // transmembrane protein 50B-like // --- // 100052430 ///	LOC100052430	Cluster0004
15031016	XM_001496005 // LOC100052481 // proteasome subunit beta type-8-like // --- // 100052481	LOC100052481	Cluster0004
15068436	XM_001917814 // LOC100052572 // transmembrane protein 129-like // --- // 100052572 ///	LOC100052572	Cluster0004
15065932	ENSECAT00000020496 // LOC100052665 // transcription factor 25-like // --- // 100052665	LOC100052665	Cluster0004
15003673	ENSECAT00000010662 // LOC100052699 // ceroid-lipofuscinosis neuronal protein 5-like //	LOC100052699	Cluster0004
15066347	ENSECAT00000008370 // LOC100052823 // estradiol 17-beta-dehydrogenase 11-like // --- //	LOC100052823	Cluster0004
15027108	ENSECAT00000026293 // LOC100052917 // tripartite motif-containing protein 38-like // --	LOC100052917	Cluster0004
15121653	XM_001488342 // LOC100052944 // OTU domain-containing protein 6B-like // --- // 1000529	LOC100052944	Cluster0004
14951676	XM_001488024 // LOC100052952 // 40S ribosomal protein S9-like // --- // 100052952 /// E	LOC100052952	Cluster0004
15047051	XM_001499937 // LOC100052972 // vesicle transport through interaction with t-SNAREs hom	LOC100052972	Cluster0004
15060549	ENSECAT00000013639 // LOC100053117 // WASH complex subunit CCDC53-like // --- // 100053	LOC100053117	Cluster0004
15064594	XM_001915760 // LOC100053119 // transcription factor E2F4-like // --- // 100053119 ///	LOC100053119	Cluster0004
14946286	XM_001497817 // LOC100053192 // 3-hydroxyacyl-CoA dehydratase 3-like // --- // 10005319	LOC100053192	Cluster0004
15041503	XM_001488638 // LOC100053331 // uncharacterized LOC100053331 // --- // 100053331 /// EN	LOC100053331	Cluster0004
15045693	XM_001497576 // LOC100053356 // UPF0694 transmembrane protein C14orf109 homolog // ---	LOC100053356	Cluster0004
15093394	XM_001498949 // LOC100053370 // tRNA wybutosine-synthesizing protein 3 homolog // --- /	LOC100053370	Cluster0004
14995401	XM_003363051 // LOC100053493 // uncharacterized glycosyltransferase AER61-like // --- /	LOC100053493	Cluster0004
15097922	ENSECAT00000014187 // LOC100053518 // phosphatidylinositol-5-phosphate 4-kinase type-2	LOC100053518	Cluster0004
14936098	XM_001502860 // LOC100053632 // calcium and integrin-binding protein 1-like // --- // 1	LOC100053632	Cluster0004
15069353	XM_001498448 // LOC100053706 // solute carrier family 12 member 4-like // --- // 100053	LOC100053706	Cluster0004
15071375	ENSECAT00000014599 // LOC100053708 // DNA-directed RNA polymerase II subunit RPB2-like	LOC100053708	Cluster0004
14996695	XM_001498943 // LOC100053786 // cytochrome b-c1 complex subunit 1, mitochondrial-like /	LOC100053786	Cluster0004
15053797	XM_001499592 // LOC100053798 // ubiquitin carboxyl-terminal hydrolase 16-like // --- //	LOC100053798	Cluster0004
15070337	ENSECAT00000018313 // LOC100053857 // dual adapter for phosphotyrosine and 3-phosphotyr	LOC100053857	Cluster0004
15060740	XM_001499461 // LOC100054153 // tRNA-splicing ligase RtcB homolog // --- // 100054153 /	LOC100054153	Cluster0004
15132894	XM_001489664 // LOC100054363 // AMME syndrome candidate gene 1 protein homolog // --- /	LOC100054363	Cluster0004
15039933	ENSECAT00000022181 // LOC100054381 // adenosylhomocysteinase-like // --- // 100054381 /	LOC100054381	Cluster0004
15129450	XM_001489834 // LOC100054407 // transmembrane protein 164-like // --- // 100054407 ///	LOC100054407	Cluster0004
15004969	ENSECAT00000015107 // LOC100054469 // ribonuclease H2 subunit B-like // --- // 10005446	LOC100054469	Cluster0004
14993723	ENSECAT00000010851 // LOC100054514 // spastin-like // --- // 100054514	LOC100054514	Cluster0004
15060852	XM_001500445 // LOC100054528 // eukaryotic translation initiation factor 3 subunit D-li	LOC100054528	Cluster0004

15038508	XM_001914822 // LOC100054563 // transmembrane protein C20orf108-like // --- // 10005456	LOC100054563	Cluster0004
15132804	ENSECAT00000022230 // LOC100054590 // 26S proteasome non-ATPase regulatory subunit 10-l	LOC100054590	Cluster0004
14997121	XM_001500901 // LOC100054643 // palmitoyltransferase ZDHHC3-like // --- // 100054643 //	LOC100054643	Cluster0004
15086945	ENSECAT00000018166 // LOC100054812 // alpha-endosulfine-like // --- // 100054812 /// XM	LOC100054812	Cluster0004
15000787	ENSECAT00000020565 // LOC100054832 // uncharacterized protein C3orf23-like // --- // 10	LOC100054832	Cluster0004
15110988	ENSECAT00000010256 // LOC100054949 // transcription activator BRG1-like // --- // 10005	LOC100054949	Cluster0004
15121392	ENSECAT00000014189 // LOC100055036 // transcription elongation factor A protein 1-like	LOC100055036	Cluster0004
14993934	XM_001502144 // LOC100055055 // GPN-loop GTPase 1-like // --- // 100055055 /// ENSECAT0	LOC100055055	Cluster0004
15086865	ENSECAT00000009525 // LOC100055075 // protein FAM63A-like // --- // 100055075 /// XM_00	LOC100055075	Cluster0004
14970674	XM_001916705 // LOC100055100 // stress-induced-phosphoprotein 1-like // --- // 10005510	LOC100055100	Cluster0004
15123833	XM_001489304 // LOC100055169 // protein virilizer homolog // --- // 100055169 /// ENSEC	LOC100055169	Cluster0004
14990415	XM_001502269 // LOC100055191 // protein phosphatase 1G-like // --- // 100055191 /// ENS	LOC100055191	Cluster0004
15065376	XM_001501541 // LOC100055295 // gamma-aminobutyric acid receptor-associated protein-lik	LOC100055295	Cluster0004
15004430	ENSECAT00000017277 // LOC100055368 // probable palmitoyltransferase ZDHHC20-like // ---	LOC100055368	Cluster0004
15075859	ENSECAT00000017712 // LOC100055381 // ribonuclease T2-like // --- // 100055381 /// XM_0	LOC100055381	Cluster0004
15082247	XM_001501128 // LOC100055464 // homocysteine-responsive endoplasmic reticulum-resident	LOC100055464	Cluster0004
15121838	XM_001490623 // LOC100055515 // plasma glutamate carboxypeptidase-like // --- // 100055	LOC100055515	Cluster0004
15008929	XM_001489698 // LOC100055572 // DNA replication licensing factor MCM6-like // --- // 10	LOC100055572	Cluster0004
15028988	XM_001502478 // LOC100055574 // cell division cycle 5-like protein-like // --- // 10005	LOC100055574	Cluster0004
14994866	XM_001489793 // LOC100055748 // transmembrane protein 43-like // --- // 100055748 /// E	LOC100055748	Cluster0004
15089727	XM_001489794 // LOC100055750 // retinoic acid receptor responder protein 1-like // ---	LOC100055750	Cluster0004
15073420	XM_001489827 // LOC100055808 // membrane magnesium transporter 1-like // --- // 1000558	LOC100055808	Cluster0004
14966168	XM_001502240 // LOC100055830 // vacuolar-sorting protein SNF8-like // --- // 100055830	LOC100055830	Cluster0004
15092647	XM_001918140 // LOC100055854 // RNA 3 -terminal phosphate cyclase-like // --- // 100055	LOC100055854	Cluster0004
14994890	ENSECAT00000000611 // LOC100055882 // uncharacterized protein C3orf19-like // --- // 10	LOC100055882	Cluster0004
15055616	ENSECAT00000005806 // LOC100055890 // GDP-fucose protein O-fucosyltransferase 2-like //	LOC100055890	Cluster0004
14957883	ENSECAT00000017181 // LOC100056149 // uncharacterized protein C17orf62 homolog // --- /	LOC100056149	Cluster0004
14971861	XM_001494530 // LOC100056151 // CD151 antigen-like // --- // 100056151 /// ENSECAT00000	LOC100056151	Cluster0004
15072344	ENSECAT00000016981 // LOC100056340 // syntaxin-18-like // --- // 100056340 /// XM_00191	LOC100056340	Cluster0004
15127104	ENSECAT00000007238 // LOC100056673 // synapse-associated protein 1-like // --- // 10005	LOC100056673	Cluster0004
14995006	XM_001492042 // LOC100056766 // 60S ribosomal protein L32-like // --- // 100056766 ///	LOC100056766	Cluster0004
15089287	XM_001915543 // LOC100056817 // protein CREG1-like // --- // 100056817 /// ENSECAT00000	LOC100056817	Cluster0004
15094398	XM_001496411 // LOC100056818 // rhomboid domain-containing protein 1-like // --- // 100	LOC100056818	Cluster0004
15122253	XM_001496257 // LOC100056859 // rRNA-processing protein UTP23 homolog // --- // 1000568	LOC100056859	Cluster0004
15061854	XM_001490463 // LOC100056911 // arylsulfatase A-like // --- // 100056911 /// ENSECAT000	LOC100056911	Cluster0004
15118490	XM_001493775 // LOC100057258 // endoplasmic reticulum resident protein 29-like // --- /	LOC100057258	Cluster0004
14939241	XM_001503583 // LOC100057403 // signal recognition particle 14 kDa protein-like // ---	LOC100057403	Cluster0004
15094978	XM_001500288 // LOC100057575 // NEDD8-conjugating enzyme UBE2F-like // --- // 100057575	LOC100057575	Cluster0004
15062832	ENSECAT00000025866 // LOC100057616 // la-related protein 4B-like // --- // 100057616 //	LOC100057616	Cluster0004
15118512	XM_001490910 // LOC100057622 // aldehyde dehydrogenase, mitochondrial-like // --- // 10	LOC100057622	Cluster0004
15115630	ENSECAT00000000828 // LOC100057795 // tyrosine-protein phosphatase non-receptor type 2-	LOC100057795	Cluster0004
15066795	XM_001916447 // LOC100057831 // n-acylethanolamine-hydrolyzing acid amidase-like // ---	LOC100057831	Cluster0004
14940520	ENSECAT00000020459 // LOC100057857 // mitochondrial ribonuclease P protein 3-like // --	LOC100057857	Cluster0004
15118546	ENSECAT00000007051 // LOC100057885 // SH2B adapter protein 3-like // --- // 100057885 /	LOC100057885	Cluster0004
14995078	XM_001493599 // LOC100057913 // protein SEC13 homolog // --- // 100057913 /// XM_003363	LOC100057913	Cluster0004
15038735	ENSECAT00000022008 // LOC100057922 // GTP-binding protein 5-like // --- // 100057922	LOC100057922	Cluster0004
14947653	ENSECAT00000025209 // LOC100057956 // uncharacterized protein C15orf29-like // --- // 1	LOC100057956	Cluster0004
14984401	ENSECAT00000000710 // LOC100058137 // protein RMD5 homolog B-like // --- // 100058137 /	LOC100058137	Cluster0004
15051929	ENSECAT00000022837 // LOC100058146 // proteasome-associated protein ECM29 homolog // --	LOC100058146	Cluster0004
15057113	ENSECAT00000017536 // LOC100058148 // protein MAK16 homolog // --- // 100058148 /// XM_	LOC100058148	Cluster0004
15057858	XM_001914713 // LOC100058283 // glioma pathogenesis-related protein 1-like // --- // 10	LOC100058283	Cluster0004
15056135	ENSECAT00000019124 // LOC100058323 // general transcription factor IIE subunit 2-like /	LOC100058323	Cluster0004
15114744	ENSECAT00000005674 // LOC100058336 // actin-related protein 2/3 complex subunit 3-like	LOC100058336	Cluster0004
15114749	XM_001915547 // LOC100058376 // anaphase-promoting complex subunit 7-like // --- // 100	LOC100058376	Cluster0004

15095505	ENSECAT00000020320 // LOC100058416 // probable fructose-2,6-bisphosphatase TIGAR-like /	LOC100058416	Cluster0004
14961836	XM_001504001 // LOC100058439 // protein Njmu-R1-like // --- // 100058439 /// ENSECAT000	LOC100058439	Cluster0004
15111388	XM_001491421 // LOC100058462 // mediator of RNA polymerase II transcription subunit 17-	LOC100058462	Cluster0004
15048243	XM_001491443 // LOC100058497 // cyclin-dependent kinase 2-interacting protein-like // -	LOC100058497	Cluster0004
14973924	ENSECAT00000014510 // LOC100058529 // probable palmitoyltransferase ZDHHC24-like // ---	LOC100058529	Cluster0004
15014054	XM_001496004 // LOC100058535 // mitochondrial import inner membrane translocase subunit	LOC100058535	Cluster0004
15101669	ENSECAT00000014273 // LOC100058626 // uncharacterized protein C12orf41 homolog // --- /	LOC100058626	Cluster0004
15133050	XM_003365863 // LOC100058639 // septin-6-like // --- // 100058639 /// XM_003365864 // L	LOC100058639	Cluster0004
15133769	XM_003365923 // LOC100058834 // b-cell receptor-associated protein 31-like // --- // 10	LOC100058834	Cluster0004
15129715	XM_001491785 // LOC100058835 // NADH dehydrogenase [ubiquinone] 1 alpha subcomplex subu	LOC100058835	Cluster0004
15030704	XM_001491355 // LOC100058853 // chloride intracellular channel protein 1-like // --- //	LOC100058853	Cluster0004
15102574	XM_001504780 // LOC100058867 // SAP domain-containing ribonucleoprotein-like // --- //	LOC100058867	Cluster0004
15130398	XM_001491684 // LOC100058868 // ATP-binding cassette sub-family D member 1-like // ---	LOC100058868	Cluster0004
15092242	XM_001917929 // LOC100058893 // ATP synthase subunit b, mitochondrial-like // --- // 10	LOC100058893	Cluster0004
15073024	XM_001490466 // LOC100058947 // probable saccharopine dehydrogenase-like // --- // 1000	LOC100058947	Cluster0004
14961954	ENSECAT00000016594 // LOC100058999 // transmembrane protein 199-like // --- // 10005899	LOC100058999	Cluster0004
15118885	ENSECAT00000006505 // LOC100059094 // RILP-like protein 1-like // --- // 100059094 ///	LOC100059094	Cluster0004
15051784	ENSECAT00000008409 // LOC100059122 // transmembrane protein C9orf5-like // --- // 10005	LOC100059122	Cluster0004
14974675	XM_001493487 // LOC100059133 // aminoacyl tRNA synthase complex-interacting multifuncti	LOC100059133	Cluster0004
15115047	XM_001498352 // LOC100059174 // general transcription factor IIH subunit 3-like // ---	LOC100059174	Cluster0004
15119431	ENSECAT00000027140 // LOC100059211 // uncharacterized protein C18orf8-like // --- // 10	LOC100059211	Cluster0004
14991601	ENSECAT00000016576 // LOC100059384 // phosducin-like protein 3-like // --- // 100059384	LOC100059384	Cluster0004
15054134	XM_001491997 // LOC100059393 // tryptophan-rich protein-like // --- // 100059393 /// EN	LOC100059393	Cluster0004
15087365	XM_001500218 // LOC100059510 // suppressor of IKBKE 1-like // --- // 100059510 /// ENSE	LOC100059510	Cluster0004
15095563	XM_001492075 // LOC100059512 // CD9 antigen-like // --- // 100059512 /// ENSECAT0000000	LOC100059512	Cluster0004
15074470	XM_001492101 // LOC100059547 // ubiquitin carboxyl-terminal hydrolase isozyme L5-like /	LOC100059547	Cluster0004
15114785	ENSECAT00000023915 // LOC100059653 // e3 ubiquitin-protein ligase RNF34-like // --- //	LOC100059653	Cluster0004
15098299	XM_001492223 // LOC100059724 // YEATS domain-containing protein 4-like // --- // 100059	LOC100059724	Cluster0004
14962228	ENSECAT00000026314 // LOC100059869 // glyoxalase domain-containing protein 4-like // --	LOC100059869	Cluster0004
14974280	ENSECAT00000002627 // LOC100060043 // nucleosome assembly protein 1-like 4-like // ---	LOC100060043	Cluster0004
14985152	XM_003362783 // LOC100060090 // ras GTPase-activating protein-binding protein 1-like //	LOC100060090	Cluster0004
15014573	XM_001501086 // LOC100060183 // serine/threonine-protein kinase PAK 2-like // --- // 10	LOC100060183	Cluster0004
15042010	ENSECAT00000015073 // LOC100060288 // e3 ubiquitin-protein ligase UHRF2-like // --- //	LOC100060288	Cluster0004
15129119	XM_001492629 // LOC100060331 // 60S ribosomal protein L36a-like // --- // 100060331 ///	LOC100060331	Cluster0004
14967935	XM_001504727 // LOC100060592 // tax1-binding protein 3-like // --- // 100060592 /// ENS	LOC100060592	Cluster0004
14944174	XM_001492833 // LOC100060623 // zinc finger RAD18 domain-containing protein C1orf124 ho	LOC100060623	Cluster0004
15055292	XM_001492982 // LOC100060848 // Down syndrome critical region protein 3-like // --- //	LOC100060848	Cluster0004
14972946	ENSECAT00000015673 // LOC100060920 // 39S ribosomal protein L16, mitochondrial-like //	LOC100060920	Cluster0004
15014887	XM_001917233 // LOC100060927 // general transcription factor IIE subunit 1-like // ---	LOC100060927	Cluster0004
15005251	XM_001493104 // LOC100061031 // response gene to complement 32 protein-like // --- // 1	LOC100061031	Cluster0004
15060049	XM_001493279 // LOC100061266 // thyroid transcription factor 1-associated protein 26-li	LOC100061266	Cluster0004
14971677	XM_001497566 // LOC100061284 // glutamine-dependent NAD(+) synthetase-like // --- // 10	LOC100061284	Cluster0004
14982551	XM_001503978 // LOC100061405 // histone deacetylase 3-like // --- // 100061405 /// ENSE	LOC100061405	Cluster0004
15084470	XM_001493405 // LOC100061486 // calcyclin-binding protein-like // --- // 100061486 ///	LOC100061486	Cluster0004
14936868	XM_001493472 // LOC100061580 // tyrosine-protein phosphatase non-receptor type 9-like /	LOC100061580	Cluster0004
15128159	ENSECAT00000020951 // LOC100061736 // structural maintenance of chromosomes protein 1A-	LOC100061736	Cluster0004
15020213	XM_001500334 // LOC100061820 // peptidyl-prolyl cis-trans isomerase D-like // --- // 10	LOC100061820	Cluster0004
15015333	XM_001503437 // LOC100061942 // PEST proteolytic signal-containing nuclear protein-like	LOC100061942	Cluster0004
15039221	XM_001490636 // LOC100062054 // DNA-directed RNA polymerase III subunit RPC6-like // --	LOC100062054	Cluster0004
14942766	ENSECAT00000023581 // LOC100062076 // ribonuclease P protein subunit p30-like // --- //	LOC100062076	Cluster0004
14975583	XM_001493817 // LOC100062129 // eukaryotic translation initiation factor 4H-like // ---	LOC100062129	Cluster0004
14975592	XM_001493840 // LOC100062168 // linker for activation of T-cells family member 2-like /	LOC100062168	Cluster0004
15134063	XM_001498280 // LOC100062170 // trimethyllysine dioxygenase, mitochondrial-like // ---	LOC100062170	Cluster0004
15068877	ENSECAT00000009689 // LOC100062226 // protein FAM192A-like // --- // 100062226	LOC100062226	Cluster0004

15073964	ENSECAT00000008202 // LOC100062283 // PPPDE peptidase domain-containing protein 1-like	LOC100062283	Cluster0004
15102361	XM_001504559 // LOC100062404 // chromobox protein homolog 5-like // --- // 100062404 //	LOC100062404	Cluster0004
14936980	XM_001494059 // LOC100062522 // ubiquitin-like protein 7-like // --- // 100062522 /// X	LOC100062522	Cluster0004
14936990	XM_001917675 // LOC100062559 // semaphorin-7A-like // --- // 100062559 /// ENSECAT00000	LOC100062559	Cluster0004
15106847	ENSECAT00000015914 // LOC100063117 // methylosome subunit pICln-like // --- // 10006311	LOC100063117	Cluster0004
15083600	ENSECAT00000011847 // LOC100063507 // ATP-binding cassette sub-family F member 2-like /	LOC100063507	Cluster0004
14979333	XM_001501595 // LOC100063884 // transmembrane protein C16orf54-like // --- // 100063884	LOC100063884	Cluster0004
15117677	ENSECAT00000019181 // LOC100063907 // mitochondrial fission process protein 1-like // -	LOC100063907	Cluster0004
15122128	XM_001494974 // LOC100063908 // tetratricopeptide repeat protein 35-like // --- // 1000	LOC100063908	Cluster0004
14935685	ENSECAT00000008181 // LOC100064139 // glutamate dehydrogenase 1, mitochondrial-like //	LOC100064139	Cluster0004
14957348	ENSECAT00000013069 // LOC100064179 // uncharacterized LOC100064179 // --- // 100064179	LOC100064179	Cluster0004
15110324	XM_001505075 // LOC100064265 // transmembrane protein 218-like // --- // 100064265 ///	LOC100064265	Cluster0004
14976061	ENSECAT00000018947 // LOC100064282 // battenin-like // --- // 100064282 /// XM_00150211	LOC100064282	Cluster0004
14979443	ENSECAT00000021076 // LOC100064341 // elongation factor Tu, mitochondrial-like // --- /	LOC100064341	Cluster0004
15039343	ENSECAT00000019651 // LOC100064380 // UPF0492 protein C20orf94-like // --- // 100064380	LOC100064380	Cluster0004
15101022	XM_001502728 // LOC100064589 // cell cycle regulator Mat89Bb homolog // --- // 10006458	LOC100064589	Cluster0004
15000554	ENSECAT00000026507 // LOC100064728 // kelch-like protein 18-like // --- // 100064728 //	LOC100064728	Cluster0004
15076896	XM_001915738 // LOC100064761 // biliverdin reductase A-like // --- // 100064761 /// ENS	LOC100064761	Cluster0004
15101070	XM_001502934 // LOC100064789 // endoplasmic reticulum-Golgi intermediate compartment pr	LOC100064789	Cluster0004
14932645	XM_001495606 // LOC100064829 // non-structural maintenance of chromosomes element 4 hom	LOC100064829	Cluster0004
15093182	XM_001497167 // LOC100064844 // ribosome production factor 1-like // --- // 100064844 /	LOC100064844	Cluster0004
15067753	XM_001495692 // LOC100064945 // RELT-like protein 1-like // --- // 100064945 /// ENSECA	LOC100064945	Cluster0004
15077709	ENSECAT00000015810 // LOC100064947 // transmembrane protein 106B-like // --- // 1000649	LOC100064947	Cluster0004
15082918	XM_001500519 // LOC100065055 // modulator of retrovirus infection homolog // --- // 100	LOC100065055	Cluster0004
15080786	XM_001495776 // LOC100065072 // nudC domain-containing protein 3-like // --- // 1000650	LOC100065072	Cluster0004
14965195	ENSECAT00000020237 // LOC100065120 // methyltransferase-like protein 2A-like // --- //	LOC100065120	Cluster0004
15057734	XM_001916797 // LOC100065372 // protein CLN8-like // --- // 100065372 /// ENSECAT000000	LOC100065372	Cluster0004
15131984	ENSECAT00000012239 // LOC100065450 // oligophrenin-1-like // --- // 100065450 /// XM_00	LOC100065450	Cluster0004
14984044	XM_001504660 // LOC100065465 // tubulin-specific chaperone A-like // --- // 100065465 /	LOC100065465	Cluster0004
15035640	XM_001496286 // LOC100065794 // UPF0600 protein C5orf51-like // --- // 100065794 /// EN	LOC100065794	Cluster0004
14952575	XM_001503886 // LOC100066096 // heat shock protein 67B2-like // --- // 100066096 /// EN	LOC100066096	Cluster0004
15039501	XM_001915990 // LOC100066127 // pantothenate kinase 2, mitochondrial-like // --- // 100	LOC100066127	Cluster0004
15128390	ENSECAT00000000280 // LOC100066275 // protein YIPF6-like // --- // 100066275 /// XM_001	LOC100066275	Cluster0004
15070378	XM_001496615 // LOC100066296 // methionine aminopeptidase 1-like // --- // 100066296 //	LOC100066296	Cluster0004
15116156	XM_001496619 // LOC100066302 // e3 ubiquitin-protein ligase RNF138-like // --- // 10006	LOC100066302	Cluster0004
14956880	XM_001503938 // LOC100066344 // autophagy protein 5-like // --- // 100066344 /// XM_001	LOC100066344	Cluster0004
15039553	XM_001496720 // LOC100066461 // attractin-like // --- // 100066461 /// ENSECAT000000179	LOC100066461	Cluster0004
15100599	XM_001496928 // LOC100066737 // heme-binding protein 1-like // --- // 100066737 /// ENS	LOC100066737	Cluster0004
15096038	ENSECAT00000019529 // LOC100066759 // uncharacterized protein KIAA1467-like // --- // 1	LOC100066759	Cluster0004
15088609	XM_001496963 // LOC100066781 // GPI-anchor transamidase-like // --- // 100066781 /// EN	LOC100066781	Cluster0004
15011858	ENSECAT00000009103 // LOC100066877 // YEATS domain-containing protein 2-like // --- //	LOC100066877	Cluster0004
15119950	ENSECAT00000021963 // LOC100067081 // zinc finger protein 396-like // --- // 100067081	LOC100067081	Cluster0004
15076420	XM_001497243 // LOC100067124 // peroxisomal biogenesis factor 3-like // --- // 10006712	LOC100067124	Cluster0004
15028723	ENSECAT00000006940 // LOC100067213 // kelch domain-containing protein 3-like // --- //	LOC100067213	Cluster0004
15093351	ENSECAT00000021689 // LOC100067295 // geranylgeranyl transferase type-2 subunit beta-li	LOC100067295	Cluster0004
15124520	XM_001497477 // LOC100067415 // putative deoxyribonuclease TATDN1-like // --- // 100067	LOC100067415	Cluster0004
15011264	XM_001505112 // LOC100067464 // ribosome biogenesis protein WDR12-like // --- // 100067	LOC100067464	Cluster0004
14932809	ENSECAT00000007590 // LOC100067493 // shootin-1-like // --- // 100067493 /// XM_0014947	LOC100067493	Cluster0004
15095129	XM_001497625 // LOC100067637 // protein phosphatase 1 regulatory subunit 7-like // ---	LOC100067637	Cluster0004
15031767	XM_001502257 // LOC100067678 // 39S ribosomal protein L14, mitochondrial-like // --- //	LOC100067678	Cluster0004
14965825	ENSECAT00000021406 // LOC100067814 // protein CASC3-like // --- // 100067814 /// XM_001	LOC100067814	Cluster0004
15103994	ENSECAT00000011477 // LOC100067852 // centrosomal protein of 57 kDa-like // --- // 1000	LOC100067852	Cluster0004
15011129	ENSECAT00000015574 // LOC100067910 // protein FAM126B-like // --- // 100067910 /// XM_0	LOC100067910	Cluster0004
15122520	ENSECAT00000023507 // LOC100068097 // myc proto-oncogene protein-like // --- // 1000680	LOC100068097	Cluster0004

14956056	XM_001498010 // LOC100068130 // sialin-like // --- // 100068130 /// ENSECAT00000016980	LOC100068130	Cluster0004
15058712	XM_001498677 // LOC100068154 // WASH complex subunit 7-like // --- // 100068154 /// ENS	LOC100068154	Cluster0004
15043902	ENSECAT00000019100 // LOC100068266 // BAG family molecular chaperone regulator 1-like /	LOC100068266	Cluster0004
15016678	XM_001503336 // LOC100068301 // palmitoyl-protein thioesterase 1-like // --- // 1000683	LOC100068301	Cluster0004
15082886	XM_001498216 // LOC100068361 // coiled-coil-helix-coiled-coil-helix domain-containing p	LOC100068361	Cluster0004
15062274	ENSECAT00000021501 // LOC100068422 // 3-hydroxyacyl-CoA dehydratase 1-like // --- // 10	LOC100068422	Cluster0004
15050887	XM_001498302 // LOC100068463 // ubiquitin-associated domain-containing protein 1-like /	LOC100068463	Cluster0004
15124597	XM_003365630 // LOC100068561 // protein FAM49B-like // --- // 100068561 /// ENSECAT0000	LOC100068561	Cluster0004
14984544	XM_001498575 // LOC100068758 // eukaryotic translation initiation factor 4E type 1B-lik	LOC100068758	Cluster0004
14992348	ENSECAT00000011523 // LOC100068778 // 39S ribosomal protein L19, mitochondrial-like //	LOC100068778	Cluster0004
15016753	ENSECAT00000015888 // LOC100068889 // rhomboid-related protein 2-like // --- // 1000688	LOC100068889	Cluster0004
15124723	ENSECAT00000025889 // LOC100068961 // src-like-adapter-like // --- // 100068961 /// XM_	LOC100068961	Cluster0004
15100973	ENSECAT00000000404 // LOC100068969 // uncharacterized LOC100068969 // --- // 100068969	LOC100068969	Cluster0004
14956167	XM_003362360 // LOC100068991 // CDGSH iron-sulfur domain-containing protein 1-like // -	LOC100068991	Cluster0004
15008431	XM_001498888 // LOC100069061 // cyclin-Y-like protein 1-like // --- // 100069061 /// EN	LOC100069061	Cluster0004
15050362	XM_001499226 // LOC100069734 // exosome complex component RRP4-like // --- // 100069734	LOC100069734	Cluster0004
15109536	ENSECAT00000025826 // LOC100069813 // KDEL motif-containing protein 2-like // --- // 10	LOC100069813	Cluster0004
15052925	ENSECAT00000020276 // LOC100069900 // uncharacterized protein C9orf78-like // --- // 10	LOC100069900	Cluster0004
14966304	ENSECAT00000019226 // LOC100069924 // leucine-rich repeat-containing protein 59-like //	LOC100069924	Cluster0004
15025522	XM_001499727 // LOC100070044 // transmembrane protein 131-like // --- // 100070044 ///	LOC100070044	Cluster0004
14933286	ENSECAT00000021996 // LOC100070070 // UPF0668 protein C10orf76-like // --- // 100070070	LOC100070070	Cluster0004
15061101	XM_001499842 // LOC100070178 // SUN domain-containing protein 2-like // --- // 10007017	LOC100070178	Cluster0004
15126744	XM_003365690 // LOC100070253 // protein cappuccino homolog // --- // 100070253 /// ENSE	LOC100070253	Cluster0004
15069971	ENSECAT00000010421 // LOC100070274 // palmitoyltransferase ZDHHC7-like // --- // 100070	LOC100070274	Cluster0004
15063713	ENSECAT00000009117 // LOC100070366 // uncharacterized protein C10orf18-like // --- // 1	LOC100070366	Cluster0004
15012696	XM_001500027 // LOC100070376 // integrin beta-5-like // --- // 100070376 /// ENSECAT000	LOC100070376	Cluster0004
15104426	ENSECAT00000013223 // LOC100070452 // uncharacterized protein C11orf52-like // --- // 1	LOC100070452	Cluster0004
15059238	ENSECAT00000024097 // LOC100070463 // small G protein signaling modulator 3-like // ---	LOC100070463	Cluster0004
15088939	XM_001500154 // LOC100070493 // TM2 domain-containing protein 1-like // --- // 10007049	LOC100070493	Cluster0004
14990219	XM_001500160 // LOC100070497 // protein FAM98A-like // --- // 100070497 /// ENSECAT0000	LOC100070497	Cluster0004
14946966	XM_001918213 // LOC100070638 // uncharacterized LOC100070638 // --- // 100070638 /// EN	LOC100070638	Cluster0004
15040444	ENSECAT00000021700 // LOC100070847 // mitochondrial import receptor subunit TOM34-like	LOC100070847	Cluster0004
15023050	XM_001500535 // LOC100070854 // protein FAM76A-like // --- // 100070854 /// ENSECAT0000	LOC100070854	Cluster0004
15032827	ENSECAT00000012102 // LOC100070946 // pyroglutamyl-peptidase 1-like // --- // 100070946	LOC100070946	Cluster0004
15013076	ENSECAT00000024846 // LOC100071372 // n-alpha-acetyltransferase 50, NatE catalytic subu	LOC100071372	Cluster0004
15023455	XM_001501495 // LOC100071667 // complement C1q subcomponent subunit B-like // --- // 10	LOC100071667	Cluster0004
14985324	XM_001503782 // LOC100071700 // grpE protein homolog 2, mitochondrial-like // --- // 10	LOC100071700	Cluster0004
15128846	XM_001501152 // LOC100071723 // SH3 domain-binding glutamic acid-rich-like protein-like	LOC100071723	Cluster0004
15128752	XM_001501735 // LOC100071798 // UPF0368 protein Cxorf26-like // --- // 100071798 /// EN	LOC100071798	Cluster0004
14952827	ENSECAT00000014894 // LOC100072366 // general transcription factor 3C polypeptide 6-lik	LOC100072366	Cluster0004
14994560	XM_001502466 // LOC100072510 // cleavage and polyadenylation specificity factor subunit	LOC100072510	Cluster0004
14994617	ENSECAT00000010440 // LOC100072610 // radical S-adenosyl methionine domain-containing p	LOC100072610	Cluster0004
14982921	XM_001502713 // LOC100072697 // uncharacterized LOC100072697 // --- // 100072697 /// EN	LOC100072697	Cluster0004
14943290	XM_001502747 // LOC100072720 // inorganic pyrophosphatase-like // --- // 100072720 ///	LOC100072720	Cluster0004
14968155	ENSECAT00000026927 // LOC100072742 // f-box only protein 39-like // --- // 100072742 //	LOC100072742	Cluster0004
14962593	ENSECAT00000010791 // LOC100072797 // putative ATP-dependent RNA helicase DHX33-like //	LOC100072797	Cluster0004
14934706	ENSECAT00000002783 // LOC100072806 // anaphase-promoting complex subunit 16-like // ---	LOC100072806	Cluster0004
14962788	ENSECAT00000007266 // LOC100072954 // dermal papilla-derived protein 6-like // --- // 1	LOC100072954	Cluster0004
14957594	XM_001503335 // LOC100073104 // syntaxin-7-like // --- // 100073104 /// ENSECAT00000021	LOC100073104	Cluster0004
14986533	XM_001918095 // LOC100073158 // AP-3 complex subunit sigma-1-like // --- // 100073158 /	LOC100073158	Cluster0004
14983384	XM_003362836 // LOC100073160 // ubiquitin-like protein ATG12-like // --- // 100073160 /	LOC100073160	Cluster0004
14986785	ENSECAT00000012224 // LOC100073217 // membrane protein FAM174A-like // --- // 100073217	LOC100073217	Cluster0004
15125111	ENSECAT00000018149 // LOC100147640 // plectin-like // --- // 100147640 /// ENSECAT00000	LOC100147640	Cluster0004
15028599	XM_003363713 // LOC100629249 // mitochondrial import receptor subunit TOM6 homolog // -	LOC100629249	Cluster0004

15051612	ENSECAT00000002358 // LOC100629329 // 39S ribosomal protein L50, mitochondrial-like //	LOC100629329	Cluster0004
14961595	ENSECAT00000024855 // LOC100629929 // zinc finger HIT domain-containing protein 3-like	LOC100629929	Cluster0004
15055596	XM_003364190 // LOC100630038 // pituitary tumor-transforming gene 1 protein-interacting	LOC100630038	Cluster0004
14985108	XM_003362867 // LOC100630302 // histone deacetylase complex subunit SAP30L-like // ---	LOC100630302	Cluster0004
14999599	XM_003363126 // LOC100630485 // ribonuclease P protein subunit p14-like // --- // 10063	LOC100630485	Cluster0004
15093592	XM_003365065 // LOC100630509 // leptin receptor gene-related protein-like // --- // 100	LOC100630509	Cluster0004
15130532	XM_003365909 // LOC100630700 // protein FAM50A-like // --- // 100630700 /// ENSECAT0000	LOC100630700	Cluster0004
15081257	XM_003364824 // LOC100630906 // transmembrane protein C7orf23 homolog // --- // 1006309	LOC100630906	Cluster0004
15092153	XM_001495327 // LRIG2 // leucine-rich repeats and immunoglobulin-like domains 2 // ---	LRIG2	Cluster0004
14984323	ENSECAT00000026630 // MAML1 // mastermind-like 1 (Drosophila) // --- // 100067311 /// X	MAML1	Cluster0004
15046385	XM_001492048 // MARK3 // MAP/microtubule affinity-regulating kinase 3 // --- // 1000594	MARK3	Cluster0004
15097862	XM_001488891 // MARS // methionyl-tRNA synthetase // --- // 100053373 /// ENSECAT000000	MARS	Cluster0004
15088442	ENSECAT00000011655 // MCOLN2 // mucolipin 2 // --- // 100052719 /// XM_001496833 // MCO	MCOLN2	Cluster0004
15131392	XM_001489243 // MED14 // mediator complex subunit 14 // --- // 100050409 /// ENSECAT000	MED14	Cluster0004
14967812	ENSECAT00000014229 // METTL16 // methyltransferase like 16 // --- // 100060313 /// XM_0	METTL16	Cluster0004
15024052	ENSECAT00000017643 // MFN2 // mitofusin 2 // --- // ---	MFN2	Cluster0004
15020790	ENSECAT00000007596 // MFSD8 // major facilitator superfamily domain containing 8 // ---	MFSD8	Cluster0004
15077657	ENSECAT00000023405 // MIOS // missing oocyte, meiosis regulator, homolog (Drosophila) /	MIOS	Cluster0004
15132189	NR_033090 // MIR421 // microRNA mir-421 // --- // 100314944	MIR421	Cluster0004
14933630	ENSECAT00000010382 // MMS19 // MMS19 nucleotide excision repair homolog (S. cerevisiae)	MMS19	Cluster0004
15113666	ENSECAT00000017550 // MORC2 // MORC family CW-type zinc finger 2 // --- // 100063412 //	MORC2	Cluster0004
15127040	XM_001917193 // MOSPD2 // motile sperm domain containing 2 // --- // 100055695 /// ENSE	MOSPD2	Cluster0004
15133995	ENSECAT00000010186 // MPP1 // membrane protein, palmitoylated 1, 55kDa // --- // 100063	MPP1	Cluster0004
15057294	ENSECAT00000011779 // MSR1 // macrophage scavenger receptor 1 // --- // 100053116 /// X	MSR1	Cluster0004
15026873	ENSECAT00000021397 // MYLIP // myosin regulatory light chain interacting protein // ---	MYLIP	Cluster0004
15032580	ENSECAT00000012036 // MYO9B // myosin IXB // --- // 100069995 /// ENSECAT00000012043 //	MYO9B	Cluster0004
15114631	ENSECAT00000009442 // NAA25 // N(alpha)-acetyltransferase 25, NatB auxiliary subunit //	NAA25	Cluster0004
14977041	ENSECAT00000012599 // NAGPA // N-acetylglucosamine-1-phosphodiester alpha-N-acetylgluco	NAGPA	Cluster0004
14965520	ENSECAT00000007802 // NBR1 // neighbor of BRCA1 gene 1 // --- // 100051928 /// XM_00336	NBR1	Cluster0004
15097390	ENSECAT00000016276 // NCKAP1L // NCK-associated protein 1-like // --- // 100062541	NCKAP1L	Cluster0004
15058287	ENSECAT00000014574 // NEDD1 // neural precursor cell expressed, developmentally down-re	NEDD1	Cluster0004
15063737	ENSECAT00000022811 // NET1 // neuroepithelial cell transforming 1 // --- // 100070450 /	NET1	Cluster0004
14941610	XM_001495643 // NHLRC2 // NHL repeat containing 2 // --- // 100059070 /// ENSECAT000000	NHLRC2	Cluster0004
15008098	XM_001497941 // NIF3L1 // NIF3 NGG1 interacting factor 3-like 1 (S. cerevisiae) // ---	NIF3L1	Cluster0004
14936613	XM_003363641 // NIPA2 // non imprinted in Prader-Willi/Angelman syndrome 2 // --- // 10	NIPA2	Cluster0004
15035731	ENSECAT00000020680 // NIPBL // Nipped-B homolog (Drosophila) // --- // 100053547	NIPBL	Cluster0004
14961930	XM_001504107 // NLK // nemo-like kinase // --- // 100058876 /// ENSECAT00000010814 // N	NLK	Cluster0004
14933917	XM_001917312 // NOC3L // nucleolar complex associated 3 homolog (S. cerevisiae) // ---	NOC3L	Cluster0004
14942103	ENSECAT00000026677 // NOLC1 // nucleolar and coiled-body phosphoprotein 1 // --- // 100	NOLC1	Cluster0004
15099989	ENSECAT00000013305 // NOP2 // NOP2 nucleolar protein homolog (yeast) // --- // 10005220	NOP2	Cluster0004
14951332	ENSECAT00000022344 // NR1H2 // nuclear receptor subfamily 1, group H, member 2 // --- /	NR1H2	Cluster0004
15003376	ENSECAT00000013896 // NUFIP1 // nuclear fragile X mental retardation protein interactin	NUFIP1	Cluster0004
15033653	XM_001499483 // NUP155 // nucleoporin 155kDa // --- // 100053405 /// ENSECAT00000007933	NUP155	Cluster0004
15073203	ENSECAT00000015379 // NVL // nuclear VCP-like // --- // 100055652 /// XM_001489738 // N	NVL	Cluster0004
15129440	ENSECAT00000015766 // NXT2 // nuclear transport factor 2-like export factor 2 // --- //	NXT2	Cluster0004
15009986	XM_001499353 // OLA1 // Obg-like ATPase 1 // --- // 100053204 /// ENSECAT00000017411 //	OLA1	Cluster0004
15012621	XM_001501564 // OSBPL11 // oxysterol binding protein-like 11 // --- // 100060459 /// EN	OSBPL11	Cluster0004
15097619	XM_001504804 // PA2G4 // proliferation-associated 2G4, 38kDa // --- // 100051912 /// EN	PA2G4	Cluster0004
15071438	ENSECAT00000021255 // PAICS // phosphoribosylaminoimidazole carboxylase, phosphoribosyl	PAICS	Cluster0004
15006036	ENSECAT00000006281 // PCID2 // PCI domain containing 2 // --- // 100067141 /// XM_00149	PCID2	Cluster0004
14937477	ENSECAT00000028929 // PDCD7 // programmed cell death 7 // --- // ---	PDCD7	Cluster0004
14969442	XM_001503314 // PDHX // pyruvate dehydrogenase complex, component X // --- // 100059260	PDHX	Cluster0004
15002886	ENSECAT00000021365 // PDS5B // PDS5, regulator of cohesion maintenance, homolog B (S. c	PDS5B	Cluster0004
15020371	XM_001501357 // PET112 // PET112 homolog (yeast) // --- // 100062330 /// ENSECAT0000000	PET112	Cluster0004

14942357	XM_001500347 // PI4K2A // phosphatidylinositol 4-kinase type 2 alpha // --- // 10007068	PI4K2A	Cluster0004
14981112	XM_001915851 // PIGQ // phosphatidylinositol glycan anchor biosynthesis, class Q // ---	PIGQ	Cluster0004
15112875	XM_001504950 // PIK3C2A // phosphoinositide-3-kinase, class 2, alpha polypeptide // ---	PIK3C2A	Cluster0004
15116337	XM_001497768 // PIK3C3 // phosphoinositide-3-kinase, class 3 // --- // 100053039 /// EN	PIK3C3	Cluster0004
15093028	ENSECAT00000026949 // PKN2 // protein kinase N2 // --- // 100052266 /// XM_001495405 //	PKN2	Cluster0004
15085179	ENSECAT00000026856 // PLA2G4A // phospholipase A2, group IVA (cytosolic, calcium-depend	PLA2G4A	Cluster0004
14941242	XM_001490808 // PLEKHA1 // pleckstrin homology domain containing, family A (phosphoinos	PLEKHA1	Cluster0004
14959587	ENSECAT00000027021 // PLEKHM1 // pleckstrin homology domain containing, family M (with	PLEKHM1	Cluster0004
14978575	XM_001504456 // PLOD3 // procollagen-lysine, 2-oxoglutarate 5-dioxygenase 3 // --- // 1	PLOD3	Cluster0004
15086766	ENSECAT00000023214 // POGZ // pogo transposable element with ZNF domain // --- // ---	POGZ	Cluster0004
15096663	ENSECAT00000016911 // PPHLN1 // periphilin 1 // --- // 100054208 /// XM_001488998 // PP	PPHLN1	Cluster0004
15059978	ENSECAT00000021971 // PPP1R12A // protein phosphatase 1, regulatory subunit 12A // ---	PPP1R12A	Cluster0004
15109600	ENSECAT00000012956 // PPP2R1B // protein phosphatase 2, regulatory subunit A, beta // -	PPP2R1B	Cluster0004
15028709	XM_001501546 // PPP2R5D // protein phosphatase 2, regulatory subunit B , delta // --- /	PPP2R5D	Cluster0004
15040678	ENSECAT00000015862 // PREX1 // phosphatidylinositol-3,4,5-trisphosphate-dependent Rac e	PREX1	Cluster0004
15033530	NM_001081803 // PRKAA1 // protein kinase, AMP-activated, alpha 1 catalytic subunit // 2	PRKAA1	Cluster0004
15106872	XM_001495142 // PRKRIR // protein-kinase, interferon-inducible double stranded RNA depe	PRKRIR	Cluster0004
15064818	ENSECAT00000009896 // PSKH1 // protein serine kinase H1 // --- // 100053657 /// XM_0014	PSKH1	Cluster0004
15051976	ENSECAT00000005057 // PTGR1 // prostaglandin reductase 1 // --- // ---	PTGR1	Cluster0004
15029811	XM_001916775 // RANBP9 // RAN binding protein 9 // --- // 100065036 /// ENSECAT00000004	RANBP9	Cluster0004
15066609	XM_001915658 // RASGEF1B // RasGEF domain family, member 1B // --- // 100146797 /// ENS	RASGEF1B	Cluster0004
15040128	NM_001171746 // RBM12 // RNA binding motif protein 12 // --- // 100069590 /// ENSECAT00	RBM12	Cluster0004
15092311	XM_001496217 // RBM15 // RNA binding motif protein 15 // --- // 100058541 /// ENSECAT00	RBM15	Cluster0004
14985461	ENSECAT00000001501 // RBM27 // RNA binding motif protein 27 // --- // 100061055 /// XM_	RBM27	Cluster0004
15000314	XM_001496577 // RBM5 // RNA binding motif protein 5 // --- // 100052535 /// ENSECAT0000	RBM5	Cluster0004
15003247	XM_001489540 // RCBTB2 // regulator of chromosome condensation (RCC1) and BTB (POZ) dom	RCBTB2	Cluster0004
15018315	ENSECAT00000026258 // RCC2 // regulator of chromosome condensation 2 // --- // ---	RCC2	Cluster0004
15089612	ENSECAT00000014284 // RFWD2 // ring finger and WD repeat domain 2, E3 ubiquitin protein	RFWD2	Cluster0004
15069661	XM_001501213 // RFWD3 // ring finger and WD repeat domain 3 // --- // 100055114 /// ENS	RFWD3	Cluster0004
14967060	ENSECAT00000010249 // RHOT1 // ras homolog family member T1 // --- // ---	RHOT1	Cluster0004
14974541	ENSECAT00000005305 // RIC8A // resistance to inhibitors of cholinesterase 8 homolog A (	RIC8A	Cluster0004
15058797	XM_001499080 // RIC8B // resistance to inhibitors of cholinesterase 8 homolog B (C. ele	RIC8B	Cluster0004
14988632	XM_001497977 // RNF103 // ring finger protein 103 // --- // 100052907 /// ENSECAT000000	RNF103	Cluster0004
15112051	XM_001496568 // RNF121 // ring finger protein 121 // --- // 100066221 /// ENSECAT000000	RNF121	Cluster0004
15048945	ENSECAT00000019819 // RNF20 // ring finger protein 20, E3 ubiquitin protein ligase // -	RNF20	Cluster0004
15102647	ENSECAT00000006650 // RNF41 // ring finger protein 41 // --- // 100052095 /// XM_001504	RNF41	Cluster0004
14956463	XM_001503749 // RNGTT // RNA guanylyltransferase and 5 -phosphatase // --- // 100065700	RNGTT	Cluster0004
14992055	XM_001497468 // RPIA // ribose 5-phosphate isomerase A // --- // 100052643 /// ENSECAT0	RPIA	Cluster0004
15061147	XM_001501907 // RPL3 // ribosomal protein L3 // --- // 100070291 /// ENSECAT00000022268	RPL3	Cluster0004
15090103	ENSECAT00000023249 // RPS6KC1 // ribosomal protein S6 kinase, 52kDa, polypeptide 1 // -	RPS6KC1	Cluster0004
15106830	XM_001493213 // RSF1 // remodeling and spacing factor 1 // --- // 100051507 /// ENSECAT	RSF1	Cluster0004
15101440	ENSECAT00000025634 // SCAF11 // SR-related CTD-associated factor 11 // --- // ---	SCAF11	Cluster0004
15066772	ENSECAT00000017448 // SDAD1 // SDA1 domain containing 1 // --- // 100057877 /// XM_0014	SDAD1	Cluster0004
15026400	ENSECAT00000017682 // SEC24B // SEC24 family, member B (S. cerevisiae) // --- // ---	SEC24B	Cluster0004
14952189	ENSECAT00000022431 // SENP6 // SUMO1/sentrin specific peptidase 6 // --- // ---	SENP6	Cluster0004
15062525	NM_001190419 // SEPHS1 // selenophosphate synthetase 1 // --- // 100056604 /// ENSECAT0	SEPHS1	Cluster0004
15033431	NM_001135605 // SEPP1 // selenoprotein P, plasma, 1 // --- // 100052968 /// ENSECAT0000	SEPP1	Cluster0004
15075659	ENSECAT00000013902 // SERAC1 // serine active site containing 1 // --- // 100051068 ///	SERAC1	Cluster0004
14971165	ENSECAT00000026515 // SF3B2 // splicing factor 3b, subunit 2, 145kDa // --- // 10005217	SF3B2	Cluster0004
15123383	ENSECAT00000023014 // SGK3 // serum/glucocorticoid regulated kinase family, member 3 //	SGK3	Cluster0004
15026446	XM_001503572 // SGMS2 // sphingomyelin synthase 2 // --- // 100072971 /// ENSECAT000000	SGMS2	Cluster0004
15097830	XM_001488536 // SHMT2 // serine hydroxymethyltransferase 2 (mitochondrial) // --- // 10	SHMT2	Cluster0004
15104403	ENSECAT00000020171 // SIK2 // salt-inducible kinase 2 // --- // 100070391 /// XM_001500	SIK2	Cluster0004
15087540	NM_001081791 // SLC16A1 // solute carrier family 16, member 1 (monocarboxylic acid tran	SLC16A1	Cluster0004

14964649	XM_001916839 // SLC16A5 // solute carrier family 16, member 5 (monocarboxylic acid tran	SLC16A5	Cluster0004
15082896	ENSECAT00000013045 // SLC35B4 // solute carrier family 35, member B4 // --- // 10006518	SLC35B4	Cluster0004
15033250	ENSECAT00000015094 // SLC38A9 // solute carrier family 38, member 9 // --- // 100051821	SLC38A9	Cluster0004
15053950	NM_001247992 // SLC5A3 // solute carrier family 5 (sodium/myo-inositol cotransporter),	SLC5A3	Cluster0004
15093971	ENSECAT00000008039 // SMARCAL1 // SWI/SNF related, matrix associated, actin dependent r	SMARCAL1	Cluster0004
14976639	XM_003362748 // SMG1 // smg-1 homolog, phosphatidylinositol 3-kinase-related kinase (C.	SMG1	Cluster0004
15090080	ENSECAT00000023764 // SMYD2 // SET and MYND domain containing 2 // --- // 100052774 ///	SMYD2	Cluster0004
15064965	XM_001497135 // SNTB2 // syntrophin, beta 2 (dystrophin-associated protein A1, 59kDa, b	SNTB2	Cluster0004
14946364	EF397509 // SNX1 // sorting nexin 1 // --- // 100066873	SNX1	Cluster0004
14956385	ENSECAT00000013411 // SNX14 // sorting nexin 14 // --- // 100065533	SNX14	Cluster0004
15012635	XM_001916714 // SNX4 // sorting nexin 4 // --- // 100060493 /// ENSECAT00000009246 // S	SNX4	Cluster0004
15094491	ENSECAT00000023767 // SP140 // SP140 nuclear body protein // --- // ---	SP140	Cluster0004
15096870	ENSECAT00000005952 // SPATS2 // spermatogenesis associated, serine-rich 2 // --- // 100	SPATS2	Cluster0004
14975916	ENSECAT00000009012 // SPN // sialophorin // --- // ---	SPN	Cluster0004
15047557	XM_001491996 // SPTLC2 // serine palmitoyltransferase, long chain base subunit 2 // ---	SPTLC2	Cluster0004
14948614	ENSECAT00000004529 // SPTSSA // serine palmitoyltransferase, small subunit A // --- //	SPTSSA	Cluster0004
15033102	ENSECAT00000010936 // SREK1IP1 // SREK1-interacting protein 1 // --- // ---	SREK1IP1	Cluster0004
15032832	ENSECAT00000018957 // SSBP4 // single stranded DNA binding protein 4 // --- // ---	SSBP4	Cluster0004
15007594	ENSECAT00000013076 // SSFA2 // sperm specific antigen 2 // --- // 100053992	SSFA2	Cluster0004
14965709	XM_001494738 // STAT5A // signal transducer and activator of transcription 5A // --- //	STAT5A	Cluster0004
15031361	XM_001494965 // STK38 // serine/threonine kinase 38 // --- // 100063891 /// ENSECAT0000	STK38	Cluster0004
14966801	ENSECAT00000009030 // SYNRG // synergin, gamma // --- // --- /// ENSECAT00000009061 //	SYNRG	Cluster0004
15124385	ENSECAT00000026272 // TAF2 // TAF2 RNA polymerase II, TATA box binding protein (TBP)-as	TAF2	Cluster0004
14965120	XM_001501300 // TANC2 // tetratricopeptide repeat, ankyrin repeat and coiled-coil conta	TANC2	Cluster0004
14975978	ENSECAT00000022913 // TAOK2 // TAO kinase 2 // --- // ---	TAOK2	Cluster0004
15035902	XM_001498175 // TARS // threonyl-tRNA synthetase // --- // 100068323 /// ENSECAT0000002	TARS	Cluster0004
14999036	ENSECAT00000011011 // TATDN2 // TatD DNase domain containing 2 // --- // 100051750 ///	TATDN2	Cluster0004
14997747	XM_001495752 // TBC1D5 // TBC1 domain family, member 5 // --- // 100052179 /// XM_00336	TBC1D5	Cluster0004
15067444	ENSECAT00000026467 // TEC // tec protein tyrosine kinase // --- // 100061381 /// XM_001	TEC	Cluster0004
15064780	ENSECAT00000006382 // THAP11 // THAP domain containing 11 // --- // ---	THAP11	Cluster0004
15082309	ENSECAT00000012449 // THAP5 // THAP domain containing 5 // --- // 100070659 /// XM_0015	THAP5	Cluster0004
14958261	AJ010315 // TIMP2 // TIMP metallopeptidase inhibitor 2 // --- // 100034134 /// EF077283	TIMP2	Cluster0004
14965173	XM_001917101 // TLK2 // tousled-like kinase 2 // --- // 100054465 /// ENSECAT0000002319	TLK2	Cluster0004
15082379	ENSECAT00000022495 // TMEM168 // transmembrane protein 168 // --- // 100055763 /// XM_0	TMEM168	Cluster0004
15098084	XM_001916898 // TMEM5 // transmembrane protein 5 // --- // 100056566 /// ENSECAT0000001	TMEM5	Cluster0004
14947096	XM_001918264 // TMEM62 // transmembrane protein 62 // --- // 100056447 /// ENSECAT00000	TMEM62	Cluster0004
15073140	XM_001490778 // TMEM63A // transmembrane protein 63A // --- // 100057436 /// ENSECAT000	TMEM63A	Cluster0004
15120610	ENSECAT00000026742 // TMX3 // thioredoxin-related transmembrane protein 3 // --- // 100	TMX3	Cluster0004
15046362	ENSECAT00000012132 // TNFAIP2 // tumor necrosis factor, alpha-induced protein 2 // ---	TNFAIP2	Cluster0004
15120895	ENSECAT00000014729 // TPD52 // tumor protein D52 // --- // ---	TPD52	Cluster0004
15119860	XM_001495283 // TRAPPC8 // trafficking protein particle complex 8 // --- // 100052273 /	TRAPPC8	Cluster0004
15124832	ENSECAT00000025994 // TRAPPC9 // trafficking protein particle complex 9 // --- // 10006	TRAPPC9	Cluster0004
15089756	NM_001256902 // TRIM59 // tripartite motif containing 59 // --- // 100049895 /// ENSECA	TRIM59	Cluster0004
14942004	ENSECAT00000017754 // TRIM8 // tripartite motif containing 8 // --- // 100147211 /// XM	TRIM8	Cluster0004
14967789	ENSECAT00000009800 // TSR1 // TSR1, 20S rRNA accumulation, homolog (S. cerevisiae) // -	TSR1	Cluster0004
14993616	XM_001501379 // TTC27 // tetratricopeptide repeat domain 27 // --- // 100054467 /// ENS	TTC27	Cluster0004
15059641	ENSECAT00000014407 // TTC38 // tetratricopeptide repeat domain 38 // --- // 100053315 /	TTC38	Cluster0004
14978898	XM_001493787 // TYW1 // tRNA-yW synthesizing protein 1 homolog (S. cerevisiae) // --- /	TYW1	Cluster0004
14949289	XM_001490648 // UBA2 // ubiquitin-like modifier activating enzyme 2 // --- // 100057234	UBA2	Cluster0004
14945349	ENSECAT00000006122 // UBE3A // ubiquitin protein ligase E3A // --- // 100050725 /// XM_	UBE3A	Cluster0004
14979634	ENSECAT00000013555 // UBFD1 // ubiquitin family domain containing 1 // --- // ---	UBFD1	Cluster0004
15090433	XM_001491629 // UHMK1 // U2AF homology motif (UHM) kinase 1 // --- // 100058796 /// ENS	UHMK1	Cluster0004
15014658	XM_001500039 // UMPS // uridine monophosphate synthetase // --- // 100070386 /// ENSECA	UMPS	Cluster0004
15032879	ENSECAT00000011373 // UPF1 // UPF1 regulator of nonsense transcripts homolog (yeast) //	UPF1	Cluster0004

15093724	ENSECAT00000011612 // USP1 // ubiquitin specific peptidase 1 // --- // 100070397 /// XM	USP1	Cluster0004
14974683	XM_001493859 // USP42 // ubiquitin specific peptidase 42 // --- // 100062198 /// ENSECA	USP42	Cluster0004
15030728	ENSECAT00000016174 // VARS // valyl-tRNA synthetase // --- // 100058924 /// XM_00149172	VARS	Cluster0004
14959958	ENSECAT00000017794 // VAT1 // vesicle amine transport protein 1 homolog (T. californica	VAT1	Cluster0004
15121164	XM_001494625 // VCPIP1 // valosin containing protein (p97)/p47 complex interacting prot	VCPIP1	Cluster0004
15047534	ENSECAT00000021132 // VIPAR // VPS33B interacting protein, apical-basolateral polarity	VIPAR	Cluster0004
15042971	ENSECAT00000022743 // VPS13A // vacuolar protein sorting 13 homolog A (S. cerevisiae) /	VPS13A	Cluster0004
15068545	XM_001490225 // VPS35 // vacuolar protein sorting 35 homolog (S. cerevisiae) // --- //	VPS35	Cluster0004
15004923	XM_001914963 // VPS36 // vacuolar protein sorting 36 homolog (S. cerevisiae) // --- //	VPS36	Cluster0004
15091719	XM_001488893 // VPS45 // vacuolar protein sorting 45 homolog (S. cerevisiae) // --- //	VPS45	Cluster0004
15103852	ENSECAT00000011283 // XAB2 // XPA binding protein 2 // --- // 100060674 /// XM_00191693	XAB2	Cluster0004
15059264	XM_001502287 // XPNPEP3 // X-prolyl aminopeptidase (aminopeptidase P) 3, putative // --	XPNPEP3	Cluster0004
15023481	ENSECAT00000017310 // ZBTB40 // zinc finger and BTB domain containing 40 // --- // 1000	ZBTB40	Cluster0004
15074567	XM_001492621 // ZBTB41 // zinc finger and BTB domain containing 41 // --- // 100051497	ZBTB41	Cluster0004
15007687	ENSECAT00000018402 // ZC3H15 // zinc finger CCCH-type containing 15 // --- // ---	ZC3H15	Cluster0004
15118767	XM_001496946 // ZCCHC8 // zinc finger, CCHC domain containing 8 // --- // 100058826 ///	ZCCHC8	Cluster0004
15057871	ENSECAT00000007163 // ZDHHC17 // zinc finger, DHHC-type containing 17 // --- // 1000592	ZDHHC17	Cluster0004
14942345	XM_001501299 // ZFYVE27 // zinc finger, FYVE domain containing 27 // --- // 100060913 /	ZFYVE27	Cluster0004
15022277	ENSECAT00000008084 // ZMPSTE24 // zinc metallopeptidase STE24 homolog (S. cerevisiae) /	ZMPSTE24	Cluster0004
15078364	XM_001500504 // ZNF277 // zinc finger protein 277 // --- // 100070827 /// ENSECAT000000	ZNF277	Cluster0004
14955997	ENSECAT00000002231 // ZNF329 // zinc finger protein 329 // --- // ---	ZNF329	Cluster0004
14984375	XM_001497818 // ZNF354B // zinc finger protein 354B // --- // 100067884 /// ENSECAT0000	ZNF354B	Cluster0004
15116973	XM_001915458 // ZNF407 // zinc finger protein 407 // --- // 100052274 /// ENSECAT000000	ZNF407	Cluster0004
15088261	XM_001493570 // ZNF644 // zinc finger protein 644 // --- // 100051777 /// ENSECAT000000	ZNF644	Cluster0004
14957237	ENSECAT00000014086 // ZUFSP // zinc finger with UFM1-specific peptidase domain // --- /	ZUFSP	Cluster0004
15065320	ENSECAT00000009335 // AARS // alanyl-tRNA synthetase // --- // 100054983 /// XM_0015010	AARS	Cluster0005
15132209	ENSECAT00000001168 // ABCB7 // ATP-binding cassette, sub-family B (MDR/TAP), member 7 /	ABCB7	Cluster0005
15062108	XM_001494640 // ABI1 // abl-interactor 1 // --- // 100055377 /// ENSECAT00000016630 //	ABI1	Cluster0005
15105599	ENSECAT00000011541 // ACAD8 // acyl-CoA dehydrogenase family, member 8 // --- // 100072	ACAD8	Cluster0005
14962768	XM_001504761 // ACADVL // acyl-CoA dehydrogenase, very long chain // --- // 100061583 /	ACADVL	Cluster0005
14941680	XM_001915998 // ACSL5 // acyl-CoA synthetase long-chain family member 5 // --- // 10005	ACSL5	Cluster0005
14977169	ENSECAT00000026215 // ADCY9 // adenylate cyclase 9 // --- // 100066046 /// XM_001502248	ADCY9	Cluster0005
14992838	ENSECAT00000012840 // AFTPH // aftiphilin // --- // 100063526	AFTPH	Cluster0005
14987308	XM_001503946 // AGGF1 // angiogenic factor with G patch and FHA domains 1 // --- // 100	AGGF1	Cluster0005
15007474	ENSECAT00000009408 // AGPS // alkylglycerone phosphate synthase // --- // ---	AGPS	Cluster0005
15133293	XM_001915156 // AIFM1 // apoptosis-inducing factor, mitochondrion-associated, 1 // ---	AIFM1	Cluster0005
15004936	XM_001487974 // ALG11 // asparagine-linked glycosylation 11, alpha-1,2-mannosyltransfer	ALG11	Cluster0005
15093686	XM_001499799 // ALG6 // asparagine-linked glycosylation 6, alpha-1,3-glucosyltransferas	ALG6	Cluster0005
15117972	XM_001496710 // ANKRD13A // ankyrin repeat domain 13A // --- // 100066442 /// ENSECAT00	ANKRD13A	Cluster0005
14943582	XM_001503944 // AP3M1 // adaptor-related protein complex 3, mu 1 subunit // --- // 1000	AP3M1	Cluster0005
14961552	XM_001918294 // APPBP2 // amyloid beta precursor protein (cytoplasmic tail) binding pro	APPBP2	Cluster0005
14999688	XM_001491548 // APPL1 // adaptor protein, phosphotyrosine interaction, PH domain and le	APPL1	Cluster0005
15121109	XM_001494559 // ARFGEF1 // ADP-ribosylation factor guanine nucleotide-exchange factor 1	ARFGEF1	Cluster0005
15090490	ENSECAT00000012392 // ARHGAP30 // Rho GTPase activating protein 30 // --- // 100066336	ARHGAP30	Cluster0005
14940456	ENSECAT00000018331 // ARHGAP5 // Rho GTPase activating protein 5 // --- // 100056199 //	ARHGAP5	Cluster0005
15000469	XM_001498598 // ARIH2 // ariadne homolog 2 (Drosophila) // --- // 100053691 /// ENSECAT	ARIH2	Cluster0005
14977858	XM_001494690 // ARPC1B // actin related protein 2/3 complex, subunit 1B, 41kDa // --- /	ARPC1B	Cluster0005
14936384	XM_001489692 // ASB7 // ankyrin repeat and SOCS box containing 7 // --- // 100050201 //	ASB7	Cluster0005
15121251	XM_001496350 // ASPH // aspartate beta-hydroxylase // --- // 100065909 /// ENSECAT00000	ASPH	Cluster0005
15048112	XM_001489195 // ATG2B // autophagy related 2B // --- // 100054652 /// ENSECAT0000000401	ATG2B	Cluster0005
15084195	EU423855 // ATP1B1 // ATPase, Na+/K+ transporting, beta 1 polypeptide // --- // 1000339	ATP1B1	Cluster0005
14965688	XM_003362552 // ATP6V0A1 // ATPase, H+ transporting, lysosomal V0 subunit a1 // --- //	ATP6V0A1	Cluster0005
15115080	XM_001915444 // ATP6V0A2 // ATPase, H+ transporting, lysosomal V0 subunit a2 // --- //	ATP6V0A2	Cluster0005
15102796	ENSECAT00000019940 // BAZ2A // bromodomain adjacent to zinc finger domain, 2A // --- //	BAZ2A	Cluster0005

15099566	ENSECAT00000010760 // BCL2L13 // BCL2-like 13 (apoptosis facilitator) // --- // 1000550	BCL2L13	Cluster0005
14967579	XM_001502104 // BLMH // bleomycin hydrolase // --- // 100072223 /// ENSECAT00000020692	BLMH	Cluster0005
15053222	ENSECAT00000023192 // BRD3 // bromodomain containing 3 // --- // 100066434 /// XM_00191	BRD3	Cluster0005
15034349	ENSECAT00000013879 // BRD9 // bromodomain containing 9 // --- // 100057849 /// XM_00149	BRD9	Cluster0005
14935846	ENSECAT00000018146 // BTBD1 // BTB (POZ) domain containing 1 // --- // 100068143 /// XM	BTBD1	Cluster0005
15106996	ENSECAT00000013130 // C2CD3 // C2 calcium-dependent domain containing 3 // --- // 10006	C2CD3	Cluster0005
15084070	XM_001492532 // C4BPA // complement component 4 binding protein, alpha // --- // 100056	C4BPA	Cluster0005
15102342	ENSECAT00000025888 // CALCOCO1 // calcium binding and coiled-coil domain 1 // --- // 10	CALCOCO1	Cluster0005
15050898	ENSECAT00000018487 // CAMSAP1 // calmodulin regulated spectrin-associated protein 1 //	CAMSAP1	Cluster0005
15098217	XM_001491339 // CAND1 // cullin-associated and neddylation-dissociated 1 // --- // 1000	CAND1	Cluster0005
15022297	XM_001503360 // CAP1 // CAP, adenylate cyclase-associated protein 1 (yeast) // --- // 1	CAP1	Cluster0005
14969372	ENSECAT00000008062 // CAPRIN1 // cell cycle associated protein 1 // --- // 100060513 //	CAPRIN1	Cluster0005
15088313	XM_001495159 // CCBL2 // cysteine conjugate-beta lyase 2 // --- // 100052141 /// ENSECA	CCBL2	Cluster0005
15077498	ENSECAT00000024807 // CCDC132 // coiled-coil domain containing 132 // --- // 100051289	CCDC132	Cluster0005
14959412	ENSECAT00000015048 // CCDC47 // coiled-coil domain containing 47 // --- // 100054134 //	CCDC47	Cluster0005
14989582	ENSECAT00000007818 // CCDC88A // coiled-coil domain containing 88A // --- // ---	CCDC88A	Cluster0005
14989361	ENSECAT00000018270 // CCT4 // chaperonin containing TCP1, subunit 4 (delta) // --- // 1	CCT4	Cluster0005
15073562	ENSECAT00000016219 // CDC73 // cell division cycle 73, Paf1/RNA polymerase II complex c	CDC73	Cluster0005
15076823	ENSECAT00000018342 // CDK13 // cyclin-dependent kinase 13 // --- // ---	CDK13	Cluster0005
15002624	XM_001490931 // CDK8 // cyclin-dependent kinase 8 // --- // 100050819 /// ENSECAT000000	CDK8	Cluster0005
15018974	XM_001497360 // CEP104 // centrosomal protein 104kDa // --- // 100052540 /// ENSECAT000	CEP104	Cluster0005
14964972	ENSECAT00000022480 // CEP95 // centrosomal protein 95kDa // --- // 100064141 /// XM_001	CEP95	Cluster0005
15019576	ENSECAT00000026613 // CHMP7 // charged multivesicular body protein 7 // --- // 10005392	CHMP7	Cluster0005
14933451	ENSECAT00000007996 // CHUK // conserved helix-loop-helix ubiquitous kinase // --- // 10	CHUK	Cluster0005
14972300	XM_001915245 // CKAP5 // cytoskeleton associated protein 5 // --- // 100050886 /// ENSE	CKAP5	Cluster0005
14937462	XM_001498202 // CLPX // ClpX caseinolytic peptidase X homolog (E. coli) // --- // 10005	CLPX	Cluster0005
15066675	XM_001491801 // CNOT6L // CCR4-NOT transcription complex, subunit 6-like // --- // 1000	CNOT6L	Cluster0005
14933520	ENSECAT00000014816 // COX15 // COX15 homolog, cytochrome c oxidase assembly protein (ye	COX15	Cluster0005
14943835	XM_001490352 // CSGALNACT2 // chondroitin sulfate N-acetylgalactosaminyltransferase 2 /	CSGALNACT2	Cluster0005
15037096	ENSECAT00000007999 // CSNK2A1 // casein kinase 2, alpha 1 polypeptide // --- // ---	CSNK2A1	Cluster0005
15051765	XM_001491865 // CTNNAL1 // catenin (cadherin-associated protein), alpha-like 1 // --- /	CTNNAL1	Cluster0005
15000906	ENSECAT00000007477 // CTNNB1 // catenin (cadherin-associated protein), beta 1, 88kDa //	CTNNB1	Cluster0005
14933436	XM_001500438 // CWF19L1 // CWF19-like 1, cell cycle control (S. pombe) // --- // 100060	CWF19L1	Cluster0005
15062469	XM_001498475 // DCLRE1C // DNA cross-link repair 1C // --- // 100056337 /// ENSECAT0000	DCLRE1C	Cluster0005
15092219	ENSECAT00000014445 // DDX20 // DEAD (Asp-Glu-Ala-Asp) box polypeptide 20 // --- // 1000	DDX20	Cluster0005
14934438	ENSECAT00000011504 // DDX21 // DEAD (Asp-Glu-Ala-Asp) box helicase 21 // --- // 1000726	DDX21	Cluster0005
14985975	ENSECAT00000019747 // DDX46 // DEAD (Asp-Glu-Ala-Asp) box polypeptide 46 // --- // 1000	DDX46	Cluster0005
14937368	XM_001497645 // DENND4A // DENN/MADD domain containing 4A // --- // 100053090 /// XM_00	DENND4A	Cluster0005
15086553	XM_001493858 // DENND4B // DENN/MADD domain containing 4B // --- // 100062197 /// ENSEC	DENND4B	Cluster0005
14982567	ENSECAT00000023229 // DIAPH1 // diaphanous homolog 1 (Drosophila) // --- // 100072186 /	DIAPH1	Cluster0005
15005384	XM_001495217 // DIS3 // DIS3 mitotic control homolog (S. cerevisiae) // --- // 10005193	DIS3	Cluster0005
15076761	XM_001491576 // DLD // dihydrolipoamide dehydrogenase // --- // 100050920 /// ENSECAT00	DLD	Cluster0005
14986452	XM_003362834 // DMXL1 // Dmx-like 1 // --- // 100064119 /// XM_001504549 // DMXL1 // Dm	DMXL1	Cluster0005
14943377	XM_001503793 // DNAJB12 // DnaJ (Hsp40) homolog, subfamily B, member 12 // --- // 10006	DNAJB12	Cluster0005
14937408	XM_003363658 // DPP8 // dipeptidyl-peptidase 8 // --- // 100053245 /// XM_001497856 //	DPP8	Cluster0005
15121755	XM_001491535 // DPY19L4 // dpy-19-like 4 (C. elegans) // --- // 100058629 /// ENSECAT00	DPY19L4	Cluster0005
15089917	ENSECAT00000019777 // EDEM3 // ER degradation enhancer, mannosidase alpha-like 3 // ---	EDEM3	Cluster0005
15113623	XM_001496800 // EIF4ENIF1 // eukaryotic translation initiation factor 4E nuclear import	EIF4ENIF1	Cluster0005
15089102	ENSECAT00000006690 // ELK4 // ELK4, ETS-domain protein (SRF accessory protein 1) // ---	ELK4	Cluster0005
15040597	XM_001503431 // ELMO2 // engulfment and cell motility 2 // --- // 100056774 /// ENSECAT	ELMO2	Cluster0005
15035126	ENSECAT00000005942 // ERBB2IP // erbb2 interacting protein // --- // 100057128	ERBB2IP	Cluster0005
15025388	XM_001500342 // ETFDH // electron-transferring-flavoprotein dehydrogenase // --- // 100	ETFDH	Cluster0005
15096308	ENSECAT00000007430 // ETNK1 // ethanolamine kinase 1 // --- // 100068424 /// XM_0019162	ETNK1	Cluster0005
15083433	XM_001504629 // EZH2 // enhancer of zeste homolog 2 (Drosophila) // --- // 100051290 //	EZH2	Cluster0005

15036000	ENSECAT00000018036 // FAM105A // family with sequence similarity 105, member A // --- /	FAM105A	Cluster0005
15133485	ENSECAT00000020006 // FAM122B // family with sequence similarity 122B // --- // ---	FAM122B	Cluster0005
15036973	NM_001256937 // FASTKD5 // FAST kinase domains 5 // --- // 100052656 /// ENSECAT0000000	FASTKD5	Cluster0005
15005446	XM_001488408 // FBXL3 // F-box and leucine-rich repeat protein 3 // --- // 100052811 //	FBXL3	Cluster0005
14989752	XM_001498271 // FBXO11 // F-box protein 11 // --- // 100053097 /// ENSECAT00000010973 /	FBXO11	Cluster0005
14985373	XM_001503809 // FBXO38 // F-box protein 38 // --- // 100060728 /// ENSECAT00000017183 /	FBXO38	Cluster0005
15029183	NM_001160296 // FBXO9 // F-box protein 9 // --- // 100069351 /// ENSECAT00000007724 //	FBXO9	Cluster0005
14946129	XM_001496225 // FEM1B // fem-1 homolog b (C. elegans) // --- // 100052688 /// ENSECAT00	FEM1B	Cluster0005
14990202	ENSECAT00000010650 // FEZ2 // fasciculation and elongation protein zeta 2 (zygin II) //	FEZ2	Cluster0005
15072966	XM_001492655 // FH // fumarate hydratase // --- // 100060381 /// ENSECAT00000022289 //	FH	Cluster0005
15071572	ENSECAT00000020783 // FIP1L1 // FIP1 like 1 (S. cerevisiae) // --- // 100060154 /// XM_	FIP1L1	Cluster0005
15090126	ENSECAT00000019051 // FLVCR1 // feline leukemia virus subgroup C cellular receptor 1 //	FLVCR1	Cluster0005
15100225	ENSECAT00000021556 // FOXJ2 // forkhead box J2 // --- // 100053321 /// XM_001498727 //	FOXJ2	Cluster0005
15016599	XM_001503156 // FOXJ3 // forkhead box J3 // --- // 100053693 /// ENSECAT00000009015 //	FOXJ3	Cluster0005
14993184	XM_001498254 // FOXN2 // forkhead box N2 // --- // 100053059 /// ENSECAT00000008546 //	FOXN2	Cluster0005
15075619	XM_001497220 // FUCA2 // fucosidase, alpha-L- 2, plasma // --- // 100067102 /// ENSECAT	FUCA2	Cluster0005
15011788	XM_001495885 // FXR1 // fragile X mental retardation, autosomal homolog 1 // --- // 100	FXR1	Cluster0005
15053773	XM_001915095 // GABPA // GA binding protein transcription factor, alpha subunit 60kDa /	GABPA	Cluster0005
15079991	ENSECAT00000022085 // GALNT11 // UDP-N-acetyl-alpha-D-galactosamine:polypeptide N-acety	GALNT11	Cluster0005
14942063	XM_001499117 // GBF1 // golgi brefeldin A resistant guanine nucleotide exchange factor	GBF1	Cluster0005
15132659	ENSECAT00000019883 // GLA // galactosidase, alpha // --- // 100060370 /// XM_001492649	GLA	Cluster0005
14944180	ENSECAT00000007086 // GNPAT // glyceronephosphate O-acyltransferase // --- // 100060758	GNPAT	Cluster0005
14949265	XM_001490607 // GPI // glucose-6-phosphate isomerase // --- // 100057160 /// ENSECAT000	GPI	Cluster0005
15077957	ENSECAT00000001905 // GPNMB // glycoprotein (transmembrane) nmb // --- // 100067870 ///	GPNMB	Cluster0005
15066947	XM_001489101 // GRSF1 // G-rich RNA sequence binding factor 1 // --- // 100054436 /// E	GRSF1	Cluster0005
14964932	XM_001499857 // HELZ // helicase with zinc finger // --- // 100053580 /// ENSECAT000000	HELZ	Cluster0005
15070532	ENSECAT00000012452 // HERC3 // HECT and RLD domain containing E3 ubiquitin protein liga	HERC3	Cluster0005
15002184	ENSECAT00000026209 // HLTF // helicase-like transcription factor // --- // 100058775 //	HLTF	Cluster0005
15004670	NM_001081835 // HMGB1 // high mobility group box 1 // --- // 100033873 /// AB275457 //	HMGB1	Cluster0005
15017855	XM_001501425 // HNRNPR // heterogeneous nuclear ribonucleoprotein R // --- // 100071619	HNRNPR	Cluster0005
15056743	XM_001488824 // HOOK3 // hook homolog 3 (Drosophila) // --- // 100053799 /// ENSECAT000	HOOK3	Cluster0005
15048217	NM_001163955 // HSP90AA1 // heat shock protein 90kDa alpha (cytosolic), class A member	HSP90AA1	Cluster0005
14986056	XM_001918224 // HSPA4 // heat shock 70kDa protein 4 // --- // 100072809 /// ENSECAT0000	HSPA4	Cluster0005
14982743	NM_001163884 // HSPA9 // heat shock 70kDa protein 9 (mortalin) // --- // 100034032 ///	HSPA9	Cluster0005
15073290	XM_001488053 // IARS2 // isoleucyl-tRNA synthetase 2, mitochondrial // --- // 100049915	IARS2	Cluster0005
15011383	ENSECAT00000012896 // IDH1 // isocitrate dehydrogenase 1 (NADP+), soluble // --- // 100	IDH1	Cluster0005
15053877	XM_001498235 // IFNAR2 // interferon (alpha, beta and omega) receptor 2 // --- // 10005	IFNAR2	Cluster0005
15107665	XM_001917959 // IPO7 // importin 7 // --- // 100055677 /// ENSECAT00000017394 // IPO7 /	IPO7	Cluster0005
15056454	ENSECAT00000007884 // IRF2 // interferon regulatory factor 2 // --- // 100050990 /// XM	IRF2	Cluster0005
15037475	XM_001916721 // ITCH // itchy E3 ubiquitin protein ligase // --- // 100069340 /// ENSEC	ITCH	Cluster0005
15001521	XM_001493724 // KAT2B // K(lysine) acetyltransferase 2B // --- // 100061976 /// ENSECAT	KAT2B	Cluster0005
14971361	XM_001497095 // KDM2A // lysine (K)-specific demethylase 2A // --- // 100052955 /// ENS	KDM2A	Cluster0005
15021948	XM_001498191 // KDM4A // lysine (K)-specific demethylase 4A // --- // 100053154 /// ENS	KDM4A	Cluster0005
15096432	XM_001916399 // KLHDC5 // kelch domain containing 5 // --- // 100069196 /// ENSECAT0000	KLHDC5	Cluster0005
15011849	ENSECAT00000022513 // KLHL24 // kelch-like 24 (Drosophila) // --- // 100058815 /// XM_0	KLHL24	Cluster0005
14993161	ENSECAT00000022186 // KLRAQ1 // protein phosphatase 1, regulatory subunit 21 // --- //	KLRAQ1	Cluster0005
15098365	ENSECAT00000009629 // LANCL1 // LanC lantibiotic synthetase component C-like 1 (bacteri	LANCL1	Cluster0005
15077192	ENSECAT00000024482 // LANCL2 // LanC lantibiotic synthetase component C-like 2 (bacteri	LANCL2	Cluster0005
14938147	XM_001501499 // LEO1 // Leo1, Paf1/RNA polymerase II complex component, homolog (S. cer	LEO1	Cluster0005
15032341	XM_001503388 // LMBRD1 // LMBR1 domain containing 1 // --- // 100057167 /// ENSECAT0000	LMBRD1	Cluster0005
15033756	ENSECAT00000021269 // LMBRD2 // LMBR1 domain containing 2 // --- // 100067733 /// XM_00	LMBRD2	Cluster0005
14977954	ENSECAT00000020961 // LMTK2 // lemur tyrosine kinase 2 // --- // 100629931	LMTK2	Cluster0005
15111401	ENSECAT00000015919 // LOC100049927 // ester hydrolase C11orf54-like // --- // 100049927	LOC100049927	Cluster0005
15048157	ENSECAT00000001245 // LOC100049970 // histone-lysine N-methyltransferase setd3-like //	LOC100049970	Cluster0005

14935068	XM_003363640 // LOC100050002 // heterogeneous nuclear ribonucleoprotein F-like // --- /	LOC100050002	Cluster0005
14936362	XM_001488965 // LOC100050003 // leucine-rich repeat-containing protein 28-like // --- /	LOC100050003	Cluster0005
15089720	ENSECAT00000015126 // LOC100050024 // latexin-like // --- // 100050024 /// XM_001489649	LOC100050024	Cluster0005
14969557	ENSECAT00000010831 // LOC100050075 // estradiol 17-beta-dehydrogenase 12-like // --- //	LOC100050075	Cluster0005
15066901	XM_001488484 // LOC100050172 // mitochondrial inner membrane protein COX18-like // ---	LOC100050172	Cluster0005
14994794	XM_001488818 // LOC100050214 // pre-mRNA-splicing factor ISY1 homolog // --- // 1000502	LOC100050214	Cluster0005
15009234	ENSECAT00000025356 // LOC100050291 // ADP-ribosylation factor-like protein 5A-like // -	LOC100050291	Cluster0005
15118233	XM_001489039 // LOC100050323 // gatC-like protein-like // --- // 100050323 /// ENSECAT0	LOC100050323	Cluster0005
15003753	XM_001489318 // LOC100050355 // ubiquitin carboxyl-terminal hydrolase isozyme L3-like /	LOC100050355	Cluster0005
15033044	ENSECAT00000003354 // LOC100050363 // small glutamine-rich tetratricopeptide repeat-con	LOC100050363	Cluster0005
15038557	XM_001490026 // LOC100050441 // mRNA export factor-like // --- // 100050441 /// ENSECAT	LOC100050441	Cluster0005
14976768	XM_001489539 // LOC100050501 // limkain-b1-like // --- // 100050501 /// ENSECAT00000014	LOC100050501	Cluster0005
15076678	ENSECAT00000021120 // LOC100050532 // phosphatidylinositol-4,5-bisphosphate 3-kinase ca	LOC100050532	Cluster0005
14992694	XM_001490819 // LOC100050657 // annexin A4-like // --- // 100050657 /// ENSECAT00000013	LOC100050657	Cluster0005
14988058	XM_001490447 // LOC100050737 // MIT domain-containing protein 1-like // --- // 10005073	LOC100050737	Cluster0005
15076724	ENSECAT00000018378 // LOC100050769 // b-cell receptor-associated protein 29-like // ---	LOC100050769	Cluster0005
15116739	ENSECAT00000008044 // LOC100050782 // lisH domain and HEAT repeat-containing protein KI	LOC100050782	Cluster0005
15021547	XM_001490954 // LOC100050901 // transcription factor BTF3 homolog 4-like // --- // 1000	LOC100050901	Cluster0005
15118305	ENSECAT00000026473 // LOC100051008 // phosphatidylethanolamine-binding protein 1-like /	LOC100051008	Cluster0005
15005169	ENSECAT00000009370 // LOC100051117 // uncharacterized protein KIAA1704-like // --- // 1	LOC100051117	Cluster0005
15029718	XM_001490955 // LOC100051123 // adenosine 3 -phospho 5 -phosphosulfate transporter 2-li	LOC100051123	Cluster0005
15058105	ENSECAT00000020243 // LOC100051280 // uncharacterized protein C12orf29 homolog // --- /	LOC100051280	Cluster0005
15038818	XM_001493585 // LOC100051346 // protein C20orf11 homolog // --- // 100051346 /// ENSECA	LOC100051346	Cluster0005
15044550	XM_001497650 // LOC100051348 // protein phosphatase 1A-like // --- // 100051348 /// XM_	LOC100051348	Cluster0005
14935485	ENSECAT00000023662 // LOC100051461 // poly(ADP-ribose) glycohydrolase-like // --- // 10	LOC100051461	Cluster0005
15102585	ENSECAT00000009591 // LOC100051590 // dnaJ homolog subfamily C member 14-like // --- //	LOC100051590	Cluster0005
15127207	XM_001492157 // LOC100051597 // pyruvate dehydrogenase E1 component subunit alpha, soma	LOC100051597	Cluster0005
15084166	ENSECAT00000023021 // LOC100051645 // TIP41-like protein-like // --- // 100051645 /// X	LOC100051645	Cluster0005
15084893	ENSECAT00000000944 // LOC100051658 // n-acetylneuraminate lyase-like // --- // 10005165	LOC100051658	Cluster0005
15061511	ENSECAT00000024700 // LOC100051710 // uncharacterized protein KIAA0930-like // --- // 1	LOC100051710	Cluster0005
14941009	ENSECAT00000026311 // LOC100051737 // serine/threonine-protein phosphatase 2A 55 kDa re	LOC100051737	Cluster0005
14972487	ENSECAT00000015176 // LOC100051744 // mitochondrial carrier homolog 2-like // --- // 10	LOC100051744	Cluster0005
15075976	ENSECAT00000017774 // LOC100051773 // programmed cell death protein 2-like // --- // 10	LOC100051773	Cluster0005
15100959	ENSECAT00000020767 // LOC100051785 // GTPase KRas-like // --- // 100051785 /// XM_00149	LOC100051785	Cluster0005
15041992	XM_001493301 // LOC100051824 // melanoma antigen recognized by T-cells 1-like // --- //	LOC100051824	Cluster0005
15027396	ENSECAT00000019450 // LOC100051847 // protein phosphatase 1 regulatory subunit 11-like	LOC100051847	Cluster0005
15121697	ENSECAT00000007598 // LOC100051849 // protein FAM92A1-like // --- // 100051849 /// XM_0	LOC100051849	Cluster0005
15116929	ENSECAT00000017749 // LOC100052148 // TIM21-like protein, mitochondrial-like // --- //	LOC100052148	Cluster0005
15089758	ENSECAT00000025442 // LOC100052650 // importin subunit alpha-4-like // --- // 100052650	LOC100052650	Cluster0005
15102850	ENSECAT00000015933 // LOC100052730 // nascent polypeptide-associated complex subunit al	LOC100052730	Cluster0005
14963768	XM_001489880 // LOC100052790 // folliculin-like // --- // 100052790 /// ENSECAT00000022	LOC100052790	Cluster0005
15121321	ENSECAT00000013628 // LOC100052888 // cholesterol 7-alpha-monooxygenase-like // --- //	LOC100052888	Cluster0005
14937289	ENSECAT00000013887 // LOC100052900 // alpha- and gamma-adaptin-binding protein p34-like	LOC100052900	Cluster0005
15093263	XM_001498098 // LOC100053123 // dnaJ homolog subfamily B member 4-like // --- // 100053	LOC100053123	Cluster0005
14937396	ENSECAT00000020284 // LOC100053141 // UPF0464 protein C15orf44 homolog // --- // 100053	LOC100053141	Cluster0005
15044990	ENSECAT00000021762 // LOC100053161 // zinc transporter ZIP9-like // --- // 100053161 //	LOC100053161	Cluster0005
15047937	XM_001497437 // LOC100053265 // legumain-like // --- // 100053265 /// ENSECAT0000001104	LOC100053265	Cluster0005
15010072	XM_001500017 // LOC100053400 // protein lunapark-like // --- // 100053400 /// ENSECAT00	LOC100053400	Cluster0005
15121630	ENSECAT00000023730 // LOC100053423 // 2,4-dienoyl-CoA reductase, mitochondrial-like //	LOC100053423	Cluster0005
15081895	XM_001916123 // LOC100053610 // hyccin-like // --- // 100053610 /// ENSECAT00000025313	LOC100053610	Cluster0005
15064836	ENSECAT00000014815 // LOC100053856 // nuclear factor of activated T-cells, cytoplasmic	LOC100053856	Cluster0005
14976793	XM_001488859 // LOC100053887 // pyridoxal-dependent decarboxylase domain-containing pro	LOC100053887	Cluster0005
15037305	XM_001500376 // LOC100053947 // microtubule-associated protein RP/EB family member 1-li	LOC100053947	Cluster0005
14990106	ENSECAT00000020438 // LOC100054231 // CCAAT/enhancer-binding protein zeta-like // --- /	LOC100054231	Cluster0005

15016748	XM_001503462 // LOC100054282 // peptidyl-prolyl cis-trans isomerase E-like // --- // 10	LOC100054282	Cluster0005
15133409	XM_001489008 // LOC100054364 // ras-related protein Rap-2c-like // --- // 100054364 ///	LOC100054364	Cluster0005
15057345	ENSECAT00000021737 // LOC100054433 // acid ceramidase-like // --- // 100054433 /// XM_0	LOC100054433	Cluster0005
15086960	XM_001917235 // LOC100054493 // acidic leucine-rich nuclear phosphoprotein 32 family me	LOC100054493	Cluster0005
15074186	XM_001489714 // LOC100054506 // presenilin-2-like // --- // 100054506 /// ENSECAT000000	LOC100054506	Cluster0005
15130093	ENSECAT00000009431 // LOC100054669 // HIV Tat-specific factor 1 homolog // --- // 10005	LOC100054669	Cluster0005
15115573	XM_001488009 // LOC100054712 // twisted gastrulation protein homolog 1-like // --- // 1	LOC100054712	Cluster0005
15086948	XM_001917162 // LOC100054765 // induced myeloid leukemia cell differentiation protein M	LOC100054765	Cluster0005
14958029	ENSECAT00000020648 // LOC100054966 // NAD-dependent deacetylase sirtuin-7-like // --- /	LOC100054966	Cluster0005
15008657	XM_001489408 // LOC100055077 // translin-like // --- // 100055077 /// ENSECAT0000001680	LOC100055077	Cluster0005
15089112	XM_001489449 // LOC100055118 // nuclear ubiquitous casein and cyclin-dependent kinases	LOC100055118	Cluster0005
15051434	ENSECAT00000005642 // LOC100055153 // exosome complex component RRP40-like // --- // 10	LOC100055153	Cluster0005
14946695	ENSECAT00000023924 // LOC100055314 // AP-4 complex subunit epsilon-1-like // --- // 100	LOC100055314	Cluster0005
15092601	XM_001488337 // LOC100055424 // zinc transporter 7-like // --- // 100055424 /// ENSECAT	LOC100055424	Cluster0005
15003200	XM_001488628 // LOC100055495 // chronic lymphocytic leukemia deletion region gene 6 pro	LOC100055495	Cluster0005
15122978	XM_001914906 // LOC100055514 // copine-3-like // --- // 100055514 /// ENSECAT0000002368	LOC100055514	Cluster0005
14946749	ENSECAT00000012768 // LOC100055565 // DTW domain-containing protein 1-like // --- // 10	LOC100055565	Cluster0005
14997471	XM_001489950 // LOC100056043 // upstream-binding protein 1-like // --- // 100056043 ///	LOC100056043	Cluster0005
15017269	XM_001503862 // LOC100056044 // protein tyrosine phosphatase type IVA 2-like // --- //	LOC100056044	Cluster0005
15043180	ENSECAT00000027014 // LOC100056086 // structural maintenance of chromosomes protein 5-l	LOC100056086	Cluster0005
15038589	ENSECAT00000013401 // LOC100056212 // ras-related protein Rab-22A-like // --- // 100056	LOC100056212	Cluster0005
15027429	ENSECAT00000025949 // LOC100056231 // patr class I histocompatibility antigen, A-126 al	LOC100056231	Cluster0005
15089258	ENSECAT00000009993 // LOC100056344 // transcriptional adapter 1-like // --- // 10005634	LOC100056344	Cluster0005
15068189	XM_001501365 // LOC100056426 // MORF4 family-associated protein 1-like // --- // 100056	LOC100056426	Cluster0005
15074418	ENSECAT00000010619 // LOC100056607 // RRP15-like protein-like // --- // 100056607 /// X	LOC100056607	Cluster0005
15063527	ENSECAT00000008224 // LOC100056689 // optineurin-like // --- // 100056689	LOC100056689	Cluster0005
15091278	XM_001496771 // LOC100056899 // HCLS1-associated protein X-1-like // --- // 100056899 /	LOC100056899	Cluster0005
15120833	ENSECAT00000024056 // LOC100057100 // sorting nexin-16-like // --- // 100057100 /// XM_	LOC100057100	Cluster0005
14941158	XM_001490606 // LOC100057158 // BRISC complex subunit Abro1-like // --- // 100057158 //	LOC100057158	Cluster0005
15090303	ENSECAT00000010143 // LOC100057176 // uncharacterized LOC100057176 // --- // 100057176	LOC100057176	Cluster0005
15115626	ENSECAT00000007438 // LOC100057183 // uncharacterized protein C18orf19 homolog // --- /	LOC100057183	Cluster0005
15108085	ENSECAT00000011744 // LOC100057256 // transmembrane protein 86A-like // --- // 10005725	LOC100057256	Cluster0005
14980277	XM_001492888 // LOC100057523 // phosphomannomutase 2-like // --- // 100057523 /// ENSEC	LOC100057523	Cluster0005
15122420	XM_001497901 // LOC100057539 // protein FAM91A1-like // --- // 100057539 /// ENSECAT000	LOC100057539	Cluster0005
15095452	XM_001490880 // LOC100057576 // tubby-related protein 3-like // --- // 100057576 /// EN	LOC100057576	Cluster0005
15099390	XM_001500579 // LOC100057698 // NADH dehydrogenase [ubiquinone] 1 alpha subcomplex subu	LOC100057698	Cluster0005
15086135	XM_001500077 // LOC100057746 // protein SMG5-like // --- // 100057746 /// ENSECAT000000	LOC100057746	Cluster0005
15129146	ENSECAT00000001525 // LOC100057749 // armadillo repeat-containing X-linked protein 3-li	LOC100057749	Cluster0005
15073111	XM_001491159 // LOC100058044 // Golgi resident protein GCP60-like // --- // 100058044 /	LOC100058044	Cluster0005
15066732	XM_001491232 // LOC100058151 // lysosome membrane protein 2-like // --- // 100058151 //	LOC100058151	Cluster0005
14969844	ENSECAT00000014195 // LOC100058223 // NADH dehydrogenase [ubiquinone] iron-sulfur prote	LOC100058223	Cluster0005
15011717	XM_001495170 // LOC100058230 // mitofusin-1-like // --- // 100058230 /// ENSECAT0000002	LOC100058230	Cluster0005
15090642	ENSECAT00000020352 // LOC100058382 // astrocytic phosphoprotein PEA-15-like // --- // 1	LOC100058382	Cluster0005
15057207	XM_001495978 // LOC100058499 // tankyrase-1-like // --- // 100058499 /// ENSECAT0000000	LOC100058499	Cluster0005
15133068	XM_001914996 // LOC100058559 // regulator of nonsense transcripts 3B-like // --- // 100	LOC100058559	Cluster0005
15056472	XM_001492050 // LOC100058620 // RWD domain-containing protein 4-like // --- // 10005862	LOC100058620	Cluster0005
14932770	ENSECAT00000013781 // LOC100058684 // uncharacterized protein C10orf46-like // --- // 1	LOC100058684	Cluster0005
15133080	XM_001914804 // LOC100058761 // NF-kappa-B-activating protein-like // --- // 100058761	LOC100058761	Cluster0005
15011908	ENSECAT00000020156 // LOC100058922 // translation initiation factor eIF-2B subunit epsi	LOC100058922	Cluster0005
15133038	XM_001914739 // LOC100059026 // UPF0428 protein CXorf56 homolog // --- // 100059026 ///	LOC100059026	Cluster0005
15026608	XM_001489220 // LOC100059043 // serine/threonine-protein kinase PRP4 homolog // --- //	LOC100059043	Cluster0005
14991570	ENSECAT00000012398 // LOC100059155 // UPF0760 protein C2orf29-like // --- // 100059155	LOC100059155	Cluster0005
15098242	ENSECAT00000008498 // LOC100059287 // nuclear pore complex protein Nup107-like // --- /	LOC100059287	Cluster0005
15024898	ENSECAT00000012264 // LOC100059300 // BTB/POZ domain-containing protein KCTD9-like // -	LOC100059300	Cluster0005

15113901	XM_001499699 // LOC100059322 // phosphatidylinositol transfer protein beta isoform-like	LOC100059322	Cluster0005
14995858	XM_001915031 // LOC100059386 // actin-related protein 8-like // --- // 100059386 /// EN	LOC100059386	Cluster0005
15133943	XM_001915515 // LOC100059443 // deoxyribonuclease-1-like 1-like // --- // 100059443 ///	LOC100059443	Cluster0005
15071507	XM_003364683 // LOC100059506 // transmembrane protein 165-like // --- // 100059506 ///	LOC100059506	Cluster0005
14997645	XM_001492115 // LOC100059572 // DNA topoisomerase 2-beta-like // --- // 100059572 /// E	LOC100059572	Cluster0005
15041965	XM_001492144 // LOC100059612 // protein RIC1 homolog // --- // 100059612 /// ENSECAT000	LOC100059612	Cluster0005
15005180	ENSECAT00000019998 // LOC100059776 // UPF0124 protein C13orf31-like // --- // 100059776	LOC100059776	Cluster0005
14984997	ENSECAT00000020573 // LOC100059807 // ubiquitin-like domain-containing CTD phosphatase	LOC100059807	Cluster0005
15091455	ENSECAT00000001614 // LOC100059977 // sorting nexin-27-like // --- // 100059977 /// XM_	LOC100059977	Cluster0005
14982038	XM_001917506 // LOC100059991 // protein FAM114A2-like // --- // 100059991 /// ENSECAT00	LOC100059991	Cluster0005
14936691	ENSECAT00000016292 // LOC100060114 // isocitrate dehydrogenase [NAD] subunit alpha, mit	LOC100060114	Cluster0005
14936702	ENSECAT00000025523 // LOC100060143 // dnaJ homolog subfamily A member 4-like // --- //	LOC100060143	Cluster0005
14972114	XM_001492591 // LOC100060270 // rhombotin-2-like // --- // 100060270 /// ENSECAT0000000	LOC100060270	Cluster0005
14987838	XM_001492722 // LOC100060477 // septin-10-like // --- // 100060477 /// ENSECAT000000155	LOC100060477	Cluster0005
15012649	XM_001501592 // LOC100060529 // zinc finger protein 148-like // --- // 100060529 /// EN	LOC100060529	Cluster0005
14969419	XM_001914718 // LOC100060647 // catalase-like // --- // 100060647 /// ENSECAT0000002124	LOC100060647	Cluster0005
15012829	XM_001502137 // LOC100060695 // coiled-coil domain-containing protein 58-like // --- //	LOC100060695	Cluster0005
14945692	XM_001917992 // LOC100060833 // electron transfer flavoprotein subunit alpha, mitochond	LOC100060833	Cluster0005
15115022	XM_001492985 // LOC100060853 // n-lysine methyltransferase SETD8-like // --- // 1000608	LOC100060853	Cluster0005
14985439	XM_003362796 // LOC100061029 // transcription elongation regulator 1-like // --- // 100	LOC100061029	Cluster0005
15003877	XM_003363200 // LOC100061123 // ubiquitin-associated domain-containing protein 2-like /	LOC100061123	Cluster0005
15015046	XM_001502844 // LOC100061223 // v-type proton ATPase catalytic subunit A-like // --- //	LOC100061223	Cluster0005
15029666	XM_001493337 // LOC100061378 // translocon-associated protein subunit alpha-like // ---	LOC100061378	Cluster0005
14974702	ENSECAT00000000171 // LOC100061601 // probable palmitoyltransferase ZDHHC4-like // ---	LOC100061601	Cluster0005
15106713	ENSECAT00000014660 // LOC100061628 // coiled-coil domain-containing protein 90B, mitoch	LOC100061628	Cluster0005
14979519	XM_001493576 // LOC100061739 // leucine carboxyl methyltransferase 1-like // --- // 100	LOC100061739	Cluster0005
15064226	ENSECAT00000011996 // LOC100062120 // homocysteine-responsive endoplasmic reticulum-res	LOC100062120	Cluster0005
15086535	ENSECAT00000022095 // LOC100062274 // zinc transporter ZIP1-like // --- // 100062274 //	LOC100062274	Cluster0005
14974659	XM_001493987 // LOC100062410 // vacuolar fusion protein CCZ1 homolog // --- // 10006241	LOC100062410	Cluster0005
14982994	ENSECAT00000013217 // LOC100062974 // voltage-dependent anion-selective channel protein	LOC100062974	Cluster0005
15047852	XM_001494497 // LOC100063171 // serine/threonine-protein phosphatase 4 regulatory subun	LOC100063171	Cluster0005
14998265	XM_001495342 // LOC100064018 // 28S ribosomal protein S22, mitochondrial-like // --- //	LOC100064018	Cluster0005
15096269	XM_001502130 // LOC100064206 // pyridine nucleotide-disulfide oxidoreductase domain-con	LOC100064206	Cluster0005
14983413	ENSECAT00000017203 // LOC100064254 // geranylgeranyl transferase type-1 subunit beta-li	LOC100064254	Cluster0005
15122229	ENSECAT00000009942 // LOC100064267 // receptor-binding cancer antigen expressed on SiSo	LOC100064267	Cluster0005
15096294	XM_001917026 // LOC100064384 // n-acylneuraminate cytidylyltransferase-like // --- // 1	LOC100064384	Cluster0005
14959726	XM_001495314 // LOC100064401 // coiled-coil domain-containing protein 43-like // --- //	LOC100064401	Cluster0005
15126186	ENSECAT00000011610 // LOC100064665 // transmembrane protein 19-like // --- // 100064665	LOC100064665	Cluster0005
14932655	ENSECAT00000012836 // LOC100064881 // arginyl-tRNA--protein transferase 1-like // --- /	LOC100064881	Cluster0005
15071959	ENSECAT00000009746 // LOC100064893 // phosphoglucomutase-2-like // --- // 100064893 ///	LOC100064893	Cluster0005
15036711	XM_001495797 // LOC100065101 // thioredoxin-related transmembrane protein 4-like // ---	LOC100065101	Cluster0005
14983835	XM_001504620 // LOC100065122 // centrin-3-like // --- // 100065122 /// ENSECAT000000266	LOC100065122	Cluster0005
15106967	ENSECAT00000007395 // LOC100065199 // potassium voltage-gated channel subfamily E membe	LOC100065199	Cluster0005
15069097	ENSECAT00000021919 // LOC100065490 // cytoplasmic dynein 1 light intermediate chain 2-l	LOC100065490	Cluster0005
15108855	ENSECAT00000000324 // LOC100065544 // general transcription factor IIF subunit 1-like /	LOC100065544	Cluster0005
15107050	ENSECAT00000024371 // LOC100065545 // ras-related protein Rab-6A-like // --- // 1000655	LOC100065545	Cluster0005
14940734	XM_001496247 // LOC100065740 // alpha-1,6-mannosyl-glycoprotein 2-beta-N-acetylglucosam	LOC100065740	Cluster0005
15085585	ENSECAT00000014997 // LOC100065940 // succinate dehydrogenase cytochrome b560 subunit,	LOC100065940	Cluster0005
15026919	ENSECAT00000016162 // LOC100066056 // protein FAM8A1-like // --- // 100066056 /// XM_00	LOC100066056	Cluster0005
14943787	ENSECAT00000005161 // LOC100066087 // ras-related protein Rab-4A-like // --- // 1000660	LOC100066087	Cluster0005
15085551	XM_001503803 // LOC100066089 // NADH dehydrogenase [ubiquinone] iron-sulfur protein 2,	LOC100066089	Cluster0005
15048672	XM_001496562 // LOC100066214 // DDB1- and CUL4-associated factor 10-like // --- // 1000	LOC100066214	Cluster0005
15029922	XM_001496593 // LOC100066265 // protein DEK-like // --- // 100066265 /// ENSECAT0000001	LOC100066265	Cluster0005
15079178	XM_001496641 // LOC100066339 // putative RNA-binding protein Luc7-like 2-like // --- //	LOC100066339	Cluster0005

14956950	XM_001503983 // LOC100066515 // sorting nexin-3-like // --- // 100066515 /// ENSECAT000	LOC100066515	Cluster0005
14940797	XM_001916196 // LOC100066874 // thioredoxin-related transmembrane protein 1-like // ---	LOC100066874	Cluster0005
15058538	ENSECAT00000020229 // LOC100066907 // cholinephosphotransferase 1-like // --- // 100066	LOC100066907	Cluster0005
15050039	XM_001500465 // LOC100066929 // protein SET-like // --- // 100066929 /// ENSECAT0000000	LOC100066929	Cluster0005
15028684	ENSECAT00000003760 // LOC100067007 // 60S ribosomal protein L7-like 1-like // --- // 10	LOC100067007	Cluster0005
14992134	ENSECAT00000022445 // LOC100067047 // pentatricopeptide repeat-containing protein 3, mi	LOC100067047	Cluster0005
15096123	XM_001497503 // LOC100067460 // putative deoxyribose-phosphate aldolase-like // --- //	LOC100067460	Cluster0005
14988770	XM_001497585 // LOC100067584 // succinyl-CoA ligase [GDP-forming] subunit alpha, mitoch	LOC100067584	Cluster0005
15030027	ENSECAT00000015105 // LOC100067631 // tyrosyl-DNA phosphodiesterase 2-like // --- // 10	LOC100067631	Cluster0005
14977584	ENSECAT00000025328 // LOC100067691 // H(+)/Cl(-) exchange transporter 7-like // --- //	LOC100067691	Cluster0005
14953331	ENSECAT00000020831 // LOC100067729 // TATA box-binding protein-like protein 1-like // -	LOC100067729	Cluster0005
15058669	XM_001497703 // LOC100067735 // host cell factor 2-like // --- // 100067735 /// ENSECAT	LOC100067735	Cluster0005
14935854	XM_001498076 // LOC100068202 // UPF0235 protein C15orf40 homolog // --- // 100068202 //	LOC100068202	Cluster0005
14975063	XM_001505046 // LOC100068657 // COP9 signalosome complex subunit 6-like // --- // 10006	LOC100068657	Cluster0005
15088803	ENSECAT00000015423 // LOC100069110 // uncharacterized LOC100069110 // --- // 100069110	LOC100069110	Cluster0005
14938044	ENSECAT00000014788 // LOC100069498 // uncharacterized protein KIAA1370-like // --- // 1	LOC100069498	Cluster0005
15052962	XM_001499306 // LOC100069565 // uridine-cytidine kinase 1-like // --- // 100069565 ///	LOC100069565	Cluster0005
15109512	ENSECAT00000008687 // LOC100069736 // protein NPAT-like // --- // 100069736 /// XM_0019	LOC100069736	Cluster0005
14956253	XM_001503661 // LOC100069784 // protein FAM46A-like // --- // 100069784 /// ENSECAT0000	LOC100069784	Cluster0005
14938217	ENSECAT00000008737 // LOC100069832 // signal peptide peptidase-like 2A-like // --- // 1	LOC100069832	Cluster0005
15068182	XM_001499798 // LOC100070117 // wolframin-like // --- // 100070117 /// ENSECAT000000137	LOC100070117	Cluster0005
15014621	ENSECAT00000011003 // LOC100070138 // run domain Beclin-1 interacting and cystein-rich	LOC100070138	Cluster0005
15059222	XM_001917207 // LOC100070427 // adenylosuccinate lyase-like // --- // 100070427 /// ENS	LOC100070427	Cluster0005
15012813	XM_001500297 // LOC100070623 // importin subunit alpha-1-like // --- // 100070623 /// E	LOC100070623	Cluster0005
14938550	ENSECAT00000001341 // LOC100070629 // spatacsin-like // --- // 100070629	LOC100070629	Cluster0005
14947085	ENSECAT00000012136 // LOC100070880 // cyclin-D1-binding protein 1-like // --- // 100070	LOC100070880	Cluster0005
15011044	XM_001917879 // LOC100070939 // BTB/POZ domain-containing protein KCTD18-like // --- //	LOC100070939	Cluster0005
15061360	XM_001500736 // LOC100071050 // NADH-cytochrome b5 reductase 3-like // --- // 100071050	LOC100071050	Cluster0005
15040751	ENSECAT00000023895 // LOC100071419 // beta-1,4-galactosyltransferase 5-like // --- // 1	LOC100071419	Cluster0005
15038464	XM_003363892 // LOC100071474 // CCAAT/enhancer-binding protein beta-like // --- // 1000	LOC100071474	Cluster0005
15036193	ENSECAT00000025728 // LOC100071527 // uncharacterized protein KIAA0947-like // --- // 1	LOC100071527	Cluster0005
15082596	ENSECAT00000016823 // LOC100071546 // uncharacterized LOC100071546 // --- // 100071546	LOC100071546	Cluster0005
14966974	XM_001501508 // LOC100071676 // zinc finger protein 830-like // --- // 100071676 /// EN	LOC100071676	Cluster0005
15018160	XM_001501765 // LOC100071914 // aflatoxin B1 aldehyde reductase member 2-like // --- //	LOC100071914	Cluster0005
14985527	XM_001502004 // LOC100072138 // NEDD4 family-interacting protein 1-like // --- // 10007	LOC100072138	Cluster0005
14994375	XM_001502047 // LOC100072175 // ATP-dependent RNA helicase DDX1-like // --- // 10007217	LOC100072175	Cluster0005
15013472	XM_001917325 // LOC100072274 // mitochondrial import receptor subunit TOM70-like // ---	LOC100072274	Cluster0005
15015391	ENSECAT00000026105 // LOC100072293 // TBC1 domain family member 23-like // --- // 10007	LOC100072293	Cluster0005
14952833	XM_001504039 // LOC100072369 // ribosome production factor 2 homolog // --- // 10007236	LOC100072369	Cluster0005
14985641	XM_001502291 // LOC100072378 // zinc finger matrin-type protein 2-like // --- // 100072	LOC100072378	Cluster0005
15108310	ENSECAT00000020331 // LOC100072557 // uncharacterized protein C11orf46 homolog // --- /	LOC100072557	Cluster0005
14943242	XM_001502612 // LOC100072614 // graves disease carrier protein-like // --- // 100072614	LOC100072614	Cluster0005
15026308	XM_001502751 // LOC100072722 // AP-1 complex-associated regulatory protein-like // ---	LOC100072722	Cluster0005
14985966	XM_001502761 // LOC100072729 // thioredoxin domain-containing protein 15-like // --- //	LOC100072729	Cluster0005
14957301	ENSECAT00000009844 // LOC100072790 // golgi-associated PDZ and coiled-coil motif-contai	LOC100072790	Cluster0005
14991186	ENSECAT00000005716 // LOC100072837 // 1,2-dihydroxy-3-keto-5-methylthiopentene dioxygen	LOC100072837	Cluster0005
15015545	XM_001502968 // LOC100072898 // putative methyltransferase NSUN3-like // --- // 1000728	LOC100072898	Cluster0005
14953042	ENSECAT00000025228 // LOC100072974 // acid sphingomyelinase-like phosphodiesterase 3a-l	LOC100072974	Cluster0005
14983486	XM_001503567 // LOC100073191 // stAR-related lipid transfer protein 4-like // --- // 10	LOC100073191	Cluster0005
14986817	ENSECAT00000026323 // LOC100073223 // endoplasmic reticulum aminopeptidase 2-like // --	LOC100073223	Cluster0005
14987439	ENSECAT00000012513 // LOC100073289 // beta-hexosaminidase subunit beta-like // --- // 1	LOC100073289	Cluster0005
14964443	XM_001916777 // LOC100146544 // CST complex subunit TEN1-like // --- // 100146544 /// E	LOC100146544	Cluster0005
14975942	XM_001915497 // LOC100146790 // myc-associated zinc finger protein-like // --- // 10014	LOC100146790	Cluster0005
15090442	XM_003364970 // LOC100629275 // SLAM family member 9-like // --- // 100629275 /// ENSEC	LOC100629275	Cluster0005

15068578	ENSECAT00000009337 // LOC100629296 // UPF0547 protein C16orf87-like // --- // 100629296	LOC100629296	Cluster0005
15097300	ENSECAT00000021394 // LOC100629527 // transcription factor Sp1-like // --- // 100629527	LOC100629527	Cluster0005
15078126	ENSECAT00000014353 // LOC100630238 // e3 ubiquitin-protein ligase RNF6-like // --- // 1	LOC100630238	Cluster0005
15032646	XM_003363819 // LOC100630400 // DET1- and DDB1-associated protein 1-like // --- // 1006	LOC100630400	Cluster0005
15041007	XM_003363950 // LOC100630568 // ATP synthase subunit epsilon, mitochondrial-like // ---	LOC100630568	Cluster0005
15119372	ENSECAT00000019060 // LOC100630847 // uncharacterized LOC100630847 // --- // 100630847	LOC100630847	Cluster0005
15063887	ENSECAT00000024215 // LONP2 // lon peptidase 2, peroxisomal // --- // 100057497 /// XM_	LONP2	Cluster0005
15018994	ENSECAT00000024120 // LRRC47 // leucine rich repeat containing 47 // --- // 100146297	LRRC47	Cluster0005
14997384	XM_001489140 // LRRFIP2 // leucine rich repeat (in FLII) interacting protein 2 // --- /	LRRFIP2	Cluster0005
15014504	ENSECAT00000019224 // LSG1 // large subunit GTPase 1 homolog (S. cerevisiae) // --- //	LSG1	Cluster0005
15054941	XM_001915192 // LTN1 // listerin E3 ubiquitin protein ligase 1 // --- // 100065649 ///	LTN1	Cluster0005
15091950	ENSECAT00000021179 // MAN1A2 // mannosidase, alpha, class 1A, member 2 // --- // --- //	MAN1A2	Cluster0005
15006261	ENSECAT00000023172 // MAP3K2 // mitogen-activated protein kinase kinase kinase 2 // ---	MAP3K2	Cluster0005
14956629	XM_001503783 // MAP3K7 // mitogen-activated protein kinase kinase kinase 7 // --- // 10	MAP3K7	Cluster0005
15133517	ENSECAT00000000633 // MAP7D3 // MAP7 domain containing 3 // --- // ---	MAP7D3	Cluster0005
15118497	ENSECAT00000010509 // MAPKAPK5 // mitogen-activated protein kinase-activated protein ki	MAPKAPK5	Cluster0005
14985781	NM_001205261 // MATR3 // matrin 3 // --- // 100062181 /// ENSECAT00000013816 // MATR3 /	MATR3	Cluster0005
14966357	ENSECAT00000024684 // MBTD1 // mbt domain containing 1 // --- // 100070259 /// XM_00149	MBTD1	Cluster0005
15127245	ENSECAT00000000582 // MBTPS2 // membrane-bound transcription factor peptidase, site 2 /	MBTPS2	Cluster0005
15123661	XM_001487951 // MCM4 // minichromosome maintenance complex component 4 // --- // 100051	MCM4	Cluster0005
15116496	XM_001499374 // ME2 // malic enzyme 2, NAD(+)-dependent, mitochondrial // --- // 100069	ME2	Cluster0005
14957549	ENSECAT00000026671 // MED23 // mediator complex subunit 23 // --- // 100067556	MED23	Cluster0005
14985124	ENSECAT00000018975 // MFAP3 // microfibrillar-associated protein 3 // --- // 100071417	MFAP3	Cluster0005
14981370	ENSECAT00000011626 // MGAT4B // mannosyl (alpha-1,3-)-glycoprotein beta-1,4-N-acetylglu	MGAT4B	Cluster0005
15093517	ENSECAT00000022197 // MIER1 // mesoderm induction early response 1 homolog (Xenopus lae	MIER1	Cluster0005
15114871	ENSECAT00000012727 // MLXIP // MLX interacting protein // --- // 100060001	MLXIP	Cluster0005
15035560	ENSECAT00000009638 // MRPS30 // mitochondrial ribosomal protein S30 // --- // ---	MRPS30	Cluster0005
15126938	XM_001488963 // MSL3 // male-specific lethal 3 homolog (Drosophila) // --- // 100054131	MSL3	Cluster0005
15129970	ENSECAT00000014290 // MST4 // serine/threonine protein kinase MST4 // --- // 100057553	MST4	Cluster0005
15121849	ENSECAT00000011528 // MTDH // metadherin // --- // 100059885 /// XM_001492316 // MTDH /	MTDH	Cluster0005
15117714	ENSECAT00000020990 // MTMR3 // myotubularin related protein 3 // --- // 100058825 /// X	MTMR3	Cluster0005
15018112	XM_001504339 // MUL1 // mitochondrial E3 ubiquitin protein ligase 1 // --- // 100058446	MUL1	Cluster0005
15015574	XM_001914674 // MYSM1 // Myb-like, SWIRM and MPN domains 1 // --- // 100146753 /// ENSE	MYSM1	Cluster0005
14960947	XM_001502506 // MYST2 // K(lysine) acetyltransferase 7 // --- // 100056161 /// ENSECAT0	MYST2	Cluster0005
15055813	ENSECAT00000010292 // MYST3 // K(lysine) acetyltransferase 6A // --- // 100050371 /// E	MYST3	Cluster0005
15025836	XM_001502496 // NAA15 // N(alpha)-acetyltransferase 15, NatA auxiliary subunit // --- /	NAA15	Cluster0005
15008235	ENSECAT00000022068 // NBEAL1 // neurobeachin-like 1 // --- // ---	NBEAL1	Cluster0005
14998185	XM_001499088 // NCK1 // NCK adaptor protein 1 // --- // 100053397 /// ENSECAT0000000807	NCK1	Cluster0005
14963499	XM_001918389 // NCOR1 // nuclear receptor corepressor 1 // --- // 100063285 /// ENSECAT	NCOR1	Cluster0005
15090624	ENSECAT00000009997 // NCSTN // nicastrin // --- // 100058515 /// XM_001504431 // NCSTN	NCSTN	Cluster0005
14985204	ENSECAT00000018788 // NDST1 // N-deacetylase/N-sulfotransferase (heparan glucosaminyl)	NDST1	Cluster0005
15011304	XM_001505117 // NDUFS1 // NADH dehydrogenase (ubiquinone) Fe-S protein 1, 75kDa (NADH-c	NDUFS1	Cluster0005
15047422	ENSECAT00000019965 // NEK9 // NIMA (never in mitosis gene a)- related kinase 9 // --- /	NEK9	Cluster0005
14948903	ENSECAT00000018933 // NEMF // nuclear export mediator factor // --- // 100065895	NEMF	Cluster0005
14997632	ENSECAT00000024825 // NGLY1 // N-glycanase 1 // --- // 100059456 /// XM_001492043 // NG	NGLY1	Cluster0005
15019312	XM_001496756 // NOC2L // nucleolar complex associated 2 homolog (S. cerevisiae) // ---	NOC2L	Cluster0005
14937043	ENSECAT00000008566 // NPTN // neuroplastin // --- // 100052106 /// XM_001493720 // NPTN	NPTN	Cluster0005
14959508	ENSECAT00000011306 // NSF // N-ethylmaleimide-sensitive factor // --- // 100063690 ///	NSF	Cluster0005
14967499	XM_001918341 // NUFIP2 // nuclear fragile X mental retardation protein interacting prot	NUFIP2	Cluster0005
15059572	ENSECAT00000017312 // NUP50 // nucleoporin 50kDa // --- // 100051494 /// XM_001488115 /	NUP50	Cluster0005
15128658	XM_001493372 // OGT // O-linked N-acetylglucosamine (GlcNAc) transferase // --- // 1000	OGT	Cluster0005
15011113	XM_001503615 // ORC2 // origin recognition complex, subunit 2 // --- // 100067880 /// E	ORC2	Cluster0005
15038743	ENSECAT00000010959 // OSBPL2 // oxysterol binding protein-like 2 // --- // 100051127 //	OSBPL2	Cluster0005
15001122	ENSECAT00000020926 // OXSR1 // oxidative-stress responsive 1 // --- // 100050017 /// XM	OXSR1	Cluster0005

15002681	XM_001492258 // PAN3 // PAN3 poly(A) specific ribonuclease subunit homolog (S. cerevisi	PAN3	Cluster0005
14981798	ENSECAT00000022424 // PANK3 // pantothenate kinase 3 // --- // 100059344 /// XM_0015032	PANK3	Cluster0005
14939581	XM_001502551 // PARP2 // poly (ADP-ribose) polymerase 2 // --- // 100072572 /// ENSECAT	PARP2	Cluster0005
15065341	ENSECAT00000025543 // PDPR // pyruvate dehydrogenase phosphatase regulatory subunit //	PDPR	Cluster0005
15020785	ENSECAT00000026349 // PGRMC2 // progesterone receptor membrane component 2 // --- // --	PGRMC2	Cluster0005
14964268	XM_001490971 // PGS1 // phosphatidylglycerophosphate synthase 1 // --- // 100057717 ///	PGS1	Cluster0005
15130820	ENSECAT00000010527 // PIGA // phosphatidylinositol glycan anchor biosynthesis, class A	PIGA	Cluster0005
15023115	ENSECAT00000017998 // PIGV // phosphatidylinositol glycan anchor biosynthesis, class V	PIGV	Cluster0005
15076539	XM_001491173 // PION // pigeon homolog (Drosophila) // --- // 100058064 /// ENSECAT0000	PION	Cluster0005
15100625	ENSECAT00000015675 // PLBD1 // phospholipase B domain containing 1 // --- // 100066968	PLBD1	Cluster0005
14956725	ENSECAT00000021157 // PNISR // PNN-interacting serine/arginine-rich protein // --- // -	PNISR	Cluster0005
15067222	NM_001105316 // PPAT // phosphoribosyl pyrophosphate amidotransferase // --- // 1000538	PPAT	Cluster0005
15019736	XM_001494107 // PPP2R2A // protein phosphatase 2, regulatory subunit B, alpha // --- //	PPP2R2A	Cluster0005
15046932	XM_001498781 // PPP2R5E // protein phosphatase 2, regulatory subunit B , epsilon isofor	PPP2R5E	Cluster0005
15106512	ENSECAT00000025164 // PRAM1 // PML-RARA regulated adaptor molecule 1 // --- // ---	PRAM1	Cluster0005
15106728	XM_001917218 // PRCP // prolylcarboxypeptidase (angiotensinase C) // --- // 100061830 /	PRCP	Cluster0005
15011560	ENSECAT00000015325 // PRKCI // protein kinase C, iota // --- // 100063737 /// XM_001494	PRKCI	Cluster0005
14990088	XM_001500870 // PRKD3 // protein kinase D3 // --- // 100054187 /// ENSECAT00000009228 /	PRKD3	Cluster0005
15063484	ENSECAT00000007036 // PRPF18 // PRP18 pre-mRNA processing factor 18 homolog (S. cerevis	PRPF18	Cluster0005
14951634	XM_001488065 // PRPF31 // PRP31 pre-mRNA processing factor 31 homolog (S. cerevisiae) /	PRPF31	Cluster0005
14940690	XM_001493445 // PRPF39 // PRP39 pre-mRNA processing factor 39 homolog (S. cerevisiae) /	PRPF39	Cluster0005
15009273	XM_003363256 // PRPF40A // PRP40 pre-mRNA processing factor 40 homolog A (S. cerevisiae	PRPF40A	Cluster0005
15113604	XM_001496455 // PRR14L // proline rich 14-like // --- // 100062987 /// ENSECAT000000236	PRR14L	Cluster0005
15000535	XM_001495506 // PTPN23 // protein tyrosine phosphatase, non-receptor type 23 // --- //	PTPN23	Cluster0005
15017381	XM_001916895 // PUM1 // pumilio homolog 1 (Drosophila) // --- // 100070514 /// ENSECAT0	PUM1	Cluster0005
15110348	XM_001505092 // PUS3 // pseudouridylate synthase 3 // --- // 100064416 /// ENSECAT00000	PUS3	Cluster0005
15074336	ENSECAT00000019433 // RAB3GAP2 // RAB3 GTPase activating protein subunit 2 (non-catalyt	RAB3GAP2	Cluster0005
14986096	XM_001504442 // RAD50 // RAD50 homolog (S. cerevisiae) // --- // 100063198 /// ENSECAT0	RAD50	Cluster0005
15003831	ENSECAT00000013783 // RAP2A // RAP2A, member of RAS oncogene family // --- // ---	RAP2A	Cluster0005
15005551	NM_001163827 // RBM26 // RNA binding motif protein 26 // --- // 100050021 /// ENSECAT00	RBM26	Cluster0005
15109559	XM_001501445 // RDX // radixin // --- // 100061858 /// ENSECAT00000004590 // RDX // rad	RDX	Cluster0005
15067714	ENSECAT00000025999 // RFC1 // replication factor C (activator 1) 1, 145kDa // --- // 10	RFC1	Cluster0005
14983652	ENSECAT00000019853 // RIOK2 // RIO kinase 2 (yeast) // --- // 100064864 /// XM_00150460	RIOK2	Cluster0005
14967080	ENSECAT00000015507 // RNF135 // ring finger protein 135 // --- // ---	RNF135	Cluster0005
14985534	ENSECAT00000003450 // RNF14 // ring finger protein 14 // --- // ---	RNF14	Cluster0005
14953126	XM_001504242 // RNF146 // ring finger protein 146 // --- // 100067400 /// ENSECAT000000	RNF146	Cluster0005
15123972	XM_001492262 // RNF19A // ring finger protein 19A, E3 ubiquitin protein ligase // --- /	RNF19A	Cluster0005
15051394	ENSECAT00000017322 // RNF38 // ring finger protein 38 // --- // 100055331 /// XM_001504	RNF38	Cluster0005
15004541	ENSECAT00000014237 // RNF6 // ring finger protein (C3H2C3 type) 6 // --- // 100050743 /	RNF6	Cluster0005
15119329	XM_001488687 // RNMT // RNA (guanine-7-) methyltransferase // --- // 100050190 /// ENSE	RNMT	Cluster0005
14961469	XM_001503753 // RPS6KB1 // ribosomal protein S6 kinase, 70kDa, polypeptide 1 // --- //	RPS6KB1	Cluster0005
15071190	XM_001487986 // RUFY3 // RUN and FYVE domain containing 3 // --- // 100049913 /// ENSEC	RUFY3	Cluster0005
15118851	XM_001497884 // SBNO1 // strawberry notch homolog 1 (Drosophila) // --- // 100059018 //	SBNO1	Cluster0005
15076040	ENSECAT00000006620 // SCAF8 // SR-related CTD-associated factor 8 // --- // 100061852 /	SCAF8	Cluster0005
15036301	DQ402987 // SDHA // succinate dehydrogenase complex, subunit A, flavoprotein (Fp) // --	SDHA	Cluster0005
14964348	XM_001492545 // SEC14L1 // SEC14-like 1 (S. cerevisiae) // --- // 100050884 /// ENSECAT	SEC14L1	Cluster0005
14934769	ENSECAT00000023302 // SEC24C // SEC24 family, member C (S. cerevisiae) // --- // 100072	SEC24C	Cluster0005
14938354	XM_001499778 // SECISBP2L // SECIS binding protein 2-like // --- // 100070100 /// ENSEC	SECISBP2L	Cluster0005
15012170	XM_001498854 // SENP2 // SUMO1/sentrin/SMT3 specific peptidase 2 // --- // 100059276 //	SENP2	Cluster0005
15014563	XM_001499415 // SENP5 // SUMO1/sentrin specific peptidase 5 // --- // 100069684 /// ENS	SENP5	Cluster0005
15058977	ENSECAT00000008380 // SH3BP1 // SH3-domain binding protein 1 // --- // 100069810 /// EN	SH3BP1	Cluster0005
15075572	ENSECAT00000007454 // SHPRH // SNF2 histone linker PHD RING helicase, E3 ubiquitin prot	SHPRH	Cluster0005
15011579	ENSECAT00000011074 // SKIL // SKI-like oncogene // --- // 100057606 /// XM_001492164 //	SKIL	Cluster0005
14988470	ENSECAT00000015243 // SLC20A1 // solute carrier family 20 (phosphate transporter), memb	SLC20A1	Cluster0005

15087825	ENSECAT00000009428 // SLC25A24 // solute carrier family 25 (mitochondrial carrier; phos	SLC25A24	Cluster0005
15002364	ENSECAT00000024040 // SLC33A1 // solute carrier family 33 (acetyl-CoA transporter), mem	SLC33A1	Cluster0005
15015111	ENSECAT00000005619 // SLC35A5 // solute carrier family 35, member A5 // --- // 10007147	SLC35A5	Cluster0005
15064853	ENSECAT00000025033 // SLC7A6 // solute carrier family 7 (amino acid transporter light c	SLC7A6	Cluster0005
15102620	ENSECAT00000022539 // SMARCC2 // SWI/SNF related, matrix associated, actin dependent re	SMARCC2	Cluster0005
14990912	ENSECAT00000024027 // SMC6 // structural maintenance of chromosomes 6 // --- // 1000720	SMC6	Cluster0005
15043881	ENSECAT00000022765 // SMU1 // smu-1 suppressor of mec-8 and unc-52 homolog (C. elegans)	SMU1	Cluster0005
15081783	XM_001496289 // SNX13 // sorting nexin 13 // --- // 100066218 /// ENSECAT00000023365 //	SNX13	Cluster0005
15110516	XM_001917928 // SNX19 // sorting nexin 19 // --- // 100072714 /// ENSECAT00000026141 //	SNX19	Cluster0005
15036539	XM_001493978 // SNX5 // sorting nexin 5 // --- // 100062401 /// ENSECAT00000018594 // S	SNX5	Cluster0005
15004780	XM_001495257 // SPG20 // spastic paraplegia 20 (Troyer syndrome) // --- // 100062426 //	SPG20	Cluster0005
14990047	NM_001195588 // SRSF7 // serine/arginine-rich splicing factor 7 // --- // 100054033 ///	SRSF7	Cluster0005
15129773	ENSECAT00000023019 // STAG2 // stromal antigen 2 // --- // 100055046 /// XM_001915150 /	STAG2	Cluster0005
14990184	XM_001917846 // STRN // striatin, calmodulin binding protein // --- // 100054418 /// EN	STRN	Cluster0005
14961996	XM_001504156 // SUPT6H // suppressor of Ty 6 homolog (S. cerevisiae) // --- // 10005934	SUPT6H	Cluster0005
14934479	XM_001917989 // SUPV3L1 // suppressor of var1, 3-like 1 (S. cerevisiae) // --- // 10007	SUPV3L1	Cluster0005
15044077	XM_001494629 // SYK // spleen tyrosine kinase // --- // 100063383 /// ENSECAT0000001022	SYK	Cluster0005
15114320	XM_001490500 // TAOK3 // TAO kinase 3 // --- // 100050932 /// ENSECAT00000007673 // TAO	TAOK3	Cluster0005
15024160	ENSECAT00000008644 // TARDBP // TAR DNA binding protein // --- // 100051482	TARDBP	Cluster0005
14975900	ENSECAT00000024613 // TBC1D10B // TBC1 domain family, member 10B // --- // 100063852 //	TBC1D10B	Cluster0005
14951274	XM_001917360 // TBC1D17 // TBC1 domain family, member 17 // --- // 100056274 /// ENSECA	TBC1D17	Cluster0005
15021209	XM_003364530 // TBCK // TBC1 domain containing kinase // --- // 100064433 /// ENSECAT00	TBCK	Cluster0005
15123552	ENSECAT00000020705 // TGS1 // trimethylguanosine synthase 1 // --- // 100067973 /// XM_	TGS1	Cluster0005
15119564	ENSECAT00000010001 // THOC1 // THO complex 1 // --- // 100051155 /// XM_001491890 // TH	THOC1	Cluster0005
15022499	XM_001503634 // THRAP3 // thyroid hormone receptor associated protein 3 // --- // 10005	THRAP3	Cluster0005
15003888	XM_001492116 // TM9SF2 // transmembrane 9 superfamily member 2 // --- // 100061300 ///	TM9SF2	Cluster0005
14946652	ENSECAT00000022946 // TMOD3 // tropomodulin 3 (ubiquitous) // --- // 100069657 /// XM_0	TMOD3	Cluster0005
15126983	NM_001163948 // TMSB4X // thymosin beta 4, X-linked // --- // 100034015 /// ENSECAT0000	TMSB4X	Cluster0005
15058116	XM_001915050 // TMTC3 // transmembrane and tetratricopeptide repeat containing 3 // ---	TMTC3	Cluster0005
14972182	ENSECAT00000022523 // TRAF6 // TNF receptor-associated factor 6, E3 ubiquitin protein l	TRAF6	Cluster0005
15011156	ENSECAT00000011350 // TRAK2 // trafficking protein, kinesin binding 2 // --- // 1000662	TRAK2	Cluster0005
15004983	XM_001488575 // TRIM13 // tripartite motif containing 13 // --- // 100055194 /// ENSECA	TRIM13	Cluster0005
15033055	ENSECAT00000001411 // TRIM23 // tripartite motif containing 23 // --- // 100050517 ///	TRIM23	Cluster0005
14952092	ENSECAT00000011246 // TRIM28 // tripartite motif containing 28 // --- // ---	TRIM28	Cluster0005
14946342	EF397511 // TRIP4 // thyroid hormone receptor interactor 4 // --- // 100053730	TRIP4	Cluster0005
15126166	XM_001488158 // TRMT2A // tRNA methyltransferase 2 homolog A (S. cerevisiae) // --- //	TRMT2A	Cluster0005
15073553	XM_001490824 // TROVE2 // TROVE domain family, member 2 // --- // 100051140 /// ENSECAT	TROVE2	Cluster0005
14968998	ENSECAT00000005911 // TRPV2 // transient receptor potential cation channel, subfamily V	TRPV2	Cluster0005
15125043	XM_001505003 // TSTA3 // tissue specific transplantation antigen P35B // --- // 1000634	TSTA3	Cluster0005
14935417	ENSECAT00000020934 // TTC13 // tetratricopeptide repeat domain 13 // --- // 100061024	TTC13	Cluster0005
14998400	XM_001493057 // U2SURP // U2 snRNP-associated SURP domain containing // --- // 10005118	U2SURP	Cluster0005
15127796	XM_001492947 // UBA1 // ubiquitin-like modifier activating enzyme 1 // --- // 100060792	UBA1	Cluster0005
15118011	ENSECAT00000009561 // UBE3B // ubiquitin protein ligase E3B // --- // 100059725 /// XM_	UBE3B	Cluster0005
15079156	ENSECAT00000025101 // UBN2 // ubinuclein 2 // --- // ---	UBN2	Cluster0005
15128147	NM_001256912 // UBQLN2 // ubiquilin 2 // --- // 100050402	UBQLN2	Cluster0005
15028626	ENSECAT00000022605 // UBR2 // ubiquitin protein ligase E3 component n-recognin 2 // ---	UBR2	Cluster0005
15123526	ENSECAT00000018083 // UBXN2B // UBX domain protein 2B // --- // ---	UBXN2B	Cluster0005
15056386	XM_001490682 // UFSP2 // UFM1-specific peptidase 2 // --- // 100057294 /// ENSECAT00000	UFSP2	Cluster0005
15098603	ENSECAT00000019768 // USP37 // ubiquitin specific peptidase 37 // --- // 100055721 ///	USP37	Cluster0005
14996451	XM_001497962 // USP4 // ubiquitin specific peptidase 4 (proto-oncogene) // --- // 10005	USP4	Cluster0005
14956739	XM_001503881 // USP45 // ubiquitin specific peptidase 45 // --- // 100071616 /// ENSECA	USP45	Cluster0005
15107779	XM_001501192 // USP47 // ubiquitin specific peptidase 47 // --- // 100071433 /// ENSECA	USP47	Cluster0005
14976986	ENSECAT00000010341 // USP7 // ubiquitin specific peptidase 7 (herpes virus-associated)	USP7	Cluster0005
14946719	XM_001501923 // USP8 // ubiquitin specific peptidase 8 // --- // 100055443 /// ENSECAT0	USP8	Cluster0005

15111797	ENSECAT00000025789 // UVRAG // UV radiation resistance associated gene // --- // 100051	UVRAG	Cluster0005
15063432	NM_001243145 // VIM // vimentin // --- // 100056088 /// ENSECAT00000006522 // VIM // vi	VIM	Cluster0005
15104857	ENSECAT00000007408 // VPS11 // vacuolar protein sorting 11 homolog (S. cerevisiae) // -	VPS11	Cluster0005
15118731	XM_001496784 // VPS33A // vacuolar protein sorting 33 homolog A (S. cerevisiae) // ---	VPS33A	Cluster0005
14935664	ENSECAT00000017845 // WAPAL // wings apart-like homolog (Drosophila) // --- // 10005252	WAPAL	Cluster0005
15098907	XM_001915567 // WDFY1 // WD repeat and FYVE domain containing 1 // --- // 100061271 ///	WDFY1	Cluster0005
15046289	ENSECAT00000014118 // WDR20 // WD repeat domain 20 // --- // 100055061 /// XM_001917644	WDR20	Cluster0005
15001041	XM_001498297 // WDR48 // WD repeat domain 48 // --- // 100068457 /// ENSECAT00000020117	WDR48	Cluster0005
15093950	ENSECAT00000017868 // XRCC5 // X-ray repair complementing defective repair in Chinese h	XRCC5	Cluster0005
15002015	ENSECAT00000008803 // XRN1 // 5 -3 exoribonuclease 1 // --- // 100051335	XRN1	Cluster0005
15039078	XM_003363927 // XRN2 // 5 -3 exoribonuclease 2 // --- // 100050094 /// ENSECAT00000020	XRN2	Cluster0005
15119610	XM_001492336 // YES1 // v-yes-1 Yamaguchi sarcoma viral oncogene homolog 1 // --- // 10	YES1	Cluster0005
15015809	XM_001488992 // YIPF1 // Yip1 domain family, member 1 // --- // 100050435 /// ENSECAT00	YIPF1	Cluster0005
15045268	ENSECAT00000017280 // YLPM1 // YLP motif containing 1 // --- // ---	YLPM1	Cluster0005
15063055	XM_001495933 // YME1L1 // YME1-like 1 (S. cerevisiae) // --- // 100065311 /// ENSECAT00	YME1L1	Cluster0005
15089252	ENSECAT00000009946 // YOD1 // YOD1 OTU deubiquinating enzyme 1 homolog (S. cerevisiae)	YOD1	Cluster0005
15059709	NM_001256974 // ZBED4 // zinc finger, BED-type containing 4 // --- // 100054656 /// ENS	ZBED4	Cluster0005
15013371	AY246739 // ZBTB11 // zinc finger and BTB domain containing 11 // --- // 100072181 ///	ZBTB11	Cluster0005
14956999	XM_001504012 // ZBTB24 // zinc finger and BTB domain containing 24 // --- // 100066674	ZBTB24	Cluster0005
15049721	NM_001257178 // ZBTB34 // zinc finger and BTB domain containing 34 // --- // 100070589	ZBTB34	Cluster0005
14976869	XM_001491187 // ZC3H7A // zinc finger CCCH-type containing 7A // --- // 100050963 /// E	ZC3H7A	Cluster0005
15125532	XM_001495455 // ZFC3H1 // zinc finger, C3H1-type containing // --- // 100064600 /// ENS	ZFC3H1	Cluster0005
15063820	XM_001501531 // ZMYND11 // zinc finger, MYND-type containing 11 // --- // 100057731 ///	ZMYND11	Cluster0005
14978073	ENSECAT00000017355 // ZNF12 // zinc finger protein 12 // --- // --- /// ENSECAT00000017	ZNF12	Cluster0005
15034053	XM_001917248 // ZNF622 // zinc finger protein 622 // --- // 100069990 /// ENSECAT000000	ZNF622	Cluster0005
14940842	ENSECAT00000018811 // ZNF717 // zinc finger protein 717 // --- // --- /// ENSECAT000000	ZNF717	Cluster0005
14952066	XM_003362245 // ZNF8 // zinc finger protein 8 // --- // 100051604 /// ENSECAT0000001413	ZNF8	Cluster0005
15021494	ENSECAT00000011700 // ZYG11B // zyg-11 homolog B (C. elegans) // --- // 100061587 /// X	ZYG11B	Cluster0005
15088593	ENSECAT00000011906 // ZZZ3 // zinc finger, ZZ-type containing 3 // --- // 100053222 ///	ZZZ3	Cluster0005
