# Supplementary material for: Comparative transcriptome analysis of equine alveolar macrophages
Source: Equine Vet J. 2016 Jul 9;49(3):375–82. doi: 10.1111/evj.12584 (PMC5412682; doi:10.1111/evj.12584)
Supplement: Supplementary file 3 [file EVJ-49-375-s003.pdf]

Supplementary Item 1c: Using BIOLAYOUT : Top clusters of genes more highly expressed in PMs

Transcript ID	Gene_assignment	Gene symbol	MCL_2.2
15013425	ENSECAT00000002615 // ABI3BP // ABI family, member 3 (NESH) binding protein // --- // -	ABI3BP	Cluster0001
14987992	ENSECAT00000000702 // AFF3 // AF4/FMR2 family, member 3 // --- // 100059904 /// XM_0014	AFF3	Cluster0001
15037472	NM_001164017 // ASIP // agouti signaling protein // --- // 100054335 /// ENSECAT0000000	ASIP	Cluster0001
14965220	ENSECAT00000016191 // C17orf57 // chromosome 17 open reading frame 57 // --- // ---	C17orf57	Cluster0001
15006416	ENSECAT00000005374 // C1QL2 // complement component 1, q subcomponent-like 2 // --- //	C1QL2	Cluster0001
14984686	ENSECAT00000019573 // C5orf50 // chromosome 5 open reading frame 50 // --- // ---	C5orf50	Cluster0001
15030959	ENSECAT00000017521 // C6orf10 // chromosome 6 open reading frame 10 // --- // ---	C6orf10	Cluster0001
15043815	ENSECAT00000022313 // C9orf11 // chromosome 9 open reading frame 11 // --- // ---	C9orf11	Cluster0001
15080773	ENSECAT00000018737 // CAMK2B // calcium/calmodulin-dependent protein kinase II beta //	CAMK2B	Cluster0001
15078434	NM_001114143 // CAV1 // caveolin 1, caveolae protein, 22kDa // --- // 100055975 /// ENS	CAV1	Cluster0001
15106039	ENSECAT00000005738 // CCDC151 // coiled-coil domain containing 151 // --- // ---	CCDC151	Cluster0001
14964224	XM_001490355 // CCDC40 // coiled-coil domain containing 40 // --- // 100056721 /// ENSE	CCDC40	Cluster0001
14969456	NM_001085435 // CD44 // CD44 molecule (Indian blood group) // --- // 100034221 /// ENSE	CD44	Cluster0001
15065561	NM_001109812 // CDH13 // cadherin 13, H-cadherin (heart) // --- // 100055760 /// ENSECA	CDH13	Cluster0001
15132900	XM_001489536 // CHRDL1 // chordin-like 1 // --- // 100059105 /// ENSECAT00000012735 //	CHRDL1	Cluster0001
15058700	XM_001498399 // CHST11 // carbohydrate (chondroitin 4) sulfotransferase 11 // --- // 10	CHST11	Cluster0001
15093090	XM_001494684 // CLCA4 // chloride channel accessory 4 // --- // 100063474 /// ENSECAT00	CLCA4	Cluster0001
15118745	ENSECAT00000010525 // CLIP1 // CAP-GLY domain containing linker protein 1 // --- // ---	CLIP1	Cluster0001
15110136	XM_001501513 // CLMP // CXADR-like membrane protein // --- // 100071682 /// ENSECAT0000	CLMP	Cluster0001
15024953	NM_001081944 // CLU // clusterin // --- // 100034172 /// ENSECAT00000007460 // CLU // c	CLU	Cluster0001
14966267	ENSECAT00000020087 // COL1A1 // collagen, type I, alpha 1 // --- // --- /// ENSECAT0000	COL1A1	Cluster0001
15100521	ENSECAT00000014627 // CSDA // cold shock domain protein A // --- // ---	CSDA	Cluster0001
14965860	ENSECAT00000008953 // CSF3 // colony stimulating factor 3 (granulocyte) // --- // 10003	CSF3	Cluster0001
15016922	XM_001499027 // CSF3R // colony stimulating factor 3 receptor (granulocyte) // --- // 1	CSF3R	Cluster0001
15071075	AF053497 // CXCL1 // chemokine (C-X-C motif) ligand 1 (melanoma growth stimulating acti	CXCL1	Cluster0001
15071093	ENSECAT00000013283 // CXCL6 // chemokine (C-X-C motif) ligand 6 (granulocyte chemotacti	CXCL6	Cluster0001
15093139	XM_001495078 // CYR61 // cysteine-rich, angiogenic inducer, 61 // --- // 100064066 ///	CYR61	Cluster0001
15060222	NM_001081925 // DCN // decorin // --- // 100034120 /// ENSECAT00000021671 // DCN // dec	DCN	Cluster0001
14951083	ENSECAT00000026053 // DKKL1 // dickkopf-like 1 // --- // ---	DKKL1	Cluster0001
14952887	XM_001502677 // DSE // dermatan sulfate epimerase // --- // 100072666 /// ENSECAT000000	DSE	Cluster0001
15084109	ENSECAT00000000771 // DUSP27 // dual specificity phosphatase 27 (putative) // --- // 10	DUSP27	Cluster0001
15095741	JQ713942 // ENO2 // enolase 2 (gamma, neuronal) // --- // 100052776	ENO2	Cluster0001
14973124	ENSECAT00000025250 // FADS1 // fatty acid desaturase 1 // --- // ---	FADS1	Cluster0001
15060308	ENSECAT00000022648 // FGD6 // FYVE, RhoGEF and PH domain containing 6 // --- // 1000651	FGD6	Cluster0001
15055978	AY246707 // FGFR1 // fibroblast growth factor receptor 1 // --- // 100057614 /// XM_001	FGFR1	Cluster0001
15011594	XM_001493622 // FNDC3B // fibronectin type III domain containing 3B // --- // 100057824	FNDC3B	Cluster0001

14966176	ENSECAT00000008325 // GIP // gastric inhibitory polypeptide // --- // ---	GIP	Cluster0001
14941646	ENSECAT00000024517 // HABP2 // hyaluronan binding protein 2 // --- // 100068293 /// XM_	HABP2	Cluster0001
15098184	ENSECAT00000029138 // HMGA2 // high mobility group AT-hook 2 // --- // ---	HMGA2	Cluster0001
15082025	XM_001499476 // HOXA6 // homeobox A6 // --- // 100054438 /// ENSECAT00000000495 // HOXA	HOXA6	Cluster0001
15043742	NM_001114537 // IFN-ALPHA2 // interferon-alpha-2 // --- // 100052818 /// ENSECAT0000000	IFN-ALPHA2	Cluster0001
15043747	NM_001114537 // IFN-ALPHA2 // interferon-alpha-2 // --- // 100052818 /// NM_001114538 /	IFN-ALPHA2	Cluster0001
15080887	AY752932 // IGFBP-3 // insulin-like growth factor binding protein-3 // --- // 100034155	IGFBP-3	Cluster0001
15089212	ENSECAT00000009338 // IL10 // interleukin 10 // --- // 100034187 /// NM_001082490 // IL	IL10	Cluster0001
14991995	ENSECAT00000025409 // IL1A // interleukin 1, alpha // --- // 100064969 /// NM_001082500	IL1A	Cluster0001
15126653	NM_001082526 // IL1B // interleukin 1, beta // --- // 100034237 /// ENSECAT00000000066	IL1B	Cluster0001
15097702	ENSECAT00000023772 // IL23A // interleukin 23, alpha subunit p19 // --- // 100034230 //	IL23A	Cluster0001
15071098	ENSECAT00000016212 // IL8 // interleukin 8 // --- // 100037400 /// NM_001083951 // IL8	IL8	Cluster0001
14965242	NM_001081802 // ITGB3 // integrin, beta 3 (platelet glycoprotein IIIa, antigen CD61) //	ITGB3	Cluster0001
15077811	XM_001497221 // ITGB8 // integrin, beta 8 // --- // 100053462 /// ENSECAT00000020530 //	ITGB8	Cluster0001
15041816	ENSECAT00000017225 // KANK1 // KN motif and ankyrin repeat domains 1 // --- // 10005075	KANK1	Cluster0001
15131319	XM_001916706 // LOC100049934 // ferritin heavy chain-like // --- // 100049934 /// ENSEC	LOC100049934	Cluster0001
15083298	ENSECAT00000022470 // LOC100050047 // cationic trypsin-3-like // --- // 100050047 /// X	LOC100050047	Cluster0001
14991447	XM_001488300 // LOC100050144 // transmembrane protein 182-like // --- // 100050144 ///	LOC100050144	Cluster0001
15083354	XM_001491416 // LOC100050381 // olfactory receptor 9A4-like // --- // 100050381 /// ENS	LOC100050381	Cluster0001
15002234	XM_001491224 // LOC100050742 // transmembrane 4 L6 family member 1-like // --- // 10005	LOC100050742	Cluster0001
15023913	ENSECAT00000005694 // LOC100050750 // transmembrane protein 51-like // --- // 100050750	LOC100050750	Cluster0001
15088053	ENSECAT00000010311 // LOC100051002 // rho GTPase-activating protein 29-like // --- // 1	LOC100051002	Cluster0001
15097521	XM_001504776 // LOC100051379 // 11-cis retinol dehydrogenase-like // --- // 100051379 /	LOC100051379	Cluster0001
15030212	XM_001505026 // LOC100052624 // histone H2A type 1-like // --- // 100052624 /// ENSECAT	LOC100052624	Cluster0001
15030121	XM_001496648 // LOC100052966 // histone H1.1-like // --- // 100052966 /// ENSECAT000000	LOC100052966	Cluster0001
14950897	XM_001488567 // LOC100053135 // epididymal sperm-binding protein 1-like // --- // 10005	LOC100053135	Cluster0001
15000429	ENSECAT00000018328 // LOC100053301 // t-cell leukemia translocation-altered gene protei	LOC100053301	Cluster0001
15095862	ENSECAT00000026701 // LOC100053371 // solute carrier family 2, facilitated glucose tran	LOC100053371	Cluster0001
15027133	ENSECAT00000006961 // LOC100053545 // histone H2A type 1-C-like // --- // 100053545	LOC100053545	Cluster0001
15027187	XM_001505090 // LOC100053619 // high mobility group nucleosome-binding domain-containin	LOC100053619	Cluster0001
15085659	XM_001504439 // LOC100053816 // signaling lymphocytic activation molecule-like // --- /	LOC100053816	Cluster0001
15129160	XM_001493412 // LOC100053876 // transcription elongation factor A protein-like 3-like /	LOC100053876	Cluster0001
15107360	XM_001503971 // LOC100054010 // olfactory receptor 51I1-like // --- // 100054010 /// EN	LOC100054010	Cluster0001
15073333	ENSECAT00000019204 // LOC100054102 // uncharacterized protein C1orf115 homolog // --- /	LOC100054102	Cluster0001
15088985	ENSECAT00000007565 // LOC100054345 // vesicle transport protein GOT1A-like // --- // 10	LOC100054345	Cluster0001
15115552	XM_001489105 // LOC100054445 // NADH dehydrogenase [ubiquinone] flavoprotein 2, mitocho	LOC100054445	Cluster0001
15030482	ENSECAT00000018981 // LOC100054813 // uncharacterized protein C6orf15-like // --- // 10	LOC100054813	Cluster0001
14997427	ENSECAT00000021523 // LOC100054924 // serine/threonine-protein kinase DCLK3-like // ---	LOC100054924	Cluster0001
15079475	ENSECAT00000016877 // LOC100055297 // anionic trypsin-like // --- // 100055297 /// XM_0	LOC100055297	Cluster0001

15115595	XM_001489548 // LOC100055301 // thioredoxin domain-containing protein 2-like // --- //	LOC100055301	Cluster0001
15078295	ENSECAT00000013708 // LOC100055422 // neuropeptide S receptor-like // --- // 100055422	LOC100055422	Cluster0001
15030475	XM_001491016 // LOC100055430 // radiation-inducible immediate-early gene IEX-1-like //	LOC100055430	Cluster0001
15072077	XM_001499463 // LOC100055629 // sodium-dependent phosphate transport protein 2B-like //	LOC100055629	Cluster0001
15066864	ENSECAT00000020789 // LOC100055761 // ras association domain-containing protein 6-like	LOC100055761	Cluster0001
15079527	ENSECAT00000003584 // LOC100056093 // transmembrane protein 139-like // --- // 10005609	LOC100056093	Cluster0001
14958187	XM_001490118 // LOC100056324 // neuronal pentraxin-1-like // --- // 100056324 /// ENSEC	LOC100056324	Cluster0001
15032076	XM_001503079 // LOC100056506 // glutathione S-transferase A2-like // --- // 100056506 /	LOC100056506	Cluster0001
15045250	XM_001490453 // LOC100056601 // visual system homeobox 2-like // --- // 100056601 /// E	LOC100056601	Cluster0001
15085821	ENSECAT00000003234 // LOC100056753 // olfactory receptor 6K3-like // --- // 100056753 /	LOC100056753	Cluster0001
15084141	ENSECAT00000007097 // LOC100056894 // myelin protein zero-like protein 1-like // --- //	LOC100056894	Cluster0001
15122289	XM_001496635 // LOC100057055 // protein NOV homolog // --- // 100057055 /// ENSECAT0000	LOC100057055	Cluster0001
14994605	XM_001503611 // LOC100057240 // DNA-binding protein inhibitor ID-2-like // --- // 10005	LOC100057240	Cluster0001
14961550	ENSECAT00000003546 // LOC100057520 // vacuolar protein sorting 72 homolog (S. cerevisia	LOC100057520	Cluster0001
14941178	XM_001490947 // LOC100057676 // G-protein coupled receptor 26-like // --- // 100057676	LOC100057676	Cluster0001
15045327	ENSECAT00000017213 // LOC100057690 // basic leucine zipper transcriptional factor ATF-l	LOC100057690	Cluster0001
15024826	XM_001914958 // LOC100057715 // phosphatidylethanolamine-binding protein 4-like // ---	LOC100057715	Cluster0001
15027352	XM_001491116 // LOC100057985 // olfactory receptor 2B2-like // --- // 100057985 /// ENS	LOC100057985	Cluster0001
15090447	ENSECAT00000016862 // LOC100058208 // uncharacterized protein C1orf226-like // --- // 1	LOC100058208	Cluster0001
15029606	XM_001491438 // LOC100058493 // PX domain-containing protein C6orf145-like // --- // 10	LOC100058493	Cluster0001
15088044	XM_001491449 // LOC100058506 // tissue factor-like // --- // 100058506 /// ENSECAT00000	LOC100058506	Cluster0001
15133078	XM_001491814 // LOC100058519 // RING finger protein 113A-like // --- // 100058519 /// E	LOC100058519	Cluster0001
15090340	XM_001493635 // LOC100058590 // uridine-cytidine kinase 2-like // --- // 100058590 ///	LOC100058590	Cluster0001
15053450	ENSECAT00000006431 // LOC100058638 // protein FAM166A-like // --- // 100058638 /// XM_0	LOC100058638	Cluster0001
15006835	ENSECAT00000002082 // LOC100058814 // keratin, type I cytoskeletal 18-like // --- // 10	LOC100058814	Cluster0001
14991548	ENSECAT00000009189 // LOC100058919 // mitogen-activated protein kinase kinase kinase ki	LOC100058919	Cluster0001
15103237	ENSECAT00000020804 // LOC100058976 // interleukin-22-like // --- // 100058976 /// XM_00	LOC100058976	Cluster0001
15129211	XM_001503067 // LOC100059187 // protein BEX3-like // --- // 100059187 /// ENSECAT000000	LOC100059187	Cluster0001
15129209	XM_001503086 // LOC100059264 // WW domain-binding protein 5-like // --- // 100059264 //	LOC100059264	Cluster0001
15076209	ENSECAT00000023278 // LOC100060503 // uronyl 2-sulfotransferase-like // --- // 10006050	LOC100060503	Cluster0001
15129144	ENSECAT00000001318 // LOC100060576 // protein ARMCX6-like // --- // 100060576 /// XM_00	LOC100060576	Cluster0001
15056500	XM_001493010 // LOC100060890 // vascular endothelial growth factor C-like // --- // 100	LOC100060890	Cluster0001
15094377	ENSECAT00000006785 // LOC100061005 // potassium voltage-gated channel subfamily E membe	LOC100061005	Cluster0001
15100237	ENSECAT00000013235 // LOC100061006 // homeobox protein NANOG-like // --- // 100061006	LOC100061006	Cluster0001
14991679	ENSECAT00000011743 // LOC100061333 // cyclic nucleotide-gated cation channel alpha-3-li	LOC100061333	Cluster0001
15111265	XM_001493466 // LOC100061569 // olfactory receptor 7D4-like // --- // 100061569 /// ENS	LOC100061569	Cluster0001
15013212	ENSECAT00000001188 // LOC100061617 // zinc finger BED domain-containing protein 2-like	LOC100061617	Cluster0001
15091406	ENSECAT00000010407 // LOC100061730 // protein S100-A9-like // --- // 100061730 /// XM_0	LOC100061730	Cluster0001
14944245	XM_001493698 // LOC100061933 // olfactory receptor 6C4-like // --- // 100061933 /// ENS	LOC100061933	Cluster0001

15127437	XM_001493804 // LOC100062107 // melanoma-associated antigen B10-like // --- // 10006210	LOC100062107	Cluster0001
15015428	ENSECAT00000020647 // LOC100062187 // collagen alpha-1(VIII) chain-like // --- // 10006	LOC100062187	Cluster0001
15064310	ENSECAT00000019145 // LOC100062332 // c-C motif chemokine 22-like // --- // 100062332 /	LOC100062332	Cluster0001
14970456	XM_001494183 // LOC100062709 // fatty acid desaturase 2-like // --- // 100062709 /// EN	LOC100062709	Cluster0001
14996478	XM_001494391 // LOC100063006 // uncharacterized LOC100063006 // --- // 100063006 /// EN	LOC100063006	Cluster0001
15104828	XM_001503043 // LOC100063180 // uroplakin-2-like // --- // 100063180 /// ENSECAT0000001	LOC100063180	Cluster0001
15130574	XM_001494562 // LOC100063279 // olfactory receptor 10R2-like // --- // 100063279 /// EN	LOC100063279	Cluster0001
14943309	XM_001503727 // LOC100063458 // leucine-rich repeat-containing protein 20-like // --- /	LOC100063458	Cluster0001
15016149	XM_001494678 // LOC100063464 // UPF0632 protein A-like // --- // 100063464 /// ENSECAT0	LOC100063464	Cluster0001
15075503	XM_001495199 // LOC100064229 // NKG2D ligand 4-like // --- // 100064229 /// ENSECAT0000	LOC100064229	Cluster0001
15111813	XM_001495302 // LOC100064387 // diacylglycerol O-acyltransferase 2-like // --- // 10006	LOC100064387	Cluster0001
15085955	XM_001500763 // LOC100064714 // ETS translocation variant 3-like // --- // 100064714 //	LOC100064714	Cluster0001
15102421	ENSECAT00000007630 // LOC100064738 // protein phosphatase 1 regulatory subunit 1A-like	LOC100064738	Cluster0001
15048002	XM_001495656 // LOC100064891 // interferon alpha-inducible protein 27-like protein 2-li	LOC100064891	Cluster0001
15110862	ENSECAT00000023383 // LOC100064925 // zinc finger protein 709-like // --- // 100064925	LOC100064925	Cluster0001
15087145	ENSECAT00000023956 // LOC100065052 // gap junction alpha-5 protein-like // --- // 10006	LOC100065052	Cluster0001
15064509	XM_001495994 // LOC100065396 // CKLF-like MARVEL transmembrane domain-containing protei	LOC100065396	Cluster0001
15019280	ENSECAT00000021228 // LOC100066167 // tumor necrosis factor receptor superfamily member	LOC100066167	Cluster0001
15091909	ENSECAT00000019862 // LOC100066884 // hydroxyacid oxidase 2-like // --- // 100066884 //	LOC100066884	Cluster0001
15132687	ENSECAT00000013894 // LOC100067023 // transcription elongation factor A protein-like 4-	LOC100067023	Cluster0001
14972820	XM_001497247 // LOC100067132 // olfactory receptor 476-like // --- // 100067132 /// ENS	LOC100067132	Cluster0001
15012098	XM_001497358 // LOC100067260 // transmembrane epididymal protein 1-like // --- // 10006	LOC100067260	Cluster0001
14970114	XM_001497463 // LOC100067396 // olfactory receptor 1S1-like // --- // 100067396 /// ENS	LOC100067396	Cluster0001
15066093	XM_001497779 // LOC100067821 // zinc transporter ZIP8-like // --- // 100067821 /// ENSE	LOC100067821	Cluster0001
15078999	ENSECAT00000007157 // LOC100068106 // non-muscle caldesmon-like // --- // 100068106 ///	LOC100068106	Cluster0001
15053644	XR_036256 // LOC100068798 // calponin-3-like // --- // 100068798 /// XM_001490827 // LO	LOC100068798	Cluster0001
15068034	XM_001498741 // LOC100068923 // fibroblast growth factor-binding protein 1-like // ---	LOC100068923	Cluster0001
14977278	XM_003362763 // LOC100069286 // pyrin-like // --- // 100069286 /// ENSECAT00000006974 /	LOC100069286	Cluster0001
15130275	XM_001499147 // LOC100069378 // uncharacterized LOC100069378 // --- // 100069378 /// EN	LOC100069378	Cluster0001
15112287	XM_001499184 // LOC100069414 // olfactory receptor 52H1-like // --- // 100069414 /// EN	LOC100069414	Cluster0001
15068091	ENSECAT00000015583 // LOC100069539 // cytokine-dependent hematopoietic cell linker-like	LOC100069539	Cluster0001
15035987	XM_001501124 // LOC100069914 // brain acid soluble protein 1-like // --- // 100069914 /	LOC100069914	Cluster0001
15059084	XM_001499732 // LOC100070048 // ER lumen protein retaining receptor 3-like // --- // 10	LOC100070048	Cluster0001
14990082	XM_001499765 // LOC100070088 // cytochrome P450 1B1-like // --- // 100070088 /// ENSECA	LOC100070088	Cluster0001
15034562	XM_001914909 // LOC100070775 // olfactory receptor 10H4-like // --- // 100070775 /// EN	LOC100070775	Cluster0001
15012958	ENSECAT00000019225 // LOC100070833 // follistatin-related protein 1-like // --- // 1000	LOC100070833	Cluster0001
15040462	XM_001503192 // LOC100070893 // antileukoproteinase-like // --- // 100070893 /// ENSECA	LOC100070893	Cluster0001
15061292	XM_001502807 // LOC100070895 // cytochrome P450 2D14-like // --- // 100070895 /// ENSEC	LOC100070895	Cluster0001
15112574	ENSECAT00000008816 // LOC100071044 // achaete-scute homolog 3-like // --- // 100071044	LOC100071044	Cluster0001

15023175	XM_001500815 // LOC100071130 // CAMPATH-1 antigen-like // --- // 100071130 /// ENSECAT0	LOC100071130	Cluster0001
14990369	ENSECAT00000002281 // LOC100071152 // coiled-coil domain-containing protein 121-like //	LOC100071152	Cluster0001
14934007	XM_001500917 // LOC100071214 // phospholipase A2-like // --- // 100071214 /// ENSECAT00	LOC100071214	Cluster0001
14966722	ENSECAT00000019709 // LOC100071342 // uncharacterized protein C17orf64-like // --- // 1	LOC100071342	Cluster0001
14985258	XM_001501485 // LOC100071660 // homeobox protein CDX-1-like // --- // 100071660 /// ENS	LOC100071660	Cluster0001
15110169	XM_001501632 // LOC100071779 // transmembrane protein 225-like // --- // 100071779 ///	LOC100071779	Cluster0001
15078788	XM_001501658 // LOC100071801 // tetraspanin-33-like // --- // 100071801 /// ENSECAT0000	LOC100071801	Cluster0001
15110190	XM_001501768 // LOC100071915 // olfactory receptor 8D4-like // --- // 100071915 /// ENS	LOC100071915	Cluster0001
14994346	XM_001501905 // LOC100072043 // mesogenin-1-like // --- // 100072043 /// ENSECAT0000000	LOC100072043	Cluster0001
14947737	ENSECAT00000006789 // LOC100072163 // olfactory receptor 4F3/4F16/4F29-like // --- // 1	LOC100072163	Cluster0001
14967613	XM_001502177 // LOC100072280 // diazepam-binding inhibitor-like 5-like // --- // 100072	LOC100072280	Cluster0001
14967860	XM_001918357 // LOC100072577 // olfactory receptor 1A1-like // --- // 100072577 /// ENS	LOC100072577	Cluster0001
14967870	XM_001502636 // LOC100072630 // olfactory receptor 3A2-like // --- // 100072630 /// ENS	LOC100072630	Cluster0001
14939687	ENSECAT00000002318 // LOC100072707 // ribonuclease 7-like // --- // 100072707 /// XM_00	LOC100072707	Cluster0001
15085890	XM_001915440 // LOC100146177 // olfactory receptor 10K2-like // --- // 100146177 /// EN	LOC100146177	Cluster0001
15036426	XM_001915341 // LOC100146206 // thrombomodulin-like // --- // 100146206 /// ENSECAT0000	LOC100146206	Cluster0001
14954197	XM_001916316 // LOC100146413 // cytochrome P450 2A13-like // --- // 100146413 /// XM_00	LOC100146413	Cluster0001
14954007	ENSECAT00000021129 // LOC100147106 // uncharacterized LOC100147106 // --- // 100147106	LOC100147106	Cluster0001
15112423	XM_001917853 // LOC100147393 // olfactory receptor 2D2-like // --- // 100147393 /// ENS	LOC100147393	Cluster0001
15113767	ENSECAT00000015807 // LOC100629131 // leukemia inhibitory factor-like // --- // 1006291	LOC100629131	Cluster0001
14939795	ENSECAT00000011369 // LOC100629138 // uncharacterized LOC100629138 // --- // 100629138	LOC100629138	Cluster0001
15010912	ENSECAT00000021150 // LOC100629147 // uncharacterized protein C2orf66-like // --- // 10	LOC100629147	Cluster0001
14984257	XM_003362891 // LOC100629259 // multiple myeloma tumor-associated protein 2-like // ---	LOC100629259	Cluster0001
15068530	XM_003364698 // LOC100629290 // ATP synthase subunit e, mitochondrial-like // --- // 10	LOC100629290	Cluster0001
14999527	ENSECAT00000010605 // LOC100629337 // sentan-like // --- // 100629337 /// XM_003363081	LOC100629337	Cluster0001
15056545	ENSECAT00000007133 // LOC100629456 // beta-defensin 103A-like // --- // 100629456 /// X	LOC100629456	Cluster0001
15030285	XM_003363755 // LOC100629501 // olfactory receptor 2W1-like // --- // 100629501 /// ENS	LOC100629501	Cluster0001
14939913	XM_003363631 // LOC100629686 // ig kappa chain V-III region PC 2485/PC 4039-like // ---	LOC100629686	Cluster0001
15091419	XM_003365022 // LOC100630233 // uncharacterized LOC100630233 // --- // 100630233 /// EN	LOC100630233	Cluster0001
15095597	XM_003365160 // LOC100630357 // CD27 antigen-like // --- // 100630357 /// ENSECAT000000	LOC100630357	Cluster0001
15063456	XM_003364358 // LOC100630445 // meiosis expressed gene 1 protein homolog // --- // 1006	LOC100630445	Cluster0001
15048211	XM_003364027 // LOC100630573 // uncharacterized LOC100630573 // --- // 100630573 /// EN	LOC100630573	Cluster0001
15006898	ENSECAT00000016455 // LOC100630738 // death-associated protein-like 1-like // --- // 10	LOC100630738	Cluster0001
15099792	ENSECAT00000019850 // LOC100630897 // uncharacterized protein ENSP00000372125-like // -	LOC100630897	Cluster0001
15060218	NM_001081780 // LUM // lumican // --- // 100009681 /// ENSECAT00000019349 // LUM // lum	LUM	Cluster0001
15116648	XM_001489163 // MALT1 // mucosa associated lymphoid tissue lymphoma translocation gene	MALT1	Cluster0001
14998512	ENSECAT00000008123 // MED12L // mediator complex subunit 12-like // --- // 100056881 //	MED12L	Cluster0001
15070627	ENSECAT00000026165 // MEPE // matrix extracellular phosphoglycoprotein // --- // ---	MEPE	Cluster0001
15078440	NM_001114147 // MET // met proto-oncogene (hepatocyte growth factor receptor) // --- //	MET	Cluster0001

15071077	FJ469975 // MIP-2BETA // CXCL3 // --- // 100056258 /// NM_001143793 // MIP-2BETA // CXC	MIP-2BETA	Cluster0001
14976229	ENSECAT00000009720 // MIR1255B // microRNA mir-1255b // --- // 100314985	MIR1255B	Cluster0001
15072947	ENSECAT00000016295 // MIR1255B // microRNA mir-1255b // --- // 100314985	MIR1255B	Cluster0001
15085220	ENSECAT00000023431 // MIR1255B // microRNA mir-1255b // --- // 100314985	MIR1255B	Cluster0001
15113559	NR_032877 // MIR130B // microRNA mir-130b // --- // 100314829	MIR130B	Cluster0001
14966148	NR_032903 // MIR196A // microRNA mir-196a // --- // 100314843	MIR196A	Cluster0001
15012291	NR_032968 // MIR28 // microRNA mir-28 // --- // 100314878	MIR28	Cluster0001
15085386	NR_032844 // MIR29B-2 // microRNA mir-29b-2 // --- // 100315047	MIR29B-2	Cluster0001
14958183	NR_032909 // MIR338 // microRNA mir-338 // --- // 100315012	MIR338	Cluster0001
15046172	NR_033023 // MIR382 // microRNA mir-382 // --- // 100314908	MIR382	Cluster0001
14945014	NR_032791 // MIR7 // microRNA mir-7 // --- // 100314782	MIR7	Cluster0001
15086111	NR_032849 // MIR9A // microRNA mir-9a // --- // 100314814	MIR9A	Cluster0001
15031201	NM_001163967 // MLN // motilin // --- // 100033882 /// ENSECAT00000014700 // MLN // mot	MLN	Cluster0001
14940063	ENSECAT00000009008 // MMP14 // matrix metallopeptidase 14 (membrane-inserted) // --- //	MMP14	Cluster0001
15130678	XM_001500228 // MXRA5 // matrix-remodelling associated 5 // --- // 100063681 /// ENSECA	MXRA5	Cluster0001
14937807	ENSECAT00000026465 // MYO1E // myosin IE // --- // 100068162	MYO1E	Cluster0001
15000964	ENSECAT00000006532 // MYRIP // myosin VIIA and Rab interacting protein // --- // 100068	MYRIP	Cluster0001
14991781	XM_001493945 // NCAPH // non-SMC condensin I complex, subunit H // --- // 100062353 ///	NCAPH	Cluster0001
15020168	XM_001499988 // NPY1R // neuropeptide Y receptor Y1 // --- // 100061686 /// ENSECAT0000	NPY1R	Cluster0001
15028083	ENSECAT00000014028 // PACSIN1 // protein kinase C and casein kinase substrate in neuron	PACSIN1	Cluster0001
14975354	AF508034 // PAI-1 // plasminogen activator inhibitor-1 // --- // 100033931	PAI-1	Cluster0001
14942909	XM_001501590 // PAPSS2 // 3 -phosphoadenosine 5 -phosphosulfate synthase 2 // --- // 10	PAPSS2	Cluster0001
15107842	XM_001501248 // PARVA // parvin, alpha // --- // 100071481 /// ENSECAT00000006448 // PA	PARVA	Cluster0001
15054155	ENSECAT00000012965 // PCP4 // Purkinje cell protein 4 // --- // ---	PCP4	Cluster0001
15054272	ENSECAT00000018858 // PDE9A // phosphodiesterase 9A // --- // 100057783 /// XM_00149100	PDE9A	Cluster0001
15015170	ENSECAT00000017734 // PHLDB2 // pleckstrin homology-like domain, family B, member 2 //	PHLDB2	Cluster0001
15024531	XM_001492978 // PRDM16 // PR domain containing 16 // --- // 100060844 /// ENSECAT000000	PRDM16	Cluster0001
15039487	NM_001091537 // PRND // prion protein 2 (dublet) // --- // 100048937 /// ENSECAT0000000	PRND	Cluster0001
14995104	ENSECAT00000015237 // PRRT3 // proline-rich transmembrane protein 3 // --- // 100058227	PRRT3	Cluster0001
15024131	XM_001915116 // PTCHD2 // patched domain containing 2 // --- // 100056461 /// ENSECAT00	PTCHD2	Cluster0001
15052916	NM_001081935 // PTGES // prostaglandin E synthase // --- // 100034143 /// ENSECAT000000	PTGES	Cluster0001
15038468	XM_001488269 // PTPN1 // protein tyrosine phosphatase, non-receptor type 1 // --- // 10	PTPN1	Cluster0001
15011434	ENSECAT00000004554 // PTX3 // pentraxin 3, long // --- // ---	PTX3	Cluster0001
15083493	ENSECAT00000015490 // RARRES2 // retinoic acid receptor responder (tazarotene induced)	RARRES2	Cluster0001
14953625	ENSECAT00000027119 // SBSN // suprabasin // --- // ---	SBSN	Cluster0001
14942247	ENSECAT00000015611 // SCD // stearoyl-CoA desaturase (delta-9-desaturase) // --- // 100	SCD	Cluster0001
15076479	XM_001489227 // SEMA3C // sema domain, immunoglobulin domain (Ig), short basic domain,	SEMA3C	Cluster0001
15116856	XM_003365564 // SERPINB2 // serpin peptidase inhibitor, clade B (ovalbumin), member 2 /	SERPINB2	Cluster0001
14946275	XM_001496554 // SLC24A1 // solute carrier family 24 (sodium/potassium/calcium exchanger	SLC24A1	Cluster0001

15070053	ENSECAT00000009904 // SLC7A5 // solute carrier family 7 (amino acid transporter light c	SLC7A5	Cluster0001
15119256	ENSECAT00000000446 // SOGA2 // SOGA family member 2 // --- // ---	SOGA2	Cluster0001
15081313	XM_001492219 // STEAP4 // STEAP family member 4 // --- // 100059718 /// ENSECAT00000020	STEAP4	Cluster0001
15010859	XM_001917776 // STK17B // serine/threonine kinase 17b // --- // 100054971 /// ENSECAT00	STK17B	Cluster0001
14966237	ENSECAT00000015515 // TAC4 // tachykinin 4 (hemokinin) // --- // ---	TAC4	Cluster0001
15120364	ENSECAT00000018691 // TCF7L2 // transcription factor 7-like 2 (T-cell specific, HMG-box	TCF7L2	Cluster0001
15095464	XM_001915384 // TEAD4 // TEA domain family member 4 // --- // 100057619 /// ENSECAT0000	TEAD4	Cluster0001
14947542	XM_001503599 // THBS1 // thrombospondin 1 // --- // 100057478 /// ENSECAT00000009707 //	THBS1	Cluster0001
15075897	NM_001163117 // THBS2 // thrombospondin 2 // --- // 100050044 /// ENSECAT00000023244 //	THBS2	Cluster0001
15119210	ENSECAT00000011590 // TMEM200C // transmembrane protein 200C // --- // ---	TMEM200C	Cluster0001
14946662	XM_001501574 // TMOD2 // tropomodulin 2 (neuronal) // --- // 100055095 /// XM_003363503	TMOD2	Cluster0001
15098543	ENSECAT00000015236 // TNP1 // transition protein 1 (during histone to protamine replace	TNP1	Cluster0001
14939998	ENSECAT00000017987 // TRAV40 // T cell receptor alpha variable 40 // --- // ---	TRAV40	Cluster0001
14939897	ENSECAT00000003631 // TRAV9-1 // T cell receptor alpha variable 9-1 // --- // ---	TRAV9-1	Cluster0001
15045393	ENSECAT00000022464 // VASH1 // vasohibin 1 // --- // 100051887 /// XM_001492980 // VASH	VASH1	Cluster0001
15087838	ENSECAT00000012045 // VAV3 // vav 3 guanine nucleotide exchange factor // --- // 100057	VAV3	Cluster0001
15028900	NM_001081821 // VEGFA // vascular endothelial growth factor A // --- // 100033839 /// E	VEGFA	Cluster0001
15053570	ENSECAT00000010919 // VGLL3 // vestigial like 3 (Drosophila) // --- // 100069930 /// XM	VGLL3	Cluster0001
15027294	XM_001504891 // ZKSCAN4 // zinc finger with KRAB and SCAN domains 4 // --- // 100052276	ZKSCAN4	Cluster0001
15027283	ENSECAT00000006780 // ZNF187 // zinc finger protein 187 // --- // 100061664 /// XM_0014	ZNF187	Cluster0001
15119740	ENSECAT00000006598 // ZNF521 // zinc finger protein 521 // --- // 100063937 /// XM_0014	ZNF521	Cluster0001
15120887	XM_001491115 // ZNF704 // zinc finger protein 704 // --- // 100057981 /// ENSECAT000000	ZNF704	Cluster0001
14925679	---		Cluster0008
14925709	---		Cluster0008
14925909	---		Cluster0008
14926101	---		Cluster0008
14926311	---		Cluster0008
14926347	---		Cluster0008
14926353	---		Cluster0008
14926429	---		Cluster0008
14926483	---		Cluster0008
14926517	---		Cluster0008
14926599	---		Cluster0008
14926677	---		Cluster0008
14926775	---		Cluster0008
14926853	---		Cluster0008
14926879	---		Cluster0008
14926931	---		Cluster0008
14926993	---		Cluster0008

14927463	---	Cluster0008
14927627	---	Cluster0008
14927783	---	Cluster0008
14927839	---	Cluster0008
14927871	---	Cluster0008
14927897	---	Cluster0008
14927941	---	Cluster0008
14927965	---	Cluster0008
14928103	---	Cluster0008
14928215	---	Cluster0008
14928219	---	Cluster0008
14928941	---	Cluster0008
14929149	---	Cluster0008
14929225	---	Cluster0008
14929339	---	Cluster0008
14929359	---	Cluster0008
14929439	---	Cluster0008
14929449	---	Cluster0008
14929567	---	Cluster0008
14930097	---	Cluster0008
14930105	---	Cluster0008
14930187	---	Cluster0008
14930685	---	Cluster0008
14930701	---	Cluster0008
14930995	---	Cluster0008
14931029	---	Cluster0008
14931095	---	Cluster0008
14931235	---	Cluster0008
14931237	---	Cluster0008
14931247	---	Cluster0008
14931421	---	Cluster0008
14931607	---	Cluster0008
14935613	---	Cluster0008
14939915	---	Cluster0008
14944397	---	Cluster0008
14944469	---	Cluster0008
14952137	---	Cluster0008
14952392	---	Cluster0008
14952603	---	Cluster0008

14962151	---	Cluster0008
14962272	---	Cluster0008
14973825	---	Cluster0008
14979959	---	Cluster0008
14981165	---	Cluster0008
14984378	---	Cluster0008
14985555	---	Cluster0008
14986173	---	Cluster0008
14986309	---	Cluster0008
14989310	---	Cluster0008
14993032	---	Cluster0008
14999525	---	Cluster0008
15001639	---	Cluster0008
15006577	---	Cluster0008
15010789	---	Cluster0008
15011477	---	Cluster0008
15013224	---	Cluster0008
15015663	---	Cluster0008
15029053	---	Cluster0008
15033042	---	Cluster0008
15046106	---	Cluster0008
15048739	---	Cluster0008
15049712	---	Cluster0008
15053282	---	Cluster0008
15057650	---	Cluster0008
15059385	---	Cluster0008
15067801	---	Cluster0008
15070773	---	Cluster0008
15079412	---	Cluster0008
15082710	---	Cluster0008
15086646	---	Cluster0008
15088522	---	Cluster0008
15097171	---	Cluster0008
15099749	---	Cluster0008
15102129	---	Cluster0008
15102222	---	Cluster0008
15102534	---	Cluster0008
15104006	---	Cluster0008
15105087	---	Cluster0008

15111490	---	Cluster0008
15116401	---	Cluster0008
15122250	---	Cluster0008
15123452	---	Cluster0008
15125646	---	Cluster0008
15129566	---	Cluster0008
15131760	---	Cluster0008
15132341	---	Cluster0008
15133009	---	Cluster0008
15134260	---	Cluster0008
15134366	---	Cluster0008
15134522	---	Cluster0008
15134654	---	Cluster0008
15134780	---	Cluster0008
15134820	---	Cluster0008
15135106	---	Cluster0008
15135126	---	Cluster0008
15135208	---	Cluster0008
15135270	---	Cluster0008
15135314	---	Cluster0008
15135324	---	Cluster0008
15135408	---	Cluster0008
15135760	---	Cluster0008
15135844	---	Cluster0008
15135874	---	Cluster0008
15135918	---	Cluster0008
15135954	---	Cluster0008
15136136	---	Cluster0008
15136150	---	Cluster0008
15136430	---	Cluster0008
15136462	---	Cluster0008
15136488	---	Cluster0008
15136592	---	Cluster0008
15136644	---	Cluster0008
15136776	---	Cluster0008
15136900	---	Cluster0008
15137066	---	Cluster0008
15137168	---	Cluster0008
15137240	---	Cluster0008

15137266	---		Cluster0008
15137414	---		Cluster0008
15137686	---		Cluster0008
15137708	---		Cluster0008
15137716	---		Cluster0008
15137798	---		Cluster0008
15137805	---		Cluster0008
15137870	---		Cluster0008
15137872	---		Cluster0008
15137881	---		Cluster0008
15137882	---		Cluster0008
15038886	ENSECAT00000020772 // ABHD16B // abhydrolase domain containing 16B // --- // ---	ABHD16B	Cluster0008
14963231	ENSECAT00000016409 // ARHGEF15 // Rho guanine nucleotide exchange factor (GEF) 15 // --	ARHGEF15	Cluster0008
15024516	ENSECAT00000016582 // ARHGEF16 // Rho guanine nucleotide exchange factor (GEF) 16 // --	ARHGEF16	Cluster0008
14962755	XM_001918131 // BCL6B // B-cell CLL/lymphoma 6, member B // --- // 100147131 /// ENSECA	BCL6B	Cluster0008
14943336	ENSECAT00000019253 // C10orf54 // chromosome 10 open reading frame 54 // --- // ---	C10orf54	Cluster0008
15112567	ENSECAT00000008142 // C11orf16 // chromosome 11 open reading frame 16 // --- // ---	C11orf16	Cluster0008
15107936	NM_001081854 // CALCA // calcitonin-related polypeptide alpha // --- // 100033906 /// N	CALCA	Cluster0008
15111743	ENSECAT00000016388 // CAPN5 // calpain 5 // --- // 100063478	CAPN5	Cluster0008
14974792	ENSECAT00000006863 // CARD11 // caspase recruitment domain family, member 11 // --- //	CARD11	Cluster0008
14993005	ENSECAT00000010114 // CCDC85A // coiled-coil domain containing 85A // --- // 100052299	CCDC85A	Cluster0008
15090820	NM_001252308 // CD1A7 // CD1a7 molecule // --- // 100034037 /// JF749872 // CD1A7 // CD	CD1A7	Cluster0008
15015780	ENSECAT00000000539 // CDCP2 // CUB domain containing protein 2 // --- // ---	CDCP2	Cluster0008
14948149	XM_001493841 // CEBPE // CCAAT/enhancer binding protein (C/EBP), epsilon // --- // 1000	CEBPE	Cluster0008
14947331	ENSECAT00000010310 // CHAC1 // ChaC, cation transport regulator homolog 1 (E. coli) //	CHAC1	Cluster0008
14939456	NM_001257079 // CHRM5 // cholinergic receptor, muscarinic 5 // --- // 100057856 /// ENS	CHRM5	Cluster0008
15100209	ENSECAT00000013388 // CLEC4A // C-type lectin domain family 4, member A // --- // ---	CLEC4A	Cluster0008
14961081	ENSECAT00000005184 // EPN3 // epsin 3 // --- // 100056408 /// XM_001502825 // EPN3 // e	EPN3	Cluster0008
15038308	XM_001500956 // EYA2 // eyes absent homolog 2 (Drosophila) // --- // 100071250 /// ENSE	EYA2	Cluster0008
15085905	ENSECAT00000002770 // FCRL1 // Fc receptor-like 1 // --- // ---	FCRL1	Cluster0008
15097849	NM_001163860 // GLI1 // GLI family zinc finger 1 // --- // 100033937 /// ENSECAT0000001	GLI1	Cluster0008
15128370	ENSECAT00000007595 // HEPH // hephaestin // --- // 100065379 /// ENSECAT00000008319 //	HEPH	Cluster0008
15099152	ENSECAT00000020611 // HTR2B // 5-hydroxytryptamine (serotonin) receptor 2B, G protein-c	HTR2B	Cluster0008
15083118	ENSECAT00000009043 // KLRG2 // killer cell lectin-like receptor subfamily G, member 2 /	KLRG2	Cluster0008
15102044	XM_001504373 // KRT80 // keratin 80 // --- // 100061386 /// ENSECAT00000020408 // KRT80	KRT80	Cluster0008
14955109	ENSECAT00000007582 // LHB // luteinizing hormone beta polypeptide // --- // 100054774 /	LHB	Cluster0008
15111687	XM_001493089 // LOC100051297 // thyroid hormone-inducible hepatic protein-like // --- /	LOC100051297	Cluster0008
15037061	ENSECAT00000020257 // LOC100053209 // syntenin-2-like // --- // 100053209 /// XM_001498	LOC100053209	Cluster0008
15064761	ENSECAT00000011127 // LOC100053459 // uncharacterized protein C16orf86-like // --- // 1	LOC100053459	Cluster0008

15016612	XM_001503167 // LOC100053741 // guanylin-like // --- // 100053741 /// ENSECAT0000001606	LOC100053741	Cluster0008
14948439	ENSECAT00000004616 // LOC100053921 // cathepsin G-like // --- // 100053921 /// XM_00148	LOC100053921	Cluster0008
15117334	XM_001488939 // LOC100054068 // cationic amino acid transporter 4-like // --- // 100054	LOC100054068	Cluster0008
15084600	XM_001488945 // LOC100054083 // uncharacterized LOC100054083 // --- // 100054083 /// EN	LOC100054083	Cluster0008
15133326	XM_001500119 // LOC100054460 // glucose-dependent insulinotropic receptor-like // --- /	LOC100054460	Cluster0008
15041518	ENSECAT00000011551 // LOC100054543 // solute carrier family 28 member 3-like // --- //	LOC100054543	Cluster0008
15027576	ENSECAT00000007981 // LOC100054623 // transcription factor 19-like // --- // 100054623	LOC100054623	Cluster0008
14951719	ENSECAT00000013265 // LOC100054684 // leukocyte immunoglobulin-like receptor subfamily	LOC100054684	Cluster0008
15016860	ENSECAT00000022762 // LOC100054694 // uncharacterized protein C1orf109-like // --- // 1	LOC100054694	Cluster0008
15007937	ENSECAT00000003799 // LOC100054877 // glyceraldehyde-3-phosphate dehydrogenase-like //	LOC100054877	Cluster0008
14974408	XM_001494406 // LOC100055133 // cell cycle exit and neuronal differentiation protein 1-	LOC100055133	Cluster0008
15085879	XM_001490012 // LOC100056143 // olfactory receptor 6Y1-like // --- // 100056143 /// ENS	LOC100056143	Cluster0008
15040523	ENSECAT00000001682 // LOC100056463 // uncharacterized protein C20orf165-like // --- //	LOC100056463	Cluster0008
15114522	ENSECAT00000009610 // LOC100056522 // IQ domain-containing protein D-like // --- // 100	LOC100056522	Cluster0008
15083385	XM_003364861 // LOC100056743 // olfactory receptor 2F1-like // --- // 100056743 /// ENS	LOC100056743	Cluster0008
15017538	XM_001916996 // LOC100056922 // CD164 sialomucin-like 2 protein-like // --- // 10005692	LOC100056922	Cluster0008
14939197	ENSECAT00000018101 // LOC100057278 // uncharacterized protein C15orf52 homolog // --- /	LOC100057278	Cluster0008
14964264	XM_001490814 // LOC100057363 // complement C1q tumor necrosis factor-related protein 1-	LOC100057363	Cluster0008
15057026	XM_001493384 // LOC100057875 // eukaryotic translation initiation factor 4E-binding pro	LOC100057875	Cluster0008
15027603	ENSECAT00000000873 // LOC100058321 // lymphotoxin-alpha-like // --- // 100058321 /// XM	LOC100058321	Cluster0008
15130347	XM_001491513 // LOC100058594 // extracellular matrix protein 2-like // --- // 100058594	LOC100058594	Cluster0008
15115013	XM_001497564 // LOC100058978 // ADP-ribosylation factor-like protein 6-interacting prot	LOC100058978	Cluster0008
14967390	XM_001504168 // LOC100059380 // protein PROCA1-like // --- // 100059380 /// XM_00336242	LOC100059380	Cluster0008
15127305	XM_003365749 // LOC100059831 // probable ATP-dependent RNA helicase DDX53-like // --- /	LOC100059831	Cluster0008
14999355	XM_003363061 // LOC100059905 // histone-lysine N-methyltransferase SETMAR-like // --- /	LOC100059905	Cluster0008
15133241	XM_001500572 // LOC100059927 // 60S ribosomal protein L7a-like // --- // 100059927 ///	LOC100059927	Cluster0008
15030964	XM_001492393 // LOC100059994 // patr class I histocompatibility antigen, A-126 alpha ch	LOC100059994	Cluster0008
14971746	XM_001492674 // LOC100060412 // protein TSSC4-like // --- // 100060412 /// ENSECAT00000	LOC100060412	Cluster0008
15046499	ENSECAT00000004273 // LOC100060888 // transmembrane protein C14orf180 homolog // --- //	LOC100060888	Cluster0008
15019854	ENSECAT00000016483 // LOC100061049 // nociceptin-like // --- // 100061049 /// XM_001493	LOC100061049	Cluster0008
15019999	XM_001498819 // LOC100061162 // high mobility group protein B2-like // --- // 100061162	LOC100061162	Cluster0008
15013081	ENSECAT00000026424 // LOC100061258 // basic helix-loop-helix domain-containing protein	LOC100061258	Cluster0008
15097112	ENSECAT00000008634 // LOC100062582 // ankyrin repeat domain-containing protein 33-like	LOC100062582	Cluster0008
15122678	XM_001505016 // LOC100063721 // prostate stem cell antigen-like // --- // 100063721 ///	LOC100063721	Cluster0008
15086158	ENSECAT00000022658 // LOC100064070 // progestin and adipoQ receptor family member 6-lik	LOC100064070	Cluster0008
15105273	XM_001505066 // LOC100064156 // pannexin-3-like // --- // 100064156 /// ENSECAT00000001	LOC100064156	Cluster0008
15074604	ENSECAT00000025624 // LOC100064677 // v-type proton ATPase subunit G 3-like // --- // 1	LOC100064677	Cluster0008
15082993	XM_001499721 // LOC100064880 // pleiotrophin-like // --- // 100064880 /// ENSECAT000000	LOC100064880	Cluster0008
15000633	XM_001495793 // LOC100065097 // transmembrane inner ear expressed protein-like // --- /	LOC100065097	Cluster0008

15019937	ENSECAT00000021620 // LOC100065126 // transcription factor GATA-4-like // --- // 100065	LOC100065126	Cluster0008
14977810	ENSECAT00000025119 // LOC100066340 // inactive rhomboid protein 1-like // --- // 100066	LOC100066340	Cluster0008
15118153	XM_001497207 // LOC100067080 // cationic amino acid transporter 4-like // --- // 100067	LOC100067080	Cluster0008
14932336	ENSECAT00000013004 // LOC100067156 // synaptonemal complex central element protein 1-li	LOC100067156	Cluster0008
14978331	XM_001505025 // LOC100067175 // olfactory receptor 2AE1-like // --- // 100067175 /// EN	LOC100067175	Cluster0008
15109262	ENSECAT00000022319 // LOC100068095 // calmodulin-regulated spectrin-associated protein	LOC100068095	Cluster0008
15043910	ENSECAT00000014764 // LOC100068324 // aquaporin-7-like // --- // 100068324 /// XM_00149	LOC100068324	Cluster0008
15016691	XM_001916493 // LOC100068415 // protein L-Myc-1-like // --- // 100068415 /// ENSECAT000	LOC100068415	Cluster0008
15050850	ENSECAT00000020330 // LOC100068625 // uncharacterized LOC100068625 // --- // 100068625	LOC100068625	Cluster0008
15112248	ENSECAT00000012801 // LOC100068926 // hemoglobin subunit epsilon-like // --- // 1000689	LOC100068926	Cluster0008
15016979	ENSECAT00000013270 // LOC100069339 // collagen alpha-2(VIII) chain-like // --- // 10006	LOC100069339	Cluster0008
14977270	ENSECAT00000024960 // LOC100069430 // zinc finger protein 434-like // --- // 100069430	LOC100069430	Cluster0008
15068142	XM_001499406 // LOC100069677 // neuron-specific protein family member 1-like // --- //	LOC100069677	Cluster0008
15069988	ENSECAT00000026008 // LOC100070367 // DNA replication complex GINS protein PSF2-like //	LOC100070367	Cluster0008
15049824	ENSECAT00000002327 // LOC100070483 // uncharacterized protein C9orf117-like // --- // 1	LOC100070483	Cluster0008
15049643	XM_001500624 // LOC100070953 // WD repeat-containing protein 38-like // --- // 10007095	LOC100070953	Cluster0008
15017810	ENSECAT00000024937 // LOC100071592 // uncharacterized LOC100071592 // --- // 100071592	LOC100071592	Cluster0008
15023570	XM_001501686 // LOC100071833 // group IIF secretory phospholipase A2-like // --- // 100	LOC100071833	Cluster0008
14947701	XM_001501763 // LOC100071911 // olfactory receptor 4F21-like // --- // 100071911 /// EN	LOC100071911	Cluster0008
15061494	ENSECAT00000006299 // LOC100146571 // uncharacterized protein KIAA1644-like // --- // 1	LOC100146571	Cluster0008
15038836	ENSECAT00000021544 // LOC100147373 // baculoviral IAP repeat-containing protein 7-like	LOC100147373	Cluster0008
15021566	ENSECAT00000027027 // LOC100629771 // uncharacterized protein C1orf185 homolog // --- /	LOC100629771	Cluster0008
14935553	ENSECAT00000025810 // LOC100629985 // putative protein FAM170B-like // --- // 100629985	LOC100629985	Cluster0008
15079505	ENSECAT00000010780 // LOC100630088 // uncharacterized protein C7orf34-like // --- // 10	LOC100630088	Cluster0008
14979009	XM_003362728 // LOC100630155 // alpha-hemoglobin-stabilizing protein-like // --- // 100	LOC100630155	Cluster0008
15037856	ENSECAT00000021125 // LOC100630181 // adipogenin-like // --- // 100630181 /// XM_003363	LOC100630181	Cluster0008
15034697	ENSECAT00000016492 // LOC100630452 // transmembrane protein 221-like // --- // 10063045	LOC100630452	Cluster0008
15049119	ENSECAT00000028924 // LOC100630682 // a-kinase anchor protein 2-like // --- // 10063068	LOC100630682	Cluster0008
14966321	ENSECAT00000015023 // LOC100630693 // ankyrin repeat domain-containing protein 40-like	LOC100630693	Cluster0008
14950666	ENSECAT00000001234 // MARK4 // MAP/microtubule affinity-regulating kinase 4 // --- // 1	MARK4	Cluster0008
15011522	NR_032969 // MIR551B // microRNA mir-551b // --- // 100314879	MIR551B	Cluster0008
15104929	ENSECAT00000020665 // NLRX1 // NLR family member X1 // --- // 100071383 /// XM_00150112	NLRX1	Cluster0008
15051103	ENSECAT00000020347 // NOXA1 // NADPH oxidase activator 1 // --- // ---	NOXA1	Cluster0008
15102323	ENSECAT00000022450 // NPFF // neuropeptide FF-amide peptide precursor // --- // ---	NPFF	Cluster0008
15003537	ENSECAT00000018280 // PCDH17 // protocadherin 17 // --- // 100051691 /// XM_001493601 /	PCDH17	Cluster0008
15133788	ENSECAT00000026169 // PDZD4 // PDZ domain containing 4 // --- // 100057848 /// XM_00149	PDZD4	Cluster0008
14982444	ENSECAT00000026302 // PLAC8L1 // PLAC8-like 1 // --- // 100071986 /// XM_001501837 // P	PLAC8L1	Cluster0008
15036799	XM_001494638 // PROKR2 // prokineticin receptor 2 // --- // 100052008 /// ENSECAT000000	PROKR2	Cluster0008
14997779	XM_001494698 // RFTN1 // raftlin, lipid raft linker 1 // --- // 100063495 /// ENSECAT00	RFTN1	Cluster0008

14973881	XM_001916875 // RIN1 // Ras and Rab interactor 1 // --- // 100058267 /// ENSECAT0000001	RIN1	Cluster0008
15093996	ENSECAT00000011597 // RUFY4 // RUN and FYVE domain containing 4 // --- // ---	RUFY4	Cluster0008
14973175	ENSECAT00000011722 // SCGB1A1 // secretoglobin, family 1A, member 1 (uteroglobin) // --	SCGB1A1	Cluster0008
15125265	ENSECAT00000021756 // SLC39A4 // solute carrier family 39 (zinc transporter), member 4	SLC39A4	Cluster0008
15095397	XM_001492793 // SLC6A13 // solute carrier family 6 (neurotransmitter transporter, GABA)	SLC6A13	Cluster0008
14963727	XM_001918214 // SREBF1 // sterol regulatory element binding transcription factor 1 // -	SREBF1	Cluster0008
15130439	ENSECAT00000015801 // SRPK3 // SRSF protein kinase 3 // --- // 100057808 /// XM_0014932	SRPK3	Cluster0008
14978604	ENSECAT00000028907 // SRRM3 // serine/arginine repetitive matrix 3 // --- // --- /// EN	SRRM3	Cluster0008
14943254	EF032140 // TACR2 // tachykinin receptor 2 // --- // 100034168	TACR2	Cluster0008
14985217	ENSECAT00000015347 // TCOF1 // Treacher Collins-Franceschetti syndrome 1 // --- // 1000	TCOF1	Cluster0008
15095022	XM_001916026 // TRAF3IP1 // TNF receptor-associated factor 3 interacting protein 1 // -	TRAF3IP1	Cluster0008
15059454	ENSECAT00000011631 // WBP2NL // WBP2 N-terminal like // --- // ---	WBP2NL	Cluster0008
14965298	XM_001487899 // WNT3 // wingless-type MMTV integration site family, member 3 // --- //	WNT3	Cluster0008
15131585	ENSECAT00000022390 // ZNF182 // zinc finger protein 182 // --- // 100061541 /// XM_0019	ZNF182	Cluster0008
15027253	XM_003363748 // ZSCAN16 // zinc finger and SCAN domain containing 16 // --- // 10062913	ZSCAN16	Cluster0008
14925125	---		Cluster0015
14926597	---		Cluster0015
14928247	---		Cluster0015
14928639	---		Cluster0015
14930197	---		Cluster0015
14931351	---		Cluster0015
14932293	---		Cluster0015
14945595	---		Cluster0015
14948504	---		Cluster0015
14948604	---		Cluster0015
14968552	---		Cluster0015
14972192	---		Cluster0015
14981809	---		Cluster0015
14984072	---		Cluster0015
14986928	---		Cluster0015
14991173	---		Cluster0015
14991419	---		Cluster0015
14992879	---		Cluster0015
14997530	---		Cluster0015
15006329	---		Cluster0015
15008985	---		Cluster0015
15017465	---		Cluster0015
15020483	---		Cluster0015
15026550	---		Cluster0015

15036400	---	Cluster0015
15042918	---	Cluster0015
15054822	---	Cluster0015
15055631	---	Cluster0015
15057838	---	Cluster0015
15070812	---	Cluster0015
15071285	---	Cluster0015
15072142	---	Cluster0015
15077385	---	Cluster0015
15083454	---	Cluster0015
15089956	---	Cluster0015
15094485	---	Cluster0015
15110907	---	Cluster0015
15115811	---	Cluster0015
15118658	---	Cluster0015
15128304	---	Cluster0015
15128306	---	Cluster0015
15128843	---	Cluster0015
15130282	---	Cluster0015
15130792	---	Cluster0015
15132104	---	Cluster0015
15132227	---	Cluster0015
15134011	---	Cluster0015
15134384	---	Cluster0015
15136270	---	Cluster0015
15136274	---	Cluster0015
15136312	---	Cluster0015
15136360	---	Cluster0015
15136636	---	Cluster0015
15136666	---	Cluster0015
15136702	---	Cluster0015
15136842	---	Cluster0015
15136930	---	Cluster0015
15137008	---	Cluster0015
15137194	---	Cluster0015
15137280	---	Cluster0015
15137396	---	Cluster0015
15137488	---	Cluster0015
15137560	---	Cluster0015

15137626	---		Cluster0015
15137740	---		Cluster0015
14981953	ENSECAT00000019198 // ADAM19 // ADAM metallopeptidase domain 19 // --- // ---	ADAM19	Cluster0015
15113406	NM_001081897 // ADORA2A // adenosine A2a receptor // --- // 100034039 /// ENSECAT000000	ADORA2A	Cluster0015
14962731	XM_001502998 // ALOX12 // arachidonate 12-lipoxygenase // --- // 100072916 /// ENSECAT0	ALOX12	Cluster0015
15107741	XM_001504918 // AMPD3 // adenosine monophosphate deaminase 3 // --- // 100055812 /// EN	AMPD3	Cluster0015
15066646	ENSECAT00000019611 // ANTXR2 // anthrax toxin receptor 2 // --- // 100051770 /// XM_001	ANTXR2	Cluster0015
15096376	ENSECAT00000010436 // ARNTL2 // aryl hydrocarbon receptor nuclear translocator-like 2 /	ARNTL2	Cluster0015
15120425	ENSECAT00000022867 // ATP8B1 // ATPase, aminophospholipid transporter, class I, type 8B	ATP8B1	Cluster0015
15062906	ENSECAT00000010920 // CCNY // cyclin Y // --- // ---	CCNY	Cluster0015
15036423	XM_001488842 // CD93 // CD93 molecule // --- // 100059201 /// ENSECAT00000017144 // CD9	CD93	Cluster0015
14997111	ENSECAT00000027151 // CDCP1 // CUB domain containing protein 1 // --- // 100065703 ///	CDCP1	Cluster0015
14945170	XM_001490809 // CHSY1 // chondroitin sulfate synthase 1 // --- // 100057477 /// ENSECAT	CHSY1	Cluster0015
15007756	ENSECAT00000026771 // COL3A1 // collagen, type III, alpha 1 // --- // 100034123	COL3A1	Cluster0015
15094905	NM_001257078 // CXCR7 // chemokine (C-X-C motif) receptor 7 // --- // 100057501 /// ENS	CXCR7	Cluster0015
15100585	ENSECAT00000017390 // DUSP16 // dual specificity phosphatase 16 // --- // ---	DUSP16	Cluster0015
14998235	ENSECAT00000020481 // ESYT3 // extended synaptotagmin-like protein 3 // --- // 10005187	ESYT3	Cluster0015
15026036	ENSECAT00000015464 // FGF2 // fibroblast growth factor 2 (basic) // --- // 100033955 //	FGF2	Cluster0015
15108307	ENSECAT00000020310 // FSHB // follicle stimulating hormone, beta polypeptide // --- //	FSHB	Cluster0015
15025773	XM_001915633 // GAB1 // GRB2-associated binding protein 1 // --- // 100062924 /// ENSEC	GAB1	Cluster0015
15124436	ENSECAT00000022529 // HAS2 // hyaluronan synthase 2 // --- // 100009708 /// NM_00108180	HAS2	Cluster0015
14999007	NM_001081919 // HRH1 // histamine receptor H1 // --- // 100034110 /// ENSECAT0000000441	HRH1	Cluster0015
14955834	ENSECAT00000015199 // IL11 // interleukin 11 // --- // ---	IL11	Cluster0015
14979496	NM_001081774 // IL4R // interleukin 4 receptor // --- // 791252 /// ENSECAT00000023156	IL4R	Cluster0015
14957143	ENSECAT00000020501 // LAMA4 // laminin, alpha 4 // --- // 100066875	LAMA4	Cluster0015
15073176	XM_001488715 // LBR // lamin B receptor // --- // 100056997 /// ENSECAT00000011831 // L	LBR	Cluster0015
15117594	ENSECAT00000010307 // LIMK2 // LIM domain kinase 2 // --- // 100058419 /// XM_001497153	LIMK2	Cluster0015
15116468	ENSECAT00000016251 // LIPG // lipase, endothelial // --- // 100053762 /// XM_001499159	LIPG	Cluster0015
15085901	ENSECAT00000025464 // LOC100054450 // CD5 antigen-like // --- // 100054450 /// XM_00148	LOC100054450	Cluster0015
15045827	XM_001489223 // LOC100054699 // b1 bradykinin receptor-like // --- // 100054699 /// ENS	LOC100054699	Cluster0015
15071089	ENSECAT00000013150 // LOC100055973 // platelet basic protein-like // --- // 100055973 /	LOC100055973	Cluster0015
15014240	XM_001498883 // LOC100059313 // insulin-like growth factor 2 mRNA-binding protein 2-lik	LOC100059313	Cluster0015
15054124	ENSECAT00000022937 // LOC100059969 // protein C-ets-2-like // --- // 100059969 /// XM_0	LOC100059969	Cluster0015
14970224	XM_001493378 // LOC100061443 // membrane-spanning 4-domains subfamily A member 7-like /	LOC100061443	Cluster0015
14989256	ENSECAT00000009642 // LOC100061844 // CB1 cannabinoid receptor-interacting protein 1-li	LOC100061844	Cluster0015
15026012	ENSECAT00000014299 // LOC100063739 // protein sprouty homolog 1-like // --- // 10006373	LOC100063739	Cluster0015
15111950	XM_001917453 // LOC100065672 // tumor necrosis factor receptor superfamily member 19L-l	LOC100065672	Cluster0015
15039475	ENSECAT00000012970 // LOC100065724 // phosphatidate cytidylyltransferase 2-like // ---	LOC100065724	Cluster0015
14950850	XM_001503145 // LOC100065997 // c5a anaphylatoxin chemotactic receptor-like // --- // 1	LOC100065997	Cluster0015

14949069	XM_001497325 // LOC100067227 // nidogen-2-like // --- // 100067227 /// ENSECAT000000166	LOC100067227	Cluster0015
15054811	XM_001498489 // LOC100068667 // SAM domain-containing protein SAMSN-1-like // --- // 10	LOC100068667	Cluster0015
14939007	ENSECAT00000018492 // LOC100071046 // EH domain-containing protein 4-like // --- // 100	LOC100071046	Cluster0015
14939556	XM_001502449 // LOC100072499 // olfactory receptor 11G2-like // --- // 100072499 /// EN	LOC100072499	Cluster0015
15060231	XM_003364322 // LOC100629230 // protein BTG1-like // --- // 100629230 /// ENSECAT000000	LOC100629230	Cluster0015
14982656	ENSECAT00000010964 // LOC100629956 // uncharacterized LOC100629956 // --- // 100629956	LOC100629956	Cluster0015
15046132	NR_033010 // MIR329 // microRNA mir-329 // --- // 100314901	MIR329	Cluster0015
15109357	ENSECAT00000025715 // MMP1 // matrix metallopeptidase 1 (interstitial collagenase) // -	MMP1	Cluster0015
15071545	ENSECAT00000020434 // PDGFRA // platelet-derived growth factor receptor, alpha polypept	PDGFRA	Cluster0015
15026827	ENSECAT00000015816 // PHACTR1 // phosphatase and actin regulator 1 // --- // 100051555	PHACTR1	Cluster0015
15119273	XM_001489455 // PPP4R1 // protein phosphatase 4, regulatory subunit 1 // --- // 1000551	PPP4R1	Cluster0015
14974966	XM_001489025 // PRKAR1B // protein kinase, cAMP-dependent, regulatory, type I, beta //	PRKAR1B	Cluster0015
15000297	ENSECAT00000008682 // SEMA3F // sema domain, immunoglobulin domain (Ig), short basic do	SEMA3F	Cluster0015
15131031	ENSECAT00000019477 // SH3KBP1 // SH3-domain kinase binding protein 1 // --- // 10005864	SH3KBP1	Cluster0015
15016558	NM_001163971 // SLC2A1 // solute carrier family 2 (facilitated glucose transporter), me	SLC2A1	Cluster0015
15039491	ENSECAT00000006399 // SMOX // spermine oxidase // --- // 100052311 /// XM_001495439 //	SMOX	Cluster0015
15124307	XM_001496042 // TRPS1 // trichorhinophalangeal syndrome I // --- // 100056821 /// ENSEC	TRPS1	Cluster0015
15049266	ENSECAT00000021954 // ZNF618 // zinc finger protein 618 // --- // 100051828	ZNF618	Cluster0015
14925019	---		Cluster0017
14925527	---		Cluster0017
14925923	---		Cluster0017
14926359	---		Cluster0017
14926383	---		Cluster0017
14926553	---		Cluster0017
14926625	---		Cluster0017
14926861	---		Cluster0017
14927043	---		Cluster0017
14927189	---		Cluster0017
14927221	---		Cluster0017
14927235	---		Cluster0017
14927239	---		Cluster0017
14927243	---		Cluster0017
14927851	---		Cluster0017
14928035	---		Cluster0017
14928051	---		Cluster0017
14928239	---		Cluster0017
14928337	---		Cluster0017
14928425	---		Cluster0017
14928787	---		Cluster0017

14928841	---	Cluster0017
14929249	---	Cluster0017
14929303	---	Cluster0017
14929327	---	Cluster0017
14929365	---	Cluster0017
14929393	---	Cluster0017
14929403	---	Cluster0017
14929803	---	Cluster0017
14930451	---	Cluster0017
14930985	---	Cluster0017
14931223	---	Cluster0017
14931265	---	Cluster0017
14931293	---	Cluster0017
14931929	---	Cluster0017
14931957	---	Cluster0017
14932229	---	Cluster0017
14951716	---	Cluster0017
14955826	---	Cluster0017
14974089	---	Cluster0017
14978302	---	Cluster0017
14995241	---	Cluster0017
14998291	---	Cluster0017
15011672	---	Cluster0017
15021478	---	Cluster0017
15042666	---	Cluster0017
15079582	---	Cluster0017
15104229	---	Cluster0017
15116922	---	Cluster0017
15117450	---	Cluster0017
15120348	---	Cluster0017
15125810	---	Cluster0017
15134782	---	Cluster0017
15135992	---	Cluster0017
15136220	---	Cluster0017
15136328	---	Cluster0017
15136490	---	Cluster0017
15136506	---	Cluster0017
15136638	---	Cluster0017
15136870	---	Cluster0017

15137082	---		Cluster0017
15137290	---		Cluster0017
15137554	---		Cluster0017
15059443	XM_001500428 // SEPT3 // septin 3 // --- // 100070757 /// ENSECAT00000025376 // SEPT3 /	SEPT3	Cluster0017
14950769	ENSECAT00000022180 // HIF3A // hypoxia inducible factor 3, alpha subunit // --- // ---	HIF3A	Cluster0017
15042278	NM_001099441 // IFNA1 // interferon-alpha-1 // --- // 100053022 /// ENSECAT00000006710	IFNA1	Cluster0017
14988276	XM_001493078 // LOC100051611 // dual specificity protein phosphatase 2-like // --- // 1	LOC100051611	Cluster0017
14960527	ENSECAT00000006708 // LOC100054466 // nuclear receptor subfamily 1 group D member 1-lik	LOC100054466	Cluster0017
15071034	ENSECAT00000016451 // LOC100057498 // uncharacterized protein C4orf26-like // --- // 10	LOC100057498	Cluster0017
15055465	XM_001491141 // LOC100058018 // trefoil factor 3-like // --- // 100058018 /// ENSECAT00	LOC100058018	Cluster0017
15103454	XM_001493821 // LOC100059768 // growth arrest and DNA damage-inducible protein GADD45 b	LOC100059768	Cluster0017
14944560	XM_001496642 // LOC100066341 // pulmonary surfactant-associated protein D-like // --- /	LOC100066341	Cluster0017
14979434	XM_001496643 // LOC100066345 // uncharacterized LOC100066345 // --- // 100066345 /// EN	LOC100066345	Cluster0017
15110507	ENSECAT00000014904 // LOC100072682 // A disintegrin and metalloproteinase with thrombos	LOC100072682	Cluster0017
14939908	ENSECAT00000016954 // LOC100072799 // uncharacterized LOC100072799 // --- // 100072799	LOC100072799	Cluster0017
15128153	ENSECAT00000019935 // LOC100146280 // zinc finger X-linked protein ZXDB-like // --- //	LOC100146280	Cluster0017
15065731	ENSECAT00000021303 // LOC100146434 // forkhead box protein F1-like // --- // 100146434	LOC100146434	Cluster0017
14944421	ENSECAT00000028834 // LOC100629593 // protein FAM25-like // --- // 100629593 /// XM_003	LOC100629593	Cluster0017
15038111	ENSECAT00000024818 // PABPC1L // poly(A) binding protein, cytoplasmic 1-like // --- //	PABPC1L	Cluster0017
14954403	ENSECAT00000010452 // PHLDB3 // pleckstrin homology-like domain, family B, member 3 //	PHLDB3	Cluster0017
15094022	XM_003365130 // PNKD // paroxysmal nonkinesigenic dyskinesia // --- // 100055676 /// EN	PNKD	Cluster0017
15064119	ENSECAT00000012436 // SLC6A2 // solute carrier family 6 (neurotransmitter transporter,	SLC6A2	Cluster0017
15079841	ENSECAT00000017296 // ZNF775 // zinc finger protein 775 // --- // --- /// ENSECAT000000	ZNF775	Cluster0017
14926283	---		Cluster0025
14927677	---		Cluster0025
14927693	---		Cluster0025
14927823	---		Cluster0025
14928059	---		Cluster0025
14928101	---		Cluster0025
14928255	---		Cluster0025
14929341	---		Cluster0025
14931611	---		Cluster0025
14938479	---		Cluster0025
14946671	---		Cluster0025
14951437	---		Cluster0025
14953136	---		Cluster0025
14967562	---		Cluster0025
14983568	---		Cluster0025
14994501	---		Cluster0025

15020815	---		Cluster0025
15021315	---		Cluster0025
15048559	---		Cluster0025
15057681	---		Cluster0025
15090711	---		Cluster0025
15091814	---		Cluster0025
15108333	---		Cluster0025
15111259	---		Cluster0025
15115246	---		Cluster0025
15117474	---		Cluster0025
15121773	---		Cluster0025
15123566	---		Cluster0025
15124138	---		Cluster0025
15132332	---		Cluster0025
15134392	---		Cluster0025
15134788	---		Cluster0025
15135268	---		Cluster0025
15135562	---		Cluster0025
15136204	---		Cluster0025
15137132	---		Cluster0025
15137404	---		Cluster0025
15137616	---		Cluster0025
15137878	---		Cluster0025
15137880	---		Cluster0025
15137884	---		Cluster0025
14941218	ENSECAT00000020695 // DMBT1 // deleted in malignant brain tumors 1 // --- // ---	DMBT1	Cluster0025
15091762	ENSECAT00000025249 // GNRHR2 // gonadotropin-releasing hormone (type 2) receptor 2 // -	GNRHR2	Cluster0025
15079580	XM_001490205 // LOC100056474 // taste receptor type 2 member 134-like // --- // 1000564	LOC100056474	Cluster0025
14955433	ENSECAT00000026777 // LOC100066668 // myeloid cell surface antigen CD33-like // --- //	LOC100066668	Cluster0025
15110298	ENSECAT00000022178 // LOC100072289 // roundabout homolog 4-like // --- // 100072289	LOC100072289	Cluster0025
15062888	ENSECAT00000004118 // LOC100146765 // olfactory receptor 2T33-like // --- // 100146765	LOC100146765	Cluster0025
14939921	ENSECAT00000019167 // LOC100630099 // uncharacterized LOC100630099 // --- // 100630099	LOC100630099	Cluster0025
15091066	NR_032839 // MIR1905B // microRNA mir-1905b // --- // 100315113	MIR1905B	Cluster0025
15027400	ENSECAT00000025255 // TRIM40 // tripartite motif containing 40 // --- // ---	TRIM40	Cluster0025
14952029	ENSECAT00000020392 // ZNF671 // zinc finger protein 671 // --- // ---	ZNF671	Cluster0025
