# Supplementary material for: Comparative transcriptome analysis of equine alveolar macrophages
Source: Equine Vet J. 2016 Jul 9;49(3):375–82. doi: 10.1111/evj.12584 (PMC5412682; doi:10.1111/evj.12584)
Supplement: Supplementary file 4 [file EVJ-49-375-s004.pdf]

Equine Veterinary Journal

Supplementary Item 1d: Genelist of 5279 transcripts differentially expressed between AMs and PMs - with p adjusted value <0.05 and fold change >2

Transcript ID	Gene_assignment	Gene_Symbol	p-value	Fold-Change
15121321	ENSECAT00000013628 // LOC100052888 // cholesterol 7-alpha-monooxygenase-like // --- //	LOC100052888	<0.05	49.5489
15080122	XM_001487907 // LOC100049847 // platelet glycoprotein 4-like // --- // 100049847 /// EN	LOC100049847	<0.05	38.3266
15100171	ENSECAT00000020379 // CD163 // CD163 molecule // --- // ---	CD163	<0.05	28.0566
15077957	ENSECAT00000001905 // GPNMB // glycoprotein (transmembrane) nmb // --- // 100067870 ///	GPNMB	<0.05	27.0716
15066795	XM_001916447 // LOC100057831 // n-acylethanolamine-hydrolyzing acid amidase-like // ---	LOC100057831	<0.05	25.7024
14972893	NM_001257155 // MPEG1 // macrophage expressed 1 // --- // 100066669 /// ENSECAT00000001	MPEG1	<0.05	24.8713
15088442	ENSECAT00000011655 // MCOLN2 // mucolipin 2 // --- // 100052719 /// XM_001496833 // MCO	MCOLN2	<0.05	21.8081
15126979	NM_001111301 // TLR8 // toll-like receptor 8 // --- // 100054367 /// ENSECAT00000009327	TLR8	<0.05	20.9228
14934085	XM_001502962 // LIPA // lipase A, lysosomal acid, cholesterol esterase // --- // 100071	LIPA	<0.05	18.7798
14966871	---		<0.05	17.1072
15134514	---		<0.05	16.2329
15040529	ENSECAT00000021279 // PLTP // phospholipid transfer protein // --- // 100056507 /// XM_	PLTP	<0.05	15.2925
15051976	ENSECAT00000005057 // PTGR1 // prostaglandin reductase 1 // --- // ---	PTGR1	<0.05	14.2633
15023455	XM_001501495 // LOC100071667 // complement C1q subcomponent subunit B-like // --- // 10	LOC100071667	<0.05	14.0384
14958998	ENSECAT00000007910 // ABCA6 // ATP-binding cassette, sub-family A (ABC1), member 6 // -	ABCA6	<0.05	13.7175
15098294	XM_001494130 // LOC100052143 // lysozyme C-like // --- // 100052143 /// ENSECAT00000019	LOC100052143	<0.05	13.1031
14933958	ENSECAT00000020082 // MYOF // myoferlin // --- // ---	MYOF	<0.05	11.7761
15100625	ENSECAT00000015675 // PLBD1 // phospholipase B domain containing 1 // --- // 100066968	PLBD1	<0.05	11.7085
15106728	XM_001917218 // PRCP // prolylcarboxypeptidase (angiotensinase C) // --- // 100061830 /	PRCP	<0.05	11.5189
15063383	XM_001497471 // MRC1 // mannose receptor, C type 1 // --- // 100068247 /// ENSECAT00000	MRC1	<0.05	11.1187
15033029	XM_001491667 // CD180 // CD180 molecule // --- // 100056598 /// ENSECAT00000018322 // C	CD180	<0.05	11.0961
14967730			<0.05	11.0516
15053235	ENSECAT00000000831 // LOC100069029 // ficolin-1-like // --- // 100069029 /// XM_0014988	LOC100069029	<0.05	11.0335
14943095	ENSECAT00000011152 // EGR2 // early growth response 2 // --- // 100062890 /// XM_001503	EGR2	<0.05	10.7066
15023462	XM_001504261 // LOC100058097 // complement C1q subcomponent subunit A-like // --- // 10	LOC100058097	<0.05	10.5162
15089727	XM_001489794 // LOC100055750 // retinoic acid receptor responder protein 1-like // ---	LOC100055750	<0.05	10.417
15123100	XM_001489456 // LOC100057538 // fatty acid-binding protein, epidermal-like // --- // 10	LOC100057538	<0.05	10.2741
15096737	XM_001490287 // LOC100056613 // heme transporter HRG1-like // --- // 100056613 /// ENSE	LOC100056613	<0.05	10.0751
14969419	XM_001914718 // LOC100060647 // catalase-like // --- // 100060647 /// ENSECAT0000002124	LOC100060647	<0.05	9.51748
14989278			<0.05	9.5015
15015651	XM_001488028 // LOC100049896 // lipid phosphate phosphohydrolase 3-like // --- // 10004	LOC100049896	<0.05	9.48814
15126635			<0.05	9.37799
15023458	XM_001504258 // LOC100058056 // complement C1q subcomponent subunit C-like // --- // 10	LOC100058056	<0.05	9.32528
15031833	ENSECAT00000012639 // ENPP5 // ectonucleotide pyrophosphatase/phosphodiesterase 5 (puta	ENPP5	<0.05	9.27836
14962629	XM_003362509 // LOC100629451 // uncharacterized LOC100629451 // --- // 100629451 /// EN	LOC100629451	<0.05	9.19226
14952073			<0.05	9.12197
14955644	NM_001083593 // ILT11A // immunoglobulin-like transcript 11 A // 10p12 // 100036551 ///	ILT11A	<0.05	9.09651
15044308	XM_001495733 // LOC100050367 // galectin-3-like // --- // 100050367 /// ENSECAT00000006	LOC100050367	<0.05	8.87855
14994472	XM_001502263 // LOC100072348 // PQ-loop repeat-containing protein 3-like // --- // 1000	LOC100072348	<0.05	8.75719
15066585	ENSECAT00000019091 // LOC100051954 // transmembrane protein 150C-like // --- // 1000519	LOC100051954	<0.05	8.75065
14942824	ENSECAT00000010526 // LOC100071611 // interferon-induced protein with tetratricopeptide	LOC100071611	<0.05	8.67969
15029938	ENSECAT00000014629 // MBOAT1 // membrane bound O-acyltransferase domain containing 1 //	MBOAT1	<0.05	8.57184
15082071	XM_001500152 // CPVL // carboxypeptidase, vitellogenic-like // --- // 100069591 /// ENS	CPVL	<0.05	8.56527
14948606	ENSECAT00000005524 // LOC100056635 // egl nine homolog 3-like // --- // 100056635 /// X	LOC100056635	<0.05	8.50285
15057858	XM_001914713 // LOC100058283 // glioma pathogenesis-related protein 1-like // --- // 10	LOC100058283	<0.05	8.45443
14998968	ENSECAT00000017857 // LOC100051258 // peroxisome proliferator-activated receptor gamma-	LOC100051258	<0.05	8.42217
15086910	XM_001490599 // LOC100054991 // cathepsin K-like // --- // 100054991 /// ENSECAT0000002	LOC100054991	<0.05	8.39858
15030619			<0.05	8.29686
15003616	XM_001495367 // KLF5 // Kruppel-like factor 5 (intestinal) // --- // 100052058 /// ENSE	KLF5	<0.05	8.16746
15100712	ENSECAT00000016023 // EPS8 // epidermal growth factor receptor pathway substrate 8 // -	EPS8	<0.05	8.11492
15094354	ENSECAT00000006171 // LOC100056612 // 2-acylglycerol O-acyltransferase 1-like // --- //	LOC100056612	<0.05	8.08387
15004986	---		<0.05	7.98063
15123383	ENSECAT00000023014 // SGK3 // serum/glucocorticoid regulated kinase family, member 3 //	SGK3	<0.05	7.905
14993570	ENSECAT00000013445 // RASGRP3 // RAS guanyl releasing protein 3 (calcium and DAG-regula	RASGRP3	<0.05	7.83859
14972866	ENSECAT00000000688 // LPXN // leupaxin // --- // 100066577 /// XM_001504694 // LPXN //	LPXN	<0.05	7.79321
15073525	XM_001915707 // LOC100050843 // regulator of G-protein signaling 18-like // --- // 1000	LOC100050843	<0.05	7.779
15031855	ENSECAT00000026880 // LOC100055841 // platelet-activating factor acetylhydrolase-like /	LOC100055841	<0.05	7.72283
15090278	ENSECAT00000020548 // LOC100056429 // corticosteroid 11-beta-dehydrogenase isozyme 1-li	LOC100056429	<0.05	7.60787
15042343	XM_001497806 // ACO1 // aconitase 1, soluble // --- // 100067866 /// ENSECAT00000016500	ACO1	<0.05	7.59669
15084195	EU423855 // ATP1B1 // ATPase, Na+/K+ transporting, beta 1 polypeptide // --- // 1000339	ATP1B1	<0.05	7.46007
15053898	ENSECAT00000015326 // IFNGR2 // interferon gamma receptor 2 (interferon gamma transduce	IFNGR2	<0.05	7.40986
15090570	---		<0.05	7.3002
14950926	XM_001488495 // LOC100050794 // epithelial membrane protein 3-like // --- // 100050794	LOC100050794	<0.05	7.27585
15012696	XM_001500027 // LOC100070376 // integrin beta-5-like // --- // 100070376 /// ENSECAT000	LOC100070376	<0.05	7.2531
15124723	ENSECAT00000025889 // LOC100068961 // src-like-adapter-like // --- // 100068961 /// XM_	LOC100068961	<0.05	7.03199
15090308	ENSECAT00000017807 // LOC100057656 // uncharacterized LOC100057656 // --- // 100057656	LOC100057656	<0.05	6.98994
15052391	ENSECAT00000013641 // LOC100071711 // erythrocyte band 7 integral membrane protein-like	LOC100071711	<0.05	6.95172
15029563	ENSECAT00000010506 // SERPINB1 // serpin peptidase inhibitor, clade B (ovalbumin), memb	SERPINB1	<0.05	6.93706
14950576	ENSECAT00000008481 // LOC100065481 // uncharacterized LOC100065481 // --- // 100065481	LOC100065481	<0.05	6.91512
15095766	ENSECAT00000001427 // PTPN6 // protein tyrosine phosphatase, non-receptor type 6 // ---	PTPN6	<0.05	6.90845
15049156	XM_003364096 // LOC100630493 // uncharacterized LOC100630493 // --- // 100630493 /// EN	LOC100630493	<0.05	6.8862
15032447	---		<0.05	6.88201
14960951			<0.05	6.87572
15053950	NM_001247992 // SLC5A3 // solute carrier family 5 (sodium/myo-inositol cotransporter),	SLC5A3	<0.05	6.85803
15005180	ENSECAT00000019998 // LOC100059776 // UPF0124 protein C13orf31-like // --- // 100059776	LOC100059776	<0.05	6.84107
15130005	AY372182 // HPRT1 // hypoxanthine phosphoribosyltransferase 1 // --- // 100034149	HPRT1	<0.05	6.81615
14937987	ENSECAT00000018237 // CCPG1 // cell cycle progression 1 // --- // 100069165 /// XM_0015	CCPG1	<0.05	6.79334
15113074	XM_001504976 // LOC100071946 // mas-related G-protein coupled receptor member X3-like /	LOC100071946	<0.05	6.74716
14969432	ENSECAT00000001357 // LOC100059218 // ETS homologous factor-like // --- // 100059218 //	LOC100059218	<0.05	6.72527
15025388	XM_001500342 // ETFDH // electron-transferring-flavoprotein dehydrogenase // --- // 100	ETFDH	<0.05	6.71192
14935044			<0.05	6.64298
14997495	ENSECAT00000013017 // LOC100056371 // beta-galactosidase-like // --- // 100056371 /// X	LOC100056371	<0.05	6.63372
15025833	ENSECAT00000020853 // LOC100063290 // ras-related protein Rab-33B-like // --- // 100063	LOC100063290	<0.05	6.62488
14967714			<0.05	6.60556
14969059	XM_001503512 // ALDH3A2 // aldehyde dehydrogenase 3 family, member A2 // --- // 1000731	ALDH3A2	<0.05	6.58631
15107920	XM_001501420 // PDE3B // phosphodiesterase 3B, cGMP-inhibited // --- // 100071615 /// E	PDE3B	<0.05	6.58313
14983996	XM_003362887 // LOC100073268 // arylsulfatase B-like // --- // 100073268 /// ENSECAT000	LOC100073268	<0.05	6.43424
14976601	ENSECAT00000010646 // LOC100050012 // glycerophosphodiester phosphodiesterase 1-like //	LOC100050012	<0.05	6.43092
14999470	NM_001163874 // MITF // microphthalmia-associated transcription factor // --- // 100033	MITF	<0.05	6.41019
14994408	---		<0.05	6.40586
14966729	ENSECAT00000024391 // LOC100071384 // carbonic anhydrase 4-like // --- // 100071384 ///	LOC100071384	<0.05	6.40104
15092846	ENSECAT00000010453 // LOC100059248 // protein Dr1-like // --- // 100059248 /// XM_00149	LOC100059248	<0.05	6.39932
15079044	---		<0.05	6.39406
15026316	ENSECAT00000007892 // LOC100629547 // uncharacterized protein C4orf32-like // --- // 10	LOC100629547	<0.05	6.36469
15002648			<0.05	6.35294
14948614	ENSECAT00000004529 // SPTSSA // serine palmitoyltransferase, small subunit A // --- //	SPTSSA	<0.05	6.3039
15012190	XM_001497843 // LOC100067916 // nmrA-like family domain-containing protein 1-like // --	LOC100067916	<0.05	6.30263
14957489	XM_001503227 // ARHGAP18 // Rho GTPase activating protein 18 // --- // 100073070 /// EN	ARHGAP18	<0.05	6.28675
15019145	ENSECAT00000024692 // LOC100061432 // NAD kinase-like // --- // 100061432	LOC100061432	<0.05	6.25351
15033102	ENSECAT00000010936 // SREK1IP1 // SREK1-interacting protein 1 // --- // ---	SREK1IP1	<0.05	6.23632
15088609	XM_001496963 // LOC100066781 // GPI-anchor transamidase-like // --- // 100066781 /// EN	LOC100066781	<0.05	6.1836
14988241			<0.05	6.18252
15066732	XM_001491232 // LOC100058151 // lysosome membrane protein 2-like // --- // 100058151 //	LOC100058151	<0.05	6.16248

15090442	XM_003364970 // LOC100629275 // SLAM family member 9-like // --- // 100629275 /// ENSEC	LOC100629275	<0.05	6.14992
15097754	ENSECAT00000013743 // LRP1 // low density lipoprotein receptor-related protein 1 // ---	LRP1	<0.05	6.0825
15028919			<0.05	6.01945
15060512	ENSECAT00000011449 // GNPTAB // N-acetylglucosamine-1-phosphate transferase, alpha and	GNPTAB	<0.05	6.00849
15094045	ENSECAT00000017571 // SLC11A1 // solute carrier family 11 (proton-coupled divalent meta	SLC11A1	<0.05	5.97642
15104217	ENSECAT00000013638 // LOC100061627 // l-aminoadipate-semialdehyde dehydrogenase-phospho	LOC100061627	<0.05	5.95764
14934104	ENSECAT00000023736 // LOC100071652 // ankyrin repeat domain-containing protein 22-like	LOC100071652	<0.05	5.89193
15028920			<0.05	5.88862
15119610	XM_001492336 // YES1 // v-yes-1 Yamaguchi sarcoma viral oncogene homolog 1 // --- // 10	YES1	<0.05	5.88836
15132189	NR_033090 // MIR421 // microRNA mir-421 // --- // 100314944	MIR421	<0.05	5.81119
15021753	ENSECAT00000000533 // AKR1A1 // aldo-keto reductase family 1, member A1 (aldehyde reduc	AKR1A1	<0.05	5.79087
15133050	XM_003365863 // LOC100058639 // septin-6-like // --- // 100058639 /// XM_003365864 // L	LOC100058639	<0.05	5.77819
15085165	ENSECAT00000008630 // LOC100049921 // protein odr-4 homolog // --- // 100049921	LOC100049921	<0.05	5.75237
15111327			<0.05	5.7375
14939634	XM_001505137 // LOC100058767 // purine nucleoside phosphorylase-like // --- // 10005876	LOC100058767	<0.05	5.72898
14974360	XM_001494157 // LOC100055087 // interferon regulatory factor 7-like // --- // 100055087	LOC100055087	<0.05	5.69449
14961814	ENSECAT00000010739 // LOC100071750 // myosin-Id-like // --- // 100071750 /// XM_0015016	LOC100071750	<0.05	5.69068
15010072	XM_001500017 // LOC100053400 // protein lunapark-like // --- // 100053400 /// ENSECAT00	LOC100053400	<0.05	5.68659
15054013	XM_001493319 // MORC3 // MORC family CW-type zinc finger 3 // --- // 100061343 /// ENSE	MORC3	<0.05	5.6809
15024161			<0.05	5.65047
15052013	ENSECAT00000018972 // SUSD1 // sushi domain containing 1 // --- // 100057493 /// XM_001	SUSD1	<0.05	5.6035
15051765	XM_001491865 // CTNNAL1 // catenin (cadherin-associated protein), alpha-like 1 // --- /	CTNNAL1	<0.05	5.57977
15001122	ENSECAT00000020926 // OXSR1 // oxidative-stress responsive 1 // --- // 100050017 /// XM	OXSR1	<0.05	5.57353
15109765			<0.05	5.53241
14930111	---		<0.05	5.52763
14992663	ENSECAT00000016266 // PCYOX1 // prenylcysteine oxidase 1 // --- // ---	PCYOX1	<0.05	5.52387
14983672	XM_001503689 // ERAP1 // endoplasmic reticulum aminopeptidase 1 // --- // 100073224 ///	ERAP1	<0.05	5.50394
15003200	XM_001488628 // LOC100055495 // chronic lymphocytic leukemia deletion region gene 6 pro	LOC100055495	<0.05	5.49061
15066347	ENSECAT00000008370 // LOC100052823 // estradiol 17-beta-dehydrogenase 11-like // --- //	LOC100052823	<0.05	5.48856
15090577	---		<0.05	5.47315
15020790	ENSECAT00000007596 // MFSD8 // major facilitator superfamily domain containing 8 // ---	MFSD8	<0.05	5.46775
14989918	ENSECAT00000018766 // LOC100069189 // zinc finger protein 36, C3H1 type-like 2-like //	LOC100069189	<0.05	5.46674
15095505	ENSECAT00000020320 // LOC100058416 // probable fructose-2,6-bisphosphatase TIGAR-like /	LOC100058416	<0.05	5.46249
15086940	ENSECAT00000017475 // LOC100054851 // Golgi phosphoprotein 3-like // --- // 100054851 /	LOC100054851	<0.05	5.45962
14991168	ENSECAT00000013227 // CMPK2 // cytidine monophosphate (UMP-CMP) kinase 2, mitochondrial	CMPK2	<0.05	5.45677
15056735			<0.05	5.44913
14960956			<0.05	5.42345
15063605	ENSECAT00000007499 // CELF2 // CUGBP, Elav-like family member 2 // --- // 100056973	CELF2	<0.05	5.40834
15003667	XM_001488399 // IRG1 // immunoresponsive 1 homolog (mouse) // --- // 100052594 /// ENSE	IRG1	<0.05	5.40761
14988260			<0.05	5.38704
15016678	XM_001503336 // LOC100068301 // palmitoyl-protein thioesterase 1-like // --- // 1000683	LOC100068301	<0.05	5.32264
15056472	XM_001492050 // LOC100058620 // RWD domain-containing protein 4-like // --- // 10005862	LOC100058620	<0.05	5.32075
14953042	ENSECAT00000025228 // LOC100072974 // acid sphingomyelinase-like phosphodiesterase 3a-l	LOC100072974	<0.05	5.30617
15100599	XM_001496928 // LOC100066737 // heme-binding protein 1-like // --- // 100066737 /// ENS	LOC100066737	<0.05	5.2957
15089320	XM_001493813 // LOC100051501 // brain protein 44-like // --- // 100051501 /// ENSECAT00	LOC100051501	<0.05	5.29524
15096334	ENSECAT00000019669 // LOC100629790 // LYR motif-containing protein 5-like // --- // 100	LOC100629790	<0.05	5.29195
14985662			<0.05	5.28236
14992482	ENSECAT00000004426 // LOC100049814 // STAM-binding protein-like // --- // 100049814	LOC100049814	<0.05	5.27741
14948730	ENSECAT00000024001 // LOC100050882 // MAP3K12-binding inhibitory protein 1-like // ---	LOC100050882	<0.05	5.25612
14937659	EF397516 // RPS27L // ribosomal protein S27-like // --- // 100067253	RPS27L	<0.05	5.24881
14932809	ENSECAT00000007590 // LOC100067493 // shootin-1-like // --- // 100067493 /// XM_0014947	LOC100067493	<0.05	5.24188
15043389	XM_001917215 // LOC100059240 // GTP:AMP phosphotransferase, mitochondrial-like // --- /	LOC100059240	<0.05	5.22486
14975916	ENSECAT00000009012 // SPN // sialophorin // --- // ---	SPN	<0.05	5.22202
15047937	XM_001497437 // LOC100053265 // legumain-like // --- // 100053265 /// ENSECAT0000001104	LOC100053265	<0.05	5.21611
15133995	ENSECAT00000010186 // MPP1 // membrane protein, palmitoylated 1, 55kDa // --- // 100063	MPP1	<0.05	5.20584
15029644	XM_001489822 // LOC100050826 // neuritin-like // --- // 100050826 /// ENSECAT0000000711	LOC100050826	<0.05	5.19457
15087433	XM_001499911 // LOC100065284 // pre-mRNA-splicing factor SPF27-like // --- // 100065284	LOC100065284	<0.05	5.18828
15121289	XM_001497054 // LOC100066888 // protein FAN-like // --- // 100066888 /// ENSECAT0000000	LOC100066888	<0.05	5.17893
15055602	XM_001490002 // ITGB2 // integrin, beta 2 (complement component 3 receptor 3 and 4 subu	ITGB2	<0.05	5.16771
15035682	---		<0.05	5.13504
15058538	ENSECAT00000020229 // LOC100066907 // cholinephosphotransferase 1-like // --- // 100066	LOC100066907	<0.05	5.128
14952189	ENSECAT00000022431 // SENP6 // SUMO1/sentrin specific peptidase 6 // --- // ---	SENP6	<0.05	5.10071
15028926			<0.05	5.0902
15005444	XM_001488367 // LOC100052476 // uncharacterized LOC100052476 // --- // 100052476 /// EN	LOC100052476	<0.05	5.08504
15066529	XM_003364673 // LOC100630403 // placenta-specific gene 8 protein-like // --- // 1006304	LOC100630403	<0.05	5.08396
14930055	---		<0.05	5.07476
15002728	NM_001163965 // ALOX5AP // arachidonate 5-lipoxygenase-activating protein // --- // 100	ALOX5AP	<0.05	5.05691
15001420	ENSECAT00000016147 // TGFBR2 // transforming growth factor, beta receptor II (70/80kDa)	TGFBR2	<0.05	5.03595
14973811			<0.05	5.02274
15109422	ENSECAT00000009016 // LOC100069290 // caspase-12-like // --- // 100069290 /// XM_001499	LOC100069290	<0.05	5.00107
15036000	ENSECAT00000018036 // FAM105A // family with sequence similarity 105, member A // --- /	FAM105A	<0.05	4.98241
15024167			<0.05	4.98226
15089466	XM_001496519 // LOC100052201 // vesicle-associated membrane protein 4-like // --- // 10	LOC100052201	<0.05	4.97547
15020149	ENSECAT00000014733 // LOC100068165 // transmembrane protein 192-like // --- // 10006816	LOC100068165	<0.05	4.96014
15026919	ENSECAT00000016162 // LOC100066056 // protein FAM8A1-like // --- // 100066056 /// XM_00	LOC100066056	<0.05	4.95747
15106911	ENSECAT00000019345 // ARRB1 // arrestin, beta 1 // --- // 100064657	ARRB1	<0.05	4.93835
14956044	XM_001497890 // LOC100067982 // protein MB21D1-like // --- // 100067982 /// ENSECAT0000	LOC100067982	<0.05	4.92531
15054729	XM_001499808 // LOC100070130 // charged multivesicular body protein 2b-like // --- // 1	LOC100070130	<0.05	4.91391
15069788			<0.05	4.90015
15028925			<0.05	4.89625
15029439	ENSECAT00000025473 // OGFRL1 // opioid growth factor receptor-like 1 // --- // 10007108	OGFRL1	<0.05	4.89482
14952076			<0.05	4.89244
14952967	---		<0.05	4.88996
15017781	XM_001501327 // FUCA1 // fucosidase, alpha-L- 1, tissue // --- // 100071543 /// ENSECAT	FUCA1	<0.05	4.88664
15034327	XM_001491155 // LOC100058040 // solute carrier family 12 member 7-like // --- // 100058	LOC100058040	<0.05	4.87968
15093362	---		<0.05	4.87522
15017011			<0.05	4.8684
14928677	---		<0.05	4.86547
15071639	XM_001494523 // OCIAD1 // OCIA domain containing 1 // --- // 100054246 /// ENSECAT00000	OCIAD1	<0.05	4.85171
14988263			<0.05	4.84195
15090460	XM_001503884 // LOC100066720 // junctional adhesion molecule A-like // --- // 100066720	LOC100066720	<0.05	4.8304
15081257	XM_003364824 // LOC100630906 // transmembrane protein C7orf23 homolog // --- // 1006309	LOC100630906	<0.05	4.82126
15118233	XM_001489039 // LOC100050323 // gatC-like protein-like // --- // 100050323 /// ENSECAT0	LOC100050323	<0.05	4.81435
15060741			<0.05	4.80971
15092213	XM_001498786 // CTTNBP2NL // CTTNBP2 N-terminal like // --- // 100059014 /// ENSECAT000	CTTNBP2NL	<0.05	4.80206
15003673	ENSECAT00000010662 // LOC100052699 // ceroid-lipofuscinosis neuronal protein 5-like //	LOC100052699	<0.05	4.80198
15075859	ENSECAT00000017712 // LOC100055381 // ribonuclease T2-like // --- // 100055381 /// XM_0	LOC100055381	<0.05	4.7975
14956880	XM_001503938 // LOC100066344 // autophagy protein 5-like // --- // 100066344 /// XM_001	LOC100066344	<0.05	4.76593
14954371	---		<0.05	4.76428
15032122	ENSECAT00000018852 // GCLC // glutamate-cysteine ligase, catalytic subunit // --- // 10	GCLC	<0.05	4.76233
15069782			<0.05	4.74841
15106855	XM_001917175 // LOC100063217 // serine/threonine-protein kinase PAK 1-like // --- // 10	LOC100063217	<0.05	4.74717
14994617	ENSECAT00000010440 // LOC100072610 // radical S-adenosyl methionine domain-containing p	LOC100072610	<0.05	4.74414
15133967	XM_001492232 // G6PD // glucose-6-phosphate dehydrogenase // --- // 100059734 /// ENSEC	G6PD	<0.05	4.74122
14988247			<0.05	4.72857
15011355	ENSECAT00000018545 // LOC100068972 // methyltransferase-like protein 21A-like // --- //	LOC100068972	<0.05	4.72629

15017444	---		<0.05	4.72054
15131506	NR_033079 // MIR221 // microRNA mir-221 // --- // 100314997	MIR221	<0.05	4.71276
15125853	NM_001098795 // BSG // basigin (Ok blood group) // --- // 100049616 /// ENSECAT00000012	BSG	<0.05	4.70946
14988240			<0.05	4.70769
15114749	XM_001915547 // LOC100058376 // anaphase-promoting complex subunit 7-like // --- // 100	LOC100058376	<0.05	4.70019
15033431	NM_001135605 // SEPP1 // selenoprotein P, plasma, 1 // --- // 100052968 /// ENSECAT0000	SEPP1	<0.05	4.68513
15009234	ENSECAT00000025356 // LOC100050291 // ADP-ribosylation factor-like protein 5A-like // -	LOC100050291	<0.05	4.66486
15001521	XM_001493724 // KAT2B // K(lysine) acetyltransferase 2B // --- // 100061976 /// ENSECAT	KAT2B	<0.05	4.65606
14998185	XM_001499088 // NCK1 // NCK adaptor protein 1 // --- // 100053397 /// ENSECAT0000000807	NCK1	<0.05	4.64495
15132639	XM_001493218 // BTK // Bruton agammaglobulinemia tyrosine kinase // --- // 100057626 //	BTK	<0.05	4.63386
15090303	ENSECAT00000010143 // LOC100057176 // uncharacterized LOC100057176 // --- // 100057176	LOC100057176	<0.05	4.63054
15133614	ENSECAT00000006990 // ATP11C // ATPase, class VI, type 11C // --- // 100055432 /// XM_0	ATP11C	<0.05	4.63035
14930503	---		<0.05	4.6152
14988232			<0.05	4.60936
15096055	ENSECAT00000008273 // LOC100063778 // activating transcription factor 7-interacting pro	LOC100063778	<0.05	4.59914
15047051	XM_001499937 // LOC100052972 // vesicle transport through interaction with t-SNAREs hom	LOC100052972	<0.05	4.59289
15057345	ENSECAT00000021737 // LOC100054433 // acid ceramidase-like // --- // 100054433 /// XM_0	LOC100054433	<0.05	4.5865
15087730	XM_001917352 // SORT1 // sortilin 1 // --- // 100061385 /// ENSECAT00000016625 // SORT1	SORT1	<0.05	4.58639
14973807			<0.05	4.58003
15061942	---		<0.05	4.5769
15136780	---		<0.05	4.5769
15003021	XM_001496271 // LOC100066289 // ubiquitin-fold modifier 1-like // --- // 100066289 ///	LOC100066289	<0.05	4.57512
14999483	XM_001498624 // LOC100053342 // PRA1 family protein 3-like // --- // 100053342 /// ENSE	LOC100053342	<0.05	4.56939
15076569	XM_001488486 // FGL2 // fibrinogen-like 2 // --- // 100050110 /// ENSECAT00000000351 //	FGL2	<0.05	4.56555
15026446	XM_001503572 // SGMS2 // sphingomyelin synthase 2 // --- // 100072971 /// ENSECAT000000	SGMS2	<0.05	4.56477
15064009	ENSECAT00000012261 // CHD9 // chromodomain helicase DNA binding protein 9 // --- // ---	CHD9	<0.05	4.55663
14993723	ENSECAT00000010851 // LOC100054514 // spastin-like // --- // 100054514	LOC100054514	<0.05	4.54682
15019599	ENSECAT00000015968 // ADAM28 // ADAM metallopeptidase domain 28 // --- // ---	ADAM28	<0.05	4.54141
15049210	ENSECAT00000017029 // LOC100053455 // probable low affinity copper uptake protein 2-lik	LOC100053455	<0.05	4.53478
15029151	NM_001256979 // PAQR8 // progestin and adipoQ receptor family member VIII // --- // 100	PAQR8	<0.05	4.52551
15060744			<0.05	4.51952
15005251	XM_001493104 // LOC100061031 // response gene to complement 32 protein-like // --- // 1	LOC100061031	<0.05	4.51787
15109777			<0.05	4.51753
15111763	ENSECAT00000029021 // LOC100063572 // alkaline ceramidase 3-like // --- // 100063572 //	LOC100063572	<0.05	4.5018
15083149	XM_001496508 // JHDM1D // jumonji C domain containing histone demethylase 1 homolog D (	JHDM1D	<0.05	4.50023
15096123	XM_001497503 // LOC100067460 // putative deoxyribose-phosphate aldolase-like // --- //	LOC100067460	<0.05	4.49022
14967087	ENSECAT00000016351 // ADAP2 // ArfGAP with dual PH domains 2 // --- // 100058484 /// XM	ADAP2	<0.05	4.46803
15011105	ENSECAT00000021236 // LOC100067857 // peptidyl-prolyl cis-trans isomerase-like 3-like /	LOC100067857	<0.05	4.46486
14989638	ENSECAT00000012456 // RTN4 // reticulon 4 // --- // 100066612 /// XM_001496826 // RTN4	RTN4	<0.05	4.44879
14983332	XM_001503307 // LOC100073095 // peptidyl-prolyl cis-trans isomerase C-like // --- // 10	LOC100073095	<0.05	4.4427
15114731	XM_001495080 // LOC100058295 // vacuolar protein sorting-associated protein 29-like //	LOC100058295	<0.05	4.44083
15134394	---		<0.05	4.43048
15091455	ENSECAT00000001614 // LOC100059977 // sorting nexin-27-like // --- // 100059977 /// XM_	LOC100059977	<0.05	4.42695
14960961			<0.05	4.41811
15101941	ENSECAT00000012390 // LIMA1 // LIM domain and actin binding 1 // --- // 100060033	LIMA1	<0.05	4.41018
14988267	ENSECAT00000022652 // STARD7 // StAR-related lipid transfer (START) domain containing 7	STARD7	<0.05	4.40844
15085179	ENSECAT00000026856 // PLA2G4A // phospholipase A2, group IVA (cytosolic, calcium-depend	PLA2G4A	<0.05	4.40689
15047158	NM_001242552 // SYNJ2BP // synaptojanin 2 binding protein // --- // 100049833 /// ENSEC	SYNJ2BP	<0.05	4.4058
15121251	XM_001496350 // ASPH // aspartate beta-hydroxylase // --- // 100065909 /// ENSECAT00000	ASPH	<0.05	4.39055
15101040	XM_001502786 // TM7SF3 // transmembrane 7 superfamily member 3 // --- // 100069046 ///	TM7SF3	<0.05	4.38737
15089172	XM_001490878 // LOC100050772 // ras-related protein Rab-7b-like // --- // 100050772 ///	LOC100050772	<0.05	4.38511
14936651	XM_001492287 // LOC100059835 // pro-cathepsin H-like // --- // 100059835 /// ENSECAT000	LOC100059835	<0.05	4.38256
14927075	---		<0.05	4.36973
14929827	---		<0.05	4.3661
15112741	XM_001504921 // LOC100055900 // zinc finger BED domain-containing protein 5-like // ---	LOC100055900	<0.05	4.3567
15066957	---		<0.05	4.3516
15096663	ENSECAT00000016911 // PPHLN1 // periphilin 1 // --- // 100054208 /// XM_001488998 // PP	PPHLN1	<0.05	4.34733
14983820	XM_001504616 // ARRDC3 // arrestin domain containing 3 // --- // 100065030 /// ENSECAT0	ARRDC3	<0.05	4.32464
14934454	XM_001503642 // LOC100063227 // KIF1-binding protein-like // --- // 100063227 /// ENSEC	LOC100063227	<0.05	4.31682
14992741	ENSECAT00000009486 // LOC100061509 // rho GTPase-activating protein 25-like // --- // 1	LOC100061509	<0.05	4.31362
15118512	XM_001490910 // LOC100057622 // aldehyde dehydrogenase, mitochondrial-like // --- // 10	LOC100057622	<0.05	4.30952
15005437	XM_001487914 // LOC100049821 // COMM domain-containing protein 6-like // --- // 1000498	LOC100049821	<0.05	4.30663
15056490	XM_001492844 // LOC100060636 // N(4)-(beta-N-acetylglucosaminyl)-L-asparaginase-like //	LOC100060636	<0.05	4.30499
15100432	ENSECAT00000011489 // LOC100053863 // c-type lectin domain family 7 member A-like // --	LOC100053863	<0.05	4.30201
14992134	ENSECAT00000022445 // LOC100067047 // pentatricopeptide repeat-containing protein 3, mi	LOC100067047	<0.05	4.30155
14964992	ENSECAT00000006498 // LOC100064249 // allergin-1-like // --- // 100064249 /// XM_001495	LOC100064249	<0.05	4.29419
15057360	ENSECAT00000004098 // LOC100054569 // protein FRG1-like // --- // 100054569 /// XM_0014	LOC100054569	<0.05	4.28786
15130875	XM_003365756 // LOC100629680 // AP-1 complex subunit sigma-2-like // --- // 100629680 /	LOC100629680	<0.05	4.28666
15128849	---		<0.05	4.28623
14952575	XM_001503886 // LOC100066096 // heat shock protein 67B2-like // --- // 100066096 /// EN	LOC100066096	<0.05	4.27921
15015924	ENSECAT00000023091 // ZCCHC11 // zinc finger, CCHC domain containing 11 // --- // 10005	ZCCHC11	<0.05	4.26929
14961285	XM_001503344 // SCPEP1 // serine carboxypeptidase 1 // --- // 100070731 /// ENSECAT0000	SCPEP1	<0.05	4.2649
15060864			<0.05	4.26148
14932159	---		<0.05	4.26039
14978943	XM_001493663 // TPST1 // tyrosylprotein sulfotransferase 1 // --- // 100061873 /// ENSE	TPST1	<0.05	4.2585
14989940	DQ178640 // SLC8A1 // solute carrier family 8 (sodium/calcium exchanger), member 1 // -	SLC8A1	<0.05	4.2548
15008014	ENSECAT00000019755 // SPATS2L // spermatogenesis associated, serine-rich 2-like // ---	SPATS2L	<0.05	4.25212
14928159	---		<0.05	4.24452
14937799	XM_003363670 // LOC100630597 // transcription initiation factor IIA subunit 2-like // -	LOC100630597	<0.05	4.24007
15094386	ENSECAT00000005121 // LOC100061790 // uncharacterized protein KIAA1486-like // --- // 1	LOC100061790	<0.05	4.2297
15077709	ENSECAT00000015810 // LOC100064947 // transmembrane protein 106B-like // --- // 1000649	LOC100064947	<0.05	4.22365
15015111	ENSECAT00000005619 // SLC35A5 // solute carrier family 35, member A5 // --- // 10007147	SLC35A5	<0.05	4.21814
15103195	ENSECAT00000005793 // LOC100051841 // uncharacterized protein C12orf31 homolog // --- /	LOC100051841	<0.05	4.21621
15085680	ENSECAT00000022399 // LOC100058672 // SLAM family member 6-like // --- // 100058672 ///	LOC100058672	<0.05	4.21296
15131218	ENSECAT00000002080 // LOC100050940 // uncharacterized protein CXorf21-like // --- // 10	LOC100050940	<0.05	4.20879
15089287	XM_001915543 // LOC100056817 // protein CREG1-like // --- // 100056817 /// ENSECAT00000	LOC100056817	<0.05	4.20857
14974095	XM_001916968 // UNC93B1 // unc-93 homolog B1 (C. elegans) // --- // 100059773 /// ENSEC	UNC93B1	<0.05	4.20847
14930831	---		<0.05	4.19205
15133293	XM_001915156 // AIFM1 // apoptosis-inducing factor, mitochondrion-associated, 1 // ---	AIFM1	<0.05	4.17765
14952833	XM_001504039 // LOC100072369 // ribosome production factor 2 homolog // --- // 10007236	LOC100072369	<0.05	4.17386
15124597	XM_003365630 // LOC100068561 // protein FAM49B-like // --- // 100068561 /// ENSECAT0000	LOC100068561	<0.05	4.16701
15023541	ENSECAT00000014642 // LOC100058403 // cytidine deaminase-like // --- // 100058403 /// X	LOC100058403	<0.05	4.16233
15013963	ENSECAT00000008912 // TBL1XR1 // transducin (beta)-like 1 X-linked receptor 1 // --- //	TBL1XR1	<0.05	4.1568
15052043	ENSECAT00000008679 // SSBIP1 // SOSS complex subunit C // --- // 100629181	SSBIP1	<0.05	4.15575
14936341	ENSECAT00000009864 // ARRDC4 // arrestin domain containing 4 // --- // 100055139 /// XM	ARRDC4	<0.05	4.15412
14935685	ENSECAT00000008181 // LOC100064139 // glutamate dehydrogenase 1, mitochondrial-like //	LOC100064139	<0.05	4.14506
14928157	---		<0.05	4.14476
15008359	ENSECAT00000018899 // NRP2 // neuropilin 2 // --- // 100066305 /// XM_001505114 // NRP2	NRP2	<0.05	4.12875
15080189	---		<0.05	4.11662
15004780	XM_001495257 // SPG20 // spastic paraplegia 20 (Troyer syndrome) // --- // 100062426 //	SPG20	<0.05	4.11275
15073420	XM_001489827 // LOC100055808 // membrane magnesium transporter 1-like // --- // 1000558	LOC100055808	<0.05	4.10948
14988228			<0.05	4.10856
14942819	XM_003363417 // LOC100630879 // interferon-induced protein with tetratricopeptide repea	LOC100630879	<0.05	4.10788
14991117	XM_001503587 // LOC100057164 // integrin beta-1-binding protein 1-like // --- // 100057	LOC100057164	<0.05	4.08155
14928143	---		<0.05	4.08033
15089804	XM_003364942 // LOC100629233 // uncharacterized LOC100629233 // --- // 100629233 /// EN	LOC100629233	<0.05	4.07762

15007983 ENSECAT00000016494 // LOC100070523 // mps one binder kinase activator-like 3-like // --	LOC100070523	<0.05	4.07287
15026760 XM_001491437 // PAK1IP1 // PAK1 interacting protein 1 // --- // 100051197 /// ENSECAT00	PAK1IP1	<0.05	4.06323
15057871 ENSECAT00000007163 // ZDHHC17 // zinc finger, DHHC-type containing 17 // --- // 1000592	ZDHHC17	<0.05	4.06319
15085595 ---		<0.05	4.0626
15082811 ---		<0.05	4.0623
15136060 ---		<0.05	4.06183
14992694 XM_001490819 // LOC100050657 // annexin A4-like // --- // 100050657 /// ENSECAT00000013	LOC100050657	<0.05	4.04176
14984636 XM_001502804 // CPEB4 // cytoplasmic polyadenylation element binding protein 4 // --- /	CPEB4	<0.05	4.03037
14969557 ENSECAT00000010831 // LOC100050075 // estradiol 17-beta-dehydrogenase 12-like // --- //	LOC100050075	<0.05	4.02541
15036845 ENSECAT00000018434 // LOC100629353 // RING finger protein 24-like // --- // 100629353 /	LOC100629353	<0.05	4.02481
15078126 ENSECAT00000014353 // LOC100630238 // e3 ubiquitin-protein ligase RNF6-like // --- // 1	LOC100630238	<0.05	4.0223
14988619 ENSECAT00000000954 // LOC100052855 // t-cell surface glycoprotein CD8 beta chain-like /	LOC100052855	<0.05	4.0199
14928813 ---		<0.05	4.01549
15021662 XM_001495067 // LOC100051817 // putative methyltransferase NSUN4-like // --- // 1000518	LOC100051817	<0.05	4.01363
15110324 XM_001505075 // LOC100064265 // transmembrane protein 218-like // --- // 100064265 ///	LOC100064265	<0.05	4.00767
14945214 ENSECAT00000024103 // LOC100057905 // TM2 domain-containing protein 3-like // --- // 10	LOC100057905	<0.05	3.99843
15044811 ENSECAT00000019716 // LOC100052428 // protein Churchill-like // --- // 100052428 /// XM	LOC100052428	<0.05	3.98794
15003753 XM_001489318 // LOC100050355 // ubiquitin carboxyl-terminal hydrolase isozyme L3-like /	LOC100050355	<0.05	3.98352
15124754 XM_001499489 // LOC100058071 // probable ribosome biogenesis protein RLP24-like // ---	LOC100058071	<0.05	3.98174
15045260 XM_001490976 // LOC100057010 // rRNA-processing protein FCF1 homolog // --- // 10005701	LOC100057010	<0.05	3.97856
15053657 XM_001498520 // LOC100068696 // multidrug resistance-associated protein 1-like // --- /	LOC100068696	<0.05	3.97747
15078045 XM_003364826 // LOC100068610 // sorting nexin-10-like // --- // 100068610 /// ENSECAT00	LOC100068610	<0.05	3.9696
15123912		<0.05	3.96869
15106580 XM_001488653 // LOC100050120 // cysteine and histidine-rich domain-containing protein 1	LOC100050120	<0.05	3.96737
15062274 ENSECAT00000021501 // LOC100068422 // 3-hydroxyacyl-CoA dehydratase 1-like // --- // 10	LOC100068422	<0.05	3.96669
14985697 ---		<0.05	3.96657
14962163 ---		<0.05	3.96635
15044782 ENSECAT00000018657 // ZBTB1 // zinc finger and BTB domain containing 1 // --- // 100052	ZBTB1	<0.05	3.95834
15012487 ENSECAT00000015076 // TM4SF19 // transmembrane 4 L six family member 19 // --- // 10006	TM4SF19	<0.05	3.95796
15081477 XM_001492902 // LOC100051572 // BET1 homolog // --- // 100051572 /// ENSECAT00000025977	LOC100051572	<0.05	3.95737
14932724 XM_001493616 // LOC100058646 // thioredoxin-dependent peroxide reductase, mitochondrial	LOC100058646	<0.05	3.9545
14944107 XM_001492832 // LOC100050954 // geranylgeranyl pyrophosphate synthase-like // --- // 10	LOC100050954	<0.05	3.95016
15089112 XM_001489449 // LOC100055118 // nuclear ubiquitous casein and cyclin-dependent kinases	LOC100055118	<0.05	3.94219
15021905		<0.05	3.93903
15128598		<0.05	3.93453
15060932 ENSECAT00000024087 // LOC100054701 // ras-related C3 botulinum toxin substrate 2-like /	LOC100054701	<0.05	3.92737
15094026 XM_001491285 // PNKD // paroxysmal nonkinesigenic dyskinesia // --- // 100055676 /// EN	PNKD	<0.05	3.92634
15068182 XM_001499798 // LOC100070117 // wolframin-like // --- // 100070117 /// ENSECAT000000137	LOC100070117	<0.05	3.92553
15102593 ENSECAT00000011406 // LOC100058984 // matrix metalloproteinase-19-like // --- // 100058	LOC100058984	<0.05	3.91911
15068036 ENSECAT00000026208 // FBXL5 // F-box and leucine-rich repeat protein 5 // --- // 100055	FBXL5	<0.05	3.91606
15089958 ENSECAT00000022722 // LOC100052153 // TRMT1-like protein-like // --- // 100052153	LOC100052153	<0.05	3.91561
14966849 ENSECAT00000020639 // HEATR6 // HEAT repeat containing 6 // --- // 100071508 /// XM_001	HEATR6	<0.05	3.91479
15112869		<0.05	3.91313
15053870 ---		<0.05	3.90891
15104450 ENSECAT00000023060 // DLAT // dihydrolipoamide S-acetyltransferase // --- // 100062027	DLAT	<0.05	3.90747
14961469 XM_001503753 // RPS6KB1 // ribosomal protein S6 kinase, 70kDa, polypeptide 1 // --- //	RPS6KB1	<0.05	3.90178
14961850 XM_001501677 // CRLF3 // cytokine receptor-like factor 3 // --- // 100071822 /// ENSECA	CRLF3	<0.05	3.89881
14943242 XM_001502612 // LOC100072614 // graves disease carrier protein-like // --- // 100072614	LOC100072614	<0.05	3.89047
14941662 ENSECAT00000025956 // TCF7L2 // transcription factor 7-like 2 (T-cell specific, HMG-box	TCF7L2	<0.05	3.88782
14953260 ENSECAT00000013992 // ENPP1 // ectonucleotide pyrophosphatase/phosphodiesterase 1 // --	ENPP1	<0.05	3.88614
14943787 ENSECAT00000005161 // LOC100066087 // ras-related protein Rab-4A-like // --- // 1000660	LOC100066087	<0.05	3.88142
15124520 XM_001497477 // LOC100067415 // putative deoxyribonuclease TATDN1-like // --- // 100067	LOC100067415	<0.05	3.876
14939626 ---		<0.05	3.87235
14968427		<0.05	3.86765
15104589 XM_001502187 // LOC100062339 // oligoribonuclease, mitochondrial-like // --- // 1000623	LOC100062339	<0.05	3.86044
15135974 ---		<0.05	3.8596
15130771 ENSECAT00000014823 // LOC100050563 // trafficking protein particle complex subunit 2-li	LOC100050563	<0.05	3.85442
15049184 ENSECAT00000012160 // SNX30 // sorting nexin family member 30 // --- // 100056926 /// X	SNX30	<0.05	3.8497
15057310 XM_001488435 // LOC100050103 // CCR4-NOT transcription complex subunit 7-like // --- //	LOC100050103	<0.05	3.84917
15062697		<0.05	3.84882
15069787		<0.05	3.84608
14942619 XM_001502528 // EXOC6 // exocyst complex component 6 // --- // 100061840 /// ENSECAT000	EXOC6	<0.05	3.84499
15035773 XM_001499617 // SLC1A3 // solute carrier family 1 (glial high affinity glutamate transp	SLC1A3	<0.05	3.84198
14997121 XM_001500901 // LOC100054643 // palmitoyltransferase ZDHHC3-like // --- // 100054643 //	LOC100054643	<0.05	3.83805
14942434 XM_003363448 // CCNJ // cyclin J // --- // 100061183 /// ENSECAT00000016852 // CCNJ //	CCNJ	<0.05	3.83732
14951074 XM_001490969 // LOC100055274 // leukocyte antigen CD37-like // --- // 100055274 /// ENS	LOC100055274	<0.05	3.82184
15026308 XM_001502751 // LOC100072722 // AP-1 complex-associated regulatory protein-like // ---	LOC100072722	<0.05	3.81476
14928135 ---		<0.05	3.81008
15055539 XM_001491140 // LOC100050760 // cystatin-B-like // --- // 100050760 /// ENSECAT00000014	LOC100050760	<0.05	3.80832
15024164		<0.05	3.80654
14945010 ENSECAT00000021485 // AEN // apoptosis enhancing nuclease // --- // ---	AEN	<0.05	3.8037
15009087 ENSECAT00000022776 // LOC100050088 // rho-related GTP-binding protein RhoE-like // ---	LOC100050088	<0.05	3.80256
15098777 XM_001491910 // TUBA4A // tubulin, alpha 4a // --- // 100059249 /// ENSECAT00000015274	TUBA4A	<0.05	3.80253
15048857 ENSECAT00000005562 // ALK5 // TGF beta receptor type I // --- // 100034117 /// XM_00149	ALK5	<0.05	3.79296
15004629 ENSECAT00000011800 // SLC46A3 // solute carrier family 46, member 3 // --- // 100062949	SLC46A3	<0.05	3.7911
14997788 ENSECAT00000017393 // LOC100052302 // DPH3 homolog // --- // 100052302 /// XM_003363153	LOC100052302	<0.05	3.78244
15024962 ENSECAT00000012563 // LOC100060683 // coiled-coil domain-containing protein 25-like //	LOC100060683	<0.05	3.78192
15121244 ---		<0.05	3.78001
14941354 XM_001496265 // INPP5F // inositol polyphosphate-5-phosphatase F // --- // 100065762 //	INPP5F	<0.05	3.77421
15019985 XM_001498470 // FBXO8 // F-box protein 8 // --- // 100061095 /// ENSECAT00000007947 //	FBXO8	<0.05	3.77145
14934940 XM_001503115 // LOC100073005 // 40S ribosomal protein S24-like // --- // 100073005 ///	LOC100073005	<0.05	3.77112
15082649 XM_001502540 // ZNF800 // zinc finger protein 800 // --- // 100056562 /// ENSECAT000000	ZNF800	<0.05	3.76032
14957883 ENSECAT00000017181 // LOC100056149 // uncharacterized protein C17orf62 homolog // --- /	LOC100056149	<0.05	3.75932
14983717 XM_001504609 // LOC100064937 // glutaredoxin-1-like // --- // 100064937 /// ENSECAT0000	LOC100064937	<0.05	3.75916
14933903 XM_001502179 // LOC100061542 // cytochrome P450 2C19-like // --- // 100061542 /// ENSEC	LOC100061542	<0.05	3.75904
15129012 ---		<0.05	3.75801
14933661 ENSECAT00000011077 // LOC100070729 // exosome complex component CSL4-like // --- // 100	LOC100070729	<0.05	3.75747
14988255		<0.05	3.75574
15106008 NM_001246672 // ACP5 // acid phosphatase 5, tartrate resistant // --- // 100056621 ///	ACP5	<0.05	3.75354
15102680 NM_001195515 // CS // citrate synthase // --- // 100052279 /// ENSECAT00000017737 // CS	CS	<0.05	3.75263
14967720		<0.05	3.74739
14961552 XM_001918294 // APPBP2 // amyloid beta precursor protein (cytoplasmic tail) binding pro	APPBP2	<0.05	3.73817
15085655 XM_001504441 // LOC100053868 // CD48 antigen-like // --- // 100053868 /// ENSECAT000000	LOC100053868	<0.05	3.73816
14967732		<0.05	3.73657
15038451 XM_001501211 // LOC100071447 // RING finger protein 114-like // --- // 100071447 /// EN	LOC100071447	<0.05	3.73607
15115760 XM_001915642 // LOC100060329 // abhydrolase domain-containing protein 3-like // --- //	LOC100060329	<0.05	3.72663
15091719 XM_001488893 // VPS45 // vacuolar protein sorting 45 homolog (S. cerevisiae) // --- //	VPS45	<0.05	3.72616
15082794 ENSECAT00000013308 // LOC100071970 // transmembrane protein 209-like // --- // 10007197	LOC100071970	<0.05	3.71343
15073336 ENSECAT00000019881 // LOC100054203 // MOSC domain-containing protein 1, mitochondrial-l	LOC100054203	<0.05	3.71332
14940028 ENSECAT00000003088 // LOC100056825 // abhydrolase domain-containing protein 4-like // -	LOC100056825	<0.05	3.71258
14930799 ---		<0.05	3.71143
14937289 ENSECAT00000013887 // LOC100052900 // alpha- and gamma-adaptin-binding protein p34-like	LOC100052900	<0.05	3.70794
15041841 ENSECAT00000026435 // SMARCA2 // SWI/SNF related, matrix associated, actin dependent re	SMARCA2	<0.05	3.70758
15130603 ENSECAT00000023027 // BRCC3 // BRCA1/BRCA2-containing complex, subunit 3 // --- // 1000	BRCC3	<0.05	3.70705
15131330 XM_001489506 // LOC100055232 // dynein light chain Tctex-type 3-like // --- // 10005523	LOC100055232	<0.05	3.69631
15085717 XM_001491425 // LOC100058470 // peroxisomal biogenesis factor 19-like // --- // 1000584	LOC100058470	<0.05	3.68753

15136914	---		<0.05	3.68743
14928665	---		<0.05	3.68655
14942821	ENSECAT00000010390 // IFIT3 // interferon-induced protein with tetratricopeptide repeat	IFIT3	<0.05	3.68505
15126621			<0.05	3.68229
14960955			<0.05	3.6821
15123220	XM_001492421 // TERF1 // telomeric repeat binding factor (NIMA-interacting) 1 // --- //	TERF1	<0.05	3.67989
15059278	XM_001502319 // LOC100055581 // e3 ubiquitin-protein ligase RBX1-like // --- // 1000555	LOC100055581	<0.05	3.6798
15105668	ENSECAT00000011207 // LOC100064396 // EGF-like module-containing mucin-like hormone rec	LOC100064396	<0.05	3.67961
15100959	ENSECAT00000020767 // LOC100051785 // GTPase KRas-like // --- // 100051785 /// XM_00149	LOC100051785	<0.05	3.6767
14964777	ENSECAT00000024658 // LOC100060986 // protein tweety homolog 2-like // --- // 100060986	LOC100060986	<0.05	3.67495
15045693	XM_001497576 // LOC100053356 // UPF0694 transmembrane protein C14orf109 homolog // ---	LOC100053356	<0.05	3.67395
15091713	ENSECAT00000012027 // LOC100058752 // pleckstrin homology domain-containing family O me	LOC100058752	<0.05	3.6728
15135028	---		<0.05	3.67209
14945692	XM_001917992 // LOC100060833 // electron transfer flavoprotein subunit alpha, mitochond	LOC100060833	<0.05	3.67046
15128658	XM_001493372 // OGT // O-linked N-acetylglucosamine (GlcNAc) transferase // --- // 1000	OGT	<0.05	3.65722
14962198	XM_001504257 // ABR // active BCR-related // --- // 100059804 /// ENSECAT00000023892 //	ABR	<0.05	3.65164
15052072	ENSECAT00000019055 // FKBP15 // FK506 binding protein 15, 133kDa // --- // 100053357 //	FKBP15	<0.05	3.65059
14932187	---		<0.05	3.65059
14985695	XM_001504164 // LOC100061903 // probable UDP-sugar transporter protein SLC35A4-like //	LOC100061903	<0.05	3.64844
15090318	ENSECAT00000023700 // LOC100629269 // c4b-binding protein alpha chain-like // --- // 10	LOC100629269	<0.05	3.64563
14935922	ENSECAT00000022627 // LOC100053244 // signal peptidase complex catalytic subunit SEC11A	LOC100053244	<0.05	3.64172
14960957			<0.05	3.637
14934438	ENSECAT00000011504 // DDX21 // DEAD (Asp-Glu-Ala-Asp) box helicase 21 // --- // 1000726	DDX21	<0.05	3.63357
15121027	XM_001493900 // LOC100062272 // beta-lactamase-like protein 2-like // --- // 100062272	LOC100062272	<0.05	3.63273
15118670			<0.05	3.63254
15050324	XM_001499340 // ASS1 // argininosuccinate synthase 1 // --- // 100069776 /// ENSECAT000	ASS1	<0.05	3.63118
15089884	XM_001490231 // NCF2 // neutrophil cytosolic factor 2 // --- // 100051787 /// ENSECAT00	NCF2	<0.05	3.63064
15018160	XM_001501765 // LOC100071914 // aflatoxin B1 aldehyde reductase member 2-like // --- //	LOC100071914	<0.05	3.63038
15004990	ENSECAT00000015762 // PHF11 // PHD finger protein 11 // --- // ---	PHF11	<0.05	3.63029
14929807	---		<0.05	3.62174
15035177	XM_001492811 // PPWD1 // peptidylprolyl isomerase domain and WD repeat containing 1 //	PPWD1	<0.05	3.62169
15092219	ENSECAT00000014445 // DDX20 // DEAD (Asp-Glu-Ala-Asp) box polypeptide 20 // --- // 1000	DDX20	<0.05	3.62153
15130030	XM_001494693 // ZNF75D // zinc finger protein 75D // --- // 100063485 /// ENSECAT000000	ZNF75D	<0.05	3.61866
15027429	ENSECAT00000025949 // LOC100056231 // patr class I histocompatibility antigen, A-126 al	LOC100056231	<0.05	3.61366
15057734	XM_001916797 // LOC100065372 // protein CLN8-like // --- // 100065372 /// ENSECAT000000	LOC100065372	<0.05	3.61245
15101327	ENSECAT00000011938 // GXYLT1 // glucoside xylosyltransferase 1 // --- // 100054009 ///	GXYLT1	<0.05	3.6114
14942766	ENSECAT00000023581 // LOC100062076 // ribonuclease P protein subunit p30-like // --- //	LOC100062076	<0.05	3.61092
15057441	NM_001081798 // TLR3 // toll-like receptor 3 // --- // 100009703 /// ENSECAT00000000204	TLR3	<0.05	3.60992
15003160	ENSECAT00000014628 // INTS6 // integrator complex subunit 6 // --- // 100053346 /// XM_	INTS6	<0.05	3.60845
15126968	XM_001489046 // LOC100054319 // ribose-phosphate pyrophosphokinase 2-like // --- // 100	LOC100054319	<0.05	3.60294
14925169	---		<0.05	3.59702
15050086	ENSECAT00000016032 // LOC100147452 // phytanoyl-CoA dioxygenase domain-containing prote	LOC100147452	<0.05	3.58626
15029718	XM_001490955 // LOC100051123 // adenosine 3'-phospho 5'-phosphosulfate transporter 2-li	LOC100051123	<0.05	3.58512
15120866	XM_001490771 // LOC100057425 // fatty acid-binding protein, adipocyte-like // --- // 10	LOC100057425	<0.05	3.58283
14995422	XM_001495004 // SUCLG2 // succinate-CoA ligase, GDP-forming, beta subunit // --- // 100	SUCLG2	<0.05	3.58161
14932099	---		<0.05	3.57982
15062811	ENSECAT00000006430 // LOC100630762 // signal peptidase complex subunit 1-like // --- //	LOC100630762	<0.05	3.57732
15042863	XM_001488771 // LOC100053674 // UPF0553 protein C9orf64 homolog // --- // 100053674 ///	LOC100053674	<0.05	3.57716
15102031	ENSECAT00000017151 // GALNT6 // UDP-N-acetyl-alpha-D-galactosamine:polypeptide N-acetyl	GALNT6	<0.05	3.57692
15107665	XM_001917959 // IPO7 // importin 7 // --- // 100055677 /// ENSECAT00000017394 // IPO7 /	IPO7	<0.05	3.57657
15098299	XM_001492223 // LOC100059724 // YEATS domain-containing protein 4-like // --- // 100059	LOC100059724	<0.05	3.5732
15022277	ENSECAT00000008084 // ZMPSTE24 // zinc metallopeptidase STE24 homolog (S. cerevisiae) /	ZMPSTE24	<0.05	3.56854
14987636	ENSECAT00000003247 // NAIP // NLR family, apoptosis inhibitory protein // --- // 100073	NAIP	<0.05	3.56829
15042971	ENSECAT00000022743 // VPS13A // vacuolar protein sorting 13 homolog A (S. cerevisiae) /	VPS13A	<0.05	3.56564
14932147	---		<0.05	3.56438
14986817	ENSECAT00000026323 // LOC100073223 // endoplasmic reticulum aminopeptidase 2-like // --	LOC100073223	<0.05	3.55615
15089720	ENSECAT00000015126 // LOC100050024 // latexin-like // --- // 100050024 /// XM_001489649	LOC100050024	<0.05	3.553
15111874	ENSECAT00000013561 // POLD3 // polymerase (DNA-directed), delta 3, accessory subunit //	POLD3	<0.05	3.55035
14961884	XM_001504035 // LOC100058650 // protein EVI2B-like // --- // 100058650 /// ENSECAT00000	LOC100058650	<0.05	3.54974
14964298	ENSECAT00000023421 // SYNGR2 // synaptogyrin 2 // --- // ---	SYNGR2	<0.05	3.54859
15111341			<0.05	3.54604
14929967	---		<0.05	3.54582
15129440	ENSECAT00000015766 // NXT2 // nuclear transport factor 2-like export factor 2 // --- //	NXT2	<0.05	3.54506
15071190	XM_001487986 // RUFY3 // RUN and FYVE domain containing 3 // --- // 100049913 /// ENSEC	RUFY3	<0.05	3.5447
15001499	XM_001492478 // NR1D2 // nuclear receptor subfamily 1, group D, member 2 // --- // 1000	NR1D2	<0.05	3.54179
15024166			<0.05	3.53954
14997384	XM_001489140 // LRRFIP2 // leucine rich repeat (in FLII) interacting protein 2 // --- /	LRRFIP2	<0.05	3.53518
15123201	ENSECAT00000000005 // LOC100146309 // transmembrane protein 70, mitochondrial-like // -	LOC100146309	<0.05	3.53282
15066772	ENSECAT00000017448 // SDAD1 // SDA1 domain containing 1 // --- // 100057877 /// XM_0014	SDAD1	<0.05	3.53259
15081288	ENSECAT00000009979 // LOC100050248 // solute carrier family 25 member 40-like // --- //	LOC100050248	<0.05	3.52645
15118305	ENSECAT00000026473 // LOC100051008 // phosphatidylethanolamine-binding protein 1-like /	LOC100051008	<0.05	3.5258
15046692	XM_003364080 // LOC100630267 // protein cornichon homolog // --- // 100630267 /// ENSEC	LOC100630267	<0.05	3.523
14995999	ENSECAT00000004449 // PBRM1 // polybromo 1 // --- // 100051687	PBRM1	<0.05	3.52136
15046904	ENSECAT00000000056 // TRMT5 // tRNA methyltransferase 5 homolog (S. cerevisiae) // ---	TRMT5	<0.05	3.52034
15117903	XM_001496058 // ADRBK2 // adrenergic, beta, receptor kinase 2 // --- // 100065495 /// E	ADRBK2	<0.05	3.51911
15038910	---		<0.05	3.51827
14976906	ENSECAT00000019016 // LITAF // lipopolysaccharide-induced TNF factor // --- // 10003392	LITAF	<0.05	3.51364
14939112	XM_001503436 // LOC100071164 // complex I intermediate-associated protein 30, mitochond	LOC100071164	<0.05	3.50662
15078973	---		<0.05	3.50042
14956133	ENSECAT00000014850 // TMEM30A // transmembrane protein 30A // --- // ---	TMEM30A	<0.05	3.49746
14959667	XM_001495148 // LOC100064169 // dephospho-CoA kinase domain-containing protein-like //	LOC100064169	<0.05	3.49331
14961882	XM_003362422 // LOC100630810 // protein EVI2A-like // --- // 100630810 /// ENSECAT00000	LOC100630810	<0.05	3.49284
14978980	ENSECAT00000026699 // LOC100061188 // protein NipSnap homolog 2-like // --- // 10006118	LOC100061188	<0.05	3.49177
15118546	ENSECAT00000007051 // LOC100057885 // SH2B adapter protein 3-like // --- // 100057885 /	LOC100057885	<0.05	3.49155
15041503	XM_001488638 // LOC100053331 // uncharacterized LOC100053331 // --- // 100053331 /// EN	LOC100053331	<0.05	3.49049
14952819	XM_001502273 // LOC100072355 // s-adenosylmethionine decarboxylase proenzyme-like // --	LOC100072355	<0.05	3.48522
14989582	ENSECAT00000007818 // CCDC88A // coiled-coil domain containing 88A // --- // ---	CCDC88A	<0.05	3.48409
15029016	ENSECAT00000009130 // ENPP4 // ectonucleotide pyrophosphatase/phosphodiesterase 4 (puta	ENPP4	<0.05	3.48283
14996695	XM_001498943 // LOC100053786 // cytochrome b-c1 complex subunit 1, mitochondrial-like /	LOC100053786	<0.05	3.48247
14996446	NM_001166479 // GPX1 // glutathione peroxidase 1 // --- // 100053396 /// ENSECAT0000001	GPX1	<0.05	3.47784
15089532	---		<0.05	3.47735
15004541	ENSECAT00000014237 // RNF6 // ring finger protein (C3H2C3 type) 6 // --- // 100050743 /	RNF6	<0.05	3.47637
15060855			<0.05	3.47606
15000947			<0.05	3.46986
14988137	XM_001491463 // ACTR1B // ARP1 actin-related protein 1 homolog B, centractin beta (yeas	ACTR1B	<0.05	3.46984
14989276			<0.05	3.46665
15099120	ENSECAT00000019696 // SLC16A14 // solute carrier family 16, member 14 (monocarboxylic a	SLC16A14	<0.05	3.46638
15022934	ENSECAT00000024759 // LOC100070667 // YTH domain family protein 2-like // --- // 100070	LOC100070667	<0.05	3.46622
14953331	ENSECAT00000020831 // LOC100067729 // TATA box-binding protein-like protein 1-like // -	LOC100067729	<0.05	3.46333
14937668	ENSECAT00000019241 // LOC100067582 // vacuolar protein sorting-associated protein 13C-l	LOC100067582	<0.05	3.45844
14947323	ENSECAT00000020958 // LOC100056957 // calcium-binding protein p22-like // --- // 100056	LOC100056957	<0.05	3.45809
14928149	---		<0.05	3.45665
14992055	XM_001497468 // RPIA // ribose 5-phosphate isomerase A // --- // 100052643 /// ENSECAT0	RPIA	<0.05	3.45454
15043751	NM_001257143 // KLHL9 // kelch-like 9 (Drosophila) // --- // 100064784 /// ENSECAT00000	KLHL9	<0.05	3.45444
15131576	XM_001492729 // LOC100051459 // protein UXT-like // --- // 100051459 /// ENSECAT0000001	LOC100051459	<0.05	3.45438
14936613	XM_003363641 // NIPA2 // non imprinted in Prader-Willi/Angelman syndrome 2 // --- // 10	NIPA2	<0.05	3.44958

15021553	XM_001491520 // OSBPL9 // oxysterol binding protein-like 9 // --- // 100051050 /// ENSE	OSBPL9	<0.05	3.44836
15047056	ENSECAT00000022194 // LOC100063295 // retinol dehydrogenase 11-like // --- // 100063295	LOC100063295	<0.05	3.44749
15043978	XM_001499560 // DCAF12 // DDB1 and CUL4 associated factor 12 // --- // 100068462 /// EN	DCAF12	<0.05	3.44122
14965688	XM_003362552 // ATP6V0A1 // ATPase, H+ transporting, lysosomal V0 subunit a1 // --- //	ATP6V0A1	<0.05	3.43884
14985680			<0.05	3.43752
14983652	ENSECAT00000019853 // RIOK2 // RIO kinase 2 (yeast) // --- // 100064864 /// XM_00150460	RIOK2	<0.05	3.43433
14944874	EF442070 // ANPEP // alanyl (membrane) aminopeptidase // --- // 100050397	ANPEP	<0.05	3.4326
15129146	ENSECAT00000001525 // LOC100057749 // armadillo repeat-containing X-linked protein 3-li	LOC100057749	<0.05	3.42915
15036711	XM_001495797 // LOC100065101 // thioredoxin-related transmembrane protein 4-like // ---	LOC100065101	<0.05	3.42701
15002158	XM_001492309 // LOC100051043 // phospholipid scramblase 2-like // --- // 100051043 ///	LOC100051043	<0.05	3.42646
14956167	XM_003362360 // LOC100068991 // CDGSH iron-sulfur domain-containing protein 1-like // -	LOC100068991	<0.05	3.42553
15120215			<0.05	3.42173
15081895	XM_001916123 // LOC100053610 // hyccin-like // --- // 100053610 /// ENSECAT00000025313	LOC100053610	<0.05	3.42108
15117681	ENSECAT00000021479 // SEC14L2 // SEC14-like 2 (S. cerevisiae) // --- // 100058707 /// X	SEC14L2	<0.05	3.42021
15025836	XM_001502496 // NAA15 // N(alpha)-acetyltransferase 15, NatA auxiliary subunit // --- /	NAA15	<0.05	3.41962
15127079	XM_001490349 // LOC100051095 // carbonic anhydrase 5B, mitochondrial-like // --- // 100	LOC100051095	<0.05	3.41936
15010507	ENSECAT00000011599 // CWC22 // CWC22 spliceosome-associated protein homolog (S. cerevis	CWC22	<0.05	3.41876
15059641	ENSECAT00000014407 // TTC38 // tetratricopeptide repeat domain 38 // --- // 100053315 /	TTC38	<0.05	3.41658
14991570	ENSECAT00000012398 // LOC100059155 // UPF0760 protein C2orf29-like // --- // 100059155	LOC100059155	<0.05	3.41645
14951686	---		<0.05	3.41336
15086952	XM_001489164 // LOC100054581 // gamma-secretase subunit APH-1A-like // --- // 100054581	LOC100054581	<0.05	3.41293
14988244			<0.05	3.41247
15087365	XM_001500218 // LOC100059510 // suppressor of IKBKE 1-like // --- // 100059510 /// ENSE	LOC100059510	<0.05	3.41085
15075599	ENSECAT00000017954 // FBXO30 // F-box protein 30 // --- // 100066297 /// XM_001502353 /	FBXO30	<0.05	3.40589
15016778	ENSECAT00000012199 // LOC100054519 // four and a half LIM domains protein 3-like // ---	LOC100054519	<0.05	3.40444
15134298	---		<0.05	3.39964
15060745			<0.05	3.39898
15061511	ENSECAT00000024700 // LOC100051710 // uncharacterized protein KIAA0930-like // --- // 1	LOC100051710	<0.05	3.39646
15110348	XM_001505092 // PUS3 // pseudouridylate synthase 3 // --- // 100064416 /// ENSECAT00000	PUS3	<0.05	3.39215
14967060	ENSECAT00000010249 // RHOT1 // ras homolog family member T1 // --- // ---	RHOT1	<0.05	3.38817
14998480	ENSECAT00000025072 // RNF13 // ring finger protein 13 // --- // 100058094 /// XM_001491	RNF13	<0.05	3.3851
14962675			<0.05	3.38461
14970893	ENSECAT00000024544 // LOC100051106 // calpain-1 catalytic subunit-like // --- // 100051	LOC100051106	<0.05	3.38391
14937077	ENSECAT00000021871 // ADPGK // ADP-dependent glucokinase // --- // 100052166 /// XM_001	ADPGK	<0.05	3.38199
15047557	XM_001491996 // SPTLC2 // serine palmitoyltransferase, long chain base subunit 2 // ---	SPTLC2	<0.05	3.37997
14979763	ENSECAT00000024745 // EEF2K // eukaryotic elongation factor-2 kinase // --- // 10005838	EEF2K	<0.05	3.37781
15025108	XM_001496538 // LOC100054913 // PIN2/TERF1-interacting telomerase inhibitor 1-like // -	LOC100054913	<0.05	3.3718
15014054	XM_001496004 // LOC100058535 // mitochondrial import inner membrane translocase subunit	LOC100058535	<0.05	3.37082
14990202	ENSECAT00000010650 // FEZ2 // fasciculation and elongation protein zeta 2 (zygin II) //	FEZ2	<0.05	3.36866
14952516	XM_001503820 // MANEA // mannosidase, endo-alpha // --- // 100065922 /// ENSECAT0000001	MANEA	<0.05	3.3669
15066675	XM_001491801 // CNOT6L // CCR4-NOT transcription complex, subunit 6-like // --- // 1000	CNOT6L	<0.05	3.36479
14932611	---		<0.05	3.36114
14935474	XM_001494039 // NCOA4 // nuclear receptor coactivator 4 // --- // 100062485 /// ENSECAT	NCOA4	<0.05	3.35612
14994203	ENSECAT00000008355 // HADHB // hydroxyacyl-CoA dehydrogenase/3-ketoacyl-CoA thiolase/en	HADHB	<0.05	3.35493
15005093	XM_003363226 // LRCH1 // leucine-rich repeats and calponin homology (CH) domain contain	LRCH1	<0.05	3.35459
14991926	ENSECAT00000007536 // ANAPC1 // anaphase promoting complex subunit 1 // --- // 10005186	ANAPC1	<0.05	3.35371
15069786			<0.05	3.35231
14985080	XM_001917441 // LOC100059963 // CCR4-NOT transcription complex subunit 8-like // --- //	LOC100059963	<0.05	3.35179
14997257	---		<0.05	3.35007
15135654	---		<0.05	3.34669
15084893	ENSECAT00000000944 // LOC100051658 // n-acetylneuraminate lyase-like // --- // 10005165	LOC100051658	<0.05	3.34642
15063527	ENSECAT00000008224 // LOC100056689 // optineurin-like // --- // 100056689	LOC100056689	<0.05	3.34451
15057113	ENSECAT00000017536 // LOC100058148 // protein MAK16 homolog // --- // 100058148 /// XM_	LOC100058148	<0.05	3.34393
14928171	---		<0.05	3.34031
14929589	---		<0.05	3.33865
15077269	---		<0.05	3.33677
15058669	XM_001497703 // LOC100067735 // host cell factor 2-like // --- // 100067735 /// ENSECAT	LOC100067735	<0.05	3.33597
14948827	ENSECAT00000019341 // LOC100063728 // peptidyl-prolyl cis-trans isomerase FKBP3-like //	LOC100063728	<0.05	3.3331
15104243	ENSECAT00000025920 // LOC100061694 // ras-related protein Rab-39A-like // --- // 100061	LOC100061694	<0.05	3.332
14994882	ENSECAT00000014207 // LOC100055795 // sodium- and chloride-dependent taurine transporte	LOC100055795	<0.05	3.33139
14949221	XM_003362325 // LOC100630820 // CCAAT/enhancer-binding protein gamma-like // --- // 100	LOC100630820	<0.05	3.329
15063887	ENSECAT00000024215 // LONP2 // lon peptidase 2, peroxisomal // --- // 100057497 /// XM_	LONP2	<0.05	3.32891
14956950	XM_001503983 // LOC100066515 // sorting nexin-3-like // --- // 100066515 /// ENSECAT000	LOC100066515	<0.05	3.32806
15024160	ENSECAT00000008644 // TARDBP // TAR DNA binding protein // --- // 100051482	TARDBP	<0.05	3.32745
15006508	XM_001489470 // RAB3GAP1 // RAB3 GTPase activating protein subunit 1 (catalytic) // ---	RAB3GAP1	<0.05	3.32721
15067708			<0.05	3.32528
15057051	ENSECAT00000009280 // LOC100059780 // proline synthase co-transcribed bacterial homolog	LOC100059780	<0.05	3.32365
15106847	ENSECAT00000015914 // LOC100063117 // methylosome subunit pICln-like // --- // 10006311	LOC100063117	<0.05	3.32266
15109126	---		<0.05	3.32252
15130624	ENSECAT00000002112 // LOC100062203 // prefoldin subunit 3-like // --- // 100062203 ///	LOC100062203	<0.05	3.32059
15120912	ENSECAT00000022041 // LOC100059131 // interleukin-7-like // --- // 100059131 /// XM_001	LOC100059131	<0.05	3.32045
14995628	ENSECAT00000006325 // PDHB // pyruvate dehydrogenase (lipoamide) beta // --- // 1000505	PDHB	<0.05	3.31555
15109769			<0.05	3.31533
14928137	---		<0.05	3.31524
15008657	XM_001489408 // LOC100055077 // translin-like // --- // 100055077 /// ENSECAT0000001680	LOC100055077	<0.05	3.31439
15126341	ENSECAT00000013625 // LOC100066807 // protein FAM168B-like // --- // 100066807 /// ENSE	LOC100066807	<0.05	3.3141
14928661	---		<0.05	3.31176
15114785	ENSECAT00000023915 // LOC100059653 // e3 ubiquitin-protein ligase RNF34-like // --- //	LOC100059653	<0.05	3.31113
14966650	XM_001503741 // LOC100071096 // peptidyl-tRNA hydrolase 2, mitochondrial-like // --- //	LOC100071096	<0.05	3.30074
14959280			<0.05	3.29868
14983275	XM_003362879 // LOC100630603 // glutaredoxin-like protein YDR286C homolog // --- // 100	LOC100630603	<0.05	3.29838
15133409	XM_001489008 // LOC100054364 // ras-related protein Rap-2c-like // --- // 100054364 ///	LOC100054364	<0.05	3.29594
15088800	ENSECAT00000014687 // LOC100630574 // guanine nucleotide-binding protein G(I)/G(S)/G(O)	LOC100630574	<0.05	3.2931
15115080	XM_001915444 // ATP6V0A2 // ATPase, H+ transporting, lysosomal V0 subunit a2 // --- //	ATP6V0A2	<0.05	3.28719
14984926	ENSECAT00000020093 // LOC100059534 // cyclin-G1-like // --- // 100059534 /// XM_0015033	LOC100059534	<0.05	3.28543
15058549	ENSECAT00000009020 // LOC100067057 // DNA damage-regulated autophagy modulator protein	LOC100067057	<0.05	3.28436
14956830	XM_001501736 // HACE1 // HECT domain and ankyrin repeat containing E3 ubiquitin protein	HACE1	<0.05	3.28352
14925079	---		<0.05	3.27757
15109249	XM_001499359 // CCDC82 // coiled-coil domain containing 82 // --- // 100061101 /// ENSE	CCDC82	<0.05	3.27753
15013165	XM_001502989 // LOC100061410 // ubiquitin-like-conjugating enzyme ATG3-like // --- // 1	LOC100061410	<0.05	3.27751
14958766	XM_001492365 // LOC100059961 // hematological and neurological expressed 1 protein-like	LOC100059961	<0.05	3.27718
15122733	XM_001496011 // GRINA // glutamate receptor, ionotropic, N-methyl D-aspartate-associate	GRINA	<0.05	3.27663
15056741			<0.05	3.27567
15081535	ENSECAT00000008745 // PDK4 // pyruvate dehydrogenase kinase, isozyme 4 // --- // 100052	PDK4	<0.05	3.27475
15078035	XM_001498349 // NFE2L3 // nuclear factor (erythroid-derived 2)-like 3 // --- // 1000685	NFE2L3	<0.05	3.27367
15087776	ENSECAT00000008255 // WDR47 // WD repeat domain 47 // --- // 100061070 /// XM_001493773	WDR47	<0.05	3.27351
14988227			<0.05	3.27267
15045187	XM_001489871 // LOC100056126 // acyl-coenzyme A thioesterase 6-like // --- // 100056126	LOC100056126	<0.05	3.26896
14925175	---		<0.05	3.26669
15085258	XM_003364961 // INTS7 // integrator complex subunit 7 // --- // 100051001 /// XM_001489	INTS7	<0.05	3.25982
15121207	XM_001495470 // LOC100052510 // armadillo repeat-containing protein 1-like // --- // 10	LOC100052510	<0.05	3.25898
14976695	ENSECAT00000020527 // LOC100630410 // 40S ribosomal protein S15a-like // --- // 1006304	LOC100630410	<0.05	3.25694
14932105	---		<0.05	3.25632
15095973	ENSECAT00000015525 // LOC100053911 // gamma-aminobutyric acid receptor-associated prote	LOC100053911	<0.05	3.25526
15129450	XM_001489834 // LOC100054407 // transmembrane protein 164-like // --- // 100054407 ///	LOC100054407	<0.05	3.25353
15061338	XM_001503005 // LOC100071003 // NFAT activation molecule 1-like // --- // 100071003 ///	LOC100071003	<0.05	3.25343

14925171	---		<0.05	3.25258
15077498	ENSECAT00000024807 // CCDC132 // coiled-coil domain containing 132 // --- // 100051289	CCDC132	<0.05	3.25169
14935417	ENSECAT00000020934 // TTC13 // tetratricopeptide repeat domain 13 // --- // 100061024	TTC13	<0.05	3.24693
15071516	XM_001916943 // LOC100059582 // probable polyprenol reductase-like // --- // 100059582	LOC100059582	<0.05	3.2437
15119372	ENSECAT00000019060 // LOC100630847 // uncharacterized LOC100630847 // --- // 100630847	LOC100630847	<0.05	3.24257
15125594	XM_001493761 // LOC100062035 // pre-rRNA-processing protein TSR2 homolog // --- // 1000	LOC100062035	<0.05	3.23547
14972114	XM_001492591 // LOC100060270 // rhombotin-2-like // --- // 100060270 /// ENSECAT0000000	LOC100060270	<0.05	3.23434
15008138	ENSECAT00000019389 // STRADB // STE20-related kinase adaptor beta // --- // 100066574 /	STRADB	<0.05	3.23324
14940797	XM_001916196 // LOC100066874 // thioredoxin-related transmembrane protein 1-like // ---	LOC100066874	<0.05	3.23249
14979519	XM_001493576 // LOC100061739 // leucine carboxyl methyltransferase 1-like // --- // 100	LOC100061739	<0.05	3.22788
15088569	XM_001498433 // USP33 // ubiquitin specific peptidase 33 // --- // 100053171 /// ENSECA	USP33	<0.05	3.22769
15120610	ENSECAT00000026742 // TMX3 // thioredoxin-related transmembrane protein 3 // --- // 100	TMX3	<0.05	3.22615
14956056	XM_001498010 // LOC100068130 // sialin-like // --- // 100068130 /// ENSECAT00000016980	LOC100068130	<0.05	3.22494
15052116	XM_001488664 // LOC100052763 // delta-aminolevulinic acid dehydratase-like // --- // 10	LOC100052763	<0.05	3.22173
15100100	XM_001497952 // LPCAT3 // lysophosphatidylcholine acyltransferase 3 // --- // 100053035	LPCAT3	<0.05	3.22082
15111593	ENSECAT00000017377 // EED // embryonic ectoderm development // --- // ---	EED	<0.05	3.22022
15104426	ENSECAT00000013223 // LOC100070452 // uncharacterized protein C11orf52-like // --- // 1	LOC100070452	<0.05	3.21957
15025522	XM_001499727 // LOC100070044 // transmembrane protein 131-like // --- // 100070044 ///	LOC100070044	<0.05	3.21895
14925641	---		<0.05	3.2184
14940465	ENSECAT00000021183 // AKAP6 // A kinase (PRKA) anchor protein 6 // --- // 100056405 ///	AKAP6	<0.05	3.21711
15114775	XM_001492153 // P2RX4 // purinergic receptor P2X, ligand-gated ion channel, 4 // --- //	P2RX4	<0.05	3.21434
14932083	---		<0.05	3.21252
15015997	XM_003364373 // LOC100062254 // thioredoxin domain-containing protein 12-like // --- //	LOC100062254	<0.05	3.21229
14959412	ENSECAT00000015048 // CCDC47 // coiled-coil domain containing 47 // --- // 100054134 //	CCDC47	<0.05	3.21216
15014937	ENSECAT00000025756 // LOC100070997 // Protein ADP-ribosylarginine hydrolase-like // ---	LOC100070997	<0.05	3.21068
14944694	XM_001502380 // LOC100053089 // protein FAM103A1-like // --- // 100053089 /// ENSECAT00	LOC100053089	<0.05	3.20977
15104851	XM_001503069 // LOC100071306 // trafficking protein particle complex subunit 4-like //	LOC100071306	<0.05	3.20816
15048752	ENSECAT00000019048 // LOC100064733 // tudor domain-containing protein 7-like // --- //	LOC100064733	<0.05	3.20571
15048243	XM_001491443 // LOC100058497 // cyclin-dependent kinase 2-interacting protein-like // -	LOC100058497	<0.05	3.20252
15024669	---		<0.05	3.20145
15070805	XM_001493012 // LOC100060891 // enolase-phosphatase E1-like // --- // 100060891 /// ENS	LOC100060891	<0.05	3.20021
14966801	ENSECAT00000009030 // SYNRG // synergin, gamma // --- // --- /// ENSECAT00000009061 //	SYNRG	<0.05	3.19934
15067996	XM_001498618 // TAPT1 // transmembrane anterior posterior transformation 1 // --- // 10	TAPT1	<0.05	3.19858
15066609	XM_001915658 // RASGEF1B // RasGEF domain family, member 1B // --- // 100146797 /// ENS	RASGEF1B	<0.05	3.19696
15041680	ENSECAT00000021328 // LOC100054931 // retinal dehydrogenase 1-like // --- // 100054931	LOC100054931	<0.05	3.19448
14941856	XM_003363424 // LOC100069640 // glutathione S-transferase omega-1-like // --- // 100069	LOC100069640	<0.05	3.19387
14938340	ENSECAT00000026695 // COPS2 // COP9 constitutive photomorphogenic homolog subunit 2 (Ar	COPS2	<0.05	3.19351
14957594	XM_001503335 // LOC100073104 // syntaxin-7-like // --- // 100073104 /// ENSECAT00000021	LOC100073104	<0.05	3.19104
15111925	ENSECAT00000023582 // LOC100065518 // 39S ribosomal protein L48, mitochondrial-like //	LOC100065518	<0.05	3.18888
15075976	ENSECAT00000017774 // LOC100051773 // programmed cell death protein 2-like // --- // 10	LOC100051773	<0.05	3.18669
14984245	XM_001495023 // LOC100061017 // histone H2A type 3-like // --- // 100061017 /// ENSECAT	LOC100061017	<0.05	3.18642
15076100	XM_001501744 // LOC100063142 // UPF0364 protein C6orf211 homolog // --- // 100063142 //	LOC100063142	<0.05	3.18571
15125783	---		<0.05	3.18119
14960960			<0.05	3.17936
15123439	ENSECAT00000019642 // LOC100064104 // mitochondrial fission regulator 1-like // --- //	LOC100064104	<0.05	3.17611
14997215	---		<0.05	3.17544
15129707	ENSECAT00000020216 // UBE2A // ubiquitin-conjugating enzyme E2A // --- // 100058679 ///	UBE2A	<0.05	3.17327
15028238	ENSECAT00000015735 // MAPK14 // mitogen-activated protein kinase 14 // --- // 100063532	MAPK14	<0.05	3.16767
15122111	ENSECAT00000024127 // OXR1 // oxidation resistance 1 // --- // 100063414 /// XM_0014946	OXR1	<0.05	3.16704
14971836	XM_001489918 // LOC100055991 // transaldolase-like // --- // 100055991 /// ENSECAT00000	LOC100055991	<0.05	3.1646
15109768			<0.05	3.16348
15044246	---		<0.05	3.16286
15086598			<0.05	3.15827
15098217	XM_001491339 // CAND1 // cullin-associated and neddylation-dissociated 1 // --- // 1000	CAND1	<0.05	3.15815
14964870	ENSECAT00000011973 // AMZ2 // archaelysin family metallopeptidase 2 // --- // 100062918	AMZ2	<0.05	3.1576
15044389	ENSECAT00000020367 // LOC100058966 // actin-related protein 10-like // --- // 100058966	LOC100058966	<0.05	3.15423
14975641	ENSECAT00000022482 // GTF2I // general transcription factor IIi // --- // --- /// ENSEC	GTF2I	<0.05	3.15259
14989791	ENSECAT00000000364 // LOC100068298 // multiple coagulation factor deficiency protein 2-	LOC100068298	<0.05	3.15177
15086960	XM_001917235 // LOC100054493 // acidic leucine-rich nuclear phosphoprotein 32 family me	LOC100054493	<0.05	3.14756
14952904	XM_001504125 // LOC100067001 // RWD domain-containing protein 1-like // --- // 10006700	LOC100067001	<0.05	3.14442
14968426			<0.05	3.14361
14942992	ENSECAT00000008137 // IPMK // inositol polyphosphate multikinase // --- // 100072153 //	IPMK	<0.05	3.14249
14936362	XM_001488965 // LOC100050003 // leucine-rich repeat-containing protein 28-like // --- /	LOC100050003	<0.05	3.14155
15127004	XM_003365792 // LOC100050487 // ras-related protein Rab-9A-like // --- // 100050487 ///	LOC100050487	<0.05	3.14003
15029269	ENSECAT00000009618 // LOC100069975 // uncharacterized protein KIAA1586-like // --- // 1	LOC100069975	<0.05	3.13987
15106967	ENSECAT00000007395 // LOC100065199 // potassium voltage-gated channel subfamily E membe	LOC100065199	<0.05	3.13801
15012029	NM_001163871 // EIF4G1 // eukaryotic translation initiation factor 4 gamma, 1 // --- //	EIF4G1	<0.05	3.1376
15115573	XM_001488009 // LOC100054712 // twisted gastrulation protein homolog 1-like // --- // 1	LOC100054712	<0.05	3.13716
14959996	XM_001493268 // LOC100052353 // coiled-coil domain-containing protein 56-like // --- //	LOC100052353	<0.05	3.13712
14930469	---		<0.05	3.13616
15101070	XM_001502934 // LOC100064789 // endoplasmic reticulum-Golgi intermediate compartment pr	LOC100064789	<0.05	3.13589
14984972	NR_032937 // MIR146A // microRNA mir-146a // --- // 100314861	MIR146A	<0.05	3.13569
14986785	ENSECAT00000012224 // LOC100073217 // membrane protein FAM174A-like // --- // 100073217	LOC100073217	<0.05	3.13529
15070378	XM_001496615 // LOC100066296 // methionine aminopeptidase 1-like // --- // 100066296 //	LOC100066296	<0.05	3.13422
15037460	XM_001499007 // LOC100069227 // charged multivesicular body protein 4b-like // --- // 1	LOC100069227	<0.05	3.13224
14976292	ENSECAT00000018524 // ARHGAP17 // Rho GTPase activating protein 17 // --- // 100069347	ARHGAP17	<0.05	3.13165
15096294	XM_001917026 // LOC100064384 // n-acylneuraminate cytidylyltransferase-like // --- // 1	LOC100064384	<0.05	3.13138
15084669	XM_001487852 // LOC100050551 // sterol O-acyltransferase 1-like // --- // 100050551 ///	LOC100050551	<0.05	3.12977
14975942	XM_001915497 // LOC100146790 // myc-associated zinc finger protein-like // --- // 10014	LOC100146790	<0.05	3.12872
15116149	XM_001495353 // LOC100052335 // e3 ubiquitin-protein ligase RNF125-like // --- // 10005	LOC100052335	<0.05	3.12696
15065262	ENSECAT00000013402 // VAC14 // Vac14 homolog (S. cerevisiae) // --- // 100054657 /// XM	VAC14	<0.05	3.12655
15094978	XM_001500288 // LOC100057575 // NEDD8-conjugating enzyme UBE2F-like // --- // 100057575	LOC100057575	<0.05	3.12598
15086535	ENSECAT00000022095 // LOC100062274 // zinc transporter ZIP1-like // --- // 100062274 //	LOC100062274	<0.05	3.12561
15060863			<0.05	3.12541
15122860	---		<0.05	3.12372
15126625			<0.05	3.12287
14930123	---		<0.05	3.11929
15046321	ENSECAT00000012690 // RCOR1 // REST corepressor 1 // --- // 100058618 /// XM_001491522	RCOR1	<0.05	3.11876
14966919	ENSECAT00000018440 // SLFN5 // schlafen family member 5 // --- // 100071635	SLFN5	<0.05	3.11681
15105472	ENSECAT00000012827 // LOC100064503 // toll/interleukin-1 receptor domain-containing ada	LOC100064503	<0.05	3.11604
15020354	XM_003364538 // LOC100629231 // uncharacterized LOC100629231 // --- // 100629231 /// EN	LOC100629231	<0.05	3.11597
15093686	XM_001499799 // ALG6 // asparagine-linked glycosylation 6, alpha-1,3-glucosyltransferas	ALG6	<0.05	3.11567
15100256	ENSECAT00000023553 // LOC100061559 // cation-dependent mannose-6-phosphate receptor-lik	LOC100061559	<0.05	3.11303
15100413	XM_003365170 // LOC100629907 // c-type lectin domain family 2 member B-like // --- // 1	LOC100629907	<0.05	3.11073
15020302	XM_001500980 // PLRG1 // pleiotropic regulator 1 // --- // 100062153 /// ENSECAT0000001	PLRG1	<0.05	3.10896
15043040	ENSECAT00000009859 // LOC100146171 // forkhead box protein B2-like // --- // 100146171	LOC100146171	<0.05	3.10893
15124608	---		<0.05	3.10482
14984491	---		<0.05	3.10377
15118657			<0.05	3.10332
14925245	---		<0.05	3.10173
15129618	ENSECAT00000022507 // DOCK11 // dedicator of cytokinesis 11 // --- // 100055441 /// ENS	DOCK11	<0.05	3.10019
14925173	---		<0.05	3.09756
14928675	---		<0.05	3.09619
14950873	NM_001170422 // SEPW1 // selenoprotein W, 1 // --- // 100050195 /// ENSECAT00000006991	SEPW1	<0.05	3.09326
15108140	ENSECAT00000021450 // LOC100057340 // oxidoreductase HTATIP2-like // --- // 100057340 /	LOC100057340	<0.05	3.09241
15122687	XM_001496550 // LOC100066193 // lymphocyte antigen 6E-like // --- // 100066193 /// ENSE	LOC100066193	<0.05	3.09141

14932103	---		<0.05	3.08957
14995401	XM_003363051 // LOC100053493 // uncharacterized glycosyltransferase AER61-like // --- /	LOC100053493	<0.05	3.08952
14964348	XM_001492545 // SEC14L1 // SEC14-like 1 (S. cerevisiae) // --- // 100050884 /// ENSECAT	SEC14L1	<0.05	3.08879
15127351	ENSECAT00000027015 // LOC100052103 // zinc finger X-chromosomal protein-like // --- //	LOC100052103	<0.05	3.08412
15093351	ENSECAT00000021689 // LOC100067295 // geranylgeranyl transferase type-2 subunit beta-li	LOC100067295	<0.05	3.08328
15017381	XM_001916895 // PUM1 // pumilio homolog 1 (Drosophila) // --- // 100070514 /// ENSECAT0	PUM1	<0.05	3.08182
14932085	---		<0.05	3.08159
14960958			<0.05	3.07834
15072226	XM_001498491 // LAP3 // leucine aminopeptidase 3 // --- // 100068668 /// ENSECAT0000001	LAP3	<0.05	3.07705
14950270	ENSECAT00000012479 // AXL // AXL receptor tyrosine kinase // --- // 100064907 /// ENSEC	AXL	<0.05	3.07523
15076797	XM_001492195 // LOC100051143 // MLN64 N-terminal domain homolog // --- // 100051143 ///	LOC100051143	<0.05	3.07355
15049327	XM_001487977 // LOC100049971 // transmembrane protein C9orf91-like // --- // 100049971	LOC100049971	<0.05	3.07217
15124610	ENSECAT00000017553 // ASAP1 // ArfGAP with SH3 domain, ankyrin repeat and PH domain 1 /	ASAP1	<0.05	3.07134
15007258	ENSECAT00000025338 // DCAF17 // DDB1 and CUL4 associated factor 17 // --- // 100063621	DCAF17	<0.05	3.07011
14932055	---		<0.05	3.06957
15076970	ENSECAT00000026848 // ZMIZ2 // zinc finger, MIZ-type containing 2 // --- // 100051958 /	ZMIZ2	<0.05	3.06936
15126938	XM_001488963 // MSL3 // male-specific lethal 3 homolog (Drosophila) // --- // 100054131	MSL3	<0.05	3.06788
15019882	ENSECAT00000001509 // LOC100061773 // peptide methionine sulfoxide reductase-like // --	LOC100061773	<0.05	3.06606
15119478	ENSECAT00000019913 // LOC100050857 // DNA endonuclease RBBP8-like // --- // 100050857 /	LOC100050857	<0.05	3.06489
14929813	---		<0.05	3.06485
15100889	ENSECAT00000022411 // LOC100068403 // uncharacterized protein KIAA0528-like // --- // 1	LOC100068403	<0.05	3.06434
15000906	ENSECAT00000007477 // CTNNB1 // catenin (cadherin-associated protein), beta 1, 88kDa //	CTNNB1	<0.05	3.06404
15051612	ENSECAT00000002358 // LOC100629329 // 39S ribosomal protein L50, mitochondrial-like //	LOC100629329	<0.05	3.06294
14990571	XM_001502783 // HADHA // hydroxyacyl-CoA dehydrogenase/3-ketoacyl-CoA thiolase/enoyl-Co	HADHA	<0.05	3.06087
15116587	ENSECAT00000007500 // WDR7 // WD repeat domain 7 // --- // 100049995 /// ENSECAT0000001	WDR7	<0.05	3.06053
15076539	XM_001491173 // PION // pigeon homolog (Drosophila) // --- // 100058064 /// ENSECAT0000	PION	<0.05	3.0603
14965582	XM_001500289 // LOC100061423 // proteasome activator complex subunit 3-like // --- // 1	LOC100061423	<0.05	3.05897
14965995			<0.05	3.05855
15120950	ENSECAT00000000223 // STAU2 // staufen, RNA binding protein, homolog 2 (Drosophila) //	STAU2	<0.05	3.05682
15131212	ENSECAT00000017005 // OSBPL9 // oxysterol binding protein-like 9 // --- // 100051050 //	OSBPL9	<0.05	3.05477
14995548	XM_001488333 // LOC100050081 // BTB/POZ domain-containing protein KCTD20-like // --- //	LOC100050081	<0.05	3.05345
15071822	XM_003364726 // LOC100054984 // rho-related GTP-binding protein RhoH-like // --- // 100	LOC100054984	<0.05	3.05341
15115022	XM_001492985 // LOC100060853 // n-lysine methyltransferase SETD8-like // --- // 1000608	LOC100060853	<0.05	3.05316
15067826	ENSECAT00000002179 // LOC100072076 // 60S ribosomal protein L23a-like // --- // 1000720	LOC100072076	<0.05	3.05254
15072090	XM_001497457 // ANAPC4 // anaphase promoting complex subunit 4 // --- // 100067388 ///	ANAPC4	<0.05	3.05225
15023385	XM_001501350 // LOC100071559 // acyl-protein thioesterase 2-like // --- // 100071559 //	LOC100071559	<0.05	3.05186
14980106	XM_001488886 // LOC100053937 // protein N-terminal asparagine amidohydrolase-like // --	LOC100053937	<0.05	3.05081
15065478	ENSECAT00000012349 // ATMIN // ATM interactor // --- // 100069801 /// XM_001501932 // A	ATMIN	<0.05	3.04478
15070337	ENSECAT00000018313 // LOC100053857 // dual adapter for phosphotyrosine and 3-phosphotyr	LOC100053857	<0.05	3.0438
15060388	XM_001494765 // LTA4H // leukotriene A4 hydrolase // --- // 100052257 /// ENSECAT000000	LTA4H	<0.05	3.04357
14960880	XM_001502181 // CALCOCO2 // calcium binding and coiled-coil domain 2 // --- // 10005579	CALCOCO2	<0.05	3.03689
14957301	ENSECAT00000009844 // LOC100072790 // golgi-associated PDZ and coiled-coil motif-contai	LOC100072790	<0.05	3.036
15121383	ENSECAT00000017567 // LOC100146256 // acyl-protein thioesterase 1-like // --- // 100146	LOC100146256	<0.05	3.0355
15035030	XM_001491166 // LOC100050028 // 28S ribosomal protein S36, mitochondrial-like // --- //	LOC100050028	<0.05	3.0352
14939190	ENSECAT00000026484 // LOC100071368 // uncharacterized protein C15orf57-like // --- // 1	LOC100071368	<0.05	3.03474
14973012	XM_001916178 // SLC15A3 // solute carrier family 15, member 3 // --- // 100062045 /// E	SLC15A3	<0.05	3.03437
14942763	ENSECAT00000017042 // LOC100062658 // polycomb group RING finger protein 5-like // ---	LOC100062658	<0.05	3.03375
15115872	---		<0.05	3.03341
15082896	ENSECAT00000013045 // SLC35B4 // solute carrier family 35, member B4 // --- // 10006518	SLC35B4	<0.05	3.03262
15056451	---		<0.05	3.03237
15052048	ENSECAT00000019674 // LOC100056842 // thymic stromal cotransporter homolog // --- // 10	LOC100056842	<0.05	3.03114
15052925	ENSECAT00000020276 // LOC100069900 // uncharacterized protein C9orf78-like // --- // 10	LOC100069900	<0.05	3.02791
15031437	ENSECAT00000020970 // LOC100065189 // lactoylglutathione lyase-like // --- // 100065189	LOC100065189	<0.05	3.0275
14929891	---		<0.05	3.02713
15043902	ENSECAT00000019100 // LOC100068266 // BAG family molecular chaperone regulator 1-like /	LOC100068266	<0.05	3.02274
15038166	XM_001503260 // PIGT // phosphatidylinositol glycan anchor biosynthesis, class T // ---	PIGT	<0.05	3.02241
15120833	ENSECAT00000024056 // LOC100057100 // sorting nexin-16-like // --- // 100057100 /// XM_	LOC100057100	<0.05	3.02121
15076537	XM_001488320 // LOC100049981 // transmembrane protein 60-like // --- // 100049981 /// E	LOC100049981	<0.05	3.02055
15090490	ENSECAT00000012392 // ARHGAP30 // Rho GTPase activating protein 30 // --- // 100066336	ARHGAP30	<0.05	3.02022
15016244	ENSECAT00000002623 // MKNK1 // MAP kinase interacting serine/threonine kinase 1 // ---	MKNK1	<0.05	3.01872
14985667			<0.05	3.0178
14925243	---		<0.05	3.01482
14988402	ENSECAT00000015896 // TMEM87B // transmembrane protein 87B // --- // 100064755 /// XM_0	TMEM87B	<0.05	3.01409
15086865	ENSECAT00000009525 // LOC100055075 // protein FAM63A-like // --- // 100055075 /// XM_00	LOC100055075	<0.05	3.0137
14936098	XM_001502860 // LOC100053632 // calcium and integrin-binding protein 1-like // --- // 1	LOC100053632	<0.05	3.0132
14965554			<0.05	3.01299
15127262	ENSECAT00000018780 // LOC100051857 // spermine synthase-like // --- // 100051857 /// XM	LOC100051857	<0.05	3.01254
15024070	XM_001491331 // LOC100050902 // procollagen-lysine,2-oxoglutarate 5-dioxygenase 1-like	LOC100050902	<0.05	3.01193
15008748	XM_001487877 // DBI // diazepam binding inhibitor (GABA receptor modulator, acyl-CoA bi	DBI	<0.05	3.00882
14933520	ENSECAT00000014816 // COX15 // COX15 homolog, cytochrome c oxidase assembly protein (ye	COX15	<0.05	3.00776
14988041	ENSECAT00000004169 // LOC100050502 // thioredoxin domain-containing protein 9-like // -	LOC100050502	<0.05	3.00717
15080114	XM_001497336 // LOC100067238 // dnaJ homolog subfamily B member 6-like // --- // 100067	LOC100067238	<0.05	3.00635
14929825	---		<0.05	3.00344
15114791	ENSECAT00000013459 // LOC100059726 // calcium release-activated calcium channel protein	LOC100059726	<0.05	3.00337
15128347	NR_033081 // MIR223 // microRNA mir-223 // --- // 100315091	MIR223	<0.05	3.00247
15057426	ENSECAT00000022544 // LOC100056739 // protein FAM149A-like // --- // 100056739 /// XM_0	LOC100056739	<0.05	3.00166
15025584	XM_003364540 // ARFIP1 // ADP-ribosylation factor interacting protein 1 // --- // 10062	ARFIP1	<0.05	3.00125
14968998	ENSECAT00000005911 // TRPV2 // transient receptor potential cation channel, subfamily V	TRPV2	<0.05	3.001
15100973	ENSECAT00000000404 // LOC100068969 // uncharacterized LOC100068969 // --- // 100068969	LOC100068969	<0.05	2.99819
15004715	---		<0.05	2.99819
15044550	XM_001497650 // LOC100051348 // protein phosphatase 1A-like // --- // 100051348 /// XM_	LOC100051348	<0.05	2.99165
15033111	XM_001493893 // LOC100059778 // probable dimethyladenosine transferase-like // --- // 1	LOC100059778	<0.05	2.99129
15011940	XM_001497146 // AP2M1 // adaptor-related protein complex 2, mu 1 subunit // --- // 1000	AP2M1	<0.05	2.99029
15047422	ENSECAT00000019965 // NEK9 // NIMA (never in mitosis gene a)- related kinase 9 // --- /	NEK9	<0.05	2.98831
14929375	---		<0.05	2.98769
15069785			<0.05	2.98666
15127861	ENSECAT00000025939 // LOC100061212 // serine/threonine-protein kinase A-Raf-like // ---	LOC100061212	<0.05	2.98645
14959197	XM_001500037 // NOL11 // nucleolar protein 11 // --- // 100063762 /// ENSECAT0000000879	NOL11	<0.05	2.98638
15100438	XM_001493960 // LOC100062370 // oxidized low-density lipoprotein receptor 1-like // ---	LOC100062370	<0.05	2.98616
15116496	XM_001499374 // ME2 // malic enzyme 2, NAD(+)-dependent, mitochondrial // --- // 100069	ME2	<0.05	2.98567
15032646	XM_003363819 // LOC100630400 // DET1- and DDB1-associated protein 1-like // --- // 1006	LOC100630400	<0.05	2.98477
14932043	---		<0.05	2.98304
15093724	ENSECAT00000011612 // USP1 // ubiquitin specific peptidase 1 // --- // 100070397 /// XM	USP1	<0.05	2.98266
15093532	ENSECAT00000000659 // LOC100069321 // tctex1 domain-containing protein 1-like // --- //	LOC100069321	<0.05	2.9821
14932163	---		<0.05	2.98171
14947140	ENSECAT00000009600 // LOC100056719 // synaptosomal-associated protein 23-like // --- //	LOC100056719	<0.05	2.98137
15074258	XM_003364554 // LOC100629313 // protein cornichon homolog 4-like // --- // 100629313 //	LOC100629313	<0.05	2.98101
14996788	ENSECAT00000008879 // LOC100063734 // protein shisa-5-like // --- // 100063734 /// XM_0	LOC100063734	<0.05	2.98096
15071419	XM_001492168 // SRP72 // signal recognition particle 72kDa // --- // 100053755 /// ENSE	SRP72	<0.05	2.98027
15027718	ENSECAT00000005079 // C2 // complement component 2 // --- // 100059162 /// XM_001492501	C2	<0.05	2.9796
14945826	XM_001492384 // CSK // c-src tyrosine kinase // --- // 100051462 /// ENSECAT00000014515	CSK	<0.05	2.9764
14987432	ENSECAT00000005789 // LOC100065600 // ribosome biogenesis protein NSA2 homolog // --- /	LOC100065600	<0.05	2.97526
15126680			<0.05	2.97422
15054340	ENSECAT00000023958 // LOC100050911 // pyridoxal kinase-like // --- // 100050911 /// XM_	LOC100050911	<0.05	2.97272
14994375	XM_001502047 // LOC100072175 // ATP-dependent RNA helicase DDX1-like // --- // 10007217	LOC100072175	<0.05	2.97186
14975716	XM_001499997 // LOC100060921 // ribosome maturation protein SBDS-like // --- // 1000609	LOC100060921	<0.05	2.97082

15126737	ENSECAT00000029068 // VPS25 // vacuolar protein sorting 25 homolog (S. cerevisiae) // -	VPS25	<0.05	2.97006
15042661	---		<0.05	2.96889
15012340	ENSECAT00000015618 // CCDC50 // coiled-coil domain containing 50 // --- // ---	CCDC50	<0.05	2.96831
15093366	ENSECAT00000022666 // ACADM // acyl-CoA dehydrogenase, C-4 to C-12 straight chain // --	ACADM	<0.05	2.96826
15013472	XM_001917325 // LOC100072274 // mitochondrial import receptor subunit TOM70-like // ---	LOC100072274	<0.05	2.96802
14925639	---		<0.05	2.96793
15024168			<0.05	2.96622
14939738	XM_001505162 // LOC100059448 // TOX high mobility group box family member 4-like // ---	LOC100059448	<0.05	2.96604
14943342	XM_001503764 // PSAP // prosaposin // --- // 100063520 /// ENSECAT00000023432 // PSAP /	PSAP	<0.05	2.96589
14980277	XM_001492888 // LOC100057523 // phosphomannomutase 2-like // --- // 100057523 /// ENSEC	LOC100057523	<0.05	2.96572
15006122	XM_001504923 // PTPN18 // protein tyrosine phosphatase, non-receptor type 18 (brain-der	PTPN18	<0.05	2.96436
15067377	XM_003364714 // LOC100630825 // OCIA domain-containing protein 2-like // --- // 1006308	LOC100630825	<0.05	2.96194
15064712	XM_001497809 // CTCF // CCCTC-binding factor (zinc finger protein) // --- // 100053363	CTCF	<0.05	2.96101
14936281	XM_001488013 // CHD2 // chromodomain helicase DNA binding protein 2 // --- // 100049803	CHD2	<0.05	2.96077
15126718			<0.05	2.95917
15121755	XM_001491535 // DPY19L4 // dpy-19-like 4 (C. elegans) // --- // 100058629 /// ENSECAT00	DPY19L4	<0.05	2.95811
15071959	ENSECAT00000009746 // LOC100064893 // phosphoglucomutase-2-like // --- // 100064893 ///	LOC100064893	<0.05	2.95574
15065376	XM_001501541 // LOC100055295 // gamma-aminobutyric acid receptor-associated protein-lik	LOC100055295	<0.05	2.95568
15118640	XM_001915599 // LOC100058510 // calcium/calmodulin-dependent protein kinase kinase 2-li	LOC100058510	<0.05	2.95567
15098154	XM_001491955 // LOC100051721 // ras association domain-containing protein 3-like // ---	LOC100051721	<0.05	2.95396
15092910	ENSECAT00000007616 // LOC100059972 // putative RNA polymerase II subunit B1 CTD phospha	LOC100059972	<0.05	2.9537
15088313	XM_001495159 // CCBL2 // cysteine conjugate-beta lyase 2 // --- // 100052141 /// ENSECA	CCBL2	<0.05	2.95261
14985671			<0.05	2.95237
15107779	XM_001501192 // USP47 // ubiquitin specific peptidase 47 // --- // 100071433 /// ENSECA	USP47	<0.05	2.95217
15114669	XM_001494131 // BRAP // BRCA1 associated protein // --- // 100057742 /// ENSECAT0000000	BRAP	<0.05	2.95203
15018344	NM_001163823 // SDHB // succinate dehydrogenase complex, subunit B, iron sulfur (Ip) //	SDHB	<0.05	2.9513
14971495	XM_001498106 // LOC100053249 // glutathione S-transferase P-like // --- // 100053249 //	LOC100053249	<0.05	2.94889
15012498	XM_001501275 // PCYT1A // phosphate cytidylyltransferase 1, choline, alpha // --- // 10	PCYT1A	<0.05	2.94749
15017855	XM_001501425 // HNRNPR // heterogeneous nuclear ribonucleoprotein R // --- // 100071619	HNRNPR	<0.05	2.94712
14933630	ENSECAT00000010382 // MMS19 // MMS19 nucleotide excision repair homolog (S. cerevisiae)	MMS19	<0.05	2.94692
14941745	XM_001496787 // SMC3 // structural maintenance of chromosomes 3 // --- // 100059335 ///	SMC3	<0.05	2.94568
15074323	XM_001488034 // LOC100053608 // 3'(2'),5'-bisphosphate nucleotidase 1-like // --- // 10	LOC100053608	<0.05	2.94397
15024163			<0.05	2.94392
15011775	XM_001495771 // TTC14 // tetratricopeptide repeat domain 14 // --- // 100058491 /// ENS	TTC14	<0.05	2.94187
14959587	ENSECAT00000027021 // PLEKHM1 // pleckstrin homology domain containing, family M (with	PLEKHM1	<0.05	2.94177
14947940			<0.05	2.93984
15033385	---		<0.05	2.93963
14979184	XM_001500978 // LOC100063559 // protein orai-3-like // --- // 100063559 /// ENSECAT0000	LOC100063559	<0.05	2.93925
15014563	XM_001499415 // SENP5 // SUMO1/sentrin specific peptidase 5 // --- // 100069684 /// ENS	SENP5	<0.05	2.93784
14988235			<0.05	2.93659
15056160	XM_001494597 // LOC100063334 // malignant fibrous histiocytoma-amplified sequence 1-lik	LOC100063334	<0.05	2.93649
14938550	ENSECAT00000001341 // LOC100070629 // spatacsin-like // --- // 100070629	LOC100070629	<0.05	2.93646
15091539	ENSECAT00000025732 // LOC100055389 // sodium channel modifier 1-like // --- // 10005538	LOC100055389	<0.05	2.9363
14990841	ENSECAT00000015437 // LOC100071892 // syndecan-1-like // --- // 100071892 /// XM_001501	LOC100071892	<0.05	2.93595
15105373	XM_001505079 // LOC100064327 // etoposide-induced protein 2.4 homolog // --- // 1000643	LOC100064327	<0.05	2.93502
14934969	XM_001504023 // ANXA11 // annexin A11 // --- // 100064425 /// ENSECAT00000021182 // ANX	ANXA11	<0.05	2.93436
14979157	ENSECAT00000011636 // LOC100063491 // 3 beta-hydroxysteroid dehydrogenase type 7-like /	LOC100063491	<0.05	2.93413
15067593	ENSECAT00000025132 // ATP8A1 // ATPase, aminophospholipid transporter (APLT), class I,	ATP8A1	<0.05	2.93374
14947933			<0.05	2.93332
15115626	ENSECAT00000007438 // LOC100057183 // uncharacterized protein C18orf19 homolog // --- /	LOC100057183	<0.05	2.93267
15016231	---		<0.05	2.93232
15040286	XM_003363924 // LOC100055373 // bladder cancer-associated protein-like // --- // 100055	LOC100055373	<0.05	2.93195
15015545	XM_001502968 // LOC100072898 // putative methyltransferase NSUN3-like // --- // 1000728	LOC100072898	<0.05	2.92964
15092465	---		<0.05	2.92933
14967469	XM_003362429 // MYO18A // myosin XVIIIA // --- // 100059638 /// ENSECAT00000022672 // M	MYO18A	<0.05	2.92716
14980116	XM_001488925 // RRN3 // RRN3 RNA polymerase I transcription factor homolog (S. cerevisi	RRN3	<0.05	2.92676
15090159	XM_001489103 // LOC100054440 // serine/threonine-protein phosphatase 2A 56 kDa regulato	LOC100054440	<0.05	2.92635
14939658	ENSECAT00000012091 // LOC100629897 // ribonuclease K6-like // --- // 100629897 /// XM_0	LOC100629897	<0.05	2.92623
15048157	ENSECAT00000001245 // LOC100049970 // histone-lysine N-methyltransferase setd3-like //	LOC100049970	<0.05	2.92476
15000805	XM_001496955 // ABHD5 // abhydrolase domain containing 5 // --- // 100066775 /// ENSECA	ABHD5	<0.05	2.92366
15111630	---		<0.05	2.92088
15012813	XM_001500297 // LOC100070623 // importin subunit alpha-1-like // --- // 100070623 /// E	LOC100070623	<0.05	2.92035
15054941	XM_001915192 // LTN1 // listerin E3 ubiquitin protein ligase 1 // --- // 100065649 ///	LTN1	<0.05	2.91737
14993184	XM_001498254 // FOXN2 // forkhead box N2 // --- // 100053059 /// ENSECAT00000008546 //	FOXN2	<0.05	2.91697
15074203			<0.05	2.91668
14985174	NM_001081912 // GM2A // GM2 ganglioside activator // --- // 100034082 /// ENSECAT000000	GM2A	<0.05	2.91629
15079178	XM_001496641 // LOC100066339 // putative RNA-binding protein Luc7-like 2-like // --- //	LOC100066339	<0.05	2.91518
15011304	XM_001505117 // NDUFS1 // NADH dehydrogenase (ubiquinone) Fe-S protein 1, 75kDa (NADH-c	NDUFS1	<0.05	2.91479
15088703	XM_001499174 // LOC100053515 // zinc finger Ran-binding domain-containing protein 2-lik	LOC100053515	<0.05	2.91401
15051115	XM_001917558 // LOC100059025 // arrestin domain-containing protein 1-like // --- // 100	LOC100059025	<0.05	2.91189
15028274	XM_001499713 // KCTD20 // potassium channel tetramerisation domain containing 20 // ---	KCTD20	<0.05	2.91145
15025826	XM_003364513 // LOC100630562 // microsomal glutathione S-transferase 2-like // --- // 1	LOC100630562	<0.05	2.90963
15003062	ENSECAT00000011756 // COG6 // component of oligomeric golgi complex 6 // --- // 1000508	COG6	<0.05	2.90934
15125361	XM_001917184 // ZNF251 // zinc finger protein 251 // --- // 100064391	ZNF251	<0.05	2.90933
14933917	XM_001917312 // NOC3L // nucleolar complex associated 3 homolog (S. cerevisiae) // ---	NOC3L	<0.05	2.90594
15106280	XM_001916046 // LOC100055350 // eukaryotic translation initiation factor 3 subunit G-li	LOC100055350	<0.05	2.90533
15031016	XM_001496005 // LOC100052481 // proteasome subunit beta type-8-like // --- // 100052481	LOC100052481	<0.05	2.90108
14956256	XM_001914953 // IBTK // inhibitor of Bruton agammaglobulinemia tyrosine kinase // --- /	IBTK	<0.05	2.89763
15001342	ENSECAT00000026925 // LOC100056080 // f-box/LRR-repeat protein 2-like // --- // 1000560	LOC100056080	<0.05	2.89724
15115781	NM_001163985 // ROCK1 // Rho-associated, coiled-coil containing protein kinase 1 // ---	ROCK1	<0.05	2.89649
14930119	---		<0.05	2.89601
15085297	XM_001489279 // LOC100054805 // zinc transporter 1-like // --- // 100054805 /// ENSECAT	LOC100054805	<0.05	2.89511
15129715	XM_001491785 // LOC100058835 // NADH dehydrogenase [ubiquinone] 1 alpha subcomplex subu	LOC100058835	<0.05	2.89305
15069524	XM_001916072 // LOC100146422 // IST1 homolog // --- // 100146422 /// ENSECAT00000024550	LOC100146422	<0.05	2.89281
15032840	XM_001503333 // LOC100070992 // UPF0459 protein C19orf50 homolog // --- // 100070992 //	LOC100070992	<0.05	2.89199
15091795	ENSECAT00000014675 // LOC100065612 // Golgi pH regulator-like // --- // 100065612 /// X	LOC100065612	<0.05	2.89061
15126632	XM_001498387 // LOC100068567 // guanine nucleotide-binding protein subunit alpha-15-lik	LOC100068567	<0.05	2.89056
14963548	XM_001504879 // AKAP10 // A kinase (PRKA) anchor protein 10 // --- // 100073152 /// ENS	AKAP10	<0.05	2.89047
15058413	ENSECAT00000010773 // GAS2L3 // growth arrest-specific 2 like 3 // --- // 100052872 ///	GAS2L3	<0.05	2.88935
15069790			<0.05	2.88871
14992400	XM_001500046 // LOC100053688 // serine protease HTRA2, mitochondrial-like // --- // 100	LOC100053688	<0.05	2.88856
15076724	ENSECAT00000018378 // LOC100050769 // b-cell receptor-associated protein 29-like // ---	LOC100050769	<0.05	2.88649
15134704	---		<0.05	2.88578
14970071	ENSECAT00000015605 // LOC100067021 // thioredoxin-related transmembrane protein 2-like	LOC100067021	<0.05	2.88441
14981437	ENSECAT00000012891 // ZNF354A // zinc finger protein 354A // --- // 100068186 /// XM_00	ZNF354A	<0.05	2.88394
15019835	XM_001495587 // ELP3 // elongation protein 3 homolog (S. cerevisiae) // --- // 10005468	ELP3	<0.05	2.88272
15016748	XM_001503462 // LOC100054282 // peptidyl-prolyl cis-trans isomerase E-like // --- // 10	LOC100054282	<0.05	2.87954
15025716	XM_001501949 // ABCE1 // ATP-binding cassette, sub-family E (OABP), member 1 // --- //	ABCE1	<0.05	2.87644
14995006	XM_001492042 // LOC100056766 // 60S ribosomal protein L32-like // --- // 100056766 ///	LOC100056766	<0.05	2.8737
15107234	ENSECAT00000022985 // PGAP2 // post-GPI attachment to proteins 2 // --- // ---	PGAP2	<0.05	2.87194
15007474	ENSECAT00000009408 // AGPS // alkylglycerone phosphate synthase // --- // ---	AGPS	<0.05	2.87072
15111217	XM_001493676 // LOC100055431 // ubiquitin-like protein 5-like // --- // 100055431 /// E	LOC100055431	<0.05	2.86962
14972182	ENSECAT00000022523 // TRAF6 // TNF receptor-associated factor 6, E3 ubiquitin protein l	TRAF6	<0.05	2.86908
14991615	ENSECAT00000024261 // EIF5B // eukaryotic translation initiation factor 5B // --- // 10	EIF5B	<0.05	2.86841
15035725	ENSECAT00000000250 // WDR70 // WD repeat domain 70 // --- // ---	WDR70	<0.05	2.86682
14952626	XM_001915854 // AIM1 // absent in melanoma 1 // --- // 100066371 /// ENSECAT00000015934	AIM1	<0.05	2.86467

15008105	XM_001503625 // LOC100067936 // NADH dehydrogenase [ubiquinone] 1 beta subcomplex subun	LOC100067936	<0.05	2.86365
15098365	ENSECAT00000009629 // LANCL1 // LanC lantibiotic synthetase component C-like 1 (bacteri	LANCL1	<0.05	2.86347
15049168	ENSECAT00000026861 // HSDL2 // hydroxysteroid dehydrogenase like 2 // --- // 100057249	HSDL2	<0.05	2.86016
15054719	---		<0.05	2.85902
15113996	ENSECAT00000006341 // LOC100066526 // glycolipid transfer protein-like // --- // 100066	LOC100066526	<0.05	2.85899
14928167	---		<0.05	2.85898
15047952	ENSECAT00000025731 // ITPK1 // inositol-tetrakisphosphate 1-kinase // --- // ---	ITPK1	<0.05	2.85758
14945627	ENSECAT00000026577 // TBC1D2B // TBC1 domain family, member 2B // --- // 100060015 ///	TBC1D2B	<0.05	2.85713
15124184	ENSECAT00000000231 // EIF3E // eukaryotic translation initiation factor 3, subunit E //	EIF3E	<0.05	2.85634
15101338	XM_001914700 // LOC100049989 // zinc finger CCHC-type and RNA-binding motif-containing	LOC100049989	<0.05	2.85586
15096516	ENSECAT00000014031 // ALG10 // asparagine-linked glycosylation 10, alpha-1,2-glucosyltr	ALG10	<0.05	2.85585
15121187	---		<0.05	2.85575
15053593	NM_001081940 // GBE1 // glucan (1,4-alpha-), branching enzyme 1 // --- // 100034152 ///	GBE1	<0.05	2.85551
15061761	---		<0.05	2.8554
15118767	XM_001496946 // ZCCHC8 // zinc finger, CCHC domain containing 8 // --- // 100058826 ///	ZCCHC8	<0.05	2.85472
14976380	ENSECAT00000025755 // LOC100068356 // conserved oligomeric Golgi complex subunit 7-like	LOC100068356	<0.05	2.85432
15118668			<0.05	2.85084
15073388	XM_001488930 // EPRS // glutamyl-prolyl-tRNA synthetase // --- // 100050245 /// ENSECAT	EPRS	<0.05	2.85032
15001238	XM_001489013 // GOLGA4 // golgin A4 // --- // 100050216 /// ENSECAT00000014443 // GOLGA	GOLGA4	<0.05	2.85019
14974139	ENSECAT00000018528 // CPT1A // carnitine palmitoyltransferase 1A (liver) // --- // 1000	CPT1A	<0.05	2.85012
14971870	ENSECAT00000013475 // LOC100055228 // tetraspanin-4-like // --- // 100055228 /// XM_001	LOC100055228	<0.05	2.84996
15035530	ENSECAT00000012122 // PARP8 // poly (ADP-ribose) polymerase family, member 8 // --- //	PARP8	<0.05	2.84795
15014887	XM_001917233 // LOC100060927 // general transcription factor IIE subunit 1-like // ---	LOC100060927	<0.05	2.84752
15121392	ENSECAT00000014189 // LOC100055036 // transcription elongation factor A protein 1-like	LOC100055036	<0.05	2.84621
14957828	ENSECAT00000017544 // LOC100073201 // ralBP1-associated Eps domain-containing protein 1	LOC100073201	<0.05	2.84326
15013076	ENSECAT00000024846 // LOC100071372 // n-alpha-acetyltransferase 50, NatE catalytic subu	LOC100071372	<0.05	2.84241
15001041	XM_001498297 // WDR48 // WD repeat domain 48 // --- // 100068457 /// ENSECAT00000020117	WDR48	<0.05	2.84187
15009057	XM_001487889 // ORC4 // origin recognition complex, subunit 4 // --- // 100055921 /// E	ORC4	<0.05	2.83973
15041011	ENSECAT00000000477 // LOC100056734 // protein slowmo homolog 2-like // --- // 100056734	LOC100056734	<0.05	2.83885
15082247	XM_001501128 // LOC100055464 // homocysteine-responsive endoplasmic reticulum-resident	LOC100055464	<0.05	2.83884
15027712	NM_001256923 // HSPA1A // heat shock 70kDa protein 1A // --- // 100050827 /// AF397192	HSPA1A	<0.05	2.83753
15046955	---		<0.05	2.83744
14997130	ENSECAT00000026712 // LOC100065901 // uncharacterized protein KIAA1143-like // --- // 1	LOC100065901	<0.05	2.83705
15015011	XM_001501053 // QTRTD1 // queuine tRNA-ribosyltransferase domain containing 1 // --- //	QTRTD1	<0.05	2.83623
15116557	ENSECAT00000005340 // LOC100070107 // DNA polymerase iota-like // --- // 100070107 ///	LOC100070107	<0.05	2.8351
15128880	XM_001502247 // LOC100072334 // apolipoprotein O-like // --- // 100072334 /// ENSECAT00	LOC100072334	<0.05	2.83421
14946548	ENSECAT00000020987 // GCOM1 // GRINL1A complex locus 1 // --- // 100068476	GCOM1	<0.05	2.834
14995890	AY343543 // TKT // transketolase // --- // 100034031	TKT	<0.05	2.83376
14972487	ENSECAT00000015176 // LOC100051744 // mitochondrial carrier homolog 2-like // --- // 10	LOC100051744	<0.05	2.83293
14943266	XM_001503713 // LOC100063387 // apoptosis-inducing factor 2-like // --- // 100063387 //	LOC100063387	<0.05	2.83144
15108508	XM_001504389 // LOC100068453 // mps one binder kinase activator-like 2A-like // --- //	LOC100068453	<0.05	2.83087
15035560	ENSECAT00000009638 // MRPS30 // mitochondrial ribosomal protein S30 // --- // ---	MRPS30	<0.05	2.82891
15095274	ENSECAT00000006674 // LOC100054988 // BH3-interacting domain death agonist-like // ---	LOC100054988	<0.05	2.82867
14988058	XM_001490447 // LOC100050737 // MIT domain-containing protein 1-like // --- // 10005073	LOC100050737	<0.05	2.82832
14975739	ENSECAT00000015734 // GUSB // glucuronidase, beta // --- // 100037416 /// XM_001493514	GUSB	<0.05	2.82701
14928205	---		<0.05	2.82696
14958261	AJ010315 // TIMP2 // TIMP metallopeptidase inhibitor 2 // --- // 100034134 /// EF077283	TIMP2	<0.05	2.82676
14999883	XM_003363080 // LOC100629294 // signal peptidase complex subunit 1-like // --- // 10062	LOC100629294	<0.05	2.82514
15066947	XM_001489101 // GRSF1 // G-rich RNA sequence binding factor 1 // --- // 100054436 /// E	GRSF1	<0.05	2.82451
14994788	XM_003363052 // LOC100050146 // cellular nucleic acid-binding protein-like // --- // 10	LOC100050146	<0.05	2.82292
15117667	ENSECAT00000022284 // LOC100063639 // transcobalamin-2-like // --- // 100063639 /// XM_	LOC100063639	<0.05	2.82226
15063713	ENSECAT00000009117 // LOC100070366 // uncharacterized protein C10orf18-like // --- // 1	LOC100070366	<0.05	2.82173
15028928			<0.05	2.82108
15002662	XM_001493058 // LOC100060960 // transcription factor IIIA-like // --- // 100060960 ///	LOC100060960	<0.05	2.821
14967287	XM_001504140 // LOC100059151 // protein unc-119 homolog A-like // --- // 100059151 ///	LOC100059151	<0.05	2.81741
14958744	XM_001492288 // LOC100059836 // MIF4G domain-containing protein-like // --- // 10005983	LOC100059836	<0.05	2.81665
14959493	XM_003362531 // LOC100054597 // MLL1/MLL complex subunit KIAA1267-like // --- // 100054	LOC100054597	<0.05	2.81661
15121915	ENSECAT00000021375 // VPS13B // vacuolar protein sorting 13 homolog B (yeast) // --- //	VPS13B	<0.05	2.81604
14984985	XM_001503474 // LOC100059774 // tetratricopeptide repeat protein 1-like // --- // 10005	LOC100059774	<0.05	2.81587
15063552	XM_001499137 // CDC123 // cell division cycle 123 homolog (S. cerevisiae) // --- // 100	CDC123	<0.05	2.81521
14997632	ENSECAT00000024825 // NGLY1 // N-glycanase 1 // --- // 100059456 /// XM_001492043 // NG	NGLY1	<0.05	2.81449
14956503	XM_001500685 // LOC100071009 // ubiquitin-conjugating enzyme E2 J1-like // --- // 10007	LOC100071009	<0.05	2.814
15069409	XM_001499093 // LOC100053955 // protein SLC7A6OS-like // --- // 100053955 /// ENSECAT00	LOC100053955	<0.05	2.81383
15088261	XM_001493570 // ZNF644 // zinc finger protein 644 // --- // 100051777 /// ENSECAT000000	ZNF644	<0.05	2.81246
15091467	XM_003365031 // LOC100059946 // tuftelin-like // --- // 100059946 /// ENSECAT0000001160	LOC100059946	<0.05	2.81034
15101311	XM_001915404 // SLC2A13 // solute carrier family 2 (facilitated glucose transporter), m	SLC2A13	<0.05	2.80955
15058212	XM_001494372 // METAP2 // methionyl aminopeptidase 2 // --- // 100052132 /// XM_0014943	METAP2	<0.05	2.80895
15122482	XM_001498132 // LOC100057623 // NADH dehydrogenase [ubiquinone] 1 beta subcomplex subun	LOC100057623	<0.05	2.80759
15011788	XM_001495885 // FXR1 // fragile X mental retardation, autosomal homolog 1 // --- // 100	FXR1	<0.05	2.80666
14970550	XM_001916375 // LOC100146759 // tetratricopeptide repeat protein 9C-like // --- // 1001	LOC100146759	<0.05	2.80441
15046770	ENSECAT00000008793 // LOC100058449 // exocyst complex component 5-like // --- // 100058	LOC100058449	<0.05	2.80425
14983486	XM_001503567 // LOC100073191 // stAR-related lipid transfer protein 4-like // --- // 10	LOC100073191	<0.05	2.80391
15064836	ENSECAT00000014815 // LOC100053856 // nuclear factor of activated T-cells, cytoplasmic	LOC100053856	<0.05	2.80369
15006245	ENSECAT00000010106 // IWS1 // IWS1 homolog (S. cerevisiae) // --- // ---	IWS1	<0.05	2.80352
14956739	XM_001503881 // USP45 // ubiquitin specific peptidase 45 // --- // 100071616 /// ENSECA	USP45	<0.05	2.80352
15090433	XM_001491629 // UHMK1 // U2AF homology motif (UHM) kinase 1 // --- // 100058796 /// ENS	UHMK1	<0.05	2.80292
14981651	ENSECAT00000003803 // LOC100058730 // UPF0498 protein KIAA1191-like // --- // 100058730	LOC100058730	<0.05	2.80282
15047620	XM_001492371 // GTF2A1 // general transcription factor IIA, 1, 19/37kDa // --- // 10005	GTF2A1	<0.05	2.80248
15134700	---		<0.05	2.80234
15088939	XM_001500154 // LOC100070493 // TM2 domain-containing protein 1-like // --- // 10007049	LOC100070493	<0.05	2.80136
15002429	XM_001488592 // PARP4 // poly (ADP-ribose) polymerase family, member 4 // --- // 100053	PARP4	<0.05	2.80134
15127040	XM_001917193 // MOSPD2 // motile sperm domain containing 2 // --- // 100055695 /// ENSE	MOSPD2	<0.05	2.80099
15069423	ENSECAT00000009199 // LOC100054003 // chromosome transmission fidelity protein 8 homolo	LOC100054003	<0.05	2.79977
15133906	ENSECAT00000011997 // LOC100058256 // filamin-A-like // --- // 100058256	LOC100058256	<0.05	2.79886
15135426	---		<0.05	2.79825
14930417	---		<0.05	2.79608
15135940	---		<0.05	2.79594
15068679	XM_001491009 // N4BP1 // NEDD4 binding protein 1 // --- // 100057787 /// ENSECAT0000002	N4BP1	<0.05	2.79538
15032504	XM_003363817 // AP1M1 // adaptor-related protein complex 1, mu 1 subunit // --- // 1000	AP1M1	<0.05	2.79432
14956385	ENSECAT00000013411 // SNX14 // sorting nexin 14 // --- // 100065533	SNX14	<0.05	2.79409
14925075	---		<0.05	2.79408
15106791	ENSECAT00000024510 // ALG8 // asparagine-linked glycosylation 8, alpha-1,3-glucosyltran	ALG8	<0.05	2.79402
14932053	---		<0.05	2.79215
14946695	ENSECAT00000023924 // LOC100055314 // AP-4 complex subunit epsilon-1-like // --- // 100	LOC100055314	<0.05	2.79213
15078932	ENSECAT00000026029 // LOC100068568 // muskelin-like // --- // 100068568 /// XM_00150099	LOC100068568	<0.05	2.79195
14994258	XM_001503106 // NCOA1 // nuclear receptor coactivator 1 // --- // 100056042 /// XM_0033	NCOA1	<0.05	2.79162
15108981	XM_001496766 // LOC100066525 // syntaxin-binding protein 2-like // --- // 100066525 ///	LOC100066525	<0.05	2.79113
15016465	ENSECAT00000026685 // LOC100065903 // transmembrane protein 53-like // --- // 100065903	LOC100065903	<0.05	2.7898
15114735	XM_001495246 // LOC100059433 // GPN-loop GTPase 3-like // --- // 100059433 /// ENSECAT0	LOC100059433	<0.05	2.78939
15095534	XM_001494601 // LOC100051504 // NADH dehydrogenase [ubiquinone] 1 alpha subcomplex subu	LOC100051504	<0.05	2.78931
15126627			<0.05	2.78915
15067222	NM_001105316 // PPAT // phosphoribosyl pyrophosphate amidotransferase // --- // 1000538	PPAT	<0.05	2.78822
15090762	XM_001490503 // LOC100056988 // myeloid cell nuclear differentiation antigen-like // --	LOC100056988	<0.05	2.78772
15121144	XM_001494215 // COPS5 // COP9 constitutive photomorphogenic homolog subunit 5 (Arabidop	COPS5	<0.05	2.78732
15122756	ENSECAT00000014440 // LOC100063382 // glycosylphosphatidylinositol anchor attachment 1	LOC100063382	<0.05	2.78712
14992130	XM_001498189 // LOC100053006 // 39S ribosomal protein L35, mitochondrial-like // --- //	LOC100053006	<0.05	2.78685

15099658 NM_001163830 // ADIPOR2 // adiponectin receptor 2 // --- // 100050051 /// ENSECAT000000	ADIPOR2	<0.05	2.78628
15118895 ENSECAT00000022871 // LOC100059129 // translation initiation factor eIF-2B subunit alph	LOC100059129	<0.05	2.78413
15011241 XM_001497226 // LOC100067107 // small ubiquitin-related modifier 1-like // --- // 10006	LOC100067107	<0.05	2.78383
14969333 ENSECAT00000017152 // FBXO3 // F-box protein 3 // --- // 100059295 /// XM_001503169 //	FBXO3	<0.05	2.78189
15105879 ENSECAT00000014998 // TNPO2 // transportin 2 // --- // 100063482 /// XM_001504888 // TN	TNPO2	<0.05	2.78189
15084828 ENSECAT00000020986 // LOC100054060 // major histocompatibility complex class I-related	LOC100054060	<0.05	2.78145
15080649 ENSECAT00000007739 // VPS41 // vacuolar protein sorting 41 homolog (S. cerevisiae) // -	VPS41	<0.05	2.78137
14934146 XM_001503226 // SGMS1 // sphingomyelin synthase 1 // --- // 100062484 /// ENSECAT000000	SGMS1	<0.05	2.78132
15083184 XM_001496338 // MKRN1 // makorin ring finger protein 1 // --- // 100065893 /// ENSECAT0	MKRN1	<0.05	2.77906
15129767 XM_001500906 // XIAP // X-linked inhibitor of apoptosis // --- // 100060870 /// ENSECAT	XIAP	<0.05	2.77902
15135002 ---		<0.05	2.77879
15074567 XM_001492621 // ZBTB41 // zinc finger and BTB domain containing 41 // --- // 100051497	ZBTB41	<0.05	2.77728
14983173 ENSECAT00000022661 // RAPGEF6 // Rap guanine nucleotide exchange factor (GEF) 6 // ---	RAPGEF6	<0.05	2.77659
15051570 XM_001504059 // ALG2 // asparagine-linked glycosylation 2, alpha-1,3-mannosyltransferas	ALG2	<0.05	2.77659
15098880 XM_001495406 // FARSB // phenylalanyl-tRNA synthetase, beta subunit // --- // 100056565	FARSB	<0.05	2.77543
14967499 XM_001918341 // NUFIP2 // nuclear fragile X mental retardation protein interacting prot	NUFIP2	<0.05	2.77399
14928817 ---		<0.05	2.77394
15134558 ---		<0.05	2.77387
14937368 XM_001497645 // DENND4A // DENN/MADD domain containing 4A // --- // 100053090 /// XM_00	DENND4A	<0.05	2.77318
14956757 ENSECAT00000026298 // LOC100066119 // cyclin-C-like // --- // 100066119 /// XM_00150389	LOC100066119	<0.05	2.77307
14964932 XM_001499857 // HELZ // helicase with zinc finger // --- // 100053580 /// ENSECAT000000	HELZ	<0.05	2.7712
14987308 XM_001503946 // AGGF1 // angiogenic factor with G patch and FHA domains 1 // --- // 100	AGGF1	<0.05	2.76992
15088722 XM_001499345 // ANKRD13C // ankyrin repeat domain 13C // --- // 100053662 /// ENSECAT00	ANKRD13C	<0.05	2.7696
15052893 XM_001917228 // CRAT // carnitine O-acetyltransferase // --- // 100070062 /// ENSECAT00	CRAT	<0.05	2.76943
14988770 XM_001497585 // LOC100067584 // succinyl-CoA ligase [GDP-forming] subunit alpha, mitoch	LOC100067584	<0.05	2.76579
15069451 ENSECAT00000021852 // LOC100067237 // uncharacterized LOC100067237 // --- // 100067237	LOC100067237	<0.05	2.76557
14959958 ENSECAT00000017794 // VAT1 // vesicle amine transport protein 1 homolog (T. californica	VAT1	<0.05	2.76448
15066146 ENSECAT00000008640 // DNAJB14 // DnaJ (Hsp40) homolog, subfamily B, member 14 // --- //	DNAJB14	<0.05	2.76424
15003272 XM_003363225 // LOC100050430 // succinyl-CoA ligase [ADP-forming] subunit beta, mitocho	LOC100050430	<0.05	2.76396
15099853 ENSECAT00000023705 // LOC100051294 // uncharacterized protein C12orf4 homolog // --- //	LOC100051294	<0.05	2.76352
15064782 XM_001498248 // LOC100053553 // nuclear transport factor 2-like // --- // 100053553 ///	LOC100053553	<0.05	2.76266
14940154 XM_001489375 // LOC100054994 // dehydrogenase/reductase SDR family member 4-like // ---	LOC100054994	<0.05	2.76234
15105599 ENSECAT00000011541 // ACAD8 // acyl-CoA dehydrogenase family, member 8 // --- // 100072	ACAD8	<0.05	2.76225
15089816 ENSECAT00000012272 // LOC100054016 // syntaxin-6-like // --- // 100054016 /// XM_001488	LOC100054016	<0.05	2.76194
14999688 XM_001491548 // APPL1 // adaptor protein, phosphotyrosine interaction, PH domain and le	APPL1	<0.05	2.76147
15012747 XM_001500147 // LOC100070489 // 3-hydroxyacyl-CoA dehydratase 2-like // --- // 10007048	LOC100070489	<0.05	2.76123
14934217 NM_001242449 // UBE2D1 // ubiquitin-conjugating enzyme E2D 1 // --- // 100062598 /// EN	UBE2D1	<0.05	2.7612
15097530 XM_001504781 // LOC100051518 // ORM1-like protein 2-like // --- // 100051518 /// ENSECA	LOC100051518	<0.05	2.76071
15109929 ENSECAT00000015047 // LOC100063051 // intraflagellar transport protein 46 homolog // --	LOC100063051	<0.05	2.76008
15119945 XM_001496673 // ZNF24 // zinc finger protein 24 // --- // 100052725 /// ENSECAT00000021	ZNF24	<0.05	2.75997
15105278 XM_001505067 // TBRG1 // transforming growth factor beta regulator 1 // --- // 10006418	TBRG1	<0.05	2.75944
15074418 ENSECAT00000010619 // LOC100056607 // RRP15-like protein-like // --- // 100056607 /// X	LOC100056607	<0.05	2.7591
15051240 ENSECAT00000019337 // LOC100055711 // uncharacterized protein KIAA1539-like // --- // 1	LOC100055711	<0.05	2.75841
15126705		<0.05	2.75792
15057230 ENSECAT00000011594 // ERI1 // exoribonuclease 1 // --- // 100147421 /// XM_001915823 //	ERI1	<0.05	2.75714
14992226 ENSECAT00000029001 // LOC100067403 // trans-Golgi network integral membrane protein 1-l	LOC100067403	<0.05	2.75644
15123526 ENSECAT00000018083 // UBXN2B // UBX domain protein 2B // --- // ---	UBXN2B	<0.05	2.75484
15102326 XM_001504543 // ATF7 // activating transcription factor 7 // --- // 100062229 /// ENSEC	ATF7	<0.05	2.75468
15101931 ENSECAT00000007770 // LASS5 // ceramide synthase 5 // --- // 100059999 /// XM_001504248	LASS5	<0.05	2.75392
15008507 XM_001504925 // LOC100067394 // protein FAM168B-like // --- // 100067394 /// ENSECAT000	LOC100067394	<0.05	2.75336
14998428 ---		<0.05	2.75303
15057193 XM_001495715 // LOC100058409 // dynactin subunit 6-like // --- // 100058409 /// ENSECAT	LOC100058409	<0.05	2.7526
15031488 ENSECAT00000018050 // MOCS1 // molybdenum cofactor synthesis 1 // --- // 100065704 ///	MOCS1	<0.05	2.75257
14988179 ENSECAT00000000093 // LOC100062047 // ankyrin repeat domain-containing protein 39-like	LOC100062047	<0.05	2.75031
14974702 ENSECAT00000000171 // LOC100061601 // probable palmitoyltransferase ZDHHC4-like // ---	LOC100061601	<0.05	2.75008
15035640 XM_001496286 // LOC100065794 // UPF0600 protein C5orf51-like // --- // 100065794 /// EN	LOC100065794	<0.05	2.74974
15049923 ENSECAT00000012147 // SLC27A4 // solute carrier family 27 (fatty acid transporter), mem	SLC27A4	<0.05	2.74927
15119860 XM_001495283 // TRAPPC8 // trafficking protein particle complex 8 // --- // 100052273 /	TRAPPC8	<0.05	2.74822
14928681 ---		<0.05	2.74745
14939241 XM_001503583 // LOC100057403 // signal recognition particle 14 kDa protein-like // ---	LOC100057403	<0.05	2.74595
15009076 XM_001488182 // LOC100050023 // methylmalonic aciduria and homocystinuria type D homolo	LOC100050023	<0.05	2.74584
14973472 ENSECAT00000021404 // LOC100054275 // atlastin-3-like // --- // 100054275 /// XM_001488	LOC100054275	<0.05	2.74465
14941610 XM_001495643 // NHLRC2 // NHL repeat containing 2 // --- // 100059070 /// ENSECAT000000	NHLRC2	<0.05	2.74216
15061854 XM_001490463 // LOC100056911 // arylsulfatase A-like // --- // 100056911 /// ENSECAT000	LOC100056911	<0.05	2.74105
15062032 XM_001494766 // MAP3K8 // mitogen-activated protein kinase kinase kinase 8 // --- // 10	MAP3K8	<0.05	2.74066
14947096 XM_001918264 // TMEM62 // transmembrane protein 62 // --- // 100056447 /// ENSECAT00000	TMEM62	<0.05	2.74049
14958435 ENSECAT00000023874 // LOC100051178 // inactive rhomboid protein 2-like // --- // 100051	LOC100051178	<0.05	2.73939
15036301 DQ402987 // SDHA // succinate dehydrogenase complex, subunit A, flavoprotein (Fp) // --	SDHA	<0.05	2.73917
14977797 ENSECAT00000018658 // NPRL3 // nitrogen permease regulator-like 3 (S. cerevisiae) // --	NPRL3	<0.05	2.73766
15123972 XM_001492262 // RNF19A // ring finger protein 19A, E3 ubiquitin protein ligase // --- /	RNF19A	<0.05	2.73693
14966080 XM_001501874 // LOC100055406 // 39S ribosomal protein L10, mitochondrial-like // --- //	LOC100055406	<0.05	2.73688
14928169 ---		<0.05	2.73548
15037905 ENSECAT00000015014 // DHX35 // DEAH (Asp-Glu-Ala-His) box polypeptide 35 // --- // 1000	DHX35	<0.05	2.73534
15071400 XM_001916811 // LOC100058152 // RE1-silencing transcription factor-like // --- // 10005	LOC100058152	<0.05	2.73529
15131490 ENSECAT00000024242 // LOC100059599 // FUN14 domain-containing protein 1-like // --- //	LOC100059599	<0.05	2.73506
15126633		<0.05	2.73479
14987439 ENSECAT00000012513 // LOC100073289 // beta-hexosaminidase subunit beta-like // --- // 1	LOC100073289	<0.05	2.73364
15083640 XM_001495351 // LOC100064440 // GTP-binding protein Rheb-like // --- // 100064440 /// E	LOC100064440	<0.05	2.73235
15023292 XM_001501046 // LOC100071309 // macoilin-like // --- // 100071309 /// ENSECAT0000000321	LOC100071309	<0.05	2.731
15072515 ENSECAT00000009087 // ACOX3 // acyl-CoA oxidase 3, pristanoyl // --- // 100056690 /// X	ACOX3	<0.05	2.72916
14970086 ENSECAT00000019101 // CTNND1 // catenin (cadherin-associated protein), delta 1 // --- /	CTNND1	<0.05	2.72911
15068447 ENSECAT00000016987 // CTBP1 // C-terminal binding protein 1 // --- // 100052216 /// XM_	CTBP1	<0.05	2.72909
15058287 ENSECAT00000014574 // NEDD1 // neural precursor cell expressed, developmentally down-re	NEDD1	<0.05	2.7278
15005938 ENSECAT00000028905 // ARGLU1 // arginine and glutamate rich 1 // --- // ---	ARGLU1	<0.05	2.72744
15035018 XM_001491054 // CDK7 // cyclin-dependent kinase 7 // --- // 100049964 /// ENSECAT000000	CDK7	<0.05	2.72538
14930497 ---		<0.05	2.72361
15033526		<0.05	2.7225
15090605 ENSECAT00000013398 // LOC100058797 // SLAM family member 7-like // --- // 100058797 ///	LOC100058797	<0.05	2.72242
14988625 XM_001496953 // LOC100066774 // t-cell surface glycoprotein CD8 alpha chain-like // ---	LOC100066774	<0.05	2.72205
15036539 XM_001493978 // SNX5 // sorting nexin 5 // --- // 100062401 /// ENSECAT00000018594 // S	SNX5	<0.05	2.7219
15006261 ENSECAT00000023172 // MAP3K2 // mitogen-activated protein kinase kinase kinase 2 // ---	MAP3K2	<0.05	2.72188
15062552 XM_001499156 // LOC100056812 // ADP-sugar pyrophosphatase-like // --- // 100056812 ///	LOC100056812	<0.05	2.72153
15017528 XM_001504025 // LOC100056884 // wiskott-Aldrich syndrome protein family member 2-like /	LOC100056884	<0.05	2.72108
14965658 XM_001494084 // LOC100052692 // max-like protein X-like // --- // 100052692 /// XM_0014	LOC100052692	<0.05	2.71809
15120299 XM_001499216 // CXXC1 // CXXC finger protein 1 // --- // 100069452 /// ENSECAT000000190	CXXC1	<0.05	2.71676
15070282 ENSECAT00000021872 // LOC100630458 // CDGSH iron-sulfur domain-containing protein 2-lik	LOC100630458	<0.05	2.71662
15052312 XM_001501657 // LOC100071800 // 26S proteasome non-ATPase regulatory subunit 5-like //	LOC100071800	<0.05	2.71641
15129374 ---		<0.05	2.71634
15128390 ENSECAT00000000280 // LOC100066275 // protein YIPF6-like // --- // 100066275 /// XM_001	LOC100066275	<0.05	2.7162
15107050 ENSECAT00000024371 // LOC100065545 // ras-related protein Rab-6A-like // --- // 1000655	LOC100065545	<0.05	2.71609
15100800 XM_001502159 // RECQL // RecQ protein-like (DNA helicase Q1-like) // --- // 100068195 /	RECQL	<0.05	2.71446
15016763 ENSECAT00000016449 // LOC100068904 // c-Myc-binding protein-like // --- // 100068904 //	LOC100068904	<0.05	2.71414
15076945 XM_001495859 // LOC100051895 // synaptobrevin homolog YKT6-like // --- // 100051895 ///	LOC100051895	<0.05	2.7141
14983505 ENSECAT00000008235 // PJA2 // praja ring finger 2, E3 ubiquitin protein ligase // --- /	PJA2	<0.05	2.71258
15008235 ENSECAT00000022068 // NBEAL1 // neurobeachin-like 1 // --- // ---	NBEAL1	<0.05	2.71136
15060549 ENSECAT00000013639 // LOC100053117 // WASH complex subunit CCDC53-like // --- // 100053	LOC100053117	<0.05	2.71009

15111087	ENSECAT00000011689 // SLC44A2 // solute carrier family 44, member 2 // --- // 100055038	SLC44A2	<0.05	2.70946
15105493	ENSECAT00000022701 // FLI1 // Friend leukemia virus integration 1 // --- // 100064591 /	FLI1	<0.05	2.70834
15126109	ENSECAT00000004611 // LOC100630659 // UPF0545 protein C22orf39-like // --- // 100630659	LOC100630659	<0.05	2.70807
14959426	ENSECAT00000025770 // STRADA // STE20-related kinase adaptor alpha // --- // ---	STRADA	<0.05	2.70712
14928415	---		<0.05	2.70687
14928503	---		<0.05	2.70687
15085619	XM_001487994 // LOC100050332 // dual specificity protein phosphatase 12-like // --- //	LOC100050332	<0.05	2.70635
14948624	ENSECAT00000013453 // LOC100057360 // sorting nexin-6-like // --- // 100057360 /// XM_0	LOC100057360	<0.05	2.70476
15124457	XM_001497638 // LOC100057464 // zinc fingers and homeoboxes protein 1-like // --- // 10	LOC100057464	<0.05	2.70449
14998229	ENSECAT00000019828 // LOC100064781 // ras-related protein M-Ras-like // --- // 10006478	LOC100064781	<0.05	2.70405
14930809	---		<0.05	2.70359
15017433	XM_001503934 // MECR // mitochondrial trans-2-enoyl-CoA reductase // --- // 100070598 /	MECR	<0.05	2.70162
15091054	XM_001499888 // LMNA // lamin A/C // --- // 100057663 /// ENSECAT00000011877 // LMNA //	LMNA	<0.05	2.70102
15084024	ENSECAT00000020621 // LOC100051000 // ras association domain-containing protein 5-like	LOC100051000	<0.05	2.70092
15031770	XM_001497724 // SLC35B2 // solute carrier family 35, member B2 // --- // 100067750 ///	SLC35B2	<0.05	2.70031
14979576	AY184957 // RBBP6 // retinoblastoma binding protein 6 // --- // 100033991 /// ENSECAT00	RBBP6	<0.05	2.69869
15121328	---		<0.05	2.69642
14982551	XM_001503978 // LOC100061405 // histone deacetylase 3-like // --- // 100061405 /// ENSE	LOC100061405	<0.05	2.69383
14956977	XM_001504003 // MICAL1 // microtubule associated monoxygenase, calponin and LIM domain	MICAL1	<0.05	2.69181
14960949			<0.05	2.69118
15033055	ENSECAT00000001411 // TRIM23 // tripartite motif containing 23 // --- // 100050517 ///	TRIM23	<0.05	2.69059
14984044	XM_001504660 // LOC100065465 // tubulin-specific chaperone A-like // --- // 100065465 /	LOC100065465	<0.05	2.6901
15040426	XM_001500443 // ADA // adenosine deaminase // --- // 100070773 /// ENSECAT00000008811 /	ADA	<0.05	2.68904
15084742	ENSECAT00000008540 // CEP350 // centrosomal protein 350kDa // --- // 100051014 /// XM_0	CEP350	<0.05	2.68798
15093182	XM_001497167 // LOC100064844 // ribosome production factor 1-like // --- // 100064844 /	LOC100064844	<0.05	2.68604
15028684	ENSECAT00000003760 // LOC100067007 // 60S ribosomal protein L7-like 1-like // --- // 10	LOC100067007	<0.05	2.68472
15032479	ENSECAT00000006719 // LOC100147248 // ras-related protein Rab-8A-like // --- // 1001472	LOC100147248	<0.05	2.68458
15020688	XM_001502431 // LOC100063257 // histone-lysine N-methyltransferase SETD7-like // --- //	LOC100063257	<0.05	2.68446
15133154	ENSECAT00000011124 // THOC2 // THO complex 2 // --- // 100055091 /// XM_001500947 // TH	THOC2	<0.05	2.68387
14931993	---		<0.05	2.68321
14972946	ENSECAT00000015673 // LOC100060920 // 39S ribosomal protein L16, mitochondrial-like //	LOC100060920	<0.05	2.68218
15053764	XM_001500020 // LOC100053905 // junctional adhesion molecule B-like // --- // 100053905	LOC100053905	<0.05	2.68149
15107414	---		<0.05	2.68114
14967812	ENSECAT00000014229 // METTL16 // methyltransferase like 16 // --- // 100060313 /// XM_0	METTL16	<0.05	2.68098
14982977	XM_001917927 // LOC100072780 // serine/threonine-protein phosphatase 2A catalytic subun	LOC100072780	<0.05	2.6804
15103994	ENSECAT00000011477 // LOC100067852 // centrosomal protein of 57 kDa-like // --- // 1000	LOC100067852	<0.05	2.67977
15000661	ENSECAT00000010050 // LOC100065601 // phosphatidylinositide phosphatase SAC1-like // --	LOC100065601	<0.05	2.67948
15047046	XM_001499883 // LOC100052868 // phosphatidylinositol N-acetylglucosaminyltransferase su	LOC100052868	<0.05	2.67921
15056014	XM_001491885 // LOC100059204 // phosphatidate phosphatase PPAPDC1B-like // --- // 10005	LOC100059204	<0.05	2.67856
15132026	XM_001490186 // LOC100056440 // PDZ domain-containing protein 11-like // --- // 1000564	LOC100056440	<0.05	2.67826
15038464	XM_003363892 // LOC100071474 // CCAAT/enhancer-binding protein beta-like // --- // 1000	LOC100071474	<0.05	2.6763
14932770	ENSECAT00000013781 // LOC100058684 // uncharacterized protein C10orf46-like // --- // 1	LOC100058684	<0.05	2.6762
14979825	XM_001494381 // LOC100057847 // cytochrome b-c1 complex subunit 2, mitochondrial-like /	LOC100057847	<0.05	2.6748
15115331	XM_001493904 // LOC100062278 // tRNA pseudouridine synthase A, mitochondrial-like // --	LOC100062278	<0.05	2.67377
15114631	ENSECAT00000009442 // NAA25 // N(alpha)-acetyltransferase 25, NatB auxiliary subunit //	NAA25	<0.05	2.67356
14988254			<0.05	2.67349
14976735	XM_003362733 // LOC100630456 // lisH domain-containing protein FOPNL-like // --- // 100	LOC100630456	<0.05	2.6727
14988650	ENSECAT00000026571 // IMMT // inner membrane protein, mitochondrial // --- // 100067027	IMMT	<0.05	2.67264
15056135	ENSECAT00000019124 // LOC100058323 // general transcription factor IIE subunit 2-like /	LOC100058323	<0.05	2.6713
15112721			<0.05	2.67117
15109773			<0.05	2.67105
15004923	XM_001914963 // VPS36 // vacuolar protein sorting 36 homolog (S. cerevisiae) // --- //	VPS36	<0.05	2.6699
15110516	XM_001917928 // SNX19 // sorting nexin 19 // --- // 100072714 /// ENSECAT00000026141 //	SNX19	<0.05	2.66853
14925257	---		<0.05	2.66815
15092694	NM_001110308 // AGL // amylo-alpha-1, 6-glucosidase, 4-alpha-glucanotransferase // ---	AGL	<0.05	2.66815
15129738	XM_003365870 // LOC100629673 // malignant T cell-amplified sequence 1-like // --- // 10	LOC100629673	<0.05	2.66625
14992092	XM_001496971 // RMND5A // required for meiotic nuclear division 5 homolog A (S. cerevis	RMND5A	<0.05	2.66606
14932606	ENSECAT00000007095 // IKZF5 // IKAROS family zinc finger 5 (Pegasus) // --- // 10005813	IKZF5	<0.05	2.66605
14930015	---		<0.05	2.66544
14999682	ENSECAT00000005601 // PDE12 // phosphodiesterase 12 // --- // 100058532 /// XM_00148997	PDE12	<0.05	2.66514
15120493	ENSECAT00000008399 // CCBE1 // collagen and calcium binding EGF domains 1 // --- // ---	CCBE1	<0.05	2.66495
15011156	ENSECAT00000011350 // TRAK2 // trafficking protein, kinesin binding 2 // --- // 1000662	TRAK2	<0.05	2.66447
15097534	ENSECAT00000011075 // LOC100058940 // transmembrane protein 198-B-like // --- // 100058	LOC100058940	<0.05	2.66259
15068519	XM_001488157 // MFSD7 // major facilitator superfamily domain containing 7 // --- // 10	MFSD7	<0.05	2.66198
15111388	XM_001491421 // LOC100058462 // mediator of RNA polymerase II transcription subunit 17-	LOC100058462	<0.05	2.66173
15112763	XM_001504936 // BTBD10 // BTB (POZ) domain containing 10 // --- // 100056058 /// ENSECA	BTBD10	<0.05	2.66073
15041299	XM_001495344 // LOC100051702 // regulator of G-protein signaling 19-like // --- // 1000	LOC100051702	<0.05	2.65994
14964957	XM_001499941 // LOC100063689 // 26S proteasome non-ATPase regulatory subunit 12-like //	LOC100063689	<0.05	2.65974
15009273	XM_003363256 // PRPF40A // PRP40 pre-mRNA processing factor 40 homolog A (S. cerevisiae	PRPF40A	<0.05	2.65945
15089758	ENSECAT00000025442 // LOC100052650 // importin subunit alpha-4-like // --- // 100052650	LOC100052650	<0.05	2.6581
15003438	ENSECAT00000008076 // LOC100060926 // uncharacterized protein KIAA0564-like // --- // 1	LOC100060926	<0.05	2.65757
14986682	ENSECAT00000024822 // MAN2A1 // mannosidase, alpha, class 2A, member 1 // --- // 100064	MAN2A1	<0.05	2.65746
15047153	ENSECAT00000029087 // COX16 // COX16 cytochrome c oxidase assembly homolog (S. cerevisi	COX16	<0.05	2.65704
15133943	XM_001915515 // LOC100059443 // deoxyribonuclease-1-like 1-like // --- // 100059443 ///	LOC100059443	<0.05	2.65481
14939304	ENSECAT00000014987 // ATPBD4 // ATP binding domain 4 // --- // ---	ATPBD4	<0.05	2.65409
15021131	XM_001502916 // LOC100072858 // group XIIA secretory phospholipase A2-like // --- // 10	LOC100072858	<0.05	2.65404
14929829	---		<0.05	2.65396
15112116	ENSECAT00000006616 // TRIM21 // tripartite motif containing 21 // --- // 100066782 ///	TRIM21	<0.05	2.65386
14929857	---		<0.05	2.65324
15042068	XM_001495345 // SNAPC3 // small nuclear RNA activating complex, polypeptide 3, 50kDa //	SNAPC3	<0.05	2.65321
15129595	XM_001488038 // WDR44 // WD repeat domain 44 // --- // 100053726 /// ENSECAT00000013036	WDR44	<0.05	2.65283
15010915	ENSECAT00000021411 // PGAP1 // post-GPI attachment to proteins 1 // --- // 100070459 //	PGAP1	<0.05	2.64986
15062832	ENSECAT00000025866 // LOC100057616 // la-related protein 4B-like // --- // 100057616 //	LOC100057616	<0.05	2.64907
14937572	XM_001918045 // LOC100067089 // probable E3 ubiquitin-protein ligase HERC1-like // ---	LOC100067089	<0.05	2.64903
14994221	XM_001502813 // LOC100055747 // ras-related protein Rab-10-like // --- // 100055747 ///	LOC100055747	<0.05	2.64895
15024860	XM_001492944 // ENTPD4 // ectonucleoside triphosphate diphosphohydrolase 4 // --- // 10	ENTPD4	<0.05	2.64887
15079227	---		<0.05	2.64831
15044264	XM_001494419 // LOC100049903 // 26S protease regulatory subunit 10B-like // --- // 1000	LOC100049903	<0.05	2.6483
15072859	ENSECAT00000008955 // PIGG // phosphatidylinositol glycan anchor biosynthesis, class G	PIGG	<0.05	2.64783
14953461	XM_001488752 // LOC100053634 // cytochrome b-c1 complex subunit Rieske, mitochondrial-l	LOC100053634	<0.05	2.64703
15096670	XM_001488439 // IRAK4 // interleukin-1 receptor-associated kinase 4 // --- // 100054848	IRAK4	<0.05	2.64699
15059696	ENSECAT00000011568 // TBC1D22A // TBC1 domain family, member 22A // --- // 100049975 //	TBC1D22A	<0.05	2.64661
14972436	---		<0.05	2.64621
15044573	ENSECAT00000005976 // SLC38A6 // solute carrier family 38, member 6 // --- // 100060809	SLC38A6	<0.05	2.64481
15088124	XM_003365092 // DNTTIP2 // deoxynucleotidyltransferase, terminal, interacting protein 2	DNTTIP2	<0.05	2.6446
15029913	ENSECAT00000014717 // TPMT // thiopurine S-methyltransferase // --- // 100034066 /// NM	TPMT	<0.05	2.64438
14978914	XM_001499915 // LOC100060879 // UPF0458 protein C7orf42-like // --- // 100060879 /// EN	LOC100060879	<0.05	2.64202
15092278	ENSECAT00000009453 // CEPT1 // choline/ethanolamine phosphotransferase 1 // --- // 1000	CEPT1	<0.05	2.64193
15012635	XM_001916714 // SNX4 // sorting nexin 4 // --- // 100060493 /// ENSECAT00000009246 // S	SNX4	<0.05	2.64091
15071184	ENSECAT00000025549 // LOC100054658 // mps one binder kinase activator-like 1A-like // -	LOC100054658	<0.05	2.64063
14953936	ENSECAT00000026940 // LOC100066427 // delta(3,5)-Delta(2,4)-dienoyl-CoA isomerase, mito	LOC100066427	<0.05	2.64015
14938044	ENSECAT00000014788 // LOC100069498 // uncharacterized protein KIAA1370-like // --- // 1	LOC100069498	<0.05	2.63978
15064847	XM_001498913 // PLA2G15 // phospholipase A2, group XV // --- // 100053908 /// ENSECAT00	PLA2G15	<0.05	2.63955
15096977	XM_001504251 // LARP4 // La ribonucleoprotein domain family, member 4 // --- // 1000523	LARP4	<0.05	2.63901
15043180	ENSECAT00000027014 // LOC100056086 // structural maintenance of chromosomes protein 5-l	LOC100056086	<0.05	2.63811
15065741	ENSECAT00000010217 // LOC100630367 // microtubule-associated proteins 1A/1B light chain	LOC100630367	<0.05	2.63749

14928391	---		<0.05	2.63727
14928479	---		<0.05	2.63727
15017233	ENSECAT00000022402 // LOC100070183 // protein archease-like // --- // 100070183 /// XM_	LOC100070183	<0.05	2.6369
15004670	NM_001081835 // HMGB1 // high mobility group box 1 // --- // 100033873 /// AB275457 //	HMGB1	<0.05	2.63672
14999059	XM_001493888 // LOC100058184 // probable protein BRICK1-like // --- // 100058184 /// EN	LOC100058184	<0.05	2.63465
14953059	ENSECAT00000010921 // LOC100073006 // probable E3 ubiquitin-protein ligase RNF217-like	LOC100073006	<0.05	2.63432
15128447	ENSECAT00000025237 // IGBP1 // immunoglobulin (CD79A) binding protein 1 // --- // ---	IGBP1	<0.05	2.63277
15014134	XM_001496686 // PARL // presenilin associated, rhomboid-like // --- // 100058851 /// EN	PARL	<0.05	2.63254
15000944			<0.05	2.63106
15061101	XM_001499842 // LOC100070178 // SUN domain-containing protein 2-like // --- // 10007017	LOC100070178	<0.05	2.63051
15023603	XM_001504374 // LOC100071907 // PQ-loop repeat-containing protein 2-like // --- // 1000	LOC100071907	<0.05	2.63025
15127245	ENSECAT00000000582 // MBTPS2 // membrane-bound transcription factor peptidase, site 2 /	MBTPS2	<0.05	2.62921
15075619	XM_001497220 // FUCA2 // fucosidase, alpha-L- 2, plasma // --- // 100067102 /// ENSECAT	FUCA2	<0.05	2.6292
15135376	---		<0.05	2.62774
14986096	XM_001504442 // RAD50 // RAD50 homolog (S. cerevisiae) // --- // 100063198 /// ENSECAT0	RAD50	<0.05	2.62752
14994794	XM_001488818 // LOC100050214 // pre-mRNA-splicing factor ISY1 homolog // --- // 1000502	LOC100050214	<0.05	2.62735
15063055	XM_001495933 // YME1L1 // YME1-like 1 (S. cerevisiae) // --- // 100065311 /// ENSECAT00	YME1L1	<0.05	2.62577
15094937	ENSECAT00000017623 // LRRFIP1 // leucine rich repeat (in FLII) interacting protein 1 //	LRRFIP1	<0.05	2.62526
15004441	XM_001489625 // EFHA1 // EF-hand domain family, member A1 // --- // 100055452 /// ENSEC	EFHA1	<0.05	2.62495
15020540	ENSECAT00000025178 // OTUD4 // OTU domain containing 4 // --- // 100070999 /// XM_00150	OTUD4	<0.05	2.62484
14951584	---		<0.05	2.62357
15069886	XM_001502034 // LOC100069947 // m-phase phosphoprotein 6-like // --- // 100069947 /// E	LOC100069947	<0.05	2.62339
15028988	XM_001502478 // LOC100055574 // cell division cycle 5-like protein-like // --- // 10005	LOC100055574	<0.05	2.62329
14956862	XM_001501777 // PREP // prolyl endopeptidase // --- // 100071926 /// ENSECAT00000025507	PREP	<0.05	2.62312
14925223	---		<0.05	2.62148
15012829	XM_001502137 // LOC100060695 // coiled-coil domain-containing protein 58-like // --- //	LOC100060695	<0.05	2.62122
15007594	ENSECAT00000013076 // SSFA2 // sperm specific antigen 2 // --- // 100053992	SSFA2	<0.05	2.62099
14960950			<0.05	2.62081
15135030	---		<0.05	2.62077
14984544	XM_001498575 // LOC100068758 // eukaryotic translation initiation factor 4E type 1B-lik	LOC100068758	<0.05	2.62074
15038433	XM_001501184 // SLC9A8 // solute carrier family 9, subfamily A (NHE8, cation proton ant	SLC9A8	<0.05	2.61937
14948784	ENSECAT00000012263 // LOC100051031 // trafficking protein particle complex subunit 6B-l	LOC100051031	<0.05	2.61825
14965457	ENSECAT00000026227 // G6PC3 // glucose 6 phosphatase, catalytic, 3 // --- // 100064862	G6PC3	<0.05	2.61722
15028922			<0.05	2.61656
15011051	ENSECAT00000021847 // AOX1 // aldehyde oxidase 1 // --- // 100147243 /// ENSECAT0000002	AOX1	<0.05	2.61423
15131220	ENSECAT00000018502 // TAB3 // TGF-beta activated kinase 1/MAP3K7 binding protein 3 // -	TAB3	<0.05	2.6137
15116025	ENSECAT00000021625 // IMPACT // Impact homolog (mouse) // --- // ---	IMPACT	<0.05	2.61267
15091689	XM_001489373 // LOC100058935 // 28S ribosomal protein S21, mitochondrial-like // --- //	LOC100058935	<0.05	2.61193
14947054	XM_001503141 // TUBGCP4 // tubulin, gamma complex associated protein 4 // --- // 100056	TUBGCP4	<0.05	2.60939
15072301	ENSECAT00000008984 // CPEB2 // cytoplasmic polyadenylation element binding protein 2 //	CPEB2	<0.05	2.60897
15126634			<0.05	2.6087
15004969	ENSECAT00000015107 // LOC100054469 // ribonuclease H2 subunit B-like // --- // 10005446	LOC100054469	<0.05	2.60846
15090624	ENSECAT00000009997 // NCSTN // nicastrin // --- // 100058515 /// XM_001504431 // NCSTN	NCSTN	<0.05	2.60825
15133857	ENSECAT00000024419 // HCFC1 // host cell factor C1 (VP16-accessory protein) // --- // -	HCFC1	<0.05	2.60746
15033387	XM_001495595 // LOC100064807 // polyadenylate-binding protein-interacting protein 1-lik	LOC100064807	<0.05	2.60742
15120169	AB071949 // SMAD2 // SMAD family member 2 // --- // 100033843	SMAD2	<0.05	2.606
15092153	XM_001495327 // LRIG2 // leucine-rich repeats and immunoglobulin-like domains 2 // ---	LRIG2	<0.05	2.60581
15042677	ENSECAT00000008136 // LOC100063182 // putative DNA repair and recombination protein RAD	LOC100063182	<0.05	2.6057
14927767	---		<0.05	2.60455
14953805	ENSECAT00000024380 // ZNF91 // zinc finger protein 91 // --- // 100062418 /// XM_001493	ZNF91	<0.05	2.60432
14936123	ENSECAT00000025885 // LOC100069456 // AP-3 complex subunit sigma-2-like // --- // 10006	LOC100069456	<0.05	2.60413
15022161	XM_001916408 // LOC100067336 // nuclease-sensitive element-binding protein 1-like // --	LOC100067336	<0.05	2.60391
15002764	XM_001495151 // B3GALTL // beta 1,3-galactosyltransferase-like // --- // 100064175 ///	B3GALTL	<0.05	2.60319
14926375	---		<0.05	2.60297
15073653	ENSECAT00000008088 // PTPRC // protein tyrosine phosphatase, receptor type, C // --- //	PTPRC	<0.05	2.60261
14985663			<0.05	2.60237
15004807	XM_001495632 // LOC100062531 // protein FAM48A-like // --- // 100062531 /// ENSECAT0000	LOC100062531	<0.05	2.6011
15092355	XM_001495694 // AHCYL1 // adenosylhomocysteinase-like 1 // --- // 100058372 /// ENSECAT	AHCYL1	<0.05	2.60038
14989028	ENSECAT00000007059 // LOC100049885 // TP53RK-binding protein-like // --- // 100049885 /	LOC100049885	<0.05	2.60035
15012792	XM_001500212 // HSPBAP1 // HSPB (heat shock 27kDa) associated protein 1 // --- // 10007	HSPBAP1	<0.05	2.60002
15059038	XM_001501206 // EIF3L // eukaryotic translation initiation factor 3, subunit L // --- /	EIF3L	<0.05	2.59948
15097922	ENSECAT00000014187 // LOC100053518 // phosphatidylinositol-5-phosphate 4-kinase type-2	LOC100053518	<0.05	2.59853
15075233	---		<0.05	2.59822
14941009	ENSECAT00000026311 // LOC100051737 // serine/threonine-protein phosphatase 2A 55 kDa re	LOC100051737	<0.05	2.59681
14942165	XM_001500003 // LOC100060384 // sideroflexin-3-like // --- // 100060384 /// ENSECAT0000	LOC100060384	<0.05	2.59665
15065723	ENSECAT00000016408 // IRF8 // interferon regulatory factor 8 // --- // 100056218 /// XM	IRF8	<0.05	2.59623
14994518	ENSECAT00000016692 // KLF11 // Kruppel-like factor 11 // --- // 100072433	KLF11	<0.05	2.59437
15111336			<0.05	2.59362
14987398	ENSECAT00000016002 // POLK // polymerase (DNA directed) kappa // --- // 100065534 /// X	POLK	<0.05	2.59358
15016802	ENSECAT00000021978 // INPP5B // inositol polyphosphate-5-phosphatase, 75kDa // --- // 1	INPP5B	<0.05	2.59311
15034955	XM_001500904 // ATP13A1 // ATPase type 13A1 // --- // 100071205 /// ENSECAT00000022204	ATP13A1	<0.05	2.59309
15104370	ENSECAT00000018671 // LOC100630891 // adrenodoxin, mitochondrial-like // --- // 1006308	LOC100630891	<0.05	2.59255
15047222	XM_001489361 // NUMB // numb homolog (Drosophila) // --- // 100050235 /// XM_001489322	NUMB	<0.05	2.5922
14934134	---		<0.05	2.58953
15081362	ENSECAT00000018262 // KRIT1 // KRIT1, ankyrin repeat containing // --- // 100050998 ///	KRIT1	<0.05	2.58931
14986673	ENSECAT00000023748 // LOC100064546 // solute carrier family 25 member 46-like // --- //	LOC100064546	<0.05	2.58919
14975063	XM_001505046 // LOC100068657 // COP9 signalosome complex subunit 6-like // --- // 10006	LOC100068657	<0.05	2.58671
15121776	ENSECAT00000022027 // INTS8 // integrator complex subunit 8 // --- // 100055266	INTS8	<0.05	2.58625
15030618			<0.05	2.58534
15064965	XM_001497135 // SNTB2 // syntrophin, beta 2 (dystrophin-associated protein A1, 59kDa, b	SNTB2	<0.05	2.58434
15126186	ENSECAT00000011610 // LOC100064665 // transmembrane protein 19-like // --- // 100064665	LOC100064665	<0.05	2.5843
14961689	ENSECAT00000026830 // LOC100071540 // c-C motif chemokine 15-like // --- // 100071540 /	LOC100071540	<0.05	2.58292
14961158	XM_001917699 // LOC100070071 // luc7-like protein 3-like // --- // 100070071 /// ENSECA	LOC100070071	<0.05	2.58273
15020243	ENSECAT00000021241 // LOC100069500 // cathepsin O-like // --- // 100069500 /// XM_00149	LOC100069500	<0.05	2.58222
15130398	XM_001491684 // LOC100058868 // ATP-binding cassette sub-family D member 1-like // ---	LOC100058868	<0.05	2.58129
15045243	XM_001490424 // LOC100050909 // protein lin-52 homolog // --- // 100050909 /// ENSECAT0	LOC100050909	<0.05	2.58017
14997512	XM_001917517 // LOC100056767 // cytoplasmic dynein 1 light intermediate chain 1-like //	LOC100056767	<0.05	2.5796
15073553	XM_001490824 // TROVE2 // TROVE domain family, member 2 // --- // 100051140 /// ENSECAT	TROVE2	<0.05	2.57843
14931279	---		<0.05	2.57709
15087182	XM_001501227 // LOC100059997 // vesicle-trafficking protein SEC22b-like // --- // 10005	LOC100059997	<0.05	2.57612
14959829	XM_001490397 // ATXN7L3 // ataxin 7-like 3 // --- // 100064752 /// XM_003362543 // ATXN	ATXN7L3	<0.05	2.57596
14929325	---		<0.05	2.57524
15131052	ENSECAT00000026569 // CXorf23 // chromosome X open reading frame 23 // --- // ---	CXorf23	<0.05	2.57324
15063820	XM_001501531 // ZMYND11 // zinc finger, MYND-type containing 11 // --- // 100057731 ///	ZMYND11	<0.05	2.57275
14999463	XM_001493702 // GXYLT2 // glucoside xylosyltransferase 2 // --- // 100061938 /// ENSECA	GXYLT2	<0.05	2.57087
14925241	---		<0.05	2.57078
14941158	XM_001490606 // LOC100057158 // BRISC complex subunit Abro1-like // --- // 100057158 //	LOC100057158	<0.05	2.57023
15004279	XM_001499063 // CDC16 // cell division cycle 16 homolog (S. cerevisiae) // --- // 10006	CDC16	<0.05	2.56909
14954133	XM_001498028 // LOC100068145 // flavin reductase-like // --- // 100068145 /// ENSECAT00	LOC100068145	<0.05	2.56888
15068209	ENSECAT00000005488 // TBC1D14 // TBC1 domain family, member 14 // --- // 100070312 ///	TBC1D14	<0.05	2.56803
15026725	ENSECAT00000028958 // LOC100061589 // u11/U12 small nuclear ribonucleoprotein 48 kDa pr	LOC100061589	<0.05	2.56693
15034634	ENSECAT00000028973 // LOC100069519 // UPF0608 protein C19orf42 homolog // --- // 100069	LOC100069519	<0.05	2.56687
15049846	XM_001917182 // LOC100070437 // cyclin-dependent kinase 9-like // --- // 100070437 ///	LOC100070437	<0.05	2.56677
15088593	ENSECAT00000011906 // ZZZ3 // zinc finger, ZZ-type containing 3 // --- // 100053222 ///	ZZZ3	<0.05	2.56512
14968428			<0.05	2.5644
15012113	XM_001498641 // VPS8 // vacuolar protein sorting 8 homolog (S. cerevisiae) // --- // 10	VPS8	<0.05	2.56304

15062385 XM_001498178 // LOC100056170 // protein FAM188A-like // --- // 100056170 /// ENSECAT000	LOC100056170	<0.05	2.56273
15069781		<0.05	2.5624
15122053 ENSECAT00000010208 // DCAF13 // DDB1 and CUL4 associated factor 13 // --- // 100062586	DCAF13	<0.05	2.56222
15099390 XM_001500579 // LOC100057698 // NADH dehydrogenase [ubiquinone] 1 alpha subcomplex subu	LOC100057698	<0.05	2.56201
15121164 XM_001494625 // VCPIP1 // valosin containing protein (p97)/p47 complex interacting prot	VCPIP1	<0.05	2.56191
15130496 ---		<0.05	2.5618
14933451 ENSECAT00000007996 // CHUK // conserved helix-loop-helix ubiquitous kinase // --- // 10	CHUK	<0.05	2.56063
14975199 XM_001505059 // MOSPD3 // motile sperm domain containing 3 // --- // 100067442 /// ENSE	MOSPD3	<0.05	2.55958
15006197 XM_001504934 // AMMECR1L // AMME chromosomal region gene 1-like // --- // 100067484 ///	AMMECR1L	<0.05	2.55952
15005161 XM_001491491 // LOC100050971 // general transcription factor IIF subunit 2-like // ---	LOC100050971	<0.05	2.55746
15067652 ENSECAT00000004810 // RBM47 // RNA binding motif protein 47 // --- // ---	RBM47	<0.05	2.55606
14925637 ---		<0.05	2.55591
14945899 ENSECAT00000006078 // PML // promyelocytic leukemia // --- // 100062744 /// XM_00149324	PML	<0.05	2.55582
15086701 NM_001163867 // S100A10 // S100 calcium binding protein A10 // --- // 100034012 /// ENS	S100A10	<0.05	2.55396
15030268 XM_001492536 // TRIM27 // tripartite motif containing 27 // --- // 100060192 /// ENSECA	TRIM27	<0.05	2.55382
15069779 XM_003364638 // KARS // lysyl-tRNA synthetase // --- // 100055335 /// XM_001501578 // K	KARS	<0.05	2.55331
14932744 XM_001496462 // EIF3A // eukaryotic translation initiation factor 3, subunit A // --- /	EIF3A	<0.05	2.5526
15066743 XM_001916000 // LOC100051138 // nuclear pore complex protein Nup54-like // --- // 10005	LOC100051138	<0.05	2.55236
14957549 ENSECAT00000026671 // MED23 // mediator complex subunit 23 // --- // 100067556	MED23	<0.05	2.55131
15044384 XM_001491495 // LOC100058576 // uncharacterized LOC100058576 // --- // 100058576 /// EN	LOC100058576	<0.05	2.55018
15064558 XM_001496846 // LOC100065750 // UPF0183 protein C16orf70-like // --- // 100065750 /// E	LOC100065750	<0.05	2.55004
14991186 ENSECAT00000005716 // LOC100072837 // 1,2-dihydroxy-3-keto-5-methylthiopentene dioxygen	LOC100072837	<0.05	2.54971
15121816 ENSECAT00000017216 // LOC100059691 // phosphatidylserine synthase 1-like // --- // 1000	LOC100059691	<0.05	2.54899
14950206 XM_001499336 // LOC100068274 // u1 small nuclear ribonucleoprotein A-like // --- // 100	LOC100068274	<0.05	2.548
15000943 ENSECAT00000028989 // LOC100067962 // 60S ribosomal protein L14-like // --- // 10006796	LOC100067962	<0.05	2.54787
15026455 ENSECAT00000006504 // LOC100073027 // aminoacyl tRNA synthase complex-interacting multi	LOC100073027	<0.05	2.54764
15056407 ENSECAT00000020946 // LOC100057615 // UPF0501 protein KIAA1430 homolog // --- // 100057	LOC100057615	<0.05	2.54755
15128873 ENSECAT00000002701 // LOC100071709 // peroxiredoxin-1-like // --- // 100071709 /// XM_0	LOC100071709	<0.05	2.54671
14982994 ENSECAT00000013217 // LOC100062974 // voltage-dependent anion-selective channel protein	LOC100062974	<0.05	2.5465
15075942 XM_001499464 // PHF10 // PHD finger protein 10 // --- // 100049916 /// ENSECAT000000202	PHF10	<0.05	2.54607
15047924 ENSECAT00000016559 // LOC100053162 // ataxin-3-like // --- // 100053162 /// XM_00149703	LOC100053162	<0.05	2.54549
15006926 ENSECAT00000016745 // MARCH7 // membrane-associated ring finger (C3HC4) 7, E3 ubiquitin	Mar-07	<0.05	2.54475
15013371 AY246739 // ZBTB11 // zinc finger and BTB domain containing 11 // --- // 100072181 ///	ZBTB11	<0.05	2.54391
15060742		<0.05	2.54375
14988191 XM_001492041 // LOC100051330 // VIP36-like protein-like // --- // 100051330 /// ENSECAT	LOC100051330	<0.05	2.54368
15087571 XM_001498901 // ST7L // suppression of tumorigenicity 7 like // --- // 100059051 /// EN	ST7L	<0.05	2.54333
14930003 ---		<0.05	2.5415
15127896 ---		<0.05	2.54141
14980147 ENSECAT00000003256 // MKL2 // MKL/myocardin-like 2 // --- // ---	MKL2	<0.05	2.54044
15111156 ENSECAT00000014969 // ICAM1 // intercellular adhesion molecule 1 // --- // ---	ICAM1	<0.05	2.53897
15097390 ENSECAT00000016276 // NCKAP1L // NCK-associated protein 1-like // --- // 100062541	NCKAP1L	<0.05	2.53866
14932115 ---		<0.05	2.53835
15091950 ENSECAT00000021179 // MAN1A2 // mannosidase, alpha, class 1A, member 2 // --- // --- //	MAN1A2	<0.05	2.53798
15060461 XM_001496669 // UHRF1BP1L // UHRF1 binding protein 1-like // --- // 100066380 /// ENSEC	UHRF1BP1L	<0.05	2.53777
15074200 XM_001489242 // LOC100054462 // histone H3.3-like // --- // 100054462 /// ENSECAT000000	LOC100054462	<0.05	2.53547
14998509 XM_003363113 // SELT // selenoprotein T // --- // 100057206 /// ENSECAT00000027127 // S	SELT	<0.05	2.53518
14983452 ENSECAT00000007807 // LOC100073183 // receptor expression-enhancing protein 5-like // -	LOC100073183	<0.05	2.53501
15089612 ENSECAT00000014284 // RFWD2 // ring finger and WD repeat domain 2, E3 ubiquitin protein	RFWD2	<0.05	2.53459
14932655 ENSECAT00000012836 // LOC100064881 // arginyl-tRNA--protein transferase 1-like // --- /	LOC100064881	<0.05	2.53392
14956511 XM_001500697 // RRAGD // Ras-related GTP binding D // --- // 100071018 /// ENSECAT00000	RRAGD	<0.05	2.53313
14979289 EU881921 // ITGAL // integrin, alpha L (antigen CD11A (p180), lymphocyte function-assoc	ITGAL	<0.05	2.5328
15105694 ENSECAT00000021327 // DNAJB1 // DnaJ (Hsp40) homolog, subfamily B, member 1 // --- // -	DNAJB1	<0.05	2.53241
15091393 ENSECAT00000021710 // LOC100056434 // SNARE-associated protein Snapin-like // --- // 10	LOC100056434	<0.05	2.53203
14957406 XM_001503134 // LOC100073017 // HD domain-containing protein 2-like // --- // 100073017	LOC100073017	<0.05	2.53146
15126677 XM_001500534 // LOC100070853 // leucine-rich repeat-containing protein 58-like // --- /	LOC100070853	<0.05	2.53112
15073535 NM_001081939 // RGS1 // regulator of G-protein signaling 1 // --- // 100034151 /// ENSE	RGS1	<0.05	2.53093
15028924		<0.05	2.53084
15121653 XM_001488342 // LOC100052944 // OTU domain-containing protein 6B-like // --- // 1000529	LOC100052944	<0.05	2.53047
15069839 ENSECAT00000029000 // LOC100629755 // glycine cleavage system H protein, mitochondrial-	LOC100629755	<0.05	2.52973
15004871 ENSECAT00000018650 // LOC100066403 // uncharacterized protein C13orf23-like // --- // 1	LOC100066403	<0.05	2.52849
15082309 ENSECAT00000012449 // THAP5 // THAP domain containing 5 // --- // 100070659 /// XM_0015	THAP5	<0.05	2.52637
15071656 ENSECAT00000025177 // LOC100061309 // SLAIN motif-containing protein 2-like // --- // 1	LOC100061309	<0.05	2.52612
14992990 XM_001495848 // VRK2 // vaccinia related kinase 2 // --- // 100052238 /// ENSECAT000000	VRK2	<0.05	2.52579
14949449 XM_001915953 // LOC100059603 // transmembrane protein 147-like // --- // 100059603 ///	LOC100059603	<0.05	2.52574
14969531 XM_001489510 // TTC17 // tetratricopeptide repeat domain 17 // --- // 100055237 /// ENS	TTC17	<0.05	2.52409
14937462 XM_001498202 // CLPX // ClpX caseinolytic peptidase X homolog (E. coli) // --- // 10005	CLPX	<0.05	2.52409
15091101 XM_001499126 // LOC100057427 // 28S ribosomal protein S29, mitochondrial-like // --- //	LOC100057427	<0.05	2.52363
15028907 ENSECAT00000014451 // TMEM63B // transmembrane protein 63B // --- // 100067695	TMEM63B	<0.05	2.52336
14990912 ENSECAT00000024027 // SMC6 // structural maintenance of chromosomes 6 // --- // 1000720	SMC6	<0.05	2.52279
15131421 ---		<0.05	2.52208
14947663 XM_001918315 // SLC12A6 // solute carrier family 12 (potassium/chloride transporters),	SLC12A6	<0.05	2.52139
15123833 XM_001489304 // LOC100055169 // protein virilizer homolog // --- // 100055169 /// ENSEC	LOC100055169	<0.05	2.52079
14982984 XM_001504404 // LOC100062946 // s-phase kinase-associated protein 1-like // --- // 1000	LOC100062946	<0.05	2.51976
15096021 XM_001501444 // DDX47 // DEAD (Asp-Glu-Ala-Asp) box polypeptide 47 // --- // 100063569	DDX47	<0.05	2.51906
14985439 XM_003362796 // LOC100061029 // transcription elongation regulator 1-like // --- // 100	LOC100061029	<0.05	2.51871
14943332 XM_001502798 // LOC100072758 // uncharacterized LOC100072758 // --- // 100072758 /// EN	LOC100072758	<0.05	2.51862
15069337 XM_001498476 // LOC100066381 // proteasome subunit beta type-10-like // --- // 10006638	LOC100066381	<0.05	2.5186
14978073 ENSECAT00000017355 // ZNF12 // zinc finger protein 12 // --- // --- /// ENSECAT00000017	ZNF12	<0.05	2.51703
15011129 ENSECAT00000015574 // LOC100067910 // protein FAM126B-like // --- // 100067910 /// XM_0	LOC100067910	<0.05	2.51668
15043871 XM_001498912 // TOPORS // topoisomerase I binding, arginine/serine-rich, E3 ubiquitin p	TOPORS	<0.05	2.51642
15080173 ENSECAT00000009183 // PTPN12 // protein tyrosine phosphatase, non-receptor type 12 // -	PTPN12	<0.05	2.51565
14966407 ---		<0.05	2.51545
14937499 XM_001498388 // LOC100053578 // maspardin-like // --- // 100053578 /// ENSECAT000000135	LOC100053578	<0.05	2.51497
15050157 XM_001499731 // LOC100070047 // serine/threonine-protein phosphatase 2A activator-like	LOC100070047	<0.05	2.5148
15009979 XM_001495258 // SP3 // Sp3 transcription factor // --- // 100064317 /// ENSECAT00000014	SP3	<0.05	2.51469
15001456 ENSECAT00000022248 // LOC100058489 // COX assembly mitochondrial protein homolog // ---	LOC100058489	<0.05	2.51449
14941556 ENSECAT00000012003 // FAM160B1 // family with sequence similarity 160, member B1 // ---	FAM160B1	<0.05	2.51436
15055157 XM_001494782 // GCFC1 // GC-rich sequence DNA-binding factor 1 // --- // 100063627 ///	GCFC1	<0.05	2.51397
15104341 XM_001499568 // DDX10 // DEAD (Asp-Glu-Ala-Asp) box polypeptide 10 // --- // 100069845	DDX10	<0.05	2.5139
14948567 ENSECAT00000017428 // HEATR5A // HEAT repeat containing 5A // --- // ---	HEATR5A	<0.05	2.51331
15054879 XM_001496687 // LOC100066408 // 39S ribosomal protein L39, mitochondrial-like // --- //	LOC100066408	<0.05	2.51242
14989361 ENSECAT00000018270 // CCT4 // chaperonin containing TCP1, subunit 4 (delta) // --- // 1	CCT4	<0.05	2.50754
14930137 ---		<0.05	2.50682
15093263 XM_001498098 // LOC100053123 // dnaJ homolog subfamily B member 4-like // --- // 100053	LOC100053123	<0.05	2.50681
15023353 ENSECAT00000024594 // NIPAL3 // NIPA-like domain containing 3 // --- // 100071495 /// X	NIPAL3	<0.05	2.50653
15003376 ENSECAT00000013896 // NUFIP1 // nuclear fragile X mental retardation protein interactin	NUFIP1	<0.05	2.50538
15037475 XM_001916721 // ITCH // itchy E3 ubiquitin protein ligase // --- // 100069340 /// ENSEC	ITCH	<0.05	2.50496
15070936 ENSECAT00000026405 // LOC100059167 // 39S ribosomal protein L1, mitochondrial-like // -	LOC100059167	<0.05	2.50484
15031760 XM_001502158 // LOC100055455 // 28S ribosomal protein S18a, mitochondrial-like // --- /	LOC100055455	<0.05	2.50312
15122229 ENSECAT00000009942 // LOC100064267 // receptor-binding cancer antigen expressed on SiSo	LOC100064267	<0.05	2.50209
15062469 XM_001498475 // DCLRE1C // DNA cross-link repair 1C // --- // 100056337 /// ENSECAT0000	DCLRE1C	<0.05	2.50127
15020371 XM_001501357 // PET112 // PET112 homolog (yeast) // --- // 100062330 /// ENSECAT0000000	PET112	<0.05	2.50111
15022499 XM_001503634 // THRAP3 // thyroid hormone receptor associated protein 3 // --- // 10005	THRAP3	<0.05	2.50109
15109144 ENSECAT00000020594 // ELAVL1 // ELAV (embryonic lethal, abnormal vision, Drosophila)-li	ELAVL1	<0.05	2.49955
15060860		<0.05	2.49906
15129695 XM_001914705 // LOC100058718 // membrane-associated progesterone receptor component 1-l	LOC100058718	<0.05	2.49728

15056121	ENSECAT00000015216 // LOC100062365 // glutathione reductase, mitochondrial-like // ---	LOC100062365	<0.05	2.4971
15082596	ENSECAT00000016823 // LOC100071546 // uncharacterized LOC100071546 // --- // 100071546	LOC100071546	<0.05	2.49686
15007144	XM_001916938 // UBR3 // ubiquitin protein ligase E3 component n-recognin 3 (putative) /	UBR3	<0.05	2.49651
14965825	ENSECAT00000021406 // LOC100067814 // protein CASC3-like // --- // 100067814 /// XM_001	LOC100067814	<0.05	2.4965
15121849	ENSECAT00000011528 // MTDH // metadherin // --- // 100059885 /// XM_001492316 // MTDH /	MTDH	<0.05	2.49571
15031248	ENSECAT00000017692 // LOC100053260 // transcription initiation factor TFIID subunit 11-	LOC100053260	<0.05	2.49495
14979358	XM_001496402 // LOC100065972 // BTB/POZ domain-containing adapter for CUL3-mediated Rho	LOC100065972	<0.05	2.49313
15098273	ENSECAT00000014580 // LOC100052084 // e3 ubiquitin-protein ligase Mdm2-like // --- // 1	LOC100052084	<0.05	2.49263
15085585	ENSECAT00000014997 // LOC100065940 // succinate dehydrogenase cytochrome b560 subunit,	LOC100065940	<0.05	2.49204
15016333	XM_001496034 // LOC100052421 // peroxiredoxin-1-like // --- // 100052421 /// ENSECAT000	LOC100052421	<0.05	2.49076
15076754	XM_001491503 // CBLL1 // Cbl proto-oncogene, E3 ubiquitin protein ligase-like 1 // ---	CBLL1	<0.05	2.48979
14995366	XM_001498639 // UBA3 // ubiquitin-like modifier activating enzyme 3 // --- // 100053395	UBA3	<0.05	2.48817
14925503	---		<0.05	2.48801
14957087	ENSECAT00000028978 // LOC100072399 // DNA polymerase zeta catalytic subunit-like // ---	LOC100072399	<0.05	2.488
14997562	XM_001493003 // AZI2 // 5-azacytidine induced 2 // --- // 100051113 /// ENSECAT00000021	AZI2	<0.05	2.4874
15010479	ENSECAT00000012633 // SESTD1 // SEC14 and spectrin domains 1 // --- // 100067450 /// XM	SESTD1	<0.05	2.48672
15025647	ENSECAT00000017805 // ARHGAP10 // Rho GTPase activating protein 10 // --- // 100070647	ARHGAP10	<0.05	2.48615
15035671	XM_001499068 // LOC100053208 // prostaglandin E2 receptor EP4 subtype-like // --- // 10	LOC100053208	<0.05	2.4849
14981305	XM_003362894 // MAPK9 // mitogen-activated protein kinase 9 // --- // 100057961 /// ENS	MAPK9	<0.05	2.4845
15100051	XM_001496567 // LOC100052330 // inhibitor of growth protein 4-like // --- // 100052330	LOC100052330	<0.05	2.4845
15121047	---		<0.05	2.48363
15041634	ENSECAT00000016395 // LOC100063011 // uncharacterized LOC100063011 // --- // 100063011	LOC100063011	<0.05	2.48345
15032879	ENSECAT00000011373 // UPF1 // UPF1 regulator of nonsense transcripts homolog (yeast) //	UPF1	<0.05	2.48327
15090080	ENSECAT00000023764 // SMYD2 // SET and MYND domain containing 2 // --- // 100052774 ///	SMYD2	<0.05	2.48314
14940616	---		<0.05	2.48288
15042382	XM_001499262 // NFX1 // nuclear transcription factor, X-box binding 1 // --- // 1000537	NFX1	<0.05	2.48273
15106807	ENSECAT00000003443 // INTS4 // integrator complex subunit 4 // --- // ---	INTS4	<0.05	2.4822
15030027	ENSECAT00000015105 // LOC100067631 // tyrosyl-DNA phosphodiesterase 2-like // --- // 10	LOC100067631	<0.05	2.4807
15135944	---		<0.05	2.47953
14965512	XM_001491967 // LOC100065621 // transmembrane protein 106A-like // --- // 100065621 ///	LOC100065621	<0.05	2.47868
14965653	XM_001493847 // LOC100052586 // tubulin gamma-1 chain-like // --- // 100052586 /// ENSE	LOC100052586	<0.05	2.4775
15049718	XM_001501758 // ZBTB43 // zinc finger and BTB domain containing 43 // --- // 100067188	ZBTB43	<0.05	2.47708
14928133	---		<0.05	2.47705
14978031	ENSECAT00000013878 // DAGLB // diacylglycerol lipase, beta // --- // 100061701 /// XM_0	DAGLB	<0.05	2.47677
14987167	XM_001504645 // ZFYVE16 // zinc finger, FYVE domain containing 16 // --- // 100065363 /	ZFYVE16	<0.05	2.47649
15078229	ENSECAT00000020747 // AVL9 // AVL9 homolog (S. cerevisiase) // --- // 100070105 /// XM_	AVL9	<0.05	2.47645
15104949	ENSECAT00000005112 // CBL // Cbl proto-oncogene, E3 ubiquitin protein ligase // --- //	CBL	<0.05	2.47557
15113604	XM_001496455 // PRR14L // proline rich 14-like // --- // 100062987 /// ENSECAT000000236	PRR14L	<0.05	2.47512
14929689	---		<0.05	2.47495
14937396	ENSECAT00000020284 // LOC100053141 // UPF0464 protein C15orf44 homolog // --- // 100053	LOC100053141	<0.05	2.47462
14968442	ENSECAT00000011216 // LOC100061712 // G protein pathway suppressor 2-like // --- // 100	LOC100061712	<0.05	2.47312
15072130	ENSECAT00000008585 // PI4K2B // phosphatidylinositol 4-kinase type 2 beta // --- // ---	PI4K2B	<0.05	2.47059
14989934	XM_001499916 // LOC100053736 // cytochrome c oxidase subunit 7A-related protein, mitoch	LOC100053736	<0.05	2.47018
14985679			<0.05	2.46999
15096112	XM_001497473 // LOC100067410 // serine-threonine kinase receptor-associated protein-lik	LOC100067410	<0.05	2.46993
15004936	XM_001487974 // ALG11 // asparagine-linked glycosylation 11, alpha-1,2-mannosyltransfer	ALG11	<0.05	2.46987
15067265	DQ988040 // CLOCK // clock homolog (mouse) // --- // 100034166	CLOCK	<0.05	2.46931
15081633	XM_003364859 // LOC100629458 // NADH dehydrogenase [ubiquinone] 1 alpha subcomplex subu	LOC100629458	<0.05	2.46768
14993407	XM_003363001 // EML4 // echinoderm microtubule associated protein like 4 // --- // 1000	EML4	<0.05	2.46717
14927901	---		<0.05	2.46631
15094491	ENSECAT00000023767 // SP140 // SP140 nuclear body protein // --- // ---	SP140	<0.05	2.46585
14956463	XM_001503749 // RNGTT // RNA guanylyltransferase and 5'-phosphatase // --- // 100065700	RNGTT	<0.05	2.46456
15043894	ENSECAT00000012966 // LOC100068209 // beta-1,4-galactosyltransferase 1-like // --- // 1	LOC100068209	<0.05	2.46399
14929351	---		<0.05	2.46366
14927113	---		<0.05	2.46337
14925657	---		<0.05	2.46288
15011908	ENSECAT00000020156 // LOC100058922 // translation initiation factor eIF-2B subunit epsi	LOC100058922	<0.05	2.46214
15035258	XM_001493976 // KIF2A // kinesin heavy chain member 2A // --- // 100050982 /// ENSECAT0	KIF2A	<0.05	2.46195
15087064	ENSECAT00000002893 // LOC100065233 // peroxisomal membrane protein 11B-like // --- // 1	LOC100065233	<0.05	2.46181
14988632	XM_001497977 // RNF103 // ring finger protein 103 // --- // 100052907 /// ENSECAT000000	RNF103	<0.05	2.46138
14954951	XM_001488542 // LOC100050944 // ER lumen protein retaining receptor 1-like // --- // 10	LOC100050944	<0.05	2.46084
15057003	ENSECAT00000010688 // BAG4 // BCL2-associated athanogene 4 // --- // 100057730 /// XM_0	BAG4	<0.05	2.46059
14933286	ENSECAT00000021996 // LOC100070070 // UPF0668 protein C10orf76-like // --- // 100070070	LOC100070070	<0.05	2.46022
15126678			<0.05	2.45963
14962593	ENSECAT00000010791 // LOC100072797 // putative ATP-dependent RNA helicase DHX33-like //	LOC100072797	<0.05	2.45938
15073960	XM_003364550 // LOC100630213 // protein FAM36A-like // --- // 100630213 /// ENSECAT0000	LOC100630213	<0.05	2.45903
14977169	ENSECAT00000026215 // ADCY9 // adenylate cyclase 9 // --- // 100066046 /// XM_001502248	ADCY9	<0.05	2.45836
14999577	XM_001489012 // LOC100050505 // BTB/POZ domain-containing protein KCTD6-like // --- //	LOC100050505	<0.05	2.4575
14932969	ENSECAT00000014008 // ZDHHC6 // zinc finger, DHHC-type containing 6 // --- // ---	ZDHHC6	<0.05	2.45623
14995904	XM_001492187 // RFT1 // RFT1 homolog (S. cerevisiae) // --- // 100059676 /// ENSECAT000	RFT1	<0.05	2.45599
15045459	XM_001493748 // AHSA1 // AHA1, activator of heat shock 90kDa protein ATPase homolog 1 (	AHSA1	<0.05	2.45377
14943290	XM_001502747 // LOC100072720 // inorganic pyrophosphatase-like // --- // 100072720 ///	LOC100072720	<0.05	2.45306
15056840	XM_001490279 // LOC100050524 // golgin subfamily A member 7-like // --- // 100050524 //	LOC100050524	<0.05	2.45279
14957654	XM_003362372 // LOC100073151 // serine/threonine-protein kinase Sgk1-like // --- // 100	LOC100073151	<0.05	2.45258
15061257	ENSECAT00000012509 // LOC100070677 // PPPDE peptidase domain-containing protein 2-like	LOC100070677	<0.05	2.45228
15099226	XM_001495779 // USP40 // ubiquitin specific peptidase 40 // --- // 100065074 /// ENSECA	USP40	<0.05	2.45072
15133639	NR_033102 // MIR505 // microRNA mir-505 // --- // 100315095	MIR505	<0.05	2.45019
15026054	ENSECAT00000020736 // LOC100063889 // uncharacterized protein KIAA1109-like // --- // 1	LOC100063889	<0.05	2.44996
14944547	XM_001496540 // LOC100066171 // UPF0765 protein C10orf58-like // --- // 100066171 /// E	LOC100066171	<0.05	2.44987
15078776	ENSECAT00000010372 // IRF5 // interferon regulatory factor 5 // --- // 100071786 /// XM	IRF5	<0.05	2.44941
15117585	XM_003365448 // DRG1 // developmentally regulated GTP binding protein 1 // --- // 10005	DRG1	<0.05	2.44896
14979627	XM_001501020 // LOC100068373 // dynactin subunit 5-like // --- // 100068373 /// ENSECAT	LOC100068373	<0.05	2.44861
14936941	XM_001493806 // LOC100062111 // secretory carrier-associated membrane protein 2-like //	LOC100062111	<0.05	2.44827
15001139	ENSECAT00000002299 // LOC100053940 // myeloid differentiation primary response protein	LOC100053940	<0.05	2.44737
14934326	ENSECAT00000014702 // SIRT1 // sirtuin 1 // --- // 100072571 /// XM_001502550 // SIRT1	SIRT1	<0.05	2.44712
14961325	XM_001503426 // LOC100070859 // dynein light chain 2, cytoplasmic-like // --- // 100070	LOC100070859	<0.05	2.447
15129726	ENSECAT00000007209 // ZBTB33 // zinc finger and BTB domain containing 33 // --- // 1000	ZBTB33	<0.05	2.44559
14929591	---		<0.05	2.44543
15036485	ENSECAT00000023884 // LOC100050228 // crooked neck-like protein 1-like // --- // 100050	LOC100050228	<0.05	2.44488
15024898	ENSECAT00000012264 // LOC100059300 // BTB/POZ domain-containing protein KCTD9-like // -	LOC100059300	<0.05	2.44483
15026806	ENSECAT00000008100 // LOC100063959 // transmembrane protein 170B-like // --- // 1000639	LOC100063959	<0.05	2.4425
15053877	XM_001498235 // IFNAR2 // interferon (alpha, beta and omega) receptor 2 // --- // 10005	IFNAR2	<0.05	2.44186
15097539	XM_003365259 // LOC100051662 // diacylglycerol kinase alpha-like // --- // 100051662 //	LOC100051662	<0.05	2.44173
15109442	ENSECAT00000026255 // CASP1 // caspase 1, apoptosis-related cysteine peptidase // --- /	CASP1	<0.05	2.44171
15025876	ENSECAT00000025577 // LOC100063562 // UPF0462 protein C4orf33-like // --- // 100063562	LOC100063562	<0.05	2.44003
15001668	ENSECAT00000017594 // MRPL3 // mitochondrial ribosomal protein L3 // --- // 100052750 /	MRPL3	<0.05	2.43918
15000886	ENSECAT00000015724 // TRAK1 // trafficking protein, kinesin binding 1 // --- // 1000676	TRAK1	<0.05	2.43711
15007296	XM_001494902 // LOC100063802 // histone acetyltransferase type B catalytic subunit-like	LOC100063802	<0.05	2.43658
15014349	XM_001499782 // BCL6 // B-cell CLL/lymphoma 6 // --- // 100059642 /// ENSECAT0000000568	BCL6	<0.05	2.43638
14930133	---		<0.05	2.43629
15020785	ENSECAT00000026349 // PGRMC2 // progesterone receptor membrane component 2 // --- // --	PGRMC2	<0.05	2.43591
14936714	ENSECAT00000018244 // IREB2 // iron-responsive element binding protein 2 // --- // 1000	IREB2	<0.05	2.43545
15047234	---		<0.05	2.43499
14987651	XM_001504043 // GTF2H2 // general transcription factor IIH, polypeptide 2, 44kDa // ---	GTF2H2	<0.05	2.43305
15012935	XM_001502372 // LOC100060962 // rab-like protein 3-like // --- // 100060962 /// ENSECAT	LOC100060962	<0.05	2.43259
15007540	XM_001917545 // LOC100067359 // pleckstrin homology domain-containing family A member 3	LOC100067359	<0.05	2.43224

14928395	---		<0.05	2.4317
14928483	---		<0.05	2.4317
15091575	XM_001491319 // PRUNE // prune homolog (Drosophila) // --- // 100055127 /// ENSECAT0000	PRUNE	<0.05	2.43161
14945585	XM_001487863 // LOC100049875 // AN1-type zinc finger protein 6-like // --- // 100049875	LOC100049875	<0.05	2.43097
14952991	XM_001504161 // LOC100067138 // nogo-B receptor-like // --- // 100067138 /// ENSECAT000	LOC100067138	<0.05	2.43086
15052994	XM_001498768 // LOC100069513 // mediator of RNA polymerase II transcription subunit 27-	LOC100069513	<0.05	2.4308
15090760	---		<0.05	2.43077
15071764	XM_001496906 // LOC100054802 // transmembrane protein 33-like // --- // 100054802 /// E	LOC100054802	<0.05	2.43029
15000469	XM_001498598 // ARIH2 // ariadne homolog 2 (Drosophila) // --- // 100053691 /// ENSECAT	ARIH2	<0.05	2.43017
15127085	XM_001490045 // ZRSR2 // zinc finger (CCCH type), RNA-binding motif and serine/arginine	ZRSR2	<0.05	2.42996
15044098	ENSECAT00000017002 // SECISBP2 // SECIS binding protein 2 // --- // ---	SECISBP2	<0.05	2.42984
15133068	XM_001914996 // LOC100058559 // regulator of nonsense transcripts 3B-like // --- // 100	LOC100058559	<0.05	2.42979
14959726	XM_001495314 // LOC100064401 // coiled-coil domain-containing protein 43-like // --- //	LOC100064401	<0.05	2.42975
15062601	XM_001916725 // KIN // KIN, antigenic determinant of recA protein homolog (mouse) // --	KIN	<0.05	2.42941
15095342	ENSECAT00000026686 // FBXL14 // F-box and leucine-rich repeat protein 14 // --- // ---	FBXL14	<0.05	2.42922
15016514	XM_001498548 // LOC100053305 // elongation of very long chain fatty acids protein 1-lik	LOC100053305	<0.05	2.4285
15062525	NM_001190419 // SEPHS1 // selenophosphate synthetase 1 // --- // 100056604 /// ENSECAT0	SEPHS1	<0.05	2.42675
15121697	ENSECAT00000007598 // LOC100051849 // protein FAM92A1-like // --- // 100051849 /// XM_0	LOC100051849	<0.05	2.42596
14928673	---		<0.05	2.42553
15097636	ENSECAT00000014392 // ESYT1 // extended synaptotagmin-like protein 1 // --- // 10005197	ESYT1	<0.05	2.42484
14985672			<0.05	2.42467
15011264	XM_001505112 // LOC100067464 // ribosome biogenesis protein WDR12-like // --- // 100067	LOC100067464	<0.05	2.42456
14984053	XM_001504661 // WDR41 // WD repeat domain 41 // --- // 100065484 /// ENSECAT00000009889	WDR41	<0.05	2.42422
15044375	ENSECAT00000019007 // MUDENG // adaptor-related protein complex 5, mu 1 subunit // ---	MUDENG	<0.05	2.42411
15132251	XM_001502685 // LOC100072678 // transcriptional regulator ATRX-like // --- // 100072678	LOC100072678	<0.05	2.42305
14993934	XM_001502144 // LOC100055055 // GPN-loop GTPase 1-like // --- // 100055055 /// ENSECAT0	LOC100055055	<0.05	2.4225
14960117	XM_001494956 // LOC100053004 // ras-related protein Rab-5C-like // --- // 100053004 ///	LOC100053004	<0.05	2.42155
15116337	XM_001497768 // PIK3C3 // phosphoinositide-3-kinase, class 3 // --- // 100053039 /// EN	PIK3C3	<0.05	2.42131
15026664	XM_001490025 // RREB1 // ras responsive element binding protein 1 // --- // 100050903 /	RREB1	<0.05	2.42084
15017401	---		<0.05	2.42049
14930125	---		<0.05	2.41978
15110764	ENSECAT00000026537 // LOC100146594 // UV excision repair protein RAD23 homolog A-like /	LOC100146594	<0.05	2.41896
15041646	ENSECAT00000014654 // LOC100064023 // UPF0586 protein C9orf41 homolog // --- // 1000640	LOC100064023	<0.05	2.41863
15039937	XM_001501271 // PIGU // phosphatidylinositol glycan anchor biosynthesis, class U // ---	PIGU	<0.05	2.41849
15055592	XM_003364189 // LOC100630015 // uncharacterized LOC100630015 // --- // 100630015 /// EN	LOC100630015	<0.05	2.41788
15008186	XM_001497246 // NOP58 // NOP58 ribonucleoprotein homolog (yeast) // --- // 100067129 //	NOP58	<0.05	2.41755
15120138	ENSECAT00000004441 // PIAS2 // protein inhibitor of activated STAT, 2 // --- // 1000688	PIAS2	<0.05	2.41717
15068545	XM_001490225 // VPS35 // vacuolar protein sorting 35 homolog (S. cerevisiae) // --- //	VPS35	<0.05	2.41695
15019492	XM_001490720 // LOC100057357 // serine/threonine-protein phosphatase 2B catalytic subun	LOC100057357	<0.05	2.4161
14948273	XM_001490602 // LOC100051729 // proteasome activator complex subunit 2-like // --- // 1	LOC100051729	<0.05	2.41607
14945741	XM_001493197 // LOC100061150 // tetraspanin-3-like // --- // 100061150 /// ENSECAT00000	LOC100061150	<0.05	2.41577
14937555	ENSECAT00000010583 // FAM96A // family with sequence similarity 96, member A // --- //	FAM96A	<0.05	2.4152
15041007	XM_003363950 // LOC100630568 // ATP synthase subunit epsilon, mitochondrial-like // ---	LOC100630568	<0.05	2.41492
14930009	---		<0.05	2.41446
14962228	ENSECAT00000026314 // LOC100059869 // glyoxalase domain-containing protein 4-like // --	LOC100059869	<0.05	2.41388
15134732	---		<0.05	2.41359
14961836	XM_001504001 // LOC100058439 // protein Njmu-R1-like // --- // 100058439 /// ENSECAT000	LOC100058439	<0.05	2.41346
15047852	XM_001494497 // LOC100063171 // serine/threonine-protein phosphatase 4 regulatory subun	LOC100063171	<0.05	2.41272
15116915	NM_001256934 // SOCS6 // suppressor of cytokine signaling 6 // --- // 100052089 /// ENS	SOCS6	<0.05	2.41177
15057511	XM_001491998 // LOC100051135 // UPF0636 protein C4orf41-like // --- // 100051135 /// EN	LOC100051135	<0.05	2.41169
14949289	XM_001490648 // UBA2 // ubiquitin-like modifier activating enzyme 2 // --- // 100057234	UBA2	<0.05	2.40919
14951148	ENSECAT00000003823 // LOC100147583 // IgG receptor FcRn large subunit p51-like // --- /	LOC100147583	<0.05	2.40893
15109487	ENSECAT00000020755 // LOC100061658 // alkylated DNA repair protein alkB homolog 8-like	LOC100061658	<0.05	2.40892
14950330	---		<0.05	2.40849
15006164	XM_001504928 // LOC100068341 // pleckstrin homology domain-containing family B member 2	LOC100068341	<0.05	2.40837
14977584	ENSECAT00000025328 // LOC100067691 // H(+)/Cl(-) exchange transporter 7-like // --- //	LOC100067691	<0.05	2.40837
15030623			<0.05	2.4083
14926851	---		<0.05	2.40779
14934895	ENSECAT00000006180 // SAMD8 // sterile alpha motif domain containing 8 // --- // 100072	SAMD8	<0.05	2.4074
15056454	ENSECAT00000007884 // IRF2 // interferon regulatory factor 2 // --- // 100050990 /// XM	IRF2	<0.05	2.40737
15093066	XM_003365100 // SH3GLB1 // SH3-domain GRB2-like endophilin B1 // --- // 100052441 /// X	SH3GLB1	<0.05	2.40727
15077776	XM_001496547 // AHR // aryl hydrocarbon receptor // --- // 100066186 /// ENSECAT0000001	AHR	<0.05	2.40705
15049158	XM_001490903 // UGCG // UDP-glucose ceramide glucosyltransferase // --- // 100057611 //	UGCG	<0.05	2.40675
15037717	ENSECAT00000011343 // LOC100147598 // uncharacterized protein C20orf24 homolog // --- /	LOC100147598	<0.05	2.40629
14998631	XM_001488228 // LOC100051936 // GMP synthase [glutamine-hydrolyzing]-like // --- // 100	LOC100051936	<0.05	2.40615
14940404	XM_003363690 // SCFD1 // sec1 family domain containing 1 // --- // 100050005 /// ENSECA	SCFD1	<0.05	2.40602
15126716			<0.05	2.40529
14989018	XM_001491302 // LOC100058270 // RNA/RNP complex-1-interacting phosphatase-like // --- /	LOC100058270	<0.05	2.40418
15111351	XM_001917013 // LOC100146180 // ras-related protein Rab-11B-like // --- // 100146180 //	LOC100146180	<0.05	2.40364
15072753	ENSECAT00000011849 // TACC3 // transforming, acidic coiled-coil containing protein 3 //	TACC3	<0.05	2.4031
15069438	ENSECAT00000026430 // LOC100067122 // telomeric repeat-binding factor 2-like // --- //	LOC100067122	<0.05	2.40291
15073290	XM_001488053 // IARS2 // isoleucyl-tRNA synthetase 2, mitochondrial // --- // 100049915	IARS2	<0.05	2.40251
15029811	XM_001916775 // RANBP9 // RAN binding protein 9 // --- // 100065036 /// ENSECAT00000004	RANBP9	<0.05	2.40213
15013000	---		<0.05	2.40146
14948903	ENSECAT00000018933 // NEMF // nuclear export mediator factor // --- // 100065895	NEMF	<0.05	2.40096
15104363	ENSECAT00000000381 // ZC3H12C // zinc finger CCCH-type containing 12C // --- // 1000700	ZC3H12C	<0.05	2.3987
15002651			<0.05	2.39835
14928711	---		<0.05	2.3965
15000852	ENSECAT00000021804 // NKTR // natural killer-tumor recognition sequence // --- // 10006	NKTR	<0.05	2.39648
15003247	XM_001489540 // RCBTB2 // regulator of chromosome condensation (RCC1) and BTB (POZ) dom	RCBTB2	<0.05	2.39616
14965996			<0.05	2.39572
14978355	ENSECAT00000026545 // ZNF3 // zinc finger protein 3 // --- // 100067274 /// XM_00191552	ZNF3	<0.05	2.39523
15004412	ENSECAT00000010216 // LATS2 // LATS, large tumor suppressor, homolog 2 (Drosophila) //	LATS2	<0.05	2.39377
14929353	---		<0.05	2.39327
15017183	ENSECAT00000025632 // LOC100055706 // adenylate kinase 2, mitochondrial-like // --- //	LOC100055706	<0.05	2.39323
15014621	ENSECAT00000011003 // LOC100070138 // run domain Beclin-1 interacting and cystein-rich	LOC100070138	<0.05	2.39227
15130232	ENSECAT00000016970 // MTM1 // myotubularin 1 // --- // 100069680 /// XM_001505085 // MT	MTM1	<0.05	2.39019
14965709	XM_001494738 // STAT5A // signal transducer and activator of transcription 5A // --- //	STAT5A	<0.05	2.39012
15058712	XM_001498677 // LOC100068154 // WASH complex subunit 7-like // --- // 100068154 /// ENS	LOC100068154	<0.05	2.38967
14956456	XM_001500351 // LOC100070681 // akirin-2-like // --- // 100070681 /// ENSECAT0000001108	LOC100070681	<0.05	2.38944
15086128	XM_001495102 // LOC100064105 // lysosomal protein NCU-G1-like // --- // 100064105 /// E	LOC100064105	<0.05	2.388
14960947	XM_001502506 // MYST2 // K(lysine) acetyltransferase 7 // --- // 100056161 /// ENSECAT0	MYST2	<0.05	2.38793
14989998	XM_001500324 // SOS1 // son of sevenless homolog 1 (Drosophila) // --- // 100053889 ///	SOS1	<0.05	2.3874
15000554	ENSECAT00000026507 // LOC100064728 // kelch-like protein 18-like // --- // 100064728 //	LOC100064728	<0.05	2.3869
15076604	XM_001914803 // PSMC2 // proteasome (prosome, macropain) 26S subunit, ATPase, 2 // ---	PSMC2	<0.05	2.38598
14946959	XM_003363514 // LOC100630319 // e3 ubiquitin ligase RNF4-like // --- // 100630319 /// E	LOC100630319	<0.05	2.38491
15122420	XM_001497901 // LOC100057539 // protein FAM91A1-like // --- // 100057539 /// ENSECAT000	LOC100057539	<0.05	2.38373
15062562	XM_001916601 // UPF2 // UPF2 regulator of nonsense transcripts homolog (yeast) // --- /	UPF2	<0.05	2.38327
14982492	ENSECAT00000024748 // LOC100061298 // glucosamine-6-phosphate isomerase 1-like // --- /	LOC100061298	<0.05	2.38223
15067570	ENSECAT00000016939 // LOC100062333 // glucosamine-6-phosphate isomerase 2-like // --- /	LOC100062333	<0.05	2.38218
15121810	ENSECAT00000004552 // LOC100055388 // pleckstrin homology domain-containing family F me	LOC100055388	<0.05	2.38215
14957380	XM_001504205 // SERINC1 // serine incorporator 1 // --- // 100067255 /// ENSECAT0000001	SERINC1	<0.05	2.38206
14929671	---		<0.05	2.38125
14934473	---		<0.05	2.3809
15047168	XM_001487916 // LOC100054092 // mediator of RNA polymerase II transcription subunit 6-l	LOC100054092	<0.05	2.38077
15055179	XM_001497986 // LOC100052430 // transmembrane protein 50B-like // --- // 100052430 ///	LOC100052430	<0.05	2.3806

14982730	ENSECAT00000020878 // SIL1 // SIL1 homolog, endoplasmic reticulum chaperone (S. cerevis	SIL1	<0.05	2.38002
15069783			<0.05	2.37977
15091003	XM_001500143 // LOC100064158 // ammonium transporter Rh type B-like // --- // 100064158	LOC100064158	<0.05	2.37975
15043365	---		<0.05	2.37974
15036202	XM_003363841 // LOC100071527 // uncharacterized protein KIAA0947-like // --- // 1000715	LOC100071527	<0.05	2.37908
14977731	XM_001915361 // LOC100147047 // jmjC domain-containing protein 8-like // --- // 1001470	LOC100147047	<0.05	2.37841
15078059	ENSECAT00000004275 // TAX1BP1 // Tax1 (human T-cell leukemia virus type I) binding prot	TAX1BP1	<0.05	2.37821
15123517	ENSECAT00000016077 // SDCBP // syndecan binding protein (syntenin) // --- // 100052836	SDCBP	<0.05	2.37808
14990415	XM_001502269 // LOC100055191 // protein phosphatase 1G-like // --- // 100055191 /// ENS	LOC100055191	<0.05	2.37776
15021423	ENSECAT00000006894 // LOC100060596 // transcription elongation factor A N-terminal and	LOC100060596	<0.05	2.37771
14982649	XM_001502340 // LOC100072416 // steroid receptor RNA activator 1-like // --- // 1000724	LOC100072416	<0.05	2.37759
14998084	ENSECAT00000016410 // CDV3 // CDV3 homolog (mouse) // --- // ---	CDV3	<0.05	2.37756
15089252	ENSECAT00000009946 // YOD1 // YOD1 OTU deubiquinating enzyme 1 homolog (S. cerevisiae)	YOD1	<0.05	2.37709
14963647	XM_001488126 // LOC100051522 // serine hydroxymethyltransferase, cytosolic-like // ---	LOC100051522	<0.05	2.37659
15112685	ENSECAT00000008111 // LOC100071307 // RING finger protein 141-like // --- // 100071307	LOC100071307	<0.05	2.37568
14974710	ENSECAT00000012149 // C7orf26 // chromosome 7 open reading frame 26 // --- // ---	C7orf26	<0.05	2.37485
15019185	XM_003364468 // LOC100629488 // uncharacterized LOC100629488 // --- // 100629488 /// EN	LOC100629488	<0.05	2.37478
15094507	---		<0.05	2.37407
14976364	ENSECAT00000021755 // GGA2 // golgi-associated, gamma adaptin ear containing, ARF bindi	GGA2	<0.05	2.37396
15040177	ENSECAT00000017782 // LOC100069714 // protein NDRG3-like // --- // 100069714	LOC100069714	<0.05	2.37373
15044990	ENSECAT00000021762 // LOC100053161 // zinc transporter ZIP9-like // --- // 100053161 //	LOC100053161	<0.05	2.37368
15086428	ENSECAT00000002639 // ADAR // adenosine deaminase, RNA-specific // --- // ---	ADAR	<0.05	2.37352
15063976	XM_001915288 // LOC100059123 // nucleotide-binding oligomerization domain-containing pr	LOC100059123	<0.05	2.37324
15045207	XM_001490028 // ZNF410 // zinc finger protein 410 // --- // 100050679 /// ENSECAT000000	ZNF410	<0.05	2.37302
14935396	XM_001916677 // LOC100060551 // egl nine homolog 1-like // --- // 100060551 /// ENSECAT	LOC100060551	<0.05	2.37289
14998166	XM_001496380 // PCCB // propionyl CoA carboxylase, beta polypeptide // --- // 100065950	PCCB	<0.05	2.37289
15025032	XM_001493351 // INTS9 // integrator complex subunit 9 // --- // 100061398 /// XM_003364	INTS9	<0.05	2.37248
14935485	ENSECAT00000023662 // LOC100051461 // poly(ADP-ribose) glycohydrolase-like // --- // 10	LOC100051461	<0.05	2.37217
15005451	ENSECAT00000013234 // LOC100053014 // probable E3 ubiquitin-protein ligase MYCBP2-like	LOC100053014	<0.05	2.3717
15017042	XM_001499327 // LOC100069588 // splicing factor, proline- and glutamine-rich-like // --	LOC100069588	<0.05	2.37129
15098084	XM_001916898 // TMEM5 // transmembrane protein 5 // --- // 100056566 /// ENSECAT0000001	TMEM5	<0.05	2.37116
15102008	ENSECAT00000009299 // BIN2 // bridging integrator 2 // --- // 100060850 /// XM_00149298	BIN2	<0.05	2.37113
15115169	ENSECAT00000005660 // LOC100059210 // zinc finger protein 664-like // --- // 100059210	LOC100059210	<0.05	2.37033
15114744	ENSECAT00000005674 // LOC100058336 // actin-related protein 2/3 complex subunit 3-like	LOC100058336	<0.05	2.3689
15121035	XM_001493139 // LOC100051585 // translocating chain-associated membrane protein 1-like	LOC100051585	<0.05	2.36869
15055715	ENSECAT00000002632 // LOC100049837 // arylamine N-acetyltransferase 1-like // --- // 10	LOC100049837	<0.05	2.36862
15040763	ENSECAT00000012363 // UBE2V1 // ubiquitin-conjugating enzyme E2 variant 1 // --- // ---	UBE2V1	<0.05	2.3685
14969518	XM_001488265 // API5 // apoptosis inhibitor 5 // --- // 100050009 /// ENSECAT0000002421	API5	<0.05	2.36674
15021494	ENSECAT00000011700 // ZYG11B // zyg-11 homolog B (C. elegans) // --- // 100061587 /// X	ZYG11B	<0.05	2.36664
15096449	XM_001499230 // FAR2 // fatty acyl CoA reductase 2 // --- // 100069466 /// ENSECAT00000	FAR2	<0.05	2.36633
15106554	XM_001491833 // SLC36A4 // solute carrier family 36 (proton/amino acid symporter), memb	SLC36A4	<0.05	2.36631
14932035	---		<0.05	2.366
15080408	ENSECAT00000010067 // LOC100059468 // synaptophysin-like protein 1-like // --- // 10005	LOC100059468	<0.05	2.36575
14964662	XM_001492450 // LOC100060086 // BTB/POZ domain-containing protein KCTD2-like // --- //	LOC100060086	<0.05	2.36486
14998265	XM_001495342 // LOC100064018 // 28S ribosomal protein S22, mitochondrial-like // --- //	LOC100064018	<0.05	2.36408
15120852	ENSECAT00000024737 // LOC100050326 // inositol monophosphatase 1-like // --- // 1000503	LOC100050326	<0.05	2.36276
14943835	XM_001490352 // CSGALNACT2 // chondroitin sulfate N-acetylgalactosaminyltransferase 2 /	CSGALNACT2	<0.05	2.36273
14983519	XM_001918313 // FBXL17 // F-box and leucine-rich repeat protein 17 // --- // 100146140	FBXL17	<0.05	2.36247
15061235	XM_001502489 // LOC100055713 // protein Tob2-like // --- // 100055713 /// ENSECAT000000	LOC100055713	<0.05	2.36237
15031178	ENSECAT00000025691 // LOC100061755 // putative Bcl-2 homologous antagonist/killer 2-lik	LOC100061755	<0.05	2.36203
15042401	XM_001498260 // LOC100068420 // ubiquitin-conjugating enzyme E2 R2-like // --- // 10006	LOC100068420	<0.05	2.36188
15018974	XM_001497360 // CEP104 // centrosomal protein 104kDa // --- // 100052540 /// ENSECAT000	CEP104	<0.05	2.36147
15043564	ENSECAT00000015124 // LOC100062221 // probable palmitoyltransferase ZDHHC21-like // ---	LOC100062221	<0.05	2.36144
15064973	XM_001497165 // LOC100067034 // vacuolar protein sorting-associated protein 4A-like //	LOC100067034	<0.05	2.36128
14960598	ENSECAT00000009851 // MED1 // mediator complex subunit 1 // --- // 100054967	MED1	<0.05	2.36122
14947295	ENSECAT00000022504 // RTF1 // Rtf1, Paf1/RNA polymerase II complex component, homolog (	RTF1	<0.05	2.36063
15136294	---		<0.05	2.36007
14925253	---		<0.05	2.36003
15073562	ENSECAT00000016219 // CDC73 // cell division cycle 73, Paf1/RNA polymerase II complex c	CDC73	<0.05	2.35935
15065320	ENSECAT00000009335 // AARS // alanyl-tRNA synthetase // --- // 100054983 /// XM_0015010	AARS	<0.05	2.35906
15044163	ENSECAT00000011672 // LOC100057755 // constitutive coactivator of PPAR-gamma-like prote	LOC100057755	<0.05	2.35892
15003288	ENSECAT00000026161 // LOC100050585 // s-formylglutathione hydrolase-like // --- // 1000	LOC100050585	<0.05	2.35847
15012621	XM_001501564 // OSBPL11 // oxysterol binding protein-like 11 // --- // 100060459 /// EN	OSBPL11	<0.05	2.35847
15025669	XM_001501751 // LOC100062612 // transmembrane protein 184C-like // --- // 100062612 ///	LOC100062612	<0.05	2.35785
15108589	---		<0.05	2.35774
15131753	ENSECAT00000025881 // LOC100052348 // a-kinase anchor protein 4-like // --- // 10005234	LOC100052348	<0.05	2.35675
15069606	XM_001498400 // DDX19B // DEAD (Asp-Glu-Ala-Asp) box polypeptide 19B // --- // 10006857	DDX19B	<0.05	2.35672
14972403	ENSECAT00000014025 // ACP2 // acid phosphatase 2, lysosomal // --- // 100057910 /// XM_	ACP2	<0.05	2.35653
14995383	XM_001498669 // TMF1 // TATA element modulatory factor 1 // --- // 100053441 /// ENSECA	TMF1	<0.05	2.35511
14965963	XM_001917560 // LOC100068316 // cyclin-dependent kinase 12-like // --- // 100068316 ///	LOC100068316	<0.05	2.35414
15031285	ENSECAT00000011657 // FKBP5 // FK506 binding protein 5 // --- // 100053546 /// XM_00149	FKBP5	<0.05	2.35322
15061360	XM_001500736 // LOC100071050 // NADH-cytochrome b5 reductase 3-like // --- // 100071050	LOC100071050	<0.05	2.3522
15052962	XM_001499306 // LOC100069565 // uridine-cytidine kinase 1-like // --- // 100069565 ///	LOC100069565	<0.05	2.35198
15057173	XM_001495441 // LOC100058282 // UBX domain-containing protein 8-like // --- // 10005828	LOC100058282	<0.05	2.35128
14974840	ENSECAT00000005115 // LOC100059822 // protein tweety homolog 3-like // --- // 100059822	LOC100059822	<0.05	2.351
15036906	ENSECAT00000015332 // GFRA4 // GDNF family receptor alpha 4 // --- // ---	GFRA4	<0.05	2.35082
14976819	ENSECAT00000019299 // LOC100050655 // poly(A)-specific ribonuclease PARN-like // --- //	LOC100050655	<0.05	2.35072
14948679	XM_001490994 // LOC100057766 // serine/threonine-protein phosphatase 2A regulatory subu	LOC100057766	<0.05	2.35071
15048672	XM_001496562 // LOC100066214 // DDB1- and CUL4-associated factor 10-like // --- // 1000	LOC100066214	<0.05	2.3506
15097256	ENSECAT00000024043 // MFSD5 // major facilitator superfamily domain containing 5 // ---	MFSD5	<0.05	2.35029
15122538	ENSECAT00000016194 // EFR3A // EFR3 homolog A (S. cerevisiae) // --- // 100057938 /// X	EFR3A	<0.05	2.34959
15041337	ENSECAT00000000099 // LOC100062308 // UPF0308 protein C9orf21-like // --- // 100062308	LOC100062308	<0.05	2.3494
15104282	ENSECAT00000023313 // ATM // ataxia telangiectasia mutated // --- // 100061765	ATM	<0.05	2.34929
14986533	XM_001918095 // LOC100073158 // AP-3 complex subunit sigma-1-like // --- // 100073158 /	LOC100073158	<0.05	2.3484
14937788	ENSECAT00000024286 // BNIP2 // BCL2/adenovirus E1B 19kDa interacting protein 2 // --- /	BNIP2	<0.05	2.34742
14967717			<0.05	2.34734
14953126	XM_001504242 // RNF146 // ring finger protein 146 // --- // 100067400 /// ENSECAT000000	RNF146	<0.05	2.34715
15132403	---		<0.05	2.34584
15005384	XM_001495217 // DIS3 // DIS3 mitotic control homolog (S. cerevisiae) // --- // 10005193	DIS3	<0.05	2.3457
15012996	ENSECAT00000002157 // CD80 // CD80 molecule // --- // ---	CD80	<0.05	2.34538
15055973	XM_001491499 // LOC100058661 // TM2 domain-containing protein 2-like // --- // 10005866	LOC100058661	<0.05	2.34429
15088736	ENSECAT00000020999 // LOC100068435 // leucine-rich repeat-containing protein 40-like //	LOC100068435	<0.05	2.34398
14938354	XM_001499778 // SECISBP2L // SECIS binding protein 2-like // --- // 100070100 /// ENSEC	SECISBP2L	<0.05	2.34349
15096465	ENSECAT00000011054 // LOC100064870 // uncharacterized protein C12orf35-like // --- // 1	LOC100064870	<0.05	2.34213
15047065	ENSECAT00000019613 // ZFYVE26 // zinc finger, FYVE domain containing 26 // --- // 10006	ZFYVE26	<0.05	2.3416
15011113	XM_001503615 // ORC2 // origin recognition complex, subunit 2 // --- // 100067880 /// E	ORC2	<0.05	2.34117
15118967	---		<0.05	2.34102
15091618	XM_003364988 // ADAMTSL4 // ADAMTS-like 4 // --- // 100059096 /// ENSECAT00000020422 //	ADAMTSL4	<0.05	2.34075
15044413	ENSECAT00000020848 // ARID4A // AT rich interactive domain 4A (RBP1-like) // --- // 100	ARID4A	<0.05	2.34073
15074458	---		<0.05	2.34029
14968547	ENSECAT00000011785 // LOC100062246 // trafficking protein particle complex subunit 1-li	LOC100062246	<0.05	2.34001
14969150	XM_001488362 // LOC100050478 // phosphoribosyl pyrophosphate synthase-associated protei	LOC100050478	<0.05	2.33981
14934479	XM_001917989 // SUPV3L1 // suppressor of var1, 3-like 1 (S. cerevisiae) // --- // 10007	SUPV3L1	<0.05	2.33852
15037048	ENSECAT00000007007 // LOC100067632 // NSFL1 cofactor p47-like // --- // 100067632 /// X	LOC100067632	<0.05	2.33851
15014573	XM_001501086 // LOC100060183 // serine/threonine-protein kinase PAK 2-like // --- // 10	LOC100060183	<0.05	2.33845
14961954	ENSECAT00000016594 // LOC100058999 // transmembrane protein 199-like // --- // 10005899	LOC100058999	<0.05	2.33817

15029056	XM_001498345 // LOC100068514 // CD2-associated protein-like // --- // 100068514 /// ENS	LOC100068514	<0.05	2.33733
15042753	XM_001496258 // NAA35 // N(alpha)-acetyltransferase 35, NatC auxiliary subunit // --- /	NAA35	<0.05	2.33672
15120156	XM_001498811 // LOC100053377 // haloacid dehalogenase-like hydrolase domain-containing	LOC100053377	<0.05	2.33642
15006497	XM_001915413 // CCNT2 // cyclin T2 // --- // 100050510 /// ENSECAT00000024357 // CCNT2	CCNT2	<0.05	2.33566
15118724	ENSECAT00000021600 // LOC100058709 // diablo homolog, mitochondrial-like // --- // 1000	LOC100058709	<0.05	2.33492
14926723	---		<0.05	2.33459
15124419	ENSECAT00000022595 // LOC100066471 // 39S ribosomal protein L13, mitochondrial-like //	LOC100066471	<0.05	2.33453
15002647	---		<0.05	2.33355
15035571	XM_001498159 // LOC100052705 // NAD(P) transhydrogenase, mitochondrial-like // --- // 1	LOC100052705	<0.05	2.33333
15125111	ENSECAT00000018149 // LOC100147640 // plectin-like // --- // 100147640 /// ENSECAT00000	LOC100147640	<0.05	2.3325
15067444	ENSECAT00000026467 // TEC // tec protein tyrosine kinase // --- // 100061381 /// XM_001	TEC	<0.05	2.33215
15101778	ENSECAT00000018950 // LOC100058932 // histone-lysine N-methyltransferase MLL2-like // -	LOC100058932	<0.05	2.33143
14983727	XM_001918332 // TTC37 // tetratricopeptide repeat domain 37 // --- // 100065001 /// ENS	TTC37	<0.05	2.33119
15026873	ENSECAT00000021397 // MYLIP // myosin regulatory light chain interacting protein // ---	MYLIP	<0.05	2.33003
14970661	XM_001489294 // LOC100054829 // ubiquitin thioesterase OTUB1-like // --- // 100054829 /	LOC100054829	<0.05	2.32977
15109397	XM_001498937 // LOC100069130 // DCN1-like protein 5-like // --- // 100069130 /// ENSECA	LOC100069130	<0.05	2.32908
15041992	XM_001493301 // LOC100051824 // melanoma antigen recognized by T-cells 1-like // --- //	LOC100051824	<0.05	2.32908
15134818	---		<0.05	2.32868
15137830	---		<0.05	2.32848
15071487	ENSECAT00000025747 // EXOC1 // exocyst complex component 1 // --- // 100059087	EXOC1	<0.05	2.32785
15089756	NM_001256902 // TRIM59 // tripartite motif containing 59 // --- // 100049895 /// ENSECA	TRIM59	<0.05	2.32768
14952011	---		<0.05	2.32763
15044315	ENSECAT00000017524 // KTN1 // kinectin 1 (kinesin receptor) // --- // 100050521 /// ENS	KTN1	<0.05	2.32527
15070391	ENSECAT00000022636 // RAP1GDS1 // RAP1, GTP-GDP dissociation stimulator 1 // --- // ---	RAP1GDS1	<0.05	2.3248
14987684	ENSECAT00000011814 // RAD17 // RAD17 homolog (S. pombe) // --- // 100065769 /// XM_0015	RAD17	<0.05	2.3247
15061351	ENSECAT00000019601 // POLDIP3 // polymerase (DNA-directed), delta interacting protein 3	POLDIP3	<0.05	2.3246
14963988	ENSECAT00000011767 // LOC100055613 // hepatocyte growth factor-regulated tyrosine kinas	LOC100055613	<0.05	2.32445
14925287	---		<0.05	2.32434
15048663	XM_001504273 // LOC100066293 // RNA (guanine-9-)-methyltransferase domain-containing pr	LOC100066293	<0.05	2.32422
15108310	ENSECAT00000020331 // LOC100072557 // uncharacterized protein C11orf46 homolog // --- /	LOC100072557	<0.05	2.32388
15057415	ENSECAT00000018058 // LOC100056687 // cytochrome P450 4V2-like // --- // 100056687 ///	LOC100056687	<0.05	2.32385
14984074	XM_001504673 // POC5 // POC5 centriolar protein homolog (Chlamydomonas) // --- // 10007	POC5	<0.05	2.32273
14929809	---		<0.05	2.32228
15130174	XM_001490008 // LOC100056788 // 60S ribosomal protein L34-like // --- // 100056788 ///	LOC100056788	<0.05	2.32175
14981370	ENSECAT00000011626 // MGAT4B // mannosyl (alpha-1,3-)-glycoprotein beta-1,4-N-acetylglu	MGAT4B	<0.05	2.32161
15008411	XM_001498513 // FASTKD2 // FAST kinase domains 2 // --- // 100068684 /// ENSECAT0000000	FASTKD2	<0.05	2.32155
14954224	ENSECAT00000012048 // TGFB1 // transforming growth factor, beta 1 // --- // 100033900 /	TGFB1	<0.05	2.32042
14929687	---		<0.05	2.32
14996160	XM_001915582 // VPRBP // Vpr (HIV-1) binding protein // --- // 100061218 /// ENSECAT000	VPRBP	<0.05	2.31984
15023835	ENSECAT00000001121 // DDI2 // DNA-damage inducible 1 homolog 2 (S. cerevisiae) // --- /	DDI2	<0.05	2.31952
15096954	ENSECAT00000023621 // ACCN2 // acid-sensing (proton-gated) ion channel 1 // --- // 1000	ACCN2	<0.05	2.31865
15042177	ENSECAT00000000697 // DENND4C // DENN/MADD domain containing 4C // --- // 100052312	DENND4C	<0.05	2.31834
15102840	---		<0.05	2.31816
14937408	XM_003363658 // DPP8 // dipeptidyl-peptidase 8 // --- // 100053245 /// XM_001497856 //	DPP8	<0.05	2.31787
15047965	ENSECAT00000013587 // BTBD7 // BTB (POZ) domain containing 7 // --- // 100053454	BTBD7	<0.05	2.31782
14928147	---		<0.05	2.31737
14932781	ENSECAT00000020131 // LOC100058723 // uncharacterized protein C10orf84-like // --- // 1	LOC100058723	<0.05	2.31726
14969350	ENSECAT00000025010 // CSTF3 // cleavage stimulation factor, 3' pre-RNA, subunit 3, 77kD	CSTF3	<0.05	2.31688
15120723	ENSECAT00000002752 // ZADH2 // zinc binding alcohol dehydrogenase domain containing 2 /	ZADH2	<0.05	2.31681
15075572	ENSECAT00000007454 // SHPRH // SNF2 histone linker PHD RING helicase, E3 ubiquitin prot	SHPRH	<0.05	2.3159
15101686	ENSECAT00000016868 // CCNT1 // cyclin T1 // --- // 100033893 /// NM_001081846 // CCNT1	CCNT1	<0.05	2.31438
14937776	NM_001123380 // ANXA2 // annexin A2 // --- // 100054320 /// ENSECAT00000013926 // ANXA2	ANXA2	<0.05	2.31338
14991243	ENSECAT00000008824 // LOC100073041 // transmembrane protein 18-like // --- // 100073041	LOC100073041	<0.05	2.31229
14998456	ENSECAT00000008736 // HPS3 // Hermansky-Pudlak syndrome 3 // --- // 100058614	HPS3	<0.05	2.31222
15009986	XM_001499353 // OLA1 // Obg-like ATPase 1 // --- // 100053204 /// ENSECAT00000017411 //	OLA1	<0.05	2.31174
15021462	XM_001493315 // LOC100061338 // non-specific lipid-transfer protein-like // --- // 1000	LOC100061338	<0.05	2.31003
14928213	---		<0.05	2.30904
15066901	XM_001488484 // LOC100050172 // mitochondrial inner membrane protein COX18-like // ---	LOC100050172	<0.05	2.30883
15110265	ENSECAT00000014843 // SIAE // sialic acid acetylesterase // --- // 100072250	SIAE	<0.05	2.30839
15061879	XM_001491887 // CUL2 // cullin 2 // --- // 100054982 /// ENSECAT00000016792 // CUL2 //	CUL2	<0.05	2.30741
14968520	ENSECAT00000008309 // TP53 // tumor protein p53 // --- // 100062044 /// NM_001202405 //	TP53	<0.05	2.30708
14933187	---		<0.05	2.30632
15033267	XM_001493894 // LOC100062257 // lipid phosphate phosphohydrolase 1-like // --- // 10006	LOC100062257	<0.05	2.30609
14956701	XM_001503857 // FBXL4 // F-box and leucine-rich repeat protein 4 // --- // 100066051 //	FBXL4	<0.05	2.30597
15079262	XM_003364867 // LOC100629380 // single-stranded DNA-binding protein, mitochondrial-like	LOC100629380	<0.05	2.30593
14928627	---		<0.05	2.30526
15082918	XM_001500519 // LOC100065055 // modulator of retrovirus infection homolog // --- // 100	LOC100065055	<0.05	2.30518
15058161	ENSECAT00000029120 // LOC100629608 // 39S ribosomal protein L42, mitochondrial-like //	LOC100629608	<0.05	2.30486
15099566	ENSECAT00000010760 // BCL2L13 // BCL2-like 13 (apoptosis facilitator) // --- // 1000550	BCL2L13	<0.05	2.3046
14983384	XM_003362836 // LOC100073160 // ubiquitin-like protein ATG12-like // --- // 100073160 /	LOC100073160	<0.05	2.30441
15102842	XM_001492303 // LOC100059858 // prostaglandin E synthase 3-like // --- // 100059858 ///	LOC100059858	<0.05	2.30415
15079112	XM_001496985 // TRIM24 // tripartite motif containing 24 // --- // 100066810 /// ENSECA	TRIM24	<0.05	2.30382
15119950	ENSECAT00000021963 // LOC100067081 // zinc finger protein 396-like // --- // 100067081	LOC100067081	<0.05	2.30279
15011717	XM_001495170 // LOC100058230 // mitofusin-1-like // --- // 100058230 /// ENSECAT0000002	LOC100058230	<0.05	2.30275
14962032	---		<0.05	2.302
15057755	ENSECAT00000024916 // FBXO25 // F-box protein 25 // --- // 100051951 /// XM_001494835 /	FBXO25	<0.05	2.30143
14992775	ENSECAT00000011823 // LOC100061904 // RNA-binding protein PNO1-like // --- // 100061904	LOC100061904	<0.05	2.30093
15022824			<0.05	2.30082
15048250	XM_001491699 // CDC42BPB // CDC42 binding protein kinase beta (DMPK-like) // --- // 100	CDC42BPB	<0.05	2.3008
15008947	XM_001490023 // DARS // aspartyl-tRNA synthetase // --- // 100050898 /// ENSECAT0000002	DARS	<0.05	2.30061
14999605	XM_001489265 // ABHD6 // abhydrolase domain containing 6 // --- // 100057821 /// ENSECA	ABHD6	<0.05	2.30028
15120841	XM_001915075 // LOC100057220 // AN1-type zinc finger protein 1-like // --- // 100057220	LOC100057220	<0.05	2.29938
15058116	XM_001915050 // TMTC3 // transmembrane and tetratricopeptide repeat containing 3 // ---	TMTC3	<0.05	2.2991
15107893	ENSECAT00000021058 // LOC100071555 // fatty acyl-CoA reductase 1-like // --- // 1000715	LOC100071555	<0.05	2.29883
15021209	XM_003364530 // TBCK // TBC1 domain containing kinase // --- // 100064433 /// ENSECAT00	TBCK	<0.05	2.29826
14938147	XM_001501499 // LEO1 // Leo1, Paf1/RNA polymerase II complex component, homolog (S. cer	LEO1	<0.05	2.29807
14966049	ENSECAT00000000672 // LOC100055317 // 39S ribosomal protein L45, mitochondrial-like //	LOC100055317	<0.05	2.29781
14986608	XM_001504579 // APC // adenomatous polyposis coli // --- // 100064431 /// XM_003362839	APC	<0.05	2.29764
15024052	ENSECAT00000017643 // MFN2 // mitofusin 2 // --- // ---	MFN2	<0.05	2.29717
14966200	XM_001502391 // LOC100056039 // prohibitin-like // --- // 100056039 /// ENSECAT00000018	LOC100056039	<0.05	2.29668
15090155	XM_001915627 // LOC100054392 // neudesin-like // --- // 100054392 /// ENSECAT0000001558	LOC100054392	<0.05	2.29662
15109557	---		<0.05	2.29624
14932023	---		<0.05	2.29603
15024477	XM_001492683 // LOC100060427 // DNA fragmentation factor subunit beta-like // --- // 10	LOC100060427	<0.05	2.29576
15085516	ENSECAT00000014497 // LOC100066229 // ubiquitin-fold modifier-conjugating enzyme 1-like	LOC100066229	<0.05	2.29548
14991723	XM_001493723 // LOC100061973 // cytochrome c oxidase subunit 5B, mitochondrial-like //	LOC100061973	<0.05	2.29524
14935153	ENSECAT00000009430 // ERO1LB // ERO1-like beta (S. cerevisiae) // --- // 100050567 ///	ERO1LB	<0.05	2.29466
14976946	---		<0.05	2.29456
15131559	XM_001492656 // CFP // complement factor properdin // --- // 100051317 /// ENSECAT00000	CFP	<0.05	2.29378
15003956	ENSECAT00000005088 // TPP2 // tripeptidyl peptidase II // --- // 100050973	TPP2	<0.05	2.29354
14939581	XM_001502551 // PARP2 // poly (ADP-ribose) polymerase 2 // --- // 100072572 /// ENSECAT	PARP2	<0.05	2.2926
15011383	ENSECAT00000012896 // IDH1 // isocitrate dehydrogenase 1 (NADP+), soluble // --- // 100	IDH1	<0.05	2.29152
15131668	XM_001495045 // WDR45 // WD repeat domain 45 // --- // 100052044 /// ENSECAT00000007316	WDR45	<0.05	2.29109
14929859	---		<0.05	2.2904
15133485	ENSECAT00000020006 // FAM122B // family with sequence similarity 122B // --- // ---	FAM122B	<0.05	2.29022
15091547	XM_001491678 // LOC100055347 // tumor necrosis factor alpha-induced protein 8-like prot	LOC100055347	<0.05	2.28911

14931747	---		<0.05	2.28866
14928839	---		<0.05	2.28831
14989961	XM_001499556 // MAP4K3 // mitogen-activated protein kinase kinase kinase kinase 3 // --	MAP4K3	<0.05	2.28822
14932031	---		<0.05	2.28776
15066535	XM_001494273 // LIN54 // lin-54 homolog (C. elegans) // --- // 100052135 /// ENSECAT000	LIN54	<0.05	2.28769
15070651	ENSECAT00000020843 // NUDT9 // nudix (nucleoside diphosphate linked moiety X)-type moti	NUDT9	<0.05	2.28728
15076761	XM_001491576 // DLD // dihydrolipoamide dehydrogenase // --- // 100050920 /// ENSECAT00	DLD	<0.05	2.2869
14968167	XM_001504736 // LOC100060837 // thioredoxin domain-containing protein 17-like // --- //	LOC100060837	<0.05	2.28662
15024088	ENSECAT00000016665 // CLCN6 // chloride channel, voltage-sensitive 6 // --- // 10005620	CLCN6	<0.05	2.28617
15050655	ENSECAT00000021079 // LOC100069096 // retinoic acid receptor RXR-alpha-like // --- // 1	LOC100069096	<0.05	2.28581
14979634	ENSECAT00000013555 // UBFD1 // ubiquitin family domain containing 1 // --- // ---	UBFD1	<0.05	2.28466
15012255	---		<0.05	2.28405
14983202			<0.05	2.28403
14947647	XM_001503666 // LOC100057906 // UPF0480 protein C15orf24-like // --- // 100057906 /// E	LOC100057906	<0.05	2.28399
14959469	XM_003362530 // CDC27 // cell division cycle 27 homolog (S. cerevisiae) // --- // 10005	CDC27	<0.05	2.28396
15028930			<0.05	2.28187
15114716	---		<0.05	2.28172
15114717			<0.05	2.28172
15109469	XM_001500896 // CWF19L2 // CWF19-like 2, cell cycle control (S. pombe) // --- // 100069	CWF19L2	<0.05	2.28155
14941182	XM_001490046 // BUB3 // budding uninhibited by benzimidazoles 3 homolog (yeast) // ---	BUB3	<0.05	2.2814
14966386	ENSECAT00000016323 // LOC100056543 // cytochrome c oxidase assembly protein COX11, mito	LOC100056543	<0.05	2.28135
15109189	ENSECAT00000014032 // LOC100061007 // protein CWC15 homolog // --- // 100061007 /// XM_	LOC100061007	<0.05	2.28103
15054820	NM_001256955 // NRIP1 // nuclear receptor interacting protein 1 // --- // 100054242 ///	NRIP1	<0.05	2.28071
15018702	XM_001493871 // LOC100051553 // protein LZIC-like // --- // 100051553 /// ENSECAT000000	LOC100051553	<0.05	2.2807
14932113	---		<0.05	2.28039
15030129	ENSECAT00000026948 // LOC100053593 // histone H1.4-like // --- // 100053593 /// ENSECAT	LOC100053593	<0.05	2.2796
15015391	ENSECAT00000026105 // LOC100072293 // TBC1 domain family member 23-like // --- // 10007	LOC100072293	<0.05	2.27873
15001886	XM_001499235 // DBR1 // debranching enzyme homolog 1 (S. cerevisiae) // --- // 10005354	DBR1	<0.05	2.27843
15108665	ENSECAT00000010103 // MAP2K2 // mitogen-activated protein kinase kinase 2 // --- // ---	MAP2K2	<0.05	2.27842
15039454	ENSECAT00000025772 // TRMT6 // tRNA methyltransferase 6 homolog (S. cerevisiae) // ---	TRMT6	<0.05	2.27795
15010053	XM_003363286 // ATF2 // activating transcription factor 2 // --- // 100053304 /// XM_00	ATF2	<0.05	2.27774
15044031	XM_001503698 // LOC100068546 // dynactin subunit 3-like // --- // 100068546 /// ENSECAT	LOC100068546	<0.05	2.27772
14951260	XM_001490016 // LOC100056147 // mediator of RNA polymerase II transcription subunit 25-	LOC100056147	<0.05	2.27661
15119564	ENSECAT00000010001 // THOC1 // THO complex 1 // --- // 100051155 /// XM_001491890 // TH	THOC1	<0.05	2.2761
14938609	XM_001502921 // MFAP1 // microfibrillar-associated protein 1 // --- // 100070739 /// EN	MFAP1	<0.05	2.27516
14966715	XM_001917870 // PPM1D // protein phosphatase, Mg2+/Mn2+ dependent, 1D // --- // 1000713	PPM1D	<0.05	2.27498
14928177	---		<0.05	2.27411
15048584	ENSECAT00000013054 // LOC100067265 // uncharacterized LOC100067265 // --- // 100067265	LOC100067265	<0.05	2.27407
15010067	XM_001499935 // LOC100053347 // ATP synthase lipid-binding protein, mitochondrial-like	LOC100053347	<0.05	2.27116
15052297	ENSECAT00000000118 // MEGF9 // multiple EGF-like-domains 9 // --- // ---	MEGF9	<0.05	2.27116
15009320	ENSECAT00000018424 // LOC100059499 // cytohesin-interacting protein-like // --- // 1000	LOC100059499	<0.05	2.27095
14944698	XM_001498201 // WHAMM // WAS protein homolog associated with actin, golgi membranes and	WHAMM	<0.05	2.27067
15007810	ENSECAT00000026450 // ASNSD1 // asparagine synthetase domain containing 1 // --- // 100	ASNSD1	<0.05	2.27041
14988259			<0.05	2.27021
14970631	---		<0.05	2.26994
14926083	---		<0.05	2.26993
15063044	---		<0.05	2.26967
14936835	XM_001490810 // HMG20A // high mobility group 20A // --- // 100061369 /// ENSECAT000000	HMG20A	<0.05	2.26957
15031223	ENSECAT00000005973 // LOC100062360 // 40S ribosomal protein S10-like // --- // 10006236	LOC100062360	<0.05	2.26867
15053837	ENSECAT00000009319 // SOD1 // superoxide dismutase 1, soluble // --- // 100033855 /// N	SOD1	<0.05	2.2684
15053028	ENSECAT00000028848 // TTF1 // transcription termination factor, RNA polymerase I // ---	TTF1	<0.05	2.26825
15133413	ENSECAT00000022620 // MBNL3 // muscleblind-like splicing regulator 3 // --- // 10005431	MBNL3	<0.05	2.26792
14925279	---		<0.05	2.26789
15060858			<0.05	2.26783
14944092	XM_001492754 // LOC100050879 // tubulin-specific chaperone E-like // --- // 100050879 /	LOC100050879	<0.05	2.26568
15088033	XM_001491203 // LOC100058111 // UDP-N-acetylglucosamine transferase subunit ALG14 homol	LOC100058111	<0.05	2.26563
14941242	XM_001490808 // PLEKHA1 // pleckstrin homology domain containing, family A (phosphoinos	PLEKHA1	<0.05	2.2653
14946129	XM_001496225 // FEM1B // fem-1 homolog b (C. elegans) // --- // 100052688 /// ENSECAT00	FEM1B	<0.05	2.26521
14998956	XM_001492367 // TSEN2 // tRNA splicing endonuclease 2 homolog (S. cerevisiae) // --- //	TSEN2	<0.05	2.26511
15038345	ENSECAT00000025931 // ARFGEF2 // ADP-ribosylation factor guanine nucleotide-exchange fa	ARFGEF2	<0.05	2.26507
15029640	XM_001489627 // LOC100050671 // LYR motif-containing protein 4-like // --- // 100050671	LOC100050671	<0.05	2.26452
14983835	XM_001504620 // LOC100065122 // centrin-3-like // --- // 100065122 /// ENSECAT000000266	LOC100065122	<0.05	2.26406
14963755	ENSECAT00000022283 // COPS3 // COP9 constitutive photomorphogenic homolog subunit 3 (Ar	COPS3	<0.05	2.26316
15114320	XM_001490500 // TAOK3 // TAO kinase 3 // --- // 100050932 /// ENSECAT00000007673 // TAO	TAOK3	<0.05	2.26295
15077284	XM_001489324 // DBF4 // DBF4 homolog (S. cerevisiae) // --- // 100050313 /// ENSECAT000	DBF4	<0.05	2.26291
15063631	XM_003364333 // TAF3 // TAF3 RNA polymerase II, TATA box binding protein (TBP)-associat	TAF3	<0.05	2.26225
14987274	ENSECAT00000023530 // LOC100073271 // secretory carrier-associated membrane protein 1-l	LOC100073271	<0.05	2.26221
15111557	ENSECAT00000007695 // TMEM135 // transmembrane protein 135 // --- // 100060782	TMEM135	<0.05	2.26216
14971251	XM_001496281 // DPP3 // dipeptidyl-peptidase 3 // --- // 100058485 /// ENSECAT000000171	DPP3	<0.05	2.26163
15130836	ENSECAT00000014292 // LOC100050951 // pirin-like // --- // 100050951 /// XM_001490018 /	LOC100050951	<0.05	2.26143
15103333	ENSECAT00000000406 // LOC100068855 // NADH dehydrogenase [ubiquinone] iron-sulfur prote	LOC100068855	<0.05	2.26012
15047903	ENSECAT00000007937 // LOC100063370 // thyroid receptor-interacting protein 11-like // -	LOC100063370	<0.05	2.25998
14951143			<0.05	2.25876
15121402	XM_001914675 // LOC100054947 // v-type proton ATPase subunit H-like // --- // 100054947	LOC100054947	<0.05	2.25866
15132894	XM_001489664 // LOC100054363 // AMME syndrome candidate gene 1 protein homolog // --- /	LOC100054363	<0.05	2.2579
14957237	ENSECAT00000014086 // ZUFSP // zinc finger with UFM1-specific peptidase domain // --- /	ZUFSP	<0.05	2.25773
15031426	ENSECAT00000018372 // BTBD9 // BTB (POZ) domain containing 9 // --- // 100065100 /// EN	BTBD9	<0.05	2.25672
15049099	XM_001915992 // RAD23B // RAD23 homolog B (S. cerevisiae) // --- // 100059968 /// ENSEC	RAD23B	<0.05	2.25651
15017051	XM_003364411 // ZMYM6 // zinc finger, MYM-type 6 // --- // 100629888 /// XM_003364412 /	ZMYM6	<0.05	2.25613
15086375	ENSECAT00000007364 // LOC100063346 // dolichol-phosphate mannosyltransferase subunit 3-	LOC100063346	<0.05	2.25531
14992966	XM_001917544 // LOC100052114 // poly(A) polymerase gamma-like // --- // 100052114 /// E	LOC100052114	<0.05	2.2546
15038589	ENSECAT00000013401 // LOC100056212 // ras-related protein Rab-22A-like // --- // 100056	LOC100056212	<0.05	2.25456
15103945	ENSECAT00000020044 // ANGPTL4 // angiopoietin-like 4 // --- // ---	ANGPTL4	<0.05	2.25446
14976845	ENSECAT00000019796 // CPPED1 // calcineurin-like phosphoesterase domain containing 1 //	CPPED1	<0.05	2.25422
14931815	---		<0.05	2.25324
14929921	---		<0.05	2.25311
15123896	XM_001490437 // MTERFD1 // MTERF domain containing 1 // --- // 100055471 /// ENSECAT000	MTERFD1	<0.05	2.25305
15117714	ENSECAT00000020990 // MTMR3 // myotubularin related protein 3 // --- // 100058825 /// X	MTMR3	<0.05	2.25247
14964697	ENSECAT00000006437 // TMEM104 // transmembrane protein 104 // --- // ---	TMEM104	<0.05	2.25245
15023007	ENSECAT00000012197 // LOC100056596 // ATPase inhibitor, mitochondrial-like // --- // 10	LOC100056596	<0.05	2.2524
15073923	ENSECAT00000008973 // TMEM183A // transmembrane protein 183A // --- // 100065104 /// XM	TMEM183A	<0.05	2.25232
15008760	ENSECAT00000012884 // MARCO // macrophage receptor with collagenous structure // --- //	MARCO	<0.05	2.24995
15069784			<0.05	2.24954
14985641	XM_001502291 // LOC100072378 // zinc finger matrin-type protein 2-like // --- // 100072	LOC100072378	<0.05	2.24915
14950588	XM_001916976 // CLPTM1 // cleft lip and palate associated transmembrane protein 1 // --	CLPTM1	<0.05	2.24731
15077970	ENSECAT00000016879 // LOC100067896 // uncharacterized protein C7orf30-like // --- // 10	LOC100067896	<0.05	2.24653
14982567	ENSECAT00000023229 // DIAPH1 // diaphanous homolog 1 (Drosophila) // --- // 100072186 /	DIAPH1	<0.05	2.24635
14963499	XM_001918389 // NCOR1 // nuclear receptor corepressor 1 // --- // 100063285 /// ENSECAT	NCOR1	<0.05	2.24607
14928185	---		<0.05	2.2446
14967386	XM_001504154 // LOC100059307 // stromal cell-derived factor 2-like // --- // 100059307	LOC100059307	<0.05	2.24398
15030929	XM_001493561 // LOC100051820 // G-protein-signaling modulator 3-like // --- // 10005182	LOC100051820	<0.05	2.24353
15008289	ENSECAT00000011539 // LOC100067617 // cytochrome P450 20A1-like // --- // 100067617	LOC100067617	<0.05	2.24237
15076691	ENSECAT00000012415 // LOC100060030 // cAMP-dependent protein kinase type II-beta regula	LOC100060030	<0.05	2.24115
15041749	XM_001916945 // LOC100050300 // Krueppel-like factor 9-like // --- // 100050300 /// ENS	LOC100050300	<0.05	2.24069
15132781	ENSECAT00000023296 // RBM41 // RNA binding motif protein 41 // --- // 100060588 /// XM_	RBM41	<0.05	2.24015
15022195	ENSECAT00000019067 // LOC100067429 // phosphopantothenate--cysteine ligase-like // ---	LOC100067429	<0.05	2.23987

14948945 XM_001496609 // SOS2 // son of sevenless homolog 2 (Drosophila) // --- // 100066282 ///	SOS2	<0.05	2.23967
15110911 ---		<0.05	2.23807
15054300 ENSECAT00000016528 // LOC100057570 // splicing factor U2AF 35 kDa subunit-like // --- /	LOC100057570	<0.05	2.23752
14993989 ENSECAT00000008586 // NRBP1 // nuclear receptor binding protein 1 // --- // 100055145 /	NRBP1	<0.05	2.23752
15068851 XM_001915110 // LOC100051355 // cleavage and polyadenylation specificity factor subunit	LOC100051355	<0.05	2.23713
14957677 ENSECAT00000006393 // LOC100073159 // HBS1-like protein-like // --- // 100073159	LOC100073159	<0.05	2.23697
15068739 XM_001490172 // LOC100050450 // AKT-interacting protein-like // --- // 100050450 /// EN	LOC100050450	<0.05	2.23609
15127834 XM_001917074 // USP11 // ubiquitin specific peptidase 11 // --- // 100051171 /// ENSECA	USP11	<0.05	2.23606
15062694		<0.05	2.23571
15130636 XM_001498198 // LOC100062131 // vesicle-associated membrane protein 7-like // --- // 10	LOC100062131	<0.05	2.23569
14994663 XM_001502898 // LOC100072846 // tetratricopeptide repeat protein 15-like // --- // 1000	LOC100072846	<0.05	2.23439
15126702		<0.05	2.23395
14989752 XM_001498271 // FBXO11 // F-box protein 11 // --- // 100053097 /// ENSECAT00000010973 /	FBXO11	<0.05	2.23393
14932097 ---		<0.05	2.23291
14964878 ENSECAT00000018682 // LOC100062972 // guanine nucleotide-binding protein subunit alpha-	LOC100062972	<0.05	2.23289
14978946 ENSECAT00000011475 // LOC100061681 // DNA-directed RNA polymerase III subunit RPC9-like	LOC100061681	<0.05	2.23287
14931903 ---		<0.05	2.23246
15042406 XM_001499590 // UBAP1 // ubiquitin associated protein 1 // --- // 100053997 /// ENSECAT	UBAP1	<0.05	2.23239
14990184 XM_001917846 // STRN // striatin, calmodulin binding protein // --- // 100054418 /// EN	STRN	<0.05	2.23228
14984469 XM_001502429 // NSD1 // nuclear receptor binding SET domain protein 1 // --- // 1000585	NSD1	<0.05	2.23221
15130042 ENSECAT00000003600 // ZNF449 // zinc finger protein 449 // --- // 100057349 /// XM_0014	ZNF449	<0.05	2.23209
15001323 XM_001489975 // PDCD6IP // programmed cell death 6 interacting protein // --- // 100050	PDCD6IP	<0.05	2.23198
14940106 XM_003363553 // LOC100055644 // polyadenylate-binding protein 2-like // --- // 10005564	LOC100055644	<0.05	2.23189
15024368 XM_003364465 // LOC100630321 // uncharacterized LOC100630321 // --- // 100630321 /// EN	LOC100630321	<0.05	2.23106
15052495 XM_001502306 // ZBTB6 // zinc finger and BTB domain containing 6 // --- // 100067456 //	ZBTB6	<0.05	2.23053
15117485 ---		<0.05	2.23027
15051784 ENSECAT00000008409 // LOC100059122 // transmembrane protein C9orf5-like // --- // 10005	LOC100059122	<0.05	2.22945
15129773 ENSECAT00000023019 // STAG2 // stromal antigen 2 // --- // 100055046 /// XM_001915150 /	STAG2	<0.05	2.22922
15023118 ENSECAT00000019681 // ARID1A // AT rich interactive domain 1A (SWI-like) // --- // ---	ARID1A	<0.05	2.22766
15000960 XM_001501861 // LOC100055285 // eukaryotic translation initiation factor 1b-like // ---	LOC100055285	<0.05	2.22761
15128147 NM_001256912 // UBQLN2 // ubiquilin 2 // --- // 100050402	UBQLN2	<0.05	2.2275
15080090 ENSECAT00000008489 // LOC100067217 // ubiquitin-protein ligase E3C-like // --- // 10006	LOC100067217	<0.05	2.22679
15057200 XM_001495800 // LOC100062721 // leptin receptor overlapping transcript-like 1-like // -	LOC100062721	<0.05	2.22641
15047991 XM_001497967 // DDX24 // DEAD (Asp-Glu-Ala-Asp) box polypeptide 24 // --- // 100053504	DDX24	<0.05	2.22637
15028528 XM_001500888 // NFYA // nuclear transcription factor Y, alpha // --- // 100054237 /// E	NFYA	<0.05	2.22636
15041965 XM_001492144 // LOC100059612 // protein RIC1 homolog // --- // 100059612 /// ENSECAT000	LOC100059612	<0.05	2.22626
15134976 ---		<0.05	2.22563
15097516 XM_001504775 // LOC100051308 // biogenesis of lysosome-related organelles complex 1 sub	LOC100051308	<0.05	2.22539
15077383 NM_001257104 // CLDN12 // claudin 12 // --- // 100060361 /// ENSECAT00000005680 // CLDN	CLDN12	<0.05	2.22469
15038627 ENSECAT00000026245 // LOC100056464 // guanine nucleotide-binding protein G(s) subunit a	LOC100056464	<0.05	2.22468
14952092 ENSECAT00000011246 // TRIM28 // tripartite motif containing 28 // --- // ---	TRIM28	<0.05	2.22411
15038603 ENSECAT00000019946 // LOC100050597 // syntaxin-16-like // --- // 100050597 /// XM_00149	LOC100050597	<0.05	2.22402
15060265 ENSECAT00000020822 // LOC100064471 // ubiquitin-conjugating enzyme E2 N-like // --- //	LOC100064471	<0.05	2.22364
15096269 XM_001502130 // LOC100064206 // pyridine nucleotide-disulfide oxidoreductase domain-con	LOC100064206	<0.05	2.22272
15052920 XM_003364107 // LOC100629960 // torsin-1A-like // --- // 100629960 /// ENSECAT000000147	LOC100629960	<0.05	2.22265
14929753 ---		<0.05	2.22239
15107397 XM_003365380 // LOC100054490 // tripartite motif-containing protein 5-like // --- // 10	LOC100054490	<0.05	2.22223
15128547 XM_001915329 // MED12 // mediator complex subunit 12 // --- // 100056909 /// ENSECAT000	MED12	<0.05	2.22198
15032182 ENSECAT00000019218 // DST // dystonin // --- // 100056885 /// ENSECAT00000019286 // DST	DST	<0.05	2.22155
14966316 XM_001499740 // LOC100070058 // ankyrin repeat domain-containing protein 40-like // ---	LOC100070058	<0.05	2.22149
15076823 ENSECAT00000018342 // CDK13 // cyclin-dependent kinase 13 // --- // ---	CDK13	<0.05	2.22135
14967281 ENSECAT00000006927 // SLC46A1 // solute carrier family 46 (folate transporter), member	SLC46A1	<0.05	2.21975
14944174 XM_001492833 // LOC100060623 // zinc finger RAD18 domain-containing protein C1orf124 ho	LOC100060623	<0.05	2.21908
15123552 ENSECAT00000020705 // TGS1 // trimethylguanosine synthase 1 // --- // 100067973 /// XM_	TGS1	<0.05	2.21792
15025423 ENSECAT00000017141 // TMEM144 // transmembrane protein 144 // --- // 100061910 /// XM_0	TMEM144	<0.05	2.21782
15096308 ENSECAT00000007430 // ETNK1 // ethanolamine kinase 1 // --- // 100068424 /// XM_0019162	ETNK1	<0.05	2.21747
14986452 XM_003362834 // DMXL1 // Dmx-like 1 // --- // 100064119 /// XM_001504549 // DMXL1 // Dm	DMXL1	<0.05	2.21723
15089051 ENSECAT00000022453 // RBBP5 // retinoblastoma binding protein 5 // --- // 100054708 ///	RBBP5	<0.05	2.21651
15119431 ENSECAT00000027140 // LOC100059211 // uncharacterized protein C18orf8-like // --- // 10	LOC100059211	<0.05	2.21623
15028124 ENSECAT00000021585 // ANKS1A // ankyrin repeat and sterile alpha motif domain containin	ANKS1A	<0.05	2.2159
15008523 XM_001504931 // UGGT1 // UDP-glucose glycoprotein glucosyltransferase 1 // --- // 10006	UGGT1	<0.05	2.21547
15077485 ENSECAT00000011565 // LOC100061456 // UPF0712 protein C7orf64-like // --- // 100061456	LOC100061456	<0.05	2.21435
15088803 ENSECAT00000015423 // LOC100069110 // uncharacterized LOC100069110 // --- // 100069110	LOC100069110	<0.05	2.21431
14993835 ENSECAT00000016734 // LOC100071067 // serine/threonine-protein phosphatase PP1-beta cat	LOC100071067	<0.05	2.21334
14962611 ENSECAT00000013793 // NUP88 // nucleoporin 88kDa // --- // 100061117 /// XM_001504741 /	NUP88	<0.05	2.21273
15132804 ENSECAT00000022230 // LOC100054590 // 26S proteasome non-ATPase regulatory subunit 10-l	LOC100054590	<0.05	2.21194
15022798 XM_001500028 // LOC100070377 // importin subunit alpha-7-like // --- // 100070377 /// E	LOC100070377	<0.05	2.21185
14956184 XM_001499042 // PHIP // pleckstrin homology domain interacting protein // --- // 100069	PHIP	<0.05	2.21171
15032956 XM_001503477 // MAU2 // MAU2 chromatid cohesion factor homolog (C. elegans) // --- // 1	MAU2	<0.05	2.2116
14967348 ENSECAT00000019247 // KIAA0100 // KIAA0100 // --- // --- /// ENSECAT00000019280 // KIAA	KIAA0100	<0.05	2.21115
15002649		<0.05	2.21066
15052959 XM_001499008 // LOC100066755 // protein FAM78A-like // --- // 100066755 /// ENSECAT0000	LOC100066755	<0.05	2.21025
15093028 ENSECAT00000026949 // PKN2 // protein kinase N2 // --- // 100052266 /// XM_001495405 //	PKN2	<0.05	2.20917
14972905 XM_001498329 // OSBP // oxysterol binding protein // --- // 100058845 /// ENSECAT000000	OSBP	<0.05	2.20874
14974659 XM_001493987 // LOC100062410 // vacuolar fusion protein CCZ1 homolog // --- // 10006241	LOC100062410	<0.05	2.20827
14935846 ENSECAT00000018146 // BTBD1 // BTB (POZ) domain containing 1 // --- // 100068143 /// XM	BTBD1	<0.05	2.20796
14981864 ENSECAT00000014600 // LOC100059674 // pre-mRNA-splicing factor SLU7-like // --- // 1000	LOC100059674	<0.05	2.20779
14943282 XM_001503722 // LOC100063421 // GTP-binding protein SAR1a-like // --- // 100063421 ///	LOC100063421	<0.05	2.20737
14933274 XM_001499233 // LOC100060168 // LIM domain-binding protein 1-like // --- // 100060168 /	LOC100060168	<0.05	2.20554
15006753 XM_001490973 // RIF1 // RAP1 interacting factor homolog (yeast) // --- // 100057721 ///	RIF1	<0.05	2.20537
14996429 XM_001497632 // LOC100053200 // nicolin-1-like // --- // 100053200 /// ENSECAT000000116	LOC100053200	<0.05	2.20514
15026771 XM_003363705 // LOC100630350 // transmembrane protein 14C-like // --- // 100630350 ///	LOC100630350	<0.05	2.20461
15073203 ENSECAT00000015379 // NVL // nuclear VCP-like // --- // 100055652 /// XM_001489738 // N	NVL	<0.05	2.20454
14993811 ENSECAT00000025709 // LOC100071019 // WD repeat-containing protein 43-like // --- // 10	LOC100071019	<0.05	2.20433
14975909 XM_001501545 // LOC100065719 // CD2 antigen cytoplasmic tail-binding protein 2-like //	LOC100065719	<0.05	2.20407
15123477 XM_001915768 // CHD7 // chromodomain helicase DNA binding protein 7 // --- // 100052622	CHD7	<0.05	2.20392
14985769 ENSECAT00000028887 // LOC100630045 // ubiquitin-conjugating enzyme E2 D2-like // --- //	LOC100630045	<0.05	2.20361
15032975 XM_001500843 // GATAD2A // GATA zinc finger domain containing 2A // --- // 100071147 //	GATAD2A	<0.05	2.2035
14940842 ENSECAT00000018811 // ZNF717 // zinc finger protein 717 // --- // --- /// ENSECAT000000	ZNF717	<0.05	2.20315
15116739 ENSECAT00000008044 // LOC100050782 // lisH domain and HEAT repeat-containing protein KI	LOC100050782	<0.05	2.20302
14963562 ENSECAT00000009563 // ULK2 // unc-51-like kinase 2 (C. elegans) // --- // 100073154 ///	ULK2	<0.05	2.20261
15070754 XM_001915383 // CDS1 // CDP-diacylglycerol synthase (phosphatidate cytidylyltransferase	CDS1	<0.05	2.20255
15117972 XM_001496710 // ANKRD13A // ankyrin repeat domain 13A // --- // 100066442 /// ENSECAT00	ANKRD13A	<0.05	2.20233
15007881 ENSECAT00000011641 // GLS // glutaminase // --- // 100069617 /// XM_001499354 // GLS //	GLS	<0.05	2.20229
15095704 XM_001915524 // USP5 // ubiquitin specific peptidase 5 (isopeptidase T) // --- // 10006	USP5	<0.05	2.20142
14971165 ENSECAT00000026515 // SF3B2 // splicing factor 3b, subunit 2, 145kDa // --- // 10005217	SF3B2	<0.05	2.20131
15130532 XM_003365909 // LOC100630700 // protein FAM50A-like // --- // 100630700 /// ENSECAT0000	LOC100630700	<0.05	2.20056
15122776 XM_001495843 // LOC100065171 // repressor of RNA polymerase III transcription MAF1 homo	LOC100065171	<0.05	2.19969
14943582 XM_001503944 // AP3M1 // adaptor-related protein complex 3, mu 1 subunit // --- // 1000	AP3M1	<0.05	2.1996
15059155 ENSECAT00000006610 // LOC100055376 // smith-Magenis syndrome chromosomal region candida	LOC100055376	<0.05	2.19876
15023177 NM_001242455 // SH3BGRL3 // SH3 domain binding glutamic acid-rich protein like 3 // ---	SH3BGRL3	<0.05	2.19873
14940734 XM_001496247 // LOC100065740 // alpha-1,6-mannosyl-glycoprotein 2-beta-N-acetylglucosam	LOC100065740	<0.05	2.19808
15037636 ENSECAT00000026813 // PHF20 // PHD finger protein 20 // --- // 100055019 /// XM_0015018	PHF20	<0.05	2.1974
15046289 ENSECAT00000014118 // WDR20 // WD repeat domain 20 // --- // 100055061 /// XM_001917644	WDR20	<0.05	2.19729
15080164 XM_001490684 // RSBN1L // round spermatid basic protein 1-like // --- // 100057295 ///	RSBN1L	<0.05	2.19718
15068603 ENSECAT00000014564 // ITFG1 // integrin alpha FG-GAP repeat containing 1 // --- // 1000	ITFG1	<0.05	2.19714

15093703	XM_001500914 // ATG4C // autophagy related 4C, cysteine peptidase // --- // 100054106 /	ATG4C	<0.05	2.19709
14928803	---		<0.05	2.19697
14936980	XM_001494059 // LOC100062522 // ubiquitin-like protein 7-like // --- // 100062522 /// X	LOC100062522	<0.05	2.19628
15046670	XM_001489495 // DDHD1 // DDHD domain containing 1 // --- // 100055200 /// ENSECAT000000	DDHD1	<0.05	2.19613
15000314	XM_001496577 // RBM5 // RNA binding motif protein 5 // --- // 100052535 /// ENSECAT0000	RBM5	<0.05	2.19605
14970888	XM_001492016 // LOC100056723 // 39S ribosomal protein L49, mitochondrial-like // --- //	LOC100056723	<0.05	2.19538
14967733			<0.05	2.19527
15055292	XM_001492982 // LOC100060848 // Down syndrome critical region protein 3-like // --- //	LOC100060848	<0.05	2.19418
15052813	XM_001500839 // LOC100070261 // probable tRNA pseudouridine synthase 2-like // --- // 1	LOC100070261	<0.05	2.19406
15113421	XM_001489060 // LOC100050121 // small nuclear ribonucleoprotein Sm D3-like // --- // 10	LOC100050121	<0.05	2.19302
15087473	XM_001499579 // AP4B1 // adaptor-related protein complex 4, beta 1 subunit // --- // 10	AP4B1	<0.05	2.1924
15119451	XM_001490337 // LOC100050780 // serine/threonine-protein kinase RIO3-like // --- // 100	LOC100050780	<0.05	2.19219
15043689	ENSECAT00000026587 // LOC100052253 // perilipin-2-like // --- // 100052253 /// XM_00191	LOC100052253	<0.05	2.19176
14973066	XM_001502022 // DDB1 // damage-specific DNA binding protein 1, 127kDa // --- // 1000596	DDB1	<0.05	2.19168
15014004	ENSECAT00000019698 // LOC100058361 // 39S ribosomal protein L47, mitochondrial-like //	LOC100058361	<0.05	2.19118
15039784	ENSECAT00000018124 // LOC100053597 // bcl-2-like protein 1-like // --- // 100053597 ///	LOC100053597	<0.05	2.19039
15080051	---		<0.05	2.18998
14980162	---		<0.05	2.18974
15035114	XM_001492047 // SREK1 // splicing regulatory glutamine/lysine-rich protein 1 // --- //	SREK1	<0.05	2.18837
15004676	XM_001493517 // HSPH1 // heat shock 105kDa/110kDa protein 1 // --- // 100062150 /// ENS	HSPH1	<0.05	2.18824
15083667	---		<0.05	2.18803
15096361	ENSECAT00000025617 // LOC100146613 // serine/threonine-protein kinase 38-like // --- //	LOC100146613	<0.05	2.18757
14931847	---		<0.05	2.1875
14951145			<0.05	2.18725
14951565	---		<0.05	2.18653
14928835	---		<0.05	2.18619
14993122	XM_001497145 // LOC100067003 // endoplasmic reticulum lectin 1-like // --- // 100067003	LOC100067003	<0.05	2.18614
15126616			<0.05	2.18611
15122253	XM_001496257 // LOC100056859 // rRNA-processing protein UTP23 homolog // --- // 1000568	LOC100056859	<0.05	2.18527
15026516	XM_001503217 // TET2 // tet methylcytosine dioxygenase 2 // --- // 100073065 /// ENSECA	TET2	<0.05	2.18506
15048941	XM_001504017 // ZNF189 // zinc finger protein 189 // --- // 100054526 /// ENSECAT000000	ZNF189	<0.05	2.18479
15058963	XM_001916763 // LOC100147078 // ADP-ribosylation factor-binding protein GGA1-like // --	LOC100147078	<0.05	2.18405
15051929	ENSECAT00000022837 // LOC100058146 // proteasome-associated protein ECM29 homolog // --	LOC100058146	<0.05	2.18385
14982662	XM_001504199 // LOC100061972 // prefoldin subunit 1-like // --- // 100061972 /// ENSECA	LOC100061972	<0.05	2.18362
15017509	XM_001504019 // FGR // Gardner-Rasheed feline sarcoma viral (v-fgr) oncogene homolog //	FGR	<0.05	2.1834
15054889	XM_001499999 // LOC100066379 // ATP synthase-coupling factor 6, mitochondrial-like // -	LOC100066379	<0.05	2.18315
15055319	ENSECAT00000020307 // LOC100059615 // proteasome assembly chaperone 1-like // --- // 10	LOC100059615	<0.05	2.18314
14948644	ENSECAT00000008241 // BAZ1A // bromodomain adjacent to zinc finger domain, 1A // --- //	BAZ1A	<0.05	2.18205
15107406	XM_001504451 // LOC100054534 // e3 ubiquitin-protein ligase TRIM22-like // --- // 10005	LOC100054534	<0.05	2.18191
14977467	---		<0.05	2.18173
15032832	ENSECAT00000018957 // SSBP4 // single stranded DNA binding protein 4 // --- // ---	SSBP4	<0.05	2.1816
14958200	---		<0.05	2.18159
14930501	---		<0.05	2.18042
14989648	XM_001497080 // PSME4 // proteasome (prosome, macropain) activator subunit 4 // --- //	PSME4	<0.05	2.18035
14990817	XM_001503315 // PUM2 // pumilio homolog 2 (Drosophila) // --- // 100056370 /// ENSECAT0	PUM2	<0.05	2.17977
15049721	NM_001257178 // ZBTB34 // zinc finger and BTB domain containing 34 // --- // 100070589	ZBTB34	<0.05	2.17974
15016753	ENSECAT00000015888 // LOC100068889 // rhomboid-related protein 2-like // --- // 1000688	LOC100068889	<0.05	2.17949
15068280	XM_003364694 // LOC100070415 // probable tRNA (uracil-O(2)-)-methyltransferase-like //	LOC100070415	<0.05	2.17924
14990088	XM_001500870 // PRKD3 // protein kinase D3 // --- // 100054187 /// ENSECAT00000009228 /	PRKD3	<0.05	2.17897
14928565	---		<0.05	2.17885
15104403	ENSECAT00000020171 // SIK2 // salt-inducible kinase 2 // --- // 100070391 /// XM_001500	SIK2	<0.05	2.17877
15112785	XM_001504941 // COPB1 // coatomer protein complex, subunit beta 1 // --- // 100056177 /	COPB1	<0.05	2.17817
14926143	---		<0.05	2.17812
14981880	ENSECAT00000004065 // C5orf54 // chromosome 5 open reading frame 54 // --- // ---	C5orf54	<0.05	2.17791
14991343	XM_001493146 // GCC2 // GRIP and coiled-coil domain containing 2 // --- // 100061081 //	GCC2	<0.05	2.17781
14938058	ENSECAT00000021502 // LOC100069511 // cAMP-regulated phosphoprotein 19-like // --- // 1	LOC100069511	<0.05	2.17734
14985975	ENSECAT00000019747 // DDX46 // DEAD (Asp-Glu-Ala-Asp) box polypeptide 46 // --- // 1000	DDX46	<0.05	2.17695
14943738	ENSECAT00000010965 // COG2 // component of oligomeric golgi complex 2 // --- // 1000653	COG2	<0.05	2.17637
14933535	XM_001501044 // GOT1 // glutamic-oxaloacetic transaminase 1, soluble (aspartate aminotr	GOT1	<0.05	2.17446
14932227	---		<0.05	2.17381
15118497	ENSECAT00000010509 // MAPKAPK5 // mitogen-activated protein kinase-activated protein ki	MAPKAPK5	<0.05	2.17381
15011849	ENSECAT00000022513 // KLHL24 // kelch-like 24 (Drosophila) // --- // 100058815 /// XM_0	KLHL24	<0.05	2.17321
14958029	ENSECAT00000020648 // LOC100054966 // NAD-dependent deacetylase sirtuin-7-like // --- /	LOC100054966	<0.05	2.17274
14961595	ENSECAT00000024855 // LOC100629929 // zinc finger HIT domain-containing protein 3-like	LOC100629929	<0.05	2.17247
15087392	XM_001500064 // LOC100059469 // GTPase NRas-like // --- // 100059469 /// ENSECAT0000001	LOC100059469	<0.05	2.17206
14995095	XM_001494063 // LOC100051933 // transmembrane protein 111-like // --- // 100051933 ///	LOC100051933	<0.05	2.17199
14933622	XM_001501347 // LOC100060949 // arginine vasopressin-induced protein 1-like // --- // 1	LOC100060949	<0.05	2.17108
15064321	ENSECAT00000008347 // LOC100062499 // ubiquinone biosynthesis protein COQ9, mitochondri	LOC100062499	<0.05	2.1708
15033653	XM_001499483 // NUP155 // nucleoporin 155kDa // --- // 100053405 /// ENSECAT00000007933	NUP155	<0.05	2.17068
15115669	---		<0.05	2.17012
15018529	ENSECAT00000014686 // LOC100056045 // uncharacterized protein KIAA2013 homolog // --- /	LOC100056045	<0.05	2.16928
14998400	XM_001493057 // U2SURP // U2 snRNP-associated SURP domain containing // --- // 10005118	U2SURP	<0.05	2.16887
14941023	ENSECAT00000000264 // LOC100053051 // glutaredoxin-3-like // --- // 100053051 /// XM_00	LOC100053051	<0.05	2.16883
14978750	ENSECAT00000004244 // BAZ1B // bromodomain adjacent to zinc finger domain, 1B // --- //	BAZ1B	<0.05	2.16878
15004026	XM_001495972 // ABHD13 // abhydrolase domain containing 13 // --- // 100065368 /// ENSE	ABHD13	<0.05	2.16846
15103723	XM_001916832 // LOC100065229 // vimentin-type intermediate filament-associated coiled-c	LOC100065229	<0.05	2.16798
14964801	XM_001916362 // COG1 // component of oligomeric golgi complex 1 // --- // 100061294 ///	COG1	<0.05	2.16691
15005959	ENSECAT00000022446 // COL4A1 // collagen, type IV, alpha 1 // --- // 100066148 /// XM_0	COL4A1	<0.05	2.16486
14930357	---		<0.05	2.16432
15127342	ENSECAT00000018712 // LOC100060382 // eukaryotic translation initiation factor 2 subuni	LOC100060382	<0.05	2.16345
15012475	XM_001499603 // LOC100069887 // UBX domain-containing protein 7-like // --- // 10006988	LOC100069887	<0.05	2.16297
14989284	XM_001494344 // SPRED2 // sprouty-related, EVH1 domain containing 2 // --- // 100062947	SPRED2	<0.05	2.16255
14930135	---		<0.05	2.16244
14928155	---		<0.05	2.1622
14995211	XM_001496698 // CRBN // cereblon // --- // 100052958 /// ENSECAT00000016376 // CRBN //	CRBN	<0.05	2.16144
15063854	NM_001163825 // PHKB // phosphorylase kinase, beta // --- // 100050040 /// ENSECAT00000	PHKB	<0.05	2.16094
15031093	ENSECAT00000021335 // VPS52 // vacuolar protein sorting 52 homolog (S. cerevisiae) // -	VPS52	<0.05	2.16076
15071677	ENSECAT00000013557 // ATP10D // ATPase, class V, type 10D // --- // ---	ATP10D	<0.05	2.16073
14925643	---		<0.05	2.16064
15006839	ENSECAT00000019811 // GPD2 // glycerol-3-phosphate dehydrogenase 2 (mitochondrial) // -	GPD2	<0.05	2.16044
14977745	XM_001915417 // TMEM8A // transmembrane protein 8A // --- // 100066625 /// ENSECAT00000	TMEM8A	<0.05	2.15986
15127619	XM_001489437 // USP9X // ubiquitin specific peptidase 9, X-linked // --- // 100050489 /	USP9X	<0.05	2.15965
15115436	XM_003365506 // LOC100630108 // serine/threonine-protein phosphatase PGAM5, mitochondri	LOC100630108	<0.05	2.15949
14975052	ENSECAT00000024438 // ZKSCAN1 // zinc finger with KRAB and SCAN domains 1 // --- // 100	ZKSCAN1	<0.05	2.15832
15065494	ENSECAT00000012579 // GAN // gigaxonin // --- // 100069891 /// XM_001499608 // GAN // g	GAN	<0.05	2.15827
14987821	ENSECAT00000020846 // LOC100060911 // ADP-ribosylation factor 1-like // --- // 10006091	LOC100060911	<0.05	2.15817
15097379	XM_001504569 // LOC100064590 // coatomer subunit zeta-1-like // --- // 100064590 /// EN	LOC100064590	<0.05	2.15814
15012355	ENSECAT00000026313 // OPA1 // optic atrophy 1 (autosomal dominant) // --- // 100059993	OPA1	<0.05	2.15813
14988231			<0.05	2.15787
14940052	ENSECAT00000016572 // LOC100056354 // mitochondrial inner membrane protein OXA1L-like /	LOC100056354	<0.05	2.15759
15012405	ENSECAT00000013029 // DLG1 // discs, large homolog 1 (Drosophila) // --- // 100060094 /	DLG1	<0.05	2.15716
15105451	ENSECAT00000005944 // LOC100072441 // protein FAM118B-like // --- // 100072441 /// XM_0	LOC100072441	<0.05	2.15675
15093971	ENSECAT00000008039 // SMARCAL1 // SWI/SNF related, matrix associated, actin dependent r	SMARCAL1	<0.05	2.1561
14986056	XM_001918224 // HSPA4 // heat shock 70kDa protein 4 // --- // 100072809 /// ENSECAT0000	HSPA4	<0.05	2.15552
14937043	ENSECAT00000008566 // NPTN // neuroplastin // --- // 100052106 /// XM_001493720 // NPTN	NPTN	<0.05	2.15511
14930143	---		<0.05	2.15501

15006613	ENSECAT00000014543 // SPOPL // speckle-type POZ protein-like // --- // 100056551 /// XM	SPOPL	<0.05	2.15457
15096349	ENSECAT00000017757 // LOC100064619 // FGFR1 oncogene partner 2 homolog // --- // 100064	LOC100064619	<0.05	2.15391
14965039	ENSECAT00000009384 // MAP3K3 // mitogen-activated protein kinase kinase kinase 3 // ---	MAP3K3	<0.05	2.15315
14993281	ENSECAT00000015683 // EPAS1 // endothelial PAS domain protein 1 // --- // 100068622 ///	EPAS1	<0.05	2.15192
15014525	XM_001499100 // ACAP2 // ArfGAP with coiled-coil, ankyrin repeat and PH domains 2 // --	ACAP2	<0.05	2.15171
15093394	XM_001498949 // LOC100053370 // tRNA wybutosine-synthesizing protein 3 homolog // --- /	LOC100053370	<0.05	2.15043
15052575			<0.05	2.15034
14929837	---		<0.05	2.14883
15028723	ENSECAT00000006940 // LOC100067213 // kelch domain-containing protein 3-like // --- //	LOC100067213	<0.05	2.14816
15119706	XM_001493386 // ZFP161 // zinc finger protein 161 homolog (mouse) // --- // 100051583 /	ZFP161	<0.05	2.14779
15047576	XM_001492123 // LOC100059578 // SNW domain-containing protein 1-like // --- // 10005957	LOC100059578	<0.05	2.1474
15086135	XM_001500077 // LOC100057746 // protein SMG5-like // --- // 100057746 /// ENSECAT000000	LOC100057746	<0.05	2.14735
14955261	XM_001493330 // LOC100056315 // nuclear pore glycoprotein p62-like // --- // 100056315	LOC100056315	<0.05	2.14588
15102236	ENSECAT00000017267 // SPRYD3 // SPRY domain containing 3 // --- // 100056536 /// XM_001	SPRYD3	<0.05	2.14575
15072966	XM_001492655 // FH // fumarate hydratase // --- // 100060381 /// ENSECAT00000022289 //	FH	<0.05	2.14558
14925743	---		<0.05	2.14554
15002716	XM_001493357 // USPL1 // ubiquitin specific peptidase like 1 // --- // 100062086 /// EN	USPL1	<0.05	2.14472
14996559	XM_001498330 // LOC100063232 // glutamine-rich protein 1-like // --- // 100063232 /// E	LOC100063232	<0.05	2.14471
15075529	XM_001501971 // LATS1 // LATS, large tumor suppressor, homolog 1 (Drosophila) // --- //	LATS1	<0.05	2.14455
15013088	XM_001502881 // SPICE1 // spindle and centriole associated protein 1 // --- // 10006133	SPICE1	<0.05	2.14449
15109600	ENSECAT00000012956 // PPP2R1B // protein phosphatase 2, regulatory subunit A, beta // -	PPP2R1B	<0.05	2.14406
15070386	---		<0.05	2.14383
14935281	XM_001915071 // RBM34 // RNA binding motif protein 34 // --- // 100146167 /// ENSECAT00	RBM34	<0.05	2.14382
15085726	XM_001504428 // DCAF8 // DDB1 and CUL4 associated factor 8 // --- // 100053669 /// ENSE	DCAF8	<0.05	2.14341
15011858	ENSECAT00000009103 // LOC100066877 // YEATS domain-containing protein 2-like // --- //	LOC100066877	<0.05	2.14318
15081783	XM_001496289 // SNX13 // sorting nexin 13 // --- // 100066218 /// ENSECAT00000023365 //	SNX13	<0.05	2.14294
14927209	---		<0.05	2.14271
15055130	ENSECAT00000006588 // SYNJ1 // synaptojanin 1 // --- // 100052605 /// ENSECAT0000000659	SYNJ1	<0.05	2.14167
15115047	XM_001498352 // LOC100059174 // general transcription factor IIH subunit 3-like // ---	LOC100059174	<0.05	2.14166
14929677	---		<0.05	2.14075
14975160	ENSECAT00000017199 // AGFG2 // ArfGAP with FG repeats 2 // --- // 100068998	AGFG2	<0.05	2.14025
15034792	XM_001503329 // FKBP8 // FK506 binding protein 8, 38kDa // --- // 100069569 /// ENSECAT	FKBP8	<0.05	2.14012
15109512	ENSECAT00000008687 // LOC100069736 // protein NPAT-like // --- // 100069736 /// XM_0019	LOC100069736	<0.05	2.13973
14956999	XM_001504012 // ZBTB24 // zinc finger and BTB domain containing 24 // --- // 100066674	ZBTB24	<0.05	2.13942
15036366	XM_001490637 // LOC100057209 // DNA replication complex GINS protein PSF1-like // --- /	LOC100057209	<0.05	2.13884
15118011	ENSECAT00000009561 // UBE3B // ubiquitin protein ligase E3B // --- // 100059725 /// XM_	UBE3B	<0.05	2.13836
14935112	ENSECAT00000023505 // HEATR1 // HEAT repeat containing 1 // --- // 100050411 /// XM_001	HEATR1	<0.05	2.13828
15063264	XM_003364353 // LOC100055759 // COMM domain-containing protein 3-like // --- // 1000557	LOC100055759	<0.05	2.13819
14975148	XM_001498760 // MEPCE // methylphosphate capping enzyme // --- // 100068939 /// ENSECAT	MEPCE	<0.05	2.13732
15031900	XM_001502799 // MUT // methylmalonyl CoA mutase // --- // 100055925 /// ENSECAT00000008	MUT	<0.05	2.13678
15052029	ENSECAT00000023356 // ROD1 // polypyrimidine tract binding protein 3 // --- // 10005741	ROD1	<0.05	2.13603
15113166	XM_001502214 // LOC100072314 // protein lin-7 homolog C-like // --- // 100072314 /// EN	LOC100072314	<0.05	2.13542
15120030	ENSECAT00000004752 // EPG5 // ectopic P-granules autophagy protein 5 homolog (C. elegan	EPG5	<0.05	2.13541
14983423	ENSECAT00000019193 // LOC100064283 // e3 ubiquitin-protein ligase TRIM36-like // --- //	LOC100064283	<0.05	2.13411
15091346	ENSECAT00000024229 // INTS3 // integrator complex subunit 3 // --- // 100056524 /// XM_	INTS3	<0.05	2.13406
14984203	ENSECAT00000016268 // LOC100065744 // survival motor neuron protein-like // --- // 1000	LOC100065744	<0.05	2.13394
14925213	---		<0.05	2.13309
15115880	ENSECAT00000015587 // SMCHD1 // structural maintenance of chromosomes flexible hinge do	SMCHD1	<0.05	2.13257
14993161	ENSECAT00000022186 // KLRAQ1 // protein phosphatase 1, regulatory subunit 21 // --- //	KLRAQ1	<0.05	2.13161
14930235	---		<0.05	2.13148
15124385	ENSECAT00000026272 // TAF2 // TAF2 RNA polymerase II, TATA box binding protein (TBP)-as	TAF2	<0.05	2.13123
15026441	ENSECAT00000022814 // LOC100072967 // cytochrome P450 2U1-like // --- // 100072967 ///	LOC100072967	<0.05	2.13116
15055763	XM_001488888 // LOC100053953 // RING finger protein 170-like // --- // 100053953 /// EN	LOC100053953	<0.05	2.13061
14945994	ENSECAT00000020910 // ARIH1 // ariadne homolog, ubiquitin-conjugating enzyme E2 binding	ARIH1	<0.05	2.12916
14965839	XM_001918050 // MSL1 // male-specific lethal 1 homolog (Drosophila) // --- // 100067838	MSL1	<0.05	2.12866
15006280	XM_001488507 // LOC100050708 // TFIIH basal transcription factor complex helicase XPB s	LOC100050708	<0.05	2.12841
14978770	ENSECAT00000016225 // LOC100061703 // b-cell CLL/lymphoma 7 protein family member B-lik	LOC100061703	<0.05	2.12827
15029823	XM_001495773 // LOC100065066 // coiled-coil domain-containing protein 90A, mitochondria	LOC100065066	<0.05	2.12793
14936384	XM_001489692 // ASB7 // ankyrin repeat and SOCS box containing 7 // --- // 100050201 //	ASB7	<0.05	2.1279
15005148	ENSECAT00000018279 // LOC100059040 // kidney mitochondrial carrier protein 1-like // --	LOC100059040	<0.05	2.12768
15008205	XM_001497300 // LOC100067195 // bone morphogenetic protein receptor type-2-like // ---	LOC100067195	<0.05	2.12665
15053069	ENSECAT00000010487 // TSC1 // tuberous sclerosis 1 // --- // 100066615	TSC1	<0.05	2.12642
14976850	XM_001489900 // GSPT1 // G1 to S phase transition 1 // --- // 100055960 /// XM_00336273	GSPT1	<0.05	2.12478
14963398	ENSECAT00000027173 // MAP2K4 // mitogen-activated protein kinase kinase 4 // --- // 100	MAP2K4	<0.05	2.12403
14926589	---		<0.05	2.1235
14948005	XM_001505163 // METTL3 // methyltransferase like 3 // --- // 100059413 /// ENSECAT00000	METTL3	<0.05	2.12298
14978347	ENSECAT00000014964 // TRIM4 // tripartite motif containing 4 // --- // ---	TRIM4	<0.05	2.1229
15042461	XM_001497125 // LOC100063025 // serine palmitoyltransferase 1-like // --- // 100063025	LOC100063025	<0.05	2.1224
14932107	---		<0.05	2.12212
15126715	ENSECAT00000000001 // R3HDM4 // R3H domain containing 4 // --- // ---	R3HDM4	<0.05	2.12113
15092647	XM_001918140 // LOC100055854 // RNA 3'-terminal phosphate cyclase-like // --- // 100055	LOC100055854	<0.05	2.12104
14928139	---		<0.05	2.12032
14973325	---		<0.05	2.11981
14940388	XM_001489169 // G2E3 // G2/M-phase specific E3 ubiquitin protein ligase // --- // 10004	G2E3	<0.05	2.11889
15042696	---		<0.05	2.11776
15029308	XM_001503363 // PHF3 // PHD finger protein 3 // --- // 100057084 /// XM_001503364 // PH	PHF3	<0.05	2.11715
14988361	---		<0.05	2.1165
15039951	XM_001501293 // NCOA6 // nuclear receptor coactivator 6 // --- // 100054476 /// ENSECAT	NCOA6	<0.05	2.11608
15077251	ENSECAT00000009328 // CROT // carnitine O-octanoyltransferase // --- // 100050111 /// X	CROT	<0.05	2.11522
15021189	XM_001503578 // PAPSS1 // 3'-phosphoadenosine 5'-phosphosulfate synthase 1 // --- // 10	PAPSS1	<0.05	2.11498
15001061	ENSECAT00000016498 // EXOG // endo/exonuclease (5'-3'), endonuclease G-like // --- // 1	EXOG	<0.05	2.11489
14925093	---		<0.05	2.11484
15034254	XM_001499057 // LOC100053747 // dnaJ homolog subfamily A member 1-like // --- // 100053	LOC100053747	<0.05	2.11451
15014145	ENSECAT00000008988 // ABCC5 // ATP-binding cassette, sub-family C (CFTR/MRP), member 5	ABCC5	<0.05	2.11439
15068192	ENSECAT00000002454 // MRFAP1L1 // Morf4 family associated protein 1-like 1 // --- // --	MRFAP1L1	<0.05	2.11416
14940514	ENSECAT00000012948 // LOC100050727 // protein FAM177A1-like // --- // 100050727 /// XM_	LOC100050727	<0.05	2.11403
15017212	XM_001499806 // YARS // tyrosyl-tRNA synthetase // --- // 100070128 /// ENSECAT00000010	YARS	<0.05	2.11325
15113773	ENSECAT00000025242 // ASCC2 // activating signal cointegrator 1 complex subunit 2 // --	ASCC2	<0.05	2.11316
14951389	XM_001494108 // LOC100062997 // UPF0510 protein INM02-like // --- // 100062997 /// ENSE	LOC100062997	<0.05	2.11304
15099605	XM_001489654 // IL17RA // interleukin 17 receptor A // --- // 100055511 /// ENSECAT0000	IL17RA	<0.05	2.11293
14947963	XM_003363541 // CHD8 // chromodomain helicase DNA binding protein 8 // --- // 100059337	CHD8	<0.05	2.11252
15064988	ENSECAT00000000227 // LOC100067168 // cytochrome b5 type B-like // --- // 100067168 ///	LOC100067168	<0.05	2.11197
15026504	---		<0.05	2.11038
15016599	XM_001503156 // FOXJ3 // forkhead box J3 // --- // 100053693 /// ENSECAT00000009015 //	FOXJ3	<0.05	2.1101
14928153	---		<0.05	2.10896
15067660	ENSECAT00000013372 // PDS5A // PDS5, regulator of cohesion maintenance, homolog A (S. c	PDS5A	<0.05	2.10886
15120225	XM_001499125 // ACAA2 // acetyl-CoA acyltransferase 2 // --- // 100069345 /// ENSECAT00	ACAA2	<0.05	2.10876
15122997	XM_001488288 // WWP1 // WW domain containing E3 ubiquitin protein ligase 1 // --- // 10	WWP1	<0.05	2.10873
15130244	XM_001499390 // LOC100069653 // myotubularin-related protein 1-like // --- // 100069653	LOC100069653	<0.05	2.10855
15040147	XM_001501819 // RBM39 // RNA binding motif protein 39 // --- // 100054976 /// XM_001501	RBM39	<0.05	2.10759
15022437	XM_001503545 // LOC100054471 // probable U3 small nucleolar RNA-associated protein 11-l	LOC100054471	<0.05	2.10759
14966974	XM_001501508 // LOC100071676 // zinc finger protein 830-like // --- // 100071676 /// EN	LOC100071676	<0.05	2.10671
15064821	XM_001498725 // DUS2L // dihydrouridine synthase 2-like, SMM1 homolog (S. cerevisiae) /	DUS2L	<0.05	2.10601
14937328	---		<0.05	2.10567
14937329			<0.05	2.10567
15093203	ENSECAT00000024421 // LOC100052828 // cAMP-dependent protein kinase catalytic subunit b	LOC100052828	<0.05	2.10548

15092544	XM_001492903 // PRPF38B // PRP38 pre-mRNA processing factor 38 (yeast) domain containin	PRPF38B	<0.05	2.10535
14992917	ENSECAT00000006018 // LOC100064970 // activator of 90 kDa heat shock protein ATPase hom	LOC100064970	<0.05	2.105
15115655	ENSECAT00000018481 // LOC100050392 // protein spire homolog 1-like // --- // 100050392	LOC100050392	<0.05	2.1042
15103852	ENSECAT00000011283 // XAB2 // XPA binding protein 2 // --- // 100060674 /// XM_00191693	XAB2	<0.05	2.10404
14994004	XM_001502317 // SNX17 // sorting nexin 17 // --- // 100055239 /// XM_003363022 // SNX17	SNX17	<0.05	2.10368
15056738			<0.05	2.10216
15031361	XM_001494965 // STK38 // serine/threonine kinase 38 // --- // 100063891 /// ENSECAT0000	STK38	<0.05	2.10154
14968611	XM_001503188 // LOC100073047 // vesicle-associated membrane protein 2-like // --- // 10	LOC100073047	<0.05	2.10103
15064466	ENSECAT00000008835 // SETD6 // SET domain containing 6 // --- // --- /// ENSECAT0000000	SETD6	<0.05	2.10084
15107038	XM_001498480 // LOC100052144 // mitochondrial uncoupling protein 2-like // --- // 10005	LOC100052144	<0.05	2.10069
14940229	XM_001490663 // RNF31 // ring finger protein 31 // --- // 100051661 /// ENSECAT00000015	RNF31	<0.05	2.10036
14979567	---		<0.05	2.10029
15040168	XM_001501876 // LOC100055060 // SCAN domain-containing protein 1-like // --- // 1000550	LOC100055060	<0.05	2.10022
14929833	---		<0.05	2.09896
15061219	NM_001242478 // RANGAP1 // Ran GTPase activating protein 1 // --- // 100628308 /// ENSE	RANGAP1	<0.05	2.09883
14990219	XM_001500160 // LOC100070497 // protein FAM98A-like // --- // 100070497 /// ENSECAT0000	LOC100070497	<0.05	2.09882
14961638	ENSECAT00000008813 // AATF // apoptosis antagonizing transcription factor // --- // 100	AATF	<0.05	2.09851
14967022	XM_001918320 // LOC100071770 // 26S proteasome non-ATPase regulatory subunit 11-like //	LOC100071770	<0.05	2.0985
14930509	---		<0.05	2.09749
14954698	XM_001502832 // LOC100065786 // 40S ribosomal protein S17-like // --- // 100065786 ///	LOC100065786	<0.05	2.09726
15057836	XM_001488032 // LOC100050038 // putative ataxin-7-like protein 3B-like // --- // 100050	LOC100050038	<0.05	2.09701
14932151	---		<0.05	2.09642
15050562	AF508309 // RPL7A // ribosomal protein L7a // --- // 100033932	RPL7A	<0.05	2.09568
14928709	---		<0.05	2.09531
14985204	ENSECAT00000018788 // NDST1 // N-deacetylase/N-sulfotransferase (heparan glucosaminyl)	NDST1	<0.05	2.09486
15057715	ENSECAT00000017030 // ARHGEF10 // Rho guanine nucleotide exchange factor (GEF) 10 // --	ARHGEF10	<0.05	2.09486
15058331	ENSECAT00000026768 // APAF1 // apoptotic peptidase activating factor 1 // --- // 100052	APAF1	<0.05	2.09462
15060866			<0.05	2.09453
15016052	ENSECAT00000022730 // EPS15 // epidermal growth factor receptor pathway substrate 15 //	EPS15	<0.05	2.09451
15014658	XM_001500039 // UMPS // uridine monophosphate synthetase // --- // 100070386 /// ENSECA	UMPS	<0.05	2.09329
14927219	---		<0.05	2.09298
14928375	---		<0.05	2.09288
14928463	---		<0.05	2.09288
14928637	---		<0.05	2.09283
15119329	XM_001488687 // RNMT // RNA (guanine-7-) methyltransferase // --- // 100050190 /// ENSE	RNMT	<0.05	2.09224
15013585	ENSECAT00000009151 // LOC100050292 // cyclin-L1-like // --- // 100050292 /// XM_0019153	LOC100050292	<0.05	2.09199
14996909	XM_001499841 // SCAP // SREBF chaperone // --- // 100054081 /// ENSECAT00000026470 // S	SCAP	<0.05	2.09199
14967415	ENSECAT00000012832 // FLOT2 // flotillin 2 // --- // 100072090 /// XM_001501952 // FLOT	FLOT2	<0.05	2.09189
15134424	---		<0.05	2.09175
15030704	XM_001491355 // LOC100058853 // chloride intracellular channel protein 1-like // --- //	LOC100058853	<0.05	2.09095
14975401	ENSECAT00000026013 // SH2B2 // SH2B adaptor protein 2 // --- // 100060578 /// XM_001916	SH2B2	<0.05	2.09091
15097862	XM_001488891 // MARS // methionyl-tRNA synthetase // --- // 100053373 /// ENSECAT000000	MARS	<0.05	2.0908
14976655	---		<0.05	2.09064
15090171	---		<0.05	2.09035
15124125	ENSECAT00000023585 // SLC25A32 // solute carrier family 25 (mitochondrial folate carrie	SLC25A32	<0.05	2.08736
14978575	XM_001504456 // PLOD3 // procollagen-lysine, 2-oxoglutarate 5-dioxygenase 3 // --- // 1	PLOD3	<0.05	2.08615
14930923	---		<0.05	2.08592
15060750			<0.05	2.08567
15131157	XM_001494160 // KLHL15 // kelch-like 15 (Drosophila) // --- // 100052043 /// ENSECAT000	KLHL15	<0.05	2.08517
15066315	DQ825759 // ABCG2 // ATP-binding cassette, sub-family G (WHITE), member 2 // --- // 100	ABCG2	<0.05	2.08418
15060852	XM_001500445 // LOC100054528 // eukaryotic translation initiation factor 3 subunit D-li	LOC100054528	<0.05	2.08384
15126606			<0.05	2.08356
15101117	XM_001503000 // IPO8 // importin 8 // --- // 100064845 /// ENSECAT00000014160 // IPO8 /	IPO8	<0.05	2.08353
14985087	ENSECAT00000018194 // LARP1 // La ribonucleoprotein domain family, member 1 // --- // 1	LARP1	<0.05	2.08325
14932195	---		<0.05	2.08272
15108867	XM_001496144 // KHSRP // KH-type splicing regulatory protein // --- // 100065608 /// EN	KHSRP	<0.05	2.08234
14976768	XM_001489539 // LOC100050501 // limkain-b1-like // --- // 100050501 /// ENSECAT00000014	LOC100050501	<0.05	2.08211
14967728			<0.05	2.08152
15136440	---		<0.05	2.08134
15045695	ENSECAT00000005327 // UBR7 // ubiquitin protein ligase E3 component n-recognin 7 (putat	UBR7	<0.05	2.08084
15119990	ENSECAT00000024782 // LOC100067571 // tubulin polyglutamylase complex subunit 2-like //	LOC100067571	<0.05	2.08065
15048622	ENSECAT00000009721 // LOC100066566 // glyoxylate reductase/hydroxypyruvate reductase-li	LOC100066566	<0.05	2.08062
15125640	XM_001495962 // HECA // headcase homolog (Drosophila) // --- // 100065354 /// ENSECAT00	HECA	<0.05	2.07941
14938280	---		<0.05	2.07917
15114349	ENSECAT00000007800 // LOC100054898 // WD repeat and SOCS box-containing protein 2-like	LOC100054898	<0.05	2.07877
14985534	ENSECAT00000003450 // RNF14 // ring finger protein 14 // --- // ---	RNF14	<0.05	2.07842
14992679	XM_001490697 // LOC100060958 // u4/U6.U5 small nuclear ribonucleoprotein 27 kDa protein	LOC100060958	<0.05	2.07835
15043173	ENSECAT00000022042 // LOC100055329 // uncharacterized protein C9orf85 homolog // --- //	LOC100055329	<0.05	2.07824
15101899	XM_001915611 // LOC100059723 // probable methyltransferase BCDIN3D-like // --- // 10005	LOC100059723	<0.05	2.07811
15135586	---		<0.05	2.07808
15090478	XM_001503876 // LOC100066365 // upstream stimulatory factor 1-like // --- // 100066365	LOC100066365	<0.05	2.07766
15054677	XM_001489273 // PRMT2 // protein arginine methyltransferase 2 // --- // 100054798 /// E	PRMT2	<0.05	2.07659
15115769	XM_001491258 // ESCO1 // establishment of cohesion 1 homolog 1 (S. cerevisiae) // --- /	ESCO1	<0.05	2.07645
15068371	ENSECAT00000002811 // LOC100053044 // uncharacterized LOC100053044 // --- // 100053044	LOC100053044	<0.05	2.07614
14943424	XM_001503827 // ECD // ecdysoneless homolog (Drosophila) // --- // 100063816 /// ENSECA	ECD	<0.05	2.07579
15002056	ENSECAT00000012259 // ATR // ataxia telangiectasia and Rad3 related // --- // 100062529	ATR	<0.05	2.07529
14929961	---		<0.05	2.07513
14925025	---		<0.05	2.07489
15071375	ENSECAT00000014599 // LOC100053708 // DNA-directed RNA polymerase II subunit RPB2-like	LOC100053708	<0.05	2.07488
14977041	ENSECAT00000012599 // NAGPA // N-acetylglucosamine-1-phosphodiester alpha-N-acetylgluco	NAGPA	<0.05	2.07471
14975442	XM_001504476 // LOC100059731 // 14-3-3 protein gamma-like // --- // 100059731 /// ENSEC	LOC100059731	<0.05	2.07448
14929579	---		<0.05	2.07426
14953080	ENSECAT00000015085 // NCOA7 // nuclear receptor coactivator 7 // --- // ---	NCOA7	<0.05	2.07417
15117372	ENSECAT00000018670 // LOC100050855 // crk-like protein-like // --- // 100050855 /// XM_	LOC100050855	<0.05	2.0738
15101335	ENSECAT00000012553 // LOC100054055 // YY1-associated factor 2-like // --- // 100054055	LOC100054055	<0.05	2.07377
15030606	---		<0.05	2.07335
14962379	XM_001918354 // PAFAH1B1 // platelet-activating factor acetylhydrolase 1b, regulatory s	PAFAH1B1	<0.05	2.07294
15107719	XM_001500935 // LOC100071230 // switch-associated protein 70-like // --- // 100071230 /	LOC100071230	<0.05	2.0729
15031721	XM_001918236 // XPO5 // exportin 5 // --- // 100067497 /// ENSECAT00000003174 // XPO5 /	XPO5	<0.05	2.07283
15093950	ENSECAT00000017868 // XRCC5 // X-ray repair complementing defective repair in Chinese h	XRCC5	<0.05	2.07259
15131620	ENSECAT00000000234 // SLC35A2 // solute carrier family 35 (UDP-galactose transporter),	SLC35A2	<0.05	2.07254
14973805			<0.05	2.07228
14971483	NM_001081937 // AIP // aryl hydrocarbon receptor interacting protein // --- // 10003414	AIP	<0.05	2.07219
15006113	ENSECAT00000009719 // LOC100067830 // u3 small nucleolar ribonucleoprotein protein IMP4	LOC100067830	<0.05	2.07152
15017409	ENSECAT00000025685 // LOC100070525 // syndecan-3-like // --- // 100070525 /// XM_001500	LOC100070525	<0.05	2.06933
14932047	---		<0.05	2.06885
15053773	XM_001915095 // GABPA // GA binding protein transcription factor, alpha subunit 60kDa /	GABPA	<0.05	2.06793
15036139	XM_001501048 // LOC100071310 // methionine synthase reductase-like // --- // 100071310	LOC100071310	<0.05	2.06789
14928369	---		<0.05	2.06728
14928457	---		<0.05	2.06728
14949709	XM_001495128 // ZNF829 // zinc finger protein 829 // --- // 100061841 /// ENSECAT000000	ZNF829	<0.05	2.0672
14978952	ENSECAT00000011515 // LOC100629446 // vitamin K epoxide reductase complex subunit 1-lik	LOC100629446	<0.05	2.06719
15092580	ENSECAT00000022478 // LOC100054806 // RNA-binding protein 40-like // --- // 100054806 /	LOC100054806	<0.05	2.06674
14971861	XM_001494530 // LOC100056151 // CD151 antigen-like // --- // 100056151 /// ENSECAT00000	LOC100056151	<0.05	2.06668
15015902	XM_001493380 // LOC100061450 // enoyl-CoA hydratase domain-containing protein 2, mitoch	LOC100061450	<0.05	2.06652
15018401	XM_001488662 // FBXO42 // F-box protein 42 // --- // 100053402 /// ENSECAT00000019734 /	FBXO42	<0.05	2.06631

14967254	ENSECAT00000014913 // POLDIP2 // polymerase (DNA-directed), delta interacting protein 2	POLDIP2	<0.05	2.0661
14985124	ENSECAT00000018975 // MFAP3 // microfibrillar-associated protein 3 // --- // 100071417	MFAP3	<0.05	2.06588
15117284	XM_001489018 // LOC100054251 // cationic amino acid transporter 4-like // --- // 100054	LOC100054251	<0.05	2.06532
15017354	ENSECAT00000022702 // LOC100056122 // peflin-like // --- // 100056122 /// XM_001503887	LOC100056122	<0.05	2.06404
14952375	---		<0.05	2.06396
14951144			<0.05	2.06394
15056743	XM_001488824 // HOOK3 // hook homolog 3 (Drosophila) // --- // 100053799 /// ENSECAT000	HOOK3	<0.05	2.06361
15017453	XM_001500352 // LOC100070682 // transcription initiation factor TFIID subunit 12-like /	LOC100070682	<0.05	2.06359
15123661	XM_001487951 // MCM4 // minichromosome maintenance complex component 4 // --- // 100051	MCM4	<0.05	2.06314
15003831	ENSECAT00000013783 // RAP2A // RAP2A, member of RAS oncogene family // --- // ---	RAP2A	<0.05	2.06293
15083683	ENSECAT00000024323 // LOC100064812 // histone-lysine N-methyltransferase MLL3-like // -	LOC100064812	<0.05	2.06247
14948331	XM_001489831 // LOC100051307 // NEDD8-like // --- // 100051307 /// ENSECAT00000020444 /	LOC100051307	<0.05	2.06235
14946749	ENSECAT00000012768 // LOC100055565 // DTW domain-containing protein 1-like // --- // 10	LOC100055565	<0.05	2.06202
15045306	XM_001490655 // LOC100057247 // translation initiation factor eIF-2B subunit beta-like	LOC100057247	<0.05	2.06086
15001745	ENSECAT00000016925 // TOPBP1 // topoisomerase (DNA) II binding protein 1 // --- // ---	TOPBP1	<0.05	2.06082
15084578	XM_001916737 // LOC100052982 // glycosaminoglycan xylosylkinase-like // --- // 10005298	LOC100052982	<0.05	2.06079
15061096	XM_001501639 // LOC100055255 // josephin-1-like // --- // 100055255 /// ENSECAT00000016	LOC100055255	<0.05	2.05993
15079673	XM_001504627 // LOC100051217 // cullin-1-like // --- // 100051217 /// ENSECAT0000001061	LOC100051217	<0.05	2.05948
15041752	ENSECAT00000023310 // PTAR1 // protein prenyltransferase alpha subunit repeat containin	PTAR1	<0.05	2.05913
14989117	XM_001492476 // LOC100060120 // protein TEX261-like // --- // 100060120 /// ENSECAT0000	LOC100060120	<0.05	2.05873
14980166	ENSECAT00000010995 // ERCC4 // excision repair cross-complementing rodent repair defici	ERCC4	<0.05	2.05871
14928409	---		<0.05	2.05867
14928497	---		<0.05	2.05867
15049214	ENSECAT00000020834 // LOC100050369 // high affinity copper uptake protein 1-like // ---	LOC100050369	<0.05	2.05715
15134480	---		<0.05	2.057
14927257	---		<0.05	2.05685
15054355	XM_001916663 // LOC100057293 // ribosomal RNA processing protein 1 homolog A-like // --	LOC100057293	<0.05	2.05662
15060291	ENSECAT00000016715 // LOC100065070 // NADH dehydrogenase [ubiquinone] 1 alpha subcomple	LOC100065070	<0.05	2.05632
14968425			<0.05	2.05578
15002575	ENSECAT00000016748 // NUPL1 // nucleoporin like 1 // --- // ---	NUPL1	<0.05	2.05553
15116567	ENSECAT00000012726 // LOC100070157 // uncharacterized protein C18orf54-like // --- // 1	LOC100070157	<0.05	2.05474
14939313	XM_001501580 // ZNF770 // zinc finger protein 770 // --- // 100071735 /// ENSECAT000000	ZNF770	<0.05	2.05471
15077192	ENSECAT00000024482 // LANCL2 // LanC lantibiotic synthetase component C-like 2 (bacteri	LANCL2	<0.05	2.05429
14929709	---		<0.05	2.05358
15042691	ENSECAT00000020140 // LOC100629615 // uncharacterized protein C9orf102-like // --- // 1	LOC100629615	<0.05	2.0533
15094398	XM_001496411 // LOC100056818 // rhomboid domain-containing protein 1-like // --- // 100	LOC100056818	<0.05	2.05303
14930803	---		<0.05	2.05298
15112875	XM_001504950 // PIK3C2A // phosphoinositide-3-kinase, class 2, alpha polypeptide // ---	PIK3C2A	<0.05	2.05265
15030673	XM_001490934 // ABHD16A // abhydrolase domain containing 16A // --- // 100058736 /// EN	ABHD16A	<0.05	2.05257
14981991	ENSECAT00000017014 // LOC100630177 // t-cell immunoglobulin and mucin domain-containing	LOC100630177	<0.05	2.05234
14989613	XM_001496741 // LOC100052589 // translation initiation factor IF-2, mitochondrial-like	LOC100052589	<0.05	2.05203
15102337	ENSECAT00000024651 // LOC100064131 // ATP synthase lipid-binding protein, mitochondrial	LOC100064131	<0.05	2.05129
14965520	ENSECAT00000007802 // NBR1 // neighbor of BRCA1 gene 1 // --- // 100051928 /// XM_00336	NBR1	<0.05	2.05054
14990246	XM_001917878 // LOC100147678 // protein MEMO1-like // --- // 100147678 /// ENSECAT00000	LOC100147678	<0.05	2.04944
15041424	ENSECAT00000015870 // ZCCHC6 // zinc finger, CCHC domain containing 6 // --- // 1000616	ZCCHC6	<0.05	2.04862
14960190	XM_001495646 // ACLY // ATP citrate lyase // --- // 100053195 /// ENSECAT00000010787 //	ACLY	<0.05	2.04847
14928161	---		<0.05	2.04836
14931977	---		<0.05	2.0477
15018831	ENSECAT00000013774 // NOL9 // nucleolar protein 9 // --- // ---	NOL9	<0.05	2.04732
14976858	ENSECAT00000027038 // RSL1D1 // ribosomal L1 domain containing 1 // --- // ---	RSL1D1	<0.05	2.04701
15070101	ENSECAT00000022571 // LOC100050994 // protein PIEZO1-like // --- // 100050994	LOC100050994	<0.05	2.04699
14965997			<0.05	2.04673
15000138	ENSECAT00000014540 // LOC100147234 // testis-expressed sequence 264 protein-like // ---	LOC100147234	<0.05	2.04606
15109812	XM_001502605 // PCSK7 // proprotein convertase subtilisin/kexin type 7 // --- // 100062	PCSK7	<0.05	2.04596
14952077			<0.05	2.04586
14950479	ENSECAT00000018056 // LOC100070361 // zinc finger protein 283-like // --- // 100070361	LOC100070361	<0.05	2.04534
15108855	ENSECAT00000000324 // LOC100065544 // general transcription factor IIF subunit 1-like /	LOC100065544	<0.05	2.04509
14975363	XM_001492538 // LOC100060194 // AP-1 complex subunit sigma-1A-like // --- // 100060194	LOC100060194	<0.05	2.04433
14976098	ENSECAT00000007759 // ATXN2L // ataxin 2-like // --- // 100066373 /// XM_001496666 // A	ATXN2L	<0.05	2.04304
14968881	ENSECAT00000027158 // LOC100073084 // protein SCO1 homolog, mitochondrial-like // --- /	LOC100073084	<0.05	2.04299
15074336	ENSECAT00000019433 // RAB3GAP2 // RAB3 GTPase activating protein subunit 2 (non-catalyt	RAB3GAP2	<0.05	2.04291
15108809	XM_001495919 // LOC100065287 // ran-binding protein 3-like // --- // 100065287 /// ENSE	LOC100065287	<0.05	2.04165
15060677	ENSECAT00000022563 // APLP2 // amyloid beta (A4) precursor-like protein 2 // --- // 100	APLP2	<0.05	2.04144
14976661	---		<0.05	2.04123
14977869	XM_001494582 // ARPC1A // actin related protein 2/3 complex, subunit 1A, 41kDa // --- /	ARPC1A	<0.05	2.041
15035449	XM_001494494 // LOC100063168 // NADH dehydrogenase [ubiquinone] iron-sulfur protein 4,	LOC100063168	<0.05	2.04044
15048112	XM_001489195 // ATG2B // autophagy related 2B // --- // 100054652 /// ENSECAT0000000401	ATG2B	<0.05	2.04038
15069905	XM_003364642 // MBTPS1 // membrane-bound transcription factor peptidase, site 1 // ---	MBTPS1	<0.05	2.0402
14996061	XM_001493474 // BAP1 // BRCA1 associated protein-1 (ubiquitin carboxy-terminal hydrolas	BAP1	<0.05	2.03987
14935414	ENSECAT00000017306 // FAM89A // family with sequence similarity 89, member A // --- //	FAM89A	<0.05	2.03962
15076301	ENSECAT00000024588 // LOC100065954 // uncharacterized LOC100065954 // --- // 100065954	LOC100065954	<0.05	2.03934
14971511	ENSECAT00000008593 // ALDH3B1 // aldehyde dehydrogenase 3 family, member B1 // --- // 1	ALDH3B1	<0.05	2.03905
15101022	XM_001502728 // LOC100064589 // cell cycle regulator Mat89Bb homolog // --- // 10006458	LOC100064589	<0.05	2.0389
15124487	XM_001497735 // FBXO32 // F-box protein 32 // --- // 100057506 /// ENSECAT00000017032 /	FBXO32	<0.05	2.03854
14966655	XM_001503751 // LOC100057442 // tubulin delta chain-like // --- // 100057442 /// ENSECA	LOC100057442	<0.05	2.03828
15031242	ENSECAT00000000392 // LOC100053157 // uncharacterized protein C6orf106 homolog // --- /	LOC100053157	<0.05	2.03813
15130093	ENSECAT00000009431 // LOC100054669 // HIV Tat-specific factor 1 homolog // --- // 10005	LOC100054669	<0.05	2.03807
15089991	ENSECAT00000022989 // TPR // translocated promoter region, nuclear basket protein // --	TPR	<0.05	2.03779
15007112	XM_001916894 // PPIG // peptidylprolyl isomerase G (cyclophilin G) // --- // 100052595	PPIG	<0.05	2.03748
15116254	XM_001916080 // ELP2 // elongation protein 2 homolog (S. cerevisiae) // --- // 10005293	ELP2	<0.05	2.0373
15082609	ENSECAT00000009237 // POT1 // protection of telomeres 1 homolog (S. pombe) // --- // 10	POT1	<0.05	2.0367
15039160	XM_001489907 // LOC100061304 // n-alpha-acetyltransferase 20, NatB catalytic subunit-li	LOC100061304	<0.05	2.03626
14926381	---		<0.05	2.03624
15134270	---		<0.05	2.03597
14930271	---		<0.05	2.03586
15004218	ENSECAT00000020697 // CUL4A // cullin 4A // --- // 100033930 /// ENSECAT00000020701 //	CUL4A	<0.05	2.03566
14928127	---		<0.05	2.03471
15025290	ENSECAT00000011464 // KLHL2 // kelch-like 2, Mayven (Drosophila) // --- // 100068150 //	KLHL2	<0.05	2.03433
14954131	XM_001499052 // LOC100068129 // SERTA domain-containing protein 3-like // --- // 100068	LOC100068129	<0.05	2.03379
14965410	XM_001489741 // GRN // granulin // --- // 100051035 /// ENSECAT00000019589 // GRN // gr	GRN	<0.05	2.03367
14986793	ENSECAT00000019464 // LOC100073220 // RGM domain family member B-like // --- // 1000732	LOC100073220	<0.05	2.03356
14953947	ENSECAT00000013350 // HNRNPL // heterogeneous nuclear ribonucleoprotein L // --- // 100	HNRNPL	<0.05	2.03326
14969442	XM_001503314 // PDHX // pyruvate dehydrogenase complex, component X // --- // 100059260	PDHX	<0.05	2.03315
14934905	XM_001503972 // LOC100064276 // voltage-dependent anion-selective channel protein 2-lik	LOC100064276	<0.05	2.03284
15089102	ENSECAT00000006690 // ELK4 // ELK4, ETS-domain protein (SRF accessory protein 1) // ---	ELK4	<0.05	2.03232
14995794	ENSECAT00000010119 // FAM208A // family with sequence similarity 208, member A // --- /	FAM208A	<0.05	2.03137
15006148	---		<0.05	2.03132
15072116	XM_001499564 // ZCCHC4 // zinc finger, CCHC domain containing 4 // --- // 100067435 ///	ZCCHC4	<0.05	2.03079
15000948			<0.05	2.03059
15063432	NM_001243145 // VIM // vimentin // --- // 100056088 /// ENSECAT00000006522 // VIM // vi	VIM	<0.05	2.03047
14952827	ENSECAT00000014894 // LOC100072366 // general transcription factor 3C polypeptide 6-lik	LOC100072366	<0.05	2.03006
15053039	XM_001499227 // DDX31 // DEAD (Asp-Glu-Ala-Asp) box polypeptide 31 // --- // 100069463	DDX31	<0.05	2.0296
14965992	---		<0.05	2.02889
14949997	ENSECAT00000016592 // SAMD4B // sterile alpha motif domain containing 4B // --- // 1000	SAMD4B	<0.05	2.02737
14927637	---		<0.05	2.02643
14952430	ENSECAT00000007519 // SLC35A1 // solute carrier family 35 (CMP-sialic acid transporter)	SLC35A1	<0.05	2.0263

14954978			<0.05	2.02617
14945613	ENSECAT00000008558 // LOC100059897 // mortality factor 4-like protein 1-like // --- //	LOC100059897	<0.05	2.02613
14942925	XM_001503220 // MINPP1 // multiple inositol-polyphosphate phosphatase 1 // --- // 10006	MINPP1	<0.05	2.02574
15119156	XM_001493348 // CHFR // checkpoint with forkhead and ring finger domains, E3 ubiquitin	CHFR	<0.05	2.02565
15041278	ENSECAT00000007228 // LOC100146261 // uridine-cytidine kinase-like 1-like // --- // 100	LOC100146261	<0.05	2.02558
15035633	ENSECAT00000015947 // LOC100065773 // f-box only protein 4-like // --- // 100065773 ///	LOC100065773	<0.05	2.02547
14969698	ENSECAT00000020744 // DGKZ // diacylglycerol kinase, zeta // --- // --- /// ENSECAT0000	DGKZ	<0.05	2.02475
14966668	ENSECAT00000018593 // RNFT1 // ring finger protein, transmembrane 1 // --- // 100072218	RNFT1	<0.05	2.0245
15087938	XM_001488612 // DBT // dihydrolipoamide branched chain transacylase E2 // --- // 100050	DBT	<0.05	2.02431
15089435	XM_001491829 // KIFAP3 // kinesin-associated protein 3 // --- // 100059088 /// ENSECAT0	KIFAP3	<0.05	2.02398
15074470	XM_001492101 // LOC100059547 // ubiquitin carboxyl-terminal hydrolase isozyme L5-like /	LOC100059547	<0.05	2.02398
15083882	XM_001489035 // MDM4 // Mdm4 p53 binding protein homolog (mouse) // --- // 100050113 //	MDM4	<0.05	2.02314
14992869	XM_001494265 // LOC100051612 // malate dehydrogenase, cytoplasmic-like // --- // 100051	LOC100051612	<0.05	2.02305
14930657	---		<0.05	2.02297
15072698	XM_001489181 // LOC100051309 // e3 ubiquitin ligase RNF4-like // --- // 100051309 /// E	LOC100051309	<0.05	2.02127
15020704	ENSECAT00000023598 // ELF2 // E74-like factor 2 (ets domain transcription factor) // --	ELF2	<0.05	2.02122
14979117	XM_001500749 // MYST1 // K(lysine) acetyltransferase 8 // --- // 100064910 /// ENSECAT0	MYST1	<0.05	2.02049
15111587	ENSECAT00000016335 // LOC100050619 // uncharacterized protein C11orf73 homolog // --- /	LOC100050619	<0.05	2.02037
15086875	ENSECAT00000019618 // LASS2 // ceramide synthase 2 // --- // 100055037 /// XM_001491068	LASS2	<0.05	2.02014
14971156	ENSECAT00000026135 // LOC100052051 // barrier-to-autointegration factor-like // --- //	LOC100052051	<0.05	2.02011
15012649	XM_001501592 // LOC100060529 // zinc finger protein 148-like // --- // 100060529 /// EN	LOC100060529	<0.05	2.02007
15087508	ENSECAT00000008155 // RSBN1 // round spermatid basic protein 1 // --- // ---	RSBN1	<0.05	2.0199
15101516	ENSECAT00000021486 // RPAP3 // RNA polymerase II associated protein 3 // --- // --- ///	RPAP3	<0.05	2.01973
14991284	XM_001501043 // LOC100060195 // coiled-coil domain-containing protein 138-like // --- /	LOC100060195	<0.05	2.01872
15059709	NM_001256974 // ZBED4 // zinc finger, BED-type containing 4 // --- // 100054656 /// ENS	ZBED4	<0.05	2.01737
15112130	ENSECAT00000008532 // TRIM68 // tripartite motif containing 68 // --- // 100053519 ///	TRIM68	<0.05	2.01701
15071461	---		<0.05	2.01646
15100225	ENSECAT00000021556 // FOXJ2 // forkhead box J2 // --- // 100053321 /// XM_001498727 //	FOXJ2	<0.05	2.01633
15044471	ENSECAT00000009307 // LOC100059241 // dapper homolog 1-like // --- // 100059241 /// XR_	LOC100059241	<0.05	2.01631
15002462	NM_001171173 // MPHOSPH8 // M-phase phosphoprotein 8 // --- // 100053303 /// ENSECAT000	MPHOSPH8	<0.05	2.0159
14926007	---		<0.05	2.01581
15099989	ENSECAT00000013305 // NOP2 // NOP2 nucleolar protein homolog (yeast) // --- // 10005220	NOP2	<0.05	2.01539
14950147	XM_003362315 // LOC100630098 // SH3KBP1-binding protein 1-like // --- // 100630098 ///	LOC100630098	<0.05	2.01527
15048945	ENSECAT00000019819 // RNF20 // ring finger protein 20, E3 ubiquitin protein ligase // -	RNF20	<0.05	2.0151
14973945	ENSECAT00000024543 // LOC100058729 // RNA-binding protein 4B-like // --- // 100058729 /	LOC100058729	<0.05	2.01477
15090642	ENSECAT00000020352 // LOC100058382 // astrocytic phosphoprotein PEA-15-like // --- // 1	LOC100058382	<0.05	2.01437
14951141	XM_001491984 // LOC100051978 // 40S ribosomal protein S11-like // --- // 100051978 ///	LOC100051978	<0.05	2.01337
14984247	ENSECAT00000006172 // LOC100061535 // histone H3.1-like // --- // 100061535	LOC100061535	<0.05	2.01306
14982616	XM_001504160 // LOC100061811 // NADH dehydrogenase [ubiquinone] 1 alpha subcomplex subu	LOC100061811	<0.05	2.01264
15003888	XM_001492116 // TM9SF2 // transmembrane 9 superfamily member 2 // --- // 100061300 ///	TM9SF2	<0.05	2.01234
15005551	NM_001163827 // RBM26 // RNA binding motif protein 26 // --- // 100050021 /// ENSECAT00	RBM26	<0.05	2.01213
15001371	XM_001491223 // CNOT10 // CCR4-NOT transcription complex, subunit 10 // --- // 10005081	CNOT10	<0.05	2.01207
15116777	XM_001914881 // ZCCHC2 // zinc finger, CCHC domain containing 2 // --- // 100056858 ///	ZCCHC2	<0.05	2.01203
14940306	XM_001489179 // LOC100051017 // leukotriene B4 receptor 1-like // --- // 100051017 ///	LOC100051017	<0.05	2.01162
15076717	ENSECAT00000015659 // DUS4L // dihydrouridine synthase 4-like (S. cerevisiae) // --- //	DUS4L	<0.05	2.01111
14995858	XM_001915031 // LOC100059386 // actin-related protein 8-like // --- // 100059386 /// EN	LOC100059386	<0.05	2.01107
15027919	XM_001493006 // LOC100060885 // proteasome subunit beta type-9-like // --- // 100060885	LOC100060885	<0.05	2.01103
14979264	ENSECAT00000024053 // LOC100063732 // probable fibrosin-1-like // --- // 100063732	LOC100063732	<0.05	2.0109
14962444	XM_001504725 // LOC100060628 // transmembrane protein 93-like // --- // 100060628 /// E	LOC100060628	<0.05	2.01081
15038404	XM_001501119 // DDX27 // DEAD (Asp-Glu-Ala-Asp) box polypeptide 27 // --- // 100071375	DDX27	<0.05	2.0107
14951589	ENSECAT00000007504 // ZNF331 // zinc finger protein 331 // --- // ---	ZNF331	<0.05	2.01054
14955263	XM_001493510 // LOC100052342 // inactive serine/threonine-protein kinase VRK3-like // -	LOC100052342	<0.05	2.01041
14980087	ENSECAT00000009379 // LOC100050421 // nuclear distribution protein nudE homolog 1-like	LOC100050421	<0.05	2.01007
15114871	ENSECAT00000012727 // MLXIP // MLX interacting protein // --- // 100060001	MLXIP	<0.05	2.0097
14944250	ENSECAT00000002899 // MARCH8 // membrane-associated ring finger (C3HC4) 8, E3 ubiquitin	Mar-08	<0.05	2.00909
15000535	XM_001495506 // PTPN23 // protein tyrosine phosphatase, non-receptor type 23 // --- //	PTPN23	<0.05	2.009
15015671	---		<0.05	2.00863
15110653	ENSECAT00000020391 // TECR // trans-2,3-enoyl-CoA reductase // --- // 100065024 /// XM_	TECR	<0.05	2.00832
14929685	---		<0.05	2.00806
15011951	XM_001915856 // LOC100067030 // ATP-binding cassette sub-family F member 3-like // ---	LOC100067030	<0.05	2.00795
14932121	---		<0.05	2.00717
15029183	NM_001160296 // FBXO9 // F-box protein 9 // --- // 100069351 /// ENSECAT00000007724 //	FBXO9	<0.05	2.0068
15022297	XM_001503360 // CAP1 // CAP, adenylate cyclase-associated protein 1 (yeast) // --- // 1	CAP1	<0.05	2.00657
15046932	XM_001498781 // PPP2R5E // protein phosphatase 2, regulatory subunit B', epsilon isofor	PPP2R5E	<0.05	2.00632
15065293	XM_001500840 // COG4 // component of oligomeric golgi complex 4 // --- // 100054801 ///	COG4	<0.05	2.0056
15129390	XM_001490668 // ATG4A // autophagy related 4A, cysteine peptidase // --- // 100060165 /	ATG4A	<0.05	2.00494
14967435	XM_001504186 // PHF12 // PHD finger protein 12 // --- // 100059567 /// ENSECAT000000176	PHF12	<0.05	2.00485
15040039	ENSECAT00000012218 // LOC100069487 // ubiquinol-cytochrome c reductase complex chaperon	LOC100069487	<0.05	2.00483
14928517	---		<0.05	2.00442
15126679			<0.05	2.00383
15038557	XM_001490026 // LOC100050441 // mRNA export factor-like // --- // 100050441 /// ENSECAT	LOC100050441	<0.05	2.0038
15108335	XM_001502745 // EIF3M // eukaryotic translation initiation factor 3, subunit M // --- /	EIF3M	<0.05	2.00352
15121109	XM_001494559 // ARFGEF1 // ADP-ribosylation factor guanine nucleotide-exchange factor 1	ARFGEF1	<0.05	2.00317
15026574	XM_001491137 // RIPK1 // receptor (TNFRSF)-interacting serine-threonine kinase 1 // ---	RIPK1	<0.05	2.0029
14959697	XM_003362540 // EFTUD2 // elongation factor Tu GTP binding domain containing 2 // --- /	EFTUD2	<0.05	2.00255
15095452	XM_001490880 // LOC100057576 // tubby-related protein 3-like // --- // 100057576 /// EN	LOC100057576	<0.05	2.00184
15033756	ENSECAT00000021269 // LMBRD2 // LMBR1 domain containing 2 // --- // 100067733 /// XM_00	LMBRD2	<0.05	2.00172
15056734	XM_001488787 // LOC100053704 // protein farnesyltransferase/geranylgeranyltransferase t	LOC100053704	<0.05	2.00157
15086553	XM_001493858 // DENND4B // DENN/MADD domain containing 4B // --- // 100062197 /// ENSEC	DENND4B	<0.05	2.00112
15133114	ENSECAT00000025736 // CUL4B // cullin 4B // --- // 100033938	CUL4B	<0.05	2.00032
15001836	XM_001496343 // MSL2 // male-specific lethal 2 homolog (Drosophila) // --- // 100065902	MSL2	<0.05	2.00015
15099296	ENSECAT00000022876 // LOC100066356 // collagen alpha-3(VI) chain-like // --- // 1000663	LOC100066356	<0.05	-2.00004
15035863	ENSECAT00000011679 // LOC100053996 // tetratricopeptide repeat protein 23-like // --- /	LOC100053996	<0.05	-2.00037
15044843	---		<0.05	-2.00046
14952505	XM_001915432 // GJA10 // gap junction protein, alpha 10, 62kDa // --- // 100065787 ///	GJA10	<0.05	-2.00101
14961328	XM_001503473 // LOC100056800 // olfactory receptor 4D1-like // --- // 100056800 /// ENS	LOC100056800	<0.05	-2.00181
15007743	---		<0.05	-2.00271
14981165	---		<0.05	-2.00291
14962731	XM_001502998 // ALOX12 // arachidonate 12-lipoxygenase // --- // 100072916 /// ENSECAT0	ALOX12	<0.05	-2.00349
15102460	XM_001489428 // LOC100055082 // olfactory receptor 6C2-like // --- // 100055082 /// ENS	LOC100055082	<0.05	-2.00354
15083454	---		<0.05	-2.00495
15027572	ENSECAT00000016751 // DPCR1 // diffuse panbronchiolitis critical region 1 // --- // ---	DPCR1	<0.05	-2.00507
14992726	---		<0.05	-2.00603
14979262	---		<0.05	-2.00627
15048967	---		<0.05	-2.00635
14986251	ENSECAT00000026096 // LOC100063524 // cortexin-3-like // --- // 100063524 /// XM_001504	LOC100063524	<0.05	-2.00636
14953037	ENSECAT00000024030 // LOC100067280 // fatty acid-binding protein, brain-like // --- //	LOC100067280	<0.05	-2.00659
15019432	ENSECAT00000023800 // BMP1 // bone morphogenetic protein 1 // --- // 100056914 /// ENSE	BMP1	<0.05	-2.00767
14948602	---		<0.05	-2.00835
14931029	---		<0.05	-2.01186
14925423	---		<0.05	-2.01246
14929789	---		<0.05	-2.01291
15037129	ENSECAT00000000883 // DEFB117 // defensin, beta 117 // --- // ---	DEFB117	<0.05	-2.01293
15101432	XM_001489424 // LOC100055071 // homeobox protein DBX2-like // --- // 100055071 /// ENSE	LOC100055071	<0.05	-2.0132
15077207	---		<0.05	-2.01435
14981535	XM_001498360 // DBN1 // drebrin 1 // --- // 100068529 /// ENSECAT00000011230 // DBN1 //	DBN1	<0.05	-2.01459

14966267 ENSECAT00000020087 // COL1A1 // collagen, type I, alpha 1 // --- // --- /// ENSECAT0000	COL1A1	<0.05	-2.01609
15128740 ---		<0.05	-2.017
15024391 NR_032793 // MIR1302-1 // microRNA mir-1302-1 // --- // 100315099	MIR1302-1	<0.05	-2.01736
15092259 ---		<0.05	-2.01749
15027584 ---		<0.05	-2.01812
15049011 XM_001493810 // LOC100062119 // putative olfactory receptor ENSP00000348552-like // ---	LOC100062119	<0.05	-2.01822
14929167 ---		<0.05	-2.01944
15020197 ---		<0.05	-2.01944
15037024 XM_001497665 // LOC100053108 // proenkephalin-B-like // --- // 100053108 /// ENSECAT000	LOC100053108	<0.05	-2.02004
15012098 XM_001497358 // LOC100067260 // transmembrane epididymal protein 1-like // --- // 10006	LOC100067260	<0.05	-2.02038
15046040 ---		<0.05	-2.0214
14954392 ENSECAT00000007789 // LOC100629397 // seminal plasma protein A3-like // --- // 10062939	LOC100629397	<0.05	-2.02168
15136736 ---		<0.05	-2.02221
15087836 ---		<0.05	-2.02291
15136954 ---		<0.05	-2.02291
14934007 XM_001500917 // LOC100071214 // phospholipase A2-like // --- // 100071214 /// ENSECAT00	LOC100071214	<0.05	-2.02313
15106405 XM_001494305 // LOC100062882 // olfactory receptor 7G3-like // --- // 100062882 /// ENS	LOC100062882	<0.05	-2.0232
15136712 ---		<0.05	-2.02339
15109554 ---		<0.05	-2.02426
15124978 ---		<0.05	-2.02604
14931789 ---		<0.05	-2.02664
14948474 ---		<0.05	-2.02697
14925623 ---		<0.05	-2.03004
14982360 ---		<0.05	-2.03045
15107652 ---		<0.05	-2.03049
14985875 XM_001502601 // WNT8A // wingless-type MMTV integration site family, member 8A // --- /	WNT8A	<0.05	-2.0305
15048016 NM_001114533 // SPI2 // alpha-1-antitrypsin // --- // 100065158 /// AB455571 // SPI2 //	SPI2	<0.05	-2.03077
15135866 ---		<0.05	-2.03077
15058240 XM_001494836 // ELK3 // ELK3, ETS-domain protein (SRF accessory protein 2) // --- // 10	ELK3	<0.05	-2.03209
14929803 ---		<0.05	-2.03261
14927197 ---		<0.05	-2.03277
14950850 XM_001503145 // LOC100065997 // c5a anaphylatoxin chemotactic receptor-like // --- // 1	LOC100065997	<0.05	-2.03301
15012958 ENSECAT00000019225 // LOC100070833 // follistatin-related protein 1-like // --- // 1000	LOC100070833	<0.05	-2.03313
15070376 ---		<0.05	-2.03325
15136832 ---		<0.05	-2.03325
14927235 ---		<0.05	-2.03369
14929105 ---		<0.05	-2.03386
14957606 XM_001504334 // LOC100073120 // trace amine-associated receptor 8a-like // --- // 10007	LOC100073120	<0.05	-2.03538
14927569 ---		<0.05	-2.03647
14927231 ---		<0.05	-2.03728
14926195 ---		<0.05	-2.03736
15070990 ENSECAT00000026028 // FAM47E // family with sequence similarity 47, member E // --- //	FAM47E	<0.05	-2.038
15090301 NR_032842 // MIR205 // microRNA mir-205 // --- // 100314810	MIR205	<0.05	-2.03837
14986407 ---		<0.05	-2.03909
15063751 ---		<0.05	-2.03925
14926703 ---		<0.05	-2.03935
15096771 ENSECAT00000007241 // LOC100051004 // uncharacterized protein C12orf68 homolog // --- /	LOC100051004	<0.05	-2.03945
14953790 ---		<0.05	-2.03969
15071098 ENSECAT00000016212 // IL8 // interleukin 8 // --- // 100037400 /// NM_001083951 // IL8	IL8	<0.05	-2.03993
15107855 ENSECAT00000009242 // TEAD1 // TEA domain family member 1 (SV40 transcriptional enhance	TEAD1	<0.05	-2.04003
15132171 ---		<0.05	-2.04045
14962413 ---		<0.05	-2.04127
14988276 XM_001493078 // LOC100051611 // dual specificity protein phosphatase 2-like // --- // 1	LOC100051611	<0.05	-2.04178
15100237 ENSECAT00000013235 // LOC100061006 // homeobox protein NANOG-like // --- // 100061006	LOC100061006	<0.05	-2.04249
15080674 ---		<0.05	-2.04374
15107732 ---		<0.05	-2.0451
15042379 ---		<0.05	-2.04612
15107282 ---		<0.05	-2.04639
14979496 NM_001081774 // IL4R // interleukin 4 receptor // --- // 791252 /// ENSECAT00000023156	IL4R	<0.05	-2.04688
15136942 ---		<0.05	-2.04907
14928855 ---		<0.05	-2.05023
15027232 ---		<0.05	-2.05071
14952309 ---		<0.05	-2.05264
14936247 ---		<0.05	-2.05323
14928941 ---		<0.05	-2.05398
15110167 XM_001501622 // LOC100071772 // olfactory receptor 8D4-like // --- // 100071772 /// ENS	LOC100071772	<0.05	-2.05452
15051287 XM_001504497 // LOC100055542 // tropomyosin beta chain-like // --- // 100055542 /// ENS	LOC100055542	<0.05	-2.05876
15136578 ---		<0.05	-2.05901
14930363 ---		<0.05	-2.05955
15059967 ---		<0.05	-2.05997
14939564 XM_001502491 // LOC100072530 // olfactory receptor 11H7-like // --- // 100072530 /// EN	LOC100072530	<0.05	-2.0602
12910873		<0.05	-2.06223
12910881		<0.05	-2.06223
15126585		<0.05	-2.06236
14926295 ---		<0.05	-2.06242
14983353 ---		<0.05	-2.06253
15099149 XM_001495017 // LOC100063969 // G-protein coupled receptor 55-like // --- // 100063969	LOC100063969	<0.05	-2.06428
14956770 ENSECAT00000008095 // LOC100066146 // melanin-concentrating hormone receptor 2-like //	LOC100066146	<0.05	-2.06431
15108327 XM_001502680 // LOC100072670 // reticulocalbin-1-like // --- // 100072670 /// ENSECAT00	LOC100072670	<0.05	-2.06432
15124500 ENSECAT00000022962 // LOC100067174 // annexin A13-like // --- // 100067174	LOC100067174	<0.05	-2.06511
14931465 ---		<0.05	-2.06537
14986506 ENSECAT00000008997 // LOC100064195 // uncharacterized protein C11orf46 homolog // --- /	LOC100064195	<0.05	-2.06754
15120981 ---		<0.05	-2.06834
15111265 XM_001493466 // LOC100061569 // olfactory receptor 7D4-like // --- // 100061569 /// ENS	LOC100061569	<0.05	-2.06904
14940063 ENSECAT00000009008 // MMP14 // matrix metallopeptidase 14 (membrane-inserted) // --- //	MMP14	<0.05	-2.06931
14928533 ---		<0.05	-2.07024
14951864 XM_003362300 // LOC100629902 // vomeronasal type-1 receptor 1-like // --- // 100629902	LOC100629902	<0.05	-2.07074
15007033 ---		<0.05	-2.07203
15026283 ---		<0.05	-2.07357
15075229 ---		<0.05	-2.07371
14953625 ENSECAT00000027119 // SBSN // suprabasin // --- // ---	SBSN	<0.05	-2.07443
15078650 ---		<0.05	-2.07476
14961281 ---		<0.05	-2.07484
15091037 ENSECAT00000008851 // SEMA4A // sema domain, immunoglobulin domain (Ig), transmembrane	SEMA4A	<0.05	-2.07733
15006416 ENSECAT00000005374 // C1QL2 // complement component 1, q subcomponent-like 2 // --- //	C1QL2	<0.05	-2.07738
15112421 XM_001499853 // LOC100070187 // olfactory receptor 6-like // --- // 100070187 /// ENSEC	LOC100070187	<0.05	-2.07744
14926809 ---		<0.05	-2.07833
15056320 ENSECAT00000014155 // FAT1 // FAT tumor suppressor homolog 1 (Drosophila) // --- // 100	FAT1	<0.05	-2.07834
14927475 ---		<0.05	-2.07971
14928115 ---		<0.05	-2.07971
14943917 ---		<0.05	-2.08049
15137174 ---		<0.05	-2.08159
15135264 ---		<0.05	-2.08162
14984969 ---		<0.05	-2.08235
15057831 ---		<0.05	-2.08428
14928553 ---		<0.05	-2.08457

15020465 ---		<0.05	-2.08487
14983337 ---		<0.05	-2.08489
14974963 ---		<0.05	-2.08636
15021046 ---		<0.05	-2.08733
14967613 XM_001502177 // LOC100072280 // diazepam-binding inhibitor-like 5-like // --- // 100072	LOC100072280	<0.05	-2.08759
14926061 ---		<0.05	-2.0881
15072947 ENSECAT00000016295 // MIR1255B // microRNA mir-1255b // --- // 100314985	MIR1255B	<0.05	-2.08815
15057592 ---		<0.05	-2.08822
14927957 ---		<0.05	-2.091
15110165 XM_001501618 // LOC100071766 // olfactory receptor 6M1-like // --- // 100071766 /// ENS	LOC100071766	<0.05	-2.09196
15123206 ---		<0.05	-2.09197
14941799 ---		<0.05	-2.09245
15000659 XM_001496089 // CCR9 // chemokine (C-C motif) receptor 9 // --- // 100065535 /// ENSECA	CCR9	<0.05	-2.09308
15026146 XM_001916288 // USP53 // ubiquitin specific peptidase 53 // --- // 100072411 /// ENSECA	USP53	<0.05	-2.09336
15113452 ---		<0.05	-2.09389
14969918 XM_001489062 // LOC100054355 // olfactory receptor 1030-like // --- // 100054355 /// EN	LOC100054355	<0.05	-2.09413
15093714 ENSECAT00000008704 // ANGPTL3 // angiopoietin-like 3 // --- // 100054159 /// XM_0015010	ANGPTL3	<0.05	-2.09414
14926153 ---		<0.05	-2.09511
15002995 ENSECAT00000004403 // LOC100062457 // uncharacterized protein C13orf36-like // --- // 1	LOC100062457	<0.05	-2.09753
14931599 ---		<0.05	-2.09805
15058329 ---		<0.05	-2.0988
15058330		<0.05	-2.0988
15071992 ---		<0.05	-2.09969
15016922 XM_001499027 // CSF3R // colony stimulating factor 3 receptor (granulocyte) // --- // 1	CSF3R	<0.05	-2.10044
15034562 XM_001914909 // LOC100070775 // olfactory receptor 10H4-like // --- // 100070775 /// EN	LOC100070775	<0.05	-2.10058
15046170 NR_033032 // MIR487A // microRNA mir-487a // --- // 100314913	MIR487A	<0.05	-2.10111
15033035 ---		<0.05	-2.10113
14951763 ---		<0.05	-2.1015
15093251 ---		<0.05	-2.102
14931283 ---		<0.05	-2.10209
15104828 XM_001503043 // LOC100063180 // uroplakin-2-like // --- // 100063180 /// ENSECAT0000001	LOC100063180	<0.05	-2.10474
15085955 XM_001500763 // LOC100064714 // ETS translocation variant 3-like // --- // 100064714 //	LOC100064714	<0.05	-2.10514
15062884 XM_001496461 // LOC100066044 // olfactory receptor 2T33-like // --- // 100066044 /// EN	LOC100066044	<0.05	-2.10659
15109386 NM_001081804 // MMP13 // matrix metallopeptidase 13 (collagenase 3) // --- // 100009711	MMP13	<0.05	-2.10697
15107293 ---		<0.05	-2.10715
15023530 ---		<0.05	-2.1072
14936389 XM_001490555 // ALDH1A3 // aldehyde dehydrogenase 1 family, member A3 // --- // 1000570	ALDH1A3	<0.05	-2.10783
15137322 ---		<0.05	-2.10886
14943309 XM_001503727 // LOC100063458 // leucine-rich repeat-containing protein 20-like // --- /	LOC100063458	<0.05	-2.10901
15137528 ---		<0.05	-2.10998
14991679 ENSECAT00000011743 // LOC100061333 // cyclic nucleotide-gated cation channel alpha-3-li	LOC100061333	<0.05	-2.11037
14932349 ---		<0.05	-2.11101
14949069 XM_001497325 // LOC100067227 // nidogen-2-like // --- // 100067227 /// ENSECAT000000166	LOC100067227	<0.05	-2.11135
14989035 ---		<0.05	-2.1114
15137784 ---		<0.05	-2.1114
14934452 ---		<0.05	-2.1117
15134892 ---		<0.05	-2.11382
15131323 ---		<0.05	-2.11445
15099403 XM_001497223 // LOC100067104 // olfactory receptor 6B2-like // --- // 100067104 /// ENS	LOC100067104	<0.05	-2.11463
14955834 ENSECAT00000015199 // IL11 // interleukin 11 // --- // ---	IL11	<0.05	-2.11467
14996102 NM_001081790 // TLR9 // toll-like receptor 9 // --- // 100009693 /// ENSECAT00000014884	TLR9	<0.05	-2.11484
15080372 ---		<0.05	-2.11488
15129015 ---		<0.05	-2.11509
15019043 NR_032807 // MIR551A // microRNA mir-551a // --- // 100315040	MIR551A	<0.05	-2.11509
14972723 ---		<0.05	-2.11532
14930735 ---		<0.05	-2.11659
15084466 ---		<0.05	-2.11726
14952181 ---		<0.05	-2.11778
14987334 XM_001504666 // LOC100073277 // proteinase-activated receptor 2-like // --- // 10007327	LOC100073277	<0.05	-2.11827
14950253 ---		<0.05	-2.11866
14927759 ---		<0.05	-2.11876
15078697		<0.05	-2.11901
14972623 ---		<0.05	-2.1191
14928883 ---		<0.05	-2.11948
14972641 ---		<0.05	-2.11975
14931045 ---		<0.05	-2.11997
14926241 ---		<0.05	-2.12191
14982656 ENSECAT00000010964 // LOC100629956 // uncharacterized LOC100629956 // --- // 100629956	LOC100629956	<0.05	-2.12253
14946843 ---		<0.05	-2.12373
15124206 ---		<0.05	-2.12403
15020620 ---		<0.05	-2.12575
15049266 ENSECAT00000021954 // ZNF618 // zinc finger protein 618 // --- // 100051828	ZNF618	<0.05	-2.12695
15132547 ---		<0.05	-2.12793
15014995 ---		<0.05	-2.12835
15088053 ENSECAT00000010311 // LOC100051002 // rho GTPase-activating protein 29-like // --- // 1	LOC100051002	<0.05	-2.12906
15121319 ---		<0.05	-2.13099
14939007 ENSECAT00000018492 // LOC100071046 // EH domain-containing protein 4-like // --- // 100	LOC100071046	<0.05	-2.13109
15058862 ENSECAT00000019359 // TIMP3 // TIMP metallopeptidase inhibitor 3 // --- // 100033947 //	TIMP3	<0.05	-2.13144
14974966 XM_001489025 // PRKAR1B // protein kinase, cAMP-dependent, regulatory, type I, beta //	PRKAR1B	<0.05	-2.13169
14939795 ENSECAT00000011369 // LOC100629138 // uncharacterized LOC100629138 // --- // 100629138	LOC100629138	<0.05	-2.13226
14965215 ---		<0.05	-2.13356
14925679 ---		<0.05	-2.13545
14925909 ---		<0.05	-2.13545
15112287 XM_001499184 // LOC100069414 // olfactory receptor 52H1-like // --- // 100069414 /// EN	LOC100069414	<0.05	-2.13679
15046762 ---		<0.05	-2.13748
15043508 ---		<0.05	-2.13753
15077149 ---		<0.05	-2.13911
15039029 ---		<0.05	-2.14082
15136380 ---		<0.05	-2.14152
15025088 ---		<0.05	-2.14173
15112823 ---		<0.05	-2.14217
14967427 NR_032913 // MIR451 // microRNA mir-451 // --- // 100315060	MIR451	<0.05	-2.14245
15020805 ---		<0.05	-2.14501
14929143 ---		<0.05	-2.14585
14931929 ---		<0.05	-2.1463
15016202 ---		<0.05	-2.14656
15089239 ENSECAT00000000241 // FCAMR // Fc receptor, IgA, IgM, high affinity // --- // ---	FCAMR	<0.05	-2.14675
15003284 NM_001081784 // HTR2A // 5-hydroxytryptamine (serotonin) receptor 2A, G protein-coupled	HTR2A	<0.05	-2.14696
14926649 ---		<0.05	-2.14764
15112485 ---		<0.05	-2.15017
14937089 ---		<0.05	-2.15136
15084526 ---		<0.05	-2.15166
15029435 ---		<0.05	-2.15173
15011496 ---		<0.05	-2.15182
15072196 ---		<0.05	-2.15287
15109767		<0.05	-2.1537

15018117	---		<0.05	-2.15376
14929069	---		<0.05	-2.15405
14929505	---		<0.05	-2.15405
14929909	---		<0.05	-2.15557
14985315	NR_032935 // MIR143 // microRNA mir-143 // --- // 100314979	MIR143	<0.05	-2.15673
15136400	---		<0.05	-2.15769
14929901	---		<0.05	-2.15811
14951749	NM_001256845 // NCR1 // natural cytotoxicity triggering receptor 1 // --- // 100054918	NCR1	<0.05	-2.15978
15067801	---		<0.05	-2.16113
15112196	XM_001498150 // LOC100068286 // olfactory receptor 52M1-like // --- // 100068286 /// EN	LOC100068286	<0.05	-2.16115
14927633	---		<0.05	-2.16201
15083867	---		<0.05	-2.1624
15042338	---		<0.05	-2.16267
15116468	ENSECAT00000016251 // LIPG // lipase, endothelial // --- // 100053762 /// XM_001499159	LIPG	<0.05	-2.16445
14929605	---		<0.05	-2.16508
14925723	---		<0.05	-2.16701
15016687	ENSECAT00000012770 // LOC100068351 // microtubule-associated proteins 1A/1B light chain	LOC100068351	<0.05	-2.1671
15095042	XM_001497244 // LOC100067126 // olfactory receptor 12-like // --- // 100067126 /// ENSE	LOC100067126	<0.05	-2.16712
14927325	---		<0.05	-2.16718
14927349	---		<0.05	-2.16718
14927373	---		<0.05	-2.16718
14927397	---		<0.05	-2.16718
15005050	---		<0.05	-2.16755
15063686	---		<0.05	-2.16834
14952608	---		<0.05	-2.16915
15047019	---		<0.05	-2.16927
15049206	---		<0.05	-2.17
15083262	---		<0.05	-2.17022
14939891	ENSECAT00000013494 // TRAV30 // T cell receptor alpha variable 30 // --- // ---	TRAV30	<0.05	-2.17042
15046142	NR_033036 // MIR495 // microRNA mir-495 // --- // 100315115	MIR495	<0.05	-2.17149
15091406	ENSECAT00000010407 // LOC100061730 // protein S100-A9-like // --- // 100061730 /// XM_0	LOC100061730	<0.05	-2.17191
15124434	---		<0.05	-2.17204
14927841	---		<0.05	-2.17288
15093926	---		<0.05	-2.17297
14927697	---		<0.05	-2.17385
14926047	---		<0.05	-2.17444
14939536	XM_001502286 // LOC100072368 // olfactory receptor 4F3/4F16/4F29-like // --- // 1000723	LOC100072368	<0.05	-2.17452
15106002	---		<0.05	-2.17547
14925353	---		<0.05	-2.17582
15106372	---		<0.05	-2.17583
14926359	---		<0.05	-2.17621
15081980	XM_001498848 // LOC100054053 // FMRFamide-related peptides-like // --- // 100054053 ///	LOC100054053	<0.05	-2.17831
14947737	ENSECAT00000006789 // LOC100072163 // olfactory receptor 4F3/4F16/4F29-like // --- // 1	LOC100072163	<0.05	-2.17927
15137724	---		<0.05	-2.17938
14941018	---		<0.05	-2.1806
15117446	---		<0.05	-2.18083
14940594	---		<0.05	-2.18165
15087838	ENSECAT00000012045 // VAV3 // vav 3 guanine nucleotide exchange factor // --- // 100057	VAV3	<0.05	-2.18338
15136830	---		<0.05	-2.18414
15131883	---		<0.05	-2.18537
14928041	---		<0.05	-2.18605
15089731	---		<0.05	-2.18694
15027238	---		<0.05	-2.18848
14927179	---		<0.05	-2.18866
15046062	---		<0.05	-2.18925
15125830	---		<0.05	-2.18926
15042289	ENSECAT00000020238 // DMRTA1 // DMRT-like family A1 // --- // ---	DMRTA1	<0.05	-2.19167
15130903	---		<0.05	-2.19201
14929299	---		<0.05	-2.19236
15102129	---		<0.05	-2.19241
15004637	---		<0.05	-2.19246
15020168	XM_001499988 // NPY1R // neuropeptide Y receptor Y1 // --- // 100061686 /// ENSECAT0000	NPY1R	<0.05	-2.19292
14980143	NR_032930 // MIR365-2 // microRNA mir-365-2 // --- // 100315063	MIR365-2	<0.05	-2.19465
15133511	---		<0.05	-2.19504
14931755	---		<0.05	-2.19573
14936568	---		<0.05	-2.19741
14981770	---		<0.05	-2.19875
15093558	XM_001500300 // PDE4B // phosphodiesterase 4B, cAMP-specific // --- // 100053961 /// EN	PDE4B	<0.05	-2.19912
15105578	---		<0.05	-2.19913
15136742	---		<0.05	-2.19923
15123458	---		<0.05	-2.19928
14929179	---		<0.05	-2.19951
15017036	---		<0.05	-2.19956
14963807	ENSECAT00000000076 // LOC100056359 // meteorin-like protein-like // --- // 100056359 //	LOC100056359	<0.05	-2.19974
15072017	---		<0.05	-2.1999
15112182	XM_001917627 // LOC100147186 // olfactory receptor 52D1-like // --- // 100147186 /// EN	LOC100147186	<0.05	-2.20075
15088496	---		<0.05	-2.20119
15112289	XM_001499320 // LOC100069580 // putative olfactory receptor 52P1-like // --- // 1000695	LOC100069580	<0.05	-2.20183
15137368	---		<0.05	-2.20197
15137164	---		<0.05	-2.20388
14993111	---		<0.05	-2.20398
15001283	---		<0.05	-2.20417
14947729	XM_001501951 // LOC100072089 // olfactory receptor 4K14-like // --- // 100072089 /// EN	LOC100072089	<0.05	-2.20423
15135978	---		<0.05	-2.2044
14969553	NR_032923 // MIR670 // microRNA mir-670 // --- // 100315104	MIR670	<0.05	-2.20482
15137184	---		<0.05	-2.20531
15032308	ENSECAT00000023025 // LGSN // lengsin, lens protein with glutamine synthetase domain //	LGSN	<0.05	-2.2056
15040376	---		<0.05	-2.20577
15027354	XM_001503734 // LOC100052091 // olfactory receptor 1N1-like // --- // 100052091 /// ENS	LOC100052091	<0.05	-2.20593
15015540	---		<0.05	-2.20833
15006788	---		<0.05	-2.20906
14927445	---		<0.05	-2.2095
14981287	XM_001497144 // LOC100067002 // glucosamine--fructose-6-phosphate aminotransferase [iso	LOC100067002	<0.05	-2.20969
15087351	---		<0.05	-2.21011
14931429	---		<0.05	-2.21071
15137052	---		<0.05	-2.21101
15008818	---		<0.05	-2.21224
15030482	ENSECAT00000018981 // LOC100054813 // uncharacterized protein C6orf15-like // --- // 10	LOC100054813	<0.05	-2.2128
15136622	---		<0.05	-2.21359
15008740	ENSECAT00000019338 // LOC100147031 // primary ciliary dyskinesia protein 1-like // ---	LOC100147031	<0.05	-2.21446
15024933	XM_003364473 // LOC100054505 // stathmin-4-like // --- // 100054505 /// ENSECAT00000017	LOC100054505	<0.05	-2.21455
14967701	XM_001502293 // SCARF1 // scavenger receptor class F, member 1 // --- // 100072382 ///	SCARF1	<0.05	-2.21494
15111813	XM_001495302 // LOC100064387 // diacylglycerol O-acyltransferase 2-like // --- // 10006	LOC100064387	<0.05	-2.21537
14938632	NR_032776 // MIR1282 // microRNA mir-1282 // --- // 100314999	MIR1282	<0.05	-2.21653
15033324	---		<0.05	-2.21757
15137048	---		<0.05	-2.21793

15002568	---		<0.05	-2.21912
15130574	XM_001494562 // LOC100063279 // olfactory receptor 10R2-like // --- // 100063279 /// EN	LOC100063279	<0.05	-2.21915
14931939	---		<0.05	-2.21937
15093180	---		<0.05	-2.21973
15137000	---		<0.05	-2.21973
14991409	---		<0.05	-2.21979
15040878	ENSECAT00000013888 // LOC100053354 // breast carcinoma-amplified sequence 1-like // ---	LOC100053354	<0.05	-2.21987
14926583	---		<0.05	-2.2218
14927621	---		<0.05	-2.22258
14925587	---		<0.05	-2.22259
14930591	---		<0.05	-2.22259
15039071	---		<0.05	-2.2227
15007738	---		<0.05	-2.22284
15090288	ENSECAT00000015602 // CAMK1G // calcium/calmodulin-dependent protein kinase IG // --- /	CAMK1G	<0.05	-2.22529
14939556	XM_001502449 // LOC100072499 // olfactory receptor 11G2-like // --- // 100072499 /// EN	LOC100072499	<0.05	-2.22796
15090447	ENSECAT00000016862 // LOC100058208 // uncharacterized protein C1orf226-like // --- // 1	LOC100058208	<0.05	-2.22877
15083634	ENSECAT00000008396 // CRYGN // crystallin, gamma N // --- // ---	CRYGN	<0.05	-2.22929
14973720	---		<0.05	-2.23004
14927435	---		<0.05	-2.23195
14972584	XM_001488616 // LOC100053284 // olfactory receptor 4P4-like // --- // 100053284 /// ENS	LOC100053284	<0.05	-2.2333
14986942	---		<0.05	-2.23469
15090236	---		<0.05	-2.23507
15003570	---		<0.05	-2.23555
15010680	---		<0.05	-2.23625
14926415	---		<0.05	-2.23674
14939505	XM_001918267 // LOC100072107 // olfactory receptor 4L1-like // --- // 100072107 /// ENS	LOC100072107	<0.05	-2.23751
14927911	---		<0.05	-2.23785
15048207	---		<0.05	-2.23842
14994336	---		<0.05	-2.23905
14930957	---		<0.05	-2.23972
14930929	---		<0.05	-2.2398
15094377	ENSECAT00000006785 // LOC100061005 // potassium voltage-gated channel subfamily E membe	LOC100061005	<0.05	-2.23993
14936801	---		<0.05	-2.24103
14997111	ENSECAT00000027151 // CDCP1 // CUB domain containing protein 1 // --- // 100065703 ///	CDCP1	<0.05	-2.24254
15118666			<0.05	-2.24269
15015246	ENSECAT00000011123 // HHLA2 // HERV-H LTR-associating 2 // --- // ---	HHLA2	<0.05	-2.2436
15047661	---		<0.05	-2.24532
15014454	---		<0.05	-2.24657
15125938	XM_001499571 // LOC100065839 // claudin-6-like // --- // 100065839 /// ENSECAT000000037	LOC100065839	<0.05	-2.24657
14928887	---		<0.05	-2.24719
15133003	---		<0.05	-2.24738
15113559	NR_032877 // MIR130B // microRNA mir-130b // --- // 100314829	MIR130B	<0.05	-2.24747
14975469	ENSECAT00000024168 // LOC100630602 // c-C motif chemokine 26-like // --- // 100630602 /	LOC100630602	<0.05	-2.24764
15020195	---		<0.05	-2.24924
15121886	ENSECAT00000025690 // LOC100060072 // uncharacterized protein C8orf47-like // --- // 10	LOC100060072	<0.05	-2.25113
14997796	XM_001496182 // ANKRD28 // ankyrin repeat domain 28 // --- // 100052418 /// ENSECAT0000	ANKRD28	<0.05	-2.25115
15109309	---		<0.05	-2.25128
14926843	---		<0.05	-2.25158
14961651	---		<0.05	-2.25213
14942247	ENSECAT00000015611 // SCD // stearoyl-CoA desaturase (delta-9-desaturase) // --- // 100	SCD	<0.05	-2.25238
14928973	---		<0.05	-2.2531
15105255	XM_001501884 // LOC100072027 // olfactory receptor 8G2-like // --- // 100072027 /// ENS	LOC100072027	<0.05	-2.2537
15032472	XM_001499511 // LOC100069781 // olfactory receptor 1F1-like // --- // 100069781 /// ENS	LOC100069781	<0.05	-2.25829
15067810	---		<0.05	-2.25861
15056190	---		<0.05	-2.25961
15136746	---		<0.05	-2.25961
14930263	---		<0.05	-2.26051
15062751	NM_001081908 // AKR1C1 // aldo-keto reductase family 1, member C1 // --- // 100034073 /	AKR1C1	<0.05	-2.26074
14925599	---		<0.05	-2.2608
14930603	---		<0.05	-2.2608
14952411	ENSECAT00000020664 // LOC100629691 // uncharacterized protein C6orf163-like // --- // 1	LOC100629691	<0.05	-2.26236
14990938	XM_001918082 // LOC100146419 // RAD51-associated protein 2-like // --- // 100146419 ///	LOC100146419	<0.05	-2.26254
14935840	XM_001497959 // LOC100068061 // hepatoma-derived growth factor-related protein 3-like /	LOC100068061	<0.05	-2.26264
15057679	---		<0.05	-2.26417
15127690	---		<0.05	-2.26528
15123982	---		<0.05	-2.26547
14925729	---		<0.05	-2.26585
14930001	---		<0.05	-2.26619
14997427	ENSECAT00000021523 // LOC100054924 // serine/threonine-protein kinase DCLK3-like // ---	LOC100054924	<0.05	-2.2667
15043749	XM_003363960 // LOC100629885 // interferon alpha-2-like // --- // 100629885 /// ENSECAT	LOC100629885	<0.05	-2.26809
15102171	XM_001494579 // LOC100063302 // keratin, type II cytoskeletal 1-like // --- // 10006330	LOC100063302	<0.05	-2.26966
14931383	---		<0.05	-2.27123
15112114	XM_001500854 // LOC100053374 // putative RNA polymerase II subunit A C-terminal domain	LOC100053374	<0.05	-2.27265
15136342	---		<0.05	-2.27277
15134534	---		<0.05	-2.27312
14988502	XM_001495750 // LOC100065031 // interleukin-1 family member 9-like // --- // 100065031	LOC100065031	<0.05	-2.27341
15039487	NM_001091537 // PRND // prion protein 2 (dublet) // --- // 100048937 /// ENSECAT0000000	PRND	<0.05	-2.27356
14984183	---		<0.05	-2.27492
15120701	---		<0.05	-2.27525
14925625	---		<0.05	-2.27561
15030159	XM_001496061 // LOC100065498 // olfactory receptor 2M2-like // --- // 100065498 /// ENS	LOC100065498	<0.05	-2.27576
14982483	---		<0.05	-2.27846
15098850	---		<0.05	-2.27989
14934384	---		<0.05	-2.28018
14989296	---		<0.05	-2.28085
14966148	NR_032903 // MIR196A // microRNA mir-196a // --- // 100314843	MIR196A	<0.05	-2.28094
15048241	---		<0.05	-2.28112
15137080	---		<0.05	-2.28156
15011487	---		<0.05	-2.28232
14991538	ENSECAT00000000377 // IL-1RII // interleukin-1 receptor type II // --- // 100033831 ///	IL-1RII	<0.05	-2.28279
15001479	XM_001494115 // RARB // retinoic acid receptor, beta // --- // 100051546 /// ENSECAT000	RARB	<0.05	-2.28315
15026776	---		<0.05	-2.2834
14950897	XM_001488567 // LOC100053135 // epididymal sperm-binding protein 1-like // --- // 10005	LOC100053135	<0.05	-2.28345
15024667	NR_032796 // MIR200B // microRNA mir-200b // --- // 100315038	MIR200B	<0.05	-2.28367
15126455	---		<0.05	-2.28371
14931055	---		<0.05	-2.28542
14994632	ENSECAT00000014191 // DCDC2C // doublecortin domain containing 2C // --- // ---	DCDC2C	<0.05	-2.28664
15056574	GQ259798 // DEFA35L // Paneth cell-specific alpha-defensin 35L // --- // 100307022 ///	DEFA35L	<0.05	-2.28773
14932303	---		<0.05	-2.28819
15093784	---		<0.05	-2.28848
14930455	---		<0.05	-2.28854
14954885	---		<0.05	-2.28963
15091426	ENSECAT00000017022 // LOC100061178 // cornifin-B-like // --- // 100061178 /// XM_001916	LOC100061178	<0.05	-2.29013
14931169	---		<0.05	-2.29063
15116069	---		<0.05	-2.29152
15126654			<0.05	-2.29168

14934256	---		<0.05	-2.29206
15110843	ENSECAT00000014881 // LOC100064480 // zinc finger protein 709-like // --- // 100064480	LOC100064480	<0.05	-2.29462
14970590	---		<0.05	-2.29479
15136818	---		<0.05	-2.29574
15006107	---		<0.05	-2.29882
15127446	---		<0.05	-2.29884
14970011	XM_001497002 // LOC100066827 // olfactory receptor 5AK2-like // --- // 100066827 /// EN	LOC100066827	<0.05	-2.29929
15048972	ENSECAT00000017958 // LOC100062681 // cylicin-2-like // --- // 100062681 /// XM_0014941	LOC100062681	<0.05	-2.29975
15008384	---		<0.05	-2.29993
15125592	---		<0.05	-2.30087
14925117	---		<0.05	-2.30105
15130250	ENSECAT00000021951 // LOC100069284 // high mobility group protein B3-like // --- // 100	LOC100069284	<0.05	-2.30159
15079475	ENSECAT00000016877 // LOC100055297 // anionic trypsin-like // --- // 100055297 /// XM_0	LOC100055297	<0.05	-2.30553
14982654	---		<0.05	-2.30573
15085890	XM_001915440 // LOC100146177 // olfactory receptor 10K2-like // --- // 100146177 /// EN	LOC100146177	<0.05	-2.3066
14939486	XM_001501852 // LOC100072004 // olfactory receptor 4K17-like // --- // 100072004 /// EN	LOC100072004	<0.05	-2.30774
15137540	---		<0.05	-2.30796
14981828	---		<0.05	-2.30819
15007741	---		<0.05	-2.30951
14982669	ENSECAT00000013882 // NRG2 // neuregulin 2 // --- // ---	NRG2	<0.05	-2.3101
15097454	XM_001489041 // LOC100054306 // olfactory receptor 6C4-like // --- // 100054306 /// ENS	LOC100054306	<0.05	-2.31078
14935048	---		<0.05	-2.31093
14925499	---		<0.05	-2.31104
15115622	---		<0.05	-2.31164
15122140	---		<0.05	-2.31239
15039844	---		<0.05	-2.31424
15103454	XM_001493821 // LOC100059768 // growth arrest and DNA damage-inducible protein GADD45 b	LOC100059768	<0.05	-2.31558
15057269	---		<0.05	-2.31564
15045046	XM_001489156 // PCNX // pecanex homolog (Drosophila) // --- // 100054565 /// ENSECAT000	PCNX	<0.05	-2.3161
15105518	ENSECAT00000023734 // APLP2 // amyloid beta (A4) precursor-like protein 2 // --- // 100	APLP2	<0.05	-2.31819
14949236	---		<0.05	-2.31924
14961653	ENSECAT00000027009 // LOC100071483 // uncharacterized protein C17orf78 homolog // --- /	LOC100071483	<0.05	-2.31941
15063849	---		<0.05	-2.31977
14943785	---		<0.05	-2.31986
14926755	---		<0.05	-2.32152
15110163	XM_001501616 // LOC100071761 // olfactory receptor 6M1-like // --- // 100071761 /// ENS	LOC100071761	<0.05	-2.32169
15136792	---		<0.05	-2.32195
15016197	---		<0.05	-2.3225
15034398	XM_001493989 // LOC100062413 // olfactory receptor 7A17-like // --- // 100062413 /// EN	LOC100062413	<0.05	-2.32376
14929427	---		<0.05	-2.32535
15007657	XM_001917556 // ZNF804A // zinc finger protein 804A // --- // 100054190 /// ENSECAT0000	ZNF804A	<0.05	-2.32727
14930649	---		<0.05	-2.32879
14977278	XM_003362763 // LOC100069286 // pyrin-like // --- // 100069286 /// ENSECAT00000006974 /	LOC100069286	<0.05	-2.33089
14926315	---		<0.05	-2.33159
15079527	ENSECAT00000003584 // LOC100056093 // transmembrane protein 139-like // --- // 10005609	LOC100056093	<0.05	-2.33185
14927229	---		<0.05	-2.33274
15023913	ENSECAT00000005694 // LOC100050750 // transmembrane protein 51-like // --- // 100050750	LOC100050750	<0.05	-2.333
15136700	---		<0.05	-2.33301
15055290	---		<0.05	-2.33422
14950893	NM_001193462 // SULT2A1 // sulfotransferase family, cytosolic, 2A, dehydroepiandrostero	SULT2A1	<0.05	-2.33452
15131122	---		<0.05	-2.33465
15027185	---		<0.05	-2.33507
15082034	NR_032823 // MIR196B // microRNA mir-196b // --- // 100315100	MIR196B	<0.05	-2.33559
14929433	---		<0.05	-2.33571
14998872	---		<0.05	-2.33717
15046134	---		<0.05	-2.33738
15068091	ENSECAT00000015583 // LOC100069539 // cytokine-dependent hematopoietic cell linker-like	LOC100069539	<0.05	-2.33852
15086644	XM_001493530 // LOC100061663 // protein S100-A9-like // --- // 100061663 /// ENSECAT000	LOC100061663	<0.05	-2.33919
15043499	---		<0.05	-2.34041
14991769	---		<0.05	-2.34055
15132009	---		<0.05	-2.34086
14928005	---		<0.05	-2.34142
14927989	---		<0.05	-2.34581
14956669	---		<0.05	-2.34608
15128319	---		<0.05	-2.34666
15097702	ENSECAT00000023772 // IL23A // interleukin 23, alpha subunit p19 // --- // 100034230 //	IL23A	<0.05	-2.34864
15093701	---		<0.05	-2.34999
15129283	---		<0.05	-2.35016
15020279	---		<0.05	-2.35147
15134584	---		<0.05	-2.35304
14925339	---		<0.05	-2.35565
14947779	XM_001502402 // LOC100072469 // olfactory receptor 4K2-like // --- // 100072469 /// ENS	LOC100072469	<0.05	-2.35613
14932548	---		<0.05	-2.35613
15120831	---		<0.05	-2.35621
14926627	---		<0.05	-2.35651
15110353	ENSECAT00000018456 // CDON // Cdon homolog (mouse) // --- // 100064474 /// ENSECAT00000	CDON	<0.05	-2.35741
14926773	---		<0.05	-2.35883
14931481	---		<0.05	-2.36007
15065410	---		<0.05	-2.3618
14961330	XM_001500567 // LOC100070889 // olfactory receptor 4D2-like // --- // 100070889 /// ENS	LOC100070889	<0.05	-2.36344
15100780	---		<0.05	-2.36517
15128835	---		<0.05	-2.3655
15120505	---		<0.05	-2.3656
14978879	---		<0.05	-2.36669
15116646	NR_032876 // MIR122 // microRNA mir-122 // --- // 100315102	MIR122	<0.05	-2.36729
14931891	---		<0.05	-2.37098
15071545	ENSECAT00000020434 // PDGFRA // platelet-derived growth factor receptor, alpha polypept	PDGFRA	<0.05	-2.37129
15136694	---		<0.05	-2.37171
14997862	ENSECAT00000026805 // SH3BP5 // SH3-domain binding protein 5 (BTK-associated) // --- //	SH3BP5	<0.05	-2.37375
15108307	ENSECAT00000020310 // FSHB // follicle stimulating hormone, beta polypeptide // --- //	FSHB	<0.05	-2.37434
15102491	XM_001490066 // LOC100056234 // olfactory receptor 6C76-like // --- // 100056234 /// EN	LOC100056234	<0.05	-2.37785
14925923	---		<0.05	-2.37796
15027870	---		<0.05	-2.37836
14927153	---		<0.05	-2.37858
14966403	ENSECAT00000023232 // LOC100070690 // uncharacterized protein C17orf67 homolog // --- /	LOC100070690	<0.05	-2.37912
15111490	---		<0.05	-2.37943
14931353	---		<0.05	-2.38005
14927447	---		<0.05	-2.38073
15136186	---		<0.05	-2.38253
15108925	---		<0.05	-2.3834
15136208	---		<0.05	-2.38613
14931363	---		<0.05	-2.38854
14989256	ENSECAT00000009642 // LOC100061844 // CB1 cannabinoid receptor-interacting protein 1-li	LOC100061844	<0.05	-2.38911
15029742	ENSECAT00000008298 // C6orf52 // chromosome 6 open reading frame 52 // --- // ---	C6orf52	<0.05	-2.38925
15137664	---		<0.05	-2.39127
14928337	---		<0.05	-2.39497

14928425	---		<0.05	-2.39497
15113302	---		<0.05	-2.39637
15049505	XM_001501205 // LOC100071445 // olfactory receptor 1G1-like // --- // 100071445 /// ENS	LOC100071445	<0.05	-2.39644
15072009	---		<0.05	-2.39698
15025214	---		<0.05	-2.39784
14925821	---		<0.05	-2.39983
14925851	---		<0.05	-2.39983
14925893	---		<0.05	-2.39983
14925965	---		<0.05	-2.39983
15013947	---		<0.05	-2.40129
15010599	---		<0.05	-2.40175
15078871	ENSECAT00000019550 // LOC100057093 // uncharacterized protein C7orf45 homolog // --- //	LOC100057093	<0.05	-2.40211
14929773	---		<0.05	-2.40252
15003423	---		<0.05	-2.40494
14926103	---		<0.05	-2.4057
15137004	---		<0.05	-2.40923
14944245	XM_001493698 // LOC100061933 // olfactory receptor 6C4-like // --- // 100061933 /// ENS	LOC100061933	<0.05	-2.40961
14929951	---		<0.05	-2.41249
15107307	---		<0.05	-2.4146
15137410	---		<0.05	-2.41677
15058153	---		<0.05	-2.41702
15085901	ENSECAT00000025464 // LOC100054450 // CD5 antigen-like // --- // 100054450 /// XM_00148	LOC100054450	<0.05	-2.41868
14970530	---		<0.05	-2.42003
15016746	---		<0.05	-2.42022
15002592	---		<0.05	-2.42048
14927993	---		<0.05	-2.422
15099749	---		<0.05	-2.42203
15076010	---		<0.05	-2.42232
14967870	XM_001502636 // LOC100072630 // olfactory receptor 3A2-like // --- // 100072630 /// ENS	LOC100072630	<0.05	-2.42247
15120578	XM_001490831 // LOC100057505 // serpin B4-like // --- // 100057505 /// ENSECAT000000227	LOC100057505	<0.05	-2.4228
15003530	---		<0.05	-2.42413
14925351	---		<0.05	-2.42415
15054923	AF541975 // ADAMTS1 // ADAM metallopeptidase with thrombospondin type 1 motif, 1 // ---	ADAMTS1	<0.05	-2.42455
15136212	---		<0.05	-2.42489
14926817	---		<0.05	-2.42882
14946819	---		<0.05	-2.43032
15026827	ENSECAT00000015816 // PHACTR1 // phosphatase and actin regulator 1 // --- // 100051555	PHACTR1	<0.05	-2.43301
15096793	XM_001491393 // LOC100058417 // olfactory receptor 8S1-like // --- // 100058417 /// ENS	LOC100058417	<0.05	-2.43312
15062453	---		<0.05	-2.4361
15083389	XM_001490410 // LOC100056816 // olfactory receptor 2F1-like // --- // 100056816 /// ENS	LOC100056816	<0.05	-2.43653
14943795	---		<0.05	-2.43975
15057447	---		<0.05	-2.44065
14931771	---		<0.05	-2.44085
14926449	---		<0.05	-2.44171
15077218	---		<0.05	-2.4418
15074480	---		<0.05	-2.442
15060014	---		<0.05	-2.44221
15136164	---		<0.05	-2.44322
15065561	NM_001109812 // CDH13 // cadherin 13, H-cadherin (heart) // --- // 100055760 /// ENSECA	CDH13	<0.05	-2.44327
15085386	NR_032844 // MIR29B-2 // microRNA mir-29b-2 // --- // 100315047	MIR29B-2	<0.05	-2.44422
14993776	---		<0.05	-2.44489
15131182	---		<0.05	-2.44546
15098375	---		<0.05	-2.44725
15049479	XM_001501396 // LOC100071600 // olfactory receptor 1J2-like // --- // 100071600 /// ENS	LOC100071600	<0.05	-2.44745
14926221	---		<0.05	-2.44964
15080568	XM_001491909 // PNPLA8 // patatin-like phospholipase domain containing 8 // --- // 1000	PNPLA8	<0.05	-2.45185
14962729	---		<0.05	-2.45185
14981211	---		<0.05	-2.45193
14992499	XM_001491488 // LOC100058571 // probable N-acetyltransferase 8B-like // --- // 10005857	LOC100058571	<0.05	-2.45394
15134642	---		<0.05	-2.45529
15089660	---		<0.05	-2.45587
14929085	---		<0.05	-2.45606
14933804	---		<0.05	-2.45617
15063348	ENSECAT00000009357 // LOC100068038 // ADP-ribosylation factor-like protein 5B-like // -	LOC100068038	<0.05	-2.4567
14929657	---		<0.05	-2.4568
14969995	---		<0.05	-2.45838
15135982	---		<0.05	-2.45863
15082316	---		<0.05	-2.45914
14926727	---		<0.05	-2.46016
15082025	XM_001499476 // HOXA6 // homeobox A6 // --- // 100054438 /// ENSECAT00000000495 // HOXA	HOXA6	<0.05	-2.4609
15137190	---		<0.05	-2.46112
15097448	---		<0.05	-2.46174
14927183	---		<0.05	-2.46318
14935447	XM_001493617 // LOC100061806 // olfactory receptor 6S1-like // --- // 100061806 /// ENS	LOC100061806	<0.05	-2.46376
15042813	ENSECAT00000018097 // LOC100052947 // protein FAM75D1-like // --- // 100052947 /// XM_0	LOC100052947	<0.05	-2.46422
14985881	---		<0.05	-2.46428
15083298	ENSECAT00000022470 // LOC100050047 // cationic trypsin-3-like // --- // 100050047 /// X	LOC100050047	<0.05	-2.46431
15091419	XM_003365022 // LOC100630233 // uncharacterized LOC100630233 // --- // 100630233 /// EN	LOC100630233	<0.05	-2.46485
14930629	---		<0.05	-2.46599
15130168	---		<0.05	-2.46696
14925567	---		<0.05	-2.46778
14930571	---		<0.05	-2.46778
14930237	---		<0.05	-2.46788
14993012	NR_032944 // MIR1461 // microRNA mir-1461 // --- // 100315016 /// NR_032946 // MIR216B	MIR1461	<0.05	-2.46815
14930179	---		<0.05	-2.46881
14988258			<0.05	-2.47081
14966184	---		<0.05	-2.47091
15091451	XM_003365028 // LOC100630664 // RIIa domain-containing protein 1-like // --- // 1006306	LOC100630664	<0.05	-2.47106
14926841	---		<0.05	-2.47314
15090174	---		<0.05	-2.47336
15136456	---		<0.05	-2.47404
15105676	---		<0.05	-2.47471
14991443	---		<0.05	-2.47476
15136196	---		<0.05	-2.47476
15046683	---		<0.05	-2.4776
15071236	NM_001081884 // CSN3 // casein kappa // --- // 100033983 /// ENSECAT00000001652 // CSN3	CSN3	<0.05	-2.47784
14937805	---		<0.05	-2.48279
15128959	---		<0.05	-2.48543
14926383	---		<0.05	-2.48785
15128441	---		<0.05	-2.48831
14950232	---		<0.05	-2.48846
14966036	---		<0.05	-2.48909
14927487	---		<0.05	-2.49015
15137360	---		<0.05	-2.49061
14953119	ENSECAT00000021434 // RSPO3 // R-spondin 3 // --- // 100067377 /// NM_001109682 // RSPO	RSPO3	<0.05	-2.49061
14952671	---		<0.05	-2.49098

15029932	---		<0.05	-2.49122
15095349	---		<0.05	-2.49317
14957439	---		<0.05	-2.49425
14941806	---		<0.05	-2.49481
15079350	---		<0.05	-2.495
15125808	XM_001917039 // LOC100146654 // immunoglobulin lambda-like polypeptide 5-like // --- //	LOC100146654	<0.05	-2.49543
15026746	---		<0.05	-2.49724
14930731	---		<0.05	-2.4979
14968833	NR_015344 // MYH4 // myosin, heavy chain 4, skeletal muscle pseudogene // --- // 100062	MYH4	<0.05	-2.49845
15101066	---		<0.05	-2.49854
15002547	---		<0.05	-2.50194
14928957	---		<0.05	-2.50716
15002234	XM_001491224 // LOC100050742 // transmembrane 4 L6 family member 1-like // --- // 10005	LOC100050742	<0.05	-2.50775
15137106	---		<0.05	-2.50838
14996191	---		<0.05	-2.50912
14983534	---		<0.05	-2.51041
15119496	---		<0.05	-2.51079
15137752	---		<0.05	-2.5122
14939964	---		<0.05	-2.51411
14926419	---		<0.05	-2.51553
14928071	---		<0.05	-2.51658
14999271	---		<0.05	-2.51659
14952041	---		<0.05	-2.51984
14991548	ENSECAT00000009189 // LOC100058919 // mitogen-activated protein kinase kinase kinase ki	LOC100058919	<0.05	-2.52044
14927061	---		<0.05	-2.52046
14988315	---		<0.05	-2.52055
15079357	---		<0.05	-2.52121
14965572	XM_001496268 // LOC100065767 // membrane primary amine oxidase-like // --- // 100065767	LOC100065767	<0.05	-2.52166
15121226	---		<0.05	-2.52337
15035314	---		<0.05	-2.52425
14929367	---		<0.05	-2.52627
15043755	NR_032994 // MIR31 // microRNA mir-31 // --- // 100315075	MIR31	<0.05	-2.52966
14926905	---		<0.05	-2.53194
14927199	---		<0.05	-2.53223
15043815	ENSECAT00000022313 // C9orf11 // chromosome 9 open reading frame 11 // --- // ---	C9orf11	<0.05	-2.53292
14932229	---		<0.05	-2.53408
15076314	---		<0.05	-2.5358
14931273	---		<0.05	-2.53588
14928539	---		<0.05	-2.5378
15074744	---		<0.05	-2.53783
14985592	ENSECAT00000004848 // PCDHB10 // protocadherin beta 10 // --- // ---	PCDHB10	<0.05	-2.54032
15137296	---		<0.05	-2.54306
15136496	---		<0.05	-2.54325
14934309	---		<0.05	-2.54411
15090612	---		<0.05	-2.54502
15024826	XM_001914958 // LOC100057715 // phosphatidylethanolamine-binding protein 4-like // ---	LOC100057715	<0.05	-2.54583
15093314	---		<0.05	-2.54609
14931577	---		<0.05	-2.54921
15112441	---		<0.05	-2.55077
15044306	---		<0.05	-2.55194
14928095	---		<0.05	-2.55367
15070451	---		<0.05	-2.55458
14928101	---		<0.05	-2.55754
15013338	XM_001503354 // CBLB // Cbl proto-oncogene, E3 ubiquitin protein ligase B // --- // 100	CBLB	<0.05	-2.55817
15108299	---		<0.05	-2.5591
14927221	---		<0.05	-2.56008
15132334	---		<0.05	-2.56081
15070053	ENSECAT00000009904 // SLC7A5 // solute carrier family 7 (amino acid transporter light c	SLC7A5	<0.05	-2.56166
15016558	NM_001163971 // SLC2A1 // solute carrier family 2 (facilitated glucose transporter), me	SLC2A1	<0.05	-2.56175
15020662	---		<0.05	-2.56186
15055752	---		<0.05	-2.56298
15079436	---		<0.05	-2.56307
15137142	---		<0.05	-2.56436
14926597	---		<0.05	-2.56438
14929165	---		<0.05	-2.56535
15082155	---		<0.05	-2.56541
15135180	---		<0.05	-2.56618
15075503	XM_001495199 // LOC100064229 // NKG2D ligand 4-like // --- // 100064229 /// ENSECAT0000	LOC100064229	<0.05	-2.56677
14954220	---		<0.05	-2.5695
15034066	---		<0.05	-2.57003
14939998	ENSECAT00000017987 // TRAV40 // T cell receptor alpha variable 40 // --- // ---	TRAV40	<0.05	-2.57206
15048211	XM_003364027 // LOC100630573 // uncharacterized LOC100630573 // --- // 100630573 /// EN	LOC100630573	<0.05	-2.57351
15056065	---		<0.05	-2.57771
14946612	---		<0.05	-2.57951
15137600	---		<0.05	-2.58036
15036483	---		<0.05	-2.58215
15112692	XM_001504920 // LOC100055858 // lymphatic vessel endothelial hyaluronic acid receptor 1	LOC100055858	<0.05	-2.58504
14953019	AJ319910 // CX43 // connexin 43 // --- // 100067229	CX43	<0.05	-2.58604
14954359	---		<0.05	-2.58658
14929407	---		<0.05	-2.58902
15132007	---		<0.05	-2.58914
14956181	---		<0.05	-2.58939
14929497	---		<0.05	-2.59114
15110192	---		<0.05	-2.594
15014202	ENSECAT00000006095 // LOC100059159 // thrombopoietin-like // --- // 100059159 /// XM_00	LOC100059159	<0.05	-2.59413
15132900	XM_001489536 // CHRDL1 // chordin-like 1 // --- // 100059105 /// ENSECAT00000012735 //	CHRDL1	<0.05	-2.5946
15111238	---		<0.05	-2.59608
15123605	---		<0.05	-2.59712
15070950	---		<0.05	-2.59875
15101112	---		<0.05	-2.59998
15112164	---		<0.05	-2.60059
15044644	---		<0.05	-2.60074
15021008	---		<0.05	-2.60148
15115595	XM_001489548 // LOC100055301 // thioredoxin domain-containing protein 2-like // --- //	LOC100055301	<0.05	-2.60206
15026036	ENSECAT00000015464 // FGF2 // fibroblast growth factor 2 (basic) // --- // 100033955 //	FGF2	<0.05	-2.60287
15136394	---		<0.05	-2.60763
14972150	ENSECAT00000016926 // LOC100067133 // excitatory amino acid transporter 2-like // --- /	LOC100067133	<0.05	-2.60906
15066093	XM_001497779 // LOC100067821 // zinc transporter ZIP8-like // --- // 100067821 /// ENSE	LOC100067821	<0.05	-2.61334
14980260	ENSECAT00000024095 // ATF7IP2 // activating transcription factor 7 interacting protein	ATF7IP2	<0.05	-2.61344
14996187	---		<0.05	-2.61454
15128317	---		<0.05	-2.61545
15013212	ENSECAT00000001188 // LOC100061617 // zinc finger BED domain-containing protein 2-like	LOC100061617	<0.05	-2.61894
15007756	ENSECAT00000026771 // COL3A1 // collagen, type III, alpha 1 // --- // 100034123	COL3A1	<0.05	-2.61906
15005709	---		<0.05	-2.62044
15028958			<0.05	-2.62218
14955496	---		<0.05	-2.62283

14935394	---		<0.05	-2.62368
14948020	---		<0.05	-2.6237
15082554	---		<0.05	-2.62452
15132247	---		<0.05	-2.62511
14953591	XM_001491402 // LOC100058436 // histo-blood group ABO system transferase-like // --- //	LOC100058436	<0.05	-2.62571
15021253	---		<0.05	-2.62683
14929249	---		<0.05	-2.62714
15111627	---		<0.05	-2.63169
15116648	XM_001489163 // MALT1 // mucosa associated lymphoid tissue lymphoma translocation gene	MALT1	<0.05	-2.63238
15015210	---		<0.05	-2.63473
15059852	---		<0.05	-2.63741
14998829	---		<0.05	-2.63949
15128374	---		<0.05	-2.64073
15093311	---		<0.05	-2.64239
14994469	---		<0.05	-2.64386
15055906	---		<0.05	-2.64524
14982111	---		<0.05	-2.6464
15136586	---		<0.05	-2.64874
14930633	---		<0.05	-2.65086
15013465	---		<0.05	-2.65212
15114568			<0.05	-2.6535
14954214	---		<0.05	-2.65353
14926935	---		<0.05	-2.656
14931407	---		<0.05	-2.65709
15029122	---		<0.05	-2.65724
15131837	---		<0.05	-2.65745
15125678	---		<0.05	-2.65757
14952511	---		<0.05	-2.66101
15136596	---		<0.05	-2.66428
14942885	---		<0.05	-2.66902
14930081	---		<0.05	-2.67337
15005189	---		<0.05	-2.67407
14927939	---		<0.05	-2.67421
15098184	ENSECAT00000029138 // HMGA2 // high mobility group AT-hook 2 // --- // ---	HMGA2	<0.05	-2.67538
15134924	---		<0.05	-2.67579
15105833	ENSECAT00000012847 // SYCE2 // synaptonemal complex central element protein 2 // --- //	SYCE2	<0.05	-2.68015
14943623	---		<0.05	-2.68179
14929155	---		<0.05	-2.68219
15127028	---		<0.05	-2.68383
14988261			<0.05	-2.68404
14929191	---		<0.05	-2.68569
15077811	XM_001497221 // ITGB8 // integrin, beta 8 // --- // 100053462 /// ENSECAT00000020530 //	ITGB8	<0.05	-2.68677
15134250	---		<0.05	-2.68735
14926417	---		<0.05	-2.68826
15028959			<0.05	-2.69015
15032288	---		<0.05	-2.69093
15071042	---		<0.05	-2.69144
15128655	---		<0.05	-2.69222
15023175	XM_001500815 // LOC100071130 // CAMPATH-1 antigen-like // --- // 100071130 /// ENSECAT0	LOC100071130	<0.05	-2.69322
15136524	---		<0.05	-2.69613
15060055	---		<0.05	-2.6968
15137658	---		<0.05	-2.69751
15137508	---		<0.05	-2.69844
15131301	---		<0.05	-2.70081
14948811	---		<0.05	-2.70464
14925671	---		<0.05	-2.7067
14925901	---		<0.05	-2.7067
14973455	---		<0.05	-2.70728
15132242	---		<0.05	-2.70883
15043462	---		<0.05	-2.70911
15120425	ENSECAT00000022867 // ATP8B1 // ATPase, aminophospholipid transporter, class I, type 8B	ATP8B1	<0.05	-2.70944
15006835	ENSECAT00000002082 // LOC100058814 // keratin, type I cytoskeletal 18-like // --- // 10	LOC100058814	<0.05	-2.71016
15004573	---		<0.05	-2.71026
14934999	---		<0.05	-2.71062
14984598	---		<0.05	-2.71088
15058195	---		<0.05	-2.712
14931779	---		<0.05	-2.71435
15107278	XM_001496836 // LOC100066618 // olfactory receptor 52B4-like // --- // 100066618 /// EN	LOC100066618	<0.05	-2.71543
14925389	---		<0.05	-2.71786
15013363	---		<0.05	-2.72013
15137072	---		<0.05	-2.72136
15119703	---		<0.05	-2.72177
15137482	---		<0.05	-2.72384
14956176	---		<0.05	-2.72409
14929601	---		<0.05	-2.72411
15104496	---		<0.05	-2.72447
14928015	---		<0.05	-2.72533
15131583	---		<0.05	-2.72906
14931595	---		<0.05	-2.72975
14927791	---		<0.05	-2.73122
14929171	---		<0.05	-2.7357
14947718	---		<0.05	-2.73641
15124307	XM_001496042 // TRPS1 // trichorhinophalangeal syndrome I // --- // 100056821 /// ENSEC	TRPS1	<0.05	-2.73673
15055978	AY246707 // FGFR1 // fibroblast growth factor receptor 1 // --- // 100057614 /// XM_001	FGFR1	<0.05	-2.73721
14931351	---		<0.05	-2.73734
14946849	---		<0.05	-2.73911
15062192	---		<0.05	-2.74001
14926175	---		<0.05	-2.74106
15057924	---		<0.05	-2.74216
14929635	---		<0.05	-2.74317
14936792	---		<0.05	-2.74449
15136630	---		<0.05	-2.74454
15107295	---		<0.05	-2.75079
15125627	---		<0.05	-2.75121
15068729	---		<0.05	-2.75234
14926291	---		<0.05	-2.75459
14987862	---		<0.05	-2.75528
15112962	---		<0.05	-2.75734
15003549	---		<0.05	-2.75798
14969057	---		<0.05	-2.75872
15132415	---		<0.05	-2.75907
14926929	---		<0.05	-2.75929
14925719	---		<0.05	-2.7604
15020276	---		<0.05	-2.76164
15110253	XM_001502061 // LOC100072182 // olfactory receptor 8B12-like // --- // 100072182 /// EN	LOC100072182	<0.05	-2.76445
14930399	---		<0.05	-2.76848

15128724	---		<0.05	-2.76898
14986706	---		<0.05	-2.7698
15115552	XM_001489105 // LOC100054445 // NADH dehydrogenase [ubiquinone] flavoprotein 2, mitocho	LOC100054445	<0.05	-2.7731
15057944	---		<0.05	-2.77475
15100479	---		<0.05	-2.77487
14928965	---		<0.05	-2.78008
15073589	---		<0.05	-2.78026
14939786	---		<0.05	-2.78362
14926439	---		<0.05	-2.78597
15017465	---		<0.05	-2.78746
14931389	---		<0.05	-2.78977
15026539	---		<0.05	-2.79191
14929087	---		<0.05	-2.792
14992065	XM_001497574 // LOC100052694 // protein TSC21-like // --- // 100052694 /// ENSECAT00000	LOC100052694	<0.05	-2.79246
15113583	---		<0.05	-2.79647
15136340	---		<0.05	-2.79679
15066022	---		<0.05	-2.79755
15059530	ENSECAT00000009567 // LOC100050839 // beta-parvin-like // --- // 100050839 /// XM_00148	LOC100050839	<0.05	-2.7976
15014061	---		<0.05	-2.7981
15133229	---		<0.05	-2.79833
15093426	---		<0.05	-2.79945
14972903	XM_001498418 // LOC100068597 // olfactory receptor 5A1-like // --- // 100068597 /// ENS	LOC100068597	<0.05	-2.7995
14936239	---		<0.05	-2.79997
14932235	---		<0.05	-2.80118
14991401	---		<0.05	-2.8032
15137476	---		<0.05	-2.80444
14929307	---		<0.05	-2.8061
14927071	---		<0.05	-2.80737
15086407	ENSECAT00000015768 // LOC100063020 // pygopus homolog 2-like // --- // 100063020 /// XM	LOC100063020	<0.05	-2.80855
15112126	---		<0.05	-2.80968
14940014	---		<0.05	-2.8152
15132966	---		<0.05	-2.81615
15042170	---		<0.05	-2.8168
14932207	---		<0.05	-2.81716
14976277	---		<0.05	-2.81948
14939967	---		<0.05	-2.82331
15088547	---		<0.05	-2.8247
15021342	---		<0.05	-2.82595
15025944	ENSECAT00000010490 // LOC100063623 // heat shock 70 kDa protein 4L-like // --- // 10006	LOC100063623	<0.05	-2.82666
15056555	XM_003364248 // LOC100629514 // beta-defensin 33-like // --- // 100629514 /// ENSECAT00	LOC100629514	<0.05	-2.82787
14925713	---		<0.05	-2.8281
15128930	---		<0.05	-2.82934
14976559	---		<0.05	-2.83154
15137768	---		<0.05	-2.83392
15083354	XM_001491416 // LOC100050381 // olfactory receptor 9A4-like // --- // 100050381 /// ENS	LOC100050381	<0.05	-2.83591
15081182	---		<0.05	-2.83618
14951946	XM_003362303 // LOC100630300 // olfactory receptor 10A7-like // --- // 100630300 /// EN	LOC100630300	<0.05	-2.83779
15087145	ENSECAT00000023956 // LOC100065052 // gap junction alpha-5 protein-like // --- // 10006	LOC100065052	<0.05	-2.83819
15059867	---		<0.05	-2.8383
15014855	---		<0.05	-2.83877
14929093	---		<0.05	-2.84187
15020226	---		<0.05	-2.84196
14990082	XM_001499765 // LOC100070088 // cytochrome P450 1B1-like // --- // 100070088 /// ENSECA	LOC100070088	<0.05	-2.84501
15070408	---		<0.05	-2.84512
14943907	---		<0.05	-2.84566
15097352	---		<0.05	-2.84743
15120935	---		<0.05	-2.85027
14929023	---		<0.05	-2.85118
14972858	---		<0.05	-2.85228
15123654	---		<0.05	-2.85285
14929251	---		<0.05	-2.85601
15078440	NM_001114147 // MET // met proto-oncogene (hepatocyte growth factor receptor) // --- //	MET	<0.05	-2.85853
14931443	---		<0.05	-2.85942
14931567	---		<0.05	-2.85995
15092613	ENSECAT00000017575 // VCAM // vascular cell adhesion molecule // --- // 100050252 /// N	VCAM	<0.05	-2.86015
15006666	---		<0.05	-2.86154
14931155	---		<0.05	-2.8617
15077032	---		<0.05	-2.86439
15085220	ENSECAT00000023431 // MIR1255B // microRNA mir-1255b // --- // 100314985	MIR1255B	<0.05	-2.86547
15040996	NR_032985 // MIR296 // microRNA mir-296 // --- // 100314887	MIR296	<0.05	-2.8656
14929379	---		<0.05	-2.86745
15008330	---		<0.05	-2.86915
14934829	XM_001502951 // LOC100072886 // urokinase-type plasminogen activator-like // --- // 100	LOC100072886	<0.05	-2.86939
14925155	---		<0.05	-2.87019
15024993	---		<0.05	-2.87039
14932301	---		<0.05	-2.87502
14929989	---		<0.05	-2.87699
15020754	---		<0.05	-2.87727
15054693	---		<0.05	-2.87747
14947745	XM_001502073 // LOC100072195 // olfactory receptor 4K3-like // --- // 100072195 /// ENS	LOC100072195	<0.05	-2.87797
15089458	---		<0.05	-2.88064
14927553	---		<0.05	-2.88265
15093090	XM_001494684 // CLCA4 // chloride channel accessory 4 // --- // 100063474 /// ENSECAT00	CLCA4	<0.05	-2.88329
14929727	---		<0.05	-2.88368
15136376	---		<0.05	-2.88712
15092908	---		<0.05	-2.88935
14972483	ENSECAT00000014769 // C1QTNF4 // C1q and tumor necrosis factor related protein 4 // ---	C1QTNF4	<0.05	-2.89003
14931343	---		<0.05	-2.89019
14973136	---		<0.05	-2.89281
15041693	---		<0.05	-2.89594
14999527	ENSECAT00000010605 // LOC100629337 // sentan-like // --- // 100629337 /// XM_003363081	LOC100629337	<0.05	-2.90213
14925779	---		<0.05	-2.90503
15013425	ENSECAT00000002615 // ABI3BP // ABI family, member 3 (NESH) binding protein // --- // -	ABI3BP	<0.05	-2.90669
15019975	---		<0.05	-2.90786
15120810	---		<0.05	-2.91025
15066246	---		<0.05	-2.91252
15108587	---		<0.05	-2.91441
15043266	---		<0.05	-2.91657
14945124	---		<0.05	-2.9202
15042918	---		<0.05	-2.92753
14926477	---		<0.05	-2.9284
15013022	---		<0.05	-2.92856
14927703	---		<0.05	-2.92943
14945245	---		<0.05	-2.93039
15012931	---		<0.05	-2.93068
14952139	---		<0.05	-2.93093

15125810	---		<0.05	-2.93316
14985258	XM_001501485 // LOC100071660 // homeobox protein CDX-1-like // --- // 100071660 /// ENS	LOC100071660	<0.05	-2.93772
14939531	XM_001502180 // LOC100072282 // olfactory receptor 11G2-like // --- // 100072282 /// EN	LOC100072282	<0.05	-2.93919
14948481	---		<0.05	-2.94006
15088960	---		<0.05	-2.94098
15137324	---		<0.05	-2.94358
14928923	---		<0.05	-2.94481
15101437	---		<0.05	-2.94735
14946662	XM_001501574 // TMOD2 // tropomodulin 2 (neuronal) // --- // 100055095 /// XM_003363503	TMOD2	<0.05	-2.95202
15088154	XM_001492244 // LOC100059756 // protein FAM69A-like // --- // 100059756 /// ENSECAT0000	LOC100059756	<0.05	-2.95439
14940493	---		<0.05	-2.9545
14939302	---		<0.05	-2.95452
14961550	ENSECAT00000003546 // LOC100057520 // vacuolar protein sorting 72 homolog (S. cerevisia	LOC100057520	<0.05	-2.95453
15008512	---		<0.05	-2.95503
14983670	---		<0.05	-2.95626
14945014	NR_032791 // MIR7 // microRNA mir-7 // --- // 100314782	MIR7	<0.05	-2.95709
14931969	---		<0.05	-2.95739
14929463	---		<0.05	-2.95851
15129160	XM_001493412 // LOC100053876 // transcription elongation factor A protein-like 3-like /	LOC100053876	<0.05	-2.9609
15115516	---		<0.05	-2.96538
15067883	---		<0.05	-2.96554
14928247	---		<0.05	-2.96808
14931463	---		<0.05	-2.97398
15054272	ENSECAT00000018858 // PDE9A // phosphodiesterase 9A // --- // 100057783 /// XM_00149100	PDE9A	<0.05	-2.97451
14930329	---		<0.05	-2.97564
14926199	---		<0.05	-2.97598
15100521	ENSECAT00000014627 // CSDA // cold shock domain protein A // --- // ---	CSDA	<0.05	-2.97704
14926421	---		<0.05	-2.98216
15127307	---		<0.05	-2.98425
15080471	---		<0.05	-2.98665
14930307	---		<0.05	-2.98766
15004458	---		<0.05	-2.98783
14954873	---		<0.05	-2.9937
15039650	---		<0.05	-2.99428
15105247	XM_001501860 // LOC100072010 // olfactory receptor 8G5-like // --- // 100072010 /// ENS	LOC100072010	<0.05	-2.99591
14984933	---		<0.05	-2.99594
15026552	---		<0.05	-2.99763
15024823	ENSECAT00000025456 // EGR3 // early growth response 3 // --- // 100057675 /// XM_001492	EGR3	<0.05	-3.00487
15061876	---		<0.05	-3.0056
15049503	---		<0.05	-3.0069
15136718	---		<0.05	-3.00693
14925921	---		<0.05	-3.01048
14926621	---		<0.05	-3.01292
14927577	---		<0.05	-3.01309
15072142	---		<0.05	-3.01448
14929139	---		<0.05	-3.01678
14928313	---		<0.05	-3.01711
14988559	XM_001496740 // LOC100065558 // transcription factor Spi-C-like // --- // 100065558 ///	LOC100065558	<0.05	-3.01906
15134628	---		<0.05	-3.01958
15093895	---		<0.05	-3.02161
15122994	---		<0.05	-3.02179
15093928	---		<0.05	-3.02427
14989646	---		<0.05	-3.02525
14959321	---		<0.05	-3.02571
15123574	---		<0.05	-3.03377
14926091	---		<0.05	-3.03383
14927597	---		<0.05	-3.03434
14928217	---		<0.05	-3.03489
14929197	---		<0.05	-3.03499
15035569	---		<0.05	-3.03527
15028939			<0.05	-3.03529
15136250	---		<0.05	-3.03641
14973124	ENSECAT00000025250 // FADS1 // fatty acid desaturase 1 // --- // ---	FADS1	<0.05	-3.03723
15008504	---		<0.05	-3.03768
15107661	---		<0.05	-3.04163
15003939	---		<0.05	-3.0432
14926759	---		<0.05	-3.04335
14931101	---		<0.05	-3.04488
15100743	---		<0.05	-3.04704
15031201	NM_001163967 // MLN // motilin // --- // 100033882 /// ENSECAT00000014700 // MLN // mot	MLN	<0.05	-3.05022
15054690	---		<0.05	-3.05052
15053755	---		<0.05	-3.05452
14950584	ENSECAT00000014883 // LOC100065532 // apolipoprotein C-II-like // --- // 100065532 ///	LOC100065532	<0.05	-3.0555
15044546	---		<0.05	-3.05552
14954876	NM_001193462 // SULT2A1 // sulfotransferase family, cytosolic, 2A, dehydroepiandrostero	SULT2A1	<0.05	-3.05698
14937199	---		<0.05	-3.05711
15136460	---		<0.05	-3.05778
14970456	XM_001494183 // LOC100062709 // fatty acid desaturase 2-like // --- // 100062709 /// EN	LOC100062709	<0.05	-3.05842
15015464	---		<0.05	-3.05914
15021144	---		<0.05	-3.06511
15137776	---		<0.05	-3.06867
15005589	---		<0.05	-3.06994
14929257	---		<0.05	-3.07067
14926179	---		<0.05	-3.07166
14992806	---		<0.05	-3.07266
14931477	---		<0.05	-3.07521
14987988	---		<0.05	-3.0772
14988835	---		<0.05	-3.0795
15067759	---		<0.05	-3.08019
14967620	ENSECAT00000008888 // C17orf97 // chromosome 17 open reading frame 97 // --- // ---	C17orf97	<0.05	-3.08121
14988354	---		<0.05	-3.08161
15004021	---		<0.05	-3.08214
15137356	---		<0.05	-3.08791
14925349	---		<0.05	-3.09062
15097507	XM_001491477 // LOC100058554 // olfactory receptor 6C4-like // --- // 100058554 /// ENS	LOC100058554	<0.05	-3.09228
15123719	---		<0.05	-3.09266
14936522	---		<0.05	-3.09518
15003725	---		<0.05	-3.09537
15136610	---		<0.05	-3.09606
15049022	---		<0.05	-3.09641
15075231	---		<0.05	-3.09788
15056063	---		<0.05	-3.09926
14952183	---		<0.05	-3.10158
14988606	---		<0.05	-3.10419
14991173	---		<0.05	-3.10533
15137116	---		<0.05	-3.10617

15085669	---		<0.05	-3.10739
15129942	---		<0.05	-3.1082
14990772	---		<0.05	-3.11002
14930397	---		<0.05	-3.11462
14925393	---		<0.05	-3.116
15128758	---		<0.05	-3.1178
15135502	---		<0.05	-3.11852
14925819	---		<0.05	-3.11902
14925849	---		<0.05	-3.11902
14925891	---		<0.05	-3.11902
14925963	---		<0.05	-3.11902
15099118	---		<0.05	-3.12296
15107545	---		<0.05	-3.12476
14941326	---		<0.05	-3.12659
15069482	---		<0.05	-3.12753
15027283	ENSECAT00000006780 // ZNF187 // zinc finger protein 187 // --- // 100061664 /// XM_0014	ZNF187	<0.05	-3.13068
14931141	---		<0.05	-3.13205
15046024	---		<0.05	-3.13687
15048679	---		<0.05	-3.13732
14966652	---		<0.05	-3.13877
15024877	---		<0.05	-3.14002
15073587	---		<0.05	-3.14002
15109355	---		<0.05	-3.14389
15063456	XM_003364358 // LOC100630445 // meiosis expressed gene 1 protein homolog // --- // 1006	LOC100630445	<0.05	-3.14664
14937666	---		<0.05	-3.14925
14929499	---		<0.05	-3.14958
14927773	---		<0.05	-3.15116
15121415	---		<0.05	-3.15556
14946480	---		<0.05	-3.15574
15131496	---		<0.05	-3.1582
15028965			<0.05	-3.16049
14955908	---		<0.05	-3.16182
14930617	---		<0.05	-3.16643
15126566	NM_001166081 // DEFA22 // Paneth cell-specific alpha-defensin 22 // --- // 100306994 //	DEFA22	<0.05	-3.16817
15054811	XM_001498489 // LOC100068667 // SAM domain-containing protein SAMSN-1-like // --- // 10	LOC100068667	<0.05	-3.16948
15076477	---		<0.05	-3.17004
15121223	---		<0.05	-3.17279
15025773	XM_001915633 // GAB1 // GRB2-associated binding protein 1 // --- // 100062924 /// ENSEC	GAB1	<0.05	-3.17976
14926443	---		<0.05	-3.18091
14972566	XM_003362608 // LOC100058728 // olfactory receptor 4S2-like // --- // 100058728 /// ENS	LOC100058728	<0.05	-3.18101
14929429	---		<0.05	-3.18164
15136514	---		<0.05	-3.18626
14925813	---		<0.05	-3.19016
14925843	---		<0.05	-3.19016
14925885	---		<0.05	-3.19016
14925957	---		<0.05	-3.19016
14984276	---		<0.05	-3.19054
15121846	---		<0.05	-3.19377
14945336	---		<0.05	-3.19458
15133392	---		<0.05	-3.19605
15070500	---		<0.05	-3.19664
15088028	---		<0.05	-3.19931
15137014	---		<0.05	-3.20102
15085022	ENSECAT00000019326 // LOC100052032 // uncharacterized protein C1orf21-like // --- // 10	LOC100052032	<0.05	-3.20145
15019729	---		<0.05	-3.20549
14943721	---		<0.05	-3.20739
15137222	---		<0.05	-3.21448
15071995	---		<0.05	-3.21448
15059089	---		<0.05	-3.21537
14931393	---		<0.05	-3.21558
15028943			<0.05	-3.21781
14929563	---		<0.05	-3.223
14993190	---		<0.05	-3.22346
14927665	---		<0.05	-3.22439
15137638	---		<0.05	-3.22898
15078788	XM_001501658 // LOC100071801 // tetraspanin-33-like // --- // 100071801 /// ENSECAT0000	LOC100071801	<0.05	-3.22988
14927313	---		<0.05	-3.23167
15108637	---		<0.05	-3.24019
15096376	ENSECAT00000010436 // ARNTL2 // aryl hydrocarbon receptor nuclear translocator-like 2 /	ARNTL2	<0.05	-3.24092
15128536	---		<0.05	-3.25083
15055717	---		<0.05	-3.25465
15082491	ENSECAT00000008802 // LOC100071408 // protein FAM3C-like // --- // 100071408 /// XM_001	LOC100071408	<0.05	-3.2551
14993035	---		<0.05	-3.25939
15019850	---		<0.05	-3.2602
15079472	---		<0.05	-3.26159
14927691	---		<0.05	-3.26211
14984817	---		<0.05	-3.2642
14949731	---		<0.05	-3.26431
15064133	---		<0.05	-3.26551
14926739	---		<0.05	-3.26665
15137232	---		<0.05	-3.27384
14958187	XM_001490118 // LOC100056324 // neuronal pentraxin-1-like // --- // 100056324 /// ENSEC	LOC100056324	<0.05	-3.27796
15035567	---		<0.05	-3.2846
15046196	---		<0.05	-3.28621
15010098	---		<0.05	-3.29045
14976229	ENSECAT00000009720 // MIR1255B // microRNA mir-1255b // --- // 100314985	MIR1255B	<0.05	-3.29412
14953829	---		<0.05	-3.29556
15078610	---		<0.05	-3.29819
15127773	ENSECAT00000014937 // TIMP1 // TIMP metallopeptidase inhibitor 1 // --- // 100034220 //	TIMP1	<0.05	-3.3024
14972205	---		<0.05	-3.30294
15136974	---		<0.05	-3.30964
15054753	---		<0.05	-3.30973
15136716	---		<0.05	-3.30973
15126653	NM_001082526 // IL1B // interleukin 1, beta // --- // 100034237 /// ENSECAT00000000066	IL1B	<0.05	-3.31016
14934915	---		<0.05	-3.31481
15136522	---		<0.05	-3.31806
14931545	---		<0.05	-3.31988
15121374	---		<0.05	-3.32408
15131923	---		<0.05	-3.32539
14994855	---		<0.05	-3.32577
15004307	---		<0.05	-3.32649
14931417	---		<0.05	-3.32658
14931619	---		<0.05	-3.33319
15033651	---		<0.05	-3.33392
15035558	---		<0.05	-3.33576
14926399	---		<0.05	-3.33643

14926707	---		<0.05	-3.34596
15128812	---		<0.05	-3.34604
14928029	---		<0.05	-3.34654
14984072	---		<0.05	-3.34669
15069793	---		<0.05	-3.34801
15126657			<0.05	-3.35346
15130776	---		<0.05	-3.35536
15110169	XM_001501632 // LOC100071779 // transmembrane protein 225-like // --- // 100071779 ///	LOC100071779	<0.05	-3.35798
15133192	---		<0.05	-3.36031
15094343	---		<0.05	-3.36354
14928947	---		<0.05	-3.36808
15126569			<0.05	-3.37028
15128843	---		<0.05	-3.37046
14926365	---		<0.05	-3.37069
15095792	---		<0.05	-3.37145
15116076	---		<0.05	-3.37308
15054861	---		<0.05	-3.37352
14928995	---		<0.05	-3.37426
15062886	XM_001915060 // LOC100070887 // olfactory receptor 2L3-like // --- // 100070887 /// ENS	LOC100070887	<0.05	-3.37667
15107327	---		<0.05	-3.38216
14931555	---		<0.05	-3.38307
15054867	---		<0.05	-3.3833
14929345	---		<0.05	-3.3839
15043738	XM_001495466 // LOC100064612 // interferon alpha-14-like // --- // 100064612 /// XM_001	LOC100064612	<0.05	-3.3855
14927205	---		<0.05	-3.39141
15136402	---		<0.05	-3.39637
14994760	---		<0.05	-3.39653
14929247	---		<0.05	-3.40085
14926611	---		<0.05	-3.40423
15080983	---		<0.05	-3.40786
15136388	---		<0.05	-3.40914
15113406	NM_001081897 // ADORA2A // adenosine A2a receptor // --- // 100034039 /// ENSECAT000000	ADORA2A	<0.05	-3.41149
14927243	---		<0.05	-3.41168
14927537	---		<0.05	-3.4261
15136300	---		<0.05	-3.42642
15130216	ENSECAT00000008829 // LOC100147620 // uncharacterized LOC100147620 // --- // 100147620	LOC100147620	<0.05	-3.42684
15066978	---		<0.05	-3.42702
15112574	ENSECAT00000008816 // LOC100071044 // achaete-scute homolog 3-like // --- // 100071044	LOC100071044	<0.05	-3.42842
14931307	---		<0.05	-3.43446
15029534	---		<0.05	-3.43647
15038258	ENSECAT00000014089 // MMP9 // matrix metallopeptidase 9 (gelatinase B, 92kDa gelatinase	MMP9	<0.05	-3.43892
14968552	---		<0.05	-3.43963
15108199	---		<0.05	-3.44022
14956234	---		<0.05	-3.44242
15080887	AY752932 // IGFBP-3 // insulin-like growth factor binding protein-3 // --- // 100034155	IGFBP-3	<0.05	-3.4431
14939905	---		<0.05	-3.44366
15136214	---		<0.05	-3.44415
14947607	---		<0.05	-3.44663
15063753	---		<0.05	-3.45013
15001600	---		<0.05	-3.45207
15097189	---		<0.05	-3.45297
15133078	XM_001491814 // LOC100058519 // RING finger protein 113A-like // --- // 100058519 /// E	LOC100058519	<0.05	-3.46212
15007879	---		<0.05	-3.46517
14981739	---		<0.05	-3.46795
15126658			<0.05	-3.46843
15071082	---		<0.05	-3.47044
14956667	---		<0.05	-3.47157
14957143	ENSECAT00000020501 // LAMA4 // laminin, alpha 4 // --- // 100066875	LAMA4	<0.05	-3.47189
15129823	---		<0.05	-3.47435
15011592	---		<0.05	-3.47553
14927497	---		<0.05	-3.47579
15136198	---		<0.05	-3.47608
15060308	ENSECAT00000022648 // FGD6 // FYVE, RhoGEF and PH domain containing 6 // --- // 1000651	FGD6	<0.05	-3.47951
14926543	---		<0.05	-3.48049
15121336	---		<0.05	-3.48436
15095862	ENSECAT00000026701 // LOC100053371 // solute carrier family 2, facilitated glucose tran	LOC100053371	<0.05	-3.48466
14929301	---		<0.05	-3.49072
15011672	---		<0.05	-3.494
14949318	---		<0.05	-3.49556
15121204	---		<0.05	-3.49595
15137128	---		<0.05	-3.49814
14959243	---		<0.05	-3.50216
15111704	---		<0.05	-3.50256
15057844	---		<0.05	-3.50406
15126655			<0.05	-3.50934
15088483	---		<0.05	-3.51123
14965220	ENSECAT00000016191 // C17orf57 // chromosome 17 open reading frame 57 // --- // ---	C17orf57	<0.05	-3.51209
14995832	---		<0.05	-3.51261
14952659	---		<0.05	-3.51274
15025152	---		<0.05	-3.51483
15124133	---		<0.05	-3.51496
14955480	---		<0.05	-3.51821
15063273	---		<0.05	-3.51858
15137534	---		<0.05	-3.51988
15075276	---		<0.05	-3.52059
15026423	---		<0.05	-3.52424
15137122	---		<0.05	-3.52761
15131197	---		<0.05	-3.53014
15048002	XM_001495656 // LOC100064891 // interferon alpha-inducible protein 27-like protein 2-li	LOC100064891	<0.05	-3.53681
14998181	---		<0.05	-3.54678
15071513	---		<0.05	-3.54963
15137484	---		<0.05	-3.55252
14931251	---		<0.05	-3.5536
14997253	---		<0.05	-3.55811
15077404	---		<0.05	-3.56239
14930211	---		<0.05	-3.56257
15075643	---		<0.05	-3.56714
14986789	---		<0.05	-3.57163
15119622	---		<0.05	-3.57185
15041468	---		<0.05	-3.57291
15117328	---		<0.05	-3.57349
15131850	---		<0.05	-3.57443
15137492	---		<0.05	-3.57569
15072198	---		<0.05	-3.57827
14940832	---		<0.05	-3.57894
15013980	---		<0.05	-3.57896

14995104	ENSECAT00000015237 // PRRT3 // proline-rich transmembrane protein 3 // --- // 100058227	PRRT3	<0.05	-3.58019
14939983	---		<0.05	-3.58128
15056488	---		<0.05	-3.59349
15136946	---		<0.05	-3.59355
14978993	---		<0.05	-3.59483
15100585	ENSECAT00000017390 // DUSP16 // dual specificity phosphatase 16 // --- // ---	DUSP16	<0.05	-3.60302
15050700	---		<0.05	-3.60364
15137272	---		<0.05	-3.60386
15099126	---		<0.05	-3.60392
15021096	---		<0.05	-3.6067
15122109	---		<0.05	-3.61082
15121606	---		<0.05	-3.6116
15136594	---		<0.05	-3.61469
15095985	---		<0.05	-3.61817
15036528	---		<0.05	-3.62343
15051378	---		<0.05	-3.62357
15043742	NM_001114537 // IFN-ALPHA2 // interferon-alpha-2 // --- // 100052818 /// ENSECAT0000000	IFN-ALPHA2	<0.05	-3.62698
14925143	---		<0.05	-3.63126
15003886	---		<0.05	-3.6336
14934259	---		<0.05	-3.63626
14986735	---		<0.05	-3.64197
14986584	---		<0.05	-3.64336
15128893	---		<0.05	-3.64338
15129144	ENSECAT00000001318 // LOC100060576 // protein ARMCX6-like // --- // 100060576 /// XM_00	LOC100060576	<0.05	-3.64347
15110203	XM_001501870 // LOC100072019 // olfactory receptor 8B3-like // --- // 100072019 /// ENS	LOC100072019	<0.05	-3.64371
15092562	---		<0.05	-3.64823
15128156	---		<0.05	-3.65032
14927249	---		<0.05	-3.65364
15127309	---		<0.05	-3.66238
15092377	ENSECAT00000026303 // LOC100058329 // glutathione S-transferase Mu 1-like // --- // 100	LOC100058329	<0.05	-3.66265
15039069	---		<0.05	-3.67317
14972172	---		<0.05	-3.67476
14953796	---		<0.05	-3.67574
15000297	ENSECAT00000008682 // SEMA3F // sema domain, immunoglobulin domain (Ig), short basic do	SEMA3F	<0.05	-3.6789
15086111	NR_032849 // MIR9A // microRNA mir-9a // --- // 100314814	MIR9A	<0.05	-3.6809
14929385	---		<0.05	-3.683
14994675	---		<0.05	-3.68302
15133574	---		<0.05	-3.68786
15125687	---		<0.05	-3.68863
14929261	---		<0.05	-3.69199
15088751	---		<0.05	-3.69297
14939928	---		<0.05	-3.6938
14931553	---		<0.05	-3.69495
15128859	---		<0.05	-3.69785
14925917	---		<0.05	-3.70516
15013547	---		<0.05	-3.70554
14931409	---		<0.05	-3.70615
15112423	XM_001917853 // LOC100147393 // olfactory receptor 2D2-like // --- // 100147393 /// ENS	LOC100147393	<0.05	-3.70695
15123299	---		<0.05	-3.70714
15132024	---		<0.05	-3.70923
14930979	---		<0.05	-3.71068
15137552	---		<0.05	-3.71257
15131817	---		<0.05	-3.71279
14925017	---		<0.05	-3.71781
14925551	---		<0.05	-3.72291
14930555	---		<0.05	-3.72291
15116146	---		<0.05	-3.72658
14978877	---		<0.05	-3.72893
15042280	ENSECAT00000003915 // LOC100052921 // interferon-alpha-4 // --- // 100052921	LOC100052921	<0.05	-3.7306
15045044	---		<0.05	-3.73311
14931303	---		<0.05	-3.73645
15002306	---		<0.05	-3.7442
15008883	---		<0.05	-3.74675
15136744	---		<0.05	-3.74785
15120887	XM_001491115 // ZNF704 // zinc finger protein 704 // --- // 100057981 /// ENSECAT000000	ZNF704	<0.05	-3.74862
15137024	---		<0.05	-3.75097
14926785	---		<0.05	-3.75354
15121968	---		<0.05	-3.75649
15129944	---		<0.05	-3.76219
15070503	---		<0.05	-3.76908
15014322	---		<0.05	-3.76951
15077216	---		<0.05	-3.7697
15136888	---		<0.05	-3.7697
15035255	---		<0.05	-3.77471
15080146	---		<0.05	-3.77839
15004546	---		<0.05	-3.77969
14929187	---		<0.05	-3.78482
15085001	---		<0.05	-3.78878
14997560	---		<0.05	-3.78958
15088038	XM_001490827 // LOC100058159 // calponin-3-like // --- // 100058159 /// ENSECAT00000021	LOC100058159	<0.05	-3.79526
15137292	---		<0.05	-3.79576
15081631	---		<0.05	-3.79646
14926185	---		<0.05	-3.79855
14928979	---		<0.05	-3.80392
15042909	---		<0.05	-3.80417
15035797	---		<0.05	-3.8056
15126568	NM_001166082 // DEFA23 // Paneth cell-specific alpha-defensin 23 // --- // 100306995 //	DEFA23	<0.05	-3.80625
15042175	---		<0.05	-3.80836
15080592	---		<0.05	-3.81437
14982055	---		<0.05	-3.81531
15099877	---		<0.05	-3.8262
15036665	---		<0.05	-3.82629
15005580	---		<0.05	-3.82742
15137030	---		<0.05	-3.82993
15120342	---		<0.05	-3.83093
14997530	---		<0.05	-3.83105
14929775	---		<0.05	-3.83329
15136764	---		<0.05	-3.83545
14931453	---		<0.05	-3.83779
15137210	---		<0.05	-3.84064
15076505	---		<0.05	-3.84546
15027352	XM_001491116 // LOC100057985 // olfactory receptor 2B2-like // --- // 100057985 /// ENS	LOC100057985	<0.05	-3.84678
15136448	---		<0.05	-3.84703
15066169	---		<0.05	-3.84718
15070410	---		<0.05	-3.84783
15027007	---		<0.05	-3.84815

15080135	---		<0.05	-3.8485
15123698	---		<0.05	-3.85143
15127708	---		<0.05	-3.85587
15136636	---		<0.05	-3.86036
14929123	---		<0.05	-3.86347
14926577	---		<0.05	-3.86794
14939854	---		<0.05	-3.87086
14941433	---		<0.05	-3.87819
15136972	---		<0.05	-3.87929
14939278	---		<0.05	-3.88008
15136382	---		<0.05	-3.88679
15137518	---		<0.05	-3.89693
15077850	---		<0.05	-3.89957
15081053	---		<0.05	-3.90036
14942985	---		<0.05	-3.9044
15104339	---		<0.05	-3.90609
15085246	XM_001488889 // LOC100053960 // basic leucine zipper transcriptional factor ATF-like 3-	LOC100053960	<0.05	-3.90641
15136648	---		<0.05	-3.90668
15102523	ENSECAT00000011459 // LOC100051089 // olfactory receptor 6C4-like // --- // 100051089 /	LOC100051089	<0.05	-3.9081
15119210	ENSECAT00000011590 // TMEM200C // transmembrane protein 200C // --- // ---	TMEM200C	<0.05	-3.91143
15119740	ENSECAT00000006598 // ZNF521 // zinc finger protein 521 // --- // 100063937 /// XM_0014	ZNF521	<0.05	-3.91463
15020740	---		<0.05	-3.91875
14956426	---		<0.05	-3.91946
15121507	---		<0.05	-3.92695
15120563	---		<0.05	-3.92746
14931089	---		<0.05	-3.92945
14972768	---		<0.05	-3.93131
14935368	---		<0.05	-3.93359
14952887	XM_001502677 // DSE // dermatan sulfate epimerase // --- // 100072666 /// ENSECAT000000	DSE	<0.05	-3.93468
15131935	---		<0.05	-3.93632
14931377	---		<0.05	-3.93708
14956283	---		<0.05	-3.93907
15137020	---		<0.05	-3.93908
14927191	---		<0.05	-3.94116
15048660	---		<0.05	-3.95001
14927245	---		<0.05	-3.95162
14953572	---		<0.05	-3.95849
15137602	---		<0.05	-3.95849
14931341	---		<0.05	-3.96256
15127526	---		<0.05	-3.9631
15137504	---		<0.05	-3.96393
14970224	XM_001493378 // LOC100061443 // membrane-spanning 4-domains subfamily A member 7-like /	LOC100061443	<0.05	-3.96487
14952175	---		<0.05	-3.96564
15064478	---		<0.05	-3.96691
15137704	---		<0.05	-3.97516
15120491	---		<0.05	-3.9789
14984936	---		<0.05	-3.97909
14988575	---		<0.05	-3.98533
14928927	---		<0.05	-3.98927
14927531	---		<0.05	-3.99901
14928937	---		<0.05	-4.00209
15136194	---		<0.05	-4.00748
15077385	---		<0.05	-4.00884
15008823	---		<0.05	-4.00965
15046104	---		<0.05	-4.01159
14935793	---		<0.05	-4.01269
15039350	---		<0.05	-4.01755
15061962	---		<0.05	-4.02415
15030475	XM_001491016 // LOC100055430 // radiation-inducible immediate-early gene IEX-1-like //	LOC100055430	<0.05	-4.03442
15136542	---		<0.05	-4.03717
14955446	---		<0.05	-4.0377
15028957			<0.05	-4.03861
14932293	---		<0.05	-4.04053
15136266	---		<0.05	-4.04257
15122097	---		<0.05	-4.04729
15083053	---		<0.05	-4.04829
14930415	---		<0.05	-4.0532
15137788	---		<0.05	-4.05761
15058238	---		<0.05	-4.05975
15046136	NR_033035 // MIR494 // microRNA mir-494 // --- // 100314915	MIR494	<0.05	-4.06032
15112046	ENSECAT00000010476 // IL18BP // interleukin 18 binding protein // --- // ---	IL18BP	<0.05	-4.0643
14944152	---		<0.05	-4.06446
15053717	---		<0.05	-4.0714
14952388	---		<0.05	-4.07195
14930459	---		<0.05	-4.07774
15136866	---		<0.05	-4.08715
15014998	---		<0.05	-4.08757
15063620	---		<0.05	-4.09083
15052916	NM_001081935 // PTGES // prostaglandin E synthase // --- // 100034143 /// ENSECAT000000	PTGES	<0.05	-4.09716
15035455	NM_001081811 // FST // follistatin // --- // 100033825 /// ENSECAT00000019030 // FST //	FST	<0.05	-4.09828
15003720	---		<0.05	-4.10276
15137412	---		<0.05	-4.10619
14956148	---		<0.05	-4.1094
15132723	---		<0.05	-4.12227
14970502	---		<0.05	-4.12666
15107842	XM_001501248 // PARVA // parvin, alpha // --- // 100071481 /// ENSECAT00000006448 // PA	PARVA	<0.05	-4.12741
14988786	---		<0.05	-4.12763
14937744	---		<0.05	-4.12803
15048278	XM_001490956 // LOC100055201 // creatine kinase B-type-like // --- // 100055201 /// ENS	LOC100055201	<0.05	-4.12949
14945516	---		<0.05	-4.13638
14928001	---		<0.05	-4.14284
15137218	---		<0.05	-4.14319
15056194	---		<0.05	-4.1505
15071075	AF053497 // CXCL1 // chemokine (C-X-C motif) ligand 1 (melanoma growth stimulating acti	CXCL1	<0.05	-4.15208
14986323	---		<0.05	-4.15927
14925437	---		<0.05	-4.1595
15137178	---		<0.05	-4.16197
14966873	XM_001503888 // LOC100057859 // c-C motif chemokine 4-like // --- // 100057859 /// ENSE	LOC100057859	<0.05	-4.16363
15068034	XM_001498741 // LOC100068923 // fibroblast growth factor-binding protein 1-like // ---	LOC100068923	<0.05	-4.16493
15036481	---		<0.05	-4.16686
15137690	---		<0.05	-4.1669
14990285	---		<0.05	-4.18148
14940553	---		<0.05	-4.18533
15073271	---		<0.05	-4.18933
15131806	---		<0.05	-4.18984
15129810	---		<0.05	-4.19401

15137334	---		<0.05	-4.19401
15076887	---		<0.05	-4.20215
14989359	---		<0.05	-4.20386
14940367	NM_001081881 // GZMB // granzyme B (granzyme 2, cytotoxic T-lymphocyte-associated serin	GZMB	<0.05	-4.20873
15137212	---		<0.05	-4.21335
14984285	---		<0.05	-4.21444
14927521	---		<0.05	-4.21587
15137454	---		<0.05	-4.22071
15062256	---		<0.05	-4.22496
14998897	---		<0.05	-4.22674
15001444	---		<0.05	-4.22738
14931459	---		<0.05	-4.2293
15026967	---		<0.05	-4.2298
15121477	---		<0.05	-4.23544
14991419	---		<0.05	-4.23867
15111672	---		<0.05	-4.24566
15029936	---		<0.05	-4.24611
15136190	---		<0.05	-4.25044
15076437	---		<0.05	-4.25124
15089793	---		<0.05	-4.25182
15055911	---		<0.05	-4.25734
14992068	---		<0.05	-4.2574
15026564	---		<0.05	-4.274
14970132	XM_001497902 // LOC100067995 // olfactory receptor 1020-like // --- // 100067995 /// EN	LOC100067995	<0.05	-4.276
14927523	---		<0.05	-4.28567
14945686	---		<0.05	-4.28954
14939552	---		<0.05	-4.28965
14954457	NM_001163881 // PLAUR // plasminogen activator, urokinase receptor // --- // 100033904	PLAUR	<0.05	-4.28993
14988570	---		<0.05	-4.29126
15024953	NM_001081944 // CLU // clusterin // --- // 100034172 /// ENSECAT00000007460 // CLU // c	CLU	<0.05	-4.30148
14992854	---		<0.05	-4.30794
15051462	---		<0.05	-4.31051
15011423	---		<0.05	-4.31769
15056886	---		<0.05	-4.31899
14925113	---		<0.05	-4.31987
14990270	---		<0.05	-4.32091
15126656			<0.05	-4.32445
14998430	---		<0.05	-4.34119
15137188	---		<0.05	-4.34453
14931753	---		<0.05	-4.35228
15056500	XM_001493010 // LOC100060890 // vascular endothelial growth factor C-like // --- // 100	LOC100060890	<0.05	-4.37015
14994411	---		<0.05	-4.37099
15007899	ENSECAT00000019906 // MYO1B // myosin IB // --- // 100054744	MYO1B	<0.05	-4.37723
15056893	---		<0.05	-4.37891
15136834	---		<0.05	-4.38147
14927443	---		<0.05	-4.38152
14927567	---		<0.05	-4.38416
14994605	XM_001503611 // LOC100057240 // DNA-binding protein inhibitor ID-2-like // --- // 10005	LOC100057240	<0.05	-4.39176
14928045	---		<0.05	-4.39589
15117492	---		<0.05	-4.3959
14927613	---		<0.05	-4.39679
15066673	---		<0.05	-4.39876
15119796	---		<0.05	-4.40448
15057642	---		<0.05	-4.41161
15033990	---		<0.05	-4.41256
15013467	---		<0.05	-4.42548
15136692	---		<0.05	-4.42549
14927515	---		<0.05	-4.42571
15137246	---		<0.05	-4.42656
15099667	---		<0.05	-4.42803
15066109	---		<0.05	-4.43041
14932541	---		<0.05	-4.436
15137748	---		<0.05	-4.4374
14942983	---		<0.05	-4.43819
15042650	---		<0.05	-4.44487
14928065	---		<0.05	-4.45231
15105576	---		<0.05	-4.45459
15011492	---		<0.05	-4.45522
15128897	---		<0.05	-4.45868
14956178	---		<0.05	-4.45934
15132756	---		<0.05	-4.46079
14957391	---		<0.05	-4.4665
15137630	---		<0.05	-4.4665
15030959	ENSECAT00000017521 // C6orf10 // chromosome 6 open reading frame 10 // --- // ---	C6orf10	<0.05	-4.47101
15002919	---		<0.05	-4.47488
15003751	---		<0.05	-4.48663
15077408	---		<0.05	-4.49074
15028951			<0.05	-4.49283
15005441	---		<0.05	-4.49472
14953798	---		<0.05	-4.50007
15073176	XM_001488715 // LBR // lamin B receptor // --- // 100056997 /// ENSECAT00000011831 // L	LBR	<0.05	-4.50411
14959177	---		<0.05	-4.50961
14987159	---		<0.05	-4.51084
15008619	---		<0.05	-4.51229
14998512	ENSECAT00000008123 // MED12L // mediator complex subunit 12-like // --- // 100056881 //	MED12L	<0.05	-4.51265
15136590	---		<0.05	-4.51285
15105959	---		<0.05	-4.52311
15129982	---		<0.05	-4.52772
14927507	---		<0.05	-4.52855
15059632	---		<0.05	-4.52863
15075351	---		<0.05	-4.53691
15006329	---		<0.05	-4.53932
15124232	---		<0.05	-4.55372
15136806	---		<0.05	-4.5538
15004607	ENSECAT00000023490 // FLT1 // fms-related tyrosine kinase 1 (vascular endothelial growt	FLT1	<0.05	-4.56243
14945400	---		<0.05	-4.56591
15020483	---		<0.05	-4.56753
15137696	---		<0.05	-4.57697
14945210	---		<0.05	-4.57799
15103922	---		<0.05	-4.58088
14926941	---		<0.05	-4.58235
15032457	---		<0.05	-4.59069
15136582	---		<0.05	-4.59069
15110136	XM_001501513 // CLMP // CXADR-like membrane protein // --- // 100071682 /// ENSECAT0000	CLMP	<0.05	-4.59598
15005713	---		<0.05	-4.59903
14973179	---		<0.05	-4.59948

14969948	---		<0.05	-4.61301
14927527	---		<0.05	-4.61364
15056485	---		<0.05	-4.6254
14926253	---		<0.05	-4.63524
15067037	---		<0.05	-4.64013
15013644	---		<0.05	-4.64015
15008048	---		<0.05	-4.64453
15115932	---		<0.05	-4.64933
14925487	---		<0.05	-4.65257
15122273	---		<0.05	-4.65423
14927499	---		<0.05	-4.65727
15048970	---		<0.05	-4.65865
15136684	---		<0.05	-4.65865
15053786	---		<0.05	-4.66343
15098543	ENSECAT00000015236 // TNP1 // transition protein 1 (during histone to protamine replace	TNP1	<0.05	-4.69416
15100755	---		<0.05	-4.69607
14932285	---		<0.05	-4.70084
15137692	---		<0.05	-4.70966
14986414	---		<0.05	-4.71068
15137714	---		<0.05	-4.71365
15013649	---		<0.05	-4.72129
15131804	---		<0.05	-4.72816
14945265	---		<0.05	-4.72845
15070406	---		<0.05	-4.73068
14925125	---		<0.05	-4.73134
15062489	XM_001498585 // LOC100056470 // uncharacterized LOC100056470 // --- // 100056470 /// EN	LOC100056470	<0.05	-4.73308
15137634	---		<0.05	-4.73519
15137194	---		<0.05	-4.73828
15020907	ENSECAT00000011304 // LOC100072294 // TNFAIP3-interacting protein 3-like // --- // 1000	LOC100072294	<0.05	-4.74042
15123622	---		<0.05	-4.7482
15035987	XM_001501124 // LOC100069914 // brain acid soluble protein 1-like // --- // 100069914 /	LOC100069914	<0.05	-4.74859
14994319	---		<0.05	-4.75394
15137294	---		<0.05	-4.75473
15130284	---		<0.05	-4.76081
14992029	---		<0.05	-4.76299
15056775	---		<0.05	-4.77017
14935721	---		<0.05	-4.77214
14933059	---		<0.05	-4.77644
14926219	---		<0.05	-4.78525
15019731	---		<0.05	-4.7927
15128814	---		<0.05	-4.79381
14928955	---		<0.05	-4.80113
15136236	---		<0.05	-4.80498
14948376	XM_001489335 // LOC100051088 // dehydrogenase/reductase SDR family member 1-like // ---	LOC100051088	<0.05	-4.80646
14940573	---		<0.05	-4.81395
15091917	---		<0.05	-4.82222
15118658			<0.05	-4.82496
15046034	---		<0.05	-4.83934
14930905	---		<0.05	-4.84315
14989037	---		<0.05	-4.84845
15004737	---		<0.05	-4.85358
14931221	---		<0.05	-4.85634
15028949			<0.05	-4.86139
15126669	---		<0.05	-4.86198
15126670			<0.05	-4.86198
15041530	---		<0.05	-4.86365
14927917	---		<0.05	-4.8666
15133149	---		<0.05	-4.87629
14983339	---		<0.05	-4.87687
14992346	---		<0.05	-4.88167
15080015	---		<0.05	-4.88217
15077271	---		<0.05	-4.88297
15084659	---		<0.05	-4.88912
15076165	---		<0.05	-4.90513
14940656	---		<0.05	-4.9054
15021256	---		<0.05	-4.90741
15135584	---		<0.05	-4.91171
15057170	---		<0.05	-4.91692
14986495	---		<0.05	-4.91789
15078434	NM_001114143 // CAV1 // caveolin 1, caveolae protein, 22kDa // --- // 100055975 /// ENS	CAV1	<0.05	-4.92125
14974450	---		<0.05	-4.92366
15034400	---		<0.05	-4.92386
15123769	---		<0.05	-4.92611
14941508	---		<0.05	-4.93098
14930301	---		<0.05	-4.93957
14970514	---		<0.05	-4.94845
15136732	---		<0.05	-4.95136
15012315	---		<0.05	-4.95209
14959489	---		<0.05	-4.95447
14943913	---		<0.05	-4.96362
14967860	XM_001918357 // LOC100072577 // olfactory receptor 1A1-like // --- // 100072577 /// ENS	LOC100072577	<0.05	-4.9667
15029663	---		<0.05	-4.96793
15137352	---		<0.05	-4.98302
14952258	---		<0.05	-4.98346
14929067	---		<0.05	-4.98486
14929503	---		<0.05	-4.98486
14963451	---		<0.05	-4.98502
15001979	---		<0.05	-4.99173
15131335	---		<0.05	-4.99339
15111670	---		<0.05	-4.99339
15137112	---		<0.05	-4.99339
15027133	ENSECAT00000006961 // LOC100053545 // histone H2A type 1-C-like // --- // 100053545	LOC100053545	<0.05	-4.99618
15008655	---		<0.05	-5.00189
15078051	---		<0.05	-5.00261
14982991	---		<0.05	-5.00913
15132687	ENSECAT00000013894 // LOC100067023 // transcription elongation factor A protein-like 4-	LOC100067023	<0.05	-5.01542
15066864	ENSECAT00000020789 // LOC100055761 // ras association domain-containing protein 6-like	LOC100055761	<0.05	-5.01557
15126921	---		<0.05	-5.0161
15136338	---		<0.05	-5.01767
15007935	---		<0.05	-5.03334
15007591	---		<0.05	-5.04086
15004988	---		<0.05	-5.04172
15071285	---		<0.05	-5.05896
15001471	---		<0.05	-5.06403
14947032	---		<0.05	-5.06449
15107741	XM_001504918 // AMPD3 // adenosine monophosphate deaminase 3 // --- // 100055812 /// EN	AMPD3	<0.05	-5.07406

14976733	---		<0.05	-5.07407
15071762	---		<0.05	-5.08674
14973186	---		<0.05	-5.08692
14926807	---		<0.05	-5.08894
15129209	XM_001503086 // LOC100059264 // WW domain-binding protein 5-like // --- // 100059264 //	LOC100059264	<0.05	-5.10243
14931079	---		<0.05	-5.12138
14926407	---		<0.05	-5.13331
14926197	---		<0.05	-5.14633
15003546	---		<0.05	-5.14752
14927519	---		<0.05	-5.16698
14937201	---		<0.05	-5.16733
15002544	---		<0.05	-5.1712
15137050	---		<0.05	-5.17512
15136606	---		<0.05	-5.17889
15014265	---		<0.05	-5.1816
15036423	XM_001488842 // CD93 // CD93 molecule // --- // 100059201 /// ENSECAT00000017144 // CD9	CD93	<0.05	-5.18585
14934337	---		<0.05	-5.19021
15136598	---		<0.05	-5.20474
14946717	---		<0.05	-5.20776
15132690	---		<0.05	-5.20845
15064131	---		<0.05	-5.21672
14990908	---		<0.05	-5.21851
15015212	---		<0.05	-5.22185
15034553	---		<0.05	-5.22202
14931547	---		<0.05	-5.23343
15016373	---		<0.05	-5.23527
14984221	XM_001918276 // LOC100146557 // olfactory receptor 2C3-like // --- // 100146557 /// ENS	LOC100146557	<0.05	-5.23911
15137738	---		<0.05	-5.25668
14925101	---		<0.05	-5.26282
15132104	---		<0.05	-5.27472
14927533	---		<0.05	-5.27555
15003565	---		<0.05	-5.27653
15057838	---		<0.05	-5.30154
15077156	---		<0.05	-5.30297
15123788	---		<0.05	-5.30858
15123677	---		<0.05	-5.30938
14927529	---		<0.05	-5.31406
15074165	---		<0.05	-5.32581
14989504	---		<0.05	-5.32609
14992879	---		<0.05	-5.33609
14945170	XM_001490809 // CHSY1 // chondroitin sulfate synthase 1 // --- // 100057477 /// ENSECAT	CHSY1	<0.05	-5.3364
15002182	---		<0.05	-5.3409
15084927	ENSECAT00000025271 // LOC100055269 // laminin subunit gamma-1-like // --- // 100055269	LOC100055269	<0.05	-5.3545
14948504	---		<0.05	-5.35951
14928893	---		<0.05	-5.36341
14954190	---		<0.05	-5.37368
14927539	---		<0.05	-5.37444
14958861	XM_003362466 // LOC100630791 // CMRF35-like molecule 6-like // --- // 100630791 /// ENS	LOC100630791	<0.05	-5.38815
15120827	---		<0.05	-5.40624
15013559	---		<0.05	-5.4149
14927543	---		<0.05	-5.41745
14928731	---		<0.05	-5.41818
15111694	---		<0.05	-5.42785
15111241	---		<0.05	-5.43123
15033343	---		<0.05	-5.43265
15136952	---		<0.05	-5.43337
15106523	---		<0.05	-5.43953
15116706	---		<0.05	-5.46002
14927541	---		<0.05	-5.4602
15011843	---		<0.05	-5.46751
15110862	ENSECAT00000023383 // LOC100064925 // zinc finger protein 709-like // --- // 100064925	LOC100064925	<0.05	-5.47139
14931329	---		<0.05	-5.48737
15119390	---		<0.05	-5.49763
14927517	---		<0.05	-5.50211
15136378	---		<0.05	-5.50311
15137206	---		<0.05	-5.52509
14930197	---		<0.05	-5.5305
15054822	---		<0.05	-5.54827
15136510	---		<0.05	-5.55211
15137728	---		<0.05	-5.5554
15053550	---		<0.05	-5.56152
15132779	---		<0.05	-5.56277
14926961	---		<0.05	-5.56733
15039371	---		<0.05	-5.57421
15054155	ENSECAT00000012965 // PCP4 // Purkinje cell protein 4 // --- // ---	PCP4	<0.05	-5.58551
14990213	---		<0.05	-5.58805
15136404	---		<0.05	-5.60001
15066261	---		<0.05	-5.61024
15009914	---		<0.05	-5.61078
15136550	---		<0.05	-5.61903
15136626	---		<0.05	-5.6209
15067814	---		<0.05	-5.63314
14931589	---		<0.05	-5.64608
15089212	ENSECAT00000009338 // IL10 // interleukin 10 // --- // 100034187 /// NM_001082490 // IL	IL10	<0.05	-5.65616
15137158	---		<0.05	-5.65915
14999686	---		<0.05	-5.65981
15137548	---		<0.05	-5.66585
14927509	---		<0.05	-5.67097
15130792	---		<0.05	-5.67509
14927535	---		<0.05	-5.67777
15136720	---		<0.05	-5.68619
15094485	---		<0.05	-5.69919
15071328	---		<0.05	-5.7087
14943445	---		<0.05	-5.71058
14968938	---		<0.05	-5.71722
15128871	---		<0.05	-5.71866
15132450	---		<0.05	-5.71866
15137312	---		<0.05	-5.71866
15137400	---		<0.05	-5.71866
14939769	---		<0.05	-5.73396
15026554	---		<0.05	-5.74008
15081698	---		<0.05	-5.74509
14984037	---		<0.05	-5.75227
15136344	---		<0.05	-5.79515
15067585	---		<0.05	-5.80661
15093139	XM_001495078 // CYR61 // cysteine-rich, angiogenic inducer, 61 // --- // 100064066 ///	CYR61	<0.05	-5.80807

15058700	XM_001498399 // CHST11 // carbohydrate (chondroitin 4) sulfotransferase 11 // --- // 10	CHST11	<0.05	-5.82959
15088545	---		<0.05	-5.83102
15079449	---		<0.05	-5.84968
14930185	---		<0.05	-5.85162
15136406	---		<0.05	-5.85401
15134014	---		<0.05	-5.86054
15131126	---		<0.05	-5.86659
15089956	---		<0.05	-5.88124
15136658	---		<0.05	-5.88241
15137786	---		<0.05	-5.88362
15003013	---		<0.05	-5.88365
15081065	---		<0.05	-5.89157
14927595	---		<0.05	-5.89496
14942909	XM_001501590 // PAPSS2 // 3'-phosphoadenosine 5'-phosphosulfate synthase 2 // --- // 10	PAPSS2	<0.05	-5.91165
14986586	---		<0.05	-5.92679
15082556	---		<0.05	-5.92829
14937807	ENSECAT00000026465 // MYO1E // myosin IE // --- // 100068162	MYO1E	<0.05	-5.92866
15043747	NM_001114537 // IFN-ALPHA2 // interferon-alpha-2 // --- // 100052818 /// NM_001114538 /	IFN-ALPHA2	<0.05	-5.93653
14969018	---		<0.05	-5.94463
15121419	---		<0.05	-5.95402
15121600	---		<0.05	-5.96532
14931313	---		<0.05	-5.97878
15066646	ENSECAT00000019611 // ANTXR2 // anthrax toxin receptor 2 // --- // 100051770 /// XM_001	ANTXR2	<0.05	-5.97954
15137648	---		<0.05	-5.98308
14926971	---		<0.05	-5.98355
14978976	---		<0.05	-5.98843
15054765	---		<0.05	-5.99531
15079594	XM_001490685 // LOC100057296 // olfactory receptor-like protein OLF3-like // --- // 100	LOC100057296	<0.05	-6.00117
14969944	---		<0.05	-6.01385
15055909	---		<0.05	-6.01458
14948604	---		<0.05	-6.01637
14940044	XM_001918317 // LOC100056663 // olfactory receptor 6C4-like // --- // 100056663 /// ENS	LOC100056663	<0.05	-6.02666
15137250	---		<0.05	-6.02685
14926243	---		<0.05	-6.04122
14927525	---		<0.05	-6.05089
14993140	---		<0.05	-6.0573
15069817	---		<0.05	-6.06903
15015559	---		<0.05	-6.07401
15029606	XM_001491438 // LOC100058493 // PX domain-containing protein C6orf145-like // --- // 10	LOC100058493	<0.05	-6.0786
14931385	---		<0.05	-6.09084
14992003	NM_001082526 // IL1B // interleukin 1, beta // --- // 100034237 /// ENSECAT00000000066	IL1B	<0.05	-6.09512
14927513	---		<0.05	-6.09991
14954222	---		<0.05	-6.10197
14947112	---		<0.05	-6.13064
15071077	FJ469975 // MIP-2BETA // CXCL3 // --- // 100056258 /// NM_001143793 // MIP-2BETA // CXC	MIP-2BETA	<0.05	-6.14035
15012291	NR_032968 // MIR28 // microRNA mir-28 // --- // 100314878	MIR28	<0.05	-6.14595
15043178	---		<0.05	-6.14681
15136652	---		<0.05	-6.14681
15107872	---		<0.05	-6.16222
15136938	---		<0.05	-6.16914
15005366	---		<0.05	-6.17363
14934233	---		<0.05	-6.17742
15136274	---		<0.05	-6.19053
14939871	---		<0.05	-6.19453
14941961	---		<0.05	-6.20249
15084211	---		<0.05	-6.20669
15136752	---		<0.05	-6.21503
14927549	---		<0.05	-6.22139
15077707	---		<0.05	-6.23102
14928975	---		<0.05	-6.23322
14927511	---		<0.05	-6.23367
14927505	---		<0.05	-6.24232
15032076	XM_001503079 // LOC100056506 // glutathione S-transferase A2-like // --- // 100056506 /	LOC100056506	<0.05	-6.24982
15137172	---		<0.05	-6.25677
15019280	ENSECAT00000021228 // LOC100066167 // tumor necrosis factor receptor superfamily member	LOC100066167	<0.05	-6.26861
15134630	---		<0.05	-6.2859
14927501	---		<0.05	-6.28809
15053638	---		<0.05	-6.28926
15070948	---		<0.05	-6.30507
15110909	---		<0.05	-6.30629
15009366	---		<0.05	-6.31143
15059084	XM_001499732 // LOC100070048 // ER lumen protein retaining receptor 3-like // --- // 10	LOC100070048	<0.05	-6.32099
15064310	ENSECAT00000019145 // LOC100062332 // c-C motif chemokine 22-like // --- // 100062332 /	LOC100062332	<0.05	-6.33783
15132480	---		<0.05	-6.34234
14947540	---		<0.05	-6.34468
14936833	---		<0.05	-6.34575
15109260	---		<0.05	-6.34753
15137008	---		<0.05	-6.36544
15098338	---		<0.05	-6.3853
15098898	---		<0.05	-6.39657
14935733	---		<0.05	-6.40252
15132345	---		<0.05	-6.40439
15013222	---		<0.05	-6.40521
14927547	---		<0.05	-6.41914
15134011	---		<0.05	-6.4204
14988524	ENSECAT00000005750 // IL1RN // interleukin 1 receptor antagonist // --- // 100034236 //	IL1RN	<0.05	-6.43279
15101195	---		<0.05	-6.4395
15137418	---		<0.05	-6.46331
15123246	---		<0.05	-6.46568
15083236	---		<0.05	-6.47524
15023528	---		<0.05	-6.47739
15136984	---		<0.05	-6.49337
15137624	---		<0.05	-6.4972
15070334	---		<0.05	-6.52013
15131897	---		<0.05	-6.52857
15019357	---		<0.05	-6.5655
15137300	---		<0.05	-6.57263
14945404	---		<0.05	-6.57922
15046172	NR_033023 // MIR382 // microRNA mir-382 // --- // 100314908	MIR382	<0.05	-6.58466
14953384	---		<0.05	-6.5895
14926687	---		<0.05	-6.59268
15015538	---		<0.05	-6.60518
14927955	---		<0.05	-6.61888
14927495	---		<0.05	-6.64915
15033330	XM_001497065 // LOC100052185 // endothelial cell-specific molecule 1-like // --- // 100	LOC100052185	<0.05	-6.65185
14991525	ENSECAT00000017645 // IL1R1 // interleukin 1 receptor, type I // --- // 100009699 /// N	IL1R1	<0.05	-6.66965

14956071	---		<0.05	-6.69332
15000429	ENSECAT00000018328 // LOC100053301 // t-cell leukemia translocation-altered gene protei	LOC100053301	<0.05	-6.70959
14931639	---		<0.05	-6.76582
15002374	---		<0.05	-6.78255
15059275	---		<0.05	-6.78748
15046132	NR_033010 // MIR329 // microRNA mir-329 // --- // 100314901	MIR329	<0.05	-6.80665
15137234	---		<0.05	-6.81641
14926189	---		<0.05	-6.81753
15131110	---		<0.05	-6.8634
15136850	---		<0.05	-6.87412
15081449	---		<0.05	-6.88661
14999842	---		<0.05	-6.89445
14956251	---		<0.05	-6.91141
15015570	---		<0.05	-6.91449
15136476	---		<0.05	-6.91449
14991175	---		<0.05	-6.91817
15071287	---		<0.05	-6.92516
15015289	---		<0.05	-6.93287
14927503	---		<0.05	-6.94437
15110894	---		<0.05	-6.95463
15024886	---		<0.05	-6.95559
14981809	---		<0.05	-6.95969
15137196	---		<0.05	-6.96458
14958183	NR_032909 // MIR338 // microRNA mir-338 // --- // 100315012	MIR338	<0.05	-6.97941
14950508	---		<0.05	-6.98681
15120979	---		<0.05	-7.00406
15137650	---		<0.05	-7.01243
14952585	---		<0.05	-7.03471
15001464	---		<0.05	-7.04883
15100783	---		<0.05	-7.04921
15130282	---		<0.05	-7.05112
15115811	---		<0.05	-7.08072
15067167	---		<0.05	-7.08562
15113767	ENSECAT00000015807 // LOC100629131 // leukemia inhibitory factor-like // --- // 1006291	LOC100629131	<0.05	-7.09213
15110229	---		<0.05	-7.0946
15053784	---		<0.05	-7.09474
14927545	---		<0.05	-7.12278
14991995	ENSECAT00000025409 // IL1A // interleukin 1, alpha // --- // 100064969 /// NM_001082500	IL1A	<0.05	-7.12949
14991281	---		<0.05	-7.19739
15130220	---		<0.05	-7.20169
15137354	---		<0.05	-7.20169
15057383	---		<0.05	-7.21014
15003018	---		<0.05	-7.2422
15050950	---		<0.05	-7.2487
15136968	---		<0.05	-7.24962
14962039	---		<0.05	-7.26044
15113120	---		<0.05	-7.26169
15075607	---		<0.05	-7.27229
15011678	---		<0.05	-7.29305
14955890	---		<0.05	-7.29514
14999422	---		<0.05	-7.29968
15136272	---		<0.05	-7.29968
15106565	---		<0.05	-7.32782
15122518	---		<0.05	-7.33957
15060222	NM_001081925 // DCN // decorin // --- // 100034120 /// ENSECAT00000021671 // DCN // dec	DCN	<0.05	-7.34295
14969417	---		<0.05	-7.34531
15012454	---		<0.05	-7.35196
15137568	---		<0.05	-7.36045
15063328	---		<0.05	-7.38668
15059965	---		<0.05	-7.39011
15123449	---		<0.05	-7.41895
15137636	---		<0.05	-7.43107
15055971	---		<0.05	-7.43889
15053647	---		<0.05	-7.44931
15136298	---		<0.05	-7.45222
15109510	---		<0.05	-7.45327
15077203	---		<0.05	-7.46439
15137228	---		<0.05	-7.48093
15041507	---		<0.05	-7.48775
15024317	---		<0.05	-7.48955
15110453	---		<0.05	-7.57422
15026550	---		<0.05	-7.57471
15042542	---		<0.05	-7.578
14992988	---		<0.05	-7.60698
14984066	---		<0.05	-7.62122
14998601	---		<0.05	-7.63192
15045625	---		<0.05	-7.637
15123184	---		<0.05	-7.6411
15028963			<0.05	-7.64736
15073640	---		<0.05	-7.64785
14958185	---		<0.05	-7.66483
15051448	---		<0.05	-7.66551
15020204	---		<0.05	-7.66586
15060076	---		<0.05	-7.67222
15122248	---		<0.05	-7.67403
15104066	---		<0.05	-7.6904
15072312	---		<0.05	-7.6913
14939650	---		<0.05	-7.7137
15129826	---		<0.05	-7.73411
15071089	ENSECAT00000013150 // LOC100055973 // platelet basic protein-like // --- // 100055973 /	LOC100055973	<0.05	-7.75594
15136270	---		<0.05	-7.76139
14952260	---		<0.05	-7.76415
15095844	---		<0.05	-7.77077
15066400	---		<0.05	-7.7779
14934197	---		<0.05	-7.78068
14935083	---		<0.05	-7.78525
15124436	ENSECAT00000022529 // HAS2 // hyaluronan synthase 2 // --- // 100009708 /// NM_00108180	HAS2	<0.05	-7.79228
14985498	---		<0.05	-7.79989
15056890	---		<0.05	-7.81155
15137162	---		<0.05	-7.82752
15121690	---		<0.05	-7.83272
14931609	---		<0.05	-7.84316
15039926	---		<0.05	-7.85776
14949019	---		<0.05	-7.8714
15136432	---		<0.05	-7.88126
15137270	---		<0.05	-7.88633

15007125	---		<0.05	-7.89062
15123694	---		<0.05	-7.89842
15027009	---		<0.05	-7.91625
14945080	---		<0.05	-7.92586
15137042	---		<0.05	-7.928
14969510	---		<0.05	-7.94725
15000214	---		<0.05	-7.96628
15132669	---		<0.05	-7.98623
15094482	---		<0.05	-8.00927
14945078	---		<0.05	-8.05213
14969979	---		<0.05	-8.06564
15076105	XM_001494556 // AKAP12 // A kinase (PRKA) anchor protein 12 // --- // 100063263 /// ENS	AKAP12	<0.05	-8.10626
15121610	---		<0.05	-8.10677
14985496	---		<0.05	-8.11241
14987985	---		<0.05	-8.11648
14933031	---		<0.05	-8.11774
15059859	---		<0.05	-8.1323
14965242	NM_001081802 // ITGB3 // integrin, beta 3 (platelet glycoprotein IIIa, antigen CD61) //	ITGB3	<0.05	-8.13322
14999420	---		<0.05	-8.14828
15085953	---		<0.05	-8.19096
15054869	---		<0.05	-8.1916
15035982	---		<0.05	-8.19331
15136616	---		<0.05	-8.19331
14926331	---		<0.05	-8.20532
15137626	---		<0.05	-8.21972
15062954	---		<0.05	-8.2288
15008758	---		<0.05	-8.25748
15092756	---		<0.05	-8.30135
15110535	---		<0.05	-8.32086
15136996	---		<0.05	-8.32595
15132711	---		<0.05	-8.36814
15137408	---		<0.05	-8.36814
15137010	---		<0.05	-8.37375
14994399	---		<0.05	-8.42077
15039230	---		<0.05	-8.42682
14925355	---		<0.05	-8.42887
15005345	---		<0.05	-8.45847
14927023	---		<0.05	-8.46258
15130872	---		<0.05	-8.46529
15071334	---		<0.05	-8.53064
15051602	---		<0.05	-8.54231
15136772	---		<0.05	-8.54809
15104212	---		<0.05	-8.55211
15128304	---		<0.05	-8.55287
15057842	---		<0.05	-8.56311
15060547	---		<0.05	-8.56675
15136842	---		<0.05	-8.574
15101197	---		<0.05	-8.60064
15062893	---		<0.05	-8.60105
15137278	---		<0.05	-8.61951
15014596	---		<0.05	-8.62483
15115488	---		<0.05	-8.63028
15025986	---		<0.05	-8.6793
15123776	---		<0.05	-8.68474
15013019	---		<0.05	-8.70073
14985150	---		<0.05	-8.71167
15022476	---		<0.05	-8.71329
14974292	---		<0.05	-8.72066
15104279	---		<0.05	-8.7291
15136794	---		<0.05	-8.73802
15057281	---		<0.05	-8.74745
15136350	---		<0.05	-8.7521
15003875	---		<0.05	-8.77973
15136702	---		<0.05	-8.8113
15136904	---		<0.05	-8.81622
15005308	---		<0.05	-8.81889
15048744	---		<0.05	-8.84417
15087957	---		<0.05	-8.85053
15137560	---		<0.05	-8.85836
14954007	ENSECAT00000021129 // LOC100147106 // uncharacterized LOC100147106 // --- // 100147106	LOC100147106	<0.05	-8.86357
15137064	---		<0.05	-8.87788
15137060	---		<0.05	-8.89615
15133231	---		<0.05	-8.92988
15033040	---		<0.05	-8.96715
15008795	---		<0.05	-8.97923
14939930	---		<0.05	-9.0112
15136544	---		<0.05	-9.08944
15130692	---		<0.05	-9.08971
14938040	---		<0.05	-9.09147
15131203	---		<0.05	-9.10315
14950082	---		<0.05	-9.12671
15087535	---		<0.05	-9.15312
15033411	---		<0.05	-9.18649
15060455	---		<0.05	-9.19773
15081530	---		<0.05	-9.22302
15136936	---		<0.05	-9.25399
14934920	---		<0.05	-9.27641
15123515	---		<0.05	-9.29494
14938232	---		<0.05	-9.31958
15126812	---		<0.05	-9.3368
15082470	---		<0.05	-9.33748
14989338	---		<0.05	-9.33968
15115243	---		<0.05	-9.36364
15106374	---		<0.05	-9.42303
15001110	---		<0.05	-9.44207
14942828	---		<0.05	-9.44707
15006694	---		<0.05	-9.4937
15088963	---		<0.05	-9.50891
15080985	---		<0.05	-9.52693
15071325	---		<0.05	-9.52777
15076209	ENSECAT00000023278 // LOC100060503 // uronyl 2-sulfotransferase-like // --- // 10006050	LOC100060503	<0.05	-9.60028
14966664	---		<0.05	-9.62064
15103641	NR_032873 // MIR7-2 // microRNA mir-7-2 // --- // 100315008	MIR7-2	<0.05	-9.67189
15100530	---		<0.05	-9.69646
15123607	---		<0.05	-9.69954
15018734	---		<0.05	-9.70939

15120364	ENSECAT00000018691 // TCF7L2 // transcription factor 7-like 2 (T-cell specific, HMG-box	TCF7L2	<0.05	-9.74285
15062669	NM_001111342 // IL2RA // interleukin 2 receptor, alpha // --- // 100070292 /// ENSECAT0	IL2RA	<0.05	-9.76642
14928021	---		<0.05	-9.77627
15136358	---		<0.05	-9.82317
15034555	---		<0.05	-9.82738
15082266	---		<0.05	-9.83919
15110907	---		<0.05	-9.84167
15009515	---		<0.05	-9.85658
15116897	---		<0.05	-9.85674
14972822	---		<0.05	-9.86893
15136724	---		<0.05	-9.87303
15038502	---		<0.05	-9.88557
15137306	---		<0.05	-9.89187
14949239	---		<0.05	-9.91085
15123696	---		<0.05	-9.94105
15056884	---		<0.05	-9.94743
15076475	---		<0.05	-9.96444
15131878	---		<0.05	-9.97001
14929483	---		<0.05	-9.97779
14990746	---		<0.05	-9.98512
14948616	---		<0.05	-9.9901
15035443	---		<0.05	-10.0111
15091422	---		<0.05	-10.0137
15136932	---		<0.05	-10.0562
14946017	---		<0.05	-10.1085
15021574	---		<0.05	-10.125
15078951	---		<0.05	-10.1259
15119937	---		<0.05	-10.1436
15136726	---		<0.05	-10.1502
15131829	---		<0.05	-10.1701
15136330	---		<0.05	-10.1879
15136396	---		<0.05	-10.1879
15092776	---		<0.05	-10.2483
14925107	---		<0.05	-10.2665
15133248	---		<0.05	-10.2836
15133576	---		<0.05	-10.3176
15094905	NM_001257078 // CXCR7 // chemokine (C-X-C motif) receptor 7 // --- // 100057501 /// ENS	CXCR7	<0.05	-10.324
14986670	---		<0.05	-10.3378
15013214	---		<0.05	-10.3447
14938015	---		<0.05	-10.365
15016292	---		<0.05	-10.3722
15036426	XM_001915341 // LOC100146206 // thrombomodulin-like // --- // 100146206 /// ENSECAT0000	LOC100146206	<0.05	-10.3736
14927123	---		<0.05	-10.3866
14969935	---		<0.05	-10.43
15136988	---		<0.05	-10.4448
14927731	---		<0.05	-10.4651
15136320	---		<0.05	-10.4659
14942998	---		<0.05	-10.4891
15045391	---		<0.05	-10.5134
14937067	---		<0.05	-10.5778
14976461	---		<0.05	-10.5793
15137284	---		<0.05	-10.614
14927203	---		<0.05	-10.843
15006532	---		<0.05	-10.9163
15071344	---		<0.05	-10.9631
15020143	---		<0.05	-10.9861
15003586	---		<0.05	-11.0115
15030212	XM_001505026 // LOC100052624 // histone H2A type 1-like // --- // 100052624 /// ENSECAT	LOC100052624	<0.05	-11.0312
15055631	---		<0.05	-11.0744
15029956	---		<0.05	-11.0774
14934714	---		<0.05	-11.1017
15011590	---		<0.05	-11.1183
15136368	---		<0.05	-11.1494
14981433	---		<0.05	-11.2217
15057108	---		<0.05	-11.2319
14982053	---		<0.05	-11.2916
15133239	---		<0.05	-11.3045
15026624	---		<0.05	-11.3386
15015661	---		<0.05	-11.3752
15014012	---		<0.05	-11.4103
15030285	XM_003363755 // LOC100629501 // olfactory receptor 2W1-like // --- // 100629501 /// ENS	LOC100629501	<0.05	-11.4432
14945591	---		<0.05	-11.4986
15129526	---		<0.05	-11.5022
15076479	XM_001489227 // SEMA3C // sema domain, immunoglobulin domain (Ig), short basic domain,	SEMA3C	<0.05	-11.5196
15137046	---		<0.05	-11.5565
15128302	---		<0.05	-11.5748
15136312	---		<0.05	-11.5755
14986175	---		<0.05	-11.63
14962344	---		<0.05	-11.6532
15136958	---		<0.05	-11.7001
15120682	---		<0.05	-11.7021
14934934	---		<0.05	-11.7342
15137608	---		<0.05	-11.7469
14945248	---		<0.05	-11.7834
15095352	---		<0.05	-11.7863
15120725	---		<0.05	-11.8124
15128306	---		<0.05	-11.8212
15077680	---		<0.05	-11.8639
15106867	---		<0.05	-11.883
15136798	---		<0.05	-11.923
14986928	---		<0.05	-11.9384
15129744	---		<0.05	-11.9658
15081996	---		<0.05	-12.0043
14987283	---		<0.05	-12.0545
15136978	---		<0.05	-12.1102
14943919	---		<0.05	-12.2044
14936831	---		<0.05	-12.2348
15137328	---		<0.05	-12.2747
14962196	---		<0.05	-12.3211
15136494	---		<0.05	-12.3273
15077189	---		<0.05	-12.357
15127303	---		<0.05	-12.362
15081440	---		<0.05	-12.3633
15137740	---		<0.05	-12.4339
15116856	XM_003365564 // SERPINB2 // serpin peptidase inhibitor, clade B (ovalbumin), member 2 /	SERPINB2	<0.05	-12.5161
14979733	---		<0.05	-12.5955

15107853	---		<0.05	-12.5985
15070812	---		<0.05	-12.634
15031964	---		<0.05	-12.6654
15029693	---		<0.05	-12.67
15136558	---		<0.05	-12.67
15077630	---		<0.05	-12.7092
15137092	---		<0.05	-12.7134
15101493	---		<0.05	-12.7499
15136774	---		<0.05	-12.7733
15137338	---		<0.05	-12.7785
14928003	---		<0.05	-12.8005
14934917	---		<0.05	-12.8454
15114127	---		<0.05	-12.8503
15006335	---		<0.05	-12.8522
15136364	---		<0.05	-12.8522
15080682	ENSECAT00000023984 // INHBA // inhibin, beta A // --- // 100034076 /// NM_001081909 //	INHBA	<0.05	-12.8563
15063181	---		<0.05	-12.865
15053644	XR_036256 // LOC100068798 // calponin-3-like // --- // 100068798 /// XM_001490827 // LO	LOC100068798	<0.05	-12.8677
15006938	---		<0.05	-12.8778
15033037	---		<0.05	-12.9188
14927755	---		<0.05	-12.9437
14949316	---		<0.05	-12.9467
14927167	---		<0.05	-13.0055
15054865	---		<0.05	-13.0417
15136722	---		<0.05	-13.0417
15136390	---		<0.05	-13.0457
15003625	---		<0.05	-13.0727
15045573	---		<0.05	-13.1091
15057372	---		<0.05	-13.1106
15129919	---		<0.05	-13.1292
15060545	---		<0.05	-13.2365
14937073	---		<0.05	-13.2641
15019934	---		<0.05	-13.3434
15014984	---		<0.05	-13.3484
14946299	---		<0.05	-13.3576
15136876	---		<0.05	-13.3913
15008985	---		<0.05	-13.4618
15128327	---		<0.05	-13.4813
15030305	---		<0.05	-13.5114
15128934	---		<0.05	-13.5188
14929431	---		<0.05	-13.5294
15109357	ENSECAT00000025715 // MMP1 // matrix metallopeptidase 1 (interstitial collagenase) // -	MMP1	<0.05	-13.5932
15001469	---		<0.05	-13.6178
15071093	ENSECAT00000013283 // CXCL6 // chemokine (C-X-C motif) ligand 6 (granulocyte chemotacti	CXCL6	<0.05	-13.6482
14948030	---		<0.05	-13.6582
15137280	---		<0.05	-13.6852
15054737	---		<0.05	-13.7121
15044653	---		<0.05	-13.7904
15015913	---		<0.05	-13.8098
14926777	---		<0.05	-13.8383
14973166	---		<0.05	-13.8526
15079137	---		<0.05	-13.915
15005672	---		<0.05	-13.9337
15060218	NM_001081780 // LUM // lumican // --- // 100009681 /// ENSECAT00000019349 // LUM // lum	LUM	<0.05	-13.935
14995453	---		<0.05	-13.959
15136224	---		<0.05	-13.9847
15110227	---		<0.05	-14.0682
15068623	---		<0.05	-14.1376
15121666	---		<0.05	-14.2476
14966384	---		<0.05	-14.3153
14940355	XM_001488406 // LOC100052638 // granzyme B-like // --- // 100052638 /// ENSECAT00000008	LOC100052638	<0.05	-14.4536
15126829	---		<0.05	-14.4662
15137750	---		<0.05	-14.4959
15011494	---		<0.05	-14.5218
15077402	---		<0.05	-14.555
14939195	---		<0.05	-14.5698
15054918	---		<0.05	-14.5802
14945347	---		<0.05	-14.7454
15121695	---		<0.05	-14.8225
15128857	---		<0.05	-14.966
15136466	---		<0.05	-14.9687
15132461	---		<0.05	-15.1515
15077411	---		<0.05	-15.1611
15136862	---		<0.05	-15.2483
15062898	---		<0.05	-15.2624
15137382	---		<0.05	-15.2918
15137596	---		<0.05	-15.2918
15137766	---		<0.05	-15.2999
15128427	---		<0.05	-15.3044
14932853	---		<0.05	-15.433
15137438	---		<0.05	-15.433
15062932	---		<0.05	-15.4685
15006034	---		<0.05	-15.6644
15054877	---		<0.05	-15.7366
14953056	---		<0.05	-15.7403
15029587	---		<0.05	-15.771
14956069	---		<0.05	-15.8166
14998899	---		<0.05	-15.8931
15133022	---		<0.05	-15.9749
15068686	---		<0.05	-16.0166
15003583	---		<0.05	-16.0171
15132692	---		<0.05	-16.0431
14957420	---		<0.05	-16.0549
15130203	---		<0.05	-16.2028
14981220	---		<0.05	-16.2115
15088030	---		<0.05	-16.318
14981953	ENSECAT00000019198 // ADAM19 // ADAM metallopeptidase domain 19 // --- // ---	ADAM19	<0.05	-16.3272
15067949	---		<0.05	-16.3686
15078648	---		<0.05	-16.6413
15096212	---		<0.05	-16.6892
15021779	---		<0.05	-16.771
15132014	---		<0.05	-16.9077
14987063	---		<0.05	-17.0099
14965860	ENSECAT00000008953 // CSF3 // colony stimulating factor 3 (granulocyte) // --- // 10003	CSF3	<0.05	-17.0285
15132754	---		<0.05	-17.0311
15136740	---		<0.05	-17.0521

15133394	---		<0.05	-17.0556
14972192	---		<0.05	-17.1074
15131131	---		<0.05	-17.1132
15137618	---		<0.05	-17.16
14957006	---		<0.05	-17.1707
15063592	---		<0.05	-17.1957
15020762	---		<0.05	-17.2581
14970532	---		<0.05	-17.2951
15136360	---		<0.05	-17.3386
15137394	---		<0.05	-17.4174
15136446	---		<0.05	-17.4933
15136374	---		<0.05	-17.5034
15136754	---		<0.05	-17.5074
15002525	---		<0.05	-17.5505
15127535	---		<0.05	-17.5681
15082473	---		<0.05	-17.6968
15115696	---		<0.05	-17.7049
15128311	---		<0.05	-17.7146
15117833	---		<0.05	-17.727
15018759	---		<0.05	-17.8395
14956620	---		<0.05	-17.8451
14972820	XM_001497247 // LOC100067132 // olfactory receptor 476-like // --- // 100067132 /// ENS	LOC100067132	<0.05	-17.8474
14984939	---		<0.05	-17.8527
15032277	---		<0.05	-17.8782
14925807	---		<0.05	-17.9532
14925837	---		<0.05	-17.9532
14925879	---		<0.05	-17.9532
14925951	---		<0.05	-17.9532
14978864	---		<0.05	-18.0059
15137146	---		<0.05	-18.007
15082809	---		<0.05	-18.1827
15132482	---		<0.05	-18.2294
15067568	---		<0.05	-18.2673
15020350	---		<0.05	-18.2685
14994367	---		<0.05	-18.3086
15136226	---		<0.05	-18.3086
15117290	---		<0.05	-18.4039
15101307	---		<0.05	-18.4126
15136286	---		<0.05	-18.4182
15026537	---		<0.05	-18.4198
15129022	---		<0.05	-18.5343
14947605	---		<0.05	-18.5416
15136472	---		<0.05	-18.5454
14995193	---		<0.05	-18.5635
15078277	---		<0.05	-18.5838
15029101	---		<0.05	-18.6205
14936458	---		<0.05	-18.6637
15045035	---		<0.05	-18.677
15124574	---		<0.05	-18.7169
14986730	---		<0.05	-18.8457
15136564	---		<0.05	-18.8782
15042777	---		<0.05	-18.9303
14959143	---		<0.05	-19.0141
15130707	---		<0.05	-19.2377
15136302	---		<0.05	-19.2564
15067799	---		<0.05	-19.4071
15136210	---		<0.05	-19.4734
15051455	---		<0.05	-19.4757
14943132	---		<0.05	-19.567
15053642	---		<0.05	-19.9222
14940359	XM_001488426 // LOC100052689 // granzyme B-like // --- // 100052689 /// ENSECAT00000009	LOC100052689	<0.05	-19.9253
15130705	---		<0.05	-19.9706
14992455	---		<0.05	-19.9856
15136206	---		<0.05	-19.9856
14984009	---		<0.05	-19.9861
15137736	---		<0.05	-19.9861
15092644	---		<0.05	-19.9962
15088797	---		<0.05	-20.068
15137694	---		<0.05	-20.078
14945595	---		<0.05	-20.1176
15081326	---		<0.05	-20.253
15070665	---		<0.05	-20.3069
15077744	---		<0.05	-20.5505
14981259	---		<0.05	-20.5628
15136398	---		<0.05	-20.5731
15137488	---		<0.05	-20.6121
15136990	---		<0.05	-20.6359
15137038	---		<0.05	-20.7475
15013715	---		<0.05	-20.9782
15095850	---		<0.05	-21.0675
15131874	---		<0.05	-21.2817
15130280	---		<0.05	-21.4392
15137376	---		<0.05	-21.4701
15009301	---		<0.05	-21.5584
15132186	---		<0.05	-21.6506
15081319	---		<0.05	-21.6663
15073950	---		<0.05	-21.7265
15006243	---		<0.05	-21.7552
15006991	---		<0.05	-21.8189
15081224	---		<0.05	-21.873
14994624	---		<0.05	-21.8924
15136260	---		<0.05	-22.0647
15136808	---		<0.05	-22.0968
14997932	---		<0.05	-22.1161
14951445	---		<0.05	-22.1351
14956174	---		<0.05	-22.2277
15121381	---		<0.05	-22.2542
15131450	---		<0.05	-22.2625
15137370	---		<0.05	-22.3584
15136846	---		<0.05	-22.3896
15104019	---		<0.05	-22.4938
15133141	---		<0.05	-22.5699
15137422	---		<0.05	-22.5763
15067039	---		<0.05	-22.5821
14952507	---		<0.05	-22.6912
15047659	---		<0.05	-22.7562

15104015	---		<0.05	-22.7969
15136580	---		<0.05	-23.1692
15013552	---		<0.05	-23.2454
15071332	---		<0.05	-23.2671
14978556	---		<0.05	-23.3349
15136930	---		<0.05	-23.3383
15091433	---		<0.05	-23.5413
14975354	AF508034 // PAI-1 // plasminogen activator inhibitor-1 // --- // 100033931	PAI-1	<0.05	-23.8273
15128853	---		<0.05	-24.1031
15052447	---		<0.05	-24.1896
15035006	---		<0.05	-24.1938
15002933	---		<0.05	-24.2487
15003532	---		<0.05	-24.3102
14930777	---		<0.05	-24.5117
15136434	---		<0.05	-24.7339
15025136	---		<0.05	-24.7365
15137522	---		<0.05	-24.8593
15066549	---		<0.05	-25.1399
15066331	---		<0.05	-25.1991
14927037	---		<0.05	-25.2551
15000483	---		<0.05	-25.2714
15011434	ENSECAT00000004554 // PTX3 // pentraxin 3, long // --- // ---	PTX3	<0.05	-25.3722
15067000	---		<0.05	-25.8976
15021588	---		<0.05	-25.9139
15097038	---		<0.05	-25.9746
15137450	---		<0.05	-26.0121
15075331	---		<0.05	-26.0917
15024544	---		<0.05	-26.1531
14943439	---		<0.05	-26.813
15136662	---		<0.05	-26.9099
15003542	---		<0.05	-26.9591
15137700	---		<0.05	-26.9642
15047220	---		<0.05	-27.28
15136458	---		<0.05	-27.4124
15115746	---		<0.05	-27.4148
15022133	---		<0.05	-27.6343
15019959	---		<0.05	-27.7861
15136532	---		<0.05	-27.9585
15001446	---		<0.05	-28.1817
15136292	---		<0.05	-28.1817
15136620	---		<0.05	-28.2968
15136790	---		<0.05	-28.3046
15131847	---		<0.05	-28.5569
14949750	---		<0.05	-28.8852
15085237	---		<0.05	-28.9337
15137490	---		<0.05	-29.0469
15136244	---		<0.05	-29.1084
14935042			<0.05	-29.2099
15136584	---		<0.05	-29.3036
14939913	XM_003363631 // LOC100629686 // ig kappa chain V-III region PC 2485/PC 4039-like // ---	LOC100629686	<0.05	-29.312
15025367	---		<0.05	-29.3716
15026893	---		<0.05	-29.4833
15081328	---		<0.05	-29.8041
15060263	---		<0.05	-29.8346
15137214	---		<0.05	-29.9268
15096136	---		<0.05	-29.931
15004469	---		<0.05	-29.9682
14938460	---		<0.05	-29.9793
15013653	---		<0.05	-30.0631
15137684	---		<0.05	-30.1908
14970624	---		<0.05	-30.2479
14943758	---		<0.05	-30.3293
15077222	---		<0.05	-30.5649
15136674	---		<0.05	-30.5955
15136770	---		<0.05	-30.8029
15137336	---		<0.05	-30.8412
15067793	---		<0.05	-31.2409
14949314	---		<0.05	-31.3397
15136442	---		<0.05	-31.7406
14943621	---		<0.05	-32.1469
15128313	---		<0.05	-32.2533
14927251	---		<0.05	-32.2825
15008001	---		<0.05	-32.2965
14942831	---		<0.05	-32.4793
15136884	---		<0.05	-32.5006
14939826	---		<0.05	-32.5426
15136786	---		<0.05	-32.7815
15136478	---		<0.05	-33.1294
15137138	---		<0.05	-33.5849
15026633	---		<0.05	-33.6268
15033547	---		<0.05	-33.6459
14953577	---		<0.05	-33.6867
15115930	---		<0.05	-33.8559
15047176	---		<0.05	-34.2521
14986542	---		<0.05	-34.5596
15123572	---		<0.05	-34.7115
15031962	---		<0.05	-34.8324
15013612	---		<0.05	-34.8776
15128937	---		<0.05	-35.153
15137318	---		<0.05	-35.153
15110451	---		<0.05	-35.2867
15080685	---		<0.05	-35.702
14952251	---		<0.05	-35.9024
15137586	---		<0.05	-35.9024
15137582	---		<0.05	-36.0792
14996189	---		<0.05	-36.1282
15137460	---		<0.05	-36.4128
15132102	---		<0.05	-36.6078
15136926	---		<0.05	-37.4779
15128840	---		<0.05	-37.5234
15136666	---		<0.05	-37.6173
14935334	---		<0.05	-37.6561
15136698	---		<0.05	-37.8108
15137182	---		<0.05	-37.991
15097439	---		<0.05	-38.1454
15128145	---		<0.05	-38.3314

14969512	---		<0.05	-38.3468
15136230	---		<0.05	-38.4083
14937086	---		<0.05	-38.7106
15021111	---		<0.05	-38.751
15053588	---		<0.05	-38.7772
15007548	---		<0.05	-38.8115
14944218	---		<0.05	-39.0484
14936804	---		<0.05	-39.2676
15136530	---		<0.05	-40.011
15002538	---		<0.05	-40.1598
15026631	---		<0.05	-40.6787
14978691	---		<0.05	-40.6799
14947542	XM_001503599 // THBS1 // thrombospondin 1 // --- // 100057478 /// ENSECAT00000009707 //	THBS1	<0.05	-40.7094
14995011	---		<0.05	-41.0053
15136238	---		<0.05	-41.0053
15137326	---		<0.05	-42.4186
15126570			<0.05	-42.6344
15137436	---		<0.05	-43.1264
15137530	---		<0.05	-43.429
15129820	---		<0.05	-43.4952
15137512	---		<0.05	-44.3088
15136308	---		<0.05	-44.3743
15081696	---		<0.05	-44.4924
15137562	---		<0.05	-45.255
15088044	XM_001491449 // LOC100058506 // tissue factor-like // --- // 100058506 /// ENSECAT00000	LOC100058506	<0.05	-45.2893
15128737	---		<0.05	-45.3077
15137556	---		<0.05	-45.6471
14948800	---		<0.05	-46.3879
15136922	---		<0.05	-47.074
15137244	---		<0.05	-47.511
14997839	---		<0.05	-47.6731
14996894	---		<0.05	-48.2224
15123626	---		<0.05	-48.8002
15136502	---		<0.05	-49.0912
15042340	---		<0.05	-49.9077
15128949	---		<0.05	-50.085
15132227	---		<0.05	-50.2184
15137396	---		<0.05	-50.2184
15088688	---		<0.05	-50.5678
14943127	---		<0.05	-51.5054
15004330	---		<0.05	-51.7294
15122064	---		<0.05	-51.8703
15137288	---		<0.05	-52.1903
15137524	---		<0.05	-52.5658
15137386	---		<0.05	-53.6274
15025326	---		<0.05	-56.308
15042941	---		<0.05	-56.308
14992314	---		<0.05	-57.2961
15056875	---		<0.05	-57.3263
15128806	---		<0.05	-57.683
15137304	---		<0.05	-57.683
15137420	---		<0.05	-57.893
15065033	---		<0.05	-58.9359
15137316	---		<0.05	-59.0041
15054769	---		<0.05	-59.3197
15040330	---		<0.05	-59.991
15136282	---		<0.05	-60.5216
14992104	---		<0.05	-60.7582
14926511	---		<0.05	-61.6782
15077655	---		<0.05	-62.1591
15136898	---		<0.05	-62.1591
15111236	---		<0.05	-62.2758
15136202	---		<0.05	-62.3235
15137282	---		<0.05	-62.6104
14956238	---		<0.05	-62.9583
15065994	---		<0.05	-64.2191
15137094	---		<0.05	-67.7203
14998362	---		<0.05	-68.0454
15136264	---		<0.05	-68.0454
15137612	---		<0.05	-68.869
15009517	---		<0.05	-70.1267
15136414	---		<0.05	-70.1267
14952023	---		<0.05	-70.8349
15137574	---		<0.05	-70.8349
15137466	---		<0.05	-72.524
15137516	---		<0.05	-77.4462
15132417	---		<0.05	-82.3122
15136682	---		<0.05	-89.1531
14941506	---		<0.05	-89.2852
15137340	---		<0.05	-91.5812
15025365	---		<0.05	-93.9848
15136288	---		<0.05	-98.4802
14941804	---		<0.05	-130.059
15136486	---		<0.05	-178.292
15136730	---		<0.05	-184.898
15136318	---		<0.05	-200.831
15136676	---		<0.05	-242.107
