# Supplementary material for: Comparative transcriptome analysis of equine alveolar macrophages
Source: Equine Vet J. 2016 Jul 9;49(3):375–82. doi: 10.1111/evj.12584 (PMC5412682; doi:10.1111/evj.12584)
Supplement: Supplementary file 5 — Supplementary Item 2: Genelist of lipopolysaccharide‐treated alveolar macrophages. [file EVJ-49-375-s005.pdf]

Equine Veterinary Journal

Supplementary Item 2: Genelist of LPS treated AMs (in red letters are the transcripts identified by using the Affymetrix database, NetAffx (www.affymetrix.com)

Transcript ID	Gene assignment	Official Gene Symbol *(in red: information derived from NetAFFX)*	p Value	Fold change	Description
15011434	ENSECAT00000004554 // PTX3 // pentraxin 3, long // --- // ---	PTX3	<0,05	21.2984	6h LPS treated up vs 0h untreated
15010745	ENSECAT00000025375 // STAT4 // signal transducer and activator of transcription 4 // --	STAT4	<0,05	12.1101	6h LPS treated up vs 0h untreated
14976056	ENSECAT00000015671 // LOC100066317 // interleukin-27 subunit alpha-like // --- // 10006	IL27	<0,05	10.647	6h LPS treated up vs 0h untreated
15088285	XM_001493587 // GBP5 // guanylate binding protein 5 // --- // 100061762 /// ENSECAT0000	GBP5	<0,05	8.6157	6h LPS treated up vs 0h untreated
14965860	ENSECAT00000008953 // CSF3 // colony stimulating factor 3 (granulocyte) // --- // 10003	CSF3	<0,05	7.75438	6h LPS treated up vs 0h untreated
15014787	---	ncrna:snRNA	<0,05	7.42842	6h LPS treated up vs 0h untreated
15031529	ENSECAT00000016199 // LOC100066210 // CMRF35-like molecule 7-like // --- // 100066210 /	CD300LB	<0,05	6.67726	6h LPS treated up vs 0h untreated
15064310	ENSECAT00000019145 // LOC100062332 // c-C motif chemokine 22-like // --- // 100062332 /	CCL22	<0,05	6.20255	6h LPS treated up vs 0h untreated
15009571	ENSECAT00000008688 // IFIH1 // interferon induced with helicase C domain 1 // --- // 10	IFIH1	<0,05	5.51615	6h LPS treated up vs 0h untreated
15062669	NM_001111342 // IL2RA // interleukin 2 receptor, alpha // --- // 100070292 /// ENSECAT0	IL2RA	<0,05	5.09248	6h LPS treated up vs 0h untreated
14941638	XM_001498122 // LOC100068258 // caspase-7-like // --- // 100068258 /// ENSECAT000000169	CASP7	<0,05	5.08596	6h LPS treated up vs 0h untreated
15003823	ENSECAT00000022173 // MBNL2 // muscleblind-like splicing regulator 2 // --- // --- ///	MBNL2	<0,05	4.80805	6h LPS treated up vs 0h untreated
14932458	ENSECAT00000008486 // MKI67 // antigen identified by monoclonal antibody Ki-67 // --- /	MKI67	<0,05	4.68105	6h LPS treated up vs 0h untreated
14927299	---	intronic normalisation control	<0,05	4.58106	6h LPS treated up vs 0h untreated
15062095	ENSECAT00000013248 // MASTL // microtubule associated serine/threonine kinase-like // -	MASTL	<0,05	4.56881	6h LPS treated up vs 0h untreated
15024680	XM_001496658 // LOC100066364 // ubiquitin-like protein ISG15-like // --- // 100066364 /	ISG15	<0,05	4.54354	6h LPS treated up vs 0h untreated
15070556	XM_001915080 // HERC5 // HECT and RLD domain containing E3 ubiquitin protein ligase 5 /	HERC5	<0,05	4.53092	6h LPS treated up vs 0h untreated
14932468	XM_001489412 // MKI67 // antigen identified by monoclonal antibody Ki-67 // --- // 1000	MKI67	<0,05	4.5084	6h LPS treated up vs 0h untreated
15007054	XM_001497253 // LASS6 // ceramide synthase 6 // --- // 100052305 /// ENSECAT00000020702	LASS6	<0,05	4.4782	6h LPS treated up vs 0h untreated
15012275	ENSECAT00000024871 // LOC100068279 // uncharacterized LOC100068279 // --- // 100068279	LOC100068279	<0,05	4.30006	6h LPS treated up vs 0h untreated
14995783	XM_001490400 // ARHGEF3 // Rho guanine nucleotide exchange factor (GEF) 3 // --- // 100	ARHGEF3	<0,05	4.24659	6h LPS treated up vs 0h untreated
14999300	ENSECAT00000023515 // ITPR1 // inositol 1,4,5-trisphosphate receptor, type 1 // --- //	ITPR1	<0,05	4.19797	6h LPS treated up vs 0h untreated
15107741	XM_001504918 // AMPD3 // adenosine monophosphate deaminase 3 // --- // 100055812 /// EN	AMPD3	<0,05	4.17472	6h LPS treated up vs 0h untreated
15004722	XM_001915424 // STARD13 // StAR-related lipid transfer (START) domain containing 13 //	STARD13	<0,05	4.15431	6h LPS treated up vs 0h untreated
15090605	ENSECAT00000013398 // LOC100058797 // SLAM family member 7-like // --- // 100058797 ///	SLAMF7	<0,05	3.97985	6h LPS treated up vs 0h untreated
15025944	ENSECAT00000010490 // LOC100063623 // heat shock 70 kDa protein 4L-like // --- // 10006	HSPA4L	<0,05	3.90117	6h LPS treated up vs 0h untreated
15077930	NM_001082496 // IL6 // interleukin 6 (interferon, beta 2) // --- // 100034196 /// ENSEC	IL6	<0,05	3.88815	6h LPS treated up vs 0h untreated
15020907	ENSECAT00000011304 // LOC100072294 // TNFAIP3-interacting protein 3-like // --- // 1000	TNIP3	<0,05	3.86298	6h LPS treated up vs 0h untreated
15056863	XM_001490681 // LOC100050681 // indoleamine 2,3-dioxygenase 1-like // --- // 100050681	IDO1	<0,05	3.84531	6h LPS treated up vs 0h untreated
15071093	ENSECAT00000013283 // CXCL6 // chemokine (C-X-C motif) ligand 6 (granulocyte chemotacti	CXCL6	<0,05	3.83294	6h LPS treated up vs 0h untreated
15039165	ENSECAT00000016226 // RIN2 // Ras and Rab interactor 2 // --- // ---	RIN2	<0,05	3.77088	6h LPS treated up vs 0h untreated
15135534	---	server error	<0,05	3.7501	6h LPS treated up vs 0h untreated
15101861	ENSECAT00000021874 // LOC100059624 // protein FAM186B-like // --- // 100059624 /// XM_0	FAM186B	<0,05	3.71437	6h LPS treated up vs 0h untreated
14986665	NM_001164063 // TSLP // thymic stromal lymphopoietin // --- // 100302635 /// ENSECAT000	TSLP	<0,05	3.67319	6h LPS treated up vs 0h untreated
14960140	ENSECAT00000012002 // DHX58 // DEXH (Asp-Glu-X-His) box polypeptide 58 // --- // ---	DHX58	<0,05	3.66283	6h LPS treated up vs 0h untreated
15028337	XM_001500225 // PIM1 // pim-1 oncogene // --- // 100053898 /// ENSECAT00000021930 // PI	PIM1	<0,05	3.60544	6h LPS treated up vs 0h untreated
15085806	XM_001915323 // LOC100057148 // interferon-inducible protein AIM2-like // --- // 100057	AIM2	<0,05	3.58716	6h LPS treated up vs 0h untreated
15040476	ENSECAT00000021759 // LOC100070934 // syndecan-4-like // --- // 100070934 /// XM_001500	SDC4	<0,05	3.58249	6h LPS treated up vs 0h untreated
14991995	ENSECAT00000025409 // IL1A // interleukin 1, alpha // --- // 100064969 /// NM_001082500	IL1A	<0,05	3.57645	6h LPS treated up vs 0h untreated
15031827	XM_001502577 // CLIC5 // chloride intracellular channel 5 // --- // 100055661 /// XM_00	CLIC5	<0,05	3.56439	6h LPS treated up vs 0h untreated
15126729	agrin-like (LOC100066334)	AGRN	<0,05	3.54987	6h LPS treated up vs 0h untreated
14996357	XM_001497081 // UBA7 // ubiquitin-like modifier activating enzyme 7 // --- // 100052809	UBA7	<0,05	3.48924	6h LPS treated up vs 0h untreated
15026820	ENSECAT00000012106 // EDN1 // endothelin 1 // --- // 100034060 /// NM_001163964 // EDN1	EDN1	<0,05	3.48891	6h LPS treated up vs 0h untreated
15090521	XM_001503814 // LOC100066144 // beta-1,4-galactosyltransferase 3-like // --- // 1000661	B4GaLT1	<0,05	3.45945	6h LPS treated up vs 0h untreated
15113406	NM_001081897 // ADORA2A // adenosine A2a receptor // --- // 100034039 /// ENSECAT000000	ADORA2A	<0,05	3.44998	6h LPS treated up vs 0h untreated
15001015	ENSECAT00000007282 // TTC21A // tetratricopeptide repeat domain 21A // --- // 100068431	TTC21A	<0,05	3.39419	6h LPS treated up vs 0h untreated
15078788	XM_001501658 // LOC100071801 // tetraspanin-33-like // --- // 100071801 /// ENSECAT0000	TSPAN33	<0,05	3.37837	6h LPS treated up vs 0h untreated
15025340	ENSECAT00000007816 // RAPGEF2 // Rap guanine nucleotide exchange factor (GEF) 2 // ---	RAPGEF2	<0,05	3.37765	6h LPS treated up vs 0h untreated
15017252	XM_001499925 // LOC100070269 // MARCKS-related protein-like // --- // 100070269 /// ENS	MARCKSL1	<0,05	3.35269	6h LPS treated up vs 0h untreated
14997412	XM_003363101 // LOC100054875 // TPR and ankyrin repeat-containing protein 1-like // ---	hCG2042887	<0,05	3.35195	6h LPS treated up vs 0h untreated
15027607	ENSECAT00000002048 // TNF // tumor necrosis factor // --- // 100033834 /// NM_001081819	TNF	<0,05	3.33386	6h LPS treated up vs 0h untreated
15072244	XM_001498785 // LOC100068959 // ADP-ribosyl cyclase 1-like // --- // 100068959 /// ENSE	CD38	<0,05	3.32044	6h LPS treated up vs 0h untreated
15052407	ENSECAT00000024562 // LOC100067589 // n-acetyllactosaminide alpha-1,3-galactosyltransfe	A3GALT	<0,05	3.31214	6h LPS treated up vs 0h untreated
15014775	XM_001916804 // PARP15 // poly (ADP-ribose) polymerase family, member 15 // --- // 1000	PARP15	<0,05	3.26386	6h LPS treated up vs 0h untreated
14945006	XM_001503166 // LOC100053929 // interferon-stimulated gene 20 kDa protein-like // --- /	ISG20	<0,05	3.21177	6h LPS treated up vs 0h untreated
15131305	ENSECAT00000026113 // LOC100629405 // transmembrane protein 47-like // --- // 100629405	TMEM47	<0,05	3.19285	6h LPS treated up vs 0h untreated
15098203	XM_001916981 // HELB // helicase (DNA) B // --- // 100058069 /// ENSECAT00000007687 //	HELB	<0,05	3.18387	6h LPS treated up vs 0h untreated
15015272	ENSECAT00000020241 // ALCAM // activated leukocyte cell adhesion molecule // --- // 100	ALCAM	<0,05	3.17569	6h LPS treated up vs 0h untreated
14999580	ENSECAT00000022729 // PXK // PX domain containing serine/threonine kinase // --- // 100	PXK	<0,05	3.12488	6h LPS treated up vs 0h untreated
15082178	XM_001500985 // NT5C3 // 5'-nucleotidase, cytosolic III // --- // 100055382 /// ENSECAT	NT5C3	<0,05	3.10472	6h LPS treated up vs 0h untreated
14927283	---	Intronic normalization control	<0,05	3.08807	6h LPS treated up vs 0h untreated
15093536	ENSECAT00000005160 // SGIP1 // SH3-domain GRB2-like (endophilin) interacting protein 1	SGIP1	<0,05	3.05699	6h LPS treated up vs 0h untreated
15123213	ENSECAT00000006134 // LOC100061076 // retinol dehydrogenase 10-like // --- // 100061076	RDH10	<0,05	3.05417	6h LPS treated up vs 0h untreated
15089938	ENSECAT00000007693 // LOC100055982 // protein Niban-like // --- // 100055982	FAM129A	<0,05	3.04513	6h LPS treated up vs 0h untreated

15057441	NM_001081798 // TLR3 // toll-like receptor 3 // --- // 100009703 /// ENSECAT00000000204	TLR3	<0,05	3.02161 6h LPS treated up vs 0h untreated
15020084	ENSECAT00000025859 // DDX60L // DEAD (Asp-Glu-Ala-Asp) box polypeptide 60-like // --- /	DDX60L	<0,05	3.01504 6h LPS treated up vs 0h untreated
14927305	---	Intronic normalization control	<0,05	3.01463 6h LPS treated up vs 0h untreated
14955254	ENSECAT00000019105 // LOC100147304 // l-amino-acid oxidase-like // --- // 100147304 ///	LOXL	<0,05	2.99467 6h LPS treated up vs 0h untreated
15088814	ENSECAT00000020662 // C1orf141 // chromosome 1 open reading frame 141 // --- // ---	C1orf141	<0,05	2.98896 6h LPS treated up vs 0h untreated
15029016	ENSECAT00000009130 // ENPP4 // ectonucleotide pyrophosphatase/phosphodiesterase 4 (puta	ENPP4	<0,05	2.98884 6h LPS treated up vs 0h untreated
14995340	ENSECAT00000017447 // FRMD4B // FERM domain containing 4B // --- // 100063494 /// XM_00	FRMD4B	<0,05	2.98669 6h LPS treated up vs 0h untreated
14927505	---	Intronic normalization control	<0,05	2.98019 6h LPS treated up vs 0h untreated
15005409	XM_001496646 // KLF12 // Kruppel-like factor 12 // --- // 100066348 /// ENSECAT00000013	KLF12	<0,05	2.96813 6h LPS treated up vs 0h untreated
15002294	XM_001489171 // LOC100050217 // p2Y purinoceptor 13-like // --- // 100050217 /// ENSECA	P2RY13	<0,05	2.94403 6h LPS treated up vs 0h untreated
14940837	NM_001127352 // PTGER2 // prostaglandin E receptor 2 (subtype EP2), 53kDa // --- // 100	PTGER2	<0,05	2.91721 6h LPS treated up vs 0h untreated
14927511	---	Intronic normalization control	<0,05	2.90657 6h LPS treated up vs 0h untreated
14951498	XM_001497344 // LOC100062735 // n-formyl peptide receptor 2-like // --- // 100062735 //	FPR2	<0,05	2.90079 6h LPS treated up vs 0h untreated
14999992	ENSECAT00000020460 // PHF7 // PHD finger protein 7 // --- // 100051872 /// XM_001915336	PHF7	<0,05	2.86444 6h LPS treated up vs 0h untreated
15071077	FJ469975 // MIP-2BETA // CXCL3 // --- // 100056258 /// NM_001143793 // MIP-2BETA // CXC	MIP-2BETA	<0,05	2.85587 6h LPS treated up vs 0h untreated
14927287	---	Intronic normalization control	<0,05	2.84886 6h LPS treated up vs 0h untreated
15026012	ENSECAT00000014299 // LOC100063739 // protein sprouty homolog 1-like // --- // 10006373	SPRY1	<0,05	2.84698 6h LPS treated up vs 0h untreated
15124336	ENSECAT00000024988 // EXT1 // exostosin 1 // --- // 100056983	EXT1	<0,05	2.84566 6h LPS treated up vs 0h untreated
14989542	ENSECAT00000022076 // PNPT1 // polyribonucleotide nucleotidyltransferase 1 // --- // 10	PNPT1	<0,05	2.83148 6h LPS treated up vs 0h untreated
15078825	ENSECAT00000025427 // LOC100071840 // protein FAM40B-like // --- // 100071840	STRIP2	<0,05	2.79925 6h LPS treated up vs 0h untreated
15099547	ENSECAT00000000410 // USP18 // ubiquitin specific peptidase 18 // --- // 100054488 ///	USP18	<0,05	2.77495 6h LPS treated up vs 0h untreated
15006746	ENSECAT00000012505 // TSG-6 // tumor necrosis factor alpha-induced protein 6 // --- //	TSG-6	<0,05	2.7515 6h LPS treated up vs 0h untreated
15042300	XM_001498209 // IFT74 // intraflagellar transport 74 homolog (Chlamydomonas) // --- //	IFT74	<0,05	2.74151 6h LPS treated up vs 0h untreated
15048752	ENSECAT00000019048 // LOC100064733 // tudor domain-containing protein 7-like // --- //	TDRD7	<0,05	2.72777 6h LPS treated up vs 0h untreated
15042730	XM_003363984 // DAPK1 // death-associated protein kinase 1 // --- // 100061567 /// ENSE	DAPK1	<0,05	2.72707 6h LPS treated up vs 0h untreated
15092673	ENSECAT00000006182 // LOC100050614 // hippocampus abundant transcript 1 protein-like //	HIAT1	<0,05	2.70184 6h LPS treated up vs 0h untreated
14933154	ENSECAT00000025256 // OBFC1 // oligonucleotide/oligosaccharide-binding fold containing	OBFC1	<0,05	2.68367 6h LPS treated up vs 0h untreated
14972800	XM_001498309 // LOC100066866 // ubiquitin/ISG15-conjugating enzyme E2 L6-like // --- //	UBE2L6	<0,05	2.68332 6h LPS treated up vs 0h untreated
15001999	ENSECAT00000025637 // LOC100062640 // putative glycerol kinase 5-like // --- // 1000626	GK5	<0,05	2.67734 6h LPS treated up vs 0h untreated
15085246	XM_001488889 // LOC100053960 // basic leucine zipper transcriptional factor ATF-like 3-	BATF3	<0,05	2.6642 6h LPS treated up vs 0h untreated
15099134	---	SP110	<0,05	2.66332 6h LPS treated up vs 0h untreated
15093235	ENSECAT00000009160 // LOC100053034 // interferon-induced protein 44-like // --- // 1000	IFIH1	<0,05	2.65989 6h LPS treated up vs 0h untreated
15027108	ENSECAT00000026293 // LOC100052917 // tripartite motif-containing protein 38-like // --	TRIM38	<0,05	2.64657 6h LPS treated up vs 0h untreated
15116792	XM_001915101 // PHLPP1 // PH domain and leucine rich repeat protein phosphatase 1 // --	PHLPP1	<0,05	2.64222 6h LPS treated up vs 0h untreated
14927303	---	Intronic normalization control	<0,05	2.62594 6h LPS treated up vs 0h untreated
15002158	XM_001492309 // LOC100051043 // phospholipid scramblase 2-like // --- // 100051043 ///	PLSCR2	<0,05	2.61209 6h LPS treated up vs 0h untreated
15077669	XM_001495201 // LOC100064230 // glucocorticoid-induced transcript 1 protein-like // ---	GLCCI1	<0,05	2.60872 6h LPS treated up vs 0h untreated
15039788	XM_001498301 // LOC100068461 // forkhead box protein S1-like // --- // 100068461 /// EN	FOXS1	<0,05	2.60638 6h LPS treated up vs 0h untreated
15053690	ENSECAT00000024147 // LOC100147361 // ubiquitin carboxyl-terminal hydrolase 25-like //	USP25	<0,05	2.58797 6h LPS treated up vs 0h untreated
15071771	ENSECAT00000014115 // LIMCH1 // LIM and calponin homology domains 1 // --- // 100063373	LIMCH1	<0,05	2.58409 6h LPS treated up vs 0h untreated
15004028	ENSECAT00000021044 // TNFSF13B // tumor necrosis factor (ligand) superfamily, member 13	TNFSF13B	<0,05	2.57504 6h LPS treated up vs 0h untreated
15122715	ENSECAT00000015748 // GSDMD // gasdermin D // --- // ---	GSDMD	<0,05	2.56986 6h LPS treated up vs 0h untreated
15041928	XM_001492663 // LOC100051487 // tyrosine-protein kinase JAK2-like // --- // 100051487 /	JAK2	<0,05	2.56883 6h LPS treated up vs 0h untreated
15077811	XM_001497221 // ITGB8 // integrin, beta 8 // --- // 100053462 /// ENSECAT00000020530 //	ITGB8	<0,05	2.55911 6h LPS treated up vs 0h untreated
15004168	ENSECAT00000018395 // MCF2L // MCF.2 cell line derived transforming sequence-like // --	MCF2L	<0,05	2.55818 6h LPS treated up vs 0h untreated
15051507	XM_001495095 // LOC100064095 // tripartite motif-containing protein 14-like // --- // 1	TRIM14	<0,05	2.55557 6h LPS treated up vs 0h untreated
14942041	ENSECAT00000021368 // NFKB // nuclear factor of kappa light polypeptide gene enhancer i	NFKB	<0,05	2.55553 6h LPS treated up vs 0h untreated
15031349	ENSECAT00000015230 // LOC100063827 // transcription factor ETV7-like // --- // 10006382	ETV7	<0,05	2.55344 6h LPS treated up vs 0h untreated
15052916	NM_001081935 // PTGES // prostaglandin E synthase // --- // 100034143 /// ENSECAT000000	PTGES	<0,05	2.55166 6h LPS treated up vs 0h untreated
14958362	ENSECAT00000028931 // SOCS3 // suppressor of cytokine signaling 3 // --- // 100050730 /	SOCS3	<0,05	2.54775 6h LPS treated up vs 0h untreated
14964112	---	RNF213-201	<0,05	2.5474 6h LPS treated up vs 0h untreated
15134804	---	Server error	<0,05	2.5327 6h LPS treated up vs 0h untreated
14927263	---	Intronic normalization control	<0,05	2.52193 6h LPS treated up vs 0h untreated
15111156	ENSECAT00000014969 // ICAM1 // intercellular adhesion molecule 1 // --- // ---	ICAM1	<0,05	2.51711 6h LPS treated up vs 0h untreated
14936622	XM_001487956 // LOC100049938 // bcl-2-related protein A1-like // --- // 100049938 /// E		<0,05	2.49914 6h LPS treated up vs 0h untreated
15126730	agrin-like (LOC100066334)	AGRN	<0,05	2.48772 6h LPS treated up vs 0h untreated
14927285	---	Intronic normalization control	<0,05	2.48722 6h LPS treated up vs 0h untreated
15073643	XM_001493567 // NEK7 // NIMA (never in mitosis gene a)-related kinase 7 // --- // 10006	NEK7	<0,05	2.48376 6h LPS treated up vs 0h untreated
14927289	---	Intronic normalization control	<0,05	2.47403 6h LPS treated up vs 0h untreated
14968155	ENSECAT00000026927 // LOC100072742 // f-box only protein 39-like // --- // 100072742 //	FBXO39	<0,05	2.47164 6h LPS treated up vs 0h untreated
15118424	NM_001081757 // OAS3 // 2'-5'-oligoadenylate synthetase 3, 100kDa // --- // 791225 ///	OAS3	<0,05	2.46756 6h LPS treated up vs 0h untreated
14974973	XM_001497712 // LOC100067741 // uncharacterized LOC100067741 // --- // 100067741 /// EN	LOC100067741	<0,05	2.46087 6h LPS treated up vs 0h untreated
15135080	---	server error	<0,05	2.45819 6h LPS treated up vs 0h untreated
15126646		OAS1	<0,05	2.44752 6h LPS treated up vs 0h untreated
14943690	XM_001498209 // IFT74 // intraflagellar transport 74 homolog (Chlamydomonas) // --- //	IFT74	<0,05	2.44415 6h LPS treated up vs 0h untreated
15126645		OAS1	<0,05	2.44153 6h LPS treated up vs 0h untreated
14995322	ENSECAT00000009612 // FOXP1 // forkhead box P1 // --- // 100053199 /// XM_003363048 //	FOXP1	<0,05	2.43876 6h LPS treated up vs 0h untreated

15092177	XM_001499121 // MOV10 // Mov10, Moloney leukemia virus 10, homolog (mouse) // --- // 10	MOV10	<0,05	2.42589	6h LPS treated up vs 0h untreated
15089212	ENSECAT00000009338 // IL10 // interleukin 10 // --- // 100034187 /// NM_001082490 // IL	IL10	<0,05	2.41699	6h LPS treated up vs 0h untreated
15000485	ENSECAT00000011071 // LOC100063767 // three prime repair exonuclease 1-like // --- // 1	TREX1	<0,05	2.41554	6h LPS treated up vs 0h untreated
14939007	ENSECAT00000018492 // LOC100071046 // EH domain-containing protein 4-like // --- // 100	EHD4	<0,05	2.4101	6h LPS treated up vs 0h untreated
15107064	XM_001496305 // FCHSD2 // FCH and double SH3 domains 2 // --- // 100065828 /// ENSECAT0	FCHSD2	<0,05	2.40257	6h LPS treated up vs 0h untreated
14975351	NM_001257087 // TRIM56 // tripartite motif containing 56 // --- // 100060136 /// ENSECA	TRIM56	<0,05	2.38897	6h LPS treated up vs 0h untreated
15114130	XM_001488377 // LOC100052508 // 54 kDa 2'-5'-oligoadenylate synthase-like protein 2-lik	OAS2	<0,05	2.38382	6h LPS treated up vs 0h untreated
14969876	---	UCHL5	<0,05	2.38006	6h LPS treated up vs 0h untreated
15134772	---	server error	<0,05	2.37559	6h LPS treated up vs 0h untreated
15080843	ENSECAT00000019802 // MYO1G // myosin IG // --- // 100052077	MYO1G	<0,05	2.36945	6h LPS treated up vs 0h untreated
14927509	---	Intronic normalization control	<0,05	2.36462	6h LPS treated up vs 0h untreated
15066609	XM_001915658 // RASGEF1B // RasGEF domain family, member 1B // --- // 100146797 /// ENS	RASGEF1B	<0,05	2.34918	6h LPS treated up vs 0h untreated
15098090	ENSECAT00000012670 // SRGAP1 // SLIT-ROBO Rho GTPase activating protein 1 // --- // 100	SRGAP1	<0,05	2.34424	6h LPS treated up vs 0h untreated
14945899	ENSECAT00000006078 // PML // promyelocytic leukemia // --- // 100062744 /// XM_00149324	PML	<0,05	2.33403	6h LPS treated up vs 0h untreated
15020540	ENSECAT00000025178 // OTUD4 // OTU domain containing 4 // --- // 100070999 /// XM_00150	OTUD4	<0,05	2.3282	6h LPS treated up vs 0h untreated
15070957	XM_001491674 // LOC100051425 // cyclin-G2-like // --- // 100051425 /// ENSECAT000000151	CCNG2	<0,05	2.32705	6h LPS treated up vs 0h untreated
14943011	ENSECAT00000000977 // SLC16A9 // solute carrier family 16, member 9 (monocarboxylic aci	SLC16A9	<0,05	2.32546	6h LPS treated up vs 0h untreated
15030361	ENSECAT00000017994 // TRIM26 // tripartite motif containing 26 // --- // 100051726 ///	TRIM26	<0,05	2.32091	6h LPS treated up vs 0h untreated
15105518	ENSECAT00000023734 // APLP2 // amyloid beta (A4) precursor-like protein 2 // --- // 100	APLP2	<0,05	2.31706	6h LPS treated up vs 0h untreated
15054457	ENSECAT00000025586 // TRPM2 // transient receptor potential cation channel, subfamily M	TRPM2	<0,05	2.31084	6h LPS treated up vs 0h untreated
15012672	XM_001500006 // HEG1 // HEG homolog 1 (zebrafish) // --- // 100070351 /// ENSECAT000000	HEG1	<0,05	2.31056	6h LPS treated up vs 0h untreated
15070754	XM_001915383 // CDS1 // CDP-diacylglycerol synthase (phosphatidate cytidylyltransferase	CDS1	<0,05	2.30686	6h LPS treated up vs 0h untreated
15114137	NM_001081797 // OASL // 2'-5'-oligoadenylate synthetase-like // --- // 100009702 /// EN	OASL	<0,05	2.30237	6h LPS treated up vs 0h untreated
14954367	XM_001501644 // LOC100065387 // carcinoembryonic antigen-related cell adhesion molecule	CEACAM1	<0,05	2.29995	6h LPS treated up vs 0h untreated
14957111	XM_001504045 // TRAF3IP2 // TRAF3 interacting protein 2 // --- // 100066812 /// ENSECAT	TRAF3IP2	<0,05	2.29906	6h LPS treated up vs 0h untreated
15041225	---	HELZ2-201	<0,05	2.28108	6h LPS treated up vs 0h untreated
15085004	ENSECAT00000022005 // RGL1 // ral guanine nucleotide dissociation stimulator-like 1 //	RGL1	<0,05	2.27802	6h LPS treated up vs 0h untreated
14927301	---	Intronic normalization control	<0,05	2.26701	6h LPS treated up vs 0h untreated
15096318	ENSECAT00000009829 // LRMP // lymphoid-restricted membrane protein // --- // 100068768	LRMP	<0,05	2.26077	6h LPS treated up vs 0h untreated
15047758	XM_001496017 // FOXN3 // forkhead box N3 // --- // 100052973 /// ENSECAT00000029020 //	FOXN3	<0,05	2.25344	6h LPS treated up vs 0h untreated
15071082	---	CXCL3	<0,05	2.24871	6h LPS treated up vs 0h untreated
15043736	NM_001099440 // IFNB1 // interferon, beta 1, fibroblast // --- // 100052545	IFNB1	<0,05	2.23857	6h LPS treated up vs 0h untreated
14927261	---	Intronic normalization control	<0,05	2.23488	6h LPS treated up vs 0h untreated
15081436	NM_001256925 // SAMD9 // sterile alpha motif domain containing 9 // --- // 100051216 //	SAMD9	<0,05	2.2337	6h LPS treated up vs 0h untreated
15022470	XM_001498965 // LOC100069173 // ribonuclease ZC3H12A-like // --- // 100069173 /// ENSEC	ZC3H12A	<0,05	2.23156	6h LPS treated up vs 0h untreated
15012340	ENSECAT00000015618 // CCDC50 // coiled-coil domain containing 50 // --- // ---	CCDC50	<0,05	2.23147	6h LPS treated up vs 0h untreated
15116856	XM_003365564 // SERPINB2 // serpin peptidase inhibitor, clade B (ovalbumin), member 2 /	SERPINB2	<0,05	2.22603	6h LPS treated up vs 0h untreated
14966186	ENSECAT00000016804 // LOC100069660 // guanine nucleotide-binding protein G(I)/G(S)/G(O)	GNGT2	<0,05	2.22421	6h LPS treated up vs 0h untreated
15013994	XM_001495276 // GNB4 // guanine nucleotide binding protein (G protein), beta polypeptid	GNB4	<0,05	2.22047	6h LPS treated up vs 0h untreated
15014789	ENSECAT00000009985 // LOC100060632 // e3 ubiquitin-protein ligase DTX3L-like // --- //	DTX3L	<0,05	2.20388	6h LPS treated up vs 0h untreated
15108349	XM_001502780 // LOC100072747 // transmembrane gamma-carboxyglutamic acid protein 4-like	PRRG4	<0,05	2.18858	6h LPS treated up vs 0h untreated
15108822	ENSECAT00000022192 // RFX2 // regulatory factor X, 2 (influences HLA class II expressio	RFX2	<0,05	2.18822	6h LPS treated up vs 0h untreated
15069675	XM_001498568 // LOC100068748 // mixed lineage kinase domain-like protein-like // --- //	MLKL	<0,05	2.17659	6h LPS treated up vs 0h untreated
15090753	---	IFI16	<0,05	2.1721	6h LPS treated up vs 0h untreated
15128810	ENSECAT00000003070 // LOC100072611 // putative P2Y purinoceptor 10-like // --- // 10007	P2RY10	<0,05	2.1657	6h LPS treated up vs 0h untreated
15061584	XM_001488742 // CERK // ceramide kinase // --- // 100053604 /// ENSECAT00000005822 // C	CERK	<0,05	2.15171	6h LPS treated up vs 0h untreated
15131515	XM_001917800 // SLC9A7 // solute carrier family 9, subfamily A (NHE7, cation proton ant	SLC9A7	<0,05	2.15089	6h LPS treated up vs 0h untreated
14969784	XM_001490897 // LOC100051253 // oxysterols receptor LXR-alpha-like // --- // 100051253	LXR-a	<0,05	2.15072	6h LPS treated up vs 0h untreated
15079967	ENSECAT00000023822 // NUB1 // negative regulator of ubiquitin-like proteins 1 // --- //	NUB1	<0,05	2.14782	6h LPS treated up vs 0h untreated
15134318	---	server error	<0,05	2.1403	6h LPS treated up vs 0h untreated
15104100	XM_001916286 // BIRC2 // baculoviral IAP repeat containing 2 // --- // 100061273 /// EN	BIRC2	<0,05	2.13557	6h LPS treated up vs 0h untreated
15058331	ENSECAT00000026768 // APAF1 // apoptotic peptidase activating factor 1 // --- // 100052	APAF1	<0,05	2.12972	6h LPS treated up vs 0h untreated
14960466	XM_001500159 // SMARCE1 // SWI/SNF related, matrix associated, actin dependent regulato	SMARCE1	<0,05	2.12603	6h LPS treated up vs 0h untreated
14950558	XM_003362328 // PVRL2 // poliovirus receptor-related 2 (herpesvirus entry mediator B) /	PVRL2	<0,05	2.12582	6h LPS treated up vs 0h untreated
15038286	NM_001081902 // CD40 // CD40 molecule, TNF receptor superfamily member 5 // --- // 1000	CD40	<0,05	2.12287	6h LPS treated up vs 0h untreated
15112614	XM_001500797 // DENND5A // DENN/MADD domain containing 5A // --- // 100071112 /// ENSEC	DENND5A	<0,05	2.12277	6h LPS treated up vs 0h untreated
14990274	ENSECAT00000015187 // LOC100054688 // xanthine dehydrogenase/oxidase-like // --- // 100	XDH	<0,05	2.11953	6h LPS treated up vs 0h untreated
14937807	ENSECAT00000026465 // MYO1E // myosin IE // --- // 100068162	MYO1E	<0,05	2.11603	6h LPS treated up vs 0h untreated
15107397	XM_003365380 // LOC100054490 // tripartite motif-containing protein 5-like // --- // 10	TRIM5	<0,05	2.10442	6h LPS treated up vs 0h untreated
14997779	XM_001494698 // RFTN1 // raftlin, lipid raft linker 1 // --- // 100063495 /// ENSECAT00	RFTN1	<0,05	2.10286	6h LPS treated up vs 0h untreated
15126728	XM_001917428 // LOC100066334 // agrin-like // --- // 100066334 /// ENSECAT00000012181 /	AGR	<0,05	2.10101	6h LPS treated up vs 0h untreated
15027038	ENSECAT00000021472 // LOC100629805 // acyl-coenzyme A thioesterase 13-like // --- // 10	ACOT13	<0,05	2.101	6h LPS treated up vs 0h untreated
15054174	XM_001491467 // MX2 // myxovirus (influenza virus) resistance 2 (mouse) // --- // 10005	MX2	<0,05	2.10037	6h LPS treated up vs 0h untreated
14927277	---	Intronic normalization control	<0,05	2.09666	6h LPS treated up vs 0h untreated
15075633	XM_001916048 // LOC100067364 // transcription factor HIVEP2-like // --- // 100067364 //	HIVEP2	<0,05	2.0951	6h LPS treated up vs 0h untreated
15118881	XM_001493015 // LOC100060900 // RILP-like protein 2-like // --- // 100060900 /// ENSECA	RILPL2	<0,05	2.08838	6h LPS treated up vs 0h untreated
15101868	ENSECAT00000007057 // FMNL3 // formin-like 3 // --- // 100059687 /// XM_001492200 // FM	FMNL3	<0,05	2.08206	6h LPS treated up vs 0h untreated

15134794	---	server error	<0,05	2.07957	6h LPS treated up vs 0h untreated
14974104	XM_001492289 // CHKA // choline kinase alpha // --- // 100059839 /// ENSECAT00000012200	CHKA	<0,05	2.06411	6h LPS treated up vs 0h untreated
14972150	ENSECAT00000016926 // LOC100067133 // excitatory amino acid transporter 2-like // --- /	SLC7A5P2	<0,05	2.06138	6h LPS treated up vs 0h untreated
15053920	ENSECAT00000014380 // LOC100062926 // intersectin-1-like // --- // 100062926	ITSN1	<0,05	2.06087	6h LPS treated up vs 0h untreated
15086428	ENSECAT00000002639 // ADAR // adenosine deaminase, RNA-specific // --- // ---	ADAR	<0,05	2.057	6h LPS treated up vs 0h untreated
15082491	ENSECAT00000008802 // LOC100071408 // protein FAM3C-like // --- // 100071408 /// XM_001	FAM3C	<0,05	2.05462	6h LPS treated up vs 0h untreated
15066307	ENSECAT00000005398 // PPM1K // protein phosphatase, Mg2+/Mn2+ dependent, 1K // --- // 1	PPM1K	<0,05	2.05354	6h LPS treated up vs 0h untreated
15076386	ENSECAT00000000196 // LOC100066798 // syntaxin-11-like // --- // 100066798 /// XM_00150	STX11	<0,05	2.0489	6h LPS treated up vs 0h untreated
15065381	ENSECAT00000011105 // LOC100055378 // telomeric repeat-binding factor 2-interacting pro	TERF2IP	<0,05	2.04694	6h LPS treated up vs 0h untreated
14953223	ENSECAT00000023472 // LOC100067512 // a-kinase anchor protein 7 isoform gamma-like // -	AKAP7	<0,05	2.04252	6h LPS treated up vs 0h untreated
14966919	ENSECAT00000018440 // SLFN5 // schlafen family member 5 // --- // 100071635	SLFN5	<0,05	2.03586	6h LPS treated up vs 0h untreated
15128819	XM_001501384 // GPR174 // G protein-coupled receptor 174 // --- // 100071745 /// ENSECA	GPR174	<0,05	2.03504	6h LPS treated up vs 0h untreated
15026884	ENSECAT00000024669 // LOC100065723 // GMP reductase 1-like // --- // 100065723 /// XM_0	GMPR	<0,05	2.02472	6h LPS treated up vs 0h untreated
14955063	XM_001489167 // PLEKHA4 // pleckstrin homology domain containing, family A (phosphoinos	PLEKHA4	<0,05	2.02303	6h LPS treated up vs 0h untreated
14933789	ENSECAT00000016733 // BLNK // B-cell linker // --- // 100070841	BLNK	<0,05	2.02151	6h LPS treated up vs 0h untreated
15025773	XM_001915633 // GAB1 // GRB2-associated binding protein 1 // --- // 100062924 /// ENSEC	GAB1	<0,05	2.02083	6h LPS treated up vs 0h untreated
15038468	XM_001488269 // PTPN1 // protein tyrosine phosphatase, non-receptor type 1 // --- // 10	PTPN1	<0,05	2.02059	6h LPS treated up vs 0h untreated
15130048	ENSECAT00000007861 // DDX26B // DEAD/H (Asp-Glu-Ala-Asp/His) box polypeptide 26B // ---	DDX26B	<0,05	2.02018	6h LPS treated up vs 0h untreated
15124307	XM_001496042 // TRPS1 // trichorhinophalangeal syndrome I // --- // 100056821 /// ENSEC	TRPS1	<0,05	2.01117	6h LPS treated up vs 0h untreated
15126656		IL1B	<0,05	2.00708	6h LPS treated up vs 0h untreated
15098031	ENSECAT00000024728 // USP15 // ubiquitin specific peptidase 15 // --- // 100055857	USP15	<0,05	2.00621	6h LPS treated up vs 0h untreated
14969486	ENSECAT00000021443 // PRR5L // proline rich 5 like // --- // 100052234 /// XM_001488316	PRR5L	<0,05	2.00438	6h LPS treated up vs 0h untreated
14954767	XM_001917363 // LOC100146913 // neutral amino acid transporter B(0)-like // --- // 1001	SLC1A5	<0,05	-2.00761	6h LPS treated down vs 0h untreated
14957654	XM_003362372 // LOC100073151 // serine/threonine-protein kinase Sgk1-like // --- // 100	Sgk1	<0,05	-2.02089	6h LPS treated down vs 0h untreated
14945010	ENSECAT00000021485 // AEN // apoptosis enhancing nuclease // --- // ---	AEN	<0,05	-2.06747	6h LPS treated down vs 0h untreated
14971846	XM_001917602 // LOC100146758 // patatin-like phospholipase domain-containing protein 2-	PNPLA2	<0,05	-2.07838	6h LPS treated down vs 0h untreated
15068182	XM_001499798 // LOC100070117 // wolframin-like // --- // 100070117 /// ENSECAT000000137	WFS1	<0,05	-2.08333	6h LPS treated down vs 0h untreated
14967281	ENSECAT00000006927 // SLC46A1 // solute carrier family 46 (folate transporter), member	SLC46A1	<0,05	-2.12738	6h LPS treated down vs 0h untreated
15026677	XM_001490360 // RIOK1 // RIO kinase 1 (yeast) // --- // 100050980 /// ENSECAT0000000957	RIOK1	<0,05	-2.14655	6h LPS treated down vs 0h untreated
14932187	---	Intronic normalization control	<0,05	-2.25422	6h LPS treated down vs 0h untreated
15064847	XM_001498913 // PLA2G15 // phospholipase A2, group XV // --- // 100053908 /// ENSECAT00	PLA2G15	<0,05	-2.34886	6h LPS treated down vs 0h untreated
15126975	NM_001081771 // TLR7 // toll-like receptor 7 // --- // 791248 /// ENSECAT00000007908 //	TLR7	<0,05	-2.38297	6h LPS treated down vs 0h untreated
15095766	ENSECAT00000001427 // PTPN6 // protein tyrosine phosphatase, non-receptor type 6 // ---	PTPN6	<0,05	-2.45451	6h LPS treated down vs 0h untreated
14972866	ENSECAT00000000688 // LPXN // leupaxin // --- // 100066577 /// XM_001504694 // LPXN //	LPXN	<0,05	-2.46798	6h LPS treated down vs 0h untreated
15106911	ENSECAT00000019345 // ARRB1 // arrestin, beta 1 // --- // 100064657	ARRB1	<0,05	-2.47078	6h LPS treated down vs 0h untreated
15094354	ENSECAT00000006171 // LOC100056612 // 2-acylglycerol O-acyltransferase 1-like // --- //	MOGAT1	<0,05	-2.59006	6h LPS treated down vs 0h untreated
15073546	ENSECAT00000012594 // RGS2 // regulator of G-protein signaling 2, 24kDa // --- // 10005	RGS2	<0,05	-2.61257	6h LPS treated down vs 0h untreated
15071057	XM_001490281 // LOC100056605 // proepiregulin-like // --- // 100056605 /// ENSECAT00000	EREG	<0,05	-2.7328	6h LPS treated down vs 0h untreated
14998968	ENSECAT00000017857 // LOC100051258 // peroxisome proliferator-activated receptor gamma-	PPARG	<0,05	-2.88641	6h LPS treated down vs 0h untreated
15032122	ENSECAT00000018852 // GCLC // glutamate-cysteine ligase, catalytic subunit // --- // 10	GCLC	<0,05	-2.90054	6h LPS treated down vs 0h untreated
15124723	ENSECAT00000025889 // LOC100068961 // src-like-adapter-like // --- // 100068961 /// XM_	SLA	<0,05	-3.12657	6h LPS treated down vs 0h untreated
15015651	XM_001488028 // LOC100049896 // lipid phosphate phosphohydrolase 3-like // --- // 10004	PPAP2BL	<0,05	-4.17601	6h LPS treated down vs 0h untreated
